# Association of Long Noncoding RNA Biomarkers With Clinical Immune Subtype and Prediction of Immunotherapy Response in Patients With Cancer

**DOI:** 10.1001/jamanetworkopen.2020.2149

**Published:** 2020-04-07

**Authors:** Yunfang Yu, Wenda Zhang, Anlin Li, Yongjian Chen, Qiyun Ou, Zifan He, Yiwen Zhang, Ruixin Liu, Herui Yao, Erwei Song

**Affiliations:** 1 Department of Medical Oncology, Guangdong Provincial Key Laboratory of Malignant Tumor Epigenetics and Gene Regulation, Sun Yat-sen Memorial Hospital, Sun Yat-sen University, Guangzhou, China; 2 Guangdong Medical University, Zhanjiang, China; 3 Department of Medical Oncology, Third Affiliated Hospital of Sun Yat-sen University, Guangzhou, China; 4 Breast Tumor Centre, Guangdong Provincial Key Laboratory of Malignant Tumor Epigenetics and Gene Regulation, Sun Yat-sen Memorial Hospital, Sun Yat-sen University, Guangzhou, China

## Abstract

**Importance:**

Long noncoding RNAs (lncRNAs) are involved in innate and adaptive immunity in cancer by mediating the functional state of immunologic cells, pathways, and genes. However, whether lncRNAs are associated with immune molecular classification and clinical outcomes of cancer immunotherapy is largely unknown.

**Objectives:**

To explore lncRNA-based immune subtypes associated with survival and response to cancer immunotherapy and to present a novel lncRNA score for immunotherapy prediction using computational algorithms.

**Design, Setting, and Participants:**

In this cohort study, an individual patient analysis based on a phase 2, single-arm clinical trial and multicohort was performed from June 25 through September 30, 2019. Data are from the phase 2 IMvigor210 trial and from The Cancer Genome Atlas (TCGA). The study analyzed lncRNA and genomic data of 348 patients with bladder cancer from the IMvigor210 trial and 71 patients with melanoma from TCGA who were treated with immunotherapy. In addition, a pancancer multicohort that included 2951 patients was obtained from TCGA.

**Main Outcomes and Measures:**

The primary end point was overall survival (OS).

**Results:**

Among 348 patients from the IMvigor210 trial (272 [78.2%] male) and 71 patients with melanoma from TCGA (mean [SD] age, 58.3 [13.4] years; 37 [52.1%] female), 4 distinct classes with statistically significant differences in OS (median months, not reached vs 9.6 vs 8.1 vs 6.7 months; *P* = .002) were identified. The greatest OS benefit was obtained in the immune-active class, as characterized by the immune-functional lncRNA signature and high CTL infiltration. Patients with low vs high lncRNA scores had statistically significantly longer OS (hazard ratio, 0.32; 95% CI, 0.24-0.42; *P* < .001) in the IMvigor210 trial and across various cancer types. The lncRNA score was associated with immunotherapeutic OS benefit in the IMvigor210 trial cohort (area under the curve [AUC], 0.79 at 12 months and 0.77 at 20 months) and in TCGA melanoma cohort (AUC, 0.87 at 24 months), superior tumor alteration burden, programmed cell death ligand 1 (PD-L1) expression, and cytotoxic T-lymphocyte (CTL) infiltration. Addition of the lncRNA score to the combination of tumor alteration burden, PD-L1 expression, and CTL infiltration to build a novel multiomics algorithm correlated more strongly with OS in the IMvigor210 trial cohort (AUC, 0.81 at 12 months and 0.80 at 20 months).

**Conclusions and Relevance:**

This study identifies novel lncRNA-based immune classes in cancer immunotherapy and recommends immunotherapy for patients in the immune-active class. In addition, the study recommends that the lncRNA score should be integrated into multiomic panels for precision immunotherapy.

## Key Points

**Question:**

Are long noncoding RNAs (lncRNAs) associated with immune molecular classification and clinical outcomes of cancer immunotherapy?

**Findings:**

This cohort study of 348 patients with bladder cancer from the IMvigor210 trial and 71 patients with melanoma from The Cancer Genome Analysis identified 4 distinct classes with different immunotherapeutic overall survival and response. An lncRNA score was developed that also was associated with survival and immunotherapy response.

**Meaning:**

This study identifies novel lncRNA-based immune classes in cancer immunotherapy and provides an lncRNA score for integration into multiomic panels for precision immunotherapy.

## Teaser

This cohort study explores long noncoding RNA–based immune subtypes associated with survival and response to cancer immunotherapy and presents a novel long noncoding RNA score for immunotherapy prediction using computational algorithms.

## Introduction

Previous studies^1,2,3^ that investigated the interaction between long noncoding RNAs (lncRNAs) biology and immunomicroenvironment components demonstrated that high nuclear factor (NF)-κB–interacting lncRNA (*NKILA* [Ensembl ENSG00000278709]) in tumor-specific cytotoxic T lymphocytes (CTLs) or in tumor cells and high hypoxia-inducible factor 1α–stabilizing lncRNA in tumor-associated macrophages indicated a poor prognosis. However, it is largely unknown whether lncRNA could serve as an effective biomarker for cancer immunotherapy. Herein, we present the first study to date, to our knowledge, to identify lncRNA-based immune subtypes associated with survival and response to clinical cancer immunotherapy. Furthermore, a novel lncRNA score is presented that could be integrated with tumor alteration burden, programmed cell death ligand 1 (PD-L1) expression, and CTL infiltration for precision immunotherapy.

## Methods

### Patients and Study Design

**Figure 1.**
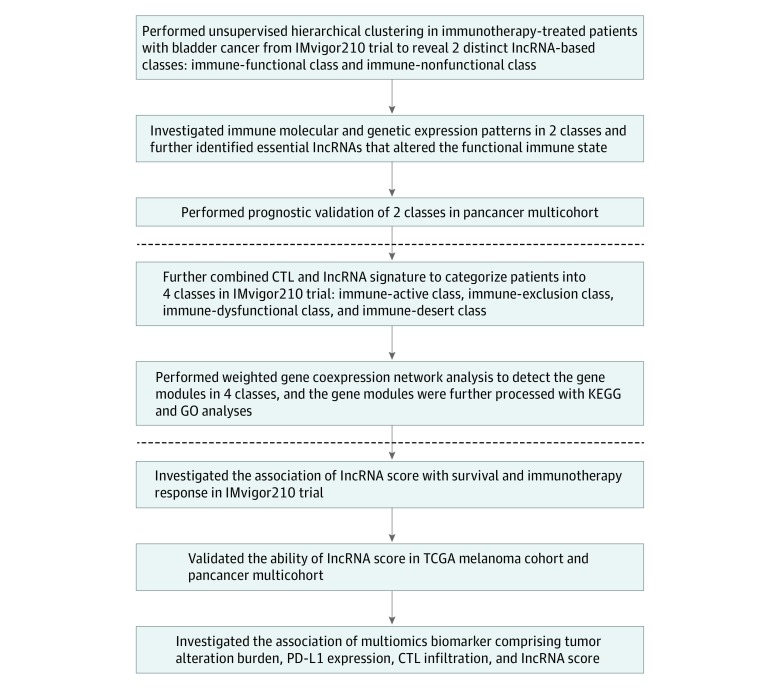
Overview of Study Design CTL indicates cytotoxic T-lymphocyte; GO, gene ontology; KEGG, Kyoto Encyclopedia of Genes and Genomes; lncRNA, long noncoding RNA; PD-L1, programmed cell death ligand 1; and TCGA, The Cancer Genome Atlas.

In this cohort study, an individual patient-level analysis was performed among 3370 patients using validated lncRNA and genomic data according to the Transparent Reporting of a Multivariable Prediction Model for Individual Prognosis or Diagnosis (TRIPOD) guideline.^4^ The overall study design is shown in Figure 1. The study was conducted from June 25 through September 30, 2019, using data from the single-arm, phase 2, multicenter IMvigor210 trial and from The Cancer Genome Atlas (TCGA). A total of 419 patients received immunotherapy, including 348 patients with bladder cancer treated with the PD-L1 inhibitor atezolizumab from the IMvigor210 trial^5,6,7,8^ and 71 patients with melanoma treated by various immunotherapeutic strategies, such as anti-PD-1, anti-cytotoxic T-lymphocyte–associated protein 4 (CTLA4), and cytokine tumor vaccine, from TCGA.^9^ In addition, a pancancer multicohort treated without immunotherapy from TCGA was included that consisted of 513 patients with lung adenocarcinoma, 493 patients with lung squamous cell carcinoma, 1082 patients with breast cancer, 406 patients with bladder cancer, and 457 patients with melanoma. Transcriptome RNA sequencing was performed in all included tumor tissues. In addition, US Food and Drug Administration–approved FoundationOne CDx next-generation sequencing was performed in the IMvigor210 trial cohort, and whole-exome sequencing was performed in TCGA melanoma cohort. The primary end point was overall survival (OS), and the secondary end points were the objective response rate (ORR) (ie, complete response plus partial response) and the disease control rate (DCR) (ie, complete response plus partial response plus stable disease). The study protocol was approved by the ethics committee of the Sun Yat-sen Memorial Hospital, Sun Yat-sen University, Guangzhou, China. The requirement for informed consent of study participants was waived by the ethics committee because the human data were obtained from publicly available data sets.

### Statistical Analysis

Statistical analyses were performed using R, version 3.6.1 (R Project for Statistical Computing). The count data were processed using the R package variance-stabilizing transformation for quality control and normalization. For lncRNAs of patients treated with immunotherapy from the IMvigor210 trial, a univariable Cox proportional hazards regression model was used to select lncRNAs with *P* < .05 for immune molecular classification. The R package CancerSubtypes was used to assess the optimal cluster number that was associated with the highest stability and the lowest ambiguity; then, unsupervised hierarchical clustering methods (K-means)^10^ were used to identify lncRNA classes. Heatmaps were generated to show the distribution and expression levels of immune molecules and immune cells across lncRNA classes. To quantify the proportions of tumor-infiltrating immune cells, single-sample gene set enrichment analysis (GSEA)^11^ and a signature containing 577 marker genes^12^ were used that are highly representative of 24 human immune cell phenotypes in the tumor microenvironment. A volcano plot was used to show differentially expressed genes between the 2 lncRNA classes with absolute log_2_ (fold change) values exceeding the estimated cutoff value of 1.11 with *P* < .05.

The R package clusterProfiler was used for gene ontology (GO), the Kyoto Encyclopedia of Genes and Genomes (KEGG) analyses, and the GSEA. The KEGG pathways and GO terms regarding cellular component, molecular function, and biological process with *P* values and false discovery rates less than .05 were considered statistically significant. The enrichment *P* values for the GSEA were based on 1000 permutations and adjusted by calculating the false discovery rates. The GSEA results were visualized using the R package enrichplot. Gene set variation analysis (GSVA) was performed using gene sets from the MSigDB database of the Broad Institute’s enrichment algorithm. The R package gsva was used to calculate the GSVA enrichment scores; then, the R package limma was applied to assess whether there was a difference between the GSVA enrichment scores and the different lncRNA classes. Genes with SDs exceeding 1 were subjected to weighted gene coexpression network analysis to investigate the genomic heterogeneity of lncRNA classes, from which the optimal power value was selected based on the scale-free topology feature of the network.

To assess the essential candidate lncRNAs in the clustering, the R package glmnet was applied to build a penalized logistic least absolute shrinkage and selector operation (LASSO) algorithm,^13^ with penalty parameter tuning performed using a 5-fold cross-validation. Finally, the R package randomForest was used to apply the random forest classification algorithm,^14^ which numerically estimated and ranked the importance of lncRNAs. Higher mean decrease Gini values indicated greater importance.

To build a novel lncRNA score associated with survival and response benefit of immunotherapy, lncRNA data with statistical significance in the univariable Cox proportional hazards regression model were narrowed down using the LASSO algorithm,^13^ with the IMvigor210 trial used as the training cohort and TCGA melanoma cohort used as the validation cohort. Using OS as the predictor variable, this procedure was repeated 10 000 times to construct the lncRNA score weighted with corresponding LASSO coefficients. The R package timeROC was used to generate time-dependent receiver operating characteristic (ROC) curves and to calculate area under the curve (AUC) to evaluate sensitivity and specificity.^15^

Aggregate OS data were computed using the Kaplan-Meier method and were compared with the log-rank test. Survival was compared between the 2 classes using hazard ratios (HRs) and 95% CIs based on the Cox proportional hazards regression model. Categorical variables were compared with χ^2^ test or Fisher exact test, and continuous variables were compared with Wilcoxon rank sum test for 2-group comparisons or Kruskal-Wallis test for multiple comparisons. The optimal cutoff value for continuous variables was generated using the R package survminer. Matrix correlation analysis and weighted linear regression models were used to estimate the strength of the correlations with Pearson ρ. For all analyses, 2-sided *P* < .05 was considered statistically significant.

## Results

This cohort study included 348 patients from the IMvigor210 trial (272 [78.2%] male). In the IMvigor210 trial, most patients had an Eastern Cooperative Oncology Group performance status of 0 to 1 (330 [94.8%]), had received platinum-based chemotherapy (272 [78.2%]), and had progressive disease (167 [48.0%]). The median follow-up was 8.1 (interquartile range, 2.9-17.8) months. Of 3022 patients from TCGA, 71 patients with melanoma received immunotherapy (mean [SD] age, 58.3 [13.4] years; 37 [52.1%] female). Detailed characteristics of the IMvigor210 trial and TCGA bladder cancer cohort are listed in eTable 1 and eTable 2 in the Supplement.

### Association of lncRNA Biomarkers With Clinical Immune Subtype

**Figure 2.**
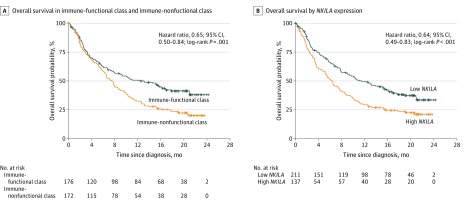
Overall Survival Analysis in 2 Long Noncoding RNA–Based Classes A, Overall survival analysis in the immune-functional class and the immune-dysfunctional class in the IMvigor210 trial. B, Overall survival analysis in patients with low nuclear factor-κB–interacting long noncoding RNA (*NKILA)* expression and high *NKILA* expression.

In the IMvigor210 trial, lncRNA interactions were comprehensively characterized and are shown in eFigure 1 in the Supplement. Forty-nine of 421 lncRNAs identified by the univariable Cox proportional hazards regression analysis were used to identify 2 distinct lncRNA-based classes associated with statistically significantly different OS benefit (HR, 0.65; 95% CI, 0.50-0.84; *P* = .001) (Figure 2A and eFigure 2 in the Supplement). Surprisingly, patients with longer survival were found to have lower expression of immune cells, immune checkpoints, and human leukocyte antigens compared with patients with shorter survival (eFigure 3 and eFigure 4 in the Supplement). We hypothesized that immune cells of patients with less survival benefit were in a nonfunctional state and thus referred to the novel lncRNA profile with favorable survival as the immune-functional class and the profile with poor survival as the immune-nonfunctional class. The GSVA showed that the immune-functional class was enriched for pathways involving DNA replication and homologous recombination, whereas the immune-nonfunctional class was enriched for pathways associated with extracellular matrix (ECM)–receptor interaction and cytokine–cytokine receptor interaction (eFigure 5 in the Supplement).

To further identify the essential lncRNAs that altered the functional immune state, the 49 lncRNAs of the 2 distinct lncRNA-based classes were subjected to LASSO and random forest analyses. The results indicated that *AC002116-2* (Ensembl ENSG00000267698), *AP000251-1* (Ensembl ENSG00000237594), *TMEM147-AS1* (Ensembl ENSG00000236144), and *NKILA* were the 4 important lncRNAs in our lncRNA clustering (eFigure 6 in the Supplement). *NKILA* was chosen for further analysis because it had been found to be critically involved in regulating CTLs in a previous study.^1^ Genes were evaluated between the 2 classes, and GSEA showed that the *NKILA*-interacted NF-κB signaling pathway was highly enriched in the immune-nonfunctional class over the immune-functional class (eFigure 7 and eFigure 8 in the Supplement). In addition, *NKILA* was found to have an enrichment pattern consistent with the immune-nonfunctional class, and it showed a positive correlation with ECM-receptor interaction (*R*^2^ = 0.45) (eFigure 9 in the Supplement). This result was further confirmed using the KEGG and GO enrichment analyses, which jointly showed that *NKILA* had high enrichment in the ECM-related pathways, biological processes, cellular components, and molecular functions (eFigure 10, eFigure 11, eTable 3, and eTable 4 in the Supplement). Moreover, Kaplan-Meier analysis indicated that among patients with bladder cancer treated with immunotherapy, low *NKILA* expression resulted in longer OS compared with high *NKILA* expression (HR, 0.64; 95% CI, 0.49-0.83; *P* < .001) (Figure 2B). As expected, *NKILA* expression was statistically significantly lower in the immune-functional class than in the immune-nonfunctional class (eFigure 12 in the Supplement). Validation of the 2 distinct lncRNA-based classes and *NKILA* in the pancancer multicohort is summarized in the eAppendix 1 and eFigure 13 and eFigure 14 in the Supplement.

### OS Benefit in the Immune-Active Class With Functional and Dense Immune Infiltration

**Figure 3.**
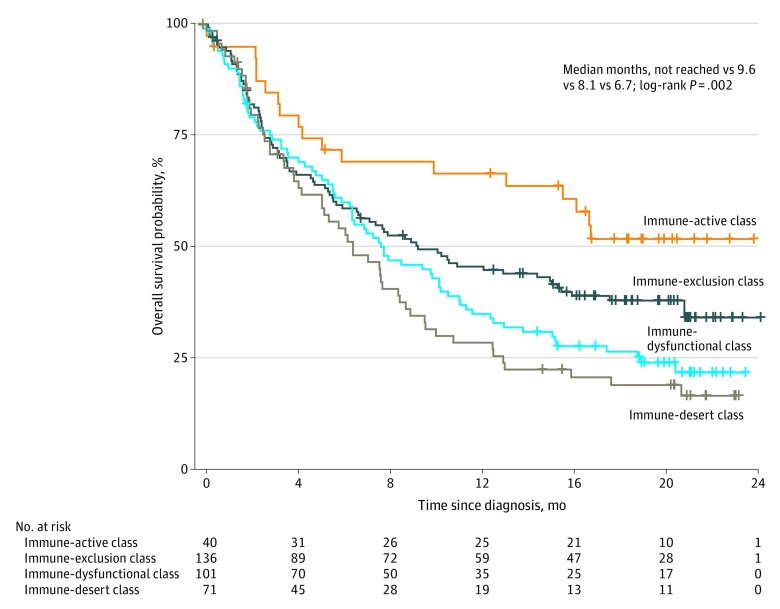
Overall Survival in 4 Classes Stratified by Long Noncoding RNAs and the Infiltration of Cytotoxic T Lymphocytes Median months of survival are listed for each of the 4 classes, respectively.

Infiltration of CTLs has been shown to be associated with survival in patients with triple-negative breast cancer receiving atezolizumab,^16^ and it was previously shown that CTLs with different lncRNA expression patterns were associated with cancer prognosis.^1^ In the present study, CTLs were associated with OS in the pancancer multicohort and correlated with multiple immune molecules (eAppendix 2, eFigure 15, and eFigure 16 in the Supplement), indicating that CTL infiltration could reflect most of the immunologic variance in the tumor microenvironment. Therefore, the lncRNA signature and CTL infiltration were combined to categorize patients with bladder cancer in the IMvigor210 trial into 4 distinct lncRNA and CTL–based classes with statistically significant differences in OS (median months, not reached vs 9.6 vs 8.1 vs 6.7; *P* = .002) (Figure 3) and ORR (41.2% vs 28.1% vs 20.0% vs 7.7%) (eFigure 17 in the Supplement).

**Figure 4.**
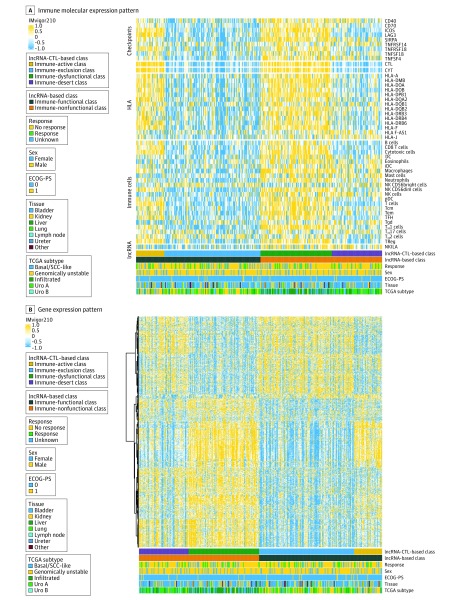
Heatmap Representation of the Immune Molecular and Gene Expression Patterns CTL indicates cytotoxic T-lymphocyte; CYT, cytolytic activity; DC, dendritic cell; ECOG-PS, Eastern Cooperative Oncology Group Performance Status; IDC, immature dendritic cell; lncRNA, long noncoding RNA; NK, natural killer; pDC, plasmacytoid dendritic cell; SCC, squamous cell carcinoma; TCGA, The Cancer Genome Atlas; Tcm, central memory T cells; Tem, effector memory T cells; TFH, T follicular helper cells; TReg, T regulatory cells; Uro A, urothelial-like A subtype; and Uro B, urothelial-like B subtype. The black lines at the y axis of panel B indicate the unsupervised hierarchical clustering of genes.

Patients with a functional immune response with high CTL infiltration had higher expression of immune molecules compared with patients with a functional immune response with low CTL infiltration, who were considered to be in the immune-active class and the immune-exclusion class, respectively. Immune-nonfunctional patients with high CTL infiltration had higher expression of immune molecules compared with immune-nonfunctional patients with low CTL infiltration, who were considered to be in the immune-dysfunctional class and the immune-desert class, respectively (Figure 4A and eFigure 18 in the Supplement). These molecular characteristics were confirmed in TCGA bladder cancer cohort (eFigure 19 in the Supplement).

In addition, using the gene modules that correlated most with each lncRNA and CTL–based class detected by weighted gene coexpression network analysis (eFigure 20 in the Supplement), it was found that distinct gene expression patterns existed among the 4 classes (Figure 4B). These classes were characterized by different functional annotations and pathways. The KEGG pathway enrichment analysis found that the immune-active class was statistically significantly involved in the cell cycle and p53 signaling pathway. The immune-exclusion class was involved in various physiological and pathological metabolism processes. The immune-dysfunctional class was involved in phagosome, primary immunodeficiency, and T_H_1 and T_H_2 cell differentiation. The immune-desert class was involved in ECM-receptor interaction and the PI3K-Akt signaling pathway (eFigure 21 and eTables 5, 6, 7, and 8 in the Supplement). The GO enrichment analysis indicated that the immune-active class had enriched genomic biological processes, such as chromatin assembly or disassembly, nucleosome assembly, DNA packaging, nucleosome organization, and DNA conformation change. The immune-exclusion class had enriched epithelial change, such as epidermis development, cornification, keratinization, and epidermal cell or keratinocyte differentiation. The immune-dysfunctional class had enriched immunologic processes, such as activation of T cells and leukocytes. The immune-desert class showed enrichment of ECM-related processes, such as ECM organization and extracellular structure organization (eFigure 22 and eTables 9, 10, 11, and 12 in the Supplement).

### Association of the lncRNA Score With Survival and Response to Cancer Immunotherapy

Given the robustness of lncRNAs in immune molecular classification, the LASSO algorithm was used to identify 29 of 49 lncRNAs to construct the lncRNA score in the IMvigor210 trial (eAppendix 3 in the Supplement). Based on the optimal threshold (eFigure 23 in the Supplement), patients with low lncRNA scores had statistically significantly greater OS (HR, 0.32; 95% CI, 0.24-0.42; *P* < .001), ORR (29.7% vs 9.7%; *P* < .001), and DCR (54.4% vs 24.3%; *P* < .001) compared with patients with high lncRNA scores (eFigures 24, 25, and 26 in the Supplement). Additional validation results for the pancancer multicohort are summarized in eAppendix 4 and eFigure 27 and eFigure 28 in the Supplement.

**Figure 5.**
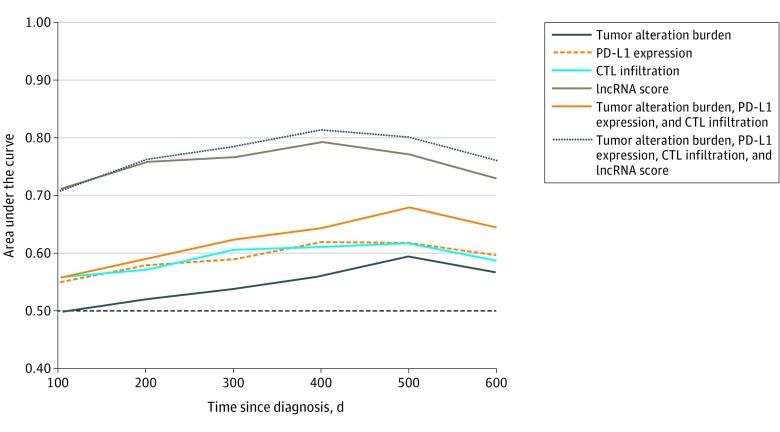
Association of the Long Noncoding RNA (lncRNA) Score and the Multiomics Biomarker With Survival and Immunotherapy Response CTL indicates cytotoxic T lymphocyte; and PD-L1, programmed cell death ligand 1.

We next compared the clinical usefulness of the lncRNA score, tumor alteration burden, PD-L1 expression, and CTL infiltration using ROC analyses (Figure 5 and eFigure 29 in the Supplement). Similarly, tumor alteration burden, PD-L1 expression, and CTL infiltration showed a modest correlation with 12-month OS (AUC, 0.55 for tumor alteration burden, 0.62 for PD-L1 expression, and 0.60 for CTL infiltration) and with 20-month OS (AUC, 0.61 for tumor alteration burden, 0.62 for PD-L1 expression, and 0.59 for CTL infiltration) in the IMvigor210 trial. Similarly, the lncRNA score was associated with immunotherapeutic OS benefit in the IMvigor210 trial cohort (AUC, 0.79 at 12 months and 0.77 at 20 months) and in TCGA melanoma cohort (AUC, 0.87 at 24 months). The ability of the lncRNA score to be used to classify patients with a response vs no response and patients with DCR vs progressive disease had an AUC of 0.72 and 0.71, respectively (eFigure 30 in the Supplement). A model was established by combining tumor alteration burden, PD-L1 expression, and CTL infiltration. However, this model did not generate substantial improvement (AUC, 0.63 at 12 months and 0.68 at 20 months) compared with using a single variable. The lncRNA score was further added to this model to build a novel multiomics algorithm, which correlated more strongly with OS in the IMvigor210 trial cohort (AUC, 0.81 at 12 months and 0.80 at 20 months) (Figure 5 and eFigure 29 in the Supplement).

## Discussion

This study first characterized lncRNA expression profiles to identify an OS advantage of the immune-functional class over the immune-nonfunctional class. In the subsequent in-depth classification that also considered CTL infiltration, patients in the immune-active class with both high density of CTLs and the immune-functional lncRNA signature had the most favorable OS and ORR benefit, whereas patients in the immune-desert class with neither of these had the worst clinical outcomes. Patients in the immune-dysfunctional class had high immune infiltration but poor OS, even worse than that among patients in the immune-exclusion class with low immune infiltration, suggesting a close interaction between dysregulated lncRNA biology and the dysfunctional state of immune cells. The 4 novel distinct classes identified in this study indicated that immune molecular classification of aspects of both immune exclusion and immune dysfunction could be informative for understanding patterns of immune escape^1^ (ie, cancers could escape immunologic destruction) and for selection of candidates for cancer immunotherapy.

The results herein identified the essential involvement of *NKILA* and its interacting NF-κB pathway in the induction of immune dysfunction to immunotherapy (ie, *NKILA*-induced dysfunctional state of immune cells), which supports the predictive and immunosuppressive role of these elements observed in previous studies.^1,3,17^ The engineering of *NKILA* (ie, targeting *NKILA* in immune cells could be a promising and feasible approach to cellular immunotherapy) might reverse the immune dysfunctional state and enhance immunotherapeutic effectiveness. Specific differences were also found in the signaling pathways that characterize each of the 4 classes, which can inform novel combination strategies, such as immunotherapy combined with targeted therapy, tumor vaccines, or cellular immunotherapy. For example, patients in the immune-active class might derive additional benefit from immunotherapy plus p53 inhibitor because the p53 signaling pathway was highly enriched in this class. In addition, the PI3K-Akt signaling pathway was highly enriched in the immune-desert class, indicating that these patients might be potential candidates for concurrent PI3K-Akt inhibition and checkpoint blockade.

Our group and others previously developed a multiomics biomarker for cancer immunotherapy combining tumor alteration burden, PD-L1 expression, and CTL infiltration.^18,19^ The present study adds the lncRNA score to this biomarker, potentially increasing and substantially improving the ability to predict immunotherapeutic benefit on OS, likely because of the additional information regarding immune dysfunction provided by the lncRNA signature. Recent work has provided a gene-based algorithm incorporating T-cell dysfunction and exclusion, which was more effective for predicting immunotherapy response compared with previous biomarkers that considered only 1 of these aspects.^20^ We recommend that factors characterizing immune cell dysfunction, such as the lncRNA score, should be integrated into multiomic panels for precision immunotherapy.

### Limitations

This study has limitations. One is the heterogeneity of populations and treatments that exists across and within cancer types. For example, patients in TCGA melanoma cohort were treated by diverse strategies, including anti-PD-1, anti-CTLA4, and cytokine tumor vaccine, which may have obscured associations between the lncRNA score and specific types of immunotherapy. Such heterogeneity might explain the small difference in response between patients with low vs high lncRNA scores in this cohort. In addition, immunotherapy was not used in some cohorts, in which case only the biomarker usefulness of the lncRNA score was validated. Therefore, the applicability of our lncRNA-based immune subtypes and algorithm to the pancancer multicohort warrants further study. Furthermore, because of a lack of available data, we were unable to further enhance the accuracy and interpretability of the multiomics biomarker by adding other information, such as neoantigen burden, single-nucleotide variants, and messenger RNA expression level.

## Conclusions

This study used signatures of lncRNAs and CTL infiltration to identify 4 distinct immune classes in clinical cancer immunotherapy and recommends immunotherapy for patients in the immune-active class with both an immune-functional lncRNA signature and dense CTL infiltration. In addition, multiomic panels composed of tumor alteration burden, PD-L1 expression, CTL infiltration, and the lncRNA score represent a useful biomarker for cancer immunotherapy.

## 

## Notes

Supplement. **eAppendix 1.** Supplemental Results 1 **eAppendix 2.** Supplemental Results 2 **eAppendix 3.** Supplemental Results 3 **eAppendix 4.** Supplemental Results 4 **eFigure 1.** Landscape of lncRNA Interactions in the IMvigor210 Trial **eFigure 2.** Unsupervised Clustering of lncRNAs for 348 Patients in the IMvigor210 Trial **eFigure 3.** Immune Molecular Expression in Immune-Functional Class Versus Immune-Nonfunctional Class in the IMvigor210 Trial **eFigure 4.** Heatmap Representation of Immune Molecules in Immune-Functional and Immune-Nonfunctional Classes in the IMvigor210 Trial **eFigure 5.** Gene Set Variation Analysis of Immune-Functional and Immune-Nonfunctional Classes in the IMvigor210 Trial **eFigure 6.** Identification of Essential lncRNAs in the Clustering in the IMvigor210 Trial **eFigure 7.** Gene Expression Analysis Between Immune-Functional and Immune-Nonfunctional Classes in the IMvigor210 Trial **eFigure 8.** Gene Set Enrichment Analysis of NF-κB Pathway Between Immune-Functional and Immune-Nonfunctional Classes **eFigure 9.** Matrix Plot Showing the Correlation of NKILA Expression With Signaling Pathways in the IMvigor210 Trial **eFigure 10.** KEGG Pathway Enrichment Analysis of NKILA in the IMvigor210 Trial **eFigure 11.** Gene Ontology Enrichment Analysis of NKILA in the IMvigor210 Trial **eFigure 12.** NKILA Expression in Immune-Functional Class Versus Immune-Nonfunctional Class in the IMvigor210 Trial **eFigure 13.** Association of lncRNA-Based Class With Overall Survival in Pancancer **eFigure 14.** Association of NKILA Expression With Overall Survival in Pancancer **eFigure 15.** Association of Cytotoxic T Lymphocytes With Overall Survival **eFigure 16.** Correlations Between Cytotoxic T Lymphocytes and Various Immune Molecules in the IMvigor210 Trial **eFigure 17.** Objective Response Rate Stratified by lncRNAs and Infiltration of Cytotoxic T Lymphocytes **eFigure 18.** Association of lncRNA-CTL-Based Class With Immune Molecular Expression in the IMvigor210 Trial **eFigure 19.** Immune Molecular Expression Pattern of lncRNA-CTL-Based Classes in TCGA Bladder Cancer Cohort **eFigure 20.** Weighted Gene Co-Expression Network Analysis of Four lncRNA-CTL-Based Classes in the IMvigor210 Trial **eFigure 21.** KEGG Pathway Enrichment Analysis of Four lncRNA-CTL-Based Classes in the IMvigor210 Trial **eFigure 22.** Gene Ontology Pathway Enrichment Analysis of Four lncRNA-CTL-Based Classes in the IMvigor210 Trial **eFigure 23.** Identification of the Optimal Cutoff for lncRNA Score **eFigure 24.** Overall Survival Stratified by lncRNA Score in the IMvigor210 Trial **eFigure 25.** Objective Response Rate Stratified by lncRNA Score in the IMvigor210 Trial **eFigure 26.** Disease Control Rate Stratified by lncRNA Score in the IMvigor210 Trial **eFigure 27.** Objective Response Rate Stratified by lncRNA Score in the Melanoma Immunotherapy Cohort **eFigure 28.** Association of lncRNA Score With Overall Survival in TCGA Cohorts **eFigure 29.** Association of lncRNA Score and Multiomics Biomarker With Overall Survival in the IMvigor210 Trial **eFigure 30.** Association of lncRNA Score With Response in the IMvigor210 Trial **eTable 1.** Characteristics and Clinical Outcomes of the Patients in IMvigor210 Trial **eTable 2.** Baseline Characteristics of the Patients in TCGA Bladder Cancer Cohort **eTable 3.** Kyoto Encyclopedia of Genes and Genomes Pathway Enrichment Analysis of NKILA in IMvigor210 Trial **eTable 4.** Gene Ontology Pathway Enrichment Analysis of NKILA in IMvigor210 Trial **eTable 5.** Kyoto Encyclopedia of Genes and Genomes Pathway Enrichment Analysis of Immune-Active Class in IMvigor210 Trial **eTable 6.** Kyoto Encyclopedia of Genes and Genomes Pathway Enrichment Analysis of Immune-Exclusion Class in IMvigor210 Trial **eTable 7.** Kyoto Encyclopedia of Genes and Genomes Pathway Enrichment Analysis of Immune-Dysfunctional Class in IMvigor210 Trial **eTable 8.** Kyoto Encyclopedia of Genes and Genomes Pathway Enrichment Analysis of Immune-Desert Class in IMvigor210 Trial **eTable 9.** Gene Ontology Pathway Enrichment Analysis of Immune-Active Class in IMvigor210 Trial **eTable 10.** Gene Ontology Pathway Enrichment Analysis of Immune-Exclusion Class in IMvigor210 Trial **eTable 11.** Gene Ontology Pathway Enrichment Analysis of Immune-Dysfunctional Class in IMvigor210 Trial **eTable 12.** Gene Ontology Pathway Enrichment Analysis of Immune-Desert Class in IMvigor210 Trial
