# Supplementary material for: Association of Long Noncoding RNA Biomarkers With Clinical Immune Subtype and Prediction of Immunotherapy Response in Patients With Cancer
Source: JAMA Netw Open. 2020 Apr 7;3(4):e202149. doi: 10.1001/jamanetworkopen.2020.2149 (PMC7139278; doi:10.1001/jamanetworkopen.2020.2149)
Supplement: Supplement. — eAppendix 1. Supplemental Results 1 eAppendix 2. Supplemental Results 2 eAppendix 3. Supplemental Results 3 eAppendix 4. Supplemental Results 4 eFigure 1. Landscape of lncRNA Interactions in the IMvigor210 Trial eFigure 2. Unsupervised Clustering of lncRNAs for 348 Patients in the IMvigor210 Trial eFigure 3. Immune Molecular Expression in Immune-Functional Class Versus Immune-Nonfunctional Class in the IMvigor210 Trial eFigure 4. Heatmap Representation of Immune Molecules in Immune-Functional and Immune-Nonfunctional Classes in the IMvigor210 Trial eFigure 5. Gene Set Variation Analysis of Immune-Functional and Immune-Nonfunctional Classes in the IMvigor210 Trial eFigure 6. Identification of Essential lncRNAs in the Clustering in the IMvigor210 Trial eFigure 7. Gene Expression Analysis Between Immune-Functional and Immune-Nonfunctional Classes in the IMvigor210 Trial eFigure 8. Gene Set Enrichment Analysis of NF-κB Pathway Between Immune-Functional and Immune-Nonfunctional Classes eFigure 9. Matrix Plot Showing the Correlation of NKILA Expression With Signaling Pathways in the IMvigor210 Trial eFigure 10. KEGG Pathway Enrichment Analysis of NKILA in the IMvigor210 Trial eFigure 11. Gene Ontology Enrichment Analysis of NKILA in the IMvigor210 Trial eFigure 12. NKILA Expression in Immune-Functional Class Versus Immune-Nonfunctional Class in the IMvigor210 Trial eFigure 13. Association of lncRNA-Based Class With Overall Survival in Pancancer eFigure 14. Association of NKILA Expression With Overall Survival in Pancancer eFigure 15. Association of Cytotoxic T Lymphocytes With Overall Survival eFigure 16. Correlations Between Cytotoxic T Lymphocytes and Various Immune Molecules in the IMvigor210 Trial eFigure 17. Objective Response Rate Stratified by lncRNAs and Infiltration of Cytotoxic T Lymphocytes eFigure 18. Association of lncRNA-CTL-Based Class With Immune Molecular Expression in the IMvigor210 Trial eFigure 19. Immune Molecular Expression Pattern of lncRNA-CTL-Base [file jamanetwopen-3-e202149-s001.pdf]

Supplementary Online Content

Yu Y, Zhang W, Li A, et al. Association of long noncoding RNA biomarkers with clinical immune subtype and prediction of immunotherapy response in patients with cancer. *JAMA Netw Open*. 2020;3(4):e202149. doi:10.1001/jamanetworkopen.2020.2149

eAppendix 1. Supplemental Results 1
eAppendix 2. Supplemental Results 2
eAppendix 3. Supplemental Results 3
eAppendix 4. Supplemental Results 4
eFigure 1. Landscape of lncRNA Interactions in the IMvigor210 Trial
eFigure 2. Unsupervised Clustering of lncRNAs for 348 Patients in the IMvigor210 Trial
eFigure 3. Immune Molecular Expression in Immune-Functional Class Versus Immune-Nonfunctional Class in the IMvigor210 Trial
eFigure 4. Heatmap Representation of Immune Molecules in Immune-Functional and Immune-Nonfunctional Classes in the IMvigor210 Trial
eFigure 5. Gene Set Variation Analysis of Immune-Functional and Immune-Nonfunctional Classes in the IMvigor210 Trial
eFigure 6. Identification of Essential lncRNAs in the Clustering in the IMvigor210 Trial
eFigure 7. Gene Expression Analysis Between Immune-Functional and Immune-Nonfunctional Classes in the IMvigor210 Trial
eFigure 8. Gene Set Enrichment Analysis of NF-κB Pathway Between Immune-Functional and Immune-Nonfunctional Classes
eFigure 9. Matrix Plot Showing the Correlation of NKILA Expression With Signaling Pathways in the IMvigor210 Trial
eFigure 10. KEGG Pathway Enrichment Analysis of NKILA in the IMvigor210 Trial
eFigure 11. Gene Ontology Enrichment Analysis of NKILA in the IMvigor210 Trial
eFigure 12. NKILA Expression in Immune-Functional Class Versus Immune-Nonfunctional Class in the IMvigor210 Trial
eFigure 13. Association of lncRNA-Based Class With Overall Survival in Pancancer
eFigure 14. Association of NKILA Expression With Overall Survival in Pancancer
eFigure 15. Association of Cytotoxic T Lymphocytes With Overall Survival
eFigure 16. Correlations Between Cytotoxic T Lymphocytes and Various Immune Molecules in the IMvigor210 Trial
eFigure 17. Objective Response Rate Stratified by lncRNAs and Infiltration of Cytotoxic T Lymphocytes
eFigure 18. Association of lncRNA-CTL-Based Class With Immune Molecular Expression in the IMvigor210 Trial
eFigure 19. Immune Molecular Expression Pattern of lncRNA-CTL-Based Classes in TCGA Bladder Cancer Cohort
eFigure 20. Weighted Gene Co-Expression Network Analysis of Four lncRNA-CTL-Based Classes in

© 2020 Yu Y et al. *JAMA Network Open.*

the IMvigor210 Trial
eFigure 21. KEGG Pathway Enrichment Analysis of Four lncRNA-CTL-Based Classes in the IMvigor210 Trial
eFigure 22. Gene Ontology Pathway Enrichment Analysis of Four lncRNA-CTL-Based Classes in the IMvigor210 Trial
eFigure 23. Identification of the Optimal Cutoff for lncRNA Score
eFigure 24. Overall Survival Stratified by lncRNA Score in the IMvigor210 Trial
eFigure 25. Objective Response Rate Stratified by lncRNA Score in the IMvigor210 Trial
eFigure 26. Disease Control Rate Stratified by lncRNA Score in the IMvigor210 Trial
eFigure 27. Objective Response Rate Stratified by lncRNA Score in the Melanoma Immunotherapy Cohort
eFigure 28. Association of lncRNA Score With Overall Survival in TCGA Cohorts
eFigure 29. Association of lncRNA Score and Multiomics Biomarker With Overall Survival in the IMvigor210 Trial
eFigure 30. Association of lncRNA Score With Response in the IMvigor210 Trial
eTable 1. Characteristics and Clinical Outcomes of the Patients in IMvigor210 Trial
eTable 2. Baseline Characteristics of the Patients in TCGA Bladder Cancer Cohort
eTable 3. Kyoto Encyclopedia of Genes and Genomes Pathway Enrichment Analysis of NKILA in IMvigor210 Trial
eTable 4. Gene Ontology Pathway Enrichment Analysis of NKILA in IMvigor210 Trial
eTable 5. Kyoto Encyclopedia of Genes and Genomes Pathway Enrichment Analysis of Immune-Active Class in IMvigor210 Trial
eTable 6. Kyoto Encyclopedia of Genes and Genomes Pathway Enrichment Analysis of Immune-Exclusion Class in IMvigor210 Trial
eTable 7. Kyoto Encyclopedia of Genes and Genomes Pathway Enrichment Analysis of Immune-Dysfunctional Class in IMvigor210 Trial
eTable 8. Kyoto Encyclopedia of Genes and Genomes Pathway Enrichment Analysis of Immune-Desert Class in IMvigor210 Trial
eTable 9. Gene Ontology Pathway Enrichment Analysis of Immune-Active Class in IMvigor210 Trial
eTable 10. Gene Ontology Pathway Enrichment Analysis of Immune-Exclusion Class in IMvigor210 Trial
eTable 11. Gene Ontology Pathway Enrichment Analysis of Immune-Dysfunctional Class in IMvigor210 Trial
eTable 12. Gene Ontology Pathway Enrichment Analysis of Immune-Desert Class in IMvigor210 Trial

This supplementary material has been provided by the authors to give readers additional information about their work.

© 2020 Yu Y et al. *JAMA Network Open.*

eAppendix 1.

The prognostic implications of two distinct long non-coding RNA (lncRNA)-based classes for overall survival (OS) were identified in lung squamous cell carcinoma (LUSC) (HR 0.75, 95% CI 0.57–0.99; *P*=.038), bladder cancer (HR 0.68, 95% CI, 0.51–0.92; *P*=.011), and melanoma (HR 0.62, 95% CI 0.47–0.81; *P*<.001) (eFigure 13 in the Supplement). High NF-κB-interacting lncRNA (NKILA) versus low NKILA was found to be prognostic of OS in lung adenocarcinoma (LUAD) (HR 0.63, 95% CI 0.45–0.88; *P*=.007) and LUSC (HR 0.66, 95% CI 0.50–0.88; *P*=.004) (eFigure 14 in the Supplement).

eAppendix 2.

High tumor specific cytotoxic T lymphocyte (CTL) versus low CTL was associated with better OS from immunotherapy in bladder cancer (HR 0.57, 95% CI 0.39-0.84; *P*=.004), better prognosis in LUAD (HR 0.74, 95% CI 0.55–0.98; *P*=.037), breast cancer (HR 0.63, 95% CI 0.46–0.86; *P*=.004), HER-2 positive breast cancer (HR 0.32, 95% CI 0.12–0.82; *P*=.013), bladder cancer (HR 0.62, 95% CI 0.45–0.86; *P*=.004), and melanoma (HR 0.52, 95% CI 0.39–0.69; *P*<.001) (eFigure 15 in the Supplement).

The CTL was closely correlated with multiple immune molecules including immune cells such as B cells, CD8 T cells, cytotoxic cells, dendritic cells, macrophages, neutrophils, T cells, natural killer cells, and type 1 T helper cells, central memory T cells, effector memory T cells, plasmacytoid dendritic cell, T follicular helper cells, and regulatory cells; immune checkpoints such as BTLA, CD160, CD226, CD274, CD28, CD70, CD80, CD86, HAVCR2, ICOS, IDO1, CTLA4, LAG3, PDCD1LG2, PVRIG, SIRPA, and TIGIT; and human leukocyte antigens such as HLA-A, HLA-B, HLA-DMA, HLA-DMB, HLA-DOA, HLA-DOB, HLA-DPA1, HLA-DPB1, HLA-DQA2, DLA-DRA, HLA-DRB1, HLA-DRB3, HLA-DRB4, HLA-DRB6, HLA-E, and HLA-F (eFigure 16 in the Supplement).

eAppendix 3.

The lncRNA score was calculated according to the following: LncRNA score = – (DLGAP1-AS1×0.0388404669) – (RAMP2-AS1×0.1155662963) – (BVES-AS1×0.0553720312) – (LIPE-AS1×0.1033882346) + (LINC01118×0.0796557151) – (FGD5-AS1×0.0289597115) – (AL513365.2×0.20586808) – (EMC1-AS1×0.0694844742) + (FTX×0.0852566063) + (SNHG15×0.1004314629) – (TMEM147-AS1×0.0720185014) + (TTN-AS1×0.0540188921) + (FLG-AS1×0.0526396975) – (CDKN2B-AS1×0.0064524961) + (USP2-AS1×0.0747066713)– (SBF2-AS1×0.1293691962) – (AC113382.1×0.2179618875) + (NOP14-AS1×0.0644565543) + (AC012636.1×0.1939511817) – (LINC01605×0.0168478032) + (PCAT1×0.1453946597) + (BRWD1-AS2×0.0298250867) – (USP3-AS1×0.0691270103) + (VPS9D1-AS1×0.0003103997) – (AC093249.6×0.0139283015) – (SNHG8×0.0014401603) – (FUT8-AS1×0.0852427371) + (NKILA×0.1318600645) – (SLFNL1-AS1×0.0472823654).

eAppendix 4.

Based on the optimal threshold (-1.41), difference in objective response rate between high lncRNA score and low lncRNA score was confirmed in melanoma immunotherapy cohort (47.8% vs 39.1%) (eFigure 27 in the Supplement). Additionally, the prognostic value of a low versus high lncRNA score was shown for LUAD (HR 0.66, 95% CI 0.49–0.90; *P*=.008), LUSC (HR 0.75, 95% CI 0.57–0.98, *P*=.036), breast cancer (HR 0.61, 95% CI 0.44–0.84; *P*=.002), HER-2 positive breast cancer (HR 0.38, 95% CI 0.15–0.97; *P*=.035), bladder cancer (HR 0.60, 95% CI 0.44–0.83; *P*=.002), and melanoma (HR 0.70, 95% CI 0.53–0.91; *P*=.007) (eFigure 28 in the Supplement).

© 2020 Yu Y et al. *JAMA Network Open.*

eFigure 1. Landscape of lncRNA interactions in IMvigor210 Trial.

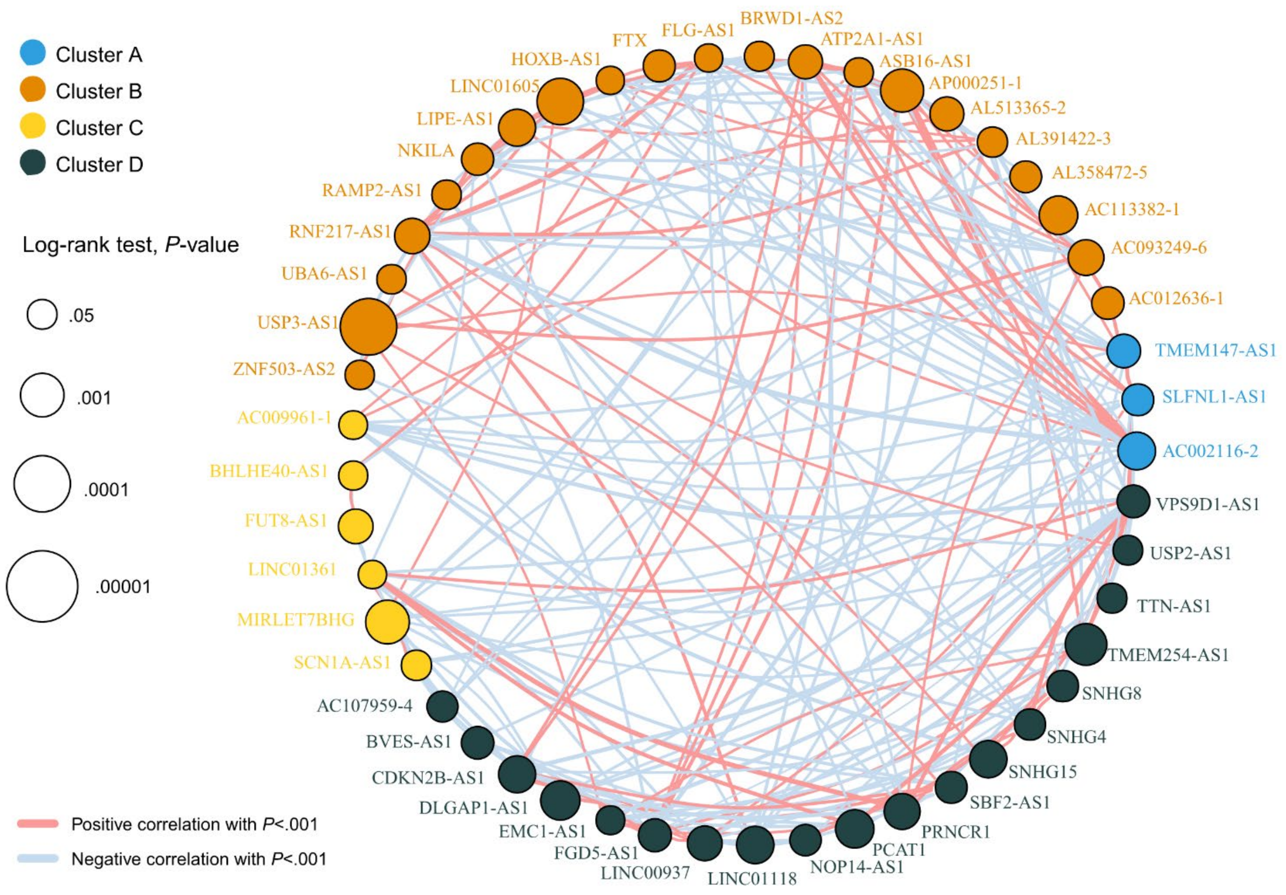

© 2020 Yu Y et al. *JAMA Network Open.*

eFigure 2. Unsupervised Clustering of lncRNAs for 348 Patients in IMvigor210 Trial.

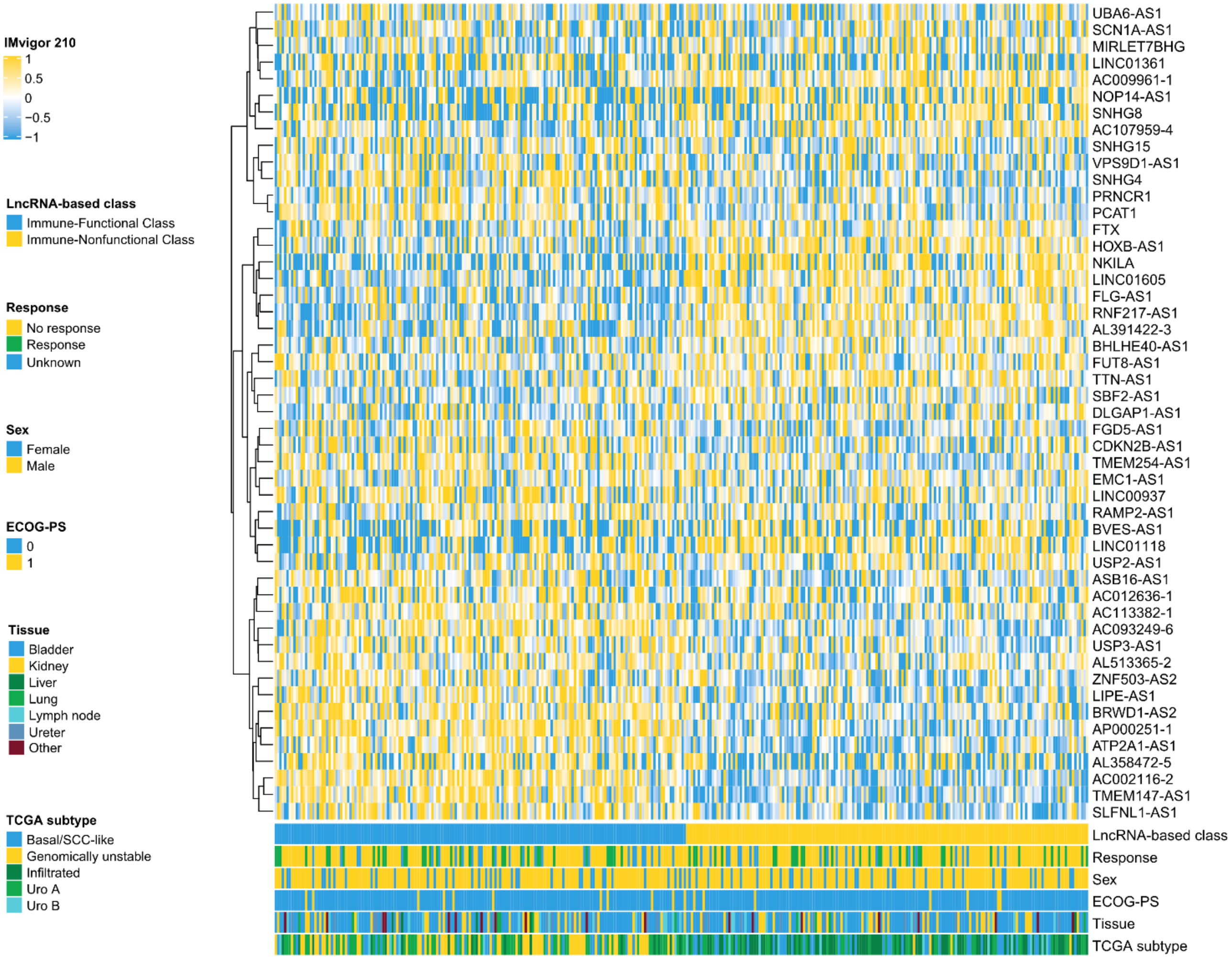

Each row of the heatmap represented a lncRNA. Each column represented a patient. The color indicates the level of expression; the color near to yellow indicates high expression, whereas the color near blue indicates low expression. LncRNA, long non-coding RNA; ECOG-PS, Eastern Cooperative Oncology Group Performance Status; TCGA, The Cancer Genome Atlas; SCC, squamous cell carcinoma.

© 2020 Yu Y et al. *JAMA Network Open.*

eFigure 3. Immune Molecular Expression in Immune-Functional Class Versus Immune-Nonfunctional Class in IMvigor210 Trial.

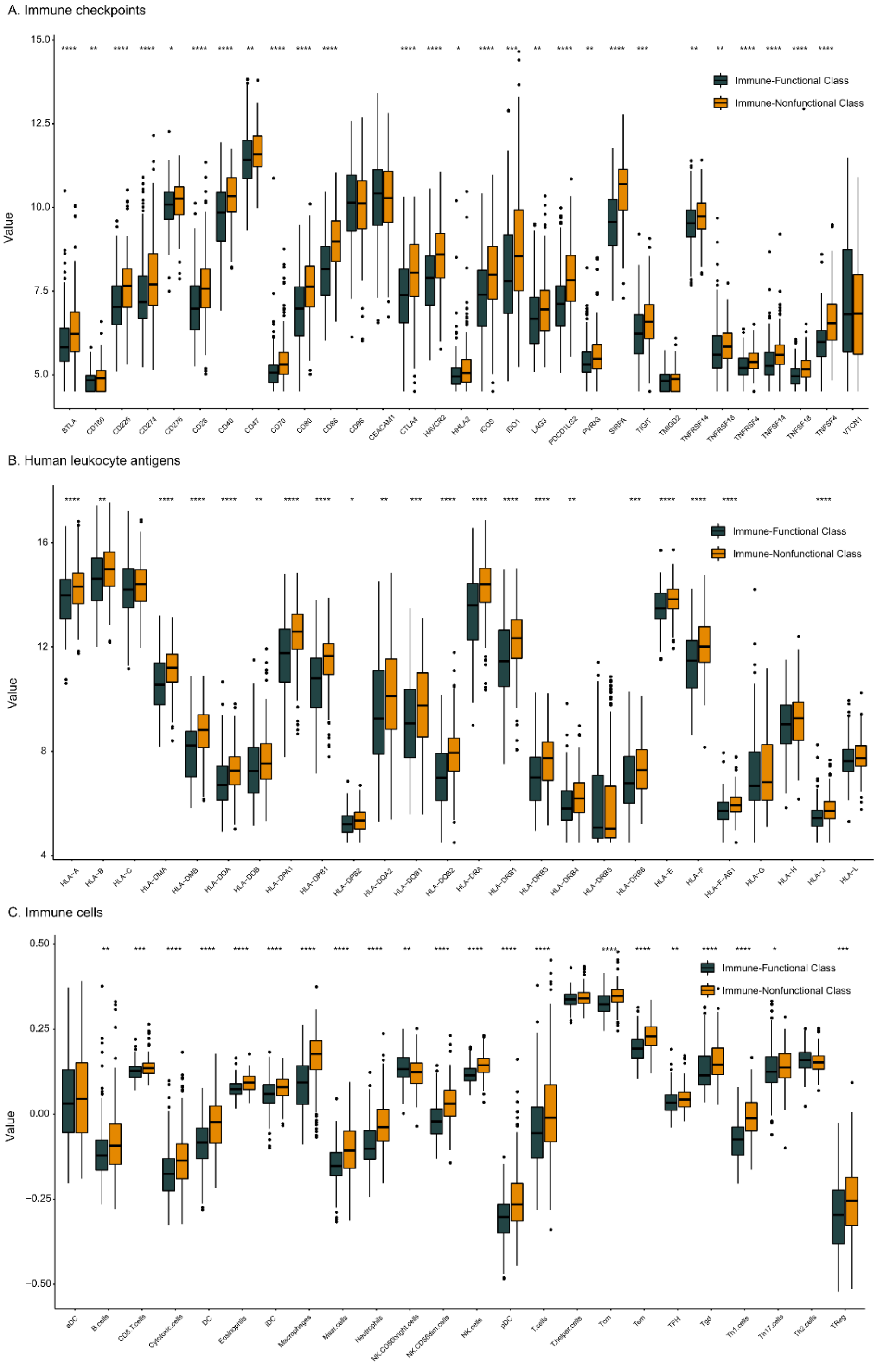

A, Immune checkpoints. B, Human leukocyte antigens. C, Immune cells. HLA, human leukocyte antigen; DC, dendritic cell; NK, natural killer; Th, helper T; Tcm, central memory T cells; Tem, effector memory T cells, pDC, plasmacytoid dendritic cell, TFH, T follicular helper cells; TReg,

© 2020 Yu Y et al. *JAMA Network Open.*

regulatory cells.

© 2020 Yu Y et al. *JAMA Network Open*.

eFigure 4. Heatmap representation of Immune Molecules in Immune-Functional and Immune-Nonfunctional Classes in IMvigor210 Trial.

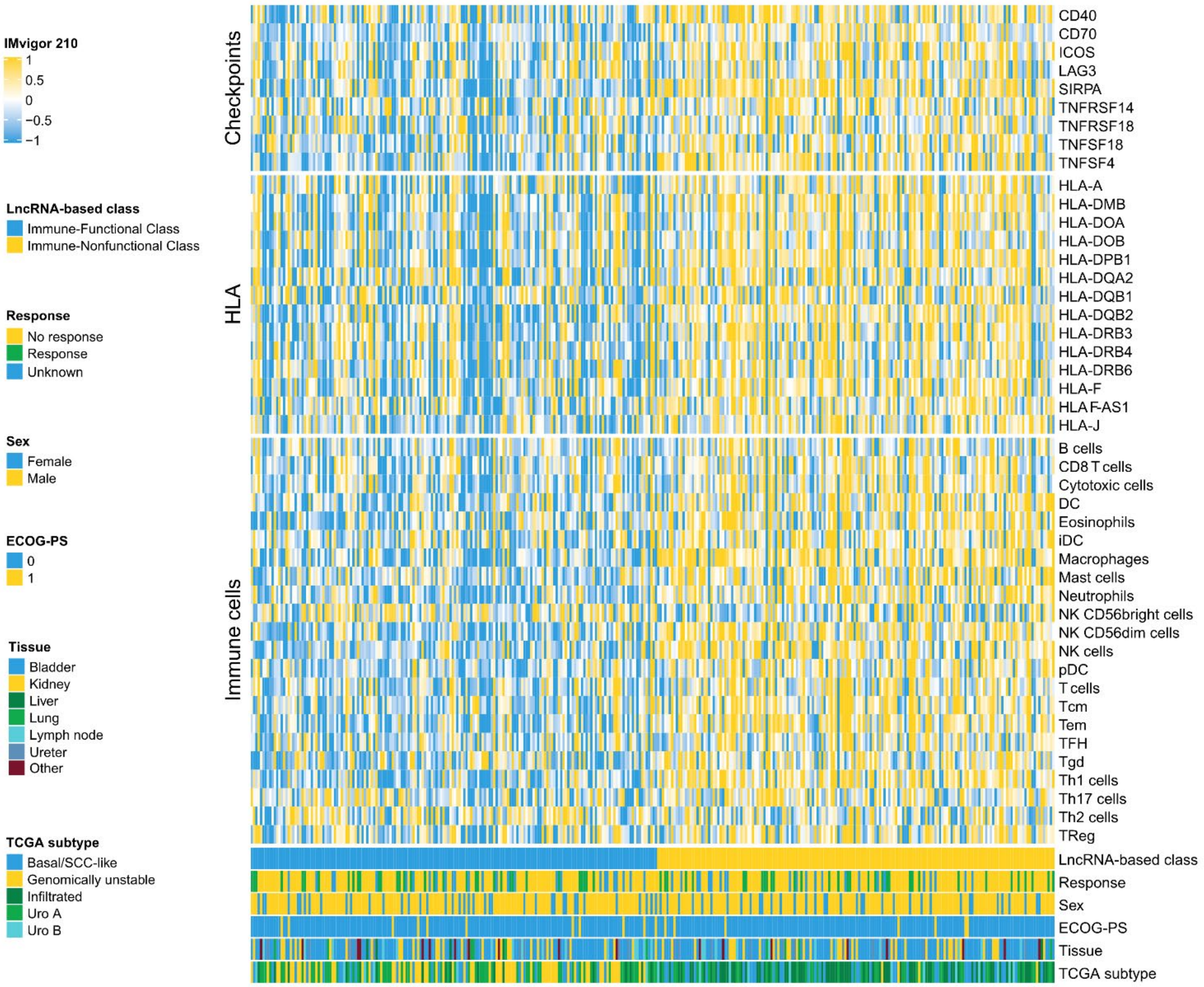

Each row of the heatmap represented an immune molecule or an immune cell. Each column represented a patient. The color indicates the level of expression; the color near to yellow indicates high expression, whereas the color near blue indicates low expression. Immune-Functional Class generally characterized by higher expressions of immune cells, immune checkpoints and human leukocyte antigens, compared to Immune-Nonfunctional Class. LncRNA, long non-coding RNA; ECOG-PS, Eastern Cooperative Oncology Group Performance Status; TCGA, The Cancer Genome Atlas; SCC, squamous cell carcinoma; HLA, human leukocyte antigen; DC, dendritic cell; NK, natural killer; Th, helper T; Tcm, central memory T cells; Tem, effector memory T cells, pDC, plasmacytoid dendritic cell, TFH, T follicular helper cells; TReg, regulatory cells.

© 2020 Yu Y et al. *JAMA Network Open.*

eFigure 5. Gene set variation analysis of Immune-Functional and Immune-Nonfunctional Classes in IMvigor210 Trial.

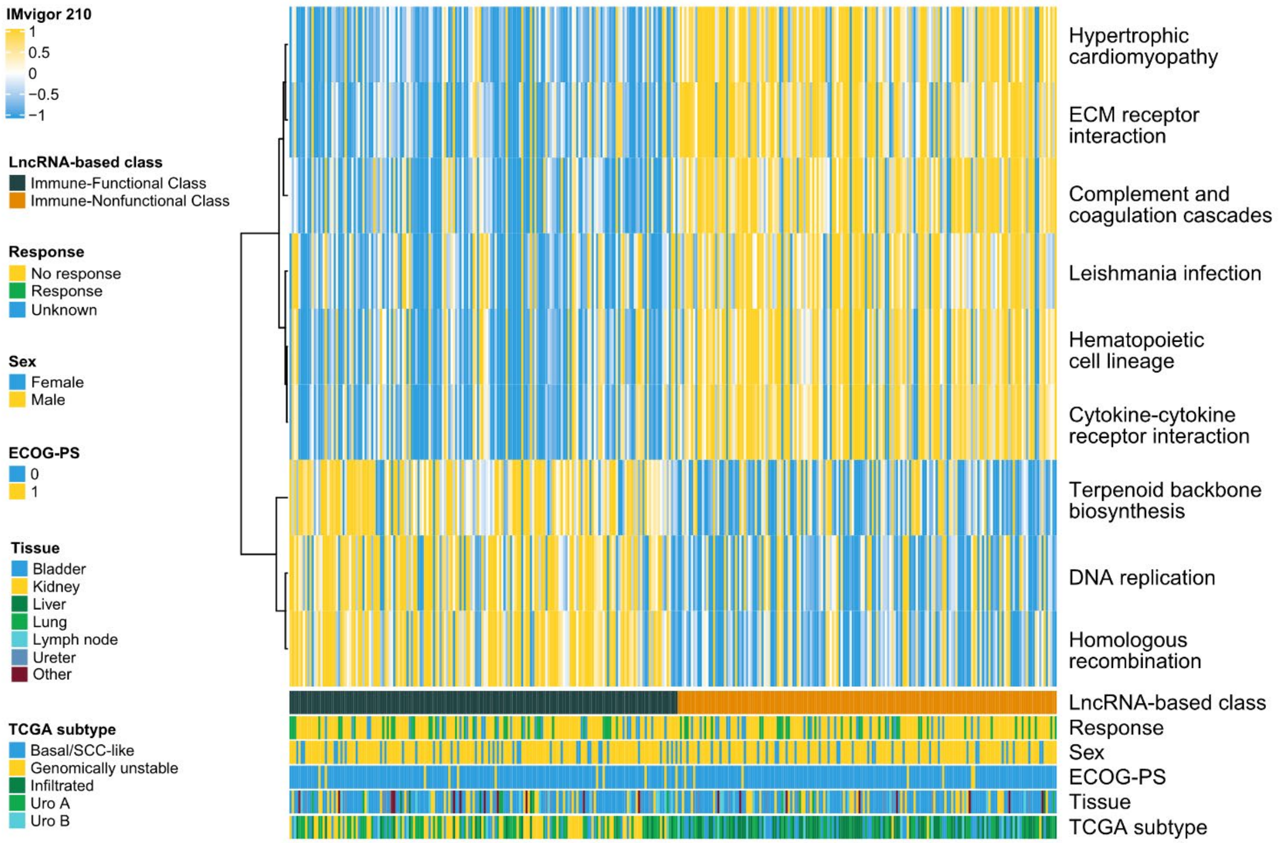

Each row of the heatmap represented a signaling pathway. Each column represented a patient. The color indicates the level of expression; the color near to yellow indicates high expression, whereas the color near blue indicates low expression. LncRNA, long non-coding RNA; ECOG-PS, Eastern Cooperative Oncology Group Performance Status; TCGA, The Cancer Genome Atlas; ECM, extracellular matrix; SCC, squamous cell carcinoma.

© 2020 Yu Y et al. *JAMA Network Open*.

eFigure 6. Identification of Essential lncRNAs in the Clustering in IMvigor210 Trial.

A. Application of LASSO algorithm

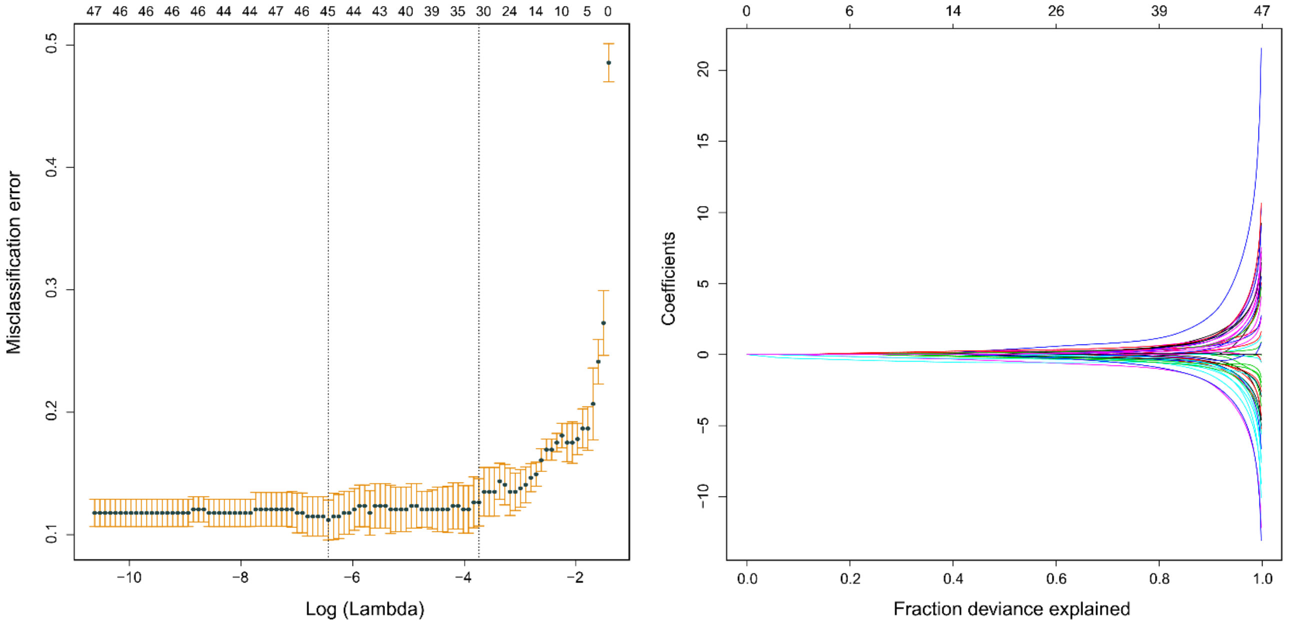

B. Application of random forest method

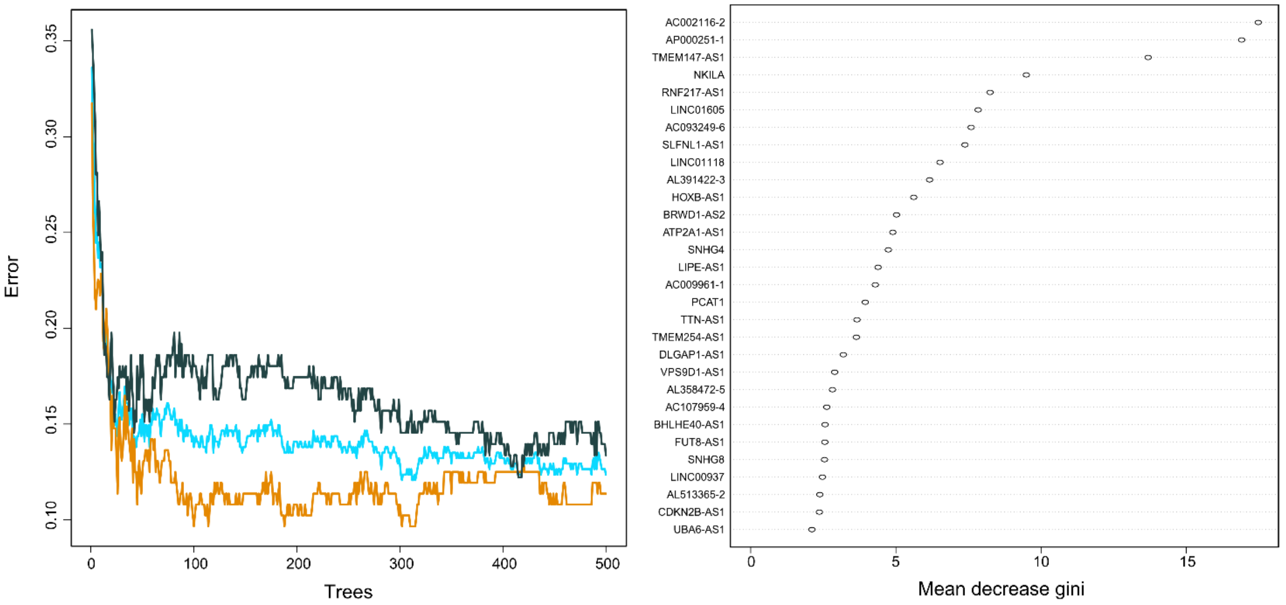

A, Application of LASSO algorithm. B, Application of random forest method. We processed 49 lncRNAs with LASSO algorithm and narrowed down the selection to 32 lncRNAs. Then, we used random forest method to rank the importance of these 32 lncRNAs. Higher scores of mean decrease gini indicated greater importance.

© 2020 Yu Y et al. *JAMA Network Open*.

eFigure 7. Gene Expression Analysis Between Immune-Functional and Immune-Nonfunctional Classes in IMvigor210 Trial.

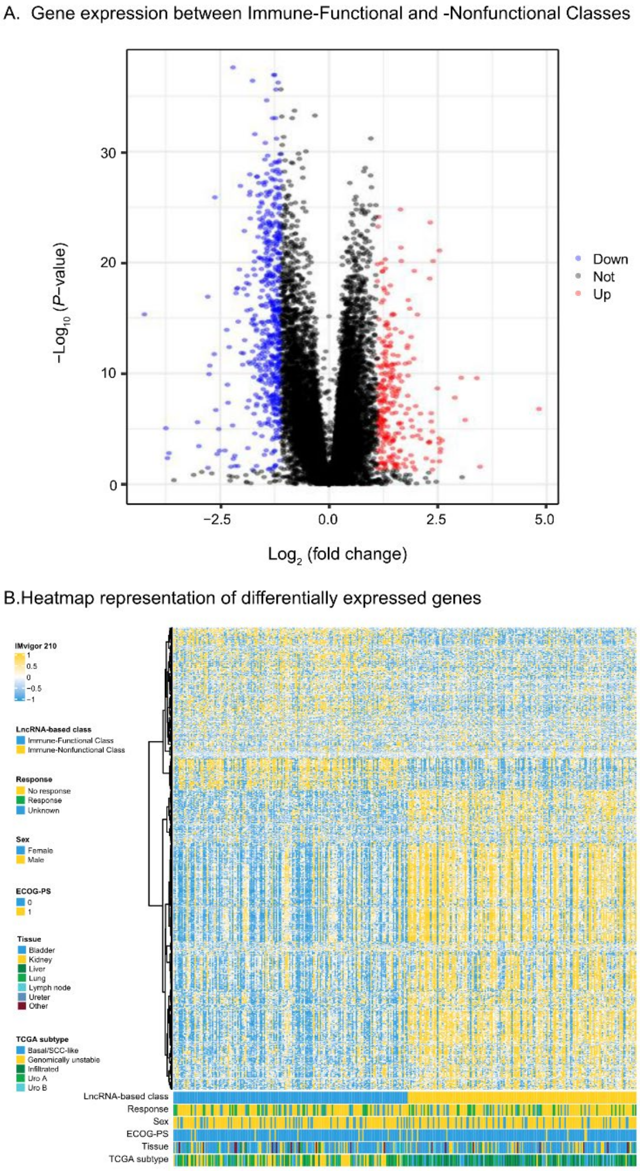

A, Volcano plot showing the gene information between the two classes. B, Heatmap representation of differentially expressed genes between the two classes. Volcano plot plotted fold-change versus significance on the x and y axes, respectively. Each dot on the plot represented one gene. Genes with absolute $\log_2$(foldchange) values larger than the estimated cutoff value 1.11 and *P* values less than .05 were defined as differentially expressed genes between Immune-Functional Class and Immune-Nonfunctional Class. We identified 778 differentially expressed genes between the two classes, of which 259 genes were up-regulated in Immune-Functional Class (Red dots) and 519 genes were up-regulated in Immune-Nonfunctional Class (Blue dots). The black dots indicated genes that were not significantly differentially expressed between the two classes. Each row of the heatmap represented a gene. Each column represented a patient. The color indicates the level of expression; the color near to yellow indicates high expression, whereas the color near blue indicates low expression. LncRNA, long non-coding

© 2020 Yu Y et al. *JAMA Network Open.*

RNA; ECOG-PS, Eastern Cooperative Oncology Group Performance Status; TCGA, The Cancer Genome Atlas; SCC, squamous cell carcinoma.

eFigure 8. Gene Set Enrichment Analysis of NF-κB Pathway Between Immune-Functional and Immune-Nonfunctional Classes in IMvigor210 Trial.

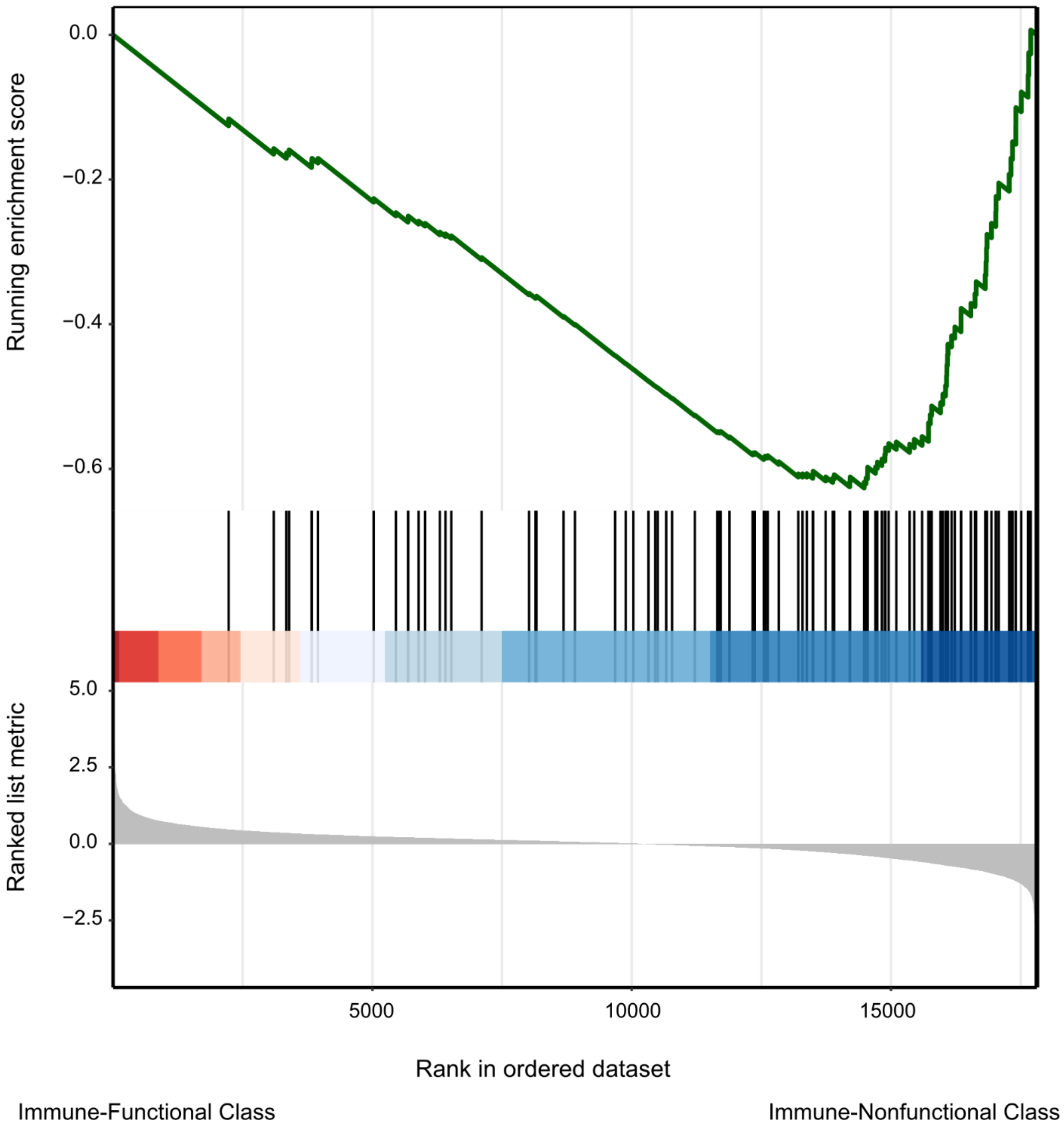

Enrichment score was negative, and vertical black solid lines in the middle of the figure were densely grouped in the right-end, indicating that most genes were low-ranked in NF-κB-related gene set. The results demonstrated that NF-κB-related gene set were highly enriched in Immune Functional Class over Immune-Nonfunctional Class.

© 2020 Yu Y et al. *JAMA Network Open.*

eFigure 9. Matrix Plot Showing the Correlation of NKILA Expression With Signaling Pathways in IMvigor210 Trial.

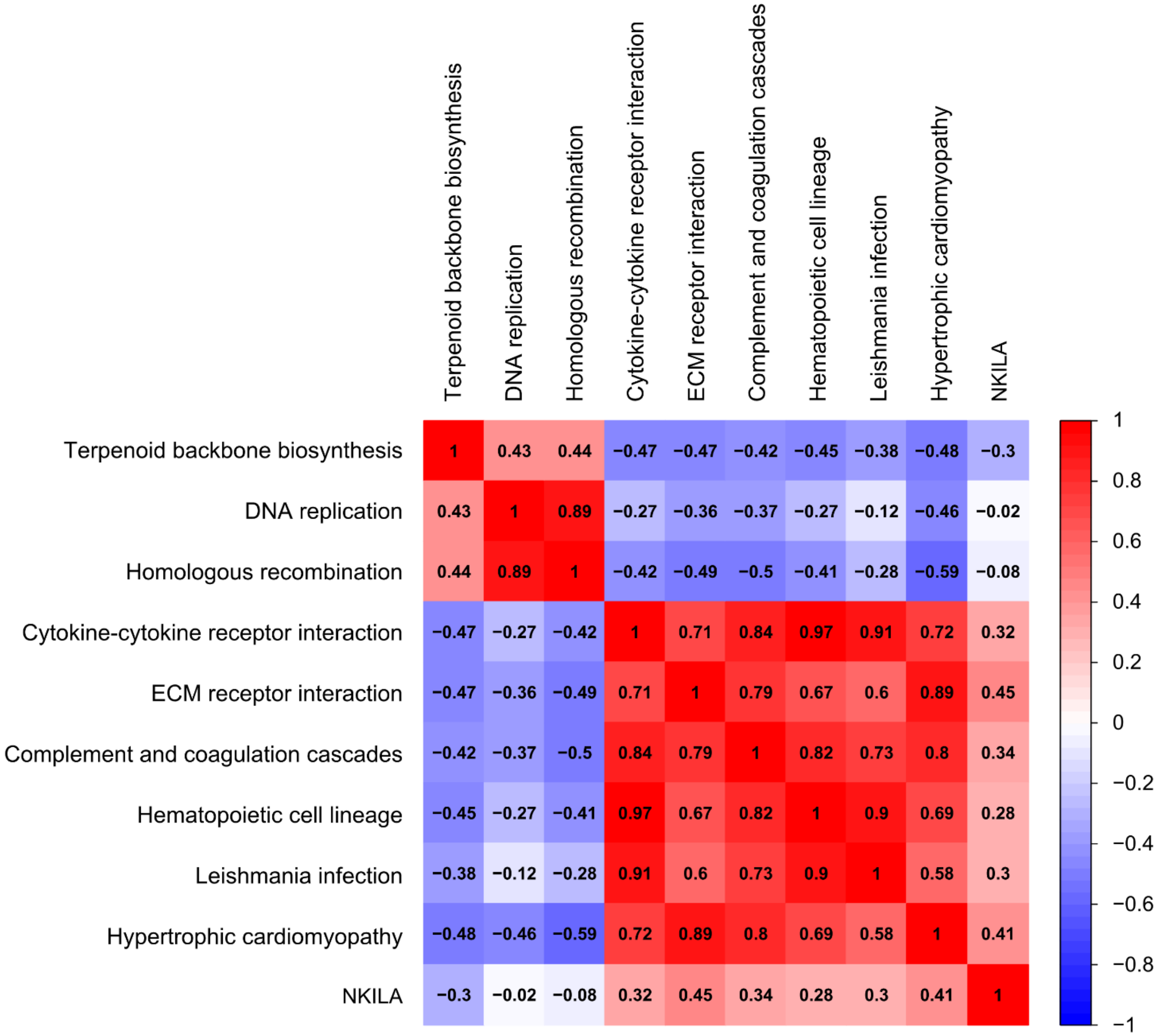

The pathways used here were consistent with those in gene set variation analysis in Immune-Functional and Immune-Nonfunctional Classes. NKILA was found to have consistent enrichment pattern with Immune-Nonfunctional Class. The correlations here were estimated using Pearson ρ. ECM, extracellular matrix.

© 2020 Yu Y et al. *JAMA Network Open*.

eFigure 10. KEGG Pathway Enrichment Analysis of NKILA in IMvigor210 Trial.

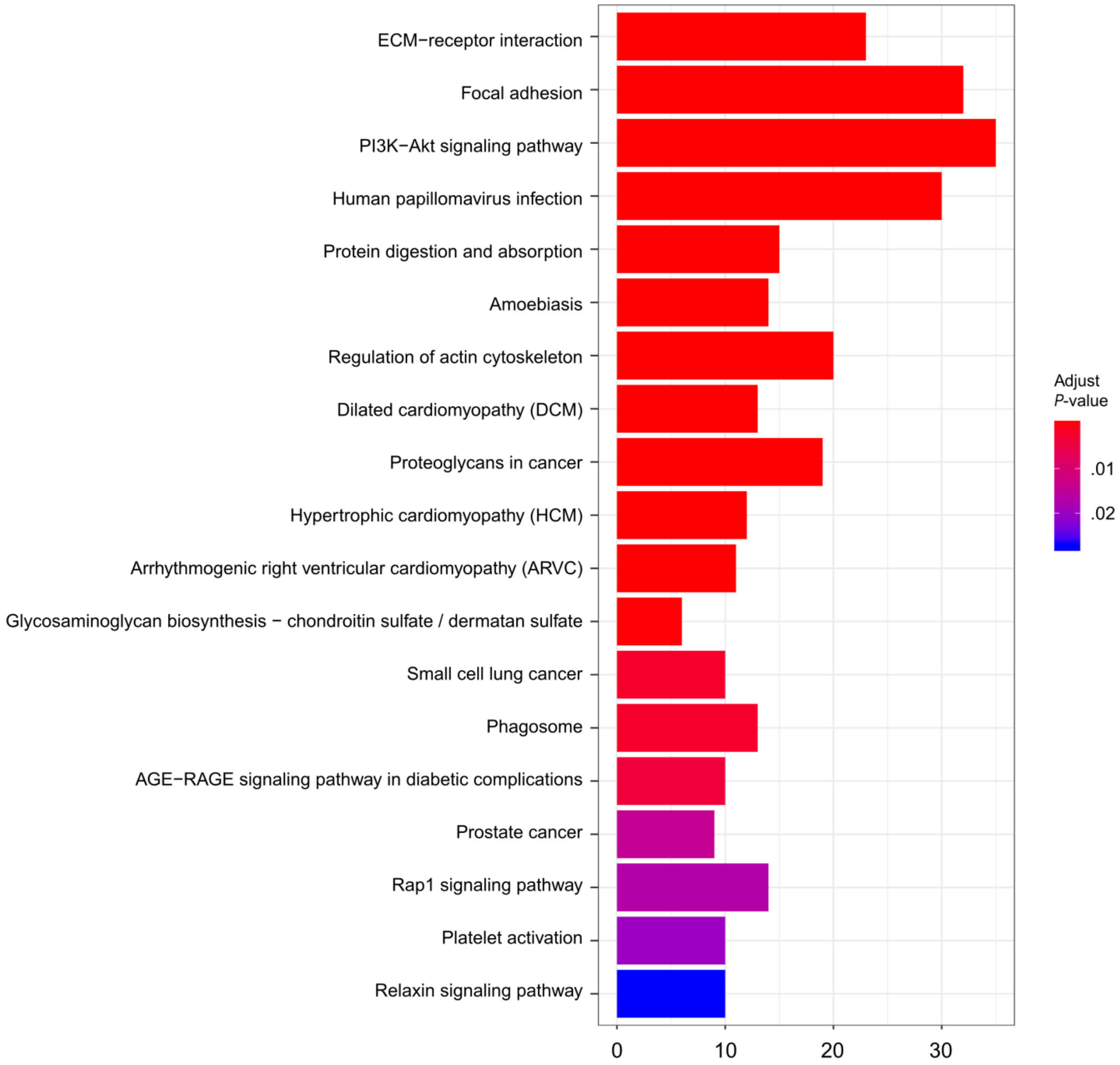

Kyoto Encyclopedia of Genes and Genomes Pathway enrichment analyses showed that NKILA displayed high enrichment in biological processes such as ECM-receptor interaction. ECM, extracellular matrix; KEGG, Kyoto Encyclopedia of Genes and Genomes.

© 2020 Yu Y et al. *JAMA Network Open*.

eFigure 11. Gene Ontology Enrichment Analysis of NKILA in IMvigor210 Trial.

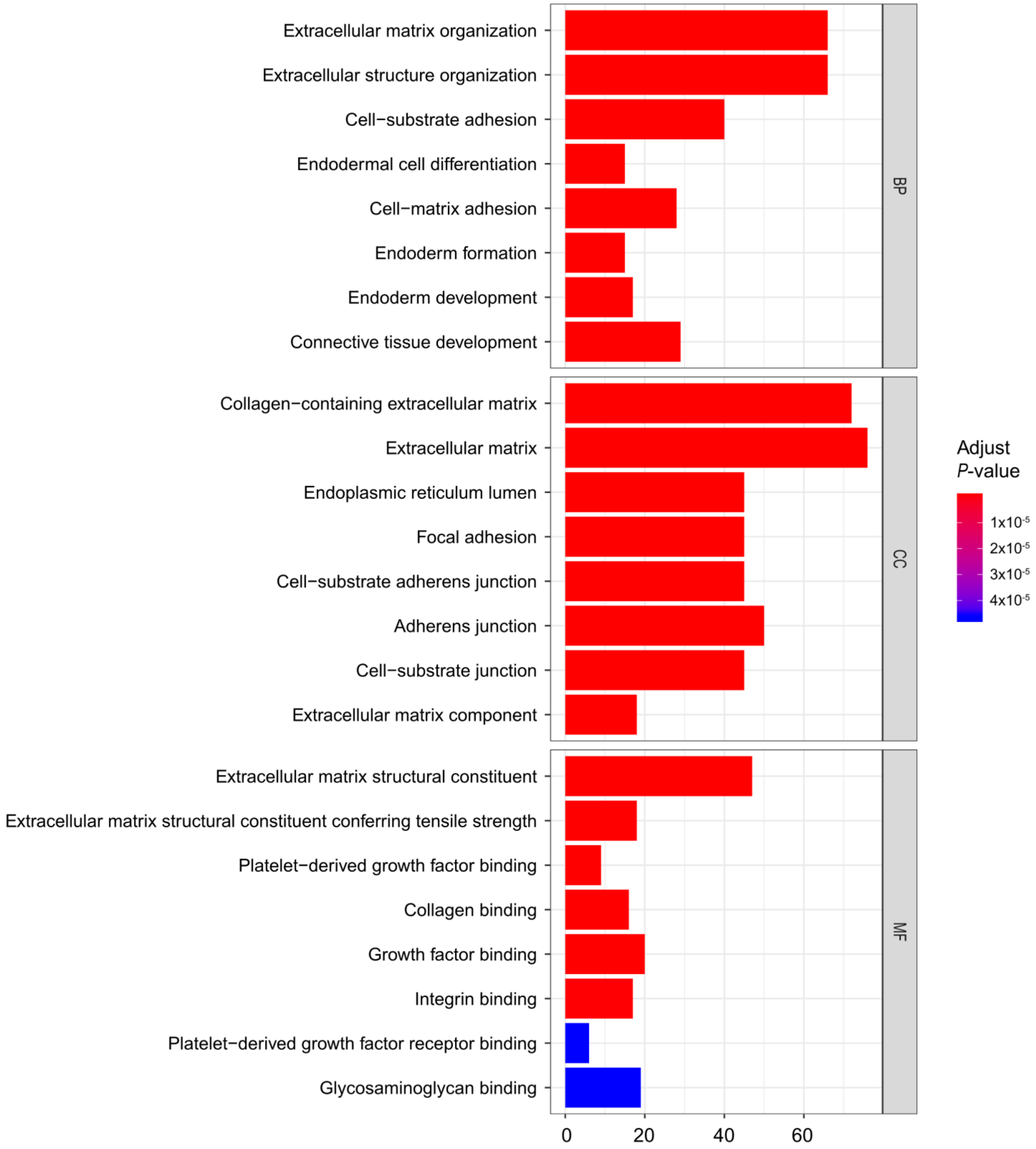

Gene Ontology enrichment analyses showed that NKILA displayed high enrichment in extracellular matrix (ECM)-related biological processes, cellular components, and molecular functions, including ECM-receptor interaction, ECM organization, ECM component, ECM structural constituent, and ECM structural constituent conferring tensile strength.

© 2020 Yu Y et al. *JAMA Network Open*.

eFigure 12. NKILA Expression in Immune-Functional Class Versus Immune-Nonfunctional Class in IMvigor210 Trial.

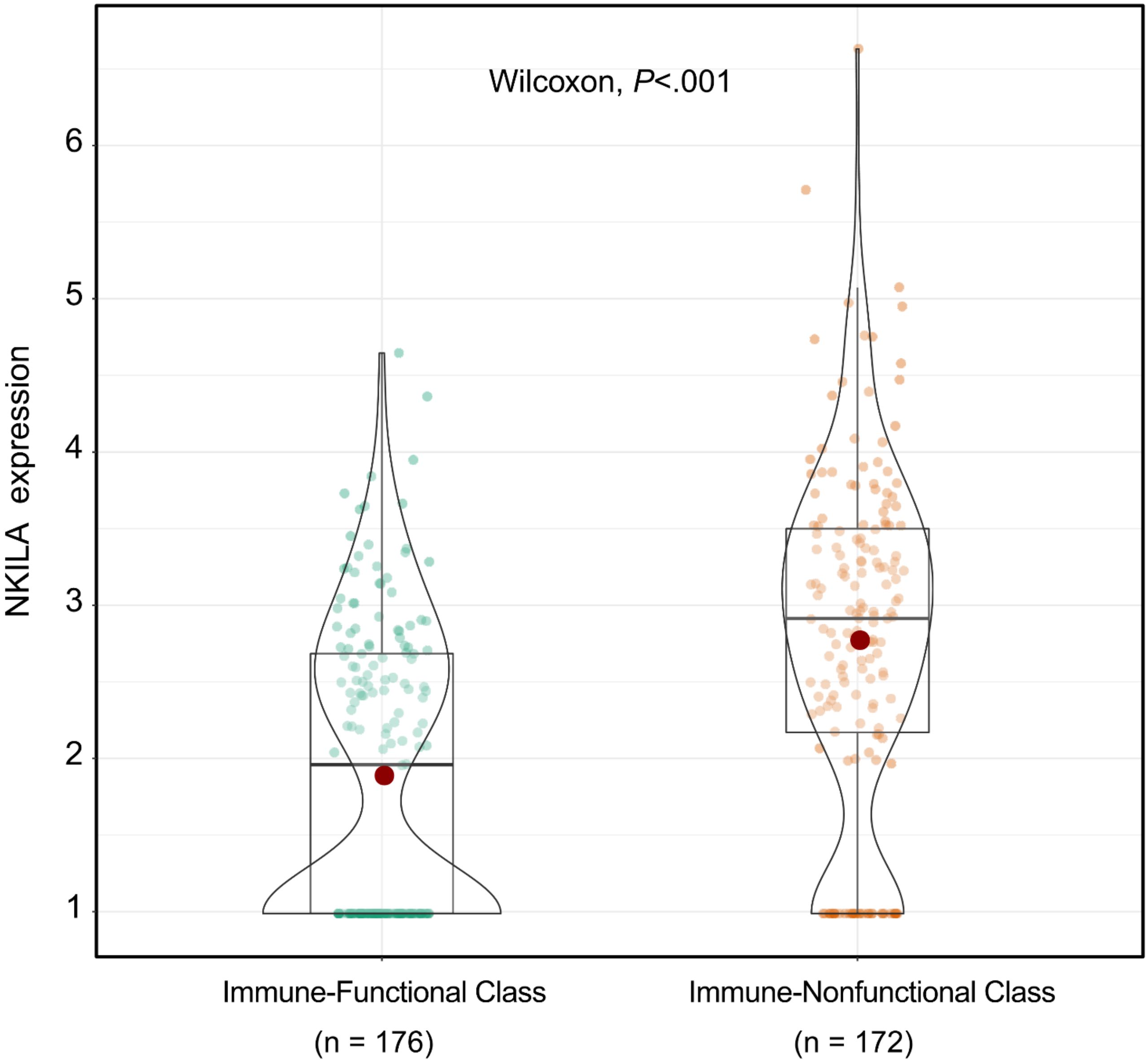

NKILA was significantly lower in Immune-Functional Class than in Immune-Nonfunctional Class.

© 2020 Yu Y et al. *JAMA Network Open.*

eFigure 13. Association of LncRNA-Based Class with Overall Survival in Pancancer.

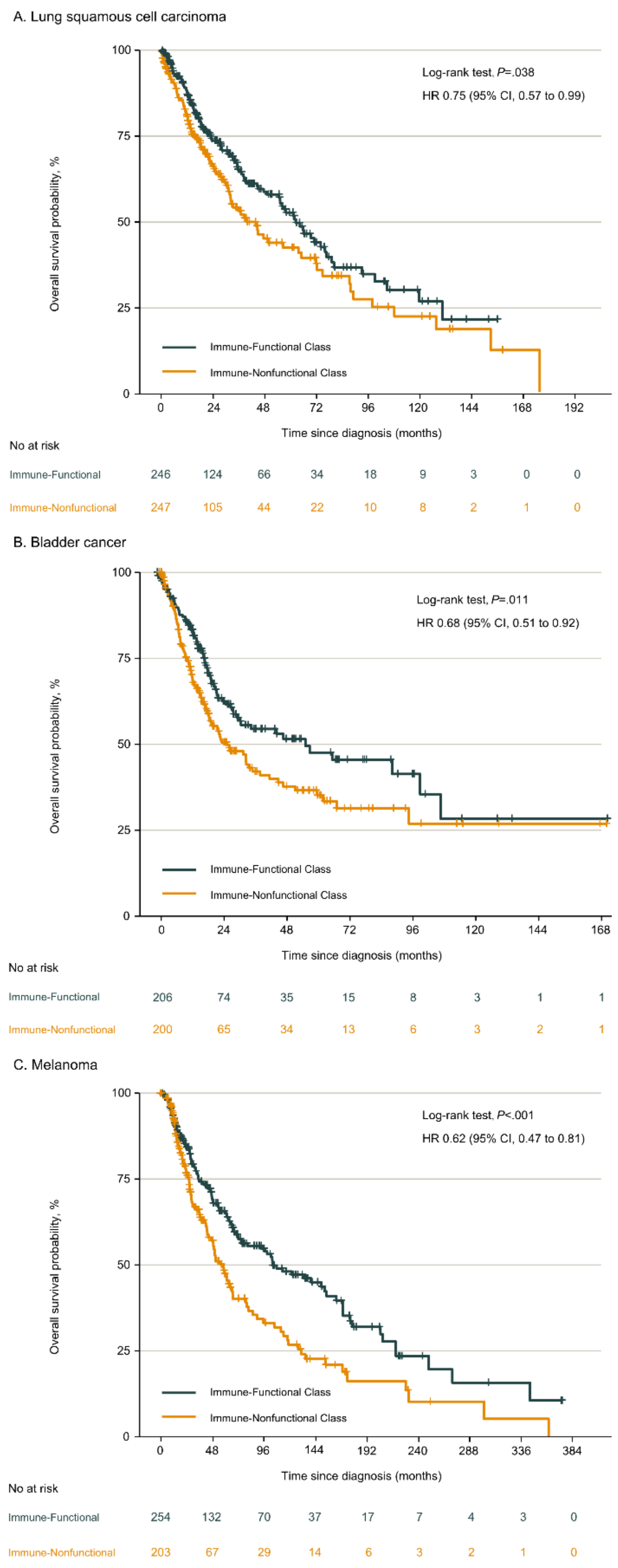

© 2020 Yu Y et al. *JAMA Network Open*.

A, Lung squamous cell carcinoma. B, Bladder cancer. C, Melanoma. HR, hazard ratio; CI, confidence interval.

© 2020 Yu Y et al. *JAMA Network Open*.

eFigure 14. Association of NKILA Expression with Overall Survival in Pancancer.

A. Lung adenocarcinoma

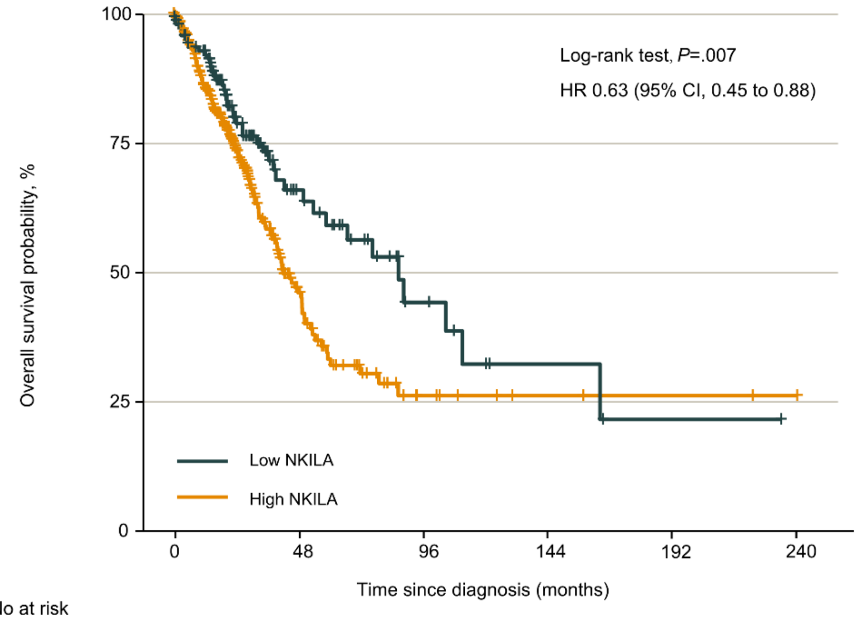

No at risk

Low NKILA	142	30	9	3	1	0
High NKILA	371	50	8	3	2	1

B. Lung squamous cell carcinoma

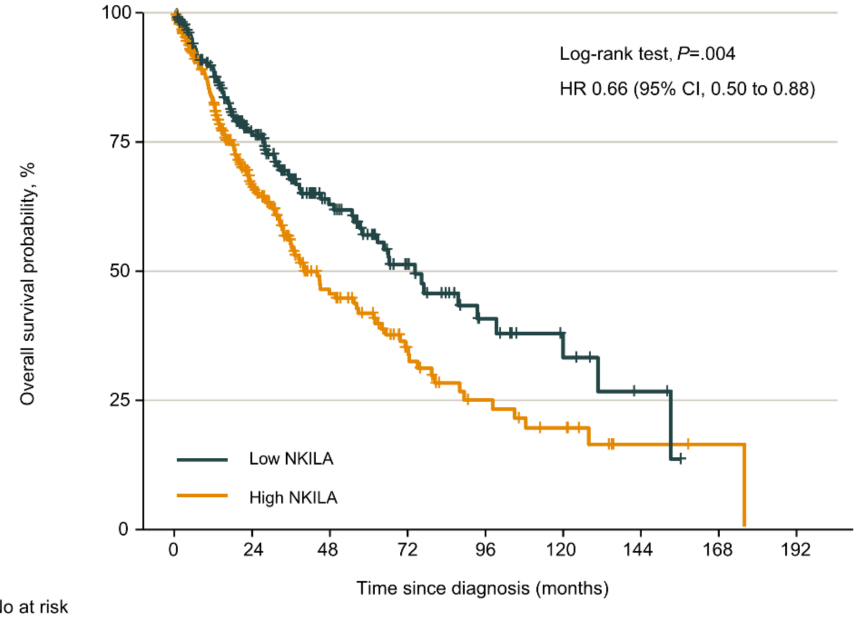

No at risk

Low NKILA	205	113	58	30	14	8	3	0	0
High NKILA	288	116	52	26	14	9	2	1	0

© 2020 Yu Y et al. *JAMA Network Open.*

A, Lung adenocarcinoma. B, Lung squamous cell carcinoma. HR, hazard ratio; CI, confidence interval.

© 2020 Yu Y et al. *JAMA Network Open*.

eFigure 15. Association of Cytotoxic T Lymphocytes with Overall Survival.

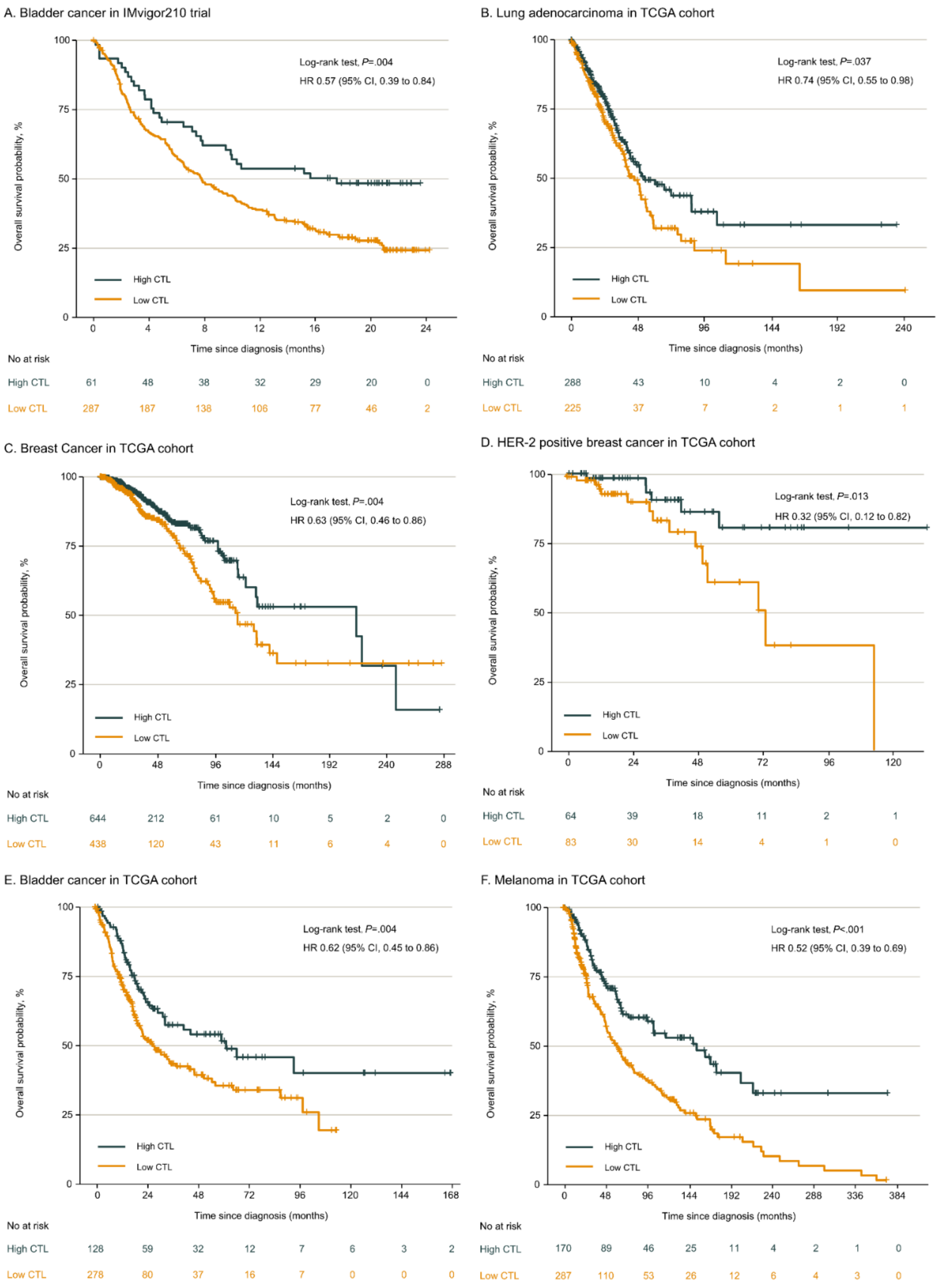

A, Bladder cancer in IMvior210 trial. B, lung adenocarcinoma in TCGA cohort. C, Breast Cancer in TCGA cohort. D, HER-2 positive breast cancer in TCGA cohort. E, Bladder cancer in TCGA cohort. F, Melanoma in TCGA cohort. HR, hazard ratio; CI, confidence interval; CTL,

© 2020 Yu Y et al. *JAMA Network Open*.

cytotoxic T lymphocyte; TCGA, The Cancer Genome Atlas.

© 2020 Yu Y et al. *JAMA Network Open*.

eFigure 16. Correlations Between Cytotoxic T Lymphocytes and Various Immune Molecules in IMvigor210 Trial.

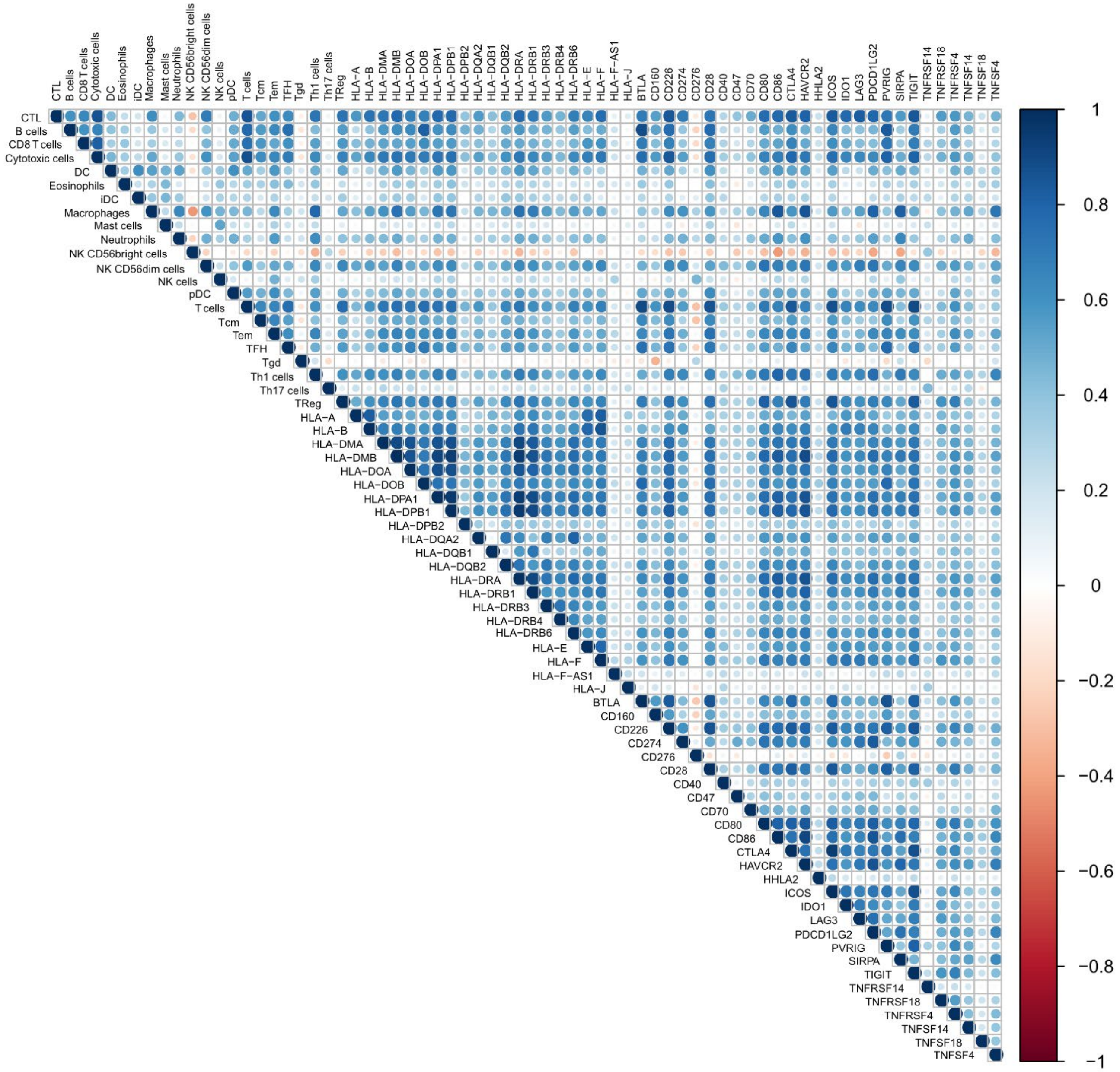

CTL was closely correlated with multiple immune molecules including immune cells such as B cells, CD8 T cells, cytotoxic cells, dendritic cells, macrophages, neutrophils, T cells, natural killer cells, and type 1 T helper cells, central memory T cells, effector memory T cells, plasmacytoid dendritic cell, T follicular helper cells, and regulatory cells; immune checkpoints such as BTLA, CD160, CD226, CD274, CD28, CD70, CD80, CD86, HAVCR2, ICOS, IDO1, CTLA4, LAG3, PDCD1LG2, PVRIG, SIRPA, and TIGIT; and human leukocyte antigens such as HLA-A, HLA-B, HLA-DMA, HLA-DMB, HLA-DOA, HLA-DOB, HLA-DPA1, HLA-DPB1, HLA-DQA2, DLA-DRA, HLA-DRB1, HLA-DRB3, HLA-DRB4, HLA-DRB6, HLA-E, and HLA-F. HLA, human leukocyte antigen; DC, dendritic cell; NK, natural killer; Th, helper T; Tcm, central memory T cells; Tem, effector memory T cells, pDC, plasmacytoid dendritic cell, TFH, T follicular helper cells; TReg, regulatory cells.

© 2020 Yu Y et al. *JAMA Network Open.*

eFigure 17. Objective Response Rate Stratified by lncRNAs and Infiltration of Cytotoxic T Lymphocytes.

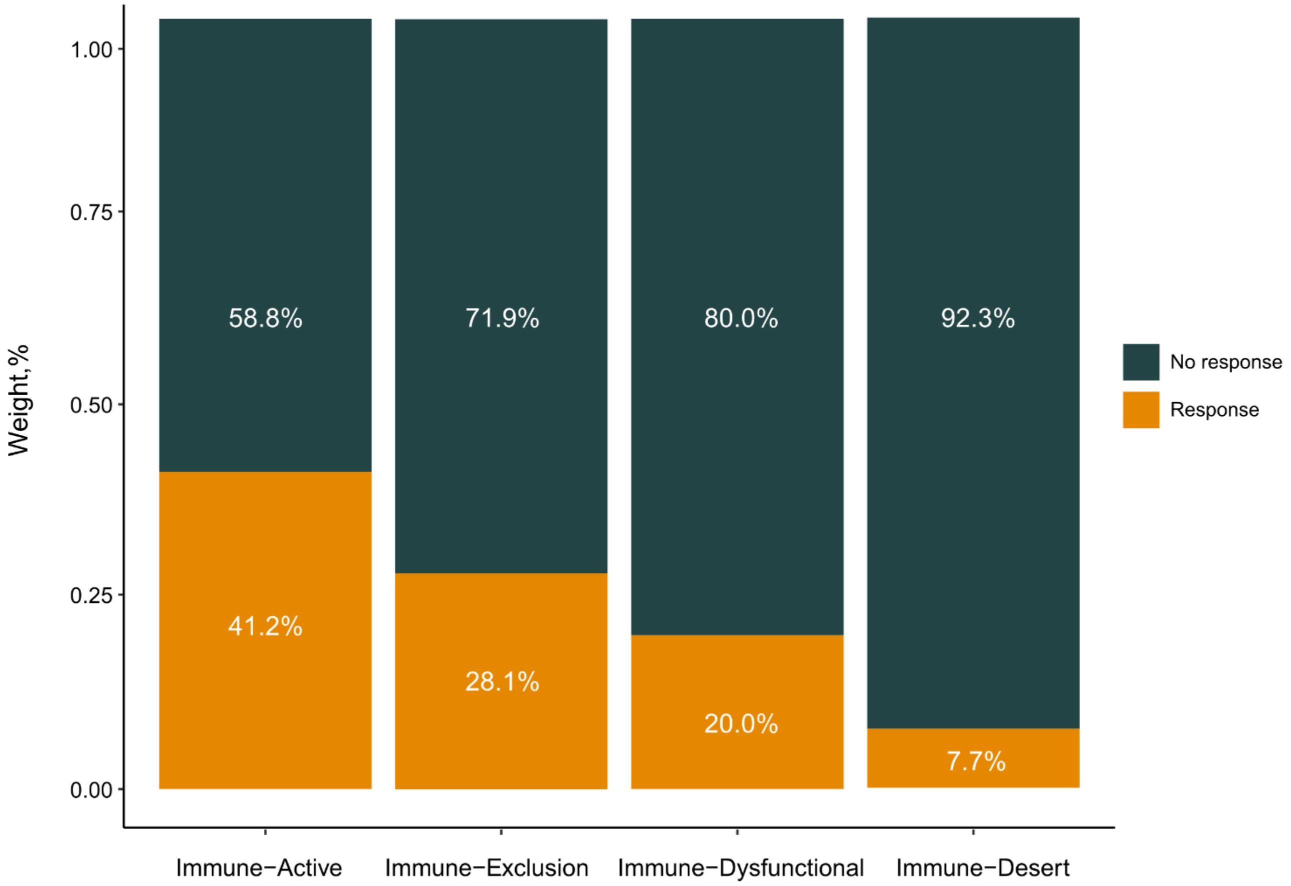

© 2020 Yu Y et al. *JAMA Network Open.*

eFigure 18. Association of LncRNA-CTL-Based Class with Immune Molecular Expression in IMvigor210 Trial.

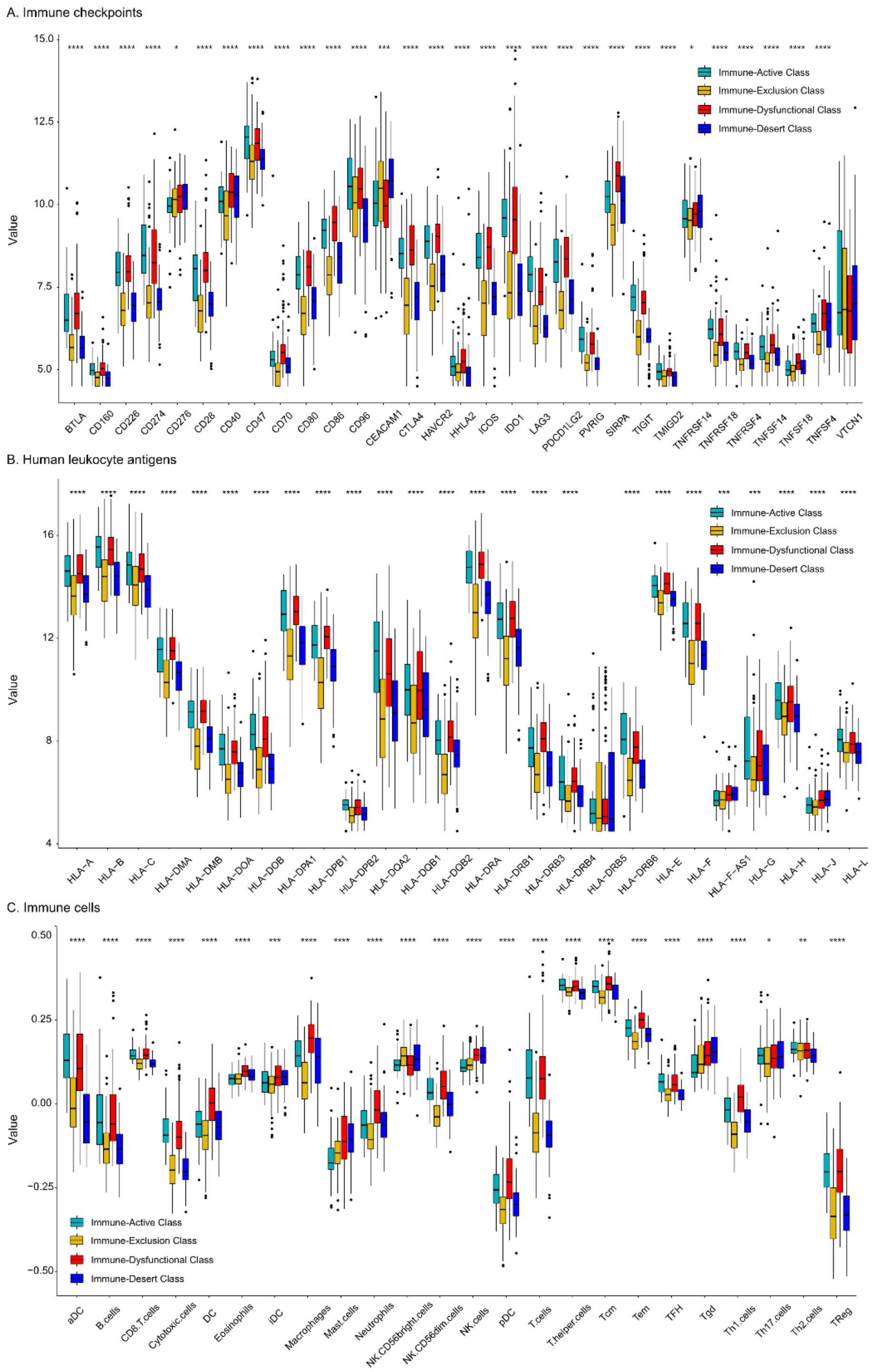

A, Immune checkpoints. B, Human leukocyte antigens. C, Immune cells. HLA, human leukocyte antigen; DC, dendritic cell; NK, natural killer; Th, helper T; Tcm, central memory T cells; Tem, effector memory T cells, pDC, plasmacytoid dendritic cell, TFH, T follicular helper cells; TReg,

© 2020 Yu Y et al. *JAMA Network Open.*

regulatory cells.

© 2020 Yu Y et al. *JAMA Network Open*.

eFigure 19. Immune Molecular Expression Pattern of LncRNA-CTL-Based Classes in TCGA Bladder Cancer Cohort.

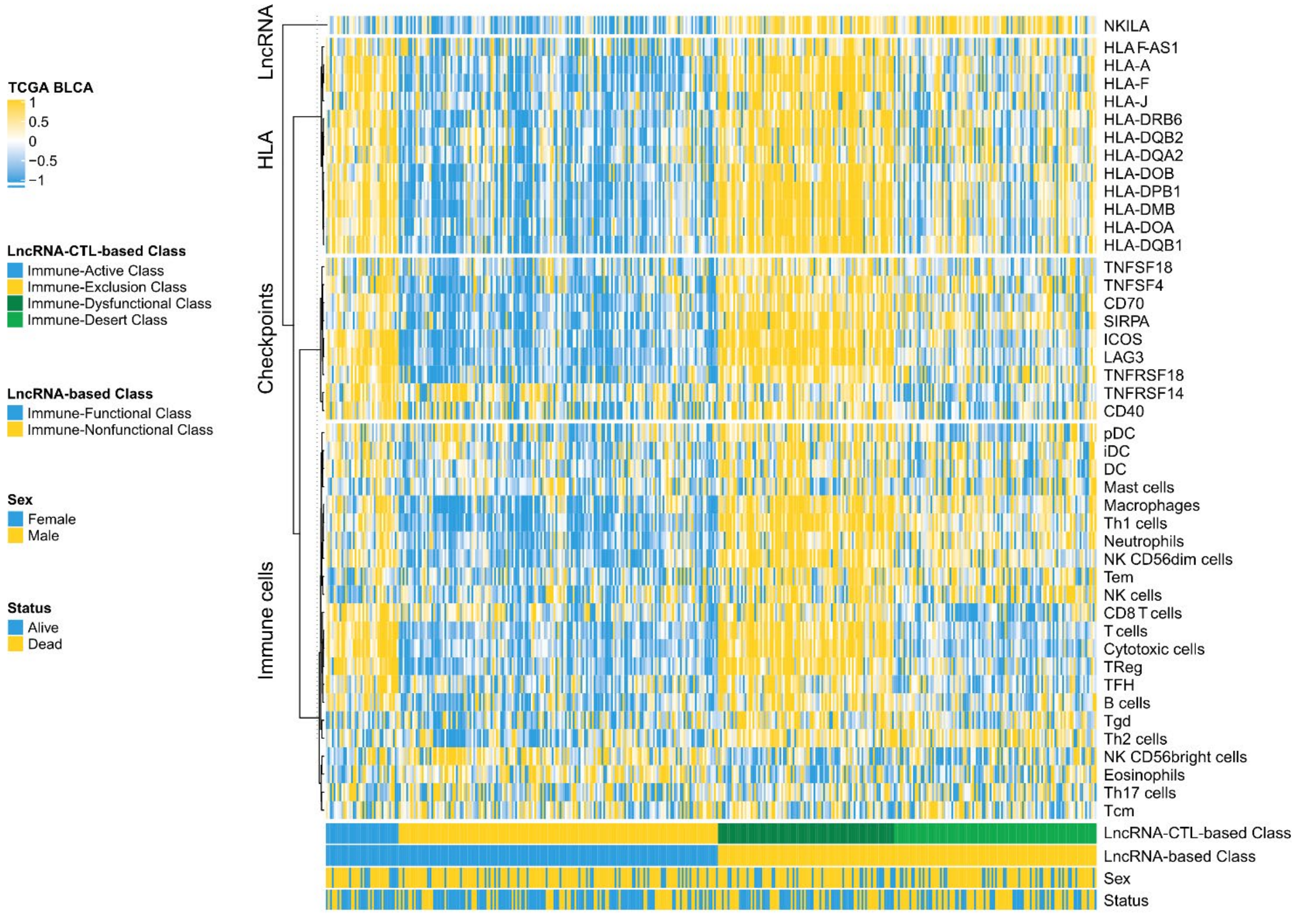

Each row of the heatmap represented an immune molecule or an immune cell. Each column represented a patient. The color indicates the level of expression; the color near to yellow indicates high expression, whereas the color near blue indicates low expression. We combined CTL expression and lncRNA signature to in-deep categorize patients with bladder cancer in TCGA cohort into four subsets. Consistent with the findings in IMvigor210 trial, Immune-Active Class and Immune-Dysfunctional Class generally characterized by higher expressions of immune cells, immune checkpoints and human leukocyte antigens, compared to Immune-Exclusion Class and Immune-Desert Class. LncRNA, long non-coding RNA; ECOG-PS, Eastern Cooperative Oncology Group Performance Status; CTL, cytotoxic T lymphocyte; BLCA, bladder cancer; HLA, human leukocyte antigen; DC, dendritic cell; NK, natural killer; Th, helper T; Tcm, central memory T cells; Tem, effector memory T cells, pDC, plasmacytoid dendritic cell, TFH, T follicular helper cells; TReg, regulatory cells.

© 2020 Yu Y et al. *JAMA Network Open.*

eFigure 20. Weighted Gene Co-Expression Network Analysis of Four LncRNA-CTL-Based Classes in IMvigor210 Trial.

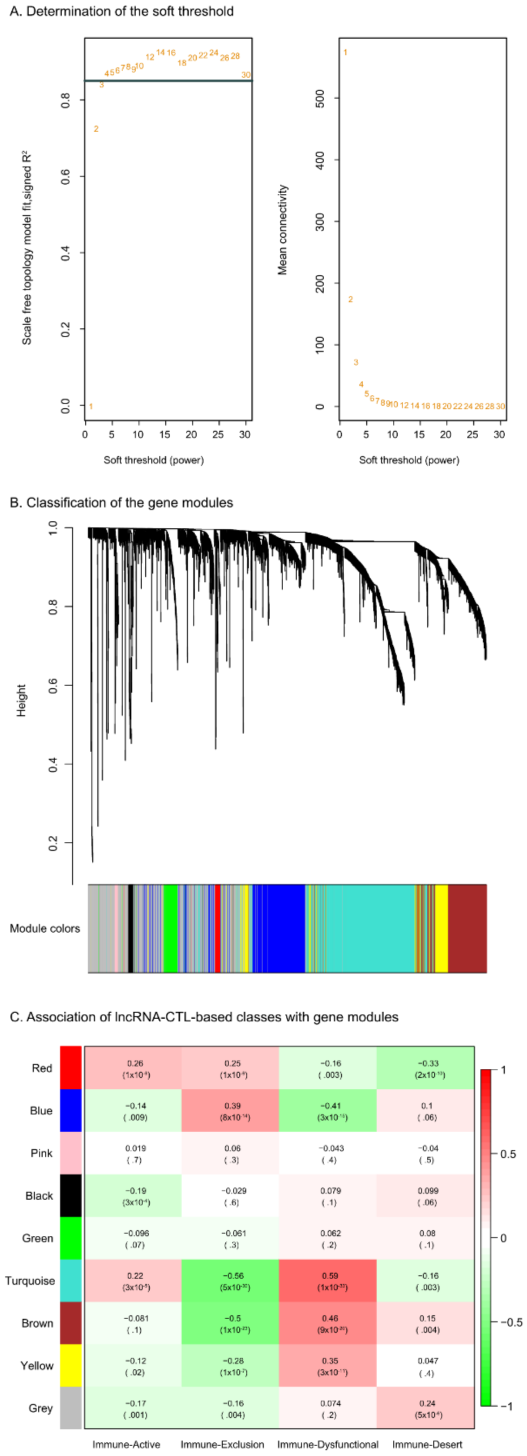

A, Determination of the soft threshold (power value). B, Classification of gene modules. C, Matrix plot showing the degree of association of Immune-Active Class, Immune-Exclusion Class, Immune-Dysfunctional Class, and Immune-Desert Class with gene modules. LncRNA, long

© 2020 Yu Y et al. *JAMA Network Open*.

non-coding RNA; CTL, cytotoxic T lymphocyte.

© 2020 Yu Y et al. *JAMA Network Open*.

eFigure 21. KEGG Pathway Enrichment Analysis of Four LncRNA-CTL-Based Classes in IMvigor210 Trial.

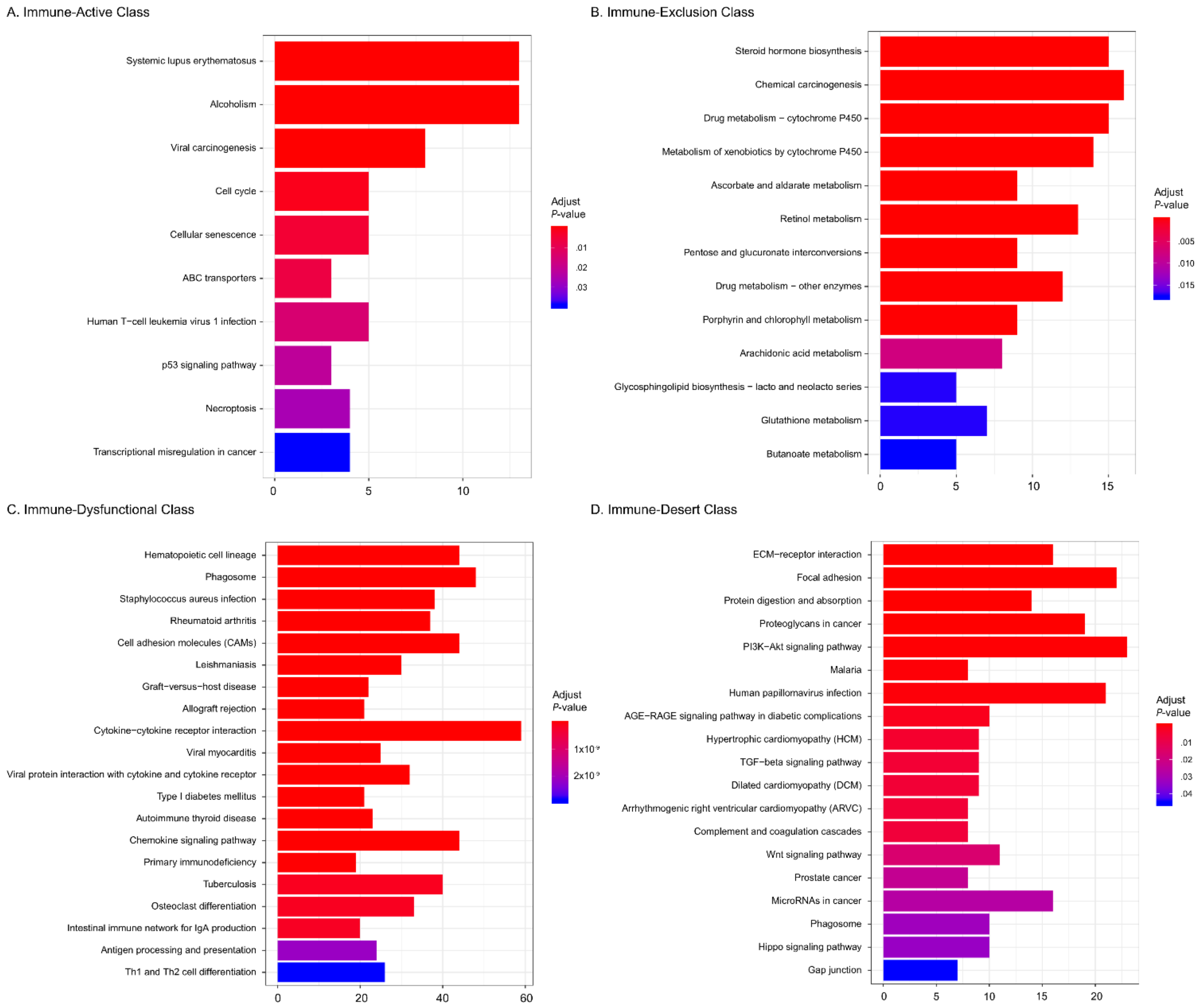

A, Immune-Active Class. B, Immune-Exclusion Class. C, Immune-Dysfunctional Class. D, Immune-Desert Class. Immune-Active Class was significantly involved in cell cycle and p53 signaling pathway; Immune-Exclusion Class was actively involved in various physiological and pathological metabolism; Immune-Dysfunctional Class was involved in phagosome, primary immunodeficiency, and Th1 and Th2 cell differentiation; and Immune-Desert Class showed enrichment in ECM−receptor interaction and PI3K−Akt signaling pathway. KEGG, Kyoto Encyclopedia of Genes and Genomes.

© 2020 Yu Y et al. *JAMA Network Open.*

eFigure 22. Gene Ontology Pathway Enrichment Analysis of Four LncRNA-CTL-Based Classes in IMvigor210 Trial.

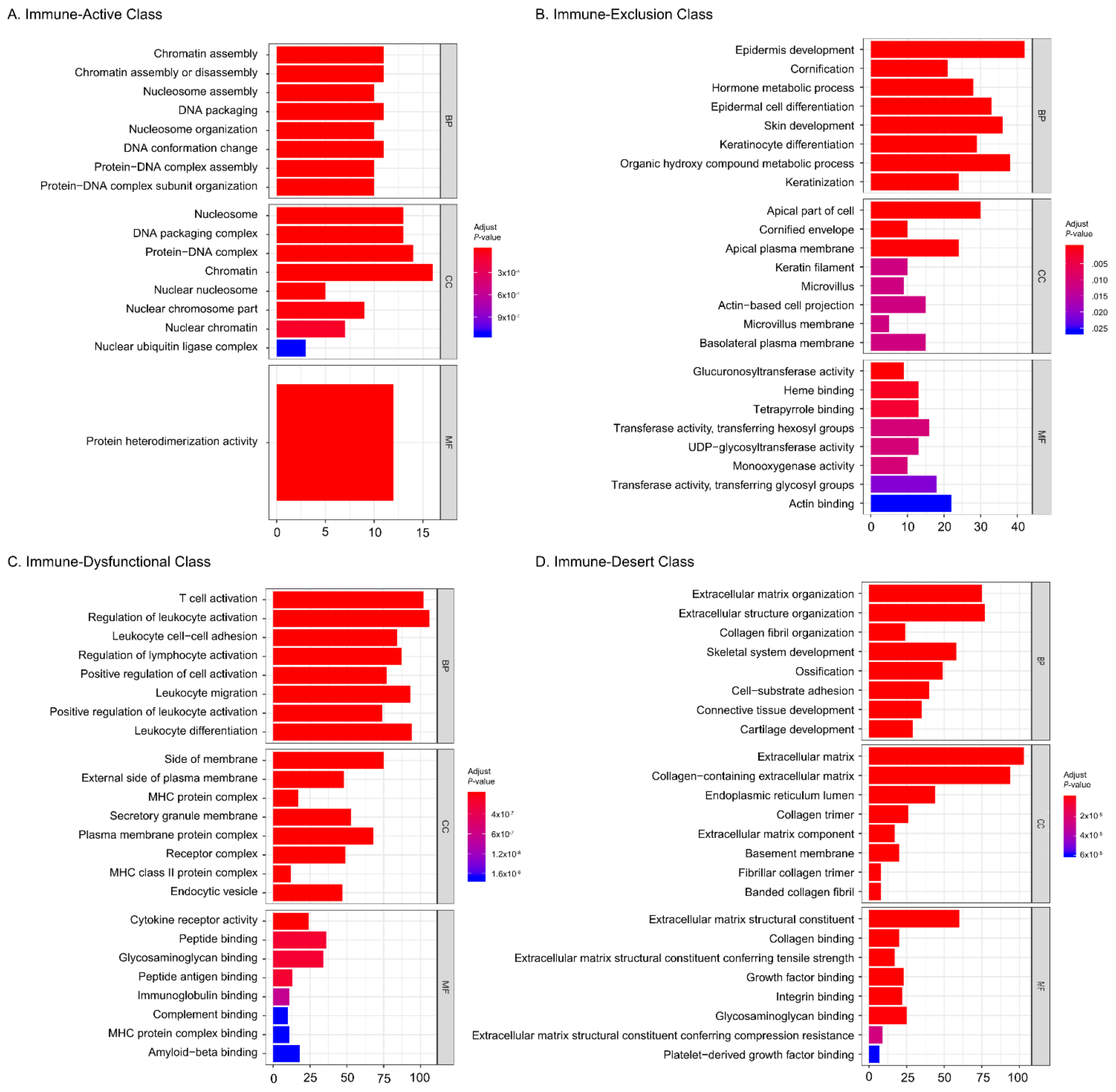

A, Immune-Active Class. B, Immune-Exclusion Class. C, Immune-Dysfunctional Class. D, Immune-Desert Class. Immune-Active Class was significantly enriched of genomic biological processes such as chromatin assembly or disassembly, nucleosome assembly, DNA packaging, nucleosome organization, and DNA conformation change; Immune-Exclusion Class was actively enriched of epithelial change such as epidermis development, cornification, keratinization, and epidermal cell or keratinocyte differentiation; Immune-Dysfunctional Class was enriched of immunologic processes such as activation of T cell and leukocyte; and Immune-Desert Class showed enrichment in ECM-related

© 2020 Yu Y et al. *JAMA Network Open*.

processes such as ECM organization and extracellular structure organization.

© 2020 Yu Y et al. *JAMA Network Open*.

eFigure 23. Identification of the Optimal Cutoff For LncRNA Score.

Distribution

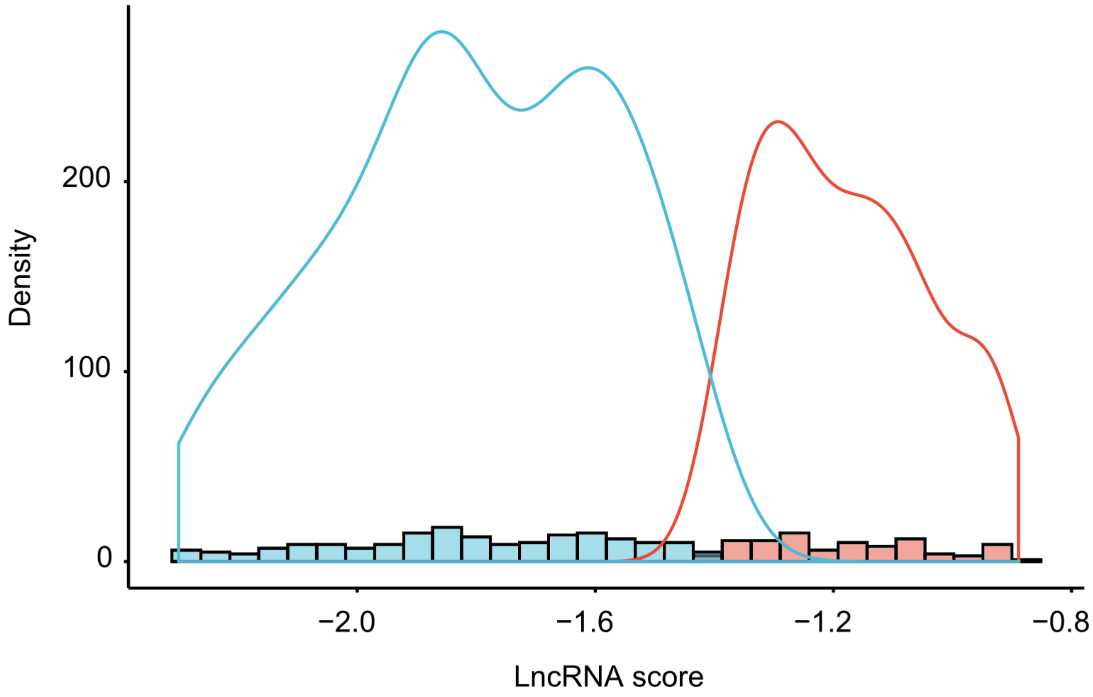

Maximally selected rank statistics

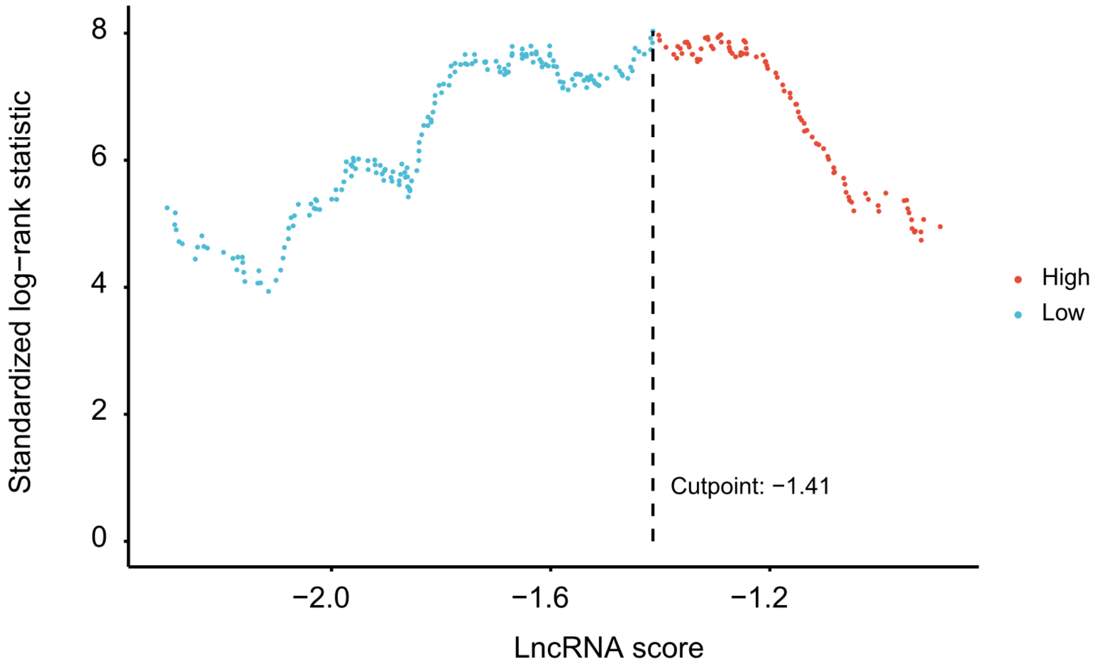

© 2020 Yu Y et al. *JAMA Network Open.*

eFigure 24. Overall Survival Stratified by LncRNA Score in IMvigor210 Trial.

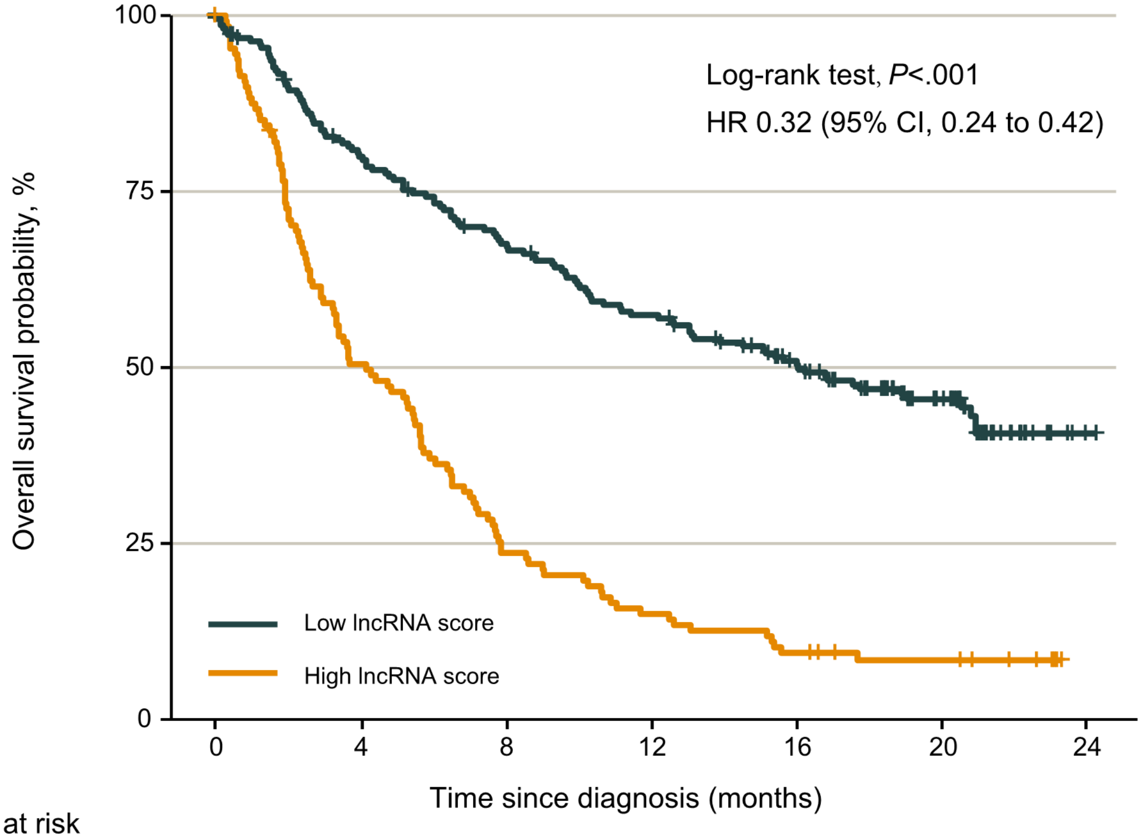

No at risk

Low lncRNA score	219	171	143	119	94	58	2
High lncRNA score	129	64	33	19	12	8	0

LncRNA, long non-coding RNA; HR, hazard ratio; CI, confidence interval.

© 2020 Yu Y et al. *JAMA Network Open.*

eFigure 25. Objective Response Rate Stratified by LncRNA Score in IMvigor210 Trial.

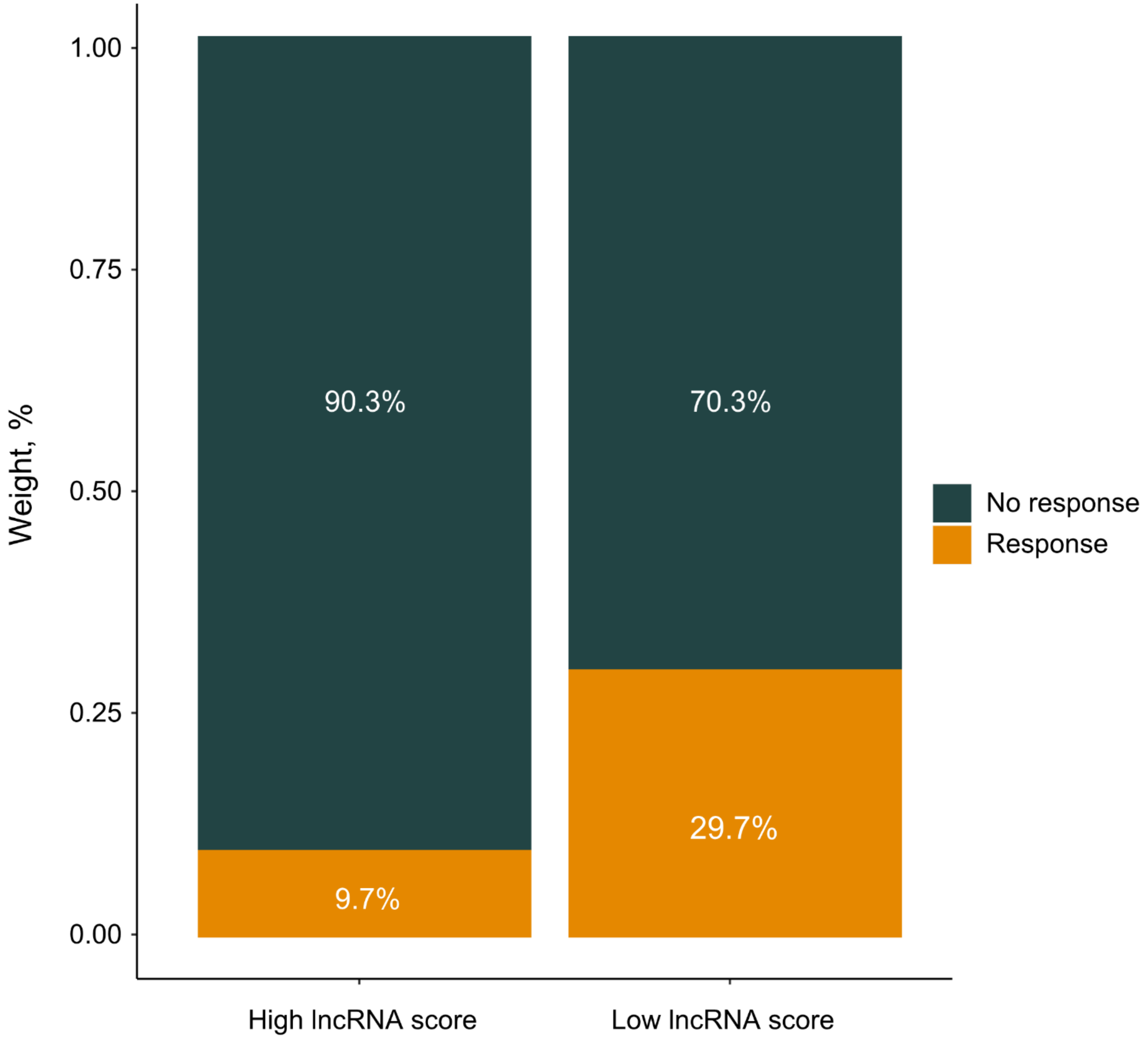

LncRNA, long non-coding RNA.

© 2020 Yu Y et al. *JAMA Network Open.*

eFigure 26. Disease Control Rate Stratified by LncRNA Score in IMvigor210 Trial.

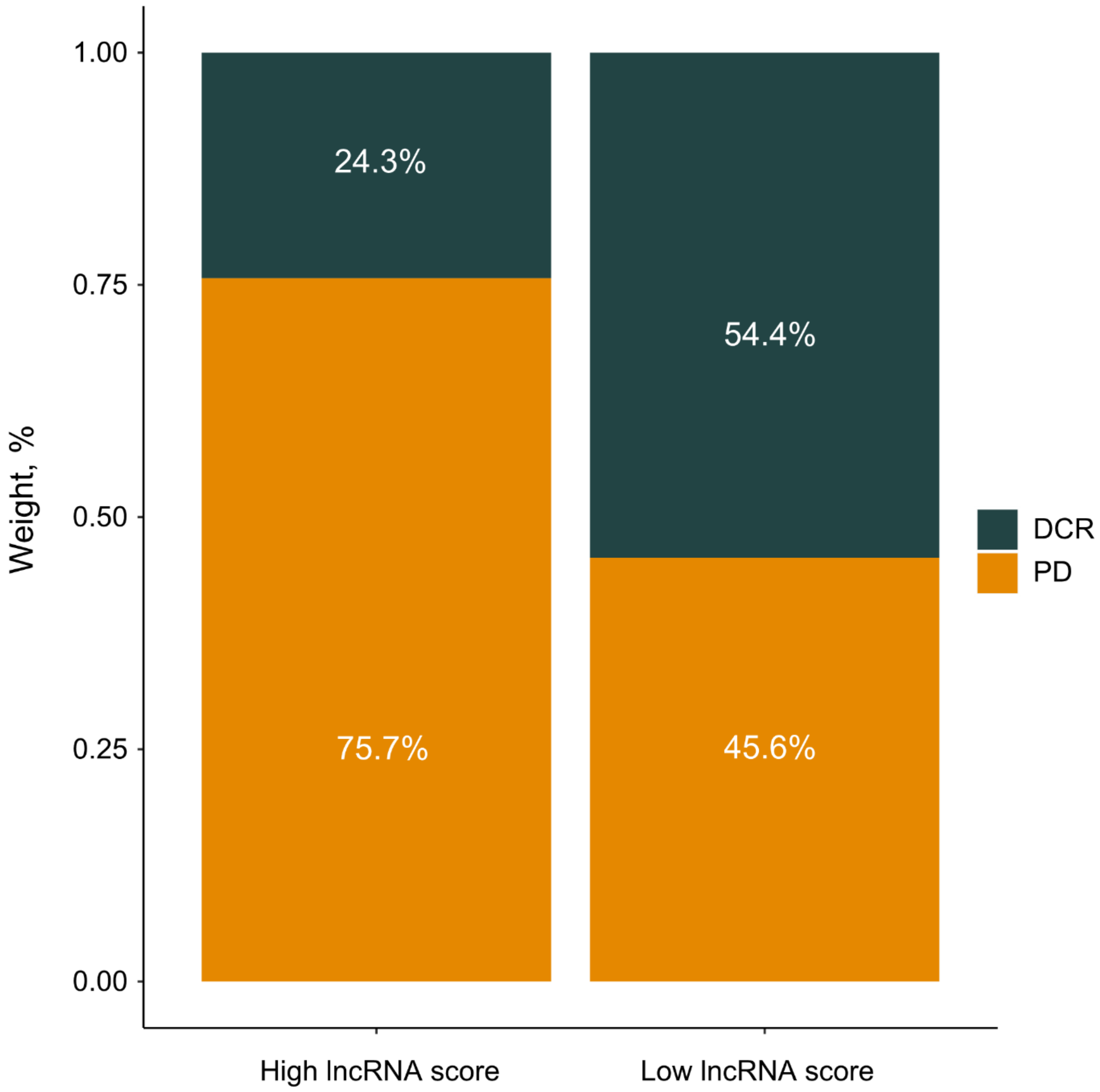

LncRNA, long non-coding RNA; DCR, disease control rate; PD, progressive disease.

© 2020 Yu Y et al. *JAMA Network Open.*

eFigure 27. Objective Response Rate stratified by LncRNA Score in Melanoma Immunotherapy Cohort.

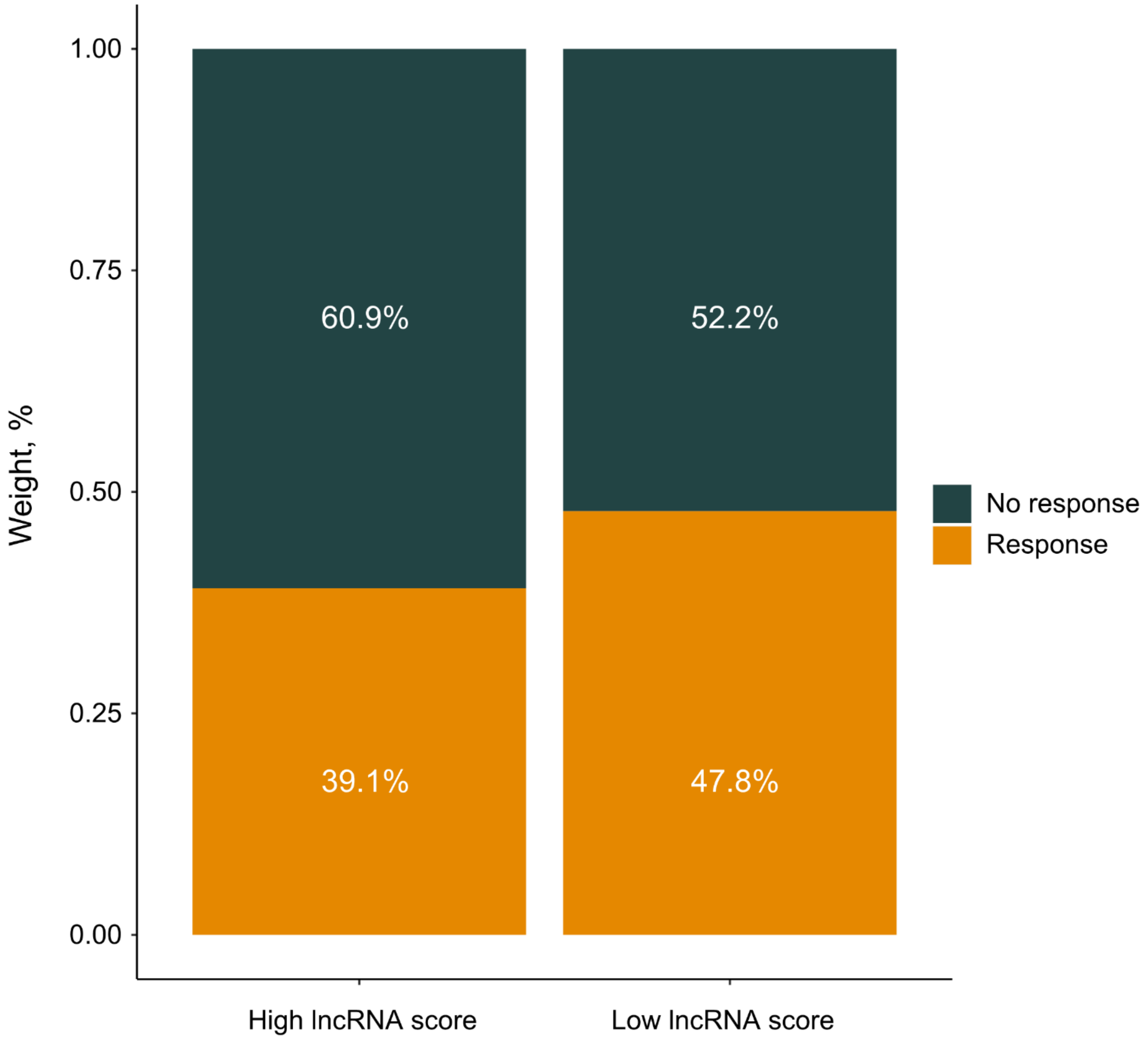

LncRNA, long non-coding RNA.

© 2020 Yu Y et al. *JAMA Network Open.*

eFigure 28. Association of LncRNA Score with Overall Survival in TCGA Cohorts.

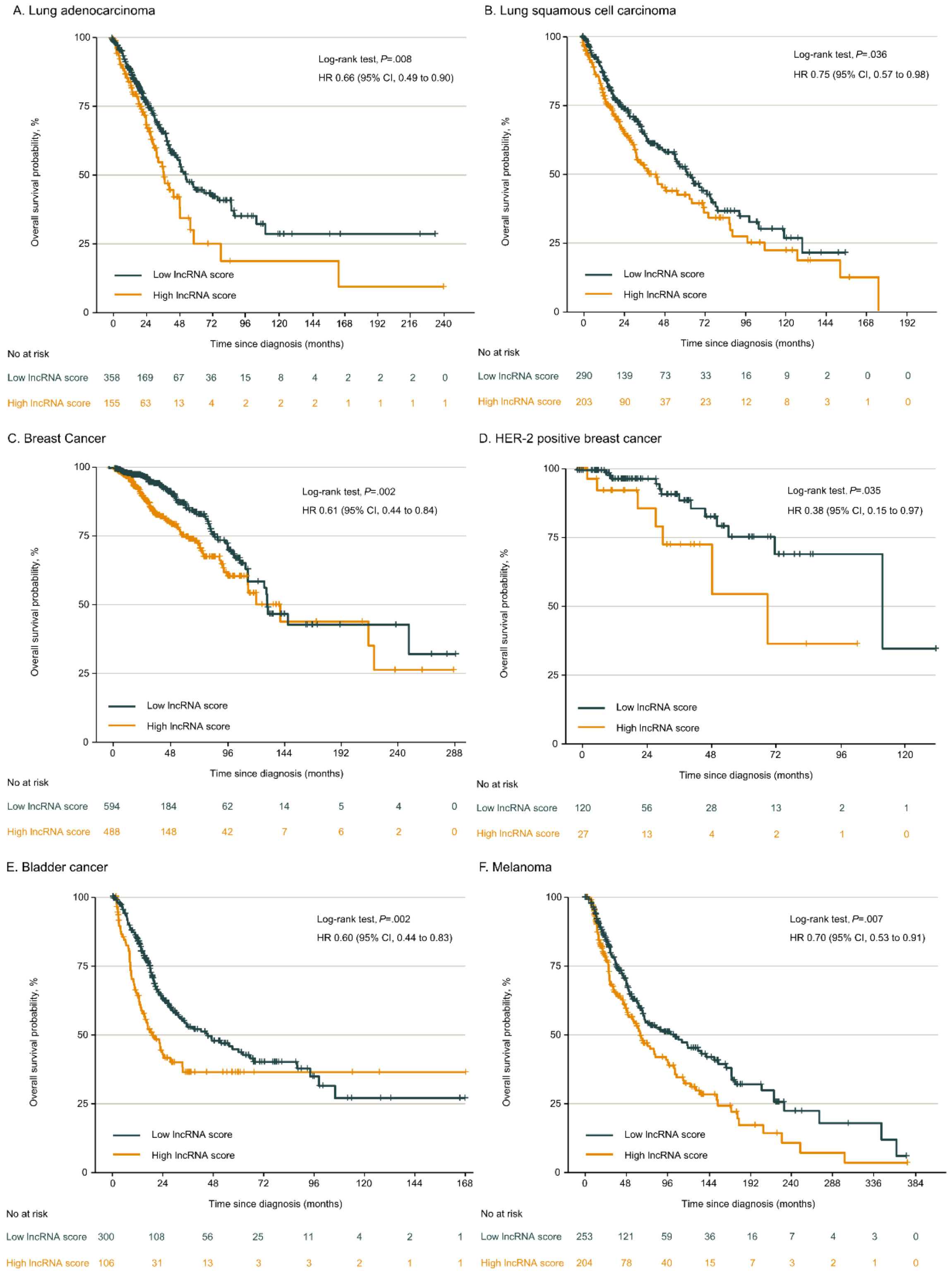

A, Lung adenocarcinoma. B, Lung squamous cell carcinoma. C, Breast Cancer. D, HER-2 positive breast cancer. E, Bladder cancer. F,

© 2020 Yu Y et al. *JAMA Network Open*.

Melanoma. LncRNA, long non-coding RNA; HR, hazard ratio; CI, confidence interval.

© 2020 Yu Y et al. *JAMA Network Open*.

eFigure 29. Association of LncRNA Score and Multiomics Biomarker with Overall Survival in IMvigor210 trial.

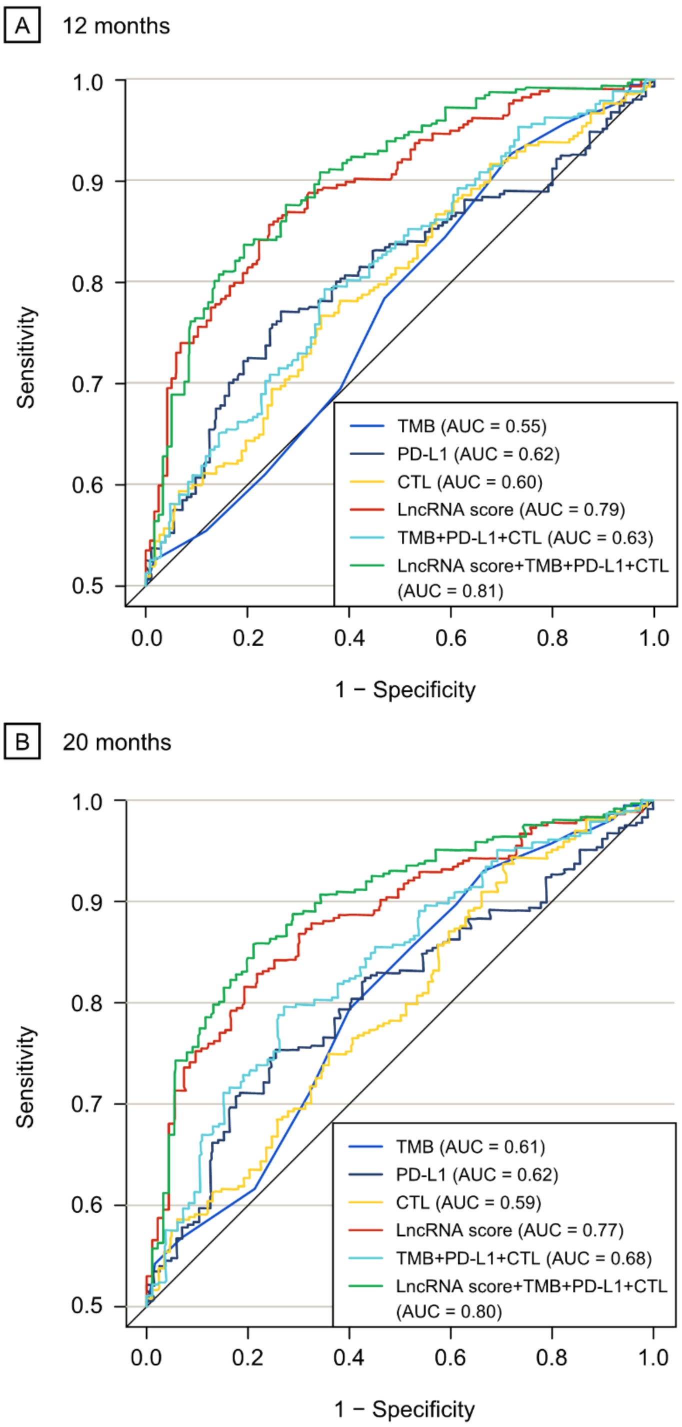

A, 12-month. B, 20-month. The lncRNA score was associated with immunotherapeutic OS benefits in IMvigor 210 trial (AUC, 12-month=0.79; 20-month=0.77) and in melanoma cohort (24-month AUC=0.87) in superior to CTL, PD-L1 expression, and TMB. We established a model combining TMB, PD-L1 expression, CTL, but did not observe a significant improvement of AUC value (12-month=0.63; 20-month=0.68) compared with single variable. We further added lncRNA to this model to build a novel multiomics algorithm and found it correlated more

strongly with OS in IMvigor 210 trial (AUC, 12-month=0.81; 20-month=0.80). LncRNA, Long Non-Coding RNA; PD-L1, programmed death-ligand 1; TMB, tumor mutation burden; AUC, area under the receiver operating characteristic curve; CTL, cytotoxic T lymphocytes.

© 2020 Yu Y et al. *JAMA Network Open*.

eFigure 30. Association of LncRNA Score with Response in IMvigor210 trial.

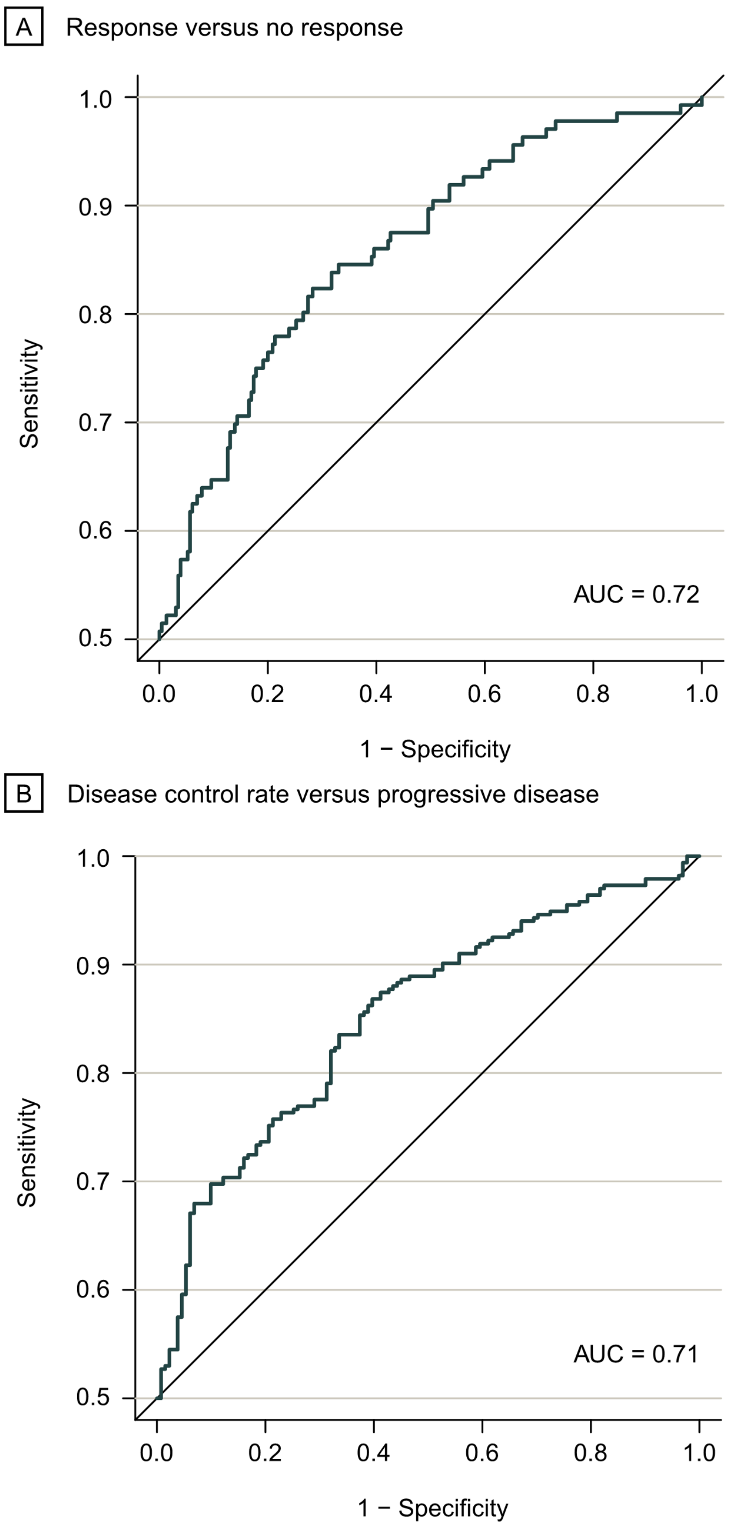

A, The ability of lncRNA score to classify patients with response versus no response. B, The ability of lncRNA score to classify patients with disease control versus progressive disease. AUC, area under the receiver operating characteristic curve.

© 2020 Yu Y et al. *JAMA Network Open.*

eTable 1. Characteristics and Clinical Outcomes of the Patients in IMvigor210 Trial.

**Characteristics/Clinical Outcomes**	**IMvigor210 Trial**
No. of patients	348
Sex (%)	
Male	272 (78.2)
Female	76 (21.8)
Race (%)	
White	318 (91.4)
Black or African American	9 (2.6)
Native Hawaiian or other pacific islander	1 (0.3)
Asian	8 (2.3)
Other	8 (2.3)
Unknown	4 (1.1)
Baseline ECOG-PS (%)	
0	134 (38.5)
1	196 (56.3)
2	18 (5.2)
Tissue (%)	
Bladder	195 (57.4)
Kidney	67 (19.7)
Liver	5 (1.5)
Lung	10 (2.9)
Lymph node	26 (7.6)
Other	11 (3.2)
Ureter	26 (7.6)
Received platinum-based chemotherapy (%)	
Yes	272 (78.2)
No	76 (21.8)
Mutation burden per Mb, mean (SD)	10.9 (9.6)
Follow up, median (IQR), months	8.1 (2.9-17.8)
TCGA Subtype (%)	
Basal/SCC-like	66 (19.0)
Genomically unstable	70 (20.1)
Infiltrated	92 (26.4)
UroA	102 (29.3)
UroB	18 (5.2)
Best confirmed overall response (%)	

© 2020 Yu Y et al. *JAMA Network Open*.

Complete response	25 (7.2)
Partial response	43 (12.4)
Stable disease	63 (18.1)
Progressive disease	167 (48.0)
Unknown	50 (14.4)

ECOG-PS, Eastern Cooperative Oncology Group Performance Status; Mb, megabase; SD, standard deviation; IQR, interquartile range; TCGA, The Cancer Genome Atlas; SCC, squamous cell carcinoma.

© 2020 Yu Y et al. *JAMA Network Open*.

eTable 2. Baseline Characteristics of the Patients in TCGA Bladder Cancer Cohort.

**Characteristic**	**TCGA Bladder Cancer Cohort**
No. of patients	406
Sex (%)	
Male	300 (73.9)
Female	106 (26.1)
Race (%)	
White	324 (79.8)
Black or African American	22 (5.4)
Asian	43 (10.6)
Unknown	17 (4.2)
Follow up, median (IQR), months	17.9 (11.0-31.6)
Tumor stage (%)	
Stage I	3 (0.7)
Stage II	128 (31.5)
Stage III	141 (34.7)
Stage IV	132 (32.5)
Unknown	2 (0.5)
History of neoadjuvant treatment (%)	
No	396 (97.5)
Yes	10 (2.5)
Lymph-vascular invasion present (%)	
Yes	149 (36.7)
No	128 (31.5)
Unknown	129 (31.8)
Neoplasm histologic grade (%)	
High grade	383 (94.3)
Low grade	20 (4.9)
Unknown	3 (0.7)
Primary lymph node presentation assessment (%)	
Yes	288 (70.9)
No	78 (19.2)
Unknown	40 (9.9)
History of radiation therapy (%)	
Yes	18 (4.4)
No	277 (68.2)
Unknown	111 (27.3)
Primary diagnosis (%)	
Papillary transitional cell carcinoma	64 (15.8)

© 2020 Yu Y et al. *JAMA Network Open.*

Transitional cell carcinoma	339 (83.5)
Other	3 (0.6)

IQR, interquartile range; TCGA, The Cancer Genome Atlas.

© 2020 Yu Y et al. *JAMA Network Open*.

eTable 3. Kyoto Encyclopedia of Genes and Genomes Pathway Enrichment Analysis of NKILA in IMvigor210 Trial

ID	Description	Gene ratio	Bg Ratio	*P* value	*P* adjust	Q value	Gene ID	Count
hsa04512	ECM-receptor interaction	23/206	86/7868	1.34E-17	3.36E-15	3.01E-15	3693/3918/2335/3371/1284/3690/3685/3678/1291/1293/1292/3909/1282/3910/1277/1278/22801/7057/7058/3673/3696/3339/284217	23
hsa04510	Focal adhesion	32/206	199/7868	5.08E-17	6.36E-15	5.70E-15	56034/87/2316/3693/3918/2335/5154/3371/1284/3690/3685/7791/3678/1291/1293/1292/7424/3909/1282/3910/1277/5155/1278/5159/22801/7057/7058/3673/3696/824/284217/29780	32
hsa04151	PI3K-Akt signaling pathway	35/206	354/7868	5.69E-12	4.74E-10	4.25E-10	56034/2246/3693/3918/2335/5154/3371/1284/9180/3690/3685/3678/1291/1293/9586/1292/7424/3909/1021/1282/3910/1277/5155/1278/5159/22801/7057/7058/2149/1435/7097/3673/3696/90993/284217	35
hsa05165	Human papillomavirus infection	30/206	330/7868	1.72E-09	1.08E-07	9.66E-08	3693/3918/2335/3371/1284/3690/3685/3678/1291/1293/2535/9586/1292/3909/1021/1282/3910/1277/355/1278/5159/22801/7057/81029/7058/3673/3696/90993/83439/284217	30
hsa04974	Protein digestion and absorption	15/206	90/7868	9.08E-09	4.54E-07	4.07E-07	1289/1284/1290/1294/80781/1291/1293/1292/1282/1277/1301/1300/1281/1278/50509	15
hsa05146	Amoebiasis	14/206	95/7868	1.44E-07	6.01E-06	5.39E-06	87/3918/2335/1284/7042/3909/1282/3910/1277/1281/1278/3684/7097/284217	14
hsa04810	Regulation of actin cytoskeleton	20/206	214/7868	7.02E-07	2.51E-05	2.25E-05	56034/87/2246/3693/2335/5154/3690/3685/3678/4478/6237/5155/54434/5159/3684/22801/2149/3673/3696/3984	20

© 2020 Yu Y et al. *JAMA Network Open.*

hsa05414	Dilated cardiomyopathy (DCM)	13/206	96/7868	1.11E-06	3.47E-05	3.11E-05	3693/6443/7042/3690/113/3685/3678/7169/22801/3673/3696/7168/284217	13
hsa05205	Proteoglycans in cancer	19/206	203/7868	1.32E-06	3.67E-05	3.29E-05	2316/3693/2335/7042/3690/5328/3685/3678/815/4478/2535/6237/355/7057/81029/7097/3673/5329/3339	19
hsa05410	Hypertrophic cardiomyopathy (HCM)	12/206	90/7868	3.43E-06	8.58E-05	7.69E-05	3693/6443/7042/3690/3685/3678/7169/22801/3673/3696/7168/284217	12
hsa05412	Arrhythmogenic right ventricular cardiomyopathy (ARVC)	11/206	77/7868	4.46E-06	0.000101429	9.10E-05	3693/6443/3690/3685/3678/1000/22801/3673/3696/83439/284217	11
ID	Description	GeneRatio	BgRatio	pvalue	p.adjust	qvalue	geneID	Count
hsa00532	Glycosaminoglycan biosynthesis - chondroitin sulfate / dermatan sulfate	6/206	20/7868	8.54E-06	0.000177974	0.000159615	50515/51363/29940/55454/337876/9469	6
hsa05222	Small cell lung cancer	10/206	93/7868	0.000147954	0.002845277	0.002551764	3918/2335/1284/3685/3909/1021/1282/3910/3673/284217	10
hsa04145	Phagosome	13/206	152/7868	0.00016585	0.00296161	0.0026561	3693/9902/3690/84617/3685/3678/2212/3684/7057/7058/7097/3673/4688	13
hsa04933	AGE-RAGE signaling pathway in diabetic complications	10/206	100/7868	0.000270397	0.004506621	0.004041728	2335/1284/7042/50507/7424/1282/1277/2152/1281/1278	10
hsa05215	Prostate cancer	9/206	97/7868	0.000945551	0.014774227	0.013250149	56034/5154/2119/5328/9586/5155/5159/90993/83439	9

© 2020 Yu Y et al. *JAMA Network Open.*

hsa04015	Rap1 signaling pathway	14/206	210/7868	0.00119227	0.017533389	0.015724681	56034/2246/5154/3690/9732/113/7424/6237/5155/5159/3684/7057/2149/1435	14
hsa04611	Platelet activation	10/206	124/7868	0.001479855	0.020553544	0.018433284	3690/113/1277/2212/1281/5592/1278/5739/2149/3673	10
hsa04926	Relaxin signaling pathway	10/206	130/7868	0.002109689	0.02775907	0.024895503	1284/113/9586/7424/1282/59350/1277/1281/1278/90993	10

© 2020 Yu Y et al. *JAMA Network Open*.

eTable 4. Gene Ontology Pathway Enrichment Analysis of NKILA in IMvigor210 Trial

Ontology	ID	Description	Gene ratio	Bg ratio	*P* value	*P* adjust	Q value	Gene ID	Count
BP	GO:0030198	extracellular matrix organization	66/434	334/17913	3.88E-41	1.47E-37	1.26E-37	8728/649/323/4241/55959/23187/3693/4017/3918/2191/2335/5154/7837/1289/3371/9509/84695/1284/7042/1307/3690/23213/1290/1294/80781/140766/4323/3685/3678/1291/30008/84168/1293/1295/1292/7076/3909/1462/165/1282/3910/1277/633/10609/1301/1300/1281/1296/11117/5155/6662/1278/3684/22801/7057/6678/3673/8038/22795/3696/90993/824/4237/3339/50509/284217	66
BP	GO:0043062	extracellular structure organization	66/434	387/17913	5.85E-37	1.11E-33	9.47E-34	8728/649/323/4241/55959/23187/3693/4017/3918/2191/2335/5154/7837/1289/3371/9509/84695/1284/7042/1307/3690/23213/1290/1294/80781/140766/4323/3685/3678/1291/30008/84168/1293/1295/1292/7076/3909/1462/165/1282/3910/1277/633/10609/1301/1300/1281/1296/11117/5155/6662/1278/3684/22801/7057/6678/3673/8038/22795/3696/90993/824/4237/3339/50509/284217	66
BP	GO:0031589	cell-substrate adhesion	40/434	322/17913	1.63E-17	2.06E-14	1.77E-14	558/3956/81792/87/342184/2316/4241/3693/2335/91663/1307/3690/51330/5328/4323/9448/3685/7791/3678/8829/84168/1295/1021/1277/23499/10631/1281/6695/11117/3897/1012/22801/7057/23603/1435/3673/22795/340485/50509/10085	40
BP	GO:0035987	endodermal cell differentiation	15/434	45/17913	8.12E-14	7.70E-11	6.58E-11	8091/3624/3693/2335/1289/1284/1290/1294/4323/3685/3678/1291/1295/3909/1301	15
BP	GO:0007160	cell-matrix adhesion	28/434	214/17913	3.59E-13	2.72E-10	2.32E-10	81792/87/342184/3693/2335/1307/3690/5328/4323/9448/3685/7791/8829/1021/23499/10631/1281/11117/3897/1012/22801/7057/23603/1435/3673/22795/340485/50509	28
BP	GO:00	endoderm	15/434	50/17913	4.75E-	3.00E-10	2.56	8091/3624/3693/2335/1289/1284/1290/1294/	15

© 2020 Yu Y et al. *JAMA Network Open.*

	01706	formation			13		E-10	4323/3685/3678/1291/1295/3909/1301	
BP	GO:00 07492	endoderm developmen t	17/434	73/17913	1.27E-12	6.80E-10	5.82 E-10	8091/3624/3693/2335/1289/1284/1290/1294/ 4323/3685/3678/1291/1295/3909/1301/53918 /2131	17
BP	GO:00 61448	connective tissue developmen t	29/434	243/1791 3	1.44E-12	6.80E-10	5.82 E-10	8091/7045/81792/649/8609/55959/2121/5051 5/4017/4094/1289/23213/353500/91252/7076 /1277/1301/7227/5155/6662/5159/81029/282 2/860/1435/2736/3696/658/1893	29
BP	GO:00 30199	collagen fibril organization	14/434	45/17913	1.64E-12	6.91E-10	5.91 E-10	4017/1289/9509/7042/1290/140766/165/1277 /10609/1301/1281/11117/1278/50509	14
BP	GO:00 32963	collagen metabolic process	18/434	102/1791 3	4.08E-11	1.55E-08	1.32 E-08	9902/2191/64175/1289/9509/7431/57333/140 766/4323/1277/10609/11117/1278/5159/1508 /2149/3673/90993	18
BP	GO:00 51216	cartilage developmen t	23/434	182/1791 3	9.73E-11	3.35E-08	2.87 E-08	8091/7045/81792/649/55959/2121/50515/401 7/4094/23213/353500/7076/1277/1301/7227/ 6662/81029/2822/860/2736/3696/658/1893	23
BP	GO:00 10810	regulation of cell-substrat e adhesion	24/434	201/1791 3	1.26E-10	3.97E-08	3.40 E-08	3956/342184/2316/4241/2335/91663/1307/53 28/4323/9448/3678/8829/1295/1021/1277/23 499/10631/6695/1012/7057/23603/1435/3404 85/10085	24
BP	GO:00 02062	chondrocyte differentiatio n	17/434	97/17913	1.60E-10	4.66E-08	3.99 E-08	8091/7045/81792/55959/50515/4017/4094/23 213/1301/7227/6662/81029/2822/860/2736/6 58/1893	17
BP	GO:00 01501	skeletal system developmen t	37/434	479/1791 3	5.42E-10	1.47E-07	1.25 E-07	8091/7045/81792/56034/649/342184/56172/5 5959/2121/50515/4017/4094/64175/56975/70 42/23213/4323/353500/7076/1277/1301/1300 /1281/7227/6662/1278/81029/6678/2822/860/ 2131/53834/2736/3696/9060/658/1893	37
BP	GO:00 01704	formation of primary germ layer	17/434	117/1791 3	3.25E-09	8.20E-07	7.01 E-07	8091/3624/3693/2335/1289/1284/3690/1290/ 1294/4323/3685/3678/1291/1295/3909/1301/ 3673	17
BP	GO:00 06029	proteoglyca n metabolic process	14/434	79/17913	5.78E-09	1.37E-06	1.17 E-06	81792/55959/50515/51363/29940/23213/554 54/1462/633/337876/1301/2131/9469/658	14
BP	GO:00	glycosamino	16/434	107/1791	6.17E-	1.37E-06	1.17	50515/51363/10402/29940/8534/55454/1462/	16

	06024	glycan biosynthetic process		3	09		E-06	633/337876/2239/5155/10082/5159/2131/9469/3339	
BP	GO:0001503	ossification	31/434	388/17913	6.50E-09	1.37E-06	1.17E-06	9260/649/56172/9902/3371/56975/25937/7042/115908/1290/4330/4323/23601/353500/1291/1462/1021/1277/1301/6662/1278/22801/6678/2822/860/1435/2131/2736/90993/658/1893	31
BP	GO:0006023	aminoglycan biosynthetic process	16/434	108/17913	7.09E-09	1.41E-06	1.21E-06	50515/51363/10402/29940/8534/55454/1462/633/337876/2239/5155/10082/5159/2131/9469/3339	16
BP	GO:0007369	gastrulation	20/434	178/17913	1.29E-08	2.44E-06	2.09E-06	8091/3624/23187/3693/2335/1289/91663/1284/3690/1290/1294/4323/3685/3678/1291/1295/3909/1301/3673/2131	20
BP	GO:0007229	integrin-mediated signaling pathway	14/434	94/17913	5.81E-08	1.05E-05	8.97E-06	2316/3693/84695/1307/3690/3685/7791/3678/8829/7076/1281/3684/22801/3673	14
BP	GO:0034330	cell junction organization	24/434	277/17913	7.63E-08	1.26E-05	1.08E-05	87/342184/2316/1002/3918/2335/7042/1307/143098/79983/4323/9448/3678/8829/1000/3909/23499/1012/7057/11228/23603/2149/3673/861	24
BP	GO:0007044	cell-substrate junction assembly	14/434	96/17913	7.64E-08	1.26E-05	1.08E-05	87/342184/3918/2335/1307/4323/9448/3678/8829/3909/23499/7057/23603/3673	14
BP	GO:0002576	platelet degranulation	16/434	128/17913	8.35E-08	1.32E-05	1.13E-05	87/2316/135228/2335/5154/7042/3690/308/7076/7424/5155/7057/6678/5265/12/1893	16
BP	GO:0030206	chondroitin sulfate biosynthetic process	8/434	26/17913	1.19E-07	1.80E-05	1.54E-05	50515/51363/29940/55454/1462/633/337876/9469	8
BP	GO:0002063	chondrocyte development	8/434	27/17913	1.65E-07	2.40E-05	2.06E-05	55959/50515/23213/1301/6662/860/658/1893	8
BP	GO:00	glycosamino	17/434	157/1791	2.74E-	3.84E-05	3.29	50515/51363/10402/29940/8534/55454/1462/	17

© 2020 Yu Y et al. *JAMA Network Open.*

	30203	glycan metabolic process		3	07		E-05	633/337876/2239/5155/10082/5159/2131/9469/3339/11261	
BP	GO:0014909	smooth muscle cell migration	13/434	91/17913	2.86E-07	3.87E-05	3.31E-05	5154/3690/9732/5328/8829/50507/10631/5155/5592/54434/5159/3673/7168	13
BP	GO:0050650	chondroitin sulfate proteoglycan biosynthetic process	8/434	29/17913	3.06E-07	3.99E-05	3.42E-05	50515/51363/29940/55454/1462/633/337876/9469	8
BP	GO:0006022	aminoglycan metabolic process	17/434	161/17913	3.93E-07	4.97E-05	4.25E-05	50515/51363/10402/29940/8534/55454/1462/633/337876/2239/5155/10082/5159/2131/9469/3339/11261	17
BP	GO:0045773	positive regulation of axon extension	9/434	41/17913	4.66E-07	5.54E-05	4.74E-05	1002/2335/8766/51330/8829/23499/3897/3984/51393	9
BP	GO:0030166	proteoglycan biosynthetic process	10/434	53/17913	4.83E-07	5.54E-05	4.74E-05	50515/51363/29940/55454/1462/633/337876/2131/9469/658	10
BP	GO:0032964	collagen biosynthetic process	10/434	53/17913	4.83E-07	5.54E-05	4.74E-05	1289/7431/57333/1277/10609/11117/5159/2149/3673/90993	10
BP	GO:0001952	regulation of cell-matrix adhesion	14/434	117/17913	9.24E-07	0.000101235	8.66E-05	342184/1307/5328/4323/9448/8829/1021/23499/10631/1012/7057/23603/1435/340485	14
BP	GO:0014910	regulation of smooth muscle cell migration	12/434	85/17913	9.35E-07	0.000101235	8.66E-05	5154/9732/5328/8829/50507/10631/5155/5592/54434/5159/3673/7168	12
BP	GO:0014812	muscle cell migration	13/434	103/17913	1.23E-06	0.000129083	0.000110387	5154/3690/9732/5328/8829/50507/10631/5155/5592/54434/5159/3673/7168	13

© 2020 Yu Y et al. *JAMA Network Open.*

BP	GO:0010811	positive regulation of cell-substrate adhesion	13/434	110/17913	2.61E-06	0.000266863	0.000228211	342184/2316/2335/91663/1307/9448/3678/8829/1295/1021/1012/1435/10085	13
BP	GO:0071230	cellular response to amino acid stimulus	10/434	64/17913	2.96E-06	0.000294887	0.000252175	56034/1307/1290/1291/1282/1277/1281/2729/1278/824	10
BP	GO:0030204	chondroitin sulfate metabolic process	8/434	39/17913	3.54E-06	0.000344277	0.000294411	50515/51363/29940/55454/1462/633/337876/9469	8
BP	GO:0010812	negative regulation of cell-substrate adhesion	10/434	67/17913	4.54E-06	0.000417838	0.000357318	3956/4241/4323/9448/1277/10631/6695/7057/23603/340485	10
BP	GO:0006929	substrate-dependent cell migration	7/434	29/17913	4.59E-06	0.000417838	0.000357318	2335/8828/51330/8829/7424/22801/3673	7
BP	GO:0034332	adherens junction organization	14/434	134/17913	4.73E-06	0.000417838	0.000357318	87/342184/1002/1307/4323/9448/8829/1000/23499/1012/7057/11228/23603/3673	14
BP	GO:0022612	gland morphogenesis	13/434	116/17913	4.74E-06	0.000417838	0.000357318	55959/5154/2119/3371/7042/23213/8829/4478/6662/1435/2736/7052/284217	13
BP	GO:0034329	cell junction assembly	18/434	214/17913	4.97E-06	0.000427966	0.000365979	87/342184/2316/3918/2335/1307/143098/79983/4323/9448/3678/8829/3909/23499/7057/23603/3673/861	18
BP	GO:0071559	response to transforming growth factor beta	19/434	239/17913	6.20E-06	0.000522493	0.000446814	56937/4053/3693/7041/50515/135228/1284/7042/7791/50507/1277/10631/1281/11117/94031/6662/1278/7057/25878	19
BP	GO:0050654	chondroitin sulfate proteoglycan metabolic	8/434	42/17913	6.38E-06	0.000525445	0.000449339	50515/51363/29940/55454/1462/633/337876/9469	8

© 2020 Yu Y et al. *JAMA Network Open.*

		process							
BP	GO:0022604	regulation of cell morphogenesis	27/434	430/17913	6.83E-06	0.000550571	0.000470826	114299/2316/4241/1002/2335/91663/8766/51330/4651/8829/91010/4478/1627/23499/2048/10631/2035/57194/3897/55785/11135/6678/23603/7168/3984/51393/29780	27
BP	GO:0034446	substrate adhesion-dependent cell spreading	11/434	86/17913	7.11E-06	0.00055499	0.000474604	558/2316/4241/2335/91663/3690/3685/8829/84168/10631/23603	11
BP	GO:0061138	morphogenesis of a branching epithelium	16/434	178/17913	7.18E-06	0.00055499	0.000474604	2246/5154/2119/3371/23213/4323/8829/1282/1909/170690/6662/5311/1435/2736/7052/284217	16
BP	GO:0045785	positive regulation of cell adhesion	25/434	388/17913	9.64E-06	0.000730885	0.000625023	8728/3956/342184/2316/2335/91663/7042/1307/9448/3685/3678/4651/8829/1295/1021/1012/1435/3673/861/2736/7292/7052/7168/10085/55083	25
BP	GO:0071774	response to fibroblast growth factor	14/434	145/17913	1.18E-05	0.000879531	0.000752139	2246/55959/3371/56975/23213/161742/1277/10631/2729/7057/860/3730/53834/90993	14
BP	GO:0032330	regulation of chondrocyte differentiation	8/434	46/17913	1.30E-05	0.000944064	0.000807325	81792/4017/4094/7227/6662/860/2736/658	8
BP	GO:0001667	ameboidal-type cell migration	27/434	446/17913	1.32E-05	0.000944813	0.000807965	2246/4017/29775/2191/2335/8828/7042/3690/8766/9448/8829/161742/7076/7424/23499/5155/79899/6662/1012/7057/22846/6678/2822/23603/3673/85360/5793	27
BP	GO:0001655	urogenital system development	22/434	324/17913	1.47E-05	0.001034313	0.000884502	342184/2246/55959/84662/5154/3371/25937/7042/23213/8829/9244/1282/3976/2048/170690/5155/6662/5159/5311/2736/5017/55083	22
BP	GO:0033627	cell adhesion mediated by	9/434	61/17913	1.50E-05	0.001034313	0.000884502	7042/1307/3690/5328/3685/3678/22801/3673/340485	9

		integrin							
BP	GO:0014911	positive regulation of smooth muscle cell migration	8/434	47/17913	1.53E-05	0.001034313	0.000884502	9732/8829/50507/10631/5155/54434/5159/3673	8
BP	GO:1901342	regulation of vasculature development	26/434	425/17913	1.59E-05	0.0010481	0.000896292	8091/2246/29775/1284/7042/8877/23213/51330/3678/8829/161742/7424/6237/2152/11117/5155/7057/22846/6678/7058/861/8038/3696/90993/3339/1893	26
BP	GO:0071560	cellular response to transforming growth factor beta stimulus	18/434	233/17913	1.60E-05	0.0010481	0.000896292	56937/4053/3693/7041/50515/135228/1284/7042/7791/50507/1277/10631/1281/11117/94031/6662/1278/7057	18
BP	GO:1903510	mucopolysaccharide metabolic process	12/434	112/17913	1.74E-05	0.00111883	0.000956778	50515/51363/10402/29940/8534/55454/1462/633/337876/5155/9469/11261	12
BP	GO:0060485	mesenchyme development	19/434	258/17913	1.84E-05	0.001154703	0.000987455	8091/23187/7041/4017/359845/2335/8828/84695/25937/7042/8829/1277/1909/54796/6662/5159/5311/23603/23414	19
BP	GO:0001763	morphogenesis of a branching structure	16/434	192/17913	1.86E-05	0.001154703	0.000987455	2246/5154/2119/3371/23213/4323/8829/1282/1909/170690/6662/5311/1435/2736/7052/284217	16
BP	GO:0010631	epithelial cell migration	23/434	354/17913	1.92E-05	0.001154703	0.000987455	2246/4017/29775/2191/8828/7042/3690/8766/9448/8829/161742/7424/23499/5155/6662/1012/7057/22846/6678/2822/23603/3673/5793	23
BP	GO:0060560	developmental growth involved in morphogenesis	17/434	214/17913	1.92E-05	0.001154703	0.000987455	342184/2246/1002/2335/8828/3371/8766/51330/8829/23499/10631/6662/3897/1435/3984/51393/55083	17

© 2020 Yu Y et al. *JAMA Network Open.*

BP	GO:0001101	response to acid chemical	22/434	330/17913	1.96E-05	0.001159269	0.000991359	56034/3371/1307/1290/80781/1291/1282/1277/1281/5155/6662/2729/1278/5159/81029/6678/5311/7097/3673/7292/340485/824	22
BP	GO:0060541	respiratory system development	16/434	193/17913	1.99E-05	0.001159269	0.000991359	2246/5154/3371/9509/84695/57333/4323/3976/6662/5159/6678/53834/2736/9242/284217/23414	16
BP	GO:0090132	epithelium migration	23/434	357/17913	2.19E-05	0.00125808	0.001075858	2246/4017/29775/2191/8828/7042/3690/8766/9448/8829/161742/7424/23499/5155/6662/1012/7057/22846/6678/2822/23603/3673/5793	23
BP	GO:0045765	regulation of angiogenesis	24/434	383/17913	2.29E-05	0.001295485	0.001107846	8091/2246/29775/1284/7042/8877/23213/51330/3678/8829/161742/6237/2152/11117/7057/22846/6678/7058/861/8038/3696/90993/3339/1893	24
BP	GO:0007045	cell-substrate adherens junction assembly	10/434	81/17913	2.54E-05	0.001395987	0.001193791	87/342184/1307/4323/9448/8829/23499/7057/23603/3673	10
BP	GO:0048041	focal adhesion assembly	10/434	81/17913	2.54E-05	0.001395987	0.001193791	87/342184/1307/4323/9448/8829/23499/7057/23603/3673	10
BP	GO:0060562	epithelial tube morphogenesis	21/434	313/17913	2.75E-05	0.001487134	0.001271736	81792/342184/2246/57822/2119/3371/7042/115908/4323/8829/2535/1282/9645/1909/170690/6662/5311/1435/2736/284217/55083	21
BP	GO:0090130	tissue migration	23/434	363/17913	2.85E-05	0.001505468	0.001287414	2246/4017/29775/2191/8828/7042/3690/8766/9448/8829/161742/7424/23499/5155/6662/1012/7057/22846/6678/2822/23603/3673/5793	23
BP	GO:0061035	regulation of cartilage development	9/434	66/17913	2.88E-05	0.001505468	0.001287414	81792/649/4017/4094/7227/6662/860/2736/658	9
BP	GO:0071229	cellular response to acid chemical	16/434	199/17913	2.90E-05	0.001505468	0.001287414	56034/3371/1307/1290/1291/1282/1277/1281/5155/6662/2729/1278/81029/5311/7292/824	16

© 2020 Yu Y et al. *JAMA Network Open.*

BP	GO:0072273	metanephric nephron morphogenesis	6/434	26/17913	2.98E-05	0.001526442	0.00130535	342184/3976/6662/5159/5311/55083	6
BP	GO:0072001	renal system development	20/434	291/17913	3.02E-05	0.001528038	0.001306716	342184/2246/55959/84662/5154/25937/7042/23213/8829/9244/1282/3976/170690/5155/6662/5159/5311/2736/5017/55083	20
BP	GO:0044344	cellular response to fibroblast growth factor stimulus	13/434	138/17913	3.14E-05	0.00156696	0.00134	2246/55959/56975/23213/161742/1277/10631/2729/7057/860/3730/53834/90993	13
BP	GO:0007266	Rho protein signal transduction	14/434	159/17913	3.36E-05	0.00165166	0.001412432	26230/9639/8829/7204/64857/1281/1012/1278/5159/55785/9828/2149/3984/55701	14
BP	GO:0010632	regulation of epithelial cell migration	20/434	294/17913	3.50E-05	0.001699495	0.001453338	2246/29775/8828/7042/3690/8766/9448/8829/161742/7424/23499/5155/6662/7057/22846/6678/2822/23603/3673/5793	20
BP	GO:0001822	kidney development	19/434	276/17913	4.66E-05	0.002233752	0.001910213	342184/2246/55959/84662/5154/25937/7042/23213/8829/9244/3976/170690/5155/6662/5159/5311/2736/5017/55083	19
BP	GO:0048008	platelet-derived growth factor receptor signaling pathway	8/434	55/17913	5.00E-05	0.002366934	0.002024105	56034/5154/8829/5782/2152/5155/5159/5739	8
BP	GO:0072210	metanephric nephron development	7/434	41/17913	5.15E-05	0.002379614	0.002034949	342184/3976/5155/6662/5159/5311/55083	7
BP	GO:1903053	regulation of extracellular matrix	7/434	41/17913	5.15E-05	0.002379614	0.002034949	4241/23187/2191/84168/165/11117/6662	7

© 2020 Yu Y et al. *JAMA Network Open.*

		organization							
BP	GO:0048754	branching morphogenesis of an epithelial tube	13/434	146/17913	5.67E-05	0.002590549	0.002215331	2246/2119/3371/4323/8829/1282/1909/170690/6662/5311/1435/2736/284217	13
BP	GO:0050772	positive regulation of axonogenesis	9/434	72/17913	5.84E-05	0.002632982	0.002251618	1002/2335/8766/51330/8829/23499/3897/3984/51393	9
BP	GO:0034333	adherens junction assembly	10/434	90/17913	6.38E-05	0.002843867	0.002431958	87/342184/1307/4323/9448/8829/23499/7057/23603/3673	10
BP	GO:0030324	lung development	14/434	169/17913	6.56E-05	0.002892039	0.002473153	2246/5154/3371/9509/84695/57333/4323/3976/6662/5159/6678/2736/284217/23414	14
BP	GO:0006909	phagocytosis	20/434	308/17913	6.70E-05	0.002920779	0.00249773	558/4240/6556/8877/3690/196051/3685/140885/4651/10417/2212/3684/7057/23603/7097/3673/7052/11031/4688/3984	20
BP	GO:0030193	regulation of blood coagulation	9/434	74/17913	7.26E-05	0.003127405	0.002674428	2191/5154/5328/2152/5155/5592/7057/2149/5329	9
BP	GO:0046578	regulation of Ras protein signal transduction	16/434	215/17913	7.37E-05	0.00313663	0.002682317	26230/7042/9639/9448/8829/22821/7204/2048/64857/1281/5159/55785/9828/2149/1435/55701	16
BP	GO:1900046	regulation of hemostasis	9/434	75/17913	8.08E-05	0.003401605	0.002908913	2191/5154/5328/2152/5155/5592/7057/2149/5329	9
BP	GO:0048010	vascular endothelial growth factor receptor signaling pathway	10/434	93/17913	8.45E-05	0.003444575	0.002945659	558/8828/3690/23213/3685/3678/8829/7424/11117/4688	10

© 2020 Yu Y et al. *JAMA Network Open.*

BP	GO:0010634	positive regulation of epithelial cell migration	14/434	173/17913	8.45E-05	0.003444575	0.002945659	2246/8828/7042/3690/8766/9448/8829/7424/5155/6662/7057/6678/2822/3673	14
BP	GO:0030323	respiratory tube development	14/434	173/17913	8.45E-05	0.003444575	0.002945659	2246/5154/3371/9509/84695/57333/4323/3976/6662/5159/6678/2736/284217/23414	14
BP	GO:0070371	ERK1 and ERK2 cascade	19/434	289/17913	8.62E-05	0.003475439	0.002972053	2335/5154/112464/3685/140885/8829/50507/161742/6237/3976/2048/11117/5155/6662/5159/8000/2149/91662/6098	19
BP	GO:0030516	regulation of axon extension	9/434	76/17913	8.97E-05	0.003578377	0.003060081	1002/2335/8766/51330/8829/23499/3897/3984/51393	9
BP	GO:0040036	regulation of fibroblast growth factor receptor signaling pathway	6/434	32/17913	0.000103727	0.00407025	0.003480711	55959/56975/23213/7057/860/90993	6
BP	GO:0051893	regulation of focal adhesion assembly	8/434	61/17913	0.000106461	0.00407025	0.003480711	342184/1307/4323/9448/8829/23499/7057/23603	8
BP	GO:0090109	regulation of cell-substrate junction assembly	8/434	61/17913	0.000106461	0.00407025	0.003480711	342184/1307/4323/9448/8829/23499/7057/23603	8
BP	GO:0048762	mesenchymal cell differentiation	15/434	199/17913	0.000106557	0.00407025	0.003480711	8091/23187/7041/4017/359845/2335/8828/84695/25937/7042/8829/1277/1909/6662/23603	15
BP	GO:0001649	osteoblast differentiatio	16/434	222/17913	0.000107394	0.00407025	0.0034807	9260/9902/3371/56975/25937/115908/23601/1291/1462/1021/1277/22801/860/2736/90993	16

© 2020 Yu Y et al. *JAMA Network Open.*

		n					11	/658	
BP	GO:0007162	negative regulation of cell adhesion	18/434	271/17913	0.000115697	0.004304876	0.003681354	7045/3956/4241/3371/84695/91663/4323/9448/1277/10631/6695/5592/1012/7057/23603/861/7292/340485	18
BP	GO:0006790	sulfur compound metabolic process	21/434	346/17913	0.000115857	0.004304876	0.003681354	50515/7915/51363/10402/29940/8534/64087/50507/23597/55454/3417/1462/9645/633/337876/2729/2687/1622/9469/56922/9060	21
BP	GO:0050818	regulation of coagulation	9/434	79/17913	0.000121555	0.004472758	0.003824919	2191/5154/5328/2152/5155/5592/7057/2149/5329	9
BP	GO:0003338	metanephros morphogenesis	6/434	33/17913	0.000124208	0.00452642	0.003870809	342184/3976/6662/5159/5311/55083	6
BP	GO:0007178	transmembrane receptor protein serine/threonine kinase signaling pathway	20/434	323/17913	0.000128251	0.004629253	0.003958747	56937/3624/4053/3693/7041/50515/135228/25937/7042/23213/7431/7791/1281/11117/94031/1278/7057/53918/860/658	20
BP	GO:0007265	Ras protein signal transduction	20/434	324/17913	0.000133695	0.004754967	0.004066253	26230/7042/9639/9448/8829/22821/7204/6237/2048/64857/1281/1012/1278/5159/55785/9828/2149/1435/3984/55701	20
BP	GO:2000027	regulation of animal organ morphogenesis	17/434	250/17913	0.000134243	0.004754967	0.004066253	2246/55959/57822/5154/2119/7042/115908/23213/51339/2535/3976/2239/10082/6662/860/1435/4919	17
BP	GO:0010770	positive regulation of cell morphogenesis involved	12/434	138/17913	0.0001 3643	0.004760878	0.004071308	2316/1002/2335/91663/8766/51330/8829/1627/23499/3897/3984/51393	12

© 2020 Yu Y et al. *JAMA Network Open.*

		in differentiation							
BP	GO:1905330	regulation of morphogenesis of an epithelium	14/434	181/17913	0.000136922	0.004760878	0.004071308	81792/2246/57822/5154/2119/115908/23213/51339/2535/3976/2239/10082/6662/4919	14
BP	GO:0090287	regulation of cellular response to growth factor stimulus	18/434	276/17913	0.000145523	0.005013942	0.004287718	56937/81792/2246/55959/7041/50515/135228/56975/3690/23213/3678/7424/11117/94031/7057/53918/860/90993	18
BP	GO:0010712	regulation of collagen metabolic process	7/434	49/17913	0.000166515	0.005634743	0.004818601	2191/7431/11117/5159/2149/3673/90993	7
BP	GO:0030195	negative regulation of blood coagulation	7/434	49/17913	0.000166515	0.005634743	0.004818601	2191/5154/5328/5155/5592/7057/5329	7
BP	GO:0048675	axon extension	10/434	101/17913	0.000169155	0.005673442	0.004851695	1002/2335/8828/8766/51330/8829/23499/3897/3984/51393	10
BP	GO:0048839	inner ear development	14/434	185/17913	0.000172333	0.005729331	0.004899489	57822/4094/7042/115908/4610/2535/2048/1301/6662/5159/6678/2736/1687/4919	14
BP	GO:0007596	blood coagulation	20/434	331/17913	0.000177856	0.005841364	0.004995295	2316/2191/5154/3690/5328/308/1277/2152/1281/5155/5592/1278/7057/2149/3673/30846/9060/5329/5265/23414	20
BP	GO:0072006	nephron development	12/434	142/17913	0.000178786	0.005841364	0.004995295	342184/2246/55959/25937/23213/3976/170690/5155/6662/5159/5311/55083	12
BP	GO:0007264	small GTPase mediated	25/434	465/17913	0.000183218	0.005935002	0.005075371	26230/7042/9732/9639/58504/9448/8829/22821/7204/6237/2048/64857/1281/1012/1278/5159/55785/9828/2149/1435/85360/3984/1126	25

		signal transduction						1/57186/55701	
BP	GO:1900047	negative regulation of hemostasis	7/434	50/17913	0.000189636	0.00609084	0.005208636	2191/5154/5328/5155/5592/7057/5329	7
BP	GO:0072215	regulation of metanephros development	5/434	23/17913	0.000191375	0.006095048	0.005212235	5154/25937/3976/5155/5159	5
BP	GO:0070208	protein heterotrimerization	4/434	13/17913	0.000204243	0.006450664	0.005516344	1291/1292/1277/1278	4
BP	GO:1903391	regulation of adherens junction organization	8/434	67/17913	0.000207784	0.006508289	0.005565622	342184/1307/4323/9448/8829/23499/7057/23603	8
BP	GO:0001954	positive regulation of cell-matrix adhesion	7/434	51/17913	0.000215281	0.006521621	0.005577023	342184/1307/9448/8829/1021/1012/1435	7
BP	GO:0048146	positive regulation of fibroblast proliferation	7/434	51/17913	0.000215281	0.006521621	0.005577023	56034/2335/5154/8877/1021/5155/5159	7
BP	GO:0008360	regulation of cell shape	11/434	124/17913	0.000215985	0.006521621	0.005577023	114299/2335/4651/91010/4478/2035/57194/55785/11135/7168/29780	11
BP	GO:0035023	regulation of Rho protein signal transduction	11/434	124/17913	0.000215985	0.006521621	0.005577023	26230/9639/8829/7204/64857/1281/5159/55785/9828/2149/55701	11
BP	GO:0007599	hemostasis	20/434	336/17913	0.000216814	0.006521621	0.005577023	2316/2191/5154/3690/5328/308/1277/2152/1281/5155/5592/1278/7057/2149/3673/30846/9060/5329/5265/23414	20
BP	GO:00	regulation of	26/434	499/1791	0.0002	0.006675	0.005	2316/9612/57822/2191/5154/3690/8766/5328	26

	50878	body fluid levels		3	23922	362	708496	/113/308/1277/2152/2048/1281/5155/5592/1278/6338/7057/2149/3673/30846/9060/5329/5265/23414	
BP	GO:0050817	coagulation	20/434	337/17913	0.000225448	0.006675362	0.005708496	2316/2191/5154/3690/5328/308/1277/2152/1281/5155/5592/1278/7057/2149/3673/30846/9060/5329/5265/23414	20
BP	GO:0090103	cochlea morphogenesis	5/434	24/17913	0.000236959	0.006961823	0.005953466	57822/115908/2535/6662/2736	5
BP	GO:0030574	collagen catabolic process	6/434	37/17913	0.000240231	0.007003666	0.005989248	9902/2191/9509/140766/4323/1508	6
BP	GO:0043200	response to amino acid	10/434	106/17913	0.000251955	0.007289378	0.006233577	56034/1307/1290/1291/1282/1277/1281/2729/1278/824	10
BP	GO:0050808	synapse organization	22/434	394/17913	0.000262729	0.007543501	0.006450893	87/8828/2119/3371/58504/8829/1000/1282/1627/2048/2239/3897/54434/1821/3684/6678/7058/2149/7097/22891/5098/11346	22
BP	GO:0051056	regulation of small GTPase mediated signal transduction	19/434	316/17913	0.000272976	0.007759433	0.006635549	26230/7042/9639/58504/9448/8829/22821/7204/2048/64857/1281/5159/55785/9828/2149/1435/85360/57186/55701	19
BP	GO:0050819	negative regulation of coagulation	7/434	53/17913	0.000274955	0.007759433	0.006635549	2191/5154/5328/5155/5592/7057/5329	7
BP	GO:0043277	apoptotic cell clearance	6/434	38/17913	0.000279505	0.007759433	0.006635549	558/4240/3690/3685/7057/7052	6
BP	GO:0035791	platelet-derived growth factor receptor-beta signaling pathway	4/434	14/17913	0.000280486	0.007759433	0.006635549	5154/5782/5155/5159	4

© 2020 Yu Y et al. *JAMA Network Open.*

BP	GO:0072216	positive regulation of metanephros development	4/434	14/17913	0.000280486	0.007759433	0.006635549	5154/3976/5155/5159	4
BP	GO:0050673	epithelial cell proliferation	23/434	425/17913	0.000299951	0.00823778	0.007044612	2246/55959/4017/135228/2191/8828/7042/3690/23213/4323/8829/1295/1021/2152/1296/5155/6662/1012/7057/22846/6678/9572/1893	23
BP	GO:0008543	fibroblast growth factor receptor signaling pathway	10/434	109/17913	0.000316246	0.008622825	0.007373887	2246/55959/56975/23213/161742/7057/860/3730/53834/90993	10
BP	GO:0061387	regulation of extent of cell growth	9/434	90/17913	0.000330119	0.008873424	0.007588188	1002/2335/8766/51330/8829/23499/3897/3984/51393	9
BP	GO:1901888	regulation of cell junction assembly	9/434	90/17913	0.000330119	0.008873424	0.007588188	342184/1307/4323/9448/8829/23499/7057/23603/861	9
BP	GO:0070372	regulation of ERK1 and ERK2 cascade	17/434	271/17913	0.00034809	0.009290561	0.007944907	2335/5154/112464/140885/8829/50507/161742/6237/3976/2048/11117/5155/5159/8000/2149/91662/6098	17
BP	GO:0032967	positive regulation of collagen biosynthetic process	5/434	26/17913	0.000352383	0.009339379	0.007986654	7431/5159/2149/3673/90993	5
BP	GO:0001656	metanephros development	9/434	91/17913	0.000358655	0.009439593	0.008072353	342184/5154/25937/3976/5155/6662/5159/5311/55083	9
BP	GO:0050651	dermatan sulfate	4/434	15/17913	0.000375192	0.009797999	0.0083788	29940/55454/1462/633	4

© 2020 Yu Y et al. *JAMA Network Open.*

		proteoglycan biosynthetic process					47		
BP	GO:0061045	negative regulation of wound healing	8/434	73/17913	0.000377443	0.009797999	0.008378847	135228/2191/5154/5328/5155/5592/7057/5329	8
BP	GO:0010714	positive regulation of collagen metabolic process	5/434	27/17913	0.000423938	0.010930102	0.009346975	7431/5159/2149/3673/90993	5
BP	GO:0001953	negative regulation of cell-matrix adhesion	6/434	42/17913	0.000489479	0.012397122	0.010601511	4323/9448/10631/7057/23603/340485	6
BP	GO:0050655	dermatan sulfate proteoglycan metabolic process	4/434	16/17913	0.00049073	0.012397122	0.010601511	29940/55454/1462/633	4
BP	GO:0060993	kidney morphogenesis	9/434	95/17913	0.000493922	0.012397122	0.010601511	342184/2246/25937/3976/170690/6662/5159/5311/55083	9
BP	GO:0061326	renal tubule development	9/434	95/17913	0.000493922	0.012397122	0.010601511	342184/2246/25937/1282/3976/170690/6662/5311/55083	9
BP	GO:0085029	extracellular matrix assembly	5/434	28/17913	0.000505923	0.012614783	0.010787645	30008/84168/11117/6662/1278	5
BP	GO:0007179	transforming growth factor beta receptor signaling	13/434	184/17913	0.000563777	0.013965461	0.011942669	56937/4053/3693/7041/50515/135228/7042/7791/1281/11117/94031/1278/7057	13

© 2020 Yu Y et al. *JAMA Network Open.*

		pathway							
BP	GO:0001935	endothelial cell proliferation	13/434	185/17913	0.000593363	0.014602896	0.012487798	4017/8828/3690/23213/4323/8829/2152/5155/1012/7057/22846/6678/1893	13
BP	GO:0040037	negative regulation of fibroblast growth factor receptor signaling pathway	4/434	17/17913	0.000629515	0.015217743	0.0130135 9	55959/23213/7057/90993	4
BP	GO:0061298	retina vasculature development in camera-type eye	4/434	17/17913	0.000629515	0.015217743	0.0130135 9	8829/1282/5159/284217	4
BP	GO:0032965	regulation of collagen biosynthetic process	6/434	44/17913	0.000632366	0.015217743	0.0130135 9	7431/11117/5159/2149/3673/90993	6
BP	GO:0010769	regulation of cell morphogenesis involved in differentiation	16/434	260/17913	0.000634407	0.015217743	0.0130135 9	2316/4241/1002/2335/91663/8766/51330/8829/1627/23499/2048/10631/3897/23603/3984/51393	16
BP	GO:0072028	nephron morphogenesis	8/434	79/17913	0.000645819	0.015394039	0.013164351	342184/2246/3976/170690/6662/5159/5311/55083	8
BP	GO:0043583	ear development	14/434	212/17913	0.000687552	0.016286391	0.013927454	57822/4094/7042/115908/4610/2535/2048/1301/6662/5159/6678/2736/1687/4919	14
BP	GO:00	regulation of	11/434	143/1791	0.0007	0.017246	0.014	135228/2191/5154/51330/5328/2152/5155/55	11

© 2020 Yu Y et al. *JAMA Network Open.*

	61041	wound healing		3	32634	488	748489	92/7057/2149/5329	
BP	GO:0030307	positive regulation of cell growth	12/434	166/17913	0.000743557	0.017395561	0.014875971	1002/2335/7042/8877/8766/51330/4323/8829/23499/3897/3984/51393	12
BP	GO:0048608	reproductive structure development	22/434	426/17913	0.000754023	0.017532186	0.014992806	3624/8728/558/2316/3371/7042/23213/4323/3417/3976/5155/6662/5159/10046/22846/79733/5311/1508/2736/3696/658/23414	22
BP	GO:0044272	sulfur compound biosynthetic process	13/434	190/17913	0.000761724	0.017603261	0.015053587	50515/51363/10402/29940/8534/55454/1462/633/337876/2729/2687/9469/9060	13
BP	GO:1901343	negative regulation of vasculature development	13/434	191/17913	0.000799814	0.018371484	0.01571054	29775/1284/7042/23213/161742/11117/5155/7057/22846/6678/7058/90993/3339	13
BP	GO:0061036	positive regulation of cartilage development	5/434	31/17913	0.000823999	0.018683567	0.01597742	649/4017/6662/860/658	5
BP	GO:0061458	reproductive system development	22/434	429/17913	0.000826577	0.018683567	0.01597742	3624/8728/558/2316/3371/7042/23213/4323/3417/3976/5155/6662/5159/10046/22846/79733/5311/1508/2736/3696/658/23414	22
BP	GO:0150063	visual system development	19/434	346/17913	0.00082819	0.018683567	0.01597742	3624/57822/1289/7042/1290/7431/8829/1295/1282/2048/1296/6662/5159/3684/84440/81029/4237/658/284217	19
BP	GO:1990138	neuron projection extension	11/434	148/17913	0.000973558	0.021490087	0.018377441	1002/2335/8828/8766/51330/8829/23499/10631/3897/3984/51393	11
BP	GO:0048880	sensory system	19/434	351/17913	0.000981344	0.021490087	0.0183774	3624/57822/1289/7042/1290/7431/8829/1295/1282/2048/1296/6662/5159/3684/84440/810	19

		development					41	29/4237/658/284217	
BP	GO:0001502	cartilage condensation	4/434	19/17913	0.000986616	0.021490087	0.018377441	649/1301/6662/658	4
BP	GO:0032332	positive regulation of chondrocyte differentiation	4/434	19/17913	0.000986616	0.021490087	0.018377441	4017/6662/860/658	4
BP	GO:0071071	regulation of phospholipid biosynthetic process	4/434	19/17913	0.000986616	0.021490087	0.018377441	5154/3417/5155/824	4
BP	GO:0072077	renal vesicle morphogenesis	4/434	19/17913	0.000986616	0.021490087	0.018377441	342184/3976/6662/55083	4
BP	GO:0051668	localization within membrane	10/434	126/17913	0.000993801	0.021522898	0.0184055	201134/2316/2119/3690/1000/1627/2239/10082/1012/54434	10
BP	GO:0045995	regulation of embryonic development	10/434	128/17913	0.001121309	0.024146365	0.020648982	23187/57822/1289/91663/23213/1290/2535/3909/3910/284217	10
BP	GO:0035272	exocrine system development	6/434	49/17913	0.001131538	0.024228986	0.020719636	5154/7042/8829/6662/7052/284217	6
BP	GO:0016055	Wnt signaling pathway	24/434	498/17913	0.001152182	0.024532407	0.020979108	55959/7041/57822/25937/5218/115908/23213/64359/1000/815/51339/2535/1277/23499/2239/80114/10082/6662/81029/5311/7097/861/83439/4919	24
BP	GO:0048568	embryonic organ development	21/434	413/17913	0.001197817	0.024791691	0.021200838	56034/50515/57822/5154/7042/115908/4323/2535/3976/9645/2048/1301/5155/6662/22846/79733/5311/860/2736/4237/23414	21

© 2020 Yu Y et al. *JAMA Network Open.*

BP	GO:0010869	regulation of receptor biosynthetic process	4/434	20/17913	0.001209864	0.024791691	0.021200838	3690/3685/22937/9572	4
BP	GO:0072087	renal vesicle development	4/434	20/17913	0.001209864	0.024791691	0.021200838	342184/3976/6662/55083	4
BP	GO:0072234	metanephric nephron tubule development	4/434	20/17913	0.001209864	0.024791691	0.021200838	25937/3976/6662/5311	4
BP	GO:0098743	cell aggregation	4/434	20/17913	0.001209864	0.024791691	0.021200838	649/1301/6662/658	4
BP	GO:0001764	neuron migration	11/434	152/17913	0.001210433	0.024791691	0.021200838	558/323/2316/8828/8829/815/1281/6695/5592/7155/54820	11
BP	GO:0198738	cell-cell signaling by wnt	24/434	500/17913	0.001216257	0.024791691	0.021200838	55959/7041/57822/25937/5218/115908/23213/64359/1000/815/51339/2535/1277/23499/2239/80114/10082/6662/81029/5311/7097/861/83439/4919	24
BP	GO:0009612	response to mechanical stimulus	13/434	200/17913	0.001220525	0.024791691	0.021200838	3371/2115/4323/1277/10631/1301/1281/355/6662/2729/7057/5311/3673	13
BP	GO:1903035	negative regulation of response to wounding	8/434	87/17913	0.001223231	0.024791691	0.021200838	135228/2191/5154/5328/5155/5592/7057/5329	8
BP	GO:0007435	salivary gland morphogenesis	5/434	34/17913	0.001271405	0.02549537	0.021802596	5154/7042/8829/7052/284217	5
BP	GO:0018149	peptide cross-linking	5/434	34/17913	0.001271405	0.02549537	0.021802596	2335/633/1281/7057/7052	5

© 2020 Yu Y et al. *JAMA Network Open.*

BP	GO:0016525	negative regulation of angiogenesis	12/434	177/17913	0.001299666	0.025924919	0.022169928	29775/1284/7042/23213/161742/11117/7057/22846/6678/7058/90993/3339	12
BP	GO:0043687	post-translational protein modification	19/434	360/17913	0.001318549	0.026163869	0.022374268	3956/84141/4241/4240/2335/64175/3371/56975/51088/8766/1000/7076/1462/114907/2822/1435/23194/83987/5265	19
BP	GO:1900024	regulation of substrate adhesion-dependent cell spreading	6/434	51/17913	0.001399273	0.027621062	0.0236204	2316/4241/91663/8829/10631/23603	6
BP	GO:2000181	negative regulation of blood vessel morphogenesis	12/434	179/17913	0.001430723	0.02809554	0.024026154	29775/1284/7042/23213/161742/11117/7057/22846/6678/7058/90993/3339	12
BP	GO:0003094	glomerular filtration	4/434	21/17913	0.001466196	0.028351448	0.024244996	55959/23213/5155/2149	4
BP	GO:0071711	basement membrane organization	4/434	21/17913	0.001466196	0.028351448	0.024244996	23187/3918/1282/22795	4
BP	GO:1903055	positive regulation of extracellular matrix organization	4/434	21/17913	0.001466196	0.028351448	0.024244996	4241/23187/11117/6662	4
BP	GO:0090288	negative regulation of cellular response to growth factor stimulus	11/434	156/17913	0.001492814	0.028433214	0.024314919	56937/81792/55959/7041/50515/135228/23213/11117/94031/7057/90993	11
BP	GO:00	low-density	3/434	10/17913	0.0014	0.028433	0.024	3690/3685/22937	3

© 2020 Yu Y et al. *JAMA Network Open.*

	45713	lipoprotein particle receptor biosynthetic process			92931	214	3149 19		
BP	GO:00 61299	retina vasculature morphogene sis in camera-type eye	3/434	10/17913	0.0014 92931	0.028433 214	0.024 3149 19	8829/1282/284217	3
BP	GO:00 50764	regulation of phagocytosi s	8/434	90/17913	0.0015 23978	0.028879 381	0.024 6964 63	4240/6556/8877/3685/140885/7097/3673/110 31	8
BP	GO:00 07409	axonogenes is	20/434	393/1791 3	0.0015 38604	0.029011 493	0.024 8094 39	9260/8609/323/1002/2335/8828/8766/51330/ 2115/8829/23499/2048/3897/7155/3730/2131 /2736/658/3984/51393	20
BP	GO:19 04018	positive regulation of vasculature developmen t	14/434	232/1791 3	0.0016 33843	0.030654 785	0.026 2147 15	8091/2246/8877/3678/8829/7424/6237/2152/ 5155/7057/861/8038/3696/1893	14
BP	GO:00 50769	positive regulation of neurogenesi s	22/434	455/1791 3	0.0017 51498	0.032344 957	0.027 6600 82	2316/1002/2335/2119/64175/8766/51330/882 9/7424/23529/1627/3976/23499/2048/3897/2 3531/1435/7097/2736/22891/3984/51393	22
BP	GO:00 32799	low-density lipoprotein receptor particle metabolic process	4/434	22/17913	0.0017 58064	0.032344 957	0.027 6600 82	3690/3685/22937/29116	4
BP	GO:00 50927	positive regulation of positive chemotaxis	4/434	22/17913	0.0017 58064	0.032344 957	0.027 6600 82	7424/2152/1012/3673	4

© 2020 Yu Y et al. *JAMA Network Open.*

BP	GO:0097205	renal filtration	4/434	22/17913	0.001758064	0.032344957	0.027660082	55959/23213/5155/2149	4
BP	GO:0001654	eye development	18/434	342/17913	0.001803426	0.033019257	0.028236715	3624/57822/1289/7042/1290/7431/8829/1295/1282/2048/1296/6662/5159/84440/81029/4237/658/284217	18
BP	GO:0072080	nephron tubule development	8/434	93/17913	0.0018 8055	0.034105199	0.029165368	342184/2246/25937/3976/170690/6662/5311/55083	8
BP	GO:0070206	protein trimerization	6/434	54/17913	0.001888199	0.034105199	0.029165368	1291/1292/1277/11117/1278/241	6
BP	GO:0007411	axon guidance	13/434	210/17913	0.001889734	0.034105199	0.029165368	9260/8609/323/1002/8828/2115/8829/2048/3897/3730/2131/2736/658	13
BP	GO:0048863	stem cell differentiation	14/434	237/17913	0.001992638	0.035791931	0.030607792	8091/2335/8828/7042/8829/81606/1021/3976/4054/1909/6662/860/23603/861	14
BP	GO:0031915	positive regulation of synaptic plasticity	3/434	11/17913	0.002016041	0.035872281	0.030676504	1627/2048/54434	3
BP	GO:1901201	regulation of extracellular matrix assembly	3/434	11/17913	0.002016041	0.035872281	0.030676504	84168/11117/6662	3
BP	GO:0097485	neuron projection guidance	13/434	212/17913	0.002054542	0.0359687	0.030758958	9260/8609/323/1002/8828/2115/8829/2048/3897/3730/2131/2736/658	13
BP	GO:0050866	negative regulation of cell activation	12/434	187/17913	0.002068889	0.0359687	0.030758958	3624/558/5154/84695/11117/5155/5592/2729/861/7292/9572/133396	12
BP	GO:0070373	negative regulation of	7/434	74/17913	0.002078581	0.0359687	0.0307589	140885/161742/3976/2048/11117/8000/91662	7

		ERK1 and ERK2 cascade					58		
BP	GO:0050926	regulation of positive chemotaxis	4/434	23/17913	0.002087893	0.0359687	0.030758958	7424/2152/1012/3673	4
BP	GO:0060445	branching involved in salivary gland morphogenesis	4/434	23/17913	0.002087893	0.0359687	0.030758958	5154/8829/7052/284217	4
BP	GO:0072170	metanephric tubule development	4/434	23/17913	0.002087893	0.0359687	0.030758958	25937/3976/6662/5311	4
BP	GO:0072243	metanephric nephron epithelium development	4/434	23/17913	0.002087893	0.0359687	0.030758958	25937/3976/6662/5311	4
BP	GO:0007431	salivary gland development	5/434	38/17913	0.002118753	0.036171505	0.030932388	5154/7042/8829/7052/284217	5
BP	GO:0071604	transforming growth factor beta production	5/434	38/17913	0.002118753	0.036171505	0.030932388	7042/3685/4054/7057/3696	5
BP	GO:0014066	regulation of phosphatidylinositol 3-kinase signaling	9/434	117/17913	0.002180085	0.037051676	0.031685075	5154/7042/8870/5155/79899/6662/5159/25827/2149	9
BP	GO:0001837	epithelial to mesenchym	10/434	140/17913	0.002196376	0.037161889	0.0317793	8091/23187/7041/4017/359845/84695/25937/7042/1277/6662	10

		al transition					24		
BP	GO:0060348	bone development	12/434	189/17913	0.0022 6037	0.038074678	0.032559904	56034/55959/64175/56975/23213/4323/1277/6662/6678/860/9060/658	12
BP	GO:0017145	stem cell division	5/434	39/17913	0.0023 82739	0.039958325	0.034170722	2119/25937/7042/81606/26354	5
BP	GO:0061564	axon development	21/434	438/17913	0.0024 41499	0.040763357	0.034859152	9260/8609/323/1002/2335/8828/3371/8766/51330/2115/8829/23499/2048/3897/7155/3730/2131/2736/658/3984/51393	21
BP	GO:0032800	receptor biosynthetic process	4/434	24/17913	0.0024 5808	0.040860183	0.034941953	3690/3685/22937/9572	4
BP	GO:0090183	regulation of kidney development	6/434	57/17913	0.0024 96581	0.041318962	0.035334283	5154/25937/3976/5155/6662/5159	6
BP	GO:0048708	astrocyte differentiation	7/434	77/17913	0.0026 11194	0.042577097	0.036410188	7431/1021/23529/3976/6662/4919/9572	7
BP	GO:0072088	nephron epithelium morphogenesis	7/434	77/17913	0.0026 11194	0.042577097	0.036410188	342184/2246/3976/170690/6662/5311/55083	7
BP	GO:0048667	cell morphogenesis involved in neuron differentiation	23/434	500/17913	0.0026 21791	0.042577097	0.036410188	9260/8609/323/1002/2335/8828/8766/51330/2115/8829/815/1627/23499/2048/3897/7155/3730/2131/2736/22891/658/3984/51393	23
BP	GO:0002468	dendritic cell antigen processing and presentation	3/434	12/17913	0.0026 40005	0.042577097	0.036410188	6556/7057/50856	3
BP	GO:00	dermatan	3/434	12/17913	0.0026	0.042577	0.036	29940/1462/633	3

© 2020 Yu Y et al. *JAMA Network Open.*

	30208	sulfate biosynthetic process			40005	097	4101 88		
BP	GO:00 90177	establishment of planar polarity involved in neural tube closure	3/434	12/17913	0.0026 40005	0.042577 097	0.036 4101 88	57822/115908/2535	3
BP	GO:00 31952	regulation of protein autophosphorylation	5/434	40/17913	0.0026 69835	0.042875 741	0.036 6655 76	5154/7424/5155/91662/11261	5
BP	GO:00 32233	positive regulation of actin filament bundle assembly	6/434	58/17913	0.0027 28864	0.043638 802	0.037 3181 15	2316/9639/8829/50507/7168/3984	6
BP	GO:00 46718	viral entry into host cell	9/434	121/17913	0.0027 40936	0.043647 686	0.037 3257 12	558/3956/3693/3690/3685/23601/3678/1508/3673	9
BP	GO:00 42730	fibrinolysis	4/434	25/17913	0.0028 70981	0.045149 449	0.038 6099 58	2191/5328/7057/5329	4
BP	GO:00 51894	positive regulation of focal adhesion assembly	4/434	25/17913	0.0028 70981	0.045149 449	0.038 6099 58	342184/1307/9448/8829	4
BP	GO:00 60740	prostate gland epithelium morphogenesis	4/434	25/17913	0.0028 70981	0.045149 449	0.038 6099 58	3371/23213/6662/2736	4
BP	GO:00	regulation of	11/434	170/1791	0.0029	0.045777	0.039	8828/3690/23213/8829/2152/5155/1012/7057	11

© 2020 Yu Y et al. *JAMA Network Open.*

	01936	endothelial cell proliferation		3	35103	947	1474 24	/22846/6678/1893	
BP	GO:00 48771	tissue remodeling	11/434	170/1791 3	0.0029 35103	0.045777 947	0.039 1474 24	558/115908/3690/4323/50507/7076/3976/633 /10609/2149/7052	11
BP	GO:00 06911	phagocytosi s, engulfment	5/434	41/17913	0.0029 81174	0.046116 931	0.039 4373 09	4240/3684/7057/3673/11031	5
BP	GO:00 90184	positive regulation of kidney developmen t	5/434	41/17913	0.0029 81174	0.046116 931	0.039 4373 09	5154/3976/5155/6662/5159	5
BP	GO:00 43542	endothelial cell migration	15/434	275/1791 3	0.0030 17262	0.046348 842	0.039 6356 3	2246/4017/29775/2191/8828/3690/8829/1617 42/7424/5155/1012/7057/22846/6678/2822	15
BP	GO:00 61333	renal tubule morphogene sis	7/434	79/17913	0.0030 20624	0.046348 842	0.039 6356 3	342184/2246/1282/170690/6662/5311/55083	7
BP	GO:00 50770	regulation of axonogenes is	10/434	148/1791 3	0.0032 89688	0.049902 654	0.042 6747 04	1002/2335/8766/51330/8829/23499/2048/389 7/3984/51393	10
BP	GO:00 30201	heparan sulfate proteoglyca n metabolic process	4/434	26/17913	0.0033 28908	0.049902 654	0.042 6747 04	55959/29940/23213/2131	4
BP	GO:00 50919	negative chemotaxis	4/434	26/17913	0.0033 28908	0.049902 654	0.042 6747 04	5154/8828/3690/3685	4
BP	GO:00 61437	renal system vasculature developmen t	4/434	26/17913	0.0033 28908	0.049902 654	0.042 6747 04	8829/5155/5159/5311	4
BP	GO:00	kidney	4/434	26/17913	0.0033	0.049902	0.042	8829/5155/5159/5311	4

© 2020 Yu Y et al. *JAMA Network Open.*

	61440	vasculature development			28908	654	674704		
BP	GO:1903034	regulation of response to wounding	11/434	173/17913	0.003356589	0.049902654	0.042674704	135228/2191/5154/51330/5328/2152/5155/5592/7057/2149/5329	11
BP	GO:0030205	dermatan sulfate metabolic process	3/434	13/17913	0.003370733	0.049902654	0.042674704	29940/1462/633	3
BP	GO:1900025	negative regulation of substrate adhesion-dependent cell spreading	3/434	13/17913	0.003370733	0.049902654	0.042674704	4241/10631/23603	3
BP	GO:2001044	regulation of integrin-mediated signaling pathway	3/434	13/17913	0.003370733	0.049902654	0.042674704	2316/84695/7076	3
CC	GO:0062023	collagen-containing extracellular matrix	72/457	399/18678	1.09E-41	3.89E-39	3.09E-39	7045/8728/3956/4053/7041/4017/3918/4240/2335/7837/64175/1289/3371/9509/1284/7042/1307/115908/23213/1290/1294/80781/439921/1000/79883/1291/30008/308/1293/1295/1292/7076/3909/1462/165/1282/3910/1277/633/4054/2152/10631/1301/1300/2239/1281/1296/11117/5155/10082/3897/1012/5118/81794/1278/7057/81029/6678/25878/1508/7058/83872/22795/7052/4237/3339/5265/50509/284217/12/10085/1893	72
CC	GO:0031012	extracellular matrix	76/457	468/18678	1.20E-40	2.15E-38	1.71E-38	7045/8728/3956/2246/4053/7041/4017/3918/4240/2335/7837/64175/1289/3371/9509/1284/7042/1307/115908/23213/1290/1294/80781/4323/439921/1000/79883/1291/30008/308/1293/1295/1292/7076/3909/1462/165/1282/3910/1277/633/4054/2152/10631/1301/1300/223	76

© 2020 Yu Y et al. *JAMA Network Open.*

								9/1281/1296/11117/5155/10082/3897/1012/5118/81794/1278/7057/81029/6678/2822/25878/1508/7058/3730/83872/22795/7052/4237/3339/5265/50509/284217/12/10085/1893	
CC	GO:0005788	endoplasmic reticulum lumen	45/457	306/18678	2.21E-22	2.64E-20	2.10E-20	79642/3956/56034/84141/4241/4240/2335/340075/5154/493869/64175/1289/3371/56975/1284/1307/1290/60681/1294/80781/57333/1000/1291/1293/1295/1292/7076/1462/1282/1277/1301/283208/1300/1281/1296/5155/1278/7057/81029/1622/1435/51148/5329/5265/50509	45
CC	GO:0005925	focal adhesion	45/457	397/18678	8.15E-18	7.07E-16	5.63E-16	9260/87/2316/60312/3693/7041/9902/2191/8496/3371/2275/3690/23022/7431/5328/58504/4323/9448/3685/7791/3678/8829/1000/50507/4478/308/2535/5782/6237/143903/3897/1012/5159/11135/22801/91179/23603/3673/7052/824/5329/3984/3339/11261/29780	45
CC	GO:0005924	cell-substrate adherens junction	45/457	399/18678	9.90E-18	7.07E-16	5.63E-16	9260/87/2316/60312/3693/7041/9902/2191/8496/3371/2275/3690/23022/7431/5328/58504/4323/9448/3685/7791/3678/8829/1000/50507/4478/308/2535/5782/6237/143903/3897/1012/5159/11135/22801/91179/23603/3673/7052/824/5329/3984/3339/11261/29780	45
CC	GO:0005912	adherens junction	50/457	496/18678	1.57E-17	7.52E-16	5.99E-16	9260/87/342184/2316/60312/3693/7041/9902/2191/8496/3371/2275/3690/23022/143098/7431/5328/79983/58504/4323/9448/3685/7791/3678/8829/1000/50507/4478/308/2535/5782/6237/143903/3897/1012/5159/11135/22801/91179/23603/3673/7052/58538/824/5329/3984/3339/11261/23150/29780	50
CC	GO:0030055	cell-substrate junction	45/457	404/18678	1.60E-17	7.52E-16	5.99E-16	9260/87/2316/60312/3693/7041/9902/2191/8496/3371/2275/3690/23022/7431/5328/58504/4323/9448/3685/7791/3678/8829/1000/50507/4478/308/2535/5782/6237/143903/3897/1012/5159/11135/22801/91179/23603/3673/7052/824/5329/3984/3339/11261/29780	45

© 2020 Yu Y et al. *JAMA Network Open.*

CC	GO:0044420	extracellular matrix component	18/457	47/18678	1.69E-17	7.52E-16	5.99E-16	3918/1289/3371/1284/1290/1294/1295/3909/1282/1277/1301/1281/11117/81794/1278/4237/50509/284217	18
CC	GO:0005604	basement membrane	22/457	91/18678	3.18E-16	1.26E-14	1.00E-14	7045/4017/3918/2335/1289/3371/1284/1294/80781/30008/1295/7076/3909/1282/3910/1296/6678/7058/83872/22795/3339/284217	22
CC	GO:0005581	collagen trimer	20/457	86/18678	1.62E-14	5.77E-13	4.60E-13	1289/1284/1307/115908/1290/1294/80781/1291/1293/1295/1292/1282/1277/1301/1300/1281/1296/11117/1278/50509	20
CC	GO:0098644	complex of collagen trimers	11/457	18/18678	4.56E-14	1.48E-12	1.18E-12	1289/1284/1290/1294/1295/1282/1277/1301/1281/1278/50509	11
CC	GO:0005583	fibrillar collagen trimer	7/457	11/18678	1.52E-09	4.18E-08	3.32E-08	1289/1290/1277/1301/1281/1278/50509	7
CC	GO:0098643	banded collagen fibril	7/457	11/18678	1.52E-09	4.18E-08	3.32E-08	1289/1290/1277/1301/1281/1278/50509	7
CC	GO:0031252	cell leading edge	30/457	384/18678	2.44E-08	6.21E-07	4.95E-07	9260/26230/87/323/2054/2191/8572/91663/3690/23022/51330/7431/3685/3678/4651/1000/84168/1021/55704/23499/143903/54434/55785/5311/23603/7168/3984/23150/29780/23516	30
CC	GO:0008305	integrin complex	9/457	30/18678	2.63E-08	6.27E-07	4.99E-07	3693/3690/3685/3678/11117/3684/22801/3673/3696	9
CC	GO:0031091	platelet alpha granule	14/457	91/18678	4.33E-08	9.65E-07	7.69E-07	87/135228/2335/5154/7042/3690/7076/7424/5155/7057/6678/7058/5265/12	14
CC	GO:0098636	protein complex involved in cell adhesion	9/457	33/18678	6.65E-08	1.40E-06	1.11E-06	3693/3690/3685/3678/11117/3684/22801/3673/3696	9
CC	GO:0030027	lamellipodium	19/457	191/18678	2.47E-07	4.89E-06	3.90E-06	26230/323/2054/2191/8572/3690/23022/3685/4651/1000/84168/55704/54434/55785/5311/23603/3984/29780/23516	19

© 2020 Yu Y et al. *JAMA Network Open.*

CC	GO:0031093	platelet alpha granule lumen	11/457	67/18678	6.24E-07	1.17E-05	9.33E-06	87/2335/5154/7042/7076/7424/5155/7057/6678/5265/12	11
CC	GO:0001726	ruffle	17/457	168/18678	8.32E-07	1.49E-05	1.18E-05	9260/87/2191/91663/3690/23022/51330/3685/3678/4651/1021/23499/143903/55785/23603/7168/23150	17
CC	GO:0015629	actin cytoskeleton	26/457	432/18678	2.53E-05	0.00042968	0.000342071	84176/9260/87/342184/2316/60312/8572/91663/23022/79983/7791/4651/1000/50507/7169/5782/1627/800/541468/5311/23603/58538/824/7168/4919/11346	26
CC	GO:0042641	actomyosin	9/457	67/18678	3.53E-05	0.000572764	0.000455982	9260/87/8572/7791/50507/1627/541468/7168/4919	9
CC	GO:0098802	plasma membrane receptor complex	13/457	153/18678	0.00010174	0.001579179	0.001257197	3693/9180/3690/3685/3678/2892/11117/3684/22801/7097/3673/3696/658	13
CC	GO:0005938	cell cortex	16/457	229/18678	0.000173008	0.00257349	0.002048776	2246/2316/23187/3918/91663/4651/1000/6764/1627/800/2035/5311/23603/83872/824/26086	16
CC	GO:0030139	endocytic vesicle	18/457	289/18678	0.000290668	0.004150741	0.003304438	3693/6556/8877/8766/7431/5287/3685/7791/815/2892/84251/2535/84440/81029/6678/7097/11031/4688	18
CC	GO:0009897	external side of plasma membrane	14/457	199/18678	0.000404766	0.005263607	0.0041904	4240/3685/3678/84168/308/118429/2152/2239/1012/6338/3684/7057/3673/824	14
CC	GO:0001725	stress fiber	7/457	56/18678	0.000412832	0.005263607	0.0041904	9260/87/8572/7791/50507/7168/4919	7
CC	GO:0097517	contractile actin filament bundle	7/457	56/18678	0.000412832	0.005263607	0.0041904	9260/87/8572/7791/50507/7168/4919	7
CC	GO:0032432	actin filament bundle	7/457	59/18678	0.000570265	0.007020163	0.005588807	9260/87/8572/7791/50507/7168/4919	7

© 2020 Yu Y et al. *JAMA Network Open.*

CC	GO:0031527	filopodium membrane	4/457	18/18678	0.000824052	0.009806219	0.007806808	3690/3685/4651/84168	4
CC	GO:0005796	Golgi lumen	9/457	102/18678	0.000891163	0.010262751	0.008170258	5154/4323/1462/633/2239/5155/10082/81029/3339	9
CC	GO:0030667	secretory granule membrane	17/457	293/18678	0.000941276	0.010501109	0.008360016	135228/6556/3690/5328/199675/3685/23601/140885/2212/148229/27180/3684/6678/7097/11031/5329/11010	17
CC	GO:0044853	plasma membrane raft	9/457	106/18678	0.001172955	0.012689239	0.010102004	284119/112464/1000/161742/1012/3684/23603/2149/30846	9
CC	GO:0098797	plasma membrane protein complex	22/457	451/18678	0.001783966	0.018596599	0.0148049	3693/2121/6443/9180/3690/143098/3685/3678/1000/2892/84251/11117/355/3684/22801/23603/7097/3673/3696/4688/658/3339	22
CC	GO:0031258	lamellipodium membrane	4/457	22/18678	0.001823196	0.018596599	0.0148049	2191/3690/3685/84168	4
CC	GO:0005884	actin filament	7/457	74/18678	0.002199078	0.021807523	0.017361142	87/342184/2316/8572/23022/79983/5311	7
CC	GO:0043235	receptor complex	16/457	292/18678	0.002353603	0.022709092	0.018078888	3693/8828/9180/3690/3685/3678/8829/2892/11117/3684/22801/7097/3673/3696/4919/658	16
CC	GO:0030175	filopodium	8/457	99/18678	0.002965534	0.027860414	0.022179896	26230/3690/3685/4651/4478/84168/10123/57451	8
CC	GO:0005901	caveola	7/457	79/18678	0.00319234	0.029222185	0.023264013	284119/112464/161742/1012/23603/2149/30846	7
CC	GO:0030863	cortical cytoskeleton	7/457	81/18678	0.003673559	0.032786518	0.026101607	2316/91663/1000/1627/800/2035/824	7
CC	GO:0044448	cell cortex part	9/457	128/18678	0.00426105	0.036218926	0.0288341	2316/23187/91663/1000/1627/800/2035/5311/824	9

© 2020 Yu Y et al. *JAMA Network Open.*

							74		
CC	GO:0045335	phagocytic vesicle	9/457	128/18678	0.00426105	0.036218926	0.028834174	3693/6556/8766/7431/3685/7791/7097/11031/4688	9
CC	GO:0034774	secretory granule lumen	16/457	321/18678	0.005871633	0.048748207	0.038808833	87/2335/5154/7042/3417/7076/7424/126014/5155/7057/3101/6678/978/5265/12/1893	16
MF	GO:0005201	extracellular matrix structural constituent	47/426	158/16969	1.62E-37	9.04E-35	7.99E-35	7045/4053/3918/4240/2335/7837/1289/3371/1284/1307/115908/1290/1294/80781/1291/30008/1293/1295/1292/3909/1462/165/1282/3910/1277/633/10631/1301/1300/1281/1296/11117/5118/1278/7057/6678/25878/7058/3730/83872/22795/4237/3339/50509/284217/10085/1893	47
MF	GO:0030020	extracellular matrix structural constituent conferring tensile strength	18/426	41/16969	1.31E-18	3.66E-16	3.23E-16	1289/1284/1307/1290/1294/80781/1291/1293/1295/1292/1282/1277/1301/1300/1281/1296/1278/50509	18
MF	GO:0048407	platelet-derived growth factor binding	9/426	11/16969	1.92E-13	3.57E-11	3.16E-11	5154/1289/1291/1282/1277/1281/5155/1278/5159	9
MF	GO:0005518	collagen binding	16/426	61/16969	1.36E-12	1.90E-10	1.68E-10	7045/9902/2335/5154/64175/84168/165/5155/5118/22801/7057/6678/1508/3673/22795/50509	16
MF	GO:0019838	growth factor binding	20/426	121/16969	2.18E-11	2.44E-09	2.15E-09	4053/135228/5154/8828/7837/1289/9180/3690/3685/8829/1291/1282/1277/4054/1281/5155/94031/1278/5159/7057	20
MF	GO:0005178	integrin binding	17/426	113/16969	3.16E-09	2.95E-07	2.60E-07	7045/87/2246/2191/4240/2335/1289/1307/3690/4323/3685/3678/1281/11117/7057/3673/10085	17
MF	GO:0005161	platelet-derived growth	6/426	14/16969	6.12E-07	4.71E-05	4.16E-05	56034/5154/3690/3678/5155/5159	6

© 2020 Yu Y et al. *JAMA Network Open.*

		factor receptor binding							
MF	GO:0005539	glycosaminoglycan binding	19/426	199/16969	6.74E-07	4.71E-05	4.16E-05	2246/4053/3918/2335/8828/1289/8829/7130/1462/633/10631/1301/143903/5118/7057/7058/7097/3730/53834	19
MF	GO:0050840	extracellular matrix binding	9/426	43/16969	9.63E-07	5.98E-05	5.28E-05	7045/3956/3690/3685/633/1301/7057/3673/1893	9
MF	GO:0004222	metalloendopeptidase activity	12/426	89/16969	2.22E-06	0.000124214	0.000109701	8728/81792/649/2191/9509/140766/4323/5069/170690/11174/81794/8038	12
MF	GO:0003779	actin binding	25/426	361/16969	5.02E-06	0.000254983	0.00022519	87/342184/2316/60312/170954/51088/23022/79983/4651/91010/4478/84168/7169/1627/9645/55704/23499/800/2035/54434/22846/23603/7168/56062/11346	25
MF	GO:1901681	sulfur compound binding	18/426	221/16969	1.25E-05	0.000580846	0.000512978	2246/4053/3918/51363/2335/8828/1289/8829/10631/1301/5118/7057/1622/7058/3673/3730/53834/56922	18
MF	GO:0008201	heparin binding	14/426	148/16969	2.20E-05	0.000899119	0.000794062	2246/4053/3918/2335/8828/1289/8829/10631/1301/5118/7057/7058/3730/53834	14
MF	GO:0050839	cell adhesion molecule binding	27/426	444/16969	2.25E-05	0.000899119	0.000794062	7045/87/2246/2316/2191/4240/2335/8496/1289/1307/3690/143098/4323/3685/3678/4478/3417/1627/23499/800/1281/11117/1012/11135/7057/3673/10085	27
MF	GO:0043394	proteoglycan binding	7/426	36/16969	2.66E-05	0.000991248	0.000875427	2335/1289/3371/7057/1508/3673/50509	7
MF	GO:0008237	metallopeptidase activity	14/426	153/16969	3.20E-05	0.001118597	0.000987896	8728/81792/649/155185/2191/9509/140766/4323/5069/170690/11174/81794/22846/8038	14
MF	GO:0046332	SMAD binding	9/426	72/16969	7.64E-05	0.002512862	0.002219249	56937/8091/2316/7041/1290/1281/1278/90993/658	9
MF	GO:00	growth	13/426	147/1696	8.67E-	0.002692	0.002	3624/56034/649/2246/5154/7042/353500/707	13

© 2020 Yu Y et al. *JAMA Network Open.*

	08083	factor activity		9	05	254	37768	6/23529/3976/5155/6320/1435	
MF	GO:0004065	arylsulfatase activity	4/426	12/16969	0.000165197	0.004860258	0.004292366	79642/55959/340075/23213	4
MF	GO:0019955	cytokine binding	10/426	100/16969	0.000206781	0.005779541	0.005104236	135228/8828/7837/3690/3685/8829/9244/4054/7057/133396	10
MF	GO:0001540	amyloid-beta binding	8/426	71/16969	0.000394142	0.01049169	0.009265799	323/7042/2892/2048/3684/7097/3673/3339	8
MF	GO:0008484	sulfuric ester hydrolase activity	4/426	15/16969	0.000429075	0.010902412	0.009628531	79642/55959/340075/23213	4
MF	GO:0015026	coreceptor activity	6/426	40/16969	0.000449959	0.010935964	0.009658162	3690/3685/8829/2239/10082/4919	6
MF	GO:0002020	protease binding	10/426	121/16969	0.000949081	0.022105685	0.019522768	2191/2335/3690/3685/1277/2152/1281/1278/5265/1893	10
MF	GO:0017048	Rho GTPase binding	11/426	146/16969	0.001155961	0.025847293	0.022827192	26230/2316/9732/9639/91010/7204/64857/55785/23603/4688/55701	11
MF	GO:0050431	transforming growth factor beta binding	4/426	21/16969	0.00166984	0.035901561	0.03170668	135228/3685/4054/7057	4
MF	GO:0005172	vascular endothelial growth factor receptor binding	3/426	11/16969	0.002230796	0.046185735	0.04078921	3690/3678/7424	3
MF	GO:0004867	serine-type endopeptidase inhibitor	7/426	73/16969	0.002343002	0.046776365	0.04131082 8	135228/1294/1293/6695/3730/5265/12	7

© 2020 Yu Y et al. *JAMA Network Open*.

		activity							
MF	GO:0001618	virus receptor activity	7/426	74/16969	0.002533147	0.047200978	0.041685827	558/3693/3690/3685/23601/3678/3673	7
MF	GO:0104005	hijacked molecular function	7/426	74/16969	0.002533147	0.047200978	0.041685827	558/3693/3690/3685/23601/3678/3673	7

© 2020 Yu Y et al. *JAMA Network Open.*

eTable 5. Kyoto Encyclopedia of Genes and Genomes Pathway Enrichment Analysis of Immune-Active Class in IMvigor210 Trial

ID	Description	Gene ratio	Bg ratio	*P* value	*P* adjust	Q value	Gene ID	Count
hsa05322	Systemic lupus erythematosus	13/33	133/7916	2.02E-15	1.13E-13	7.02E-14	3012/8329/8331/8332/8339/8345/8340/8342/8968/8355/8357/8354/8368	13
hsa05034	Alcoholism	13/33	184/7916	1.45E-13	4.05E-12	2.51E-12	3012/8329/8331/8332/8339/8345/8340/8342/8968/8355/8357/8354/8368	13
hsa05203	Viral carcinogenesis	8/33	201/7916	1.21E-06	2.26E-05	1.40E-05	898/991/1029/8339/8345/8340/8342/8368	8
hsa04110	Cell cycle	5/33	124/7916	0.000145275	0.002033854	0.001261601	898/991/1029/1869/27127	5
hsa04218	Cellular senescence	5/33	160/7916	0.000475816	0.005329135	0.003305666	898/1029/1869/2305/4605	5
hsa02010	ABC transporters	3/33	45/7916	0.000831408	0.007759806	0.004813414	10349/23460/10350	3
hsa05166	Human T-cell leukemia virus 1 infection	5/33	219/7916	0.001952437	0.015619499	0.009688787	898/991/1029/1869/7015	5

© 2020 Yu Y et al. *JAMA Network Open.*

hsa04115	p53 signaling pathway	3/33	72/7916	0.003237083	0.022659583	0.014055756	898/1029/6241	3
hsa04217	Necroptosis	4/33	162/7916	0.004354863	0.027096926	0.016808244	3012/8329/8331/8332	4
hsa05202	Transcriptional misregulation in cancer	4/33	186/7916	0.007089377	0.039700511	0.024626257	8968/8355/8357/8354	4

© 2020 Yu Y et al. *JAMA Network Open.*

eTable 6. Kyoto Encyclopedia of Genes and Genomes Pathway Enrichment Analysis of Immune-Exclusion Class in IMvigor210 Trial

ID	Description	GeneRatio	BgRatio	*P* value	*P* adjust	qvalue	geneID	Count
hsa00140	Steroid hormone biosynthesis	15/219	60/7916	4.66E-11	1.20E-08	1.14E-08	1646/8644/1551/3294/412/6783/54658/54575/54659/54657/54579/54578/54577/54600/7364	15
hsa05204	Chemical carcinogenesis	16/219	82/7916	5.65E-10	6.49E-08	6.17E-08	1646/222/1551/2947/2948/4257/6822/54658/54575/54659/54657/54579/54578/54577/54600/7364	16
hsa00982	Drug metabolism - cytochrome P450	15/219	72/7916	7.55E-10	6.49E-08	6.17E-08	222/2330/2947/2948/4128/4257/54658/54575/54659/54657/54579/54578/54577/54600/7364	15
hsa00980	Metabolism of xenobiotics by cytochrome P450	14/219	76/7916	1.53E-08	9.87E-07	9.39E-07	222/2947/2948/4257/6822/54658/54575/54659/54657/54579/54578/54577/54600/7364	14
hsa00053	Ascorbate and aldarate metabolism	9/219	27/7916	2.45E-08	1.14E-06	1.08E-06	54658/54575/54659/54657/54579/54578/54577/54600/7364	9
hsa00830	Retinol metabolism	13/219	67/7916	2.64E-08	1.14E-06	1.08E-06	53630/1551/9249/195814/54658/54575/54659/54657/54579/54578/54577/54600/7364	13
hsa00040	Pentose and glucuronate interconversions	9/219	34/7916	2.32E-07	8.53E-06	8.11E-06	54658/54575/54659/54657/54579/54578/54577/54600/7364	9
hsa00983	Drug metabolism - other enzymes	12/219	79/7916	1.49E-06	4.65E-05	4.42E-05	2947/2948/4257/54658/54575/54659/54657/54579/54578/54577/54600/7364	12
hsa00860	Porphyrin and chlorophyll	9/219	42/7916	1.62E-06	4.65E-05	4.42E-05	54658/54575/54659/54657/54579/54578/54577/54600/7364	9

© 2020 Yu Y et al. *JAMA Network Open.*

	metabolism							
hsa00590	Arachidonic acid metabolism	8/219	63/7916	0.000315458	0.00813881	0.007737017	8644/240/4051/11283/2877/64600/255189/151056	8
hsa00601	Glycosphingolipid biosynthesis - lacto and neolacto series	5/219	27/7916	0.000759957	0.017743034	0.016867103	10331/2525/2528/10690/6484	5
hsa00480	Glutathione metabolism	7/219	56/7916	0.000825257	0.017743034	0.016867103	124975/2877/2947/2948/3417/4257/4953	7

© 2020 Yu Y et al. *JAMA Network Open.*

eTable 7. Kyoto Encyclopedia of Genes and Genomes Pathway Enrichment Analysis of Immune-Dysfunctional Class in IMvigor210 Trial

ID	Description	GeneRatio	BgRatio	*P* value	*P* adjust	qvalue	geneID	Count
hsa04640	Hematopoietic cell lineage	44/540	98/7916	4.49E-26	1.25E-23	9.88E-24	290/929/930/911/914/933/948/951/952/915/916/917/960/921/925/1378/1380/1436/1438/1441/2208/2209/2322/2811/3108/3109/3112/3113/3115/3118/3119/3122/3123/3125/3553/7850/3559/3569/3575/3675/3676/3684/102723407/931	44
hsa04145	Phagosome	48/540	152/7916	1.97E-20	2.54E-18	2.00E-18	718/929/30835/948/10332/64581/11151/1514/1536/2204/2209/2212/2213/9103/2214/2215/3105/3106/3107/3108/3109/3112/3113/3115/3118/3119/3122/3123/3125/3134/3684/3689/102723407/8685/4360/4481/653361/4688/4689/4973/6890/6891/7097/10333/7280/347733/10381/84617	48
hsa05150	Staphylococcus aureus infection	38/540	96/7916	2.73E-20	2.54E-18	2.00E-18	712/713/714/717/718/719/720/721/728/629/3426/2204/2209/2212/2213/9103/2214/2215/2357/2358/2359/3108/3109/3112/3113/3115/3118/3119/3122/3123/3125/3383/3683/3684/3689/102723407/6403/6404	38
hsa05323	Rheumatoid arthritis	37/540	93/7916	7.09E-20	4.95E-18	3.90E-18	54/6347/6364/6348/6349/6352/940/941/942/1493/1514/2919/2920/6374/3576/3108/3109/3112/3113/3115/3118/3119/3122/3123/3125/3383/3553/3569/3683/3689/3725/102723407/4050/7097/8600/10673/7422	37
hsa04514	Cell adhesion molecules (CAMs)	44/540	147/7916	8.32E-18	4.64E-16	3.66E-16	57863/914/933/29126/940/923/941/942/925/999/1493/3105/3106/3107/3108/3109/3112/3113/3115/3118/3119/3122/3123/3125/3134/3383/29851/3676/3680/3683/3684/3689/3696/3897/5818/4897/80380/5788/6401/6402/6403/6404/6614/7412	44
hsa05140	Leishmaniasis	30/540	76/7916	3.18E-16	1.48E-14	1.17E-14	718/1378/1536/1917/2209/2212/9103/2214/2215/3108/3109/3112/3113/3115/3118/3119/3122/3123/3125/3553/3676/3684/3689/3725/102723407/653361/4688/4689/5579/7097	30

© 2020 Yu Y et al. *JAMA Network Open.*

hsa05332	Graft-versus-host disease	22/540	41/7916	1.06E-15	4.21E-14	3.32E-14	940/941/942/355/3002/3105/3106/3107/3108/3109/3112/3113/3115/3118/3119/3122/3123/3125/3134/3553/3569/5551	22
hsa05330	Allograft rejection	21/540	38/7916	2.15E-15	7.51E-14	5.92E-14	940/941/942/355/3002/3105/3106/3107/3108/3109/3112/3113/3115/3118/3119/3122/3123/3125/3134/102723407/5551	21
hsa04060	Cytokine-cytokine receptor interaction	59/540	294/7916	1.68E-14	5.20E-13	4.10E-13	655/6357/6362/6363/6347/6364/6366/6348/6349/6351/9560/6352/729230/1233/1236/939/1436/1438/1439/1441/2919/3627/6373/10563/2920/6374/3576/4283/3577/7852/10663/355/3587/3594/3595/3603/3553/7850/3556/9173/53832/50615/11009/3559/3560/3561/133396/9235/90865/3569/3575/3977/4050/4804/9180/4982/23495/8600/10673	59
hsa05416	Viral myocarditis	25/540	60/7916	2.23E-14	6.23E-13	4.91E-13	940/941/942/1525/3105/3106/3107/3108/3109/3112/3113/3115/3118/3119/3122/3123/3125/3134/3383/3683/3689/284217/102723407/5551/5880	25
hsa04061	Viral protein interaction with cytokine and cytokine receptor	32/540	100/7916	3.56E-14	9.02E-13	7.11E-13	6357/6362/6363/6347/6364/6366/6348/6349/6351/9560/6352/729230/1233/1236/1436/2919/3627/6373/10563/2920/6374/3576/4283/3577/7852/3587/53832/11009/3559/3560/3561/3569	32
hsa04940	Type I diabetes mellitus	21/540	43/7916	5.69E-14	1.32E-12	1.04E-12	940/941/942/355/3002/3105/3106/3107/3108/3109/3112/3113/3115/3118/3119/3122/3123/3125/3134/3553/5551	21
hsa05320	Autoimmune thyroid disease	23/540	53/7916	8.65E-14	1.86E-12	1.46E-12	940/941/942/1493/355/3002/3105/3106/3107/3108/3109/3112/3113/3115/3118/3119/3122/3123/3125/3134/102723407/5551/7253	23
hsa04062	Chemokine signaling pathway	44/540	189/7916	2.03E-13	4.05E-12	3.19E-12	107/113/10000/6357/6362/6363/6347/6364/6366/6348/6349/6351/9560/6352/729230/1233/1236/2919/3627/6373/10563/2920/6374/3576/4283/3577/7852/10663/1794/9844/59345/3055/3702/3718/653361/5293/5294/23533/5330/5579/5880/10235/7409/7454	44
hsa0	Primary immunodeficienc	19/5	38/7	5.35E-13	9.96E-12	7.85E-12	100/695/930/915/916/973/925/4261/29851/3561/3575/3718/3932/102723407/5788/6890/6891/23495/7535	19

© 2020 Yu Y et al. *JAMA Network Open.*

5340	y	40	916					
hsa05152	Tuberculosis	40/540	179/7916	1.06E-11	1.86E-10	1.46E-10	10000/718/929/30835/972/4261/10332/64581/11151/1378/2207/2209/2212/2213/9103/2214/2215/3108/3109/3112/3113/3115/3118/3119/3122/3123/3125/3587/3553/3569/3684/3687/3689/102723407/4046/4360/64127/7096/7097/10333	40
hsa04380	Osteoclast differentiation	33/540	128/7916	1.20E-11	1.98E-10	1.56E-10	54/10000/695/1436/2209/2212/2213/9103/2214/2215/3553/3725/3932/3937/353514/79168/10859/10288/11006/10990/653361/4688/4689/5293/140885/10326/55423/9021/6688/4982/8600/54209/7305	33
hsa04672	Intestinal immune network for IgA production	20/540	49/7916	1.48E-11	2.29E-10	1.81E-10	940/941/942/7852/3108/3109/3112/3113/3115/3118/3119/3122/3123/3125/29851/3569/3676/102723407/23495/10673	20
hsa04612	Antigen processing and presentation	24/540	78/7916	1.52E-10	2.23E-09	1.76E-09	972/925/4261/1514/3105/3106/3107/3108/3109/3112/3113/3115/3118/3119/3122/3123/3125/3134/3304/10437/3822/8302/6890/6891	24
hsa04658	Th1 and Th2 cell differentiation	26/540	92/7916	2.13E-10	2.97E-09	2.34E-09	919/915/916/917/3108/3109/3112/3113/3115/3118/3119/3122/3123/3125/3594/3595/3559/3560/3561/3718/3725/3932/4094/5588/6775/7535	26
hsa04659	Th17 cell differentiation	28/540	107/7916	3.09E-10	4.10E-09	3.24E-09	919/915/916/917/3108/3109/3112/3113/3115/3118/3119/3122/3123/3125/3594/3553/3556/50615/3559/3560/3561/3569/3662/3718/3725/3932/5588/7535	28
hsa05169	Epstein-Barr virus infection	40/540	201/7916	4.62E-10	5.86E-09	4.62E-09	10000/695/894/930/919/915/916/917/960/1021/1380/3627/355/2208/3105/3106/3107/3108/3109/3112/3113/3115/3118/3119/3122/3123/3125/3134/3383/3569/3683/3718/3725/102723407/4939/5293/6890/6891/7097/7128	40
hsa05321	Inflammatory bowel disease (IBD)	21/540	65/7916	7.91E-10	9.59E-09	7.56E-09	3108/3109/3112/3113/3115/3118/3119/3122/3123/3125/3594/3595/3553/50615/3561/3569/3725/4094/64127/6775/7097	21
hs	Complement	23	79	1.25	1.46E-	1.15E-0	712/713/714/717/718/719/720/721/728/730/629/3426/1378/1380/2152/3684/3	2

© 2020 Yu Y et al. *JAMA Network Open.*

a04610	and coagulation cascades	/540	/7916	E-09	08	8	687/3689/5329/5265/710/7035/11326	3
hsa05322	Systemic lupus erythematosus	30/540	133/7916	3.47E-09	3.87E-08	3.05E-08	712/713/714/717/718/720/721/730/940/941/942/2209/2212/2214/2215/2903/2904/8356/8361/3108/3109/3112/3113/3115/3118/3119/3122/3123/3125/102723407	30
hsa05166	Human T-cell leukemia virus 1 infection	40/540	219/7916	6.42E-09	6.89E-08	5.43E-08	107/113/10000/894/915/916/917/9586/1958/1959/3105/3106/3107/3108/3109/3112/3113/3115/3118/3119/3122/3123/3125/3134/3383/7850/3559/3560/3561/3569/3683/3689/3718/3725/3932/4316/5293/6513/6688/7538	40
hsa04064	NF-kappa B signaling pathway	25/540	102/7916	1.21E-08	1.25E-07	9.82E-08	597/330/695/6357/6363/6366/6351/9560/929/2919/2920/3576/3383/3553/3932/102723407/4050/23643/5579/5588/7128/8600/10673/7412/7535	25
hsa04662	B cell receptor signaling pathway	22/540	82/7916	1.54E-08	1.54E-07	1.21E-07	10000/695/930/933/973/974/1380/2213/8519/3725/353514/79168/10859/10288/11006/10990/102723407/118788/5293/5579/5880/7409	22
hsa05146	Amoebiasis	24/540	102/7916	5.54E-08	5.33E-07	4.20E-07	107/384/929/911/1285/1286/2919/2920/3576/3553/7850/3569/3684/3689/284217/3909/3914/102723407/4583/5293/5330/5579/5272/7097	24
hsa05144	Malaria	16/540	49/7916	7.15E-08	6.65E-07	5.24E-07	2532/6347/948/1378/3576/2995/3383/3553/3569/3683/3689/3820/6401/6403/7097/7412	16
hsa04620	Toll-like receptor signaling pathway	24/540	104/7916	8.25E-08	7.43E-07	5.86E-07	10000/6348/6349/6351/9560/6352/929/941/942/3627/6373/3576/4283/3553/3569/3725/23643/5293/6696/7096/7097/10333/51284/51311	24
hsa05142	Chagas disease (American trypanosomiasis)	23/540	102/7916	2.41E-07	2.10E-06	1.66E-06	107/10000/712/713/714/718/6347/6348/6349/6352/919/915/916/917/3576/355/3553/3569/3725/5293/5330/7097/10333	23
hs	Asthma	12	31	3.83	3.23E-	2.55E-0	2207/3108/3109/3112/3113/3115/3118/3119/3122/3123/3125/102723407	1

© 2020 Yu Y et al. *JAMA Network Open.*

a05310		/540	/7916	E-07	06	6		2
hsa05162	Measles	27/540	138/7916	4.77E-07	3.92E-06	3.09E-06	10000/894/30835/940/915/916/917/1021/10332/355/2213/3304/3553/3559/3560/3561/3569/3718/3725/4478/4599/4939/5293/6504/7097/51284/7128	27
hsa05133	Pertussis	19/540	76/7916	4.98E-07	3.97E-06	3.13E-06	712/713/714/717/718/720/721/929/6374/3576/3553/3569/3394/3684/3689/3725/23643/114548/710	19
hsa05164	Influenza A	30/540	170/7916	1.13E-06	8.75E-06	6.90E-06	10000/6347/6352/1021/4261/3627/3576/355/3108/3109/3112/3113/3115/3118/3119/3122/3123/3125/3383/3553/90865/3569/4599/114548/4939/5293/5579/91543/9021/51284	30
hsa04668	TNF signaling pathway	23/540	112/7916	1.41E-06	1.06E-05	8.37E-06	10000/330/6347/6364/6352/9586/2919/3627/2920/6374/1906/355/3383/3553/3569/3725/4318/64127/5293/6401/9021/7128/7412	23
hsa04650	Natural killer cell mediated cytotoxicity	25/540	131/7916	2.06E-06	1.51E-05	1.19E-05	919/962/355/2207/2214/2215/3002/3105/3106/3107/3383/3683/3689/3822/3932/3937/102723407/5293/5551/5579/5880/4068/7305/7409/7535	25
hsa05145	Toxoplasmosis	22/540	112/7916	5.16E-06	3.69E-05	2.91E-05	10000/330/4261/3108/3109/3112/3113/3115/3118/3119/3122/3123/3125/3304/3587/284217/3909/3914/23643/5294/23533/7097	22
hsa04933	AGE-RAGE signaling pathway in diabetic complications	20/540	100/7916	1.04E-05	7.27E-05	5.73E-05	10000/6347/1285/1286/3576/1536/1906/1958/2152/3383/3553/3569/3725/5293/5330/113026/5579/6401/7412/7422	20
hsa04660	T cell receptor signaling pathway	20/540	104/7916	1.92E-05	0.000130578	0.000102965	10000/919/940/915/916/917/925/1493/29851/3702/3725/3932/3937/5063/5293/5588/5788/10125/7409/7535	20

© 2020 Yu Y et al. *JAMA Network Open.*

hsa05202	Transcriptional misregulation in cancer	29/540	186/7916	2.10E-05	0.000139522	0.000110018	8938/597/330/894/929/942/5218/1436/3576/2118/2209/2322/3002/8356/8091/7850/3560/3569/3684/102723407/4094/4318/4804/8013/26471/5079/860/6688/7102	29
hsa04151	PI3K-Akt signaling pathway	45/540	354/7916	3.08E-05	0.000200084	0.000157772	10000/374/894/930/1021/1285/1286/131873/1297/9586/1436/1441/2069/2261/2322/59345/3481/3559/3560/3561/3569/3575/3675/3676/3680/3691/3696/3718/284217/3909/3914/102723407/23566/4804/3164/9180/118788/5293/5294/23533/5649/6446/6696/7097/7422	45
hsa04666	Fc gamma R-mediated phagocytosis	18/540	93/7916	4.55E-05	0.00028867	0.000227625	10000/274/1794/2209/2212/2213/2214/2215/3055/102723407/653361/5293/8395/5579/5788/5880/7409/7454	18
hsa05135	Yersinia infection	21/540	120/7916	5.25E-05	0.000325291	0.000256502	10000/64283/6347/3576/2533/3553/3569/3676/3725/3932/3937/4210/114548/5293/8395/5880/27330/7409/7454/7456/7535	21
hsa05134	Legionellosis	13/540	56/7916	7.43E-05	0.000448769	0.000353868	718/929/1378/2919/2920/3576/1917/3304/3553/3569/3684/3689/7097	13
hsa04512	ECM-receptor interaction	17/540	88/7916	7.56E-05	0.000448769	0.000353868	948/960/1285/1286/131873/1297/2811/3675/3676/3680/3691/3696/284217/3909/3914/5649/6696	17
hsa05143	African trypanosomiasis	10/540	37/7916	0.0001301	0.000756207	0.000596292	355/3383/3620/3553/3569/102723407/5330/5579/6401/7412	10
hsa04670	Leukocyte transendothelial migration	19/540	112/7916	0.000181033	0.001030779	0.000812801	7852/1536/3383/3676/3683/3684/3689/3702/4318/4478/653361/4688/4689/5293/5579/5880/7294/7409/7412	19
hsa04630	JAK-STAT signaling pathway	23/540	162/7916	0.000598507	0.003339666	0.002633429	10000/316/894/1438/1439/1441/3587/3594/3595/53832/50615/11009/3559/3560/3561/3569/3575/3718/3977/9180/5293/9021/6775	23

© 2020 Yu Y et al. *JAMA Network Open.*

hsa05163	Human cytomegalovirus infection	29/540	225/7916	0.000638238	0.003491535	0.002753181	107/113/10000/6347/6348/6349/6351/9560/6352/1021/9586/3576/7852/355/59345/3105/3106/3107/3134/3587/3553/3569/5293/5330/5579/5880/6890/6891/7422	29
hsa05167	Kaposi sarcoma-associated herpesvirus infection	25/540	186/7916	0.000805378	0.004321165	0.00340737	10000/718/1233/942/1021/9976/2919/2920/3576/355/59345/3055/3105/3106/3107/3134/3383/3569/3725/5293/5294/23533/1827/7422/7538	25
hsa04611	Platelet activation	18/540	124/7916	0.001773475	0.009335842	0.007361596	107/113/10000/54518/695/2207/2212/83706/2811/3937/5293/5294/23533/5330/5742/10125/10235/6916	18
hsa05020	Prion diseases	8/540	35/7916	0.00202325	0.010453457	0.00824287	712/713/714/730/6352/1958/3553/3569	8
hsa04510	Focal adhesion	25/540	199/7916	0.002120094	0.010754657	0.008480375	10000/330/894/1285/1286/131873/1297/3675/3676/3680/3691/3696/3725/284217/3909/3914/5063/64098/5293/5579/5880/5649/6696/7409/7422	25
hsa05235	PD-L1 expression and PD-1 checkpoint pathway in cancer	14/540	89/7916	0.002622096	0.013063656	0.010301091	10000/919/29126/940/915/916/917/3725/3932/5293/5588/10125/7097/7535	14
hsa04621	NOD-like receptor signaling pathway	23/540	181/7916	0.00271256	0.013081662	0.010315289	9447/330/6347/6352/2919/2920/3576/1536/2633/115361/115362/3428/3553/3569/3725/4210/22861/114548/64127/4939/5027/5330/7128	23
hsa05418	Fluid shear stress and atherosclerosis	19/540	139/7916	0.002719485	0.013081662	0.010315289	10000/6347/1514/1843/1906/2817/3162/3383/3553/7850/3725/4318/653361/4688/5293/5880/6401/7412/7422	19
hsa040	Rap1 signaling pathway	24/540	210/79	0.00855402	0.040450392	0.031896366	107/113/10000/54518/999/1436/2261/2357/2533/2903/2904/3683/3684/3689/3937/23566/4804/5293/5330/5579/5880/10235/7409/7422	24

© 2020 Yu Y et al. *JAMA Network Open.*

15			16	6				

© 2020 Yu Y et al. *JAMA Network Open*.

eTable 8. Kyoto Encyclopedia of Genes and Genomes Pathway Enrichment Analysis of Immune-Desert Class in IMvigor210 Trial

ID	Description	GeneRatio	BgRatio	*P* value	*P* adjust	qvalue	geneID	Count
hsa04512	ECM-receptor interaction	16/183	88/7916	1.17E-10	2.48E-08	2.24E-08	1277/1278/1291/1292/1293/1311/2335/22801/3678/3690/255743/7057/7058/7060/3371/63923	16
hsa04510	Focal adhesion	22/183	199/7916	8.39E-10	8.90E-08	8.04E-08	857/1277/1278/1291/1292/1293/1311/2316/2335/3082/22801/3678/3690/10398/56034/5156/5159/7057/7058/7060/3371/63923	22
hsa04974	Protein digestion and absorption	14/183	95/7916	3.02E-08	2.13E-06	1.93E-06	1300/1301/1303/1306/1277/1278/255631/1281/1289/1290/50509/1291/1292/1293	14
hsa05205	Proteoglycans in cancer	19/183	204/7916	2.07E-07	1.10E-05	9.92E-06	857/1277/1278/1634/2260/2316/2335/3082/3678/3690/4060/4313/5328/6383/7042/7057/7078/7291/7472	19
hsa04151	PI3K-Akt signaling pathway	23/183	354/7916	6.28E-06	0.000266071	0.000240442	1277/1278/1291/1292/1293/1311/90993/2260/2335/2690/3082/22801/3678/3690/4915/56034/5156/5159/7057/7058/7060/3371/63923	23
hsa05144	Malaria	8/183	49/7916	1.40E-05	0.00049589	0.00048123	1311/3082/6383/7042/7043/7057/7058/7060	8
hsa05165	Human papillomavirus infection	21/183	330/7916	2.24E-05	0.000679143	0.000613724	8313/1277/1278/1291/1292/1293/1311/90993/2335/22801/3678/3690/5159/5743/83439/7057/7058/7060/3371/63923/7472	21
hsa04933	AGE-RAGE signaling pathway in	10/183	100/7916	9.74E-05	0.002581782	0.00233309	185/1277/1278/1281/2335/4313/50507/5054/7042/7043	10

© 2020 Yu Y et al. *JAMA Network Open.*

	diabetic complic ations							
hsa0 5410	Hypertro phic cardiom yopathy (HCM)	9/183	90/7 916	0.0002 18311	0.0051 42449	0.0046 47099	781/22801/3678/3690/6444/7042/7043/7168/7169	9
hsa0 4350	TGF-bet a signalin g pathway	9/183	94/7 916	0.0003 03759	0.0064 39686	0.0058 19379	658/1634/2200/2331/26585/3624/7042/7043/7057	9
hsa0 5414	Dilated cardiom yopathy (DCM)	9/183	96/7 916	0.0003 5589	0.0066 66458	0.0060 24307	781/22801/3678/3690/6444/7042/7043/7168/7169	9
hsa0 5412	Arrhyth mogenic right ventricul ar cardiom yopathy (ARVC)	8/183	77/7 916	0.0003 77347	0.0066 66458	0.0060 24307	88/781/1000/22801/3678/3690/6444/83439	8
hsa0 4610	Comple ment and coagulat ion cascade s	8/183	79/7 916	0.0004 4984	0.0073 35859	0.0066 29228	715/716/3075/2162/2151/5328/5627/5054	8
hsa0 4310	Wnt signalin g	11/18 3	160/ 7916	0.0011 6434	0.0176 31442	0.0159 3308	8313/2239/85409/144165/4919/4920/5176/6423/6424/83439/7472	11

© 2020 Yu Y et al. *JAMA Network Open.*

	pathway							
hsa05215	Prostate cancer	8/183	97/7916	0.001751662	0.024756827	0.022372108	90993/2260/4314/56034/5156/5159/5328/83439	8
hsa05206	MicroRNAs in cancer	16/183	310/7916	0.002138318	0.028332717	0.025603548	1545/8660/3678/3690/5156/5159/5328/5743/7042/7057/7078/3371/63923/7168/7431/23414	16
hsa04145	Phagosome	10/183	152/7916	0.002683943	0.033470345	0.03024629	715/81035/1311/3678/3690/9902/7057/7058/7060/7846	10
hsa04390	Hippo signaling pathway	10/183	154/7916	0.00295181	0.034765759	0.031416922	8313/658/122786/2736/5054/6591/83439/7042/7043/7472	10
hsa04540	Gap junction	7/183	88/7916	0.00412983	0.04608021	0.0416415	2982/3357/56034/5156/5159/5592/7846	7

© 2020 Yu Y et al. *JAMA Network Open.*

eTable 9. Gene Ontology Pathway Enrichment Analysis of Immune-Active Class in IMvigor210 Trial

ONTOLOGY	ID	Description	GeneRatio	BgRatio	*P* value	*P* adjust	qvalue	geneID	Count
BP	GO:0031497	chromatin assembly	11/44	153/17913	7.39E-14	6.30E-11	5.00E-11	1029/3009/8339/8345/8340/8342/8968/8355/8357/8354/8368	11
BP	GO:0006333	chromatin assembly or disassembly	11/44	178/17913	3.94E-13	1.68E-10	1.33E-10	1029/3009/8339/8345/8340/8342/8968/8355/8357/8354/8368	11
BP	GO:0006334	nucleosome assembly	10/44	135/17913	8.42E-13	2.17E-10	1.72E-10	3009/8339/8345/8340/8342/8968/8355/8357/8354/8368	10
BP	GO:0006323	DNA packaging	11/44	194/17913	1.02E-12	2.17E-10	1.72E-10	1029/3009/8339/8345/8340/8342/8968/8355/8357/8354/8368	11
BP	GO:0034728	nucleosome organization	10/44	165/17913	6.33E-12	1.08E-09	8.57E-10	3009/8339/8345/8340/8342/8968/8355/8357/8354/8368	10
BP	GO:0071103	DNA conformation change	11/44	279/17913	5.23E-11	7.43E-09	5.90E-09	1029/3009/8339/8345/8340/8342/8968/8355/8357/8354/8368	11
BP	GO:0065004	protein-DNA complex assembly	10/44	210/17913	6.94E-11	8.45E-09	6.71E-09	3009/8339/8345/8340/8342/8968/8355/8357/8354/8368	10
BP	GO:0071824	protein-DNA complex subunit organization	10/44	240/17913	2.57E-10	2.74E-08	2.18E-08	3009/8339/8345/8340/8342/8968/8355/8357/8354/8368	10
BP	GO:0051290	protein heterotetramerization	6/44	53/17913	3.24E-09	3.08E-07	2.44E-07	8968/8355/8357/8354/8368/6241	6
BP	GO:0006335	DNA replication-dependent nucleosome assembly	5/44	32/17913	1.36E-08	1.05E-06	8.34E-07	8968/8355/8357/8354/8368	5
BP	GO:0034723	DNA replication-dependent nucleosome	5/44	32/17913	1.36E-08	1.05E-06	8.34E-07	8968/8355/8357/8354/8368	5

© 2020 Yu Y et al. *JAMA Network Open.*

		organization							
BP	GO:0000183	chromatin silencing at rDNA	5/44	39/17913	3.83E-08	2.72E-06	2.16E-06	8968/8355/8357/8354/8368	5
BP	GO:0032200	telomere organization	7/44	167/17913	1.55E-07	1.02E-05	8.05E-06	898/8968/8355/8357/8354/8368/7015	7
BP	GO:0060147	regulation of posttranscriptional gene silencing	6/44	117/17913	3.93E-07	2.24E-05	1.78E-05	8968/8355/8357/8354/8368/7015	6
BP	GO:0060966	regulation of gene silencing by RNA	6/44	117/17913	3.93E-07	2.24E-05	1.78E-05	8968/8355/8357/8354/8368/7015	6
BP	GO:0051291	protein heterooligomerization	6/44	128/17913	6.69E-07	3.56E-05	2.83E-05	8968/8355/8357/8354/8368/6241	6
BP	GO:1901990	regulation of mitotic cell cycle phase transition	9/44	428/17913	7.88E-07	3.92E-05	3.11E-05	898/991/1029/1869/7015/90381/22974/9319/11065	9
BP	GO:0038111	interleukin-7-mediated signaling pathway	4/44	30/17913	8.28E-07	3.92E-05	3.11E-05	8968/8355/8357/8354	4
BP	GO:0060968	regulation of gene silencing	6/44	139/17913	1.09E-06	4.87E-05	3.87E-05	8968/8355/8357/8354/8368/7015	6
BP	GO:0045652	regulation of megakaryocyte differentiation	5/44	79/17913	1.39E-06	5.94E-05	4.71E-05	8968/8355/8357/8354/8368	5
BP	GO:1901987	regulation of cell cycle phase	9/44	464/17913	1.54E-06	6.25E-05	4.96E-05	898/991/1029/1869/7015/90381/22974/9319/11065	9

© 2020 Yu Y et al. *JAMA Network Open.*

		transition							
BP	GO:0006342	chromatin silencing	5/44	83/17913	1.78E-06	6.91E-05	5.48E-05	8968/8355/8357/8354/8368	5
BP	GO:0098760	response to interleukin-7	4/44	41/17913	3.00E-06	0.000104104	8.26E-05	8968/8355/8357/8354	4
BP	GO:0098761	cellular response to interleukin-7	4/44	41/17913	3.00E-06	0.000104104	8.26E-05	8968/8355/8357/8354	4
BP	GO:0051262	protein tetramerization	6/44	166/17913	3.05E-06	0.000104104	8.26E-05	8968/8355/8357/8354/8368/6241	6
BP	GO:0030219	megakaryocyte differentiation	5/44	96/17913	3.66E-06	0.000120179	9.54E-05	8968/8355/8357/8354/8368	5
BP	GO:0045814	negative regulation of gene expression, epigenetic	5/44	101/17913	4.70E-06	0.000148613	0.000117922	8968/8355/8357/8354/8368	5
BP	GO:0060964	regulation of gene silencing by miRNA	5/44	113/17913	8.16E-06	0.000248477	0.000197163	8968/8355/8357/8354/8368	5
BP	GO:0000082	G1/S transition of mitotic cell cycle	6/44	269/17913	4.74E-05	0.001373878	0.0010109015	898/1029/1869/128239/6241/7015	6
BP	GO:0000083	regulation of transcription involved in G1/S transition of mitotic cell cycle	3/44	29/17913	4.83E-05	0.001373878	0.0010109015	898/1869/6241	3
BP	GO:00	cell cycle	6/44	288/1	6.93E-0	0.0018	0.0014	898/1029/1869/128239/6241/7015	6

	44843	G1/S phase transition		7913	5	48996	67149		
BP	GO:0051225	spindle assembly	4/44	90/17913	6.94E-05	0.001848996	0.001467149	259266/991/4605/22974	4
BP	GO:0010948	negative regulation of cell cycle process	6/44	344/17913	0.000183135	0.004733759	0.003756163	991/1029/1869/6241/90381/9319	6
BP	GO:0000280	nuclear division	6/44	357/17913	0.00022378	0.005614243	0.004454814	259266/991/4605/22974/9319/11065	6
BP	GO:0006563	L-serine metabolic process	2/44	10/17913	0.000262053	0.006306525	0.005004129	26227/29968	2
BP	GO:1901991	negative regulation of mitotic cell cycle phase transition	5/44	235/17913	0.000266477	0.006306525	0.005004129	991/1029/1869/90381/9319	5
BP	GO:0140014	mitotic nuclear division	5/44	237/17913	0.000277105	0.006306525	0.005004129	991/4605/22974/9319/11065	5
BP	GO:0090307	mitotic spindle assembly	3/44	52/17913	0.000280947	0.006306525	0.005004129	991/4605/22974	3
BP	GO:0045637	regulation of myeloid cell differentiation	5/44	250/17913	0.000354267	0.007748449	0.006148273	8968/8355/8357/8354/8368	5
BP	GO:1901988	negative regulation of cell cycle phase transition	5/44	254/17913	0.000381001	0.007811607	0.006198387	991/1029/1869/90381/9319	5
BP	GO:1904668	positive regulation of ubiquitin	2/44	12/17913	0.000383145	0.007811607	0.006198387	991/11065	2

© 2020 Yu Y et al. *JAMA Network Open.*

		protein ligase activity							
BP	GO:0048285	organelle fission	6/44	395/17913	0.000384628	0.007811607	0.006198387	259266/991/4605/22974/9319/11065	6
BP	GO:0090342	regulation of cell aging	3/44	59/17913	0.000408349	0.008100505	0.006427623	1029/2305/7015	3
BP	GO:0007051	spindle organization	4/44	147/17913	0.000457916	0.008680051	0.006887484	259266/991/4605/22974	4
BP	GO:0007093	mitotic cell cycle checkpoint	4/44	147/17913	0.000457916	0.008680051	0.006887484	991/1869/90381/9319	4
BP	GO:0090399	replicative senescence	2/44	14/17913	0.000526628	0.009765506	0.007748776	1029/7015	2
BP	GO:0009070	serine family amino acid biosynthetic process	2/44	15/17913	0.000606699	0.011010941	0.008737009	26227/29968	2
BP	GO:0045930	negative regulation of mitotic cell cycle	5/44	308/17913	0.000912087	0.015996534	0.012692999	991/1029/1869/90381/9319	5
BP	GO:2000045	regulation of G1/S transition of mitotic cell cycle	4/44	177/17913	0.00091891	0.015996534	0.012692999	898/1029/1869/7015	4
BP	GO:1901992	positive regulation of mitotic cell cycle phase transition	3/44	82/17913	0.001069508	0.018245804	0.014477759	898/7015/11065	3
BP	GO:0048477	oogenesis	3/44	84/17913	0.001146786	0.019180558	0.015219472	259266/9319/51087	3
BP	GO:1902806	regulation of cell cycle	4/44	195/17913	0.001315211	0.021248324	0.016860211	898/1029/1869/7015	4

		G1/S phase transition							
BP	GO:19 04666	regulation of ubiquitin protein ligase activity	2/44	22/17 913	0.0013 20236	0.0212 48324	0.0168 60211	991/11065	2
BP	GO:00 00075	cell cycle checkpoint	4/44	197/1 7913	0.0013 65574	0.0215 71012	0.0171 16259	991/1869/90381/9319	4
BP	GO:00 51321	meiotic cell cycle	4/44	203/1 7913	0.0015 24752	0.0236 47525	0.0187 63939	259266/991/27127/9319	4
BP	GO:00 70199	establishmen t of protein localization to chromosome	2/44	24/17 913	0.0015 72512	0.0239 5273	0.0190 06115	3009/7015	2
BP	GO:00 07052	mitotic spindle organization	3/44	95/17 913	0.0016 34887	0.0244 65946	0.0194 13344	991/4605/22974	3
BP	GO:19 01989	positive regulation of cell cycle phase transition	3/44	97/17 913	0.0017 35552	0.0255 24577	0.0202 53351	898/7015/11065	3
BP	GO:00 51443	positive regulation of ubiquitin-prot ein transferase activity	2/44	28/17 913	0.0021 40271	0.0304 27522	0.0241 43761	991/11065	2
BP	GO:00 90344	negative regulation of cell aging	2/44	28/17 913	0.0021 40271	0.0304 27522	0.0241 43761	2305/7015	2
BP	GO:00 06270	DNA replication initiation	2/44	30/17 913	0.0024 55347	0.0322 21712	0.0255 67423	898/90381	2
BP	GO:00	mitotic	2/44	30/17	0.0024	0.0322	0.0255	991/9319	2

© 2020 Yu Y et al. *JAMA Network Open.*

	07094	spindle assembly checkpoint		913	55347	21712	67423		
BP	GO:00 31577	spindle checkpoint	2/44	30/17 913	0.0024 55347	0.0322 21712	0.0255 67423	991/9319	2
BP	GO:00 71173	spindle assembly checkpoint	2/44	30/17 913	0.0024 55347	0.0322 21712	0.0255 67423	991/9319	2
BP	GO:00 71174	mitotic spindle checkpoint	2/44	30/17 913	0.0024 55347	0.0322 21712	0.0255 67423	991/9319	2
BP	GO:00 00086	G2/M transition of mitotic cell cycle	4/44	238/1 7913	0.0027 19188	0.0350 10038	0.0277 79915	2305/9833/90381/22974	4
BP	GO:00 45841	negative regulation of mitotic metaphase/a naphase transition	2/44	32/17 913	0.0027 90953	0.0350 10038	0.0277 79915	991/9319	2
BP	GO:20 00816	negative regulation of mitotic sister chromatid separation	2/44	32/17 913	0.0027 90953	0.0350 10038	0.0277 79915	991/9319	2
BP	GO:00 07569	cell aging	3/44	116/1 7913	0.0028 88414	0.0351 43495	0.0278 85812	1029/2305/7015	3
BP	GO:19 02850	microtubule cytoskeleton organization involved in mitosis	3/44	116/1 7913	0.0028 88414	0.0351 43495	0.0278 85812	991/4605/22974	3
BP	GO:19 02100	negative regulation of metaphase/a	2/44	33/17 913	0.0029 66391	0.0351 43495	0.0278 85812	991/9319	2

© 2020 Yu Y et al. *JAMA Network Open.*

		naphase transition of cell cycle							
BP	GO:19 05819	negative regulation of chromosome separation	2/44	33/17 913	0.0029 66391	0.0351 43495	0.0278 85812	991/9319	2
BP	GO:00 30099	myeloid cell differentiatio n	5/44	411/1 7913	0.0032 29005	0.0377 30702	0.0299 3872	8968/8355/8357/8354/8368	5
BP	GO:00 33048	negative regulation of mitotic sister chromatid segregation	2/44	35/17 913	0.0033 32416	0.0384 12849	0.0304 79992	991/9319	2
BP	GO:00 60251	regulation of glial cell proliferation	2/44	36/17 913	0.0035 22953	0.0400 67716	0.0317 93103	1869/7015	2
BP	GO:00 44839	cell cycle G2/M phase transition	4/44	257/1 7913	0.0035 81324	0.0401 95649	0.0318 94616	2305/9833/90381/22974	4
BP	GO:00 33046	negative regulation of sister chromatid segregation	2/44	37/17 913	0.0037 18473	0.0406 6484	0.0322 66912	991/9319	2
BP	GO:20 00648	positive regulation of stem cell proliferation	2/44	37/17 913	0.0037 18473	0.0406 6484	0.0322 66912	259266/7015	2
BP	GO:00 51985	negative regulation of chromosome segregation	2/44	38/17 913	0.0039 18952	0.0423 14763	0.0335 761	991/9319	2
BP	GO:00 07292	female gamete	3/44	132/1 7913	0.0041 5483	0.0443 0087	0.0351 52045	259266/9319/51087	3

© 2020 Yu Y et al. *JAMA Network Open.*

		generation							
BP	GO:0051445	regulation of meiotic cell cycle	2/44	41/17913	0.0045499	0.046808582	0.037141875	259266/991	2
BP	GO:0070317	negative regulation of G0 to G1 transition	2/44	41/17913	0.0045499	0.046808582	0.037141875	1869/6241	2
BP	GO:0007059	chromosome segregation	4/44	275/17913	0.004554645	0.046808582	0.037141875	991/83540/27127/9319	4
BP	GO:0009069	serine family amino acid metabolic process	2/44	42/17913	0.004769972	0.048437932	0.038434738	26227/29968	2
CC	GO:0000786	nucleosome	13/44	106/18678	1.33E-19	1.25E-17	9.37E-18	3009/3012/8329/8332/8339/8345/8340/8342/8968/8355/8357/8354/8368	13
CC	GO:0044815	DNA packaging complex	13/44	113/18678	3.17E-19	1.49E-17	1.12E-17	3009/3012/8329/8332/8339/8345/8340/8342/8968/8355/8357/8354/8368	13
CC	GO:0032993	protein-DNA complex	14/44	187/18678	5.49E-18	1.72E-16	1.29E-16	3009/3012/8329/8332/8339/8345/8340/8342/8968/8355/8357/8354/8368/7015	14
CC	GO:0000785	chromatin	16/44	500/18678	1.15E-14	2.69E-13	2.02E-13	84733/1029/1869/3009/3012/8329/8332/8339/8345/8340/8342/8968/8355/8357/8354/8368	16
CC	GO:0000788	nuclear nucleosome	5/44	38/18678	2.72E-08	5.11E-07	3.83E-07	8968/8355/8357/8354/8368	5
CC	GO:0044454	nuclear chromosome part	9/44	469/18678	1.19E-06	1.87E-05	1.40E-05	1869/3009/8968/8355/8357/8354/8368/27127/7015	9
CC	GO:0000790	nuclear chromatin	7/44	309/18678	7.18E-06	9.65E-05	7.24E-05	1869/3009/8968/8355/8357/8354/8368	7
CC	GO:0000152	nuclear ubiquitin ligase complex	3/44	38/18678	9.71E-05	0.001141309	0.000856301	84733/991/11065	3
CC	GO:0000792	heterochromatin	3/44	75/18678	0.00073166	0.007641782	0.005733476	84733/1029/3009	3

© 2020 Yu Y et al. *JAMA Network Open.*

CC	GO:0005680	anaphase-promoting complex	2/44	21/18678	0.001106978	0.010405592	0.007807107	991/11065	2
CC	GO:0000922	spindle pole	3/44	152/18678	0.005481824	0.046844681	0.035146625	259266/991/22974	3
MF	GO:0046982	protein heterodimerization activity	12/44	497/16969	3.14E-09	2.57E-07	2.31E-07	3012/8329/8332/8339/8345/8340/8342/8968/8355/8357/8354/8368	12

© 2020 Yu Y et al. *JAMA Network Open.*

eTable 10. Gene Ontology Pathway Enrichment Analysis of Immune-Exclusion Class in IMvigor210 Trial

Ontology	ID	Description	Gene ratio	Bg ratio	*P* value	*P* adjust	Q value	Gene ID	Count
BP	GO:0008544	epidermis development	42/468	452/17913	9.99E-13	2.17E-09	2.02E-09	8644/9620/1515/1825/1830/1999/2125/645027/79152/94234/51083/29841/57822/3713/26085/3859/3875/3880/54474/3852/3853/3854/286887/3855/3887/440050/57830/3846/55283/4488/4610/5080/5046/5493/6469/80736/6700/6703/6707/412/7051/79755	42
BP	GO:0070268	cornification	21/468	112/17913	1.17E-12	2.17E-09	2.02E-09	1825/1830/2125/3713/26085/3859/3875/3880/54474/3852/3853/3854/286887/3855/3887/5046/5493/6700/6703/6707/7051	21
BP	GO:0042445	hormone metabolic process	28/468	215/17913	2.51E-12	3.10E-09	2.88E-09	1646/8644/53630/650/84649/10202/9249/53905/50506/90527/3169/51083/2625/51301/3294/389434/5046/151056/195814/6469/80736/60482/6783/54658/54659/54577/54600/7364	28
BP	GO:0009913	epidermal cell differentiation	33/468	346/17913	1.50E-10	1.40E-07	1.30E-07	8644/1515/1825/1830/2125/79152/29841/3713/26085/3859/3875/3880/54474/3852/3853/3854/286887/3855/3887/440050/57830/3846/55283/4488/4610/5080/5046/5493/80736/6700/6703/6707/7051	33
BP	GO:0043588	skin development	36/468	412/17913	2.52E-10	1.87E-07	1.74E-07	8644/9620/1364/1515/1825/1830/2125/79152/94234/51083/29841/57822/3713/26085/3859/3875/3880/54474/3852/3853/3854/286887/3855/3887/440050/57830/3846/4488/5080/5046/5493/6469/6700/6703/6707/7051	36
BP	GO:003021	keratinocyte different	29/4	298/179	1.26E-09	7.79E-07	7.25E-07	8644/1515/1825/1830/2125/29841/3713/26085/3859/3875/3880/54474/3852/3853/3854/286887/3855/3887/440050/57830/3846/4488/5080/5046/5493/6700/6703/6707/7051	29

© 2020 Yu Y et al. *JAMA Network Open.*

	6	iation	68	13					
BP	GO:1901615	organic hydroxy compound metabolic process	38/468	500/17913	4.11E-09	2.18E-06	2.02E-06	18/340485/1646/8644/222/8542/5205/53630/650/1050/1591/66002/4051/84649/9249/1812/53905/50506/90527/2625/51301/3158/8821/389434/3990/4128/114880/255738/151056/5333/51196/23007/9651/195814/11001/6653/6783/6822	38
BP	GO:0031424	keratinization	24/468	224/17913	5.22E-09	2.42E-06	2.25E-06	1825/1830/2125/3713/26085/3859/3875/3880/54474/3852/3853/3854/286887/3855/3887/440050/57830/3846/5046/5493/6700/6703/6707/7051	24
BP	GO:0052695	cellular glucuronidation	8/468	18/17913	7.11E-09	2.93E-06	2.73E-06	54658/54575/54659/54657/54578/54577/54600/7364	8
BP	GO:0034754	cellular hormone metabolic process	17/468	117/17913	9.98E-09	3.70E-06	3.44E-06	1646/8644/53630/650/84649/10202/9249/3294/151056/195814/6469/6783/54658/54659/54577/54600/7364	17
BP	GO:0006063	uronic acid metabolic process	8/468	23/17913	7.10E-08	2.20E-05	2.04E-05	54658/54575/54659/54657/54578/54577/54600/7364	8
BP	GO:0019585	glucuronate metabolic process	8/468	23/17913	7.10E-08	2.20E-05	2.04E-05	54658/54575/54659/54657/54578/54577/54600/7364	8
BP	GO:009	ammonium ion	20/	193/1	1.73E-0	4.94E-0	4.59E-0	18/340485/1646/8644/635/54677/1812/79153/2822/3990/4128/84879/4953/64600/255189/151056/126969/80736/60482/55304	20

	7164	metabolic process	468	7913	7	5	5		
BP	GO:0016042	lipid catabolic process	24/468	300/17913	1.29E-06	0.000341946	0.000318127	340485/51/55289/8644/54677/1591/66002/4051/79153/2822/3417/3667/3990/200879/64600/151056/5333/51196/23007/9651/11001/412/6783/6822	24
BP	GO:0008202	steroid metabolic process	24/468	321/17913	4.20E-06	0.001039389	0.00096699	1646/8644/8542/5205/650/1050/1591/1551/84649/10202/51083/3158/3294/3990/114880/255738/6469/11001/6653/412/6783/6822/54658/7364	24
BP	GO:0001655	urogenital system development	24/468	324/17913	4.93E-06	0.001142687	0.001063093	650/100133941/1512/2018/3169/2624/2625/51301/6928/3237/3248/79191/4609/4953/130497/255738/5314/51196/6382/6469/4091/4070/7380/7477	24
BP	GO:0034308	primary alcohol metabolic process	11/468	78/17913	5.53E-06	0.001206612	0.001122565	1646/8644/222/53630/650/84649/9249/151056/195814/6783/6822	11
BP	GO:0042403	thyroid hormone metabolic process	6/468	19/17913	6.26E-06	0.001289771	0.001199931	53905/50506/90527/2625/51301/389434	6
BP	GO:0048839	inner ear development	17/468	185/17913	7.50E-06	0.001464786	0.001362756	5205/650/64072/1050/9620/341640/2624/2625/10804/57822/3784/55283/4610/140469/5816/6469/80736	17
BP	GO:000	flavonoid	5/4	12/17	8.11E-0	0.00150	0.00139	54658/54575/54659/54577/54600	5

© 2020 Yu Y et al. *JAMA Network Open.*

	9812	metabolic process	68	913	6	4154	9382		
BP	GO:0072001	renal system development	22/468	291/17913	8.84E-06	0.001562538	0.001453699	650/100133941/1512/2018/2625/51301/6928/3237/3248/79191/4609/4953/130497/255738/5314/51196/6382/6469/4091/4070/7380/7477	22
BP	GO:0043583	ear development	18/468	212/17913	1.22E-05	0.001980427	0.00184248	5205/650/64072/1050/9620/341640/2624/2625/10804/57822/3784/55283/4610/140469/130497/5816/6469/80736	18
BP	GO:1901568	fatty acid derivative metabolic process	15/468	154/17913	1.28E-05	0.001980427	0.00184248	51/51703/1646/8644/240/66002/126410/4051/11283/84649/124975/3158/3248/5019/255189	15
BP	GO:0001822	kidney development	21/468	276/17913	1.28E-05	0.001980427	0.00184248	650/100133941/1512/2625/51301/6928/3237/3248/79191/4609/4953/130497/255738/5314/51196/6382/6469/4091/4070/7380/7477	21
BP	GO:0006575	cellular modified amino acid metabolic process	16/468	179/17913	1.96E-05	0.002907023	0.002704534	10840/635/54677/53905/50506/90527/2625/51301/124975/2947/2948/3417/389434/64600/255189/347735	16
BP	GO:0001523	retinoid metabolic process	11/468	91/17913	2.48E-05	0.003539267	0.003292739	8644/53630/84649/9249/151056/6382/195814/54658/54659/54577/54600	11

© 2020 Yu Y et al. *JAMA Network Open.*

BP	GO:0072080	nephron tubule development	11/468	93/17913	3.05E-05	0.004188279	0.003896543	650/100133941/2625/6928/3237/79191/4609/130497/6469/4070/7477	11
BP	GO:0006066	alcohol metabolic process	23/468	339/17913	3.16E-05	0.004188279	0.003896543	340485/1646/8644/222/8542/53630/650/1050/84649/9249/3158/8821/3990/255738/151056/5333/51196/23007/9651/195814/6653/6783/6822	23
BP	GO:0060993	kidney morphogenesis	11/468	95/17913	3.73E-05	0.004608686	0.004287666	650/2625/51301/6928/3237/79191/4609/130497/6469/4070/7477	11
BP	GO:0061326	renal tubule development	11/468	95/17913	3.73E-05	0.004608686	0.004287666	650/100133941/2625/6928/3237/79191/4609/130497/6469/4070/7477	11
BP	GO:0006590	thyroid hormone generation	5/468	16/17913	4.10E-05	0.004907651	0.004565807	53905/50506/90527/2625/389434	5
BP	GO:0016101	diterpenoid metabolic process	11/468	97/17913	4.53E-05	0.005252396	0.004886539	8644/53630/84649/9249/151056/6382/195814/54658/54659/54577/54600	11
BP	GO:0090185	negative regulation of kidney development	5/468	17/17913	5.69E-05	0.006391349	0.005946158	2625/6928/130497/6469/4070	5

© 2020 Yu Y et al. *JAMA Network Open.*

BP	GO:0015672	monovalent inorganic cation transport	28/468	477/17913	6.15E-05	0.006710465	0.006243045	480/245973/776/1528/1812/5349/53828/51083/23171/3748/3755/3772/8645/3783/3784/130497/255738/148811/6338/6340/6564/81031/83959/60482/55117/6549/389015/54997	28
BP	GO:0006690	icosanoid metabolic process	11/468	102/17913	7.22E-05	0.007652208	0.007119191	51/1646/8644/240/66002/126410/4051/11283/124975/3248/255189	11
BP	GO:0072073	kidney epithelium development	13/468	140/17913	7.89E-05	0.008126092	0.007560067	650/100133941/2625/6928/3237/79191/4609/130497/6382/6469/4091/4070/7477	13
BP	GO:0006631	fatty acid metabolic process	22/468	339/17913	9.02E-05	0.009045105	0.008415066	51/55289/80221/51703/1646/8644/240/634/54677/57834/66002/4051/11283/84649/79152/2948/3248/3667/3990/255189/5468/11001	22
BP	GO:0017001	antibiotic catabolic process	7/468	42/17913	9.71E-05	0.009478243	0.008818034	8644/222/53905/50506/6783/6822/54658	7
BP	GO:0010876	lipid localization	23/468	366/17913	0.000101894	0.009516203	0.00885335	8714/8542/80832/23120/5205/2583/634/54677/84649/83850/51083/3783/3990/84879/114880/255738/64600/255189/5468/6469/11001/28234/6653	23
BP	GO:0006721	terpenoid metabolic process	11/468	106/17913	0.000102601	0.009516203	0.00885335	8644/53630/84649/9249/151056/6382/195814/54658/54659/54577/54600	11

© 2020 Yu Y et al. *JAMA Network Open.*

BP	GO:0042572	retinol metabolic process	6/468	31/17913	0.000130135	0.01166348	0.010851057	8644/53630/84649/9249/151056/195814	6
BP	GO:0072009	nephron epithelium development	11/468	109/17913	0.000132039	0.01166348	0.010851057	650/100133941/2625/6928/3237/79191/4609/130497/6469/4070/7477	11
BP	GO:1901616	organic hydroxy compound catabolic process	8/468	59/17913	0.000140848	0.012059697	0.011219675	8644/222/1591/66002/4051/4128/6783/6822	8
BP	GO:0072078	nephron tubule morphogenesis	9/468	75/17913	0.000143026	0.012059697	0.011219675	650/2625/6928/3237/79191/4609/130497/6469/4070	9
BP	GO:0042573	retinoic acid metabolic process	5/468	21/17913	0.000171474	0.013829743	0.012866429	8644/54658/54659/54577/54600	5
BP	GO:0072111	cell proliferation involved in kidney development	5/468	21/17913	0.000171474	0.013829743	0.012866429	650/2625/4609/130497/6469	5
BP	GO:0072088	nephron epithelium morpho	9/468	77/17913	0.000175453	0.013849566	0.012884871	650/2625/6928/3237/79191/4609/130497/6469/4070	9

© 2020 Yu Y et al. *JAMA Network Open.*

		genesis							
BP	GO:0006869	lipid transport	21/468	332/17913	0.000184845	0.014286949	0.013291788	8714/8542/80832/23120/5205/634/54677/83850/51083/3783/3990/84879/114880/255738/64600/255189/5468/6469/11001/28234/6653	21
BP	GO:0061333	renal tubule morphogenesis	9/468	79/17913	0.000213781	0.015862567	0.014757656	650/2625/6928/3237/79191/4609/130497/6469/4070	9
BP	GO:0072028	nephron morphogenesis	9/468	79/17913	0.000213781	0.015862567	0.014757656	650/2625/6928/3237/79191/4609/130497/6469/4070	9
BP	GO:0022612	gland morphogenesis	11/468	116/17913	0.000229756	0.016713632	0.01554944	634/1999/80004/3169/4488/59277/5080/10512/6469/6909/6926	11
BP	GO:0001657	ureteric bud development	10/468	98/17913	0.000241831	0.017253686	0.016051875	650/2625/6928/3237/4609/130497/6382/6469/4091/4070	10
BP	GO:0061138	morphogenesis of a branching epithelium	14/468	178/17913	0.000249962	0.017413593	0.016200644	650/9620/1512/80004/3169/6928/3237/4488/4609/59277/10512/6469/4070/6926	14
BP	GO:0015718	monocarboxylic acid transport	12/468	137/17913	0.000255358	0.017413593	0.016200644	18/8714/5205/634/54677/84879/64600/255189/5468/6566/11001/28234	12

© 2020 Yu Y et al. *JAMA Network Open.*

BP	GO:0072163	mesonephric epithelium development	10/468	99/17913	0.000262847	0.017413593	0.016200644	650/2625/6928/3237/4609/130497/6382/6469/4091/4070	10
BP	GO:0072164	mesonephric tubule development	10/468	99/17913	0.000262847	0.017413593	0.016200644	650/2625/6928/3237/4609/130497/6382/6469/4091/4070	10
BP	GO:0021978	telencephalon regionalization	4/468	13/17913	0.000272642	0.017439718	0.01622495	650/2018/5080/6469	4
BP	GO:0032530	regulation of microvillus organization	4/468	13/17913	0.000272642	0.017439718	0.01622495	5205/6624/5357/5909	4
BP	GO:0046470	phosphatidylcholine metabolic process	9/468	82/17913	0.00028413	0.017866477	0.016621983	79153/2822/3990/84879/64600/255189/151056/126969/80736	9
BP	GO:0051187	cofactor catabolic process	7/468	50/17913	0.00030028	0.01826291	0.016990802	10840/57834/53905/50506/124975/54658/54657	7
BP	GO:0060425	lung morphogenesis	7/468	50/17913	0.00030028	0.01826291	0.016990802	9620/1512/80004/3169/3397/6469/7477	7
BP	GO:	isopren	1	12	0.00	0.01	0.01	8644/53630/84649/9249/151056/6382/195814/54658/54659/54577/54600	1

© 2020 Yu Y et al. *JAMA Network Open.*

	0006720	oid metabolic process	1/468	1/17913	0332152	9711275	8338281		1
BP	GO:0021871	forebrain regionalization	5/468	24/17913	0.000335739	0.019711275	0.018338281	650/2018/5080/6469/7477	5
BP	GO:0015850	organic hydroxy compound transport	16/468	228/17913	0.000341194	0.019711275	0.018338281	18/8714/5205/634/1812/51083/3990/114880/255738/5468/10928/6469/6566/6532/28234/6623	16
BP	GO:0072171	mesonephric tubule morphogenesis	8/468	67/17913	0.000345346	0.019711275	0.018338281	650/2625/6928/3237/4609/130497/6469/4070	8
BP	GO:0072006	nephron development	12/468	142/17913	0.000355571	0.019987392	0.018595165	650/100133941/2625/6928/3237/79191/4609/130497/51196/6469/4070/7477	12
BP	GO:0001823	mesonephros development	10/468	103/17913	0.000362839	0.020091519	0.018692038	650/2625/6928/3237/4609/130497/6382/6469/4091/4070	10
BP	GO:0050891	multicellular organismal water homeostasis	8/468	68/17913	0.000382601	0.020874245	0.019420244	1364/66002/79152/29841/57822/4645/6338/6340	8

BP	GO:0006665	sphingolipid metabolic process	12/468	144/17913	0.000404071	0.021726141	0.020212801	340485/222/2583/83850/79152/2525/2528/347735/55304/165679/8869/412	12
BP	GO:0048754	branching morphogenesis of an epithelial tube	12/468	146/17913	0.000458044	0.024276354	0.022585378	650/9620/1512/80004/3169/6928/3237/4488/4609/6469/4070/6926	12
BP	GO:0034310	primary alcohol catabolic process	4/468	15/17913	0.000499317	0.025728721	0.02393658	8644/222/6783/6822	4
BP	GO:0051923	sulfation	4/468	15/17913	0.000499317	0.025728721	0.02393658	222537/6783/6822/10090	4
BP	GO:0006643	membrane lipid metabolic process	14/468	191/17913	0.000511867	0.026014071	0.024202054	340485/222/2583/83850/79152/2525/2528/2822/347735/55304/165679/6484/8869/412	14
BP	GO:0001763	morphogenesis of a branching structure	14/468	192/17913	0.000539265	0.027036128	0.025152919	650/9620/1512/80004/3169/6928/3237/4488/4609/59277/10512/6469/4070/6926	14
BP	GO:0016999	antibiotic metabolic	11/46	130/17913	0.000613255	0.030273266	0.028164573	1646/8644/222/1580/53905/50506/90527/3417/6783/6822/54658	11

© 2020 Yu Y et al. *JAMA Network Open.*

		process	8						
BP	GO:0030856	regulation of epithelial cell differentiation	12/468	151/17913	0.000620153	0.030273266	0.028164573	84913/100133941/634/1515/2625/29841/3397/4488/4610/130497/5080/6926	12
BP	GO:0006692	prostanoid metabolic process	6/468	41/17913	0.000637395	0.030317141	0.028205392	51/1646/8644/11283/3248/255189	6
BP	GO:0006693	prostaglandin metabolic process	6/468	41/17913	0.000637395	0.030317141	0.028205392	51/1646/8644/11283/3248/255189	6
BP	GO:0072189	ureter development	4/468	16/17913	0.000652088	0.030623352	0.028490274	2018/2625/130497/6469	4
BP	GO:0090183	regulation of kidney development	7/468	57/17913	0.000679418	0.03118173	0.029009758	100133941/2625/6928/4609/130497/6469/4070	7
BP	GO:0030104	water homeostasis	8/468	74/17913	0.000680787	0.03118173	0.029009758	1364/66002/79152/29841/57822/4645/6338/6340	8
BP	GO:0003002	regionalization	19/468	318/17913	0.000741454	0.033546276	0.031209602	8092/650/9620/2018/3169/6928/3237/79191/4488/130497/5080/5046/168507/10512/6405/6469/4091/6926/7477	19
BP	GO:	long-ch	9/	94/	0.00	0.03	0.03	51/51703/8644/240/66002/4051/2948/3248/11001	9

© 2020 Yu Y et al. *JAMA Network Open.*

	0001676	ain fatty acid metabolic process	468	17913	0783281	5011705	2572955		
BP	GO:0060441	epithelial tube branching involved in lung morphogenesis	5/468	29/17913	0.000842445	0.037061799	0.03448025	9620/1512/80004/3169/6469	5
BP	GO:0050673	epithelial cell proliferation	23/468	425/17913	0.000849125	0.037061799	0.03448025	84913/650/634/26298/2064/80004/9982/2624/2625/6928/3397/3486/4609/130497/5080/5468/5797/195814/9037/6469/4070/7049/7051	23
BP	GO:0060411	cardiac septum morphogenesis	8/468	77/17913	0.000888025	0.038308972	0.03564055	9249/8701/4488/10512/4091/6909/6926/7049	8
BP	GO:0018958	phenol-containing compound metabolic process	9/468	96/17913	0.000912059	0.038893563	0.036184421	18/1812/53905/50506/90527/2625/51301/389434/4128	9
BP	GO:0005996	monosaccharide metabolic process	17/468	277/17913	0.001034638	0.043619402	0.04058108	669/1528/84649/2203/2528/10690/2822/3486/3667/54658/54575/54659/54657/54578/54577/54600/7364	17

BP	GO:0033559	unsaturated fatty acid metabolic process	9/468	98/17913	0.001057519	0.044083098	0.041012478	51/1646/8644/240/66002/4051/11283/3248/255189	9
BP	GO:0007389	pattern specification process	22/468	408/17913	0.001144647	0.047184899	0.043898222	8092/650/9620/8701/2018/3169/57822/6928/3237/79191/4488/130497/5080/5046/168507/10512/6405/6469/4091/6909/6926/7477	22
CC	GO:0045177	apical part of cell	30/491	365/18678	3.87E-08	1.30E-05	1.19E-05	5205/762/634/22802/1364/1515/66002/1600/53905/50506/2064/2065/10804/9455/4118/4257/200958/4645/100506658/5314/151056/92840/6338/6340/80736/113278/389015/11045/7348/7380	30
CC	GO:0001533	cornified envelope	10/491	44/18678	1.60E-07	2.69E-05	2.47E-05	1825/1830/2125/645027/3713/5493/6700/6703/6707/7051	10
CC	GO:0016324	apical plasma membrane	24/491	300/18678	1.45E-06	0.000162905	0.000149534	5205/762/634/22802/1364/66002/53905/50506/2064/2065/10804/4118/200958/100506658/5314/151056/6338/6340/80736/113278/389015/11045/7348/7380	24
CC	GO:0045095	keratin filament	10/491	97/18678	0.000234009	0.012134544	0.011138538	3875/3852/3853/3854/286887/3855/3887/440050/57830/3846	10
CC	GO:0005902	microvillus	9/491	80/18678	0.000246843	0.012134544	0.011138538	634/1515/3169/6624/200958/55200/150696/6286/84958	9

© 2020 Yu Y et al. *JAMA Network Open.*

CC	GO:0098858	actin-based cell projection	15/491	200/18678	0.0002738111	0.0121345444	0.0111385388	5205/64072/634/1515/3169/6624/9455/200958/140469/55200/150696/5816/6286/84958/57451	15
CC	GO:0031528	microvillus membrane	5/491	23/18678	0.0002796196	0.0121345444	0.0111385388	634/200958/150696/6286/84958	5
CC	GO:0016323	basolateral plasma membrane	15/491	201/18678	0.0002889188	0.0121345444	0.0111385388	634/1364/1366/2064/2065/3248/3784/200958/150696/57127/6563/83959/389015/28234/4070	15
CC	GO:0030057	desmosome	5/491	25/18678	0.0004227688	0.0157833244	0.0144878266	1825/1830/2125/79983/5493	5
CC	GO:0016327	apicolateral plasma membrane	4/491	19/18678	0.00133566	0.0433220111	0.0397661322	1364/1366/3880/100506658	4
CC	GO:0098862	cluster of actin-based cell projections	11/491	143/18678	0.00141828	0.0433220111	0.0397661322	5205/762/64072/1600/9455/79784/140469/5357/5816/85477/6564	11
MF	GO:0015020	glucuronosyltransferase activity	9/466	32/16969	1.32E-07	7.19E-05	6.08E-05	54658/54575/54659/54657/54579/54578/54577/54600/7364	9
MF	GO:002	heme binding	13/	106/1	6.66E-0	0.00182	0.00153	1528/1591/1551/1580/57834/66002/126410/4051/11283/163720/53905/50506/79152	13

© 2020 Yu Y et al. *JAMA Network Open.*

	0037		466	6969	6	1634	8915		
MF	GO:0046906	tetrapyrrole binding	13/466	116/16969	1.80E-05	0.003274347	0.002766166	1528/1591/1551/1580/57834/66002/126410/4051/11283/163720/53905/50506/79152	13
MF	GO:0016758	transferase activity, transferring hexosyl groups	16/466	194/16969	9.35E-05	0.011060884	0.009344228	10331/2583/2525/2528/10690/51301/3038/54658/54575/54659/54657/54579/54578/54577/54600/7364	16
MF	GO:0008194	UDP-glycosyltransferase activity	13/466	138/16969	0.000111542	0.011060884	0.009344228	10331/2583/51301/3038/54658/54575/54659/54657/54579/54578/54577/54600/7364	13
MF	GO:0004497	monooxygenase activity	10/466	86/16969	0.000121326	0.011060884	0.009344228	1646/8644/1591/1551/1580/57834/66002/126410/4051/11283	10
MF	GO:0016757	transferase activity, transferring glycosyl groups	18/466	257/16969	0.000277359	0.021673591	0.018309837	10331/2583/2525/2528/10690/51301/3038/6484/8869/54658/54575/54659/54657/54579/54578/54577/54600/7364	18
MF	GO:0003779	actin binding	22/466	361/16969	0.000435143	0.026355829	0.022265388	634/57530/22837/54869/6624/9455/22998/126353/79784/140469/4645/55930/79983/6275/85477/134549/6532/54212/84501/7134/50853/7739	22

© 2020 Yu Y et al. *JAMA Network Open.*

MF	GO:0048037	cofactor binding	25/466	438/16969	0.000482995	0.026355829	0.022265388	18/51/150209/1528/1591/1551/1580/57834/66002/126410/4051/11283/163720/53905/50506/79152/2330/23171/2947/2948/3248/3417/389434/4257/55304	25
MF	GO:0051015	actin filament binding	12/466	143/16969	0.000588459	0.026355829	0.022265388	6624/126353/79784/4645/55930/79983/85477/134549/6532/7134/50853/7739	12
MF	GO:0008028	monocarboxylic acid transmembrane transporter activity	5/466	26/16969	0.000623842	0.026355829	0.022265388	8714/634/84879/6566/28234	5
MF	GO:0016405	CoA-ligase activity	5/466	26/16969	0.000623842	0.026355829	0.022265388	80221/51703/6296/142827/11001	5
MF	GO:0005506	iron ion binding	12/466	144/16969	0.000626373	0.026355829	0.022265388	240/1591/1551/1580/57834/66002/126410/4051/11283/163720/79152/4241	12
MF	GO:0008324	cation transmembrane transporter activity	26/466	484/16969	0.000920875	0.032164426	0.027172488	480/774/776/1528/53828/3748/3755/8645/3783/3784/152519/348938/168507/57127/6338/6340/6564/81031/29986/80736/83959/60482/55117/6532/57156/55503	26
MF	GO:0005001	transmembrane receptor protein	4/466	17/16969	0.001005448	0.032164426	0.027172488	5797/5801/10076/5803	4

© 2020 Yu Y et al. *JAMA Network Open.*

		tyrosine phosphatase activity							
MF	GO:0019198	transmembrane receptor protein phosphatase activity	4/466	17/16969	0.001005448	0.032164426	0.027172488	5797/5801/10076/5803	4
MF	GO:0046873	metal ion transmembrane transporter activity	20/466	337/16969	0.001082121	0.032164426	0.027172488	480/774/776/53828/3748/3755/8645/3783/3784/152519/348938/168507/6338/6340/29986/83959/60482/55117/6532/55503	20
MF	GO:0004252	serine-type endopeptidase activity	11/466	133/16969	0.001109703	0.032164426	0.027172488	1512/5655/11012/26085/341277/5046/255738/64063/54933/7113/56649	11
MF	GO:0033293	monocarboxylic acid binding	7/466	59/16969	0.001119008	0.032164426	0.027172488	1646/57834/5468/54658/54659/54577/54600	7
MF	GO:0005200	structural constituent of cytoskeleton	8/466	77/16969	0.00122042	0.032164426	0.027172488	3880/54474/3852/3853/3854/5357/5493/50853	8

© 2020 Yu Y et al. *JAMA Network Open.*

MF	GO:0016878	acid-thiol ligase activity	5/466	30/16969	0.001234832	0.032164426	0.027172488	80221/51703/6296/142827/11001	5
MF	GO:0022890	inorganic cation transmembrane transporter activity	24/466	444/16969	0.001316667	0.032737125	0.027656303	480/774/776/1528/53828/3748/3755/8645/3783/3784/152519/348938/168507/6338/6340/6564/81031/29986/83959/60482/55117/6532/57156/55503	24
MF	GO:0016705	oxidoreductase activity, acting on paired donors, with incorporation or reduction of molecular oxygen	11/466	137/16969	0.001412357	0.033589528	0.028376412	1646/8644/1591/1551/1580/57834/66002/126410/4051/11283/163720	11
MF	GO:0001972	retinoic acid binding	4/466	19/16969	0.00156773	0.035731187	0.030185684	54658/54659/54577/54600	4
MF	GO:0008519	ammonium transmembrane transporter	4/466	20/16969	0.001917554	0.038754287	0.032739598	57127/80736/60482/6532	4

© 2020 Yu Y et al. *JAMA Network Open.*

		activity							
MF	GO:0016709	oxidoreductase activity, acting on paired donors, with incorporation or reduction of molecular oxygen, NAD(P)H as one donor, and incorporation of one atom of oxygen	5/466	33/16969	0.001922236	0.038754287	0.032739598	1646/8644/57834/66002/4051	5
MF	GO:0004620	phospholipase activity	8/466	83/16969	0.001983724	0.038754287	0.032739598	2822/3990/200879/64600/255189/151056/51196/23007	8
MF	GO:0072341	modified amino acid binding	7/466	65/16969	0.001983766	0.038754287	0.032739598	8416/2947/2948/4257/84069/85477/54843	7
MF	GO:000	acyl-CoA ligase	3/4	10/16	0.00213	0.03898	0.03293	80221/51703/11001	3

© 2020 Yu Y et al. *JAMA Network Open.*

	3996	activity	66	969	8144	5499	4926		
MF	GO:0070696	transmembrane receptor protein serine/threonine kinase binding	3/466	10/16969	0.002138144	0.038985499	0.032934926	650/651/4091	3
MF	GO:0070330	aromatase activity	4/466	21/16969	0.002317892	0.04089958	0.03455194	1551/1580/66002/11283	4
MF	GO:0022838	substrate-specific channel activity	19/466	338/16969	0.002638657	0.043105329	0.036415355	8542/774/776/22802/1364/5349/53828/3748/3755/8645/3783/3784/168507/6338/6340/6563/83959/57156/55503	19
MF	GO:0008509	anion transmembrane transporter activity	15/466	240/16969	0.002690144	0.043105329	0.036415355	8714/8542/5205/634/22802/1364/5349/84879/6566/80736/83959/113278/55117/6532/28234	15
MF	GO:1901618	organic hydroxy compound transmembrane transporter activity	5/466	36/16969	0.00285435	0.043105329	0.036415355	8714/634/6566/6532/28234	5
MF	GO:004329	glutathione binding	3/46	11/1696	0.00288035	0.04310532	0.03641535	2947/2948/4257	3

	5		6	9	1	9	5		
MF	GO:0015077	monovalent inorganic cation transmembrane transporter activity	16/466	266/16969	0.002890343	0.043105329	0.036415355	480/1528/53828/3748/3755/8645/3783/3784/6338/6340/6564/81031/83959/60482/55117/6532	16
MF	GO:0016616	oxidoreductase activity, acting on the CH-OH group of donors, NAD or NADP as acceptor	9/466	108/16969	0.002915717	0.043105329	0.036415355	1646/8644/650/10202/9249/3248/3294/3417/195814	9
MF	GO:0016712	oxidoreductase activity, acting on paired donors, with incorporation or reduction of molecul	4/466	23/16969	0.003283946	0.04727154	0.039934967	1551/1580/66002/11283	4

		ar oxygen, reduced flavin or flavoprotein as one donor, and incorporation of one atom of oxygen							
MF	GO:0015293	symporter activity	9/466	112/16969	0.003722242	0.049005011	0.041399403	84879/6564/6566/9121/81031/83959/60482/55117/6532	9
MF	GO:0008417	fucosyltransferase activity	3/466	12/16969	0.003762725	0.049005011	0.041399403	2525/2528/10690	3
MF	GO:0015125	bile acid transmembrane transporter activity	3/466	12/16969	0.003762725	0.049005011	0.041399403	8714/634/28234	3
MF	GO:1900750	oligopeptide binding	3/466	12/16969	0.003762725	0.049005011	0.041399403	2947/2948/4257	3

© 2020 Yu Y et al. *JAMA Network Open.*

eTable 11. Gene Ontology Pathway Enrichment Analysis of Immune-Dysfunctional Class in IMvigor210 Trial

Ontology	ID	Description	Gene ratio	Bg ratio	*P* value	*P* adjust	Q value	Gene ID	Count
BP	GO:0042110	T cell activation	102/926	443/17913	6.98E-39	3.54E-35	2.61E-35	100/199/301/54518/384/6363/6347/6366/6352/729230/1236/911/914/30835/939/29126/940/915/916/917/960/923/972/941/942/925/1021/64581/11151/1378/1493/1525/56603/1794/1803/1958/1960/8320/2213/10875/10457/1880/84868/3109/3113/3115/3383/29851/3620/3481/3594/3553/3559/3569/3575/3662/3683/3702/3718/120425/3902/54900/3932/3936/10859/10288/11006/4063/4478/3071/197358/114548/64127/5027/5063/80380/5293/5294/26279/639/5588/26191/5788/5880/10125/9750/91543/860/54440/55423/114836/6556/11005/9840/387357/8600/10673/7409/7412/11326/7454/7535	102
BP	GO:0002694	regulation of leukocyte activation	106/926	492/17913	1.32E-37	3.34E-34	2.47E-34	100/30817/199/301/384/55024/695/712/728/6363/6347/6366/6348/6352/729230/1236/930/914/30835/933/939/29126/940/952/916/923/972/941/8832/942/11151/1378/1493/56603/1803/1960/2207/2213/115352/10457/1880/84868/3109/3113/3115/3134/3162/29851/3620/3481/22806/3594/3553/9173/3559/133396/90865/3569/3575/3662/3684/3689/3718/3902/54900/3932/10859/10288/11006/120892/4332/51237/3071/150372/114548/64127/8013/5063/80380/639/5588/26191/5788/5880/10125/9750/64092/54440/55423/56833/6622/11005/26228/6812/9840/10333/7128/8600/10673/9760/54209/7305/7409/7412/11326/7535	106
BP	GO:0007159	leukocyte cell-cell adhesion	84/926	328/17913	9.52E-36	1.61E-32	1.19E-32	100/119/84830/199/301/384/655/6363/6347/6366/6352/729230/1236/30835/939/29126/940/916/960/923/972/941/942/10332/11151/1493/1803/1960/2213/83706/2650/10457/84868/3109/3113/3115/3383/29851/3620/3481/3594/3553/3559/3569/3575/3676/3683/3689/3718/3902/54900/3932/10859/10288/11006/4478/3071/114548/64127/8013/4907/5063/80380/	84

© 2020 Yu Y et al. *JAMA Network Open.*

								50512/5588/26191/5788/5880/10125/9750/6279/54440/6401/6402/6403/6404/55423/9840/8600/10673/7409/7412/11326/7535	
BP	GO:0051249	regulation of lymphocyte activation	87/926	401/17913	3.72E-31	4.71E-28	3.48E-28	100/199/301/384/55024/695/6363/6347/6366/6352/729230/1236/930/914/30835/933/939/29126/940/952/916/923/972/941/942/11151/1378/1493/56603/1803/1960/2213/115352/10457/1880/84868/3109/3113/3115/3134/29851/3620/3481/22806/3594/3553/3559/3569/3575/3662/3718/3902/54900/3932/10859/10288/11006/4332/51237/3071/150372/114548/64127/5063/80380/639/5588/26191/5788/5880/10125/9750/64092/54440/55423/56833/11005/9840/7128/8600/10673/9760/7305/7409/7412/11326/7535	87
BP	GO:0050867	positive regulation of cell activation	77/926	319/17913	6.17E-31	6.25E-28	4.62E-28	100/199/301/695/712/6363/6347/6366/6348/6352/729230/1236/914/30835/939/29126/940/952/916/923/972/941/942/11151/1493/1803/1960/2207/115352/1880/84868/3109/3113/3115/3134/29851/3481/3594/3553/9173/3559/90865/3569/3575/3684/3689/3932/353514/10859/10288/11006/120892/3071/114548/64127/8013/5063/80380/5341/5588/5788/10125/54440/6403/55423/26228/6812/9840/10333/8600/10673/9760/54209/7305/7409/7412/7535	77
BP	GO:0050900	leukocyte migration	93/926	467/17913	3.36E-30	2.83E-27	2.09E-27	100/119/30817/84830/199/301/719/728/6357/6363/6347/6364/6366/6348/6351/6352/729230/1236/914/960/962/972/8832/9023/11151/1441/1525/3627/6373/10563/3576/3577/7852/1843/1906/2207/2358/2650/2817/2838/1880/2995/3055/3162/3383/3603/90865/3569/3675/3676/3680/3683/3684/3687/3689/120425/3512/3897/3932/4318/4354/4478/64005/3071/4973/11240/5142/5293/5294/7941/50512/5800/5880/5979/9750/6279/6401/6402/6403/6404/140885/55423/56833/9056/124976/26228/8600/54210/54209/7409/7412/7422/7535	93
BP	GO:0002696	positive regulation of leukocyte activation	74/926	305/17913	6.46E-30	4.68E-27	3.45E-27	100/199/301/695/712/6363/6347/6366/6348/6352/729230/1236/914/30835/939/29126/940/952/916/923/972/941/942/11151/1493/1803/1960/2207/115352/1880/84868/3109/3113/3115/3134/29851/3481/3594/3553/9173/3559/90865/3569/3575/3684/3689/3932/10859/10288/11006/120892/3071/114548/64127/8013/5063/80380/5588/5788/10125/54440/55423/26228/6812/9840/10333/8600/10673/9760/54209/7305/7409/7412/7535	74

© 2020 Yu Y et al. *JAMA Network Open.*

BP	GO:0002521	leukocyte differentiation	94/926	485/17913	1.46E-29	9.27E-27	6.84E-27	100/222487/301/695/714/6363/6348/729230/1236/135228/930/914/939/940/915/916/972/973/941/942/1021/1378/1380/1436/1493/56603/55619/139818/1794/1958/1960/8320/2124/2207/2213/115352/2322/1880/3059/9734/3428/10320/22806/3594/3559/133396/3569/3575/3662/3676/9452/3718/3725/3902/3932/10859/10288/11006/4057/4063/4318/931/3071/150372/114548/5293/8544/26279/23228/639/26191/5788/10125/91543/860/54440/114836/56833/6688/11005/9840/387357/7097/55365/28959/8600/9760/54209/7305/10537/7409/7412/7422/7535	94
BP	GO:1903037	regulation of leukocyte cell-cell adhesion	69/926	295/17913	6.29E-27	3.54E-24	2.62E-24	100/84830/199/301/384/6363/6347/6366/6352/729230/1236/30835/939/29126/940/916/960/923/972/941/942/11151/1493/1803/1960/2213/10457/84868/3109/3113/3115/3383/29851/3620/3481/3594/3553/3559/3569/3575/3676/3689/3718/3902/54900/3932/10859/10288/11006/3071/114548/64127/8013/5063/80380/5588/26191/5788/10125/9750/54440/55423/9840/8600/10673/7409/7412/11326/7535	69
BP	GO:0050863	regulation of T cell activation	70/926	307/17913	1.37E-26	6.95E-24	5.13E-24	100/199/301/384/6363/6347/6366/6352/729230/1236/914/30835/939/29126/940/916/923/972/941/942/11151/1378/1493/56603/1803/1960/2213/10457/84868/3109/3113/3115/29851/3620/3481/3594/3553/3559/3569/3575/3662/3718/3902/54900/3932/10859/10288/11006/3071/114548/64127/5063/80380/639/5588/26191/5788/5880/10125/9750/54440/55423/11005/9840/8600/10673/7409/7412/11326/7535	70
BP	GO:0030098	lymphocyte differentiation	72/926	325/17913	1.68E-26	7.74E-24	5.71E-24	100/222487/301/695/6363/729230/1236/930/914/939/940/915/916/972/973/941/942/1021/1378/1380/1493/56603/55619/139818/1794/1958/1960/8320/2213/115352/2322/1880/9734/10320/22806/3594/3559/3569/3575/3662/3676/9452/3718/3902/3932/10288/11006/4063/931/3071/150372/114548/5293/26279/23228/639/26191/5788/10125/91543/860/54440/114836/56833/6688/11005/9840/387357/9760/7409/7412/7535	72
BP	GO:1903039	positive regulation of leukocyte cell-cell adhesion	58/926	214/17913	2.45E-26	1.04E-23	7.64E-24	100/199/301/6363/6347/6366/6352/729230/1236/30835/939/29126/940/916/960/923/972/941/942/11151/1493/1803/1960/84868/3109/3113/3115/3383/29851/3481/3594/3553/3559/3569/3575/3676/3689/3932/10859/10288/11006/3071/114548/64127/8013/5063/80380/5588/5788/10125/54440/55423/9840/8600/10673/7409/7412/7535	58

© 2020 Yu Y et al. *JAMA Network Open.*

BP	GO:0070661	leukocyte proliferation	65/926	270/17913	3.51E-26	1.37E-23	1.01E-23	100/199/301/384/695/6363/6352/729230/930/30835/933/29126/940/952/916/923/972/973/11151/1380/1436/1493/1794/2213/115352/2322/10457/1880/84868/3109/3113/3115/3620/3481/22806/3594/3553/3559/90865/3569/3575/10859/10288/4332/931/4478/51237/3071/5027/80380/5294/23228/5588/26191/5788/5880/54440/6556/7128/8600/10673/7305/7412/11326/7535	65
BP	GO:0034341	response to interferon-gamma	52/926	173/17913	4.56E-26	1.65E-23	1.22E-23	199/6347/6348/6352/960/4261/1906/2209/2633/115361/115362/3055/3105/3106/3107/3113/3115/3118/3119/3120/3122/3123/3125/3134/3136/3383/10437/8519/10581/10410/3594/3662/3664/3394/4210/4360/4502/84166/4939/8638/140885/6556/6622/9021/6812/7097/10346/11074/7294/10537/7412/7454	52
BP	GO:0022409	positive regulation of cell-cell adhesion	62/926	251/17913	1.13E-25	3.83E-23	2.83E-23	100/8728/199/301/655/6363/6347/6366/6352/729230/1236/30835/939/29126/940/916/960/923/972/941/942/11151/1493/10563/1803/1960/2651/84868/3109/3113/3115/3383/29851/3481/3594/3553/3559/3569/3575/3676/3689/3932/10859/10288/11006/3071/114548/64127/8013/5063/80380/5588/5788/10125/54440/55423/9840/8600/10673/7409/7412/7535	62
BP	GO:0001819	positive regulation of cytokine production	84/926	446/17913	1.39E-25	4.21E-23	3.10E-23	150/199/9447/301/330/718/719/728/147372/6363/6348/729230/1236/929/914/29126/940/948/916/923/972/941/942/1436/1536/55601/1958/2069/2207/2219/55612/115362/51704/84868/29923/3113/3115/3134/3162/3304/3620/3428/3594/3595/3553/3556/9173/90865/3569/3662/3394/353514/10859/10288/4023/120892/4050/4063/23643/4321/150372/22861/114548/64127/8013/5027/5142/5588/26191/5788/10125/91543/54440/114836/6556/7096/7097/10333/51284/51311/54209/7294/7305/81030	84
BP	GO:0046651	lymphocyte proliferation	61/926	244/17913	1.41E-25	4.21E-23	3.10E-23	100/199/301/384/695/6363/6352/729230/930/30835/933/29126/940/952/916/923/972/973/11151/1380/1493/1794/2213/115352/2322/10457/1880/84868/3109/3113/3115/3620/3481/22806/3594/3553/3559/3569/3575/10859/10288/4332/931/4478/51237/3071/5027/80380/5294/23228/5588/26191/5788/5880/54440/6556/10673/7305/7412/11326/7535	61
BP	GO:0032943	mononuclear cell proliferation	61/926	246/17913	2.26E-	6.38E-	4.71E-23	100/199/301/384/695/6363/6352/729230/930/30835/933/29126/940/952/916/923/972/973/11151/1380/1493/1794/2213/115352/2322/10457/1880/84868/3109/3113/3115/3620/3481/	61

					25	23		22806/3594/3553/3559/3569/3575/10859/10288/4332/931/4478/51237/3071/5027/80380/5294/23228/5588/26191/5788/5880/54440/6556/10673/7305/7412/11326/7535	
BP	GO:0022407	regulation of cell-cell adhesion	77/926	389/17913	6.08E-25	1.62E-22	1.20E-22	100/8728/84830/199/301/384/655/6363/6347/6366/6352/729230/1236/30835/939/29126/940/916/960/923/972/941/942/999/1952/11151/1493/10563/1803/1960/2213/83706/2651/10457/84868/3109/3113/3115/3383/29851/3620/3481/3594/3553/3559/3569/3575/3676/3689/3718/3902/54900/3932/10859/10288/11006/266727/3071/114548/64127/8013/5063/80380/5588/26191/5788/10125/9750/54440/55423/9840/8600/10673/7409/7412/11326/7535	77
BP	GO:0002697	regulation of immune effector process	81/926	441/17913	5.93E-24	1.50E-21	1.11E-21	30817/9447/301/330/695/712/713/714/717/718/719/720/721/728/730/6363/729230/930/933/940/948/972/941/8832/942/10225/629/3426/1378/1380/55601/1776/2207/2208/2213/115352/10875/51704/84868/51191/3109/3134/3162/3434/3594/3553/3559/90865/3569/3575/11213/3662/3684/3689/3718/5650/3902/10859/4321/51237/114548/64127/8013/5027/5788/5880/10125/91543/54440/5272/710/4068/114836/56833/11005/6812/55061/7128/7409/11326/7454	81
BP	GO:0050870	positive regulation of T cell activation	53/926	198/17913	6.97E-24	1.68E-21	1.24E-21	100/199/301/6363/6347/6366/6352/729230/1236/30835/939/29126/940/916/923/972/941/942/11151/1493/1803/1960/84868/3109/3113/3115/29851/3481/3594/3553/3559/3569/3575/3932/10859/10288/11006/3071/114548/64127/5063/80380/5588/5788/10125/54440/55423/9840/8600/10673/7409/7412/7535	53
BP	GO:0051251	positive regulation of lymphocyte activation	60/926	258/17913	2.08E-23	4.79E-21	3.54E-21	100/199/301/695/6363/6347/6366/6352/729230/1236/30835/939/29126/940/952/916/923/972/941/942/11151/1493/1803/1960/115352/1880/84868/3109/3113/3115/3134/29851/3481/3594/3553/3559/3569/3575/3932/10859/10288/11006/3071/114548/64127/5063/80380/5588/5788/10125/54440/55423/9840/8600/10673/9760/7305/7409/7412/7535	60
BP	GO:0002768	immune response-regulating cell surface receptor signaling	78/926	434/17913	1.75E-22	3.86E-20	2.85E-20	100/640/695/719/728/1236/930/30835/933/919/952/915/916/917/973/10462/1378/1380/1493/26999/9844/2207/2209/2212/2213/2214/2219/115352/2358/2533/2633/3055/3113/3115/3118/3119/3120/3122/3123/3125/3702/3725/89857/54900/3932/3937/10859/10288/11006/4332/931/94025/4583/64005/3071/150372/8013/5063/5079/5142/5293/23228/5579/5588/	78

© 2020 Yu Y et al. *JAMA Network Open.*

		pathway						5696/5698/26191/5788/26228/9840/387357/9473/50852/7294/7409/7454/7456/7535	
BP	GO:0060326	cell chemotaxis	58/926	253/17913	2.48E-22	5.23E-20	3.86E-20	30817/199/301/51411/719/728/6357/6363/6347/6364/6366/6348/6351/6352/729230/1236/972/9023/11151/1441/1525/3627/6373/10563/3576/3577/7852/1843/1906/1960/2358/1880/1839/3603/3569/3680/3689/120425/4354/3071/3164/11240/5142/5293/5294/7941/5365/5588/5800/5880/9750/6279/56833/26228/8600/7409/7412/7422	58
BP	GO:0045785	positive regulation of cell adhesion	73/926	388/17913	2.58E-22	5.23E-20	3.86E-20	100/8728/199/301/54518/655/6363/6347/6366/6352/729230/1236/30835/939/29126/940/948/916/960/923/972/941/942/1021/11151/1493/10563/1803/1960/55612/342184/2651/84868/3109/3113/3115/3383/29851/3481/3594/3553/3559/3569/3575/3675/3676/3689/3932/10859/10288/11006/3071/114548/64127/8013/5063/80380/5588/5788/10125/5979/6281/54440/55423/9806/9840/7052/8600/10673/7409/7412/7422/7535	73
BP	GO:0071346	cellular response to interferon-gamma	45/926	153/17913	2.74E-22	5.33E-20	3.94E-20	199/6347/6348/6352/960/4261/1906/2209/2633/115361/115362/3055/3105/3106/3107/3113/3115/3118/3119/3120/3122/3123/3125/3134/3136/3383/10437/3594/3662/3664/3394/4360/4502/84166/4939/8638/140885/9021/6812/7097/10346/11074/7294/7412/7454	45
BP	GO:0002429	immune response-activating cell surface receptor signaling pathway	74/926	399/17913	3.22E-22	6.04E-20	4.46E-20	100/640/695/719/728/1236/930/30835/933/919/952/915/916/917/973/10462/1378/1380/1493/26999/9844/2207/2209/2212/2213/2214/2219/115352/2533/2633/3055/3113/3115/3118/3119/3120/3122/3123/3125/3702/89857/54900/3932/3937/11006/4332/931/94025/4583/64005/3071/150372/8013/5063/5079/5142/5293/23228/5579/5588/5696/5698/26191/5788/26228/9840/387357/9473/50852/7294/7409/7454/7456/7535	74
BP	GO:0050851	antigen receptor-mediated signaling pathway	54/926	224/17913	5.78E-22	1.05E-19	7.72E-20	100/640/695/1236/930/933/919/952/915/916/917/973/1493/2213/115352/2533/2633/3113/3115/3118/3119/3120/3122/3123/3125/3702/89857/54900/3932/3937/11006/4332/931/3071/150372/5063/5079/5142/5293/23228/5579/5588/5696/5698/26191/5788/26228/9840/387357/9473/50852/7294/7454/7535	54
BP	GO:0030595	leukocyte chemotaxis	49/926	188/17913	1.28E-21	2.23E-19	1.65E-19	30817/199/301/719/728/6357/6363/6347/6366/6348/6351/6352/729230/1236/972/9023/11151/1441/1525/3627/6373/10563/3576/3577/7852/1843/1906/2358/1880/3603/3569/3680/3689/120425/4354/3071/11240/5142/5293/5294/7941/5800/5	49

© 2020 Yu Y et al. *JAMA Network Open.*

								880/9750/6279/56833/26228/8600/7409	
BP	GO:0050727	regulation of inflammatory response	81/926	479/17913	1.45E-21	2.46E-19	1.81E-19	54/100/113/301/348/330/695/712/713/714/717/718/719/720/721/728/730/6348/6352/729230/1236/930/940/923/629/3426/1378/1380/1776/2099/2207/2213/2358/115362/51704/3055/3620/3553/7850/9173/3559/90865/3569/3684/3689/353514/4023/120892/4210/4318/197358/22861/114548/64127/4907/26471/9180/5187/118788/5294/7941/5788/6279/6401/710/89790/140885/56833/6622/9021/26228/55061/7052/81793/7097/10333/51284/7128/8600/54209/11326	81
BP	GO:0032103	positive regulation of response to external stimulus	63/926	315/17913	7.90E-21	1.29E-18	9.53E-19	199/695/712/718/719/728/6363/6366/6348/6351/6352/729230/1233/1236/4064/940/923/972/3627/10563/3576/7852/55601/1906/2152/2207/2358/115362/51704/84868/3620/3603/3553/9173/90865/3569/3684/3689/5650/353514/4023/120892/23643/3071/64127/26471/9180/5294/7941/5880/9750/6279/6622/26228/7052/81793/7097/10333/51284/8600/54209/347733/7422	63
BP	GO:0002683	negative regulation of immune system process	77/926	451/17913	8.43E-21	1.33E-18	9.85E-19	100/84830/301/384/55024/695/714/6347/6366/6348/729230/929/933/29126/972/8832/10225/1021/1378/1493/1843/2207/2213/115352/127943/10875/2633/10457/29909/84868/8361/3112/3134/3162/3218/3620/3428/9173/3559/133396/90865/3575/11213/3662/3718/3902/54900/10859/10288/11006/4057/23643/4321/4332/197358/84166/11240/80380/23228/26191/5788/9750/64092/5272/710/56833/6662/11005/26228/10333/55365/28959/7128/1831/7305/11326/7538	77
BP	GO:0050670	regulation of lymphocyte proliferation	49/926	199/17913	1.82E-20	2.80E-18	2.07E-18	100/199/301/384/695/6363/6352/729230/30835/933/29126/940/952/916/923/972/11151/1493/2213/115352/10457/1880/84868/3109/3113/3115/3620/3481/22806/3594/3553/3559/3569/10859/10288/4332/51237/3071/80380/5588/26191/5788/5880/54440/10673/7305/7412/11326/7535	49
BP	GO:0032944	regulation of mononuclear cell proliferation	49/926	200/17913	2.30E-20	3.42E-18	2.53E-18	100/199/301/384/695/6363/6352/729230/30835/933/29126/940/952/916/923/972/11151/1493/2213/115352/10457/1880/84868/3109/3113/3115/3620/3481/22806/3594/3553/3559/3569/10859/10288/4332/51237/3071/80380/5588/26191/5788/5880/54440/10673/7305/7412/11326/7535	49
BP	GO:0002237	response to molecule of bacterial	61/926	309/17913	6.64E-	9.62E-	7.10E-18	54/249/27063/721/728/6347/6348/6352/1236/929/4064/948/923/10225/1439/3627/2920/3576/1906/2213/84868/3055/3383/3620/3587/3595/3553/11009/3569/11213/3394/8564/108	61

© 2020 Yu Y et al. *JAMA Network Open.*

		origin			20	18		59/10288/4057/23643/4129/4360/114548/64127/389643/5027/5142/10891/26191/6279/6401/6403/140885/6556/6622/26228/7035/7054/7096/7097/10333/7128/54209/7412/7538	
BP	GO:0070663	regulation of leukocyte proliferation	50/926	214/17913	8.55E-20	1.20E-17	8.88E-18	100/199/301/384/695/6363/6352/729230/30835/933/29126/940/952/916/923/972/11151/1493/2213/115352/10457/1880/84868/3109/3113/3115/3620/3481/22806/3594/3553/3559/3569/10859/10288/4332/51237/3071/80380/5588/26191/5788/5880/54440/7128/10673/7305/7412/11326/7535	50
BP	GO:0002460	adaptive immune response based on somatic recombination of immune receptors built from immunoglobulin superfamily domains	58/926	295/17913	6.82E-19	9.34E-17	6.89E-17	100/301/695/712/713/714/717/718/720/721/730/6363/729230/930/939/29126/940/972/941/3426/1378/1380/10563/2207/2208/2213/10875/84868/3119/3134/3383/3594/3553/9173/90865/3569/3575/3662/3718/89857/10859/4063/64005/114548/5027/5551/5588/5788/91543/54440/710/114836/6556/55061/51311/7128/10673/7454	58
BP	GO:0060333	interferon-gamma-mediated signaling pathway	32/926	91/17913	7.98E-19	1.06E-16	7.86E-17	960/4261/2209/2633/3055/3105/3106/3107/3113/3115/3118/3119/3120/3122/3123/3125/3134/3136/3383/10437/3662/3664/3394/4502/84166/4939/8638/9021/10346/11074/7294/7412	32
BP	GO:0030217	T cell differentiation	50/926	234/17913	4.92E-18	6.39E-16	4.72E-16	100/301/6363/729230/1236/914/939/940/915/916/972/941/942/1021/1378/1493/56603/1794/1958/1960/8320/1880/3594/3559/3569/3575/3662/3718/3902/3932/10288/11006/4063/3071/114548/5293/26279/639/26191/5788/10125/91543/860/54440/114836/11005/9840/387357/7409/7535	50
BP	GO:0032496	response to lipopolysaccharide	56/926	295/17913	1.49E-17	1.89E-15	1.39E-15	54/249/27063/728/6347/6348/6352/1236/929/4064/948/923/10225/1439/3627/3576/1906/84868/3055/3383/3620/3587/3595/3553/11009/3569/11213/3394/8564/10859/10288/4057/23643/4129/4360/114548/64127/389643/5027/5142/10891/26191/6279/6401/6403/140885/6556/6622/26228/7035/7054/	56

© 2020 Yu Y et al. *JAMA Network Open.*

								7097/7128/54209/7412/7538	
BP	GO:0097529	myeloid leukocyte migration	41/926	170/17913	5.50E-17	6.80E-15	5.02E-15	30817/199/301/719/728/6357/6363/6347/6366/6348/6352/729230/1236/972/1441/1525/3627/3576/1843/1906/2358/3569/3680/3689/120425/4354/3071/5142/5293/5294/7941/5800/5880/9750/6279/140885/56833/26228/8600/54209/7409	41
BP	GO:0006909	phagocytosis	56/926	308/17913	1.13E-16	1.36E-14	1.00E-14	575/199/301/306/51411/717/718/720/721/6347/929/919/948/917/64581/11151/26999/1794/9844/2207/2209/2212/2213/2214/2219/2358/3055/3553/3560/3561/3394/3683/3684/3689/8685/4481/64005/4647/4688/4689/3071/5027/5031/5788/5806/140885/6504/6556/26228/7052/7097/54209/7305/7409/7454/7456	56
BP	GO:0042119	neutrophil activation	74/926	499/17913	1.69E-16	1.99E-14	1.47E-14	51816/84658/222487/290/306/23526/64333/10396/51411/718/719/728/6352/929/948/960/963/978/1116/1118/160364/1378/1476/2919/3576/3577/1536/8560/1776/1794/2171/2204/2207/2212/2213/2215/2219/10875/2357/2358/11010/3101/3136/3304/10788/3683/3684/3687/3689/3903/10288/4057/4069/4318/4332/3071/150372/4973/11240/51316/5329/5788/5806/54509/6279/6402/5265/140885/10326/6556/6515/6518/7097/7305	74
BP	GO:0042098	T cell proliferation	41/926	177/17913	2.55E-16	2.94E-14	2.17E-14	199/301/384/6363/6352/729230/30835/29126/940/916/923/11151/1493/1794/10457/84868/3109/3113/3115/3620/3481/3594/3553/3559/3569/10859/10288/4478/3071/5027/80380/5294/5588/5788/5880/54440/6556/10673/7412/11326/7535	41
BP	GO:1902105	regulation of leukocyte differentiation	51/926	267/17913	3.27E-16	3.68E-14	2.72E-14	100/301/695/714/6363/6348/729230/914/939/940/972/941/942/1021/1378/1493/56603/1960/2124/2213/115352/3059/22806/3594/3559/3575/3662/3718/3725/3902/10859/10288/11006/4057/3071/150372/114548/639/5788/10125/54440/56833/11005/9840/55365/28959/8600/9760/54209/7305/7535	51
BP	GO:0045088	regulation of innate immune response	68/926	440/17913	3.59E-16	3.96E-14	2.92E-14	9447/348/330/695/6352/929/30835/948/10225/10462/64581/1378/1514/55601/2069/2099/2207/2213/2219/115352/2358/115362/84868/3055/3134/3304/3428/11213/3662/3684/3689/3902/10859/4057/23643/8685/4321/4332/94025/4583/197358/84166/64127/5063/118788/5696/5698/26191/149628/10125/91543/6279/5272/710/4068/114836/56833/9021/7096/81793/7097/10333/51284/51311/7128/7294/7409/11326	68
B	GO:00	B cell	45/92	215/1791	5.	5.	4.24E-14	100/222487/55024/695/930/933/939/940/952/972/973/1380/	4

© 2020 Yu Y et al. *JAMA Network Open.*

P	42113	activation	6	3	32E-16	74E-14		1493/55619/139818/2213/115350/115352/2322/1880/9734/22806/3569/3575/3676/9452/3718/54900/4332/931/51237/3071/150372/64127/5293/23228/5579/5788/64092/56833/7128/10673/7305/7412/7535	5
BP	GO:0002449	lymphocyte mediated immunity	53/926	293/17913	9.49E-16	1.00E-13	7.39E-14	695/712/713/714/717/718/720/721/730/729230/930/939/940/972/10225/3426/11151/1378/1380/2207/2208/2213/10875/3002/84868/3119/3134/3383/3594/3553/3569/3575/3822/3902/10859/64005/114548/5027/5551/5788/10125/91543/54440/5272/710/4068/114836/57823/6556/55061/51311/7409/7454	53
BP	GO:0019882	antigen processing and presentation	45/926	220/17913	1.32E-15	1.37E-13	1.01E-13	6363/6366/1236/30835/948/972/925/10332/1514/1536/64167/2209/2213/10875/3105/3106/3107/3108/3109/3112/3113/3115/3118/3119/3120/3122/3123/3125/3134/3136/3383/10437/3902/10288/653361/4688/4689/64127/5696/5698/6556/6890/6891/54209/7454	45
BP	GO:0002446	neutrophil mediated immunity	72/926	499/17913	1.91E-15	1.94E-13	1.43E-13	51816/84658/222487/290/306/23526/64333/10396/51411/718/719/728/929/948/960/963/978/1116/1118/160364/1378/1476/2919/3577/1536/8560/1776/1794/2171/2204/2207/2212/2215/2219/10875/2357/2358/11010/3101/3136/3304/3569/10788/3683/3684/3687/3689/3903/10288/4057/4069/4318/4332/3071/150372/4973/11240/51316/5329/5788/5806/54509/6279/6402/5265/140885/10326/6556/6515/6518/7097/7305	72
BP	GO:0002283	neutrophil activation involved in immune response	71/926	488/17913	1.96E-15	1.95E-13	1.44E-13	51816/84658/222487/290/306/23526/64333/10396/51411/718/719/728/929/948/960/963/978/1116/1118/160364/1378/1476/2919/3577/1536/8560/1776/1794/2171/2204/2207/2212/2215/2219/10875/2357/2358/11010/3101/3136/3304/10788/3683/3684/3687/3689/3903/10288/4057/4069/4318/4332/3071/150372/4973/11240/51316/5329/5788/5806/54509/6279/6402/5265/140885/10326/6556/6515/6518/7097/7305	71
BP	GO:0002699	positive regulation of immune effector process	42/926	198/17913	3.09E-15	3.01E-13	2.22E-13	30817/301/695/718/6363/729230/940/948/972/941/8832/942/55601/2207/2208/51704/3109/3134/3162/3594/3553/90865/3569/3684/3689/5650/3902/10859/51237/114548/64127/8013/5027/5788/5880/10125/91543/54440/4068/114836/6812/7409	42
BP	GO:0050671	positive regulation of lymphocyte	33/926	125/17913	3.64E-	3.48E-	2.57E-13	100/199/301/6363/6352/729230/30835/29126/940/952/916/923/972/11151/115352/1880/84868/3109/3113/3115/3481/3594/3553/3559/3569/10288/3071/5588/5788/54440/10673/74	33

© 2020 Yu Y et al. *JAMA Network Open.*

		proliferation			15	13		12/7535	
BP	GO:0042129	regulation of T cell proliferation	36/926	149/17913	4.20E-15	3.94E-13	2.91E-13	199/301/384/6363/6352/729230/30835/29126/940/916/923/11151/1493/10457/84868/3109/3113/3115/3620/3481/3594/3553/3559/3569/10859/10288/3071/80380/5588/5788/5880/54440/10673/7412/11326/7535	36
BP	GO:0042102	positive regulation of T cell proliferation	28/926	90/17913	4.56E-15	4.20E-13	3.10E-13	199/301/6363/6352/729230/30835/29126/940/916/923/11151/84868/3109/3113/3115/3481/3594/3553/3559/3569/10288/3071/5588/5788/54440/10673/7412/7535	28
BP	GO:0032946	positive regulation of mononuclear cell proliferation	33/926	126/17913	4.69E-15	4.20E-13	3.10E-13	100/199/301/6363/6352/729230/30835/29126/940/952/916/923/972/11151/115352/1880/84868/3109/3113/3115/3481/3594/3553/3559/3569/10288/3071/5588/5788/54440/10673/7412/7535	33
BP	GO:0043312	neutrophil degranulation	70/926	485/17913	4.72E-15	4.20E-13	3.10E-13	51816/84658/222487/290/306/23526/64333/10396/51411/718/719/728/929/948/960/963/978/1116/1118/160364/1378/1476/2919/3577/1536/8560/1794/2171/2204/2207/2212/2215/2219/10875/2357/2358/11010/3101/3136/3304/10788/3683/3684/3687/3689/3903/10288/4057/4069/4318/4332/3071/150372/4973/11240/51316/5329/5788/5806/54509/6279/6402/5265/140885/10326/6556/6515/6518/7097/7305	70
BP	GO:0002819	regulation of adaptive immune response	36/926	150/17913	5.26E-15	4.59E-13	3.39E-13	100/113/301/695/718/6363/729230/29126/940/962/941/1378/2207/2208/2213/84868/3134/3594/3553/9173/90865/3569/3575/3718/10859/114548/5027/5588/5788/91543/64092/54440/6556/7128/10673/7454	36
BP	GO:0071621	granulocyte chemotaxis	27/926	84/17913	5.64E-15	4.85E-13	3.58E-13	30817/301/719/728/6357/6363/6366/6348/6352/1236/972/1441/1525/3576/1906/3680/3689/120425/4354/3071/5142/5293/5294/5880/9750/6279/7409	27
BP	GO:0032609	interferon-gamma production	30/926	105/17913	6.38E-15	5.39E-13	3.98E-13	729230/1236/929/914/29126/916/10225/8320/84868/3113/3115/3594/3595/3553/9173/90865/3394/3702/10859/80380/5142/26191/10125/54440/140885/114836/6556/51284/51311/7294	30
BP	GO:0030593	neutrophil chemotaxis	23/926	60/17913	7.39E-	6.09E-	4.49E-13	719/728/6363/6366/6348/1236/972/1441/1525/3576/1906/3680/3689/120425/4354/3071/5142/5293/5294/5880/9750/6279/7409	23

© 2020 Yu Y et al. *JAMA Network Open.*

					15	13			
BP	GO:0050663	cytokine secretion	45/926	230/17913	7.44E-15	6.09E-13	4.49E-13	199/9447/301/384/55024/6363/6348/1236/929/914/29126/948/1116/1436/2213/2219/55612/2633/115362/84868/9734/3553/7850/3556/9173/90865/3937/353514/10859/4023/120892/4321/22861/114548/64127/5027/26191/10125/5552/7096/81793/7097/10333/51311/7128	45
BP	GO:0050729	positive regulation of inflammatory response	36/926	153/17913	1.02E-14	8.20E-13	6.05E-13	695/712/718/6348/729230/1236/940/923/2207/115362/51704/3620/3553/9173/90865/3569/3684/3689/353514/4023/120892/64127/26471/9180/5294/7941/6279/6622/26228/7052/81793/7097/10333/51284/8600/54209	36
BP	GO:0032649	regulation of interferon-gamma production	28/926	93/17913	1.17E-14	9.24E-13	6.82E-13	729230/1236/929/914/29126/916/10225/84868/3113/3115/3594/3595/3553/9173/90865/3394/10859/80380/5142/26191/10125/54440/140885/114836/6556/51284/51311/7294	28
BP	GO:0072676	lymphocyte migration	27/926	87/17913	1.51E-14	1.17E-12	8.67E-13	84830/199/6347/6364/6366/6348/6351/6352/729230/1236/9023/3627/6373/10563/2838/1880/3383/3676/4478/64005/11240/5293/5294/5979/9750/124976/7535	27
BP	GO:0045619	regulation of lymphocyte differentiation	37/926	164/17913	1.80E-14	1.38E-12	1.02E-12	100/301/695/6363/729230/914/939/940/972/941/942/1378/1493/56603/1960/115352/22806/3594/3559/3575/3662/3718/3902/10288/11006/3071/150372/114548/639/5788/10125/54440/56833/11005/9840/9760/7535	37
BP	GO:0006959	humoral immune response	49/926	276/17913	2.50E-14	1.89E-12	1.40E-12	712/713/714/717/718/719/720/721/728/730/6357/6347/729230/1236/930/940/629/3426/1378/1380/10563/2208/2213/2219/10578/1880/3119/3553/3569/3512/5650/4057/4069/931/5079/5266/5450/5788/6279/710/4068/6556/11005/124976/6480/55061/54210/54209/11326	49
BP	GO:0071219	cellular response to molecule of bacterial origin	39/926	183/17913	2.59E-14	1.93E-12	1.43E-12	27063/6347/6348/6352/929/4064/948/923/3627/3576/2213/84868/3055/3383/3553/11009/3569/3394/8564/10859/10288/4057/23643/4360/114548/64127/389643/5142/10891/26191/140885/26228/7035/7096/7097/10333/7128/54209/7538	39
BP	GO:0050707	regulation of cytokine secretion	41/926	201/17913	2.72E-14	2.00E-12	1.47E-12	199/9447/301/384/55024/6363/6348/1236/929/914/29126/1436/2213/2219/55612/2633/84868/9734/3553/7850/3556/9173/90865/353514/10859/4023/120892/4321/22861/114548/64127/5027/26191/10125/5552/7096/81793/7097/10333/51311	41

© 2020 Yu Y et al. *JAMA Network Open.*

								/7128	
BP	GO:0042116	macrophage activation	28/926	96/17913	2.87E-14	2.08E-12	1.53E-12	199/712/728/6348/972/2213/2358/84868/9173/133396/90865/3569/3684/3689/3725/120892/5788/6556/6622/26228/7096/7097/10333/51284/51311/54209/7305/11326	28
BP	GO:0070665	positive regulation of leukocyte proliferation	33/926	134/17913	3.25E-14	2.32E-12	1.71E-12	100/199/301/6363/6352/729230/30835/29126/940/952/916/923/972/11151/115352/1880/84868/3109/3113/3115/3481/3594/3553/3559/3569/10288/3071/5588/5788/54440/10673/7412/7535	33
BP	GO:0002822	regulation of adaptive immune response based on somatic recombination of immune receptors built from immunoglobulin superfamily domains	33/926	135/17913	4.10E-14	2.88E-12	2.13E-12	100/301/695/718/6363/729230/29126/940/941/1378/2207/2208/2213/84868/3134/3594/3553/9173/90865/3569/3575/3718/10859/114548/5027/5588/5788/91543/54440/6556/7128/10673/7454	33
BP	GO:0002685	regulation of leukocyte migration	39/926	186/17913	4.54E-14	3.15E-12	2.33E-12	100/84830/199/301/719/728/6363/6347/6364/6366/6348/6351/6352/729230/1236/972/3627/10563/3576/1843/1906/2358/3162/3383/90865/3569/3676/4354/4478/3071/11240/7941/5880/9750/6403/56833/26228/54209/7422	39
BP	GO:0050853	B cell receptor signaling pathway	21/926	53/17913	5.05E-14	3.46E-12	2.55E-12	640/695/930/933/952/973/1493/2213/115352/89857/4332/931/3071/150372/5079/5293/23228/5579/26191/5788/26228	21
BP	GO:1990266	neutrophil migration	24/926	72/17913	7.34E-14	4.96E-12	3.66E-12	719/728/6363/6366/6348/1236/972/1441/1525/3576/1906/3680/3689/120425/4354/3071/5142/5293/5294/5880/9750/6279/56833/7409	24

© 2020 Yu Y et al. *JAMA Network Open.*

BP	GO:0097530	granulocyte migration	28/926	100/17913	8.96E-14	5.91E-12	4.36E-12	30817/301/719/728/6357/6363/6366/6348/6352/1236/972/1441/1525/3576/1906/3680/3689/120425/4354/3071/5142/5293/5294/5880/9750/6279/56833/7409	28
BP	GO:1903555	regulation of tumor necrosis factor superfamily cytokine production	35/926	155/17913	8.97E-14	5.91E-12	4.36E-12	54/384/6363/6348/729230/929/914/29126/948/942/1536/2207/10457/84868/11213/353514/10859/4023/120892/4057/23643/197358/64127/26191/5788/10125/54440/140885/6504/7096/7097/7128/54209/7305/7538	35
BP	GO:0098542	defense response to other organism	67/926	481/17913	1.06E-13	6.88E-12	5.08E-12	54/575/9447/306/384/330/728/6364/948/64581/3627/10563/4283/55601/10875/2358/2633/10578/84868/51191/3428/3429/10964/2537/3434/10410/3594/3553/3559/90865/3569/3662/3394/3512/5650/10859/4057/4069/4321/219972/4599/84166/22861/114548/64127/4939/8638/5027/51316/5551/5788/91543/6279/6403/56833/6556/91607/11005/23166/55576/7097/10333/51284/51311/7128/10346/7728	67
BP	GO:0045089	positive regulation of innate immune response	57/926	373/17913	1.57E-13	1.00E-11	7.41E-12	9447/330/695/6352/929/30835/948/10462/64581/1514/55601/2069/2099/2207/2219/115352/2358/115362/84868/3055/3134/3304/3428/11213/3662/3684/3689/3902/4057/23643/8685/4321/4332/94025/4583/84166/64127/5063/118788/5696/5698/26191/149628/10125/91543/6279/4068/114836/7096/81793/7097/10333/51284/51311/7128/7294/7409	57
BP	GO:0001774	microglial cell activation	20/926	50/17913	1.64E-13	1.02E-11	7.55E-12	199/712/728/6348/2358/90865/3569/3684/3689/3725/120892/5788/6622/26228/7097/10333/51284/51311/54209/7305	20
BP	GO:0002269	leukocyte activation involved in inflammatory response	20/926	50/17913	1.64E-13	1.02E-11	7.55E-12	199/712/728/6348/2358/90865/3569/3684/3689/3725/120892/5788/6622/26228/7097/10333/51284/51311/54209/7305	20
BP	GO:0032623	interleukin-2 production	22/926	62/17913	1.85E-13	1.15E-11	8.45E-12	301/729230/919/940/916/941/942/2207/2633/84868/3553/3556/3662/3902/5142/5588/5788/54440/6556/7128/11326/7538	22

© 2020 Yu Y et al. *JAMA Network Open.*

BP	GO:0002703	regulation of leukocyte mediated immunity	38/926	185/17913	1.91E-13	1.17E-11	8.62E-12	30817/695/718/729230/940/8832/10225/1378/1776/2207/2208/2213/84868/3134/3162/3594/3553/3569/3575/3684/3689/3718/3902/10859/114548/64127/5027/5788/5880/10125/91543/54440/5272/4068/114836/6812/7409/7454	38
BP	GO:0050920	regulation of chemotaxis	38/926	186/17913	2.29E-13	1.38E-11	1.02E-11	199/719/728/6363/6347/6366/6348/6351/6352/729230/1233/1236/972/3627/10563/3576/7852/1843/1906/2152/2358/1880/3603/3569/4354/3071/11240/7941/5800/5880/9750/6091/10371/56833/26228/54209/347733/7422	38
BP	GO:0150076	neuroinflammatory response	24/926	76/17913	2.85E-13	1.69E-11	1.25E-11	107/199/712/728/6348/2358/3553/90865/3569/3684/3689/3725/120892/4318/26471/5788/6622/26228/7097/10333/51284/51311/54209/7305	24
BP	GO:1903706	regulation of hemopoiesis	65/926	468/17913	2.87E-13	1.69E-11	1.25E-11	100/301/695/714/6363/6348/729230/914/939/940/972/941/942/1021/1378/1441/1493/56603/1960/2124/2213/115352/2811/29909/3059/8356/8361/3218/3304/22806/3594/3559/3575/3662/3718/3725/3902/10859/10288/11006/4057/3071/150372/114548/8013/639/5579/5588/5696/5698/5788/10125/54440/56833/6688/11005/9840/55365/28959/8600/9760/54209/7305/7535/7538	65
BP	GO:0071706	tumor necrosis factor superfamily cytokine production	35/926	161/17913	2.98E-13	1.73E-11	1.28E-11	54/384/6363/6348/729230/929/914/29126/948/942/1536/2207/10457/84868/11213/353514/10859/4023/120892/4057/23643/197358/64127/26191/5788/10125/54440/140885/6504/7096/7097/7128/54209/7305/7538	35
BP	GO:0071216	cellular response to biotic stimulus	40/926	206/17913	3.08E-13	1.77E-11	1.31E-11	27063/695/6347/6348/6352/929/4064/948/923/3627/3576/2213/84868/3055/3383/3553/11009/3569/3394/8564/10859/10288/4057/23643/4360/114548/64127/389643/5142/10891/26191/140885/26228/7035/7096/7097/10333/7128/54209/7538	40
BP	GO:0061900	glial cell activation	21/926	59/17913	6.13E-13	3.49E-11	2.58E-11	199/712/728/6348/2358/3553/90865/3569/3684/3689/3725/120892/5788/6622/26228/7097/10333/51284/51311/54209/7305	21
BP	GO:0002285	lymphocyte activation involved in	34/926	158/17913	8.86E-	4.99E-	3.68E-11	100/301/54518/6363/930/911/940/941/942/11151/55619/139818/8320/2213/10875/1880/84868/3109/3134/3383/3594/3569/3662/3683/9452/3718/3936/10859/4063/114548/23228/5	34

© 2020 Yu Y et al. *JAMA Network Open.*

		immune response			13	11		788/114836/6556	
BP	GO:0032680	regulation of tumor necrosis factor production	33/926	151/17913	1.22E-12	6.78E-11	5.00E-11	54/384/6363/6348/729230/929/914/948/1536/2207/10457/84868/11213/353514/10859/4023/120892/4057/23643/197358/64127/26191/5788/10125/54440/140885/6504/7096/7097/7128/54209/7305/7538	33
BP	GO:0002688	regulation of leukocyte chemotaxis	27/926	105/17913	2.33E-12	1.28E-10	9.45E-11	199/719/728/6363/6347/6366/6348/6351/6352/729230/1236/972/3627/10563/3576/1843/1906/2358/3569/4354/3071/11240/7941/5880/9750/56833/26228	27
BP	GO:0032640	tumor necrosis factor production	33/926	155/17913	2.63E-12	1.42E-10	1.05E-10	54/384/6363/6348/729230/929/914/948/1536/2207/10457/84868/11213/353514/10859/4023/120892/4057/23643/197358/64127/26191/5788/10125/54440/140885/6504/7096/7097/7128/54209/7305/7538	33
BP	GO:0001818	negative regulation of cytokine production	47/926	290/17913	2.63E-12	1.42E-10	1.05E-10	54/113/301/384/55024/695/29126/8832/10225/2213/2633/10457/84868/9734/51191/3134/3162/3620/7850/9173/90865/3569/11213/3718/3902/353514/10859/4057/4210/3071/197358/84166/114548/80380/26191/5788/140885/6504/6556/5552/10333/51311/7128/54209/7305/11326/7538	47
BP	GO:0060337	type I interferon signaling pathway	25/926	91/17913	3.05E-12	1.61E-10	1.19E-10	1958/3105/3106/3107/3134/3136/3429/2537/3434/3433/3437/8519/10581/10410/3662/3664/3394/4321/4599/84166/4939/8638/5696/91543/54739	25
BP	GO:0071357	cellular response to type I interferon	25/926	91/17913	3.05E-12	1.61E-10	1.19E-10	1958/3105/3106/3107/3134/3136/3429/2537/3434/3433/3437/8519/10581/10410/3662/3664/3394/4321/4599/84166/4939/8638/5696/91543/54739	25
BP	GO:0045580	regulation of T cell differentiation	31/926	139/17913	3.33E-12	1.74E-10	1.28E-10	100/301/6363/729230/914/939/940/972/941/942/1378/1493/56603/1960/3594/3559/3575/3662/3718/3902/10288/11006/3071/114548/639/5788/10125/54440/11005/9840/7535	31
BP	GO:0042100	B cell proliferation	22/926	71/17913	4.31E-12	2.23E-10	1.64E-10	100/695/930/933/952/972/973/1380/1493/2213/115352/1880/22806/3575/4332/931/51237/3071/23228/5788/10673/7305	22

© 2020 Yu Y et al. *JAMA Network Open.*

BP	GO:0071222	cellular response to lipopolysaccharide	35/926	176/17913	4.60E-12	2.36E-10	1.74E-10	27063/6347/6348/6352/929/4064/948/923/3627/3576/84868/3055/3383/3553/11009/3569/3394/8564/10859/10288/4057/23643/4360/114548/64127/389643/5142/10891/26191/140885/26228/7035/7097/7128/7538	35
BP	GO:0032612	interleukin-1 production	27/926	108/17913	4.79E-12	2.43E-10	1.79E-10	54/9447/301/384/6363/6348/1236/948/1958/115362/84868/3428/3553/7850/353514/4023/4210/22861/114548/64127/5027/140885/10333/51311/7128/54209/7305	27
BP	GO:0072678	T cell migration	20/926	59/17913	6.22E-12	3.12E-10	2.31E-10	199/6347/6364/6366/6348/6352/729230/3627/6373/10563/2838/1880/3383/3676/4478/64005/5293/5294/9750/7535	20
BP	GO:0048002	antigen processing and presentation of peptide antigen	36/926	188/17913	7.27E-12	3.61E-10	2.67E-10	30835/948/972/10332/1514/1536/64167/2209/2213/3105/3106/3107/3108/3109/3112/3113/3115/3118/3119/3120/3122/3123/3125/3134/3136/10437/3902/653361/4688/4689/5696/5698/6556/6890/6891/54209	36
BP	GO:0050777	negative regulation of immune response	31/926	143/17913	7.34E-12	3.61E-10	2.67E-10	301/384/729230/8832/10225/1378/1493/2213/127943/84868/3134/3162/3428/9173/3559/90865/3575/11213/3718/10859/4321/197358/84166/5788/64092/5272/710/56833/11005/7128/11326	31
BP	GO:0034340	response to type I interferon	25/926	95/17913	8.68E-12	4.23E-10	3.12E-10	1958/3105/3106/3107/3134/3136/3429/2537/3434/3433/3437/8519/10581/10410/3662/3664/3394/4321/4599/84166/4939/8638/5696/91543/54739	25
BP	GO:0050852	T cell receptor signaling pathway	35/926	180/17913	9.03E-12	4.36E-10	3.22E-10	100/1236/919/915/916/917/2533/2633/3113/3115/3118/3119/3120/3122/3123/3125/3702/3932/3937/11006/5063/5142/5293/5588/5696/5698/26191/5788/9840/387357/9473/50852/7294/7454/7535	35
BP	GO:0071887	leukocyte apoptotic process	26/926	103/17913	9.38E-12	4.49E-10	3.31E-10	100/384/695/6363/6366/6352/1236/939/29126/917/972/1514/2207/3059/3620/3559/3569/3575/3718/10859/64127/8013/5027/5293/5588/1831	26
BP	GO:0002286	T cell activation	24/926	88/17913	9.85	4.67	3.44E-10	301/54518/6363/911/941/942/8320/2213/10875/1880/84868/3109/3383/3594/3569/3662/3683/3718/3936/10859/4063/11	24

© 2020 Yu Y et al. *JAMA Network Open.*

		involved in immune response			E-12	E-10		4548/114836/6556	
BP	GO:0009615	response to virus	49/926	323/17913	1.08E-11	5.07E-10	3.74E-10	9447/330/6363/6351/6352/1021/3627/4283/7852/9201/55601/10875/2633/51191/8091/3428/3429/10561/10964/2537/3434/3433/3437/8519/10581/10410/3594/3553/3559/90865/3569/11213/10859/4321/4599/84166/114548/4939/8638/11040/5551/5788/91543/91607/51284/51311/7128/10346/7728	49
BP	GO:0002791	regulation of peptide secretion	63/926	483/17913	1.10E-11	5.11E-10	3.77E-10	150/84830/199/9447/27063/301/8862/348/384/8938/55024/640/777/6363/6348/6352/1236/929/914/933/29126/952/972/1436/1803/2213/2219/55612/2633/84868/9734/3553/7850/3556/9173/90865/3569/353514/10859/4023/120892/4321/22861/114548/64127/5027/26191/10125/91543/6279/6513/80725/5552/7096/81793/7097/10333/51311/7128/8600/54209/7273/7447	63
BP	GO:0002440	production of molecular mediator of immune response	36/926	191/17913	1.18E-11	5.42E-10	4.00E-10	695/729230/933/940/948/972/10225/2207/2213/115352/10875/51704/3119/3134/3162/3553/90865/3569/3575/11213/9452/3718/5650/54900/10859/51237/114548/64127/8013/5788/91543/54440/6556/11005/7097/10673	36
BP	GO:0002577	regulation of antigen processing and presentation	12/926	19/17913	1.22E-11	5.59E-10	4.12E-10	6363/6366/1236/972/2213/10875/3112/10288/64127/6556/54209/7454	12
BP	GO:2000106	regulation of leukocyte apoptotic process	23/926	82/17913	1.41E-11	6.38E-10	4.71E-10	100/384/695/6363/6366/6352/1236/939/29126/917/972/2207/3059/3620/3575/3718/10859/64127/8013/5027/5293/5588/1831	23
BP	GO:0050731	positive regulation of peptidyl-tyrosine phosphorylation	35/926	184/17913	1.73E-11	7.78E-10	5.74E-10	150/389658/374/55024/6352/948/916/960/972/941/1436/5168/2069/2209/2261/2322/51704/1839/3059/3383/3481/11009/133396/3569/353514/4038/653361/3084/5788/5649/9021/80725/26228/54209/7422	35
B	GO:00	positive	20/92	62/17913	1.	7.	5.80E-10	729230/929/914/916/84868/3113/3115/3594/3595/3553/339	2

P	32729	regulation of interferon-gamma production	6		77E-11	86E-10		4/5142/26191/10125/54440/114836/6556/51284/51311/7294	0
BP	GO:0042108	positive regulation of cytokine biosynthetic process	20/926	63/17913	2.47E-11	1.09E-09	8.02E-10	729230/940/916/941/942/1536/1958/2069/3162/3553/3569/3662/4050/5588/5788/7096/10333/51284/51311/7305	20
BP	GO:0046631	alpha-beta T cell activation	29/926	133/17913	3.00E-11	1.31E-09	9.67E-10	100/301/384/6363/729230/29126/940/916/941/942/1794/8320/1880/3594/3569/3662/3718/10859/4063/3071/114548/26279/639/5588/5788/91543/54440/114836/7535	29
BP	GO:0032611	interleukin-1 beta production	24/926	93/17913	3.56E-11	1.54E-09	1.14E-09	54/9447/384/6363/6348/1236/948/1958/115362/3428/3553/353514/4023/4210/22861/114548/64127/5027/140885/10333/51311/7128/54209/7305	24
BP	GO:0043410	positive regulation of MAPK cascade	63/926	499/17913	4.47E-11	1.92E-09	1.42E-09	150/389658/348/55024/728/6363/6366/6348/1236/939/948/960/972/50937/1116/1436/7852/1906/10595/2213/115352/2261/2322/2357/2358/64762/2651/10457/1880/84868/3383/3481/3553/3569/3725/353514/23566/120892/11184/8685/653361/64127/3084/5027/5031/5063/5294/23533/26191/5788/10125/5979/6091/388325/10371/6504/6899/10333/8600/23043/54209/7422/9839	63
BP	GO:0050864	regulation of B cell activation	26/926	110/17913	4.66E-11	1.99E-09	1.47E-09	100/55024/695/930/933/939/940/952/972/1493/2213/115352/1880/22806/3569/4332/51237/3071/150372/64127/5788/64092/56833/7128/10673/7305	26
BP	GO:0002526	acute inflammatory response	38/926	220/17913	4.85E-11	2.05E-09	1.51E-09	695/712/713/714/717/718/719/720/721/728/730/1236/9332/930/923/629/3426/1378/1380/1776/2152/2207/2213/3383/3553/3569/114548/26471/9180/5294/6279/5265/710/55061/8600/54210/7412/11326	38
BP	GO:2000107	negative regulation of leukocyte apoptotic	17/926	46/17913	4.93E-11	2.06E-09	1.52E-09	100/384/6363/6366/6352/1236/939/972/2207/3059/3620/3575/3718/10859/64127/5588/1831	17

© 2020 Yu Y et al. *JAMA Network Open.*

		process							
BP	GO:0045123	cellular extravasation	19/926	59/17913	5.86E-11	2.44E-09	1.80E-09	119/6347/6366/729230/2650/3383/3676/3689/120425/5293/5294/50512/9750/6401/6402/6403/6404/140885/7412	19
BP	GO:0006911	phagocytosis, engulfment	16/926	41/17913	6.97E-11	2.87E-09	2.12E-09	575/199/51411/718/948/9844/2207/2209/2213/3684/3689/8685/4481/3071/26228/54209	16
BP	GO:0050708	regulation of protein secretion	59/926	456/17913	7.04E-11	2.88E-09	2.12E-09	150/84830/199/9447/27063/301/348/384/8938/55024/640/777/6363/6348/6352/1236/929/914/933/29126/952/1436/1803/2213/2219/55612/2633/84868/9734/3553/7850/3556/9173/90865/3569/353514/10859/4023/120892/4321/22861/114548/64127/5027/26191/10125/91543/6513/80725/5552/7096/81793/7097/10333/51311/7128/54209/7273/7447	59
BP	GO:0002687	positive regulation of leukocyte migration	27/926	121/17913	8.20E-11	3.32E-09	2.45E-09	199/719/728/6363/6364/6366/6348/6351/6352/729230/1236/972/3627/10563/3576/1906/2358/3383/3569/3676/3071/7941/5880/9750/6403/54209/7422	27
BP	GO:0002824	positive regulation of adaptive immune response based on somatic recombination of immune receptors built from immunoglobulin superfamily domains	23/926	89/17913	8.83E-11	3.55E-09	2.62E-09	100/301/695/718/6363/729230/29126/940/941/2207/2208/3134/3594/3553/3569/114548/5027/5588/5788/91543/54440/6556/10673	23
BP	GO:0050921	positive regulation of	28/926	130/17913	8.92	3.56	2.63E-09	199/719/728/6363/6366/6348/6351/6352/729230/1233/1236/972/3627/10563/3576/7852/1906/2152/2358/3603/3569/307	28

		chemotaxis			E-11	E-09		1/7941/5880/9750/54209/347733/7422	
BP	GO:0032663	regulation of interleukin-2 production	18/926	54/17913	9.80E-11	3.88E-09	2.86E-09	301/729230/940/916/941/942/2633/84868/3553/3662/3902/5142/5588/5788/54440/7128/11326/7538	18
BP	GO:0050715	positive regulation of cytokine secretion	28/926	132/17913	1.30E-10	5.13E-09	3.78E-09	199/9447/6363/6348/929/914/29126/1436/2219/55612/84868/3553/3556/9173/90865/353514/4023/120892/4321/22861/114548/64127/5027/26191/10125/7096/7097/51311	28
BP	GO:0001776	leukocyte homeostasis	22/926	84/17913	1.71E-10	6.65E-09	4.91E-09	100/301/729230/972/11151/55619/139818/2207/2322/84636/1880/3559/3569/3718/3071/5027/5142/5293/124976/7128/10673/1831	22
BP	GO:0050854	regulation of antigen receptor-mediated signaling pathway	19/926	63/17913	2.11E-10	8.18E-09	6.04E-09	100/640/1236/930/933/2213/115352/2633/3932/11006/150372/5079/23228/5579/26191/5788/26228/9840/50852	19
BP	GO:0043062	extracellular structure organization	52/926	387/17913	2.49E-10	9.52E-09	7.02E-09	8728/56999/54507/84830/341/348/655/146206/948/960/999/1071/1310/1285/1286/1297/55790/1514/1803/2201/55612/3383/3569/3675/3676/3680/3683/3684/3687/3689/3691/3696/5650/284217/3909/3914/3936/4018/4023/4321/4316/4318/4868/7941/5806/5268/6662/11005/9806/6696/4982/7412	52
BP	GO:0002695	negative regulation of leukocyte activation	31/926	163/17913	2.50E-10	9.52E-09	7.02E-09	301/384/55024/695/729230/29126/972/8832/1493/2213/10457/84868/3134/3162/3620/3559/133396/3718/3902/54900/10859/10288/4332/80380/26191/5788/9750/64092/7128/7305/11326	31
BP	GO:0002706	regulation of lymphocyte mediated immunity	28/926	136/17913	2.72E-10	1.03E-08	7.60E-09	695/718/729230/940/10225/1378/2207/2208/2213/84868/3134/3594/3553/3569/3575/3902/10859/114548/5027/5788/10125/91543/54440/5272/4068/114836/7409/7454	28
BP	GO:0001910	regulation of leukocyte mediated	19/926	64/17913	2.86E-	1.07E-	7.93E-09	1776/2213/84868/3134/3383/3594/3575/3684/3902/10859/5027/5788/10125/5272/4068/114836/26228/7305/7409	19

© 2020 Yu Y et al. *JAMA Network Open.*

		cytotoxicity			10	08			
BP	GO:0002821	positive regulation of adaptive immune response	23/926	94/17913	2.91E-10	1.08E-08	8.00E-09	100/301/695/718/6363/729230/29126/940/941/2207/2208/3134/3594/3553/3569/114548/5027/5588/5788/91543/54440/6556/10673	23
BP	GO:0002700	regulation of production of molecular mediator of immune response	28/926	138/17913	3.89E-10	1.44E-08	1.06E-08	695/729230/933/940/948/972/10225/2207/2213/115352/51704/3134/3162/3553/90865/3569/11213/3718/5650/10859/51237/114548/64127/8013/5788/91543/54440/11005	28
BP	GO:0018108	peptidyl-tyrosine phosphorylation	48/926	346/17913	4.14E-10	1.52E-08	1.12E-08	150/389658/374/55024/640/695/6352/948/916/960/972/941/1436/5168/2048/2069/2209/2261/2322/51704/1839/3055/3059/3383/3481/3595/11009/133396/3569/3689/3718/3932/353514/4038/653361/3084/5788/5649/5979/6098/64092/9021/80725/26228/54209/7273/7422/7535	48
BP	GO:0032652	regulation of interleukin-1 production	23/926	96/17913	4.57E-10	1.67E-08	1.23E-08	54/9447/301/384/6363/6348/1236/1958/84868/3428/7850/353514/4023/4210/22861/114548/64127/5027/140885/51311/7128/54209/7305	23
BP	GO:0032675	regulation of interleukin-6 production	28/926	139/17913	4.63E-10	1.68E-08	1.24E-08	199/55024/948/2069/2207/84868/3553/3556/90865/3569/11213/353514/10288/4023/3071/197358/64127/5027/26191/140885/6504/7096/7097/10333/51311/7128/54209/7305	28
BP	GO:0030183	B cell differentiation	25/926	113/17913	4.97E-10	1.79E-08	1.32E-08	100/222487/695/930/939/973/1380/55619/139818/2213/115352/2322/1880/9734/22806/3676/9452/3718/931/3071/150372/23228/5788/56833/7412	25
BP	GO:0050866	negative regulation of cell activation	33/926	187/17913	5.13E-10	1.82E-08	1.35E-08	301/348/384/55024/695/729230/29126/972/8832/1493/2213/2729/10457/84868/3134/3162/3620/3559/133396/3718/3902/54900/10859/10288/4332/80380/26191/5788/9750/64092/7128/7305/11326	33
BP	GO:0043030	regulation of macrophage activation	19/926	66/17913	5.15E-	1.82E-	1.35E-08	712/728/6348/972/2213/84868/9173/133396/90865/3569/3684/3689/120892/5788/6622/26228/10333/54209/11326	19

© 2020 Yu Y et al. *JAMA Network Open.*

					10	08			
BP	GO:0019932	second-messenger-mediated signaling	49/926	360/17913	5.28E-10	1.86E-08	1.37E-08	100/107/113/150/348/695/6364/6348/6351/933/948/916/3627/6373/3576/4283/7852/1906/2357/2358/2633/8477/27201/2903/2904/120892/79772/4502/9722/3084/5027/5031/10846/5140/5341/5788/51655/1827/5997/6401/6403/6662/8600/50852/54209/7253/7412/7422/7535	49
BP	GO:0002690	positive regulation of leukocyte chemotaxis	21/926	81/17913	5.44E-10	1.89E-08	1.39E-08	199/719/728/6363/6366/6348/6351/6352/729230/1236/972/3627/10563/3576/1906/2358/3569/3071/7941/5880/9750	21
BP	GO:1903557	positive regulation of tumor necrosis factor superfamily cytokine production	21/926	81/17913	5.44E-10	1.89E-08	1.39E-08	6363/6348/729230/929/914/948/942/1536/2207/84868/353514/4023/120892/23643/64127/5788/10125/54440/7096/7097/7305	21
BP	GO:0018212	peptidyl-tyrosine modification	48/926	349/17913	5.57E-10	1.92E-08	1.42E-08	150/389658/374/55024/640/695/6352/948/916/960/972/941/1436/5168/2048/2069/2209/2261/2322/51704/1839/3055/3059/3383/3481/3595/11009/133396/3569/3689/3718/3932/353514/4038/653361/3084/5788/5649/5979/6098/64092/9021/80725/26228/54209/7273/7422/7535	48
BP	GO:0001909	leukocyte mediated cytotoxicity	23/926	97/17913	5.70E-10	1.95E-08	1.44E-08	11151/1776/2213/3002/84868/3134/3383/3594/3575/3684/3902/10859/5027/5551/5788/10125/5272/4068/114836/57823/26228/7305/7409	23
BP	GO:0006968	cellular defense response	17/926	53/17913	6.46E-10	2.20E-08	1.62E-08	728/729230/4283/9214/10578/3702/3822/10288/4046/23643/4332/653361/4688/5551/4068/50852/7305	17
BP	GO:0031341	regulation of cell killing	21/926	82/17913	6.96E-10	2.35E-08	1.74E-08	1776/2208/2213/84868/3134/3383/3594/3575/3684/3902/10859/5027/5551/5788/10125/5272/4068/114836/26228/7305/7409	21
B	GO:19	positive	28/92	143/1791	9.	3.	2.27E-08	100/301/695/6363/939/972/941/942/1960/2124/3059/3594/3	2

© 2020 Yu Y et al. *JAMA Network Open.*

P	02107	regulation of leukocyte differentiation	6	3	17 E-10	08 E-08		559/3575/3725/10288/11006/3071/114548/5788/10125/54440/9840/8600/9760/54209/7305/7535	8
BP	GO:0034113	heterotypic cell-cell adhesion	18/926	61/17913	9.41 E-10	3.14 E-08	2.32E-08	655/914/960/1525/1824/1829/2651/3553/3676/3687/3689/3932/10288/4897/64065/5788/140885/7412	18
BP	GO:0002920	regulation of humoral immune response	27/926	135/17913	1.12 E-09	3.70 E-08	2.73E-08	712/713/714/717/718/719/720/721/728/730/1236/930/629/3426/1378/1380/10563/2208/2213/3553/5650/5788/710/11005/124976/55061/11326	27
BP	GO:0051897	positive regulation of protein kinase B signaling	31/926	173/17913	1.16 E-09	3.82 E-08	2.82E-08	374/6363/6366/6348/1236/930/940/941/942/1116/2069/2099/2152/2261/2651/1839/3059/29851/3481/3932/3084/145957/118788/5293/5294/23533/5880/5979/8600/50852/7409	31
BP	GO:0002478	antigen processing and presentation of exogenous peptide antigen	31/926	174/17913	1.34 E-09	4.40 E-08	3.25E-08	948/972/1514/1536/2209/2213/3105/3106/3107/3108/3109/3112/3113/3115/3118/3119/3120/3122/3123/3125/3134/3136/10437/3902/653361/4688/4689/5696/5698/6890/6891	31
BP	GO:0052548	regulation of endopeptidase activity	49/926	370/17913	1.36 E-09	4.40 E-08	3.25E-08	9447/653145/274/330/718/720/721/135228/939/960/340267/1285/1378/1476/26999/1800/112399/2152/355/2903/2904/3428/2537/27074/3932/4018/4057/4210/4318/4804/22861/114548/89932/64065/5266/5329/5696/5698/6091/6279/5272/6622/11005/9806/50859/7035/7980/8626/7422	49
BP	GO:0045621	positive regulation of lymphocyte differentiation	22/926	93/17913	1.41 E-09	4.56 E-08	3.37E-08	100/301/695/6363/939/972/941/942/1960/3594/3559/3575/10288/11006/3071/114548/5788/10125/54440/9840/9760/7535	22
B	GO:00	regulation of	58/92	480/1791	1.	4.	3.46E-08	107/30817/150/301/321/8862/341/348/8938/274/717/718/72	5

P	60627	vesicle-mediated transport	6	3	46E-09	69E-08		0/721/6363/6347/6366/729230/929/933/948/8832/11151/1601/9829/1794/2207/2213/2358/3055/3134/3162/3553/3560/3561/3684/3689/10859/120892/79772/4478/3071/5579/5788/5806/5874/5880/6401/140885/6556/9892/6622/26228/85439/6812/7097/7422/7447	8
BP	GO:0042035	regulation of cytokine biosynthetic process	24/926	110/17913	1.49E-09	4.74E-08	3.50E-08	729230/940/916/941/942/1536/1958/2069/3162/10643/3553/3569/3662/3902/10859/4050/5588/5788/7096/10333/51284/51311/7305/7538	24
BP	GO:0001906	cell killing	29/926	156/17913	1.64E-09	5.21E-08	3.85E-08	718/6357/11151/1776/2208/2213/10578/3002/84868/3134/3383/3594/3575/3684/3902/10859/4057/4069/5027/5551/5788/10125/5272/4068/114836/57823/26228/7305/7409	29
BP	GO:0032760	positive regulation of tumor necrosis factor production	20/926	78/17913	1.73E-09	5.45E-08	4.02E-08	6363/6348/729230/929/914/948/1536/2207/84868/353514/4023/120892/23643/64127/5788/10125/54440/7096/7097/7305	20
BP	GO:0019884	antigen processing and presentation of exogenous antigen	31/926	176/17913	1.80E-09	5.62E-08	4.15E-08	948/972/1514/1536/2209/2213/3105/3106/3107/3108/3109/3112/3113/3115/3118/3119/3120/3122/3123/3125/3134/3136/10437/3902/653361/4688/4689/5696/5698/6890/6891	31
BP	GO:0042089	cytokine biosynthetic process	25/926	120/17913	1.89E-09	5.88E-08	4.34E-08	729230/940/916/941/942/1536/1958/2069/3162/10643/3553/3556/3569/3662/3902/10859/4050/5588/5788/7096/10333/51284/51311/7305/7538	25
BP	GO:0070372	regulation of ERK1 and ERK2 cascade	40/926	271/17913	1.97E-09	6.07E-08	4.48E-08	389658/348/467/728/6363/6366/6348/1236/948/960/972/1116/1436/1843/2048/2261/2358/64762/2633/2651/10457/1880/84868/3383/3553/3725/8685/64127/5031/26191/5788/10125/6098/27330/388325/57556/140885/6504/8600/54209	40
BP	GO:0052547	regulation of peptidase	51/926	398/17913	1.98	6.07	4.48E-08	9447/653145/274/330/718/720/721/135228/939/960/340267/1285/1378/1476/26999/1800/112399/2152/355/2903/2904/3	51

© 2020 Yu Y et al. *JAMA Network Open.*

		activity			E-09	E-08		428/2537/27074/3932/4018/4057/4210/4318/4804/22861/114548/89932/26577/64065/51050/5266/5329/5696/5698/6091/6279/5272/6622/11005/9806/50859/7035/7980/8626/7422	
BP	GO:0099024	plasma membrane invagination	16/926	50/17913	2.14E-09	6.55E-08	4.83E-08	575/199/51411/718/948/9844/2207/2209/2213/3684/3689/8685/4481/3071/26228/54209	16
BP	GO:0042107	cytokine metabolic process	25/926	121/17913	2.27E-09	6.89E-08	5.08E-08	729230/940/916/941/942/1536/1958/2069/3162/10643/3553/3556/3569/3662/3902/10859/4050/5588/5788/7096/10333/51284/51311/7305/7538	25
BP	GO:0032635	interleukin-6 production	28/926	149/17913	2.43E-09	7.34E-08	5.42E-08	199/55024/948/2069/2207/84868/3553/3556/90865/3569/11213/353514/10288/4023/3071/197358/64127/5027/26191/140885/6504/7096/7097/10333/51311/7128/54209/7305	28
BP	GO:0048872	homeostasis of number of cells	37/926	241/17913	2.66E-09	7.97E-08	5.88E-08	100/10000/301/729230/1233/1236/972/1021/11151/55619/139818/2207/2322/84636/1880/3059/3162/3216/3304/10320/3559/3569/3575/3718/3071/5027/5142/5293/54440/6662/6688/124976/7128/10673/1831/7422/7538	37
BP	GO:0002218	activation of innate immune response	44/926	319/17913	2.68E-09	7.99E-08	5.90E-08	9447/330/695/929/30835/948/10462/64581/1514/55601/2099/2207/2219/115352/84868/3055/3304/3428/11213/3662/3684/3689/4057/23643/8685/4332/94025/4583/64127/5063/118788/5696/5698/26191/149628/91543/6279/7096/81793/7097/10333/51284/51311/7128	44
BP	GO:0042094	interleukin-2 biosynthetic process	11/926	22/17913	2.77E-09	8.22E-08	6.07E-08	940/916/941/942/3553/3556/3662/3902/5588/5788/7538	11
BP	GO:0050702	interleukin-1 beta secretion	16/926	51/17913	2.98E-09	8.77E-08	6.47E-08	9447/384/6363/6348/1236/948/115362/353514/4023/22861/114548/64127/5027/10333/51311/7128	16
BP	GO:0002673	regulation of acute inflammatory response	29/926	160/17913	3.03E-09	8.87E-08	6.55E-08	695/712/713/714/717/718/719/720/721/728/730/1236/930/629/3426/1378/1380/1776/2207/2213/3553/3569/114548/9180/5294/710/55061/8600/11326	29

© 2020 Yu Y et al. *JAMA Network Open.*

BP	GO:0045807	positive regulation of endocytosis	27/926	141/17913	3.05E-09	8.89E-08	6.56E-08	8862/348/717/718/720/721/6363/6347/6366/929/948/1601/1794/2213/2358/3553/3560/3561/120892/3071/5788/5806/6401/6556/6622/26228/7422	27
BP	GO:0051250	negative regulation of lymphocyte activation	26/926	132/17913	3.19E-09	9.25E-08	6.82E-08	301/384/55024/695/29126/972/1493/2213/10457/84868/3134/3620/3559/3718/3902/54900/10859/10288/4332/80380/26191/9750/64092/7128/7305/11326	26
BP	GO:0070371	ERK1 and ERK2 cascade	41/926	289/17913	4.07E-09	1.17E-07	8.66E-08	389658/348/467/728/6363/6366/6348/1236/948/960/972/1116/1436/1843/2048/2261/2358/64762/2633/2651/10457/1880/84868/3383/3553/3725/8685/64127/5031/26191/5788/10125/6098/27330/388325/57556/140885/6504/6662/8600/54209	41
BP	GO:0050730	regulation of peptidyl-tyrosine phosphorylation	37/926	245/17913	4.21E-09	1.21E-07	8.90E-08	150/389658/374/55024/6352/948/916/960/972/941/1436/5168/2069/2209/2261/2322/51704/1839/3059/3383/3481/11009/133396/3569/3689/353514/4038/653361/3084/5788/5649/64092/9021/80725/26228/54209/7422	37
BP	GO:0046634	regulation of alpha-beta T cell activation	21/926	90/17913	4.35E-09	1.24E-07	9.11E-08	100/301/384/6363/729230/29126/940/916/941/942/3594/3662/3718/10859/3071/114548/639/5588/5788/54440/7535	21
BP	GO:0032651	regulation of interleukin-1 beta production	20/926	82/17913	4.46E-09	1.24E-07	9.11E-08	54/9447/384/6363/6348/1236/1958/3428/353514/4023/4210/22861/114548/64127/5027/140885/51311/7128/54209/7305	20
BP	GO:0030260	entry into host cell	26/926	134/17913	4.46E-09	1.24E-07	9.11E-08	290/30835/972/941/942/999/10332/1378/1380/1525/3576/7852/1803/2219/2838/3304/3383/8519/10581/10410/4360/5818/5806/6404/6504/11074	26
BP	GO:0044409	entry into host	26/926	134/17913	4.46E-09	1.24E-07	9.11E-08	290/30835/972/941/942/999/10332/1378/1380/1525/3576/7852/1803/2219/2838/3304/3383/8519/10581/10410/4360/5818/5806/6404/6504/11074	26
BP	GO:0051806	entry into cell of other organism	26/926	134/17913	4.46E-	1.24E-	9.11E-08	290/30835/972/941/942/999/10332/1378/1380/1525/3576/7852/1803/2219/2838/3304/3383/8519/10581/10410/4360/5818/5806/6404/6504/11074	26

© 2020 Yu Y et al. *JAMA Network Open.*

		involved in symbiotic interaction			09	07			
BP	GO:0051828	entry into other organism involved in symbiotic interaction	26/926	134/17913	4.46E-09	1.24E-07	9.11E-08	290/30835/972/941/942/999/10332/1378/1380/1525/3576/7852/1803/2219/2838/3304/3383/8519/10581/10410/4360/5818/5806/6404/6504/11074	26
BP	GO:0002698	negative regulation of immune effector process	24/926	116/17913	4.60E-09	1.27E-07	9.36E-08	301/729230/933/8832/10225/1378/2213/115352/10875/84868/3134/3162/3559/90865/3575/11213/3718/10859/5788/5272/710/56833/11005/11326	24
BP	GO:0050701	interleukin-1 secretion	17/926	60/17913	5.49E-09	1.48E-07	1.09E-07	9447/384/6363/6348/1236/948/115362/7850/353514/4023/22861/114548/64127/5027/10333/51311/7128	17
BP	GO:0050766	positive regulation of phagocytosis	17/926	60/17913	5.49E-09	1.48E-07	1.09E-07	717/718/720/721/6347/948/1794/2213/2358/3553/3560/3561/3071/5788/5806/6556/26228	17
BP	GO:1990868	response to chemokine	17/926	60/17913	5.49E-09	1.48E-07	1.09E-07	2532/6366/6352/729230/1233/1236/3627/6373/3577/7852/10663/1843/1906/11240/9750/6091/54209	17
BP	GO:1990869	cellular response to chemokine	17/926	60/17913	5.49E-09	1.48E-07	1.09E-07	2532/6366/6352/729230/1233/1236/3627/6373/3577/7852/10663/1843/1906/11240/9750/6091/54209	17
BP	GO:0045582	positive regulation of T cell differentiation	20/926	83/17913	5.59E-09	1.50E-07	1.11E-07	100/301/6363/939/972/941/942/1960/3594/3559/3575/10288/11006/3071/114548/5788/10125/54440/9840/7535	20
B	GO:00	negative	35/92	226/1791	5.	1.	1.11E-07	100/150/84830/301/348/384/55024/729230/933/972/8832/19	3

© 2020 Yu Y et al. *JAMA Network Open.*

P	51048	regulation of secretion	6	3	66 E-09	51 E-07		06/2213/2633/9734/3134/3162/3553/7850/90865/353514/10859/4129/114548/3084/55607/26191/91543/6622/80725/5552/10333/51311/7128/7447	5
BP	GO:1902622	regulation of neutrophil migration	13/926	34/17913	5.83 E-09	1.55 E-07	1.14E-07	719/728/6363/6366/1236/972/3576/1906/4354/3071/5880/9750/56833	13
BP	GO:0071674	mononuclear cell migration	18/926	68/17913	6.48 E-09	1.71 E-07	1.26E-07	199/301/719/728/6347/6348/6352/729230/3627/1843/2358/3569/120425/7941/5800/140885/56833/8600	18
BP	GO:0002604	regulation of dendritic cell antigen processing and presentation	8/926	11/17913	7.11 E-09	1.85 E-07	1.37E-07	6363/6366/1236/972/2213/10875/64127/6556	8
BP	GO:0043383	negative T cell selection	8/926	11/17913	7.11 E-09	1.85 E-07	1.37E-07	1236/940/916/972/1794/5788/387357/7535	8
BP	GO:0070374	positive regulation of ERK1 and ERK2 cascade	31/926	186/17913	7.12 E-09	1.85 E-07	1.37E-07	389658/348/728/6363/6366/6348/1236/948/960/972/1116/1436/2261/2358/64762/2651/10457/1880/84868/3383/3725/8685/64127/5031/26191/5788/10125/388325/6504/8600/54209	31
BP	GO:0045076	regulation of interleukin-2 biosynthetic process	10/926	19/17913	7.83 E-09	2.03 E-07	1.50E-07	940/916/941/942/3553/3662/3902/5588/5788/7538	10
BP	GO:0030100	regulation of endocytosis	38/926	262/17913	8.16 E-09	2.10 E-07	1.55E-07	8862/341/348/274/717/718/720/721/6363/6347/6366/929/933/948/1601/9829/1794/2213/2358/3055/3553/3560/3561/10859/120892/79772/3071/5788/5806/6401/140885/6556/9892/6622/26228/85439/7097/7422	38
BP	GO:0090023	positive regulation of	11/926	24/17913	8.92	2.27	1.68E-07	719/728/6363/6366/1236/972/3576/1906/3071/5880/9750	11

© 2020 Yu Y et al. *JAMA Network Open.*

		neutrophil chemotaxis			E-09	E-07			
BP	GO:0150078	positive regulation of neuroinflammatory response	11/926	24/17913	8.92E-09	2.27E-07	1.68E-07	712/6348/3553/90865/3569/3684/3689/120892/26471/26228/54209	11
BP	GO:0002793	positive regulation of peptide secretion	39/926	275/17913	9.84E-09	2.49E-07	1.84E-07	199/9447/27063/8862/8938/640/6363/6348/929/914/29126/952/1436/2219/55612/84868/3553/3556/9173/90865/3569/353514/4023/120892/4321/22861/114548/64127/5027/26191/10125/6279/7096/7097/51311/8600/54209/7273/7447	39
BP	GO:0032653	regulation of interleukin-10 production	15/926	48/17913	9.87E-09	2.49E-07	1.84E-07	29126/940/2207/2213/3620/3662/3718/353514/10859/64127/80380/54440/7097/54209/7305	15
BP	GO:0051607	defense response to virus	35/926	231/17913	1.01E-08	2.53E-07	1.87E-07	9447/330/3627/4283/55601/10875/2633/51191/3428/3429/10964/2537/3434/10410/3594/3553/3559/90865/3569/10859/4321/4599/84166/114548/4939/8638/5551/5788/91543/91607/51284/51311/7128/10346/7728	35
BP	GO:0031295	T cell costimulation	16/926	55/17913	1.01E-08	2.53E-07	1.87E-07	6363/6366/1236/29126/940/916/941/942/1493/1803/29851/3932/5063/80380/10673/7409	16
BP	GO:1903532	positive regulation of secretion by cell	48/926	381/17913	1.03E-08	2.56E-07	1.89E-07	199/9447/27063/8862/8938/640/6363/6348/929/914/29126/952/1436/1906/2207/2219/55612/84868/3134/3553/3556/9173/90865/3569/3684/3689/8564/353514/4023/120892/4321/22861/114548/64127/5027/26191/5874/10125/6622/6696/6812/7096/7097/51311/8600/54209/7273/7447	48
BP	GO:0046718	viral entry into host cell	24/926	121/17913	1.11E-08	2.73E-07	2.02E-07	290/30835/972/941/942/10332/1378/1380/1525/7852/1803/2219/2838/3304/3383/8519/10581/10410/4360/5818/5806/6404/6504/11074	24
BP	GO:0030198	extracellular matrix organization	44/926	334/17913	1.11E-08	2.73E-07	2.02E-07	8728/56999/54507/84830/146206/960/999/1310/1285/1286/1297/55790/1514/1803/2201/55612/3383/3569/3675/3676/3680/3683/3684/3687/3689/3691/3696/5650/284217/3909/3914/3936/4321/4316/4318/4868/5806/5268/6662/11005/9806/	44

© 2020 Yu Y et al. *JAMA Network Open.*

								6696/4982/7412	
BP	GO:0048247	lymphocyte chemotaxis	14/926	42/17913	1.20E-08	2.94E-07	2.17E-07	6347/6366/6348/6351/6352/729230/9023/3627/6373/10563/1880/11240/5293/5294	14
BP	GO:0032743	positive regulation of interleukin-2 production	12/926	30/17913	1.24E-08	3.00E-07	2.21E-07	301/729230/940/916/941/942/3553/3662/5142/5588/5788/54440	12
BP	GO:0090022	regulation of neutrophil chemotaxis	12/926	30/17913	1.24E-08	3.00E-07	2.21E-07	719/728/6363/6366/1236/972/3576/1906/4354/3071/5880/9750	12
BP	GO:0031294	lymphocyte costimulation	16/926	56/17913	1.35E-08	3.27E-07	2.41E-07	6363/6366/1236/29126/940/916/941/942/1493/1803/29851/3932/5063/80380/10673/7409	16
BP	GO:0051047	positive regulation of secretion	50/926	409/17913	1.38E-08	3.31E-07	2.45E-07	199/9447/27063/8862/8938/640/6363/6348/929/914/29126/952/1436/1906/2207/2219/55612/84868/3134/3553/3556/9173/90865/3569/3684/3689/8564/353514/4023/120892/4321/22861/114548/64127/5027/5029/26191/5874/10125/6279/6622/6696/6812/7096/7097/51311/8600/54209/7273/7447	50
BP	GO:1903531	negative regulation of secretion by cell	32/926	202/17913	1.46E-08	3.48E-07	2.57E-07	150/84830/301/348/384/55024/729230/933/8832/1906/2213/2633/9734/3134/3162/3553/7850/90865/353514/10859/4129/114548/55607/26191/91543/6622/80725/5552/10333/51311/7128/7447	32
BP	GO:0050901	leukocyte tethering or rolling	11/926	25/17913	1.52E-08	3.61E-07	2.67E-07	119/6366/729230/2650/3676/50512/6401/6402/6403/6404/7412	11
BP	GO:0043491	protein kinase B signaling	38/926	268/17913	1.53E-08	3.62E-07	2.67E-07	84830/374/55024/6363/6347/6366/6348/6352/1236/930/940/941/942/1116/2069/2099/2152/2261/2651/1839/3059/29851/3481/3553/3932/3084/145957/118788/5293/5294/23533/5880/5979/143686/6662/8600/50852/7409	38
BP	GO:0048667	cell morphogen	57/926	500/17913	1.69	3.99	2.95E-07	107/575/348/655/1952/10752/23242/1436/7852/1630/9201/1749/55619/1959/2048/2297/23105/2674/2817/3676/3792/38	57

© 2020 Yu Y et al. *JAMA Network Open.*

		esis involved in neuron differentiation			E-08	E-07		97/23566/4038/120892/4647/5818/4744/4781/4804/4929/8013/4897/9423/10439/5063/5293/55607/5588/5800/5649/5979/9750/6091/10371/57556/6507/84189/6696/80725/6812/23043/347733/10381/7345/7422/9839	
BP	GO:0030888	regulation of B cell proliferation	16/926	57/17913	1.79E-08	4.21E-07	3.10E-07	100/695/933/952/972/1493/2213/115352/1880/22806/4332/51237/3071/5788/10673/7305	16
BP	GO:0002468	dendritic cell antigen processing and presentation	8/926	12/17913	2.04E-08	4.73E-07	3.49E-07	6363/6366/1236/972/2213/10875/64127/6556	8
BP	GO:0070486	leukocyte aggregation	8/926	12/17913	2.04E-08	4.73E-07	3.49E-07	655/960/3553/4478/8013/5880/6279/7535	8
BP	GO:0010324	membrane invagination	16/926	58/17913	2.36E-08	5.46E-07	4.03E-07	575/199/51411/718/948/9844/2207/2209/2213/3684/3689/8685/4481/3071/26228/54209	16
BP	GO:0002548	monocyte chemotaxis	14/926	44/17913	2.37E-08	5.46E-07	4.03E-07	199/301/6347/6348/6352/729230/3627/1843/2358/3569/7941/5800/56833/8600	14
BP	GO:0050764	regulation of phagocytosis	20/926	90/17913	2.47E-08	5.65E-07	4.17E-07	717/718/720/721/6347/948/1794/2213/2358/3055/3553/3560/3561/3071/5788/5806/140885/6556/26228/7097	20
BP	GO:0032613	interleukin-10 production	15/926	51/17913	2.49E-08	5.65E-07	4.17E-07	29126/940/2207/2213/3620/3662/3718/353514/10859/64127/80380/54440/7097/54209/7305	15
BP	GO:0070098	chemokine-mediated	15/926	51/17913	2.49	5.65	4.17E-07	2532/6366/6352/729230/1233/1236/3627/6373/3577/7852/10663/1906/11240/6091/54209	15

© 2020 Yu Y et al. *JAMA Network Open.*

		signaling pathway			E-08	E-07			
BP	GO:0002825	regulation of T-helper 1 type immune response	11/926	26/17913	2.51E-08	5.65E-07	4.17E-07	301/6363/729230/941/84868/3594/3553/9173/90865/3718/6556	11
BP	GO:0071624	positive regulation of granulocyte chemotaxis	11/926	26/17913	2.51E-08	5.65E-07	4.17E-07	719/728/6363/6366/1236/972/3576/1906/3071/5880/9750	11
BP	GO:0002705	positive regulation of leukocyte mediated immunity	23/926	117/17913	2.67E-08	5.98E-07	4.42E-07	695/718/940/2207/2208/3134/3594/3553/3569/3684/3689/3902/114548/64127/5027/5788/10125/91543/54440/4068/114836/6812/7409	23
BP	GO:0002221	pattern recognition receptor signaling pathway	31/926	197/17913	2.86E-08	6.40E-07	4.72E-07	330/695/929/948/64581/1514/55601/2099/2219/115352/84868/3304/11213/3662/3684/3689/4057/23643/8685/64127/118788/26191/91543/6279/7096/81793/7097/10333/51284/51311/7128	31
BP	GO:0032633	interleukin-4 production	12/926	32/17913	2.93E-08	6.52E-07	4.81E-07	940/916/942/2207/84868/3556/90865/3662/3702/114548/5588/54440	12
BP	GO:0002758	innate immune response-activating signal transduction	40/926	298/17913	3.06E-08	6.77E-07	5.00E-07	330/695/929/30835/948/10462/64581/1514/55601/2099/2207/2219/115352/84868/3055/3304/11213/3662/3684/3689/4057/23643/8685/94025/4583/64127/5063/118788/5696/5698/26191/91543/6279/7096/81793/7097/10333/51284/51311/7128	40
BP	GO:0045058	T cell selection	14/926	45/17913	3.28E-08	7.23E-07	5.33E-07	1236/940/915/916/972/1794/3594/3569/3662/4063/5788/114836/387357/7535	14
B	GO:00	regulation of	19/92	83/17913	3.	7.	5.34E-07	695/729230/948/972/10225/2207/51704/3134/3162/3553/35	1

P	02718	cytokine production involved in immune response	6		30E-08	24E-07		69/11213/3718/10859/114548/64127/8013/91543/54440	9
BP	GO:0043900	regulation of multi-organism process	49/926	408/17913	3.41E-08	7.44E-07	5.49E-07	100/9447/348/384/330/6348/6351/6352/4064/940/948/972/3576/7852/55601/2208/2219/10875/84868/51191/8091/3428/3429/3434/8519/10581/10410/3594/3553/3559/3394/3725/5650/27074/10859/4057/23643/4321/4599/8638/5027/5551/5806/91543/11005/6812/7128/11074/7538	49
BP	GO:0002367	cytokine production involved in immune response	21/926	101/17913	3.84E-08	8.36E-07	6.17E-07	695/729230/948/972/10225/2207/51704/3134/3162/3553/3569/11213/3718/10859/114548/64127/8013/91543/54440/6556/7097	21
BP	GO:1902624	positive regulation of neutrophil migration	11/926	27/17913	4.04E-08	8.74E-07	6.45E-07	719/728/6363/6366/1236/972/3576/1906/3071/5880/9750	11
BP	GO:0070233	negative regulation of T cell apoptotic process	9/926	17/17913	4.24E-08	9.14E-07	6.74E-07	100/384/6352/939/3620/3575/3718/5588/1831	9
BP	GO:0032655	regulation of interleukin-12 production	15/926	53/17913	4.43E-08	9.51E-07	7.02E-07	54/6363/1236/948/3620/11213/3394/3718/353514/10859/4050/4210/6504/7097/51311	15
BP	GO:0050714	positive regulation of protein secretion	36/926	256/17913	4.53E-08	9.69E-07	7.15E-07	199/9447/27063/8938/640/6363/6348/929/914/29126/952/1436/2219/55612/84868/3553/3556/9173/90865/3569/353514/4023/120892/4321/22861/114548/64127/5027/26191/10125/7096/7097/51311/54209/7273/7447	36
BP	GO:0045086	positive regulation of interleukin-2 biosynthetic	8/926	13/17913	5.05E-08	1.08E-06	7.94E-07	940/916/941/942/3553/3662/5588/5788	8

© 2020 Yu Y et al. *JAMA Network Open.*

		process							
BP	GO:1903708	positive regulation of hemopoiesis	29/926	182/17913	6.03E-08	1.28E-06	9.44E-07	100/301/695/6363/939/972/941/942/1960/2124/3059/3304/3594/3559/3575/3725/10288/11006/3071/114548/5788/10125/54440/9840/8600/9760/54209/7305/7535	29
BP	GO:0050855	regulation of B cell receptor signaling pathway	11/926	28/17913	6.34E-08	1.34E-06	9.88E-07	640/930/933/2213/115352/150372/5079/23228/5579/26191/26228	11
BP	GO:0045730	respiratory burst	12/926	34/17913	6.47E-08	1.36E-06	1.00E-06	1043/1536/3055/3512/653361/4688/4689/5293/5294/5880/56833/6556	12
BP	GO:0002792	negative regulation of peptide secretion	25/926	142/17913	6.69E-08	1.40E-06	1.03E-06	150/84830/301/348/384/55024/933/972/2213/2633/9734/3553/7850/90865/353514/10859/114548/26191/91543/80725/5552/10333/51311/7128/7447	25
BP	GO:0007409	axonogenesis	47/926	393/17913	7.39E-08	1.54E-06	1.14E-06	107/575/348/655/10752/23242/1436/7852/1630/9201/1749/1959/2048/2297/23105/2674/2817/3676/3792/3897/23566/4038/5818/4744/4781/4804/4929/8013/4897/9423/10439/5063/5293/5588/5800/5649/5979/6091/10371/57556/6696/6812/347733/10381/7345/7422/9839	47
BP	GO:0032615	interleukin-12 production	15/926	55/17913	7.65E-08	1.59E-06	1.17E-06	54/6363/1236/948/3620/11213/3394/3718/353514/10859/4050/4210/6504/7097/51311	15
BP	GO:0032753	positive regulation of interleukin-4 production	10/926	23/17913	8.03E-08	1.66E-06	1.23E-06	940/916/942/2207/84868/90865/3662/114548/5588/54440	10
BP	GO:0002291	T cell activation via T cell receptor contact with	7/926	10/17913	1.01E-07	2.07E-06	1.53E-06	54518/10875/84868/3109/3383/3683/10859	7

© 2020 Yu Y et al. *JAMA Network Open.*

		antigen bound to MHC molecule on antigen presenting cell							
BP	GO:0045060	negative thymic T cell selection	7/926	10/17913	1.01E-07	2.07E-06	1.53E-06	1236/940/916/972/1794/5788/7535	7
BP	GO:0032731	positive regulation of interleukin-1 beta production	13/926	42/17913	1.09E-07	2.23E-06	1.64E-06	9447/6363/6348/1958/3428/353514/4023/22861/114548/64127/5027/51311/7305	13
BP	GO:0001771	immunological synapse formation	8/926	14/17913	1.13E-07	2.29E-06	1.69E-06	6363/6366/1236/923/1794/84868/4478/5551	8
BP	GO:0032755	positive regulation of interleukin-6 production	18/926	81/17913	1.23E-07	2.49E-06	1.84E-06	199/948/2069/2207/3553/3556/90865/3569/353514/10288/4023/64127/5027/7096/7097/10333/51311/7305	18
BP	GO:0019722	calcium-mediated signaling	29/926	188/17913	1.24E-07	2.51E-06	1.85E-06	100/695/6364/6348/6351/933/916/3576/7852/1906/2358/2633/2903/2904/120892/79772/3084/5027/5031/5341/5788/1827/6401/6403/8600/50852/54209/7412/7535	29
BP	GO:0010951	negative regulation of endopeptidase activity	31/926	210/17913	1.28E-07	2.58E-06	1.90E-06	653145/274/718/720/721/135228/939/960/340267/1285/1378/1476/1800/3428/2537/27074/4018/4057/4318/4804/89932/5266/5329/5272/6622/11005/9806/50859/7035/7980/7422	31
BP	GO:0045824	negative regulation of innate immune	15/926	57/17913	1.29E-07	2.58E-06	1.90E-06	10225/1378/84868/3134/3428/11213/10859/4321/197358/84166/5272/710/56833/7128/11326	15

© 2020 Yu Y et al. *JAMA Network Open.*

		response							
BP	GO:0002407	dendritic cell chemotaxis	10/926	24/17913	1.31E-07	2.62E-06	1.93E-06	6363/6366/6352/729230/1236/3577/7852/1880/5294/56833	10
BP	GO:0035710	CD4-positive, alpha-beta T cell activation	19/926	90/17913	1.32E-07	2.62E-06	1.93E-06	301/384/6363/29126/941/942/1880/3594/3569/3662/3718/4063/3071/114548/26279/5588/91543/54440/114836	19
BP	GO:0046632	alpha-beta T cell differentiation	20/926	99/17913	1.33E-07	2.64E-06	1.95E-06	100/301/6363/941/942/8320/1880/3594/3569/3662/3718/4063/3071/114548/26279/639/91543/54440/114836/7535	20
BP	GO:0050709	negative regulation of protein secretion	24/926	137/17913	1.34E-07	2.64E-06	1.95E-06	150/84830/301/348/384/55024/933/2213/2633/9734/3553/7850/90865/353514/10859/114548/26191/91543/80725/5552/10333/51311/7128/7447	24
BP	GO:0002224	toll-like receptor signaling pathway	25/926	147/17913	1.35E-07	2.64E-06	1.95E-06	330/695/929/948/1514/2099/115352/84868/11213/3662/3684/3689/4057/23643/118788/26191/91543/6279/7096/81793/7097/10333/51284/51311/7128	25
BP	GO:0031343	positive regulation of cell killing	14/926	50/17913	1.45E-07	2.82E-06	2.08E-06	2208/3134/3594/3684/3902/5027/5551/5788/10125/4068/114836/26228/7305/7409	14
BP	GO:0032732	positive regulation of interleukin-1 production	14/926	50/17913	1.45E-07	2.82E-06	2.08E-06	9447/6363/6348/1958/84868/3428/353514/4023/22861/114548/64127/5027/51311/7305	14
BP	GO:0002828	regulation of type 2 immune response	11/926	30/17913	1.47E-07	2.85E-06	2.10E-06	301/384/729230/972/942/3620/90865/3569/114548/64127/91543	11
BP	GO:0010466	negative regulation of	32/926	223/17913	1.57	3.04	2.25E-06	653145/274/718/720/721/135228/939/960/340267/1285/1378/1476/1800/3428/2537/27074/4018/4057/4318/4804/89932/	32

© 2020 Yu Y et al. *JAMA Network Open.*

		peptidase activity			E-07	E-06		51050/5266/5329/5272/6622/11005/9806/50859/7035/7980/7422	
BP	GO:0002708	positive regulation of lymphocyte mediated immunity	19/926	91/17913	1.58E-07	3.05E-06	2.25E-06	695/718/940/2207/2208/3134/3594/3553/3569/3902/114548/5027/5788/10125/91543/54440/4068/114836/7409	19
BP	GO:0022604	regulation of cell morphogenesis	49/926	430/17913	1.75E-07	3.37E-06	2.49E-06	301/348/55843/6357/6347/6348/960/11151/1436/7852/1630/5168/23136/2048/752/114793/23105/2633/23426/3055/3383/3689/3792/3897/23566/4038/120892/4478/4804/4897/9423/10439/5063/55607/5800/5649/5979/6091/6281/10371/57619/375611/6696/80725/51760/23043/347733/7422/9839	49
BP	GO:0070228	regulation of lymphocyte apoptotic process	14/926	51/17913	1.90E-07	3.62E-06	2.67E-06	100/384/695/6352/939/29126/917/972/3620/3575/3718/5027/5588/1831	14
BP	GO:0150077	regulation of neuroinflammatory response	14/926	51/17913	1.90E-07	3.62E-06	2.67E-06	712/728/6348/3553/90865/3569/3684/3689/120892/4318/26471/5788/26228/54209	14
BP	GO:0050706	regulation of interleukin-1 beta secretion	13/926	44/17913	2.01E-07	3.81E-06	2.81E-06	9447/384/6363/6348/1236/353514/4023/22861/114548/64127/5027/51311/7128	13
BP	GO:0071622	regulation of granulocyte chemotaxis	13/926	44/17913	2.01E-07	3.81E-06	2.81E-06	719/728/6363/6366/6352/1236/972/3576/1906/4354/3071/5880/9750	13
BP	GO:0031348	negative regulation of defense response	33/926	237/17913	2.07E-07	3.91E-06	2.88E-06	54/100/113/348/384/10225/1378/2213/10875/2358/84868/3134/3428/7850/3559/11213/10859/4210/4321/197358/84166/114548/4907/5788/5272/710/89790/140885/56833/9021/11005/7128/11326	33
BP	GO:0010818	T cell chemotaxis	10/926	25/17913	2.09E-07	3.92E-06	2.89E-06	6366/6348/6352/729230/3627/6373/10563/1880/5293/5294	10

BP	GO:0050704	regulation of interleukin-1 secretion	14/926	52/17913	2.48E-07	4.63E-06	3.42E-06	9447/384/6363/6348/1236/7850/353514/4023/22861/114548/64127/5027/51311/7128	14
BP	GO:0043277	apoptotic cell clearance	12/926	38/17913	2.64E-07	4.91E-06	3.63E-06	575/717/718/720/721/6347/948/2219/8685/7052/54209/7305	12
BP	GO:0032490	detection of molecule of bacterial origin	7/926	11/17913	2.65E-07	4.92E-06	3.63E-06	721/23643/64127/7096/7097/10333/54209	7
BP	GO:2000401	regulation of lymphocyte migration	15/926	60/17913	2.68E-07	4.96E-06	3.66E-06	84830/199/6347/6364/6366/6348/6351/6352/729230/3627/10563/3676/4478/11240/9750	15
BP	GO:0061756	leukocyte adhesion to vascular endothelial cell	13/926	45/17913	2.70E-07	4.98E-06	3.67E-06	119/6366/729230/2650/3383/3676/3689/50512/6401/6402/6403/6404/7412	13
BP	GO:0061564	axon development	49/926	438/17913	3.07E-07	5.63E-06	4.16E-06	107/575/348/655/10752/23242/1436/7852/1630/9201/1749/1959/2048/2297/23105/2674/2817/3676/3725/3792/3897/23566/4038/5818/4744/4781/4804/4929/8013/4897/9423/10439/5063/5293/5588/5800/5649/5979/56963/6091/10371/57556/6696/6812/347733/10381/7345/7422/9839	49
BP	GO:0051896	regulation of protein kinase B signaling	33/926	241/17913	3.08E-07	5.63E-06	4.16E-06	374/55024/6363/6366/6348/1236/930/940/941/942/1116/2069/2099/2152/2261/2651/1839/3059/29851/3481/3932/3084/145957/118788/5293/5294/23533/5880/5979/143686/8600/50852/7409	33
BP	GO:0043368	positive T cell selection	11/926	32/17913	3.15E-07	5.74E-06	4.23E-06	915/972/1794/3594/3569/3662/4063/5788/114836/387357/7535	11
BP	GO:0032602	chemokine production	18/926	86/17913	3.22	5.84	4.31E-06	199/384/972/1436/1958/84868/3162/3553/9173/90865/3569/4023/4210/6279/140885/7097/51284/54209	18

© 2020 Yu Y et al. *JAMA Network Open.*

					E-07	E-06			
BP	GO:0002507	tolerance induction	10/926	26/17913	3.23E-07	5.85E-06	4.32E-06	1233/29126/916/84868/29851/3620/3559/11213/10288/7128	10
BP	GO:0046635	positive regulation of alpha-beta T cell activation	15/926	61/17913	3.39E-07	6.11E-06	4.51E-06	100/301/6363/729230/940/916/941/942/3594/3071/114548/5588/5788/54440/7535	15
BP	GO:0002830	positive regulation of type 2 immune response	8/926	16/17913	4.39E-07	7.87E-06	5.81E-06	972/942/3620/90865/3569/114548/64127/91543	8
BP	GO:0032695	negative regulation of interleukin-12 production	8/926	16/17913	4.39E-07	7.87E-06	5.81E-06	54/11213/3718/353514/10859/4210/6504/51311	8
BP	GO:0032642	regulation of chemokine production	17/926	79/17913	4.48E-07	8.00E-06	5.91E-06	199/384/972/1436/1958/84868/3162/3553/9173/90865/3569/4023/4210/140885/7097/51284/54209	17
BP	GO:0043032	positive regulation of macrophage activation	11/926	33/17913	4.50E-07	8.00E-06	5.91E-06	712/6348/84868/9173/90865/3684/3689/120892/26228/10333/54209	11
BP	GO:2000116	regulation of cysteine-type endopeptidase activity	31/926	222/17913	4.52E-07	8.02E-06	5.92E-06	9447/330/939/960/1285/26999/1800/112399/2152/355/2903/2904/3428/2537/27074/3932/4057/4210/4318/4804/22861/114548/64065/5329/5698/6091/6279/5272/6622/8626/7422	31
BP	GO:0002456	T cell mediated immunity	18/926	88/17913	4.62E-	8.16E-	6.03E-06	729230/2213/3134/3383/3594/3553/3569/3575/10859/64005/114548/5027/5551/5788/91543/54440/6556/7454	18

© 2020 Yu Y et al. *JAMA Network Open.*

					07	06			
BP	GO:0032673	regulation of interleukin-4 production	10/926	27/17913	4.90E-07	8.59E-06	6.34E-06	940/916/942/2207/84868/90865/3662/114548/5588/54440	10
BP	GO:0036336	dendritic cell migration	10/926	27/17913	4.90E-07	8.59E-06	6.34E-06	6363/6366/6352/729230/1236/3577/7852/1880/5294/56833	10
BP	GO:0001912	positive regulation of leukocyte mediated cytotoxicity	12/926	40/17913	4.94E-07	8.64E-06	6.38E-06	3134/3594/3684/3902/5027/5788/10125/4068/114836/26228/7305/7409	12
BP	GO:0070664	negative regulation of leukocyte proliferation	16/926	71/17913	5.01E-07	8.73E-06	6.44E-06	384/695/29126/1493/2213/10457/84868/3620/3559/10859/10288/4332/80380/7128/7305/11326	16
BP	GO:0032102	negative regulation of response to external stimulus	41/926	343/17913	5.09E-07	8.84E-06	6.52E-06	54/100/113/84830/199/348/384/6347/135228/10563/1843/1906/83481/2213/10875/2358/2811/84868/7850/3559/4057/4210/197358/114548/4907/11240/5329/5788/5800/56963/6091/710/89790/140885/56833/9021/11005/6696/26228/7035/7128	41
BP	GO:0002573	myeloid leukocyte differentiation	29/926	201/17913	5.30E-07	9.17E-06	6.77E-06	714/6363/6348/1236/135228/972/1021/1436/2124/2207/1880/3059/3428/133396/3662/3725/10859/11006/4057/4318/5293/8544/6688/7097/8600/54209/7305/10537/7422	29
BP	GO:0002532	production of molecular mediator involved in inflammatory response	16/926	72/17913	6.14E-07	1.06E-05	7.81E-06	113/695/948/923/10225/2207/115362/3620/7850/4210/197358/64127/5187/140885/56833/10333	16
BP	GO:0002704	negative regulation of leukocyte	13/926	48/17913	6.17E-	1.06E-	7.83E-06	729230/8832/10225/1378/2213/84868/3134/3162/3575/3718/10859/5788/5272	13

© 2020 Yu Y et al. *JAMA Network Open.*

		mediated immunity			07	05			
BP	GO:0010038	response to metal ion	40/926	335/17913	7.20E-07	1.23E-05	9.10E-06	107/113/84059/241/366/712/929/999/1396/1536/1800/1843/1906/2729/3162/3383/3725/120892/4129/4318/4489/4493/4499/4501/4502/653361/10397/5027/10891/10235/6279/6331/375611/6622/7020/7031/7054/4982/7273/7412	40
BP	GO:0070229	negative regulation of lymphocyte apoptotic process	10/926	28/17913	7.27E-07	1.24E-05	9.15E-06	100/384/6352/939/972/3620/3575/3718/5588/1831	10
BP	GO:0043901	negative regulation of multi-organism process	27/926	182/17913	7.43E-07	1.26E-05	9.33E-06	100/384/6348/6351/6352/948/2219/10875/84868/8091/3428/3434/8519/10581/10410/3559/3394/3725/4057/4599/8638/5806/91543/11005/7128/11074/7538	27
BP	GO:0019058	viral life cycle	39/926	324/17913	8.07E-07	1.37E-05	1.01E-05	290/348/6347/6352/30835/940/972/941/942/10332/1378/1380/1525/3576/7852/10663/1803/2219/2838/8091/3304/3383/3428/3429/3434/8519/10581/10410/27074/4057/4360/4599/5818/8638/5806/91543/6404/6504/11074	39
BP	GO:0032945	negative regulation of mononuclear cell proliferation	15/926	65/17913	8.20E-07	1.38E-05	1.02E-05	384/695/29126/1493/2213/10457/84868/3620/3559/10859/10288/4332/80380/7305/11326	15
BP	GO:0050672	negative regulation of lymphocyte proliferation	15/926	65/17913	8.20E-07	1.38E-05	1.02E-05	384/695/29126/1493/2213/10457/84868/3620/3559/10859/10288/4332/80380/7305/11326	15
BP	GO:0002701	negative regulation of production of molecular mediator of immune response	11/926	35/17913	8.82E-07	1.47E-05	1.09E-05	933/10225/2213/115352/3134/3162/90865/11213/3718/10859/11005	11
B	GO:00	heterophilic	11/92	35/17913	8.	1.	1.09E-05	57863/30835/923/1525/3383/3683/120425/5818/6401/6403/	1

P	07157	cell-cell adhesion via plasma membrane cell adhesion molecules	6		82 E-07	47 E-05		7412	1
BP	GO:0042092	type 2 immune response	11/926	35/17913	8.82 E-07	1.47 E-05	1.09E-05	301/384/729230/972/942/3620/90865/3569/114548/64127/91543	11
BP	GO:0070838	divalent metal ion transport	49/926	454/17913	8.90 E-07	1.48 E-05	1.09E-05	150/309/489/9914/274/777/6363/6347/6366/6348/6351/6352/1236/930/8832/57214/11151/3627/6373/4283/7852/115352/2903/2904/3383/3603/3932/353514/10859/10288/23566/931/9722/5027/5031/5142/5294/5579/5788/22821/6236/6556/84102/6622/6769/342667/80036/140803/7991	49
BP	GO:0043367	CD4-positive, alpha-beta T cell differentiation	16/926	74/17913	9.10 E-07	1.51 E-05	1.11E-05	301/6363/941/942/1880/3594/3569/3662/3718/4063/3071/114548/26279/91543/54440/114836	16
BP	GO:0006816	calcium ion transport	46/926	415/17913	9.17 E-07	1.51 E-05	1.12E-05	150/309/489/9914/274/777/6363/6347/6366/6348/6351/6352/1236/930/8832/57214/11151/3627/6373/4283/7852/115352/2903/2904/3383/3603/3932/353514/10859/10288/23566/931/9722/5027/5031/5142/5294/5579/5788/22821/6236/6622/6769/342667/80036/140803	46
BP	GO:1903038	negative regulation of leukocyte cell-cell adhesion	21/926	121/17913	9.47 E-07	1.56 E-05	1.15E-05	84830/301/384/6366/29126/972/1493/2213/10457/84868/3620/3559/3718/3902/54900/10859/10288/80380/26191/9750/11326	21
BP	GO:0043270	positive regulation of ion transport	34/926	266/17913	1.04 E-06	1.71 E-05	1.26E-05	5243/5244/150/348/9914/10396/6347/6348/6351/6352/729230/930/57214/3627/6373/4283/1906/2048/3359/3553/8564/353514/23566/9722/5027/5031/5649/6331/6622/6769/342667/6812/8600/54209	34

© 2020 Yu Y et al. *JAMA Network Open.*

BP	GO:0050718	positive regulation of interleukin-1 beta secretion	10/926	29/17913	1.06E-06	1.73E-05	1.28E-05	9447/6363/6348/353514/4023/22861/114548/64127/5027/51311	10
BP	GO:0050857	positive regulation of antigen receptor-mediated signaling pathway	9/926	23/17913	1.07E-06	1.75E-05	1.29E-05	100/1236/3932/150372/5579/5788/26228/9840/50852	9
BP	GO:0002702	positive regulation of production of molecular mediator of immune response	18/926	93/17913	1.09E-06	1.77E-05	1.31E-05	940/948/972/2207/51704/3134/3553/90865/3569/5650/10859/51237/114548/64127/8013/5788/91543/54440	18
BP	GO:0072511	divalent inorganic cation transport	49/926	458/17913	1.15E-06	1.86E-05	1.37E-05	150/309/489/9914/274/777/6363/6347/6366/6348/6351/6352/1236/930/8832/57214/11151/3627/6373/4283/7852/115352/2903/2904/3383/3603/3932/353514/10859/10288/23566/931/9722/5027/5031/5142/5294/5579/5788/22821/6236/6556/84102/6622/6769/342667/80036/140803/7991	49
BP	GO:0097028	dendritic cell differentiation	12/926	43/17913	1.18E-06	1.90E-05	1.40E-05	6363/1236/2213/2322/3662/10859/10288/6688/55365/28959/54209/10537	12
BP	GO:0002755	MyD88-dependent toll-like receptor signaling pathway	11/926	36/17913	1.21E-06	1.95E-05	1.44E-05	695/929/948/11213/23643/7096/81793/7097/10333/51284/51311	11
BP	GO:0070227	lymphocyte apoptotic process	15/926	67/17913	1.24E-	1.99E-	1.47E-05	100/384/695/6352/939/29126/917/972/3620/3559/3575/3718/5027/5588/1831	15

© 2020 Yu Y et al. *JAMA Network Open.*

					06	05			
BP	GO:0050808	synapse organization	44/926	394/17913	1.29E-06	2.06E-05	1.52E-05	119/575/348/712/713/714/718/728/1006/1134/22866/55619/2048/2213/2904/57549/3587/3556/9118/3675/3684/3897/10288/4038/120892/266727/10397/5818/4744/9722/4897/3084/9423/5063/55607/5800/5649/54549/22941/84189/6622/9806/80725/7097	44
BP	GO:0042742	defense response to bacterium	32/926	245/17913	1.34E-06	2.13E-05	1.57E-05	54/575/306/384/728/6364/948/10563/2358/10578/84868/3569/3394/3512/5650/4057/4069/219972/22861/114548/64127/5027/51316/6279/6403/56833/6556/11005/23166/55576/7097/10333	32
BP	GO:0050868	negative regulation of T cell activation	19/926	104/17913	1.39E-06	2.20E-05	1.63E-05	301/384/29126/972/1493/2213/10457/84868/3620/3559/3718/3902/54900/10859/10288/80380/26191/9750/11326	19
BP	GO:0051224	negative regulation of protein transport	27/926	189/17913	1.58E-06	2.50E-05	1.84E-05	150/84830/301/348/384/55024/933/948/2213/55612/2633/9734/3553/7850/90865/353514/10859/120892/114548/26191/91543/80725/5552/10333/51311/7128/7447	27
BP	GO:0002260	lymphocyte homeostasis	14/926	60/17913	1.65E-06	2.61E-05	1.92E-05	100/972/11151/55619/139818/84636/3559/3718/3071/5027/124976/7128/10673/1831	14
BP	GO:0002720	positive regulation of cytokine production involved in immune response	13/926	52/17913	1.68E-06	2.63E-05	1.94E-05	948/972/2207/51704/3134/3553/3569/10859/114548/64127/8013/91543/54440	13
BP	GO:0002886	regulation of myeloid leukocyte mediated immunity	13/926	52/17913	1.68E-06	2.63E-05	1.94E-05	30817/695/718/729230/8832/1776/2207/2213/3162/3684/3689/5880/6812	13
BP	GO:0042590	antigen processing	16/926	78/17913	1.92	3.00	2.21E-05	948/1536/2209/3105/3106/3107/3134/3136/10437/653361/4688/4689/5696/5698/6890/6891	16

© 2020 Yu Y et al. *JAMA Network Open.*

		and presentation of exogenous peptide antigen via MHC class I			E-06	E-05			
BP	GO:0002448	mast cell mediated immunity	12/926	45/17913	2.00E-06	3.11E-05	2.30E-05	30817/695/8832/2207/3162/8013/5293/5294/5880/10125/5272/6812	12
BP	GO:0033628	regulation of cell adhesion mediated by integrin	12/926	45/17913	2.00E-06	3.11E-05	2.30E-05	100/6366/6352/916/10563/1803/55612/83706/3071/5140/5294/5979	12
BP	GO:0002709	regulation of T cell mediated immunity	14/926	61/17913	2.04E-06	3.15E-05	2.33E-05	729230/2213/3134/3594/3553/3569/3575/10859/114548/5027/5788/91543/54440/7454	14
BP	GO:0033627	cell adhesion mediated by integrin	14/926	61/17913	2.04E-06	3.15E-05	2.33E-05	100/6366/6352/916/10563/1803/55612/83706/3383/3676/3071/5140/5294/5979	14
BP	GO:1903978	regulation of microglial cell activation	10/926	31/17913	2.13E-06	3.28E-05	2.42E-05	712/728/6348/3569/3684/3689/120892/5788/26228/54209	10
BP	GO:0045061	thymic T cell selection	8/926	19/17913	2.25E-06	3.44E-05	2.54E-05	1236/940/915/916/972/1794/5788/7535	8
BP	GO:0090066	regulation of anatomical structure size	47/926	442/17913	2.25E-06	3.44E-05	2.54E-05	119/150/10000/8862/348/274/146206/6366/1236/952/11151/1630/1906/342184/23105/2729/3055/3059/3162/3274/3383/3575/10788/3792/3897/23566/120892/4478/3071/4868/4897/9423/10439/5027/5029/5063/5341/55607/5979/5997/10371/	47

© 2020 Yu Y et al. *JAMA Network Open.*

								375611/6648/6696/7409/7422/7454	
BP	GO:1904950	negative regulation of establishment of protein localization	27/926	193/17913	2.38E-06	3.63E-05	2.68E-05	150/84830/301/348/384/55024/933/948/2213/55612/2633/9734/3553/7850/90865/353514/10859/120892/114548/26191/91543/80725/5552/10333/51311/7128/7447	27
BP	GO:0002335	mature B cell differentiation	9/926	25/17913	2.45E-06	3.71E-05	2.74E-05	100/930/55619/139818/2213/1880/9452/23228/56833	9
BP	GO:1901623	regulation of lymphocyte chemotaxis	9/926	25/17913	2.45E-06	3.71E-05	2.74E-05	6347/6366/6348/6351/6352/729230/3627/10563/11240	9
BP	GO:0016064	immunoglobulin mediated immune response	25/926	171/17913	2.49E-06	3.76E-05	2.78E-05	695/712/713/714/717/718/720/721/730/930/939/940/972/3426/1378/1380/2207/2208/2213/10875/3119/5788/710/55061/51311	25
BP	GO:0034121	regulation of toll-like receptor signaling pathway	15/926	71/17913	2.71E-06	4.08E-05	3.01E-05	330/929/948/2099/115352/11213/3662/4057/23643/26191/91543/7096/7097/10333/7128	15
BP	GO:0070555	response to interleukin-1	26/926	183/17913	2.72E-06	4.09E-05	3.02E-05	27063/6347/6348/6352/952/1116/3576/1906/1958/2633/2729/3383/3553/7850/3556/3569/11213/8564/64127/5696/5698/6401/140885/6622/6662/7035	26
BP	GO:0032535	regulation of cellular component size	37/926	315/17913	2.76E-06	4.14E-05	3.05E-05	119/10000/348/274/146206/6366/1236/11151/1630/1906/342184/23105/3055/3059/3383/3575/10788/3792/3897/23566/4478/3071/4868/4897/9423/10439/5027/5063/5341/55607/5979/10371/375611/6696/7409/7422/7454	37
BP	GO:0008037	cell recognition	21/926	129/17913	2.77E-06	4.14E-05	3.06E-05	575/6363/6366/1236/30835/948/921/923/10332/64581/55790/7852/1794/2048/2219/84868/4478/4897/5551/6091/10371	21

© 2020 Yu Y et al. *JAMA Network Open.*

BP	GO:0048525	negative regulation of viral process	18/926	99/17913	2.81E-06	4.19E-05	3.09E-05	6348/6351/6352/2219/8091/3428/3434/8519/10581/10410/3725/4057/4599/8638/5806/91543/11074/7538	18
BP	GO:0070232	regulation of T cell apoptotic process	10/926	32/17913	2.96E-06	4.39E-05	3.24E-05	100/384/6352/939/29126/3620/3575/3718/5588/1831	10
BP	GO:0072376	protein activation cascade	23/926	151/17913	3.06E-06	4.53E-05	3.34E-05	712/713/714/717/718/719/720/721/728/730/930/629/3426/1378/1380/2152/2219/2811/3553/710/55061/7035/11326	23
BP	GO:0002228	natural killer cell mediated immunity	14/926	63/17913	3.08E-06	4.53E-05	3.34E-05	10225/11151/3002/84868/3134/3822/3902/10859/10125/5272/4068/114836/57823/7409	14
BP	GO:0030101	natural killer cell activation	14/926	63/17913	3.08E-06	4.53E-05	3.34E-05	914/11151/84868/3134/50615/3689/5293/639/26191/5788/10125/57823/9760/7305	14
BP	GO:0019724	B cell mediated immunity	25/926	173/17913	3.08E-06	4.53E-05	3.34E-05	695/712/713/714/717/718/720/721/730/930/939/940/972/3426/1378/1380/2207/2208/2213/10875/3119/5788/710/55061/51311	25
BP	GO:0050710	negative regulation of cytokine secretion	15/926	72/17913	3.26E-06	4.77E-05	3.52E-05	301/384/55024/2213/2633/9734/7850/353514/10859/114548/26191/5552/10333/51311/7128	15
BP	GO:0001768	establishment of T cell polarity	6/926	10/17913	3.30E-06	4.82E-05	3.55E-05	6363/6366/1236/56603/1794/9750	6
BP	GO:0031663	lipopolysaccharide-mediated signaling	13/926	55/17913	3.32E-06	4.82E-05	3.56E-05	6347/6348/6352/4064/948/923/3055/3553/4057/23643/26191/7097/7128	13

© 2020 Yu Y et al. *JAMA Network Open.*

		pathway							
BP	GO:0032722	positive regulation of chemokine production	13/926	55/17913	3.32E-06	4.82E-05	3.56E-05	199/972/1436/1958/84868/3162/3553/9173/90865/3569/4023/7097/51284	13
BP	GO:0051924	regulation of calcium ion transport	31/926	244/17913	3.50E-06	5.07E-05	3.74E-05	150/9914/274/6347/6348/6351/6352/930/8832/57214/11151/3627/6373/4283/7852/115352/3383/3603/353514/10859/10288/23566/9722/5027/5031/5142/5294/6236/6622/6769/342667	31
BP	GO:0007411	axon guidance	28/926	210/17913	4.04E-06	5.83E-05	4.30E-05	655/10752/1436/7852/1630/1749/1959/2048/2297/2674/2817/3897/5818/4781/8013/4897/9423/5293/5588/5800/5649/5979/6091/10371/57556/347733/10381/7422	28
BP	GO:0097242	amyloid-beta clearance	10/926	33/17913	4.05E-06	5.83E-05	4.30E-05	348/718/728/948/3684/3689/4038/8685/4481/54209	10
BP	GO:0061098	positive regulation of protein tyrosine kinase activity	13/926	56/17913	4.12E-06	5.91E-05	4.36E-05	150/389658/6352/1436/2069/2209/51704/353514/653361/3084/5649/80725/26228	13
BP	GO:0046629	gamma-delta T cell activation	7/926	15/17913	4.31E-06	6.17E-05	4.55E-05	1525/1960/120425/10859/3071/64127/5788	7
BP	GO:0051701	interaction with host	28/926	211/17913	4.44E-06	6.33E-05	4.67E-05	290/30835/972/941/942/999/10332/1378/1380/1525/3576/7852/1803/2219/2838/3304/3383/3434/8519/10581/10410/4360/5818/5806/6404/5272/6504/11074	28
BP	GO:0043405	regulation of MAP kinase activity	37/926	322/17913	4.62E-06	6.58E-05	4.86E-05	150/348/655/728/6363/972/7852/1843/1906/2048/10595/2322/2357/3553/11213/54900/23566/120892/11184/64127/3084/5027/5063/5294/23533/26191/5788/10125/5979/5997/6091/10333/8600/23043/7345/7422/9839	37
B	GO:00	neuron	28/92	212/1791	4.	6.	5.10E-05	655/10752/1436/7852/1630/1749/1959/2048/2297/2674/281	2

© 2020 Yu Y et al. *JAMA Network Open.*

P	97485	projection guidance	6	3	86 E-06	91 E-05		7/3897/5818/4781/8013/4897/9423/5293/5588/5800/5649/5979/6091/10371/57556/347733/10381/7422	8
BP	GO:0045576	mast cell activation	13/926	57/17913	5.08 E-06	7.20 E-05	5.31E-05	30817/695/8832/2207/3162/3937/10397/8013/5293/5294/5880/10125/6812	13
BP	GO:0002534	cytokine production involved in inflammatory response	12/926	49/17913	5.31 E-06	7.49 E-05	5.53E-05	113/923/10225/115362/3620/7850/4210/197358/64127/5187/140885/10333	12
BP	GO:0071248	cellular response to metal ion	24/926	167/17913	5.32 E-06	7.50 E-05	5.53E-05	107/113/84059/241/999/1536/1800/1906/3162/3725/120892/4318/4489/4493/4499/4501/4502/653361/10891/10235/6331/6622/7020/7054	24
BP	GO:2000403	positive regulation of lymphocyte migration	10/926	34/17913	5.47 E-06	7.68 E-05	5.67E-05	199/6364/6366/6348/6351/6352/729230/3627/10563/3676	10
BP	GO:0097186	amelogenesis	8/926	21/17913	5.51 E-06	7.72 E-05	5.69E-05	1441/54757/3691/5818/152816/64065/6899/256764	8
BP	GO:0051222	positive regulation of protein transport	43/926	403/17913	5.54 E-06	7.73 E-05	5.71E-05	199/9447/27063/8938/640/6363/6348/929/914/29126/952/999/57214/1436/2219/55612/84868/3059/3553/3556/9173/90865/3569/3684/3689/353514/4023/120892/4321/23255/22861/114548/64127/5027/26191/5880/10125/7096/7097/51311/54209/7273/7447	43
BP	GO:0002479	antigen processing and presentation of exogenous peptide	15/926	75/17913	5.56 E-06	7.74 E-05	5.71E-05	948/1536/2209/3105/3106/3107/3134/3136/653361/4688/4689/5696/5698/6890/6891	15

© 2020 Yu Y et al. *JAMA Network Open.*

		antigen via MHC class I, TAP-dependent							
BP	GO:0042088	T-helper 1 type immune response	11/926	42/17913	6.49E-06	9.01E-05	6.65E-05	301/6363/729230/941/84868/3594/3553/9173/90865/3718/6556	11
BP	GO:0002474	antigen processing and presentation of peptide antigen via MHC class I	17/926	95/17913	6.60E-06	9.14E-05	6.75E-05	948/1536/64167/2209/3105/3106/3107/3134/3136/10437/653361/4688/4689/5696/5698/6890/6891	17
BP	GO:0001767	establishment of lymphocyte polarity	6/926	11/17913	6.93E-06	9.50E-05	7.01E-05	6363/6366/1236/56603/1794/9750	6
BP	GO:0032494	response to peptidoglycan	6/926	11/17913	6.93E-06	9.50E-05	7.01E-05	728/3569/11213/114548/64127/54209	6
BP	GO:0033632	regulation of cell-cell adhesion mediated by integrin	6/926	11/17913	6.93E-06	9.50E-05	7.01E-05	100/6352/916/10563/1803/83706	6
BP	GO:0035747	natural killer cell chemotaxis	6/926	11/17913	6.93E-06	9.50E-05	7.01E-05	6347/6348/6351/6352/5293/5294	6
BP	GO:0042476	odontogenesis	20/926	126/17913	7.05E-	9.64E-	7.11E-05	249/655/1441/1906/54757/3691/4038/5818/152816/64065/5251/860/6331/6899/7020/4982/8600/8626/7286/256764	20

© 2020 Yu Y et al. *JAMA Network Open.*

					06	05			
BP	GO:0050716	positive regulation of interleukin-1 secretion	10/926	35/17913	7.30E-06	9.94E-05	7.33E-05	9447/6363/6348/353514/4023/22861/114548/64127/5027/51311	10
BP	GO:0033631	cell-cell adhesion mediated by integrin	7/926	16/17913	7.31E-06	9.94E-05	7.33E-05	100/6352/916/10563/1803/83706/3676	7
BP	GO:0030449	regulation of complement activation	19/926	116/17913	7.45E-06	0.000100922	7.45E-05	712/713/714/717/718/719/720/721/728/730/930/629/3426/1378/1380/3553/710/55061/11326	19
BP	GO:2000514	regulation of CD4-positive, alpha-beta T cell activation	13/926	59/17913	7.64E-06	0.000103209	7.62E-05	301/384/6363/29126/941/942/3594/3662/3718/3071/114548/5588/54440	13
BP	GO:0009595	detection of biotic stimulus	8/926	22/17913	8.27E-06	0.000111439	8.22E-05	721/23643/114548/64127/7096/7097/10333/54209	8
BP	GO:0043300	regulation of leukocyte degranulation	11/926	43/17913	8.31E-06	0.000111439	8.22E-05	30817/729230/8832/2207/2213/3134/3162/3684/3689/5880/6812	11
BP	GO:0043303	mast cell degranulation	11/926	43/17913	8.31E-	0.0001	8.22E-05	30817/695/8832/2207/3162/8013/5293/5294/5880/10125/6812	11

© 2020 Yu Y et al. *JAMA Network Open.*

					06	11439			
BP	GO:2000257	regulation of protein activation cascade	19/926	117/17913	8.47E-06	0.000113234	8.36E-05	712/713/714/717/718/719/720/721/728/730/930/629/3426/1378/1380/3553/710/55061/11326	19
BP	GO:0071900	regulation of protein serine/threonine kinase activity	48/926	479/17913	8.97E-06	0.000119601	8.83E-05	150/348/655/728/6363/894/972/57214/1436/7852/1843/1906/2048/10595/54757/2322/2357/51191/8091/3481/3553/11213/54900/23566/120892/4057/11184/64127/3084/5027/5063/5294/23533/5577/26191/5788/10125/5979/5997/6091/6622/10333/7128/8600/23043/7345/7422/9839	48
BP	GO:0006956	complement activation	20/926	128/17913	9.00E-06	0.000119682	8.83E-05	712/713/714/717/718/719/720/721/728/730/930/629/3426/1378/1380/2219/3553/710/55061/11326	20
BP	GO:0030099	myeloid cell differentiation	43/926	411/17913	9.08E-06	0.000120527	8.90E-05	714/6363/6348/1236/135228/972/1021/1436/1441/2124/2207/2811/29909/1880/3059/8356/8361/3218/3304/3428/10320/133396/3662/3394/3725/10859/11006/4057/4318/3071/8013/5293/8544/5579/5588/6688/7097/8600/54209/7305/10537/7422/7538	43
BP	GO:0002831	regulation of response to biotic stimulus	21/926	139/17913	9.27E-06	0.000122227	9.06E-05	9447/384/330/4064/29126/948/55601/10875/84868/51191/3434/3594/3553/3559/5650/10859/4057/23643/4321/11005/7128	21
BP	GO:0033077	T cell differentiation in thymus	14/926	69/17913	9.53E-06	0.00012583	9.29E-05	100/1236/940/915/916/972/1021/1794/1960/3575/5788/10125/9840/7535	14

© 2020 Yu Y et al. *JAMA Network Open.*

						3			
BP	GO:0072606	interleukin-8 secretion	9/926	29/17913	9.94E-06	0.000130907	9.66E-05	301/929/914/1116/2219/26191/7096/7097/10333	9
BP	GO:1904951	positive regulation of establishment of protein localization	45/926	440/17913	1.02E-05	0.000133356	9.84E-05	199/9447/27063/8938/640/6363/6348/929/914/29126/952/999/57214/1436/2219/55612/3002/84868/3059/3553/3556/9173/90865/3569/3684/3689/353514/4023/120892/4321/23255/22861/114548/64127/5027/26191/5880/10125/7096/7097/51311/8626/54209/7273/7447	45
BP	GO:0051091	positive regulation of DNA-binding transcription factor activity	31/926	257/17913	1.02E-05	0.00013387	9.88E-05	107/9447/306/695/948/1906/2099/3059/3304/3383/3553/3556/3569/11213/3689/4057/150372/114548/64127/10891/5579/5588/5649/54101/6279/7097/10333/8600/10346/11074/7422	31
BP	GO:0070997	neuron death	38/926	347/17913	1.06E-05	0.000139004	0.000102589	348/712/728/6347/6348/6352/10752/11151/1630/112399/1958/2213/2729/10457/2904/3134/3162/3684/3689/3725/8564/120892/4804/22861/4929/8013/26471/5063/10891/54463/6622/6648/6812/7020/7068/10333/8626/7305	38
BP	GO:0002287	alpha-beta T cell activation involved in immune response	13/926	61/17913	1.13E-05	0.000146275	0.000107954	301/6363/941/942/8320/1880/3594/3569/3662/3718/4063/114548/114836	13
BP	GO:0002293	alpha-beta T cell differentiation involved in immune	13/926	61/17913	1.13E-05	0.00014627	0.000107954	301/6363/941/942/8320/1880/3594/3569/3662/3718/4063/114548/114836	13

© 2020 Yu Y et al. *JAMA Network Open.*

		response				5			
BP	GO:0071902	positive regulation of protein serine/threonine kinase activity	36/926	322/17913	1.14E-05	0.000148391	0.000109517	150/728/6363/894/972/57214/1436/7852/1906/10595/54757/2322/2357/8091/3481/3553/23566/120892/4057/11184/64127/3084/5027/5063/5294/23533/5788/10125/5979/6091/6622/10333/8600/23043/7422/9839	36
BP	GO:0007162	negative regulation of cell adhesion	32/926	271/17913	1.15E-05	0.000148651	0.000109709	27299/84830/301/384/6366/29126/972/999/1493/1843/2213/2633/2651/10457/84868/3620/3559/3718/3902/54900/10859/10288/4321/80380/5140/5365/26191/5788/5800/9750/57556/11326	32
BP	GO:0002643	regulation of tolerance induction	7/926	17/17913	1.19E-05	0.000152807	0.000112776	29126/916/84868/3620/3559/11213/10288	7
BP	GO:1903980	positive regulation of microglial cell activation	7/926	17/17913	1.19E-05	0.000152807	0.000112776	712/6348/3684/3689/120892/26228/54209	7
BP	GO:0032637	interleukin-8 production	15/926	80/17913	1.27E-05	0.000163049	0.000120335	301/929/914/1116/2219/3304/3553/64127/26191/5788/7096/7097/10333/51284/51311	15
BP	GO:0045861	negative regulation of proteolysis	36/926	324/17913	1.31E-05	0.000167998	0.000123987	653145/274/718/720/721/135228/939/960/340267/1285/1378/1476/1800/3428/2537/7850/27074/4018/120892/4057/4318/4804/89932/51050/5266/5329/5272/710/6622/11005/9806/50859/7035/7980/7422/11326	36

© 2020 Yu Y et al. *JAMA Network Open.*

BP	GO:0002279	mast cell activation involved in immune response	11/926	45/17913	1.33E-05	0.000169863	0.000125363	30817/695/8832/2207/3162/8013/5293/5294/5880/10125/6812	11
BP	GO:0002820	negative regulation of adaptive immune response	11/926	45/17913	1.33E-05	0.000169863	0.000125363	1378/2213/84868/3134/9173/90865/3575/3718/10859/5788/64092	11
BP	GO:0002495	antigen processing and presentation of peptide antigen via MHC class II	17/926	100/17913	1.34E-05	0.000170554	0.000125873	972/1514/2213/3108/3109/3112/3113/3115/3118/3119/3120/3122/3123/3125/10437/3902/54209	17
BP	GO:1903556	negative regulation of tumor necrosis factor superfamily cytokine production	14/926	71/17913	1.35E-05	0.000170823	0.000126072	54/384/29126/10457/84868/11213/10859/4057/197358/26191/140885/6504/7128/54209	14
BP	GO:0002504	antigen processing and presentation of peptide or polysaccharide antigen via MHC class II	17/926	101/17913	1.54E-05	0.000194375	0.000143454	972/1514/2213/3108/3109/3112/3113/3115/3118/3119/3120/3122/3123/3125/10437/3902/54209	17

© 2020 Yu Y et al. *JAMA Network Open.*

BP	GO:0032677	regulation of interleukin-8 production	14/926	72/17913	1.59E-05	0.000201087	0.000148408	301/929/914/2219/3304/3553/64127/26191/5788/7096/7097/10333/51284/51311	14
BP	GO:0046637	regulation of alpha-beta T cell differentiation	13/926	63/17913	1.63E-05	0.000205051	0.000151333	100/301/6363/941/942/3594/3662/3718/3071/114548/639/54440/7535	13
BP	GO:0070231	T cell apoptotic process	11/926	46/17913	1.67E-05	0.000209745	0.000154798	100/384/6352/939/29126/3620/3559/3575/3718/5588/1831	11
BP	GO:0032897	negative regulation of viral transcription	8/926	24/17913	1.73E-05	0.00021696	0.000160122	6348/6351/6352/8091/10410/3725/11074/7538	8
BP	GO:0071347	cellular response to interleukin-1	22/926	156/17913	1.76E-05	0.000219454	0.000161963	27063/6347/6348/6352/1116/3576/1906/1958/2633/3383/3553/7850/3556/3569/11213/8564/64127/5696/5698/140885/6662/7035	22
BP	GO:0002675	positive regulation of acute inflammatory response	9/926	31/17913	1.82E-05	0.000227032	0.000167556	695/718/1236/2207/3553/3569/9180/5294/8600	9
BP	GO:0001911	negative regulation of	7/926	18/17913	1.86	0.00	0.000169434	2213/84868/3134/3575/10859/5788/5272	7

© 2020 Yu Y et al. *JAMA Network Open.*

		leukocyte mediated cytotoxicity			E-05	0229578			
BP	GO:0032693	negative regulation of interleukin-10 production	7/926	18/17913	1.86E-05	0.000229578	0.000169434	29126/2213/3620/3718/10859/80380/7305	7
BP	GO:0098581	detection of external biotic stimulus	7/926	18/17913	1.86E-05	0.000229578	0.000169434	721/23643/64127/7096/7097/10333/54209	7
BP	GO:0007249	I-kappaB kinase/NF-kappaB signaling	30/926	253/17913	1.97E-05	0.000243257	0.00017953	330/695/6363/6366/1236/929/948/972/2099/51704/3162/3553/4057/23643/197358/64127/5187/11040/5579/6398/140885/7052/7097/10333/51284/51311/7128/8600/10346/10537	30
BP	GO:0006836	neurotransmitter transport	29/926	241/17913	2.05E-05	0.000251707	0.000185766	100/107/321/8862/8938/695/1906/2207/8564/10859/120892/4129/79772/5027/8497/55607/5579/5997/6507/6581/6538/6535/6622/9627/6812/91683/7054/8600/9256	29
BP	GO:0072507	divalent inorganic cation homeostasis	42/926	412/17913	2.15E-05	0.000263875	0.000194747	348/719/6357/6363/6366/6348/6352/729230/1236/930/948/952/1043/57214/11151/3627/6373/10563/4283/1906/2099/54757/2903/2904/3792/3932/23566/931/5027/5031/5294/5579/5788/22821/79608/6279/6556/6622/7052/147138/8600/9256	42
BP	GO:1901222	regulation of NIK/NF-kappaB	18/926	114/17913	2.16E-	0.0002	0.000194747	222487/655/6363/929/939/1116/1906/84868/3553/197358/114548/64127/4306/26191/7097/10333/51284/54209	18

		signaling			05	63875			
BP	GO:0002455	humoral immune response mediated by circulating immunoglobulin	17/926	104/17913	2.28E-05	0.000278944	0.000205868	712/713/714/717/718/720/721/730/3426/1378/1380/2208/2213/3119/5788/710/55061	17
BP	GO:0007204	positive regulation of cytosolic calcium ion concentration	30/926	255/17913	2.30E-05	0.000279802	0.000206501	719/6363/6366/6348/1236/930/948/952/1043/57214/11151/3627/6373/10563/4283/1906/2099/2903/2904/3932/23566/931/5027/5031/5294/5788/22821/79608/6622/7052	30
BP	GO:0032733	positive regulation of interleukin-10 production	9/926	32/17913	2.42E-05	0.000294234	0.000217153	29126/940/2207/3662/353514/64127/54440/7097/54209	9
BP	GO:0090025	regulation of monocyte chemotaxis	8/926	25/17913	2.43E-05	0.000294474	0.000217329	199/6352/729230/3627/1843/2358/7941/56833	8
BP	GO:2000482	regulation of interleukin-8 secretion	8/926	25/17913	2.43E-05	0.000294474	0.000217329	301/929/914/2219/26191/7096/7097/10333	8
BP	GO:0072593	reactive oxygen species	31/926	269/17913	2.54E-	0.0003	0.000225926	54/575/199/384/655/948/1536/1906/2358/3099/3383/2537/3553/3684/3689/120892/4129/653361/4688/9722/5027/5806/5880/140885/6622/6648/7020/7097/10333/7305/7409	31

© 2020 Yu Y et al. *JAMA Network Open.*

		metabolic process			05	06122			
BP	GO:0001505	regulation of neurotransmitter levels	35/926	321/17913	2.57E-05	0.000309332	0.000228295	54/107/199/321/384/8938/948/1806/1906/2903/3383/3553/3689/8564/120892/4129/79772/9722/5027/8497/55607/5579/5806/5997/140885/6507/6581/6622/9627/6812/91683/7054/7097/10333/9256	35
BP	GO:0002686	negative regulation of leukocyte migration	11/926	48/17913	2.58E-05	0.000309332	0.000228295	100/84830/6347/6366/1843/3162/90865/11240/9750/56833/26228	11
BP	GO:0051090	regulation of DNA-binding transcription factor activity	43/926	429/17913	2.59E-05	0.000310401	0.000229084	107/222487/9447/306/655/695/948/1906/8320/2099/84868/3055/3059/3162/3304/3383/3553/3556/3569/11213/3689/3725/4057/150372/197358/84166/114548/64127/26471/10891/5579/5588/5649/54101/6279/6446/7097/10333/7128/8600/10346/11074/7422	43
BP	GO:0002823	negative regulation of adaptive immune response based on somatic recombination of immune receptors built from immunoglobulin superfamily domains	10/926	40/17913	2.66E-05	0.000318488	0.000235052	1378/2213/84868/3134/9173/90865/3575/3718/10859/5788	10

BP	GO:0009410	response to xenobiotic stimulus	31/926	270/17913	2.73E-05	0.000325 28	0.000240065	5244/100/199/7852/56603/1562/1557/1573/1577/1800/1906/1958/2327/2328/2729/2903/9734/3359/3383/64757/4929/10891/5577/5742/26575/5997/6622/6799/27284/6999/7054	31
BP	GO:0002292	T cell differentiation involved in immune response	13/926	66/17913	2.76E-05	0.000328202	0.000242221	301/6363/941/942/8320/1880/3594/3569/3662/3718/4063/114548/114836	13
BP	GO:0002827	positive regulation of T-helper 1 type immune response	7/926	19/17913	2.81E-05	0.000332698	0.00024554	301/6363/729230/941/3594/3553/6556	7
BP	GO:0002922	positive regulation of humoral immune response	7/926	19/17913	2.81E-05	0.000332698	0.00024554	718/1236/2208/2213/3553/5650/5788	7
BP	GO:0042554	superoxide anion generation	9/926	33/17913	3.18E-05	0.000375328	0.000277002	54/1536/1906/2358/3684/3689/653361/4688/7305	9
BP	GO:0042093	T-helper cell differentiation	12/926	58/17913	3.34E-05	0.000393667	0.000290536	301/6363/941/942/1880/3594/3569/3662/3718/4063/114548/114836	12
BP	GO:0051346	negative regulation of	42/926	420/17913	3.39	0.00	0.000293839	653145/341/274/718/720/721/135228/939/960/340267/1285/1378/1476/1800/3001/9734/3428/2537/3434/10788/27074/4	42

© 2020 Yu Y et al. *JAMA Network Open.*

		hydrolase activity			E-05	0398142		018/120892/4057/4318/3071/4804/89932/51050/5266/5329/5365/5806/5997/5272/6622/11005/9806/50859/7035/7980/7422	
BP	GO:0019886	antigen processing and presentation of exogenous peptide antigen via MHC class II	16/926	97/17913	3.52E-05	0.000412707	0.000304588	972/1514/2213/3108/3109/3112/3113/3115/3118/3119/3120/3122/3123/3125/10437/3902	16
BP	GO:1901652	response to peptide	47/926	491/17913	3.58E-05	0.000418821	0.000309101	107/113/374/655/948/1536/1906/1958/1959/2069/2213/2358/2729/2650/9734/3383/3481/3553/3676/3718/688/4023/4321/4318/51237/4804/22861/64127/3164/4929/8013/5140/5251/5577/5579/5588/26191/143686/6513/7031/7054/7097/10333/7128/54209/7253/7412	47
BP	GO:1901214	regulation of neuron death	34/926	313/17913	3.61E-05	0.000421683	0.000311213	348/712/728/6347/6348/6352/10752/11151/1630/112399/1958/2213/2729/10457/2904/3134/3162/3684/3689/3725/8564/120892/4929/8013/26471/5063/10891/54463/6622/6648/6812/7020/10333/7305	34
BP	GO:0006898	receptor-mediated endocytosis	33/926	301/17913	3.89E-05	0.000453483	0.000334682	8862/341/348/718/6363/6366/929/9332/948/8029/3576/3577/1601/9829/2207/2209/2213/2350/2358/3676/3684/3689/3512/10859/8685/4360/4481/6401/9892/6622/23166/55576/7422	33
BP	GO:0044546	NLRP3 inflammasome complex assembly	6/926	14/17913	3.94E-05	0.000445815	0.000338128	948/115362/4210/197358/114548/10333	6

© 2020 Yu Y et al. *JAMA Network Open.*

						3			
BP	GO:0042267	natural killer cell mediated cytotoxicity	12/926	59/17913	4.00E-05	0.000463649	0.000342185	11151/3002/84868/3134/3902/10859/10125/5272/4068/114836/57823/7409	12
BP	GO:0002438	acute inflammatory response to antigenic stimulus	7/926	20/17913	4.13E-05	0.000475722	0.000351095	695/718/1236/923/2207/2213/3383	7
BP	GO:0033630	positive regulation of cell adhesion mediated by integrin	7/926	20/17913	4.13E-05	0.000475722	0.000351095	6366/6352/916/10563/55612/3071/5979	7
BP	GO:0034505	tooth mineralization	7/926	20/17913	4.13E-05	0.000475722	0.000351095	249/54757/5818/152816/6899/7020/256764	7
BP	GO:0002715	regulation of natural killer cell mediated immunity	10/926	42/17913	4.21E-05	0.000483826	0.000357076	10225/84868/3134/3902/10859/10125/5272/4068/114836/7409	10
BP	GO:0045765	regulation of angiogenesis	39/926	383/17913	4.30E-05	0.000493157	0.000363963	56999/575/10000/301/306/718/719/728/147372/729230/1116/1285/3627/10563/3576/7852/1536/5168/2152/10457/9734/3099/8091/3162/3553/3569/3687/3689/3696/4804/5140/26051/5579/57556/11005/23166/7128/7422/7453	39

© 2020 Yu Y et al. *JAMA Network Open.*

BP	GO:0019079	viral genome replication	18/926	120/17913	4.37E-05	0.000499 67	0.000368769	6347/6352/30835/940/10332/3576/10663/8091/3428/3429/3434/8519/10581/10410/4057/4599/8638/91543	18
BP	GO:1901342	regulation of vasculature development	42/926	425/17913	4.46E-05	0.000508652	0.000375398	56999/575/10000/301/306/655/718/719/728/147372/729230/1116/1285/3627/10563/3576/7852/1536/1958/5168/2152/10457/9734/3099/8091/3162/3553/3569/3687/3689/3696/4804/5140/26051/5579/57556/6648/11005/23166/7128/7422/7453	42
BP	GO:0008361	regulation of cell size	21/926	154/17913	4.50E-05	0.00051189	0.000377788	10000/348/1630/1906/23105/3575/3792/3897/23566/4478/4897/9423/10439/5027/5341/5979/10371/375611/6696/7409/7422	21
BP	GO:0031214	biomineral tissue development	21/926	154/17913	4.50E-05	0.00051189	0.000377788	84059/249/655/6348/54757/2201/2261/10457/4057/5818/4745/152816/5027/5251/6662/6696/5552/6899/7020/7286/256764	21
BP	GO:0001782	B cell homeostasis	8/926	27/17913	4.56E-05	0.000515472	0.000380431	100/972/55619/139818/3071/124976/7128/10673	8
BP	GO:0035456	response to interferon-beta	8/926	27/17913	4.56E-05	0.000515472	0.000380431	9447/3428/8519/10581/10410/4332/149628/54739	8
BP	GO:0001894	tissue homeostasis	26/926	214/17913	4.57E-05	0.00051 6	0.000381129	84059/10000/729230/952/11151/1436/8029/1525/2651/53832/3569/3512/4057/4069/4117/4583/64127/5027/5880/54440/6662/7128/4982/8600/7399/7422	26

© 2020 Yu Y et al. *JAMA Network Open.*

						417			
BP	GO:0002294	CD4-positive, alpha-beta T cell differentiation involved in immune response	12/926	60/17913	4.76E-05	0.000536655	0.000396065	301/6363/941/942/1880/3594/3569/3662/3718/4063/114548/114836	12
BP	GO:0050871	positive regulation of B cell activation	13/926	70/17913	5.29E-05	0.000594254	0.000438575	100/695/939/940/952/972/115352/1880/3569/3071/64127/5788/10673	13
BP	GO:0050792	regulation of viral process	25/926	204/17913	5.51E-05	0.000618392	0.000456389	348/6348/6351/6352/940/972/3576/7852/2219/8091/3428/3429/3434/8519/10581/10410/3725/27074/4057/4599/8638/5806/91543/11074/7538	25
BP	GO:0055074	calcium ion homeostasis	39/926	388/17913	5.72E-05	0.000639628	0.000472062	348/719/6357/6363/6366/6348/6352/729230/1236/930/948/952/1043/57214/11151/3627/6373/10563/4283/1906/2099/54757/2903/2904/3792/3932/23566/931/5027/5031/5294/5579/5788/22821/79608/6622/7052/8600/9256	39
BP	GO:0048871	multicellular organismal homeostasis	45/926	472/17913	5.74E-05	0.000641193	0.000473217	107/113/84059/10000/216/8862/729230/948/952/11151/1436/8029/1525/7852/56603/1958/2171/2651/3553/53832/3569/3662/3512/4057/4069/4117/4583/64127/8013/5027/51316/23228/10891/5577/5880/54440/6556/6662/7128/4982/8600/8626/7253/7399/7422	45
BP	GO:0035455	response to interferon-alpha	7/926	21/17913	5.92E-	0.0006	0.000486628	3433/3437/8519/10581/10410/27074/149628	7

© 2020 Yu Y et al. *JAMA Network Open.*

					05	59365			
BP	GO:0050869	negative regulation of B cell activation	8/926	28/17913	6.09E-05	0.000675569	0.000498587	55024/695/1493/2213/4332/64092/7128/7305	8
BP	GO:2000406	positive regulation of T cell migration	8/926	28/17913	6.09E-05	0.000675569	0.000498587	199/6364/6366/6352/729230/3627/10563/3676	8
BP	GO:0022408	negative regulation of cell-cell adhesion	22/926	169/17913	6.13E-05	0.000678571	0.000500803	84830/301/384/6366/29126/972/999/1493/2213/10457/84868/3620/3559/3718/3902/54900/10859/10288/80380/26191/9750/11326	22
BP	GO:0002437	inflammatory response to antigenic stimulus	10/926	44/17913	6.46E-05	0.000712006	0.000525479	695/718/1236/940/923/2207/2213/3383/3559/10125	10
BP	GO:0010043	response to zinc ion	10/926	44/17913	6.46E-05	0.000712006	0.000525479	1396/4489/4493/4499/4501/4502/5027/6279/7054/7412	10
BP	GO:0071241	cellular response to inorganic substance	24/926	194/17913	6.63E-05	0.00072[illegible]	0.000538221	107/113/84059/241/999/1536/1800/1906/3162/3725/120892/4318/4489/4493/4499/4501/4502/653361/10891/10235/6331/6622/7020/7054	24

© 2020 Yu Y et al. *JAMA Network Open.*

						272			
BP	GO:0090218	positive regulation of lipid kinase activity	9/926	36/17913	6.74E-05	0.000738134	0.000544762	6363/6366/1236/930/1917/2261/2322/2358/64127	9
BP	GO:2000516	positive regulation of CD4-positive, alpha-beta T cell activation	9/926	36/17913	6.74E-05	0.000738134	0.000544762	301/6363/941/942/3594/3071/114548/5588/54440	9
BP	GO:0046330	positive regulation of JNK cascade	18/926	124/17913	6.79E-05	0.000741792	0.000547461	6363/6366/1236/939/1906/10595/2213/3553/11184/653361/64127/10125/10371/6504/10333/8600/23043/9839	18
BP	GO:0042130	negative regulation of T cell proliferation	11/926	53/17913	6.86E-05	0.000747439	0.00055163	384/29126/1493/10457/84868/3620/3559/10859/10288/80380/11326	11
BP	GO:0043903	regulation of symbiosis, encompassing mutualism through parasitism	27/926	232/17913	6.99E-05	0.000759872	0.000560805	348/6348/6351/6352/940/948/972/3576/7852/2219/8091/3428/3429/3434/8519/10581/10410/3394/3725/27074/4057/4599/8638/5806/91543/11074/7538	27
BP	GO:0072503	cellular divalent inorganic	39/926	392/17913	7.14E-	0.0007	0.000572059	348/719/6357/6363/6366/6348/6352/729230/1236/930/948/952/1043/57214/11151/3627/6373/10563/4283/1906/2099/2903/2904/3792/3932/23566/931/5027/5031/5294/5579/5788/2	39

© 2020 Yu Y et al. *JAMA Network Open.*

		cation homeostasis			05	7512		2821/79608/6279/6556/6622/7052/9256	
BP	GO:0002645	positive regulation of tolerance induction	5/926	10/17913	7.40E-05	0.000796497	0.000587835	29126/916/3620/11213/10288	5
BP	GO:0070099	regulation of chemokine-mediated signaling pathway	5/926	10/17913	7.40E-05	0.000796497	0.000587835	6352/1906/11240/6091/54209	5
BP	GO:0098883	synapse pruning	5/926	10/17913	7.40E-05	0.000796497	0.000587835	712/713/714/718/3684	5
BP	GO:2000425	regulation of apoptotic cell clearance	5/926	10/17913	7.40E-05	0.000796497	0.000587835	717/718/720/721/6347	5
BP	GO:0050770	regulation of axonogenesis	20/926	148/17913	7.71E-05	0.000828494	0.00061145	348/1630/2048/23105/3792/3897/23566/4038/4804/4897/9423/10439/5800/5979/6091/10371/6696/347733/7422/9839	20
BP	GO:0035914	skeletal muscle cell differentiation	12/926	63/17913	7.88E-05	0.00080844	0.000622973	467/50937/56603/1958/1959/8320/2817/59269/688/26471/5079/6899	12

						10 7			
B P	GO:00 71675	regulation of mononuclea r cell migration	10/92 6	45/17913	7. 93 E- 05	0. 00 08 47 64 8	0.000625586	199/719/728/6352/729230/3627/1843/2358/7941/56833	1 0
B P	GO:19 01216	positive regulation of neuron death	15/92 6	93/17913	7. 99 E- 05	0. 00 08 52 71	0.000629322	712/6348/1958/2213/2904/3684/3689/3725/8564/26471/506 3/6622/7020/10333/7305	1 5
B P	GO:00 06636	unsaturated fatty acid biosynthetic process	11/92 6	54/17913	8. 21 E- 05	0. 00 08 72 78	0.000644134	241/972/8560/1906/6785/2171/3553/5730/5742/79966/6916	1 1
B P	GO:19 04645	response to amyloid-bet a	11/92 6	54/17913	8. 21 E- 05	0. 00 08 72 78	0.000644134	948/2213/2358/3383/3676/4321/4318/4804/10333/54209/74 12	1 1
B P	GO:00 31342	negative regulation of cell killing	7/926	22/17913	8. 29 E- 05	0. 00 08 79 62 3	0.000649185	2213/84868/3134/3575/10859/5788/5272	7
B P	GO:00 97553	calcium ion transmembr ane import into cytosol	18/92 6	126/1791 3	8. 40 E- 05	0. 00 08 88 72 1	0.000655899	6363/6366/6348/1236/930/57214/11151/3627/6373/4283/29 03/2904/3932/5027/5031/5788/22821/6622	1 8
B	GO:00	positive	20/92	149/1791	8.	0.	0.000661435	9447/695/948/3304/3383/3553/3556/11213/3689/4057/1145	2

© 2020 Yu Y et al. *JAMA Network Open.*

P	51092	regulation of NF-kappaB transcription factor activity	6	3	49E-05	000896223		48/64127/5579/5588/54101/6279/7097/10333/8600/10346	0
BP	GO:0070613	regulation of protein processing	22/926	173/17913	8.73E-05	0.000918201	0.000677656	712/713/714/717/718/719/720/721/728/730/147372/930/629/3426/1378/1380/3553/7850/120892/710/55061/11326	22
BP	GO:1903034	regulation of response to wounding	22/926	173/17913	8.73E-05	0.000918201	0.000677656	150/84830/301/348/135228/948/7852/1906/83481/2152/2811/1839/5329/5341/5588/56963/6403/710/89790/6696/7035/7128	22
BP	GO:0060191	regulation of lipase activity	15/926	94/17913	9.06E-05	0.000950918	0.000701802	653145/341/728/6352/2099/2261/9734/3702/4023/5031/5330/5997/6401/6622/7294	15
BP	GO:0006875	cellular metal ion homeostasis	44/926	467/17913	9.10E-05	0.000952669	0.000703094	348/719/730/6357/6363/6366/6348/6352/729230/1236/930/948/952/1043/57214/11151/3627/6373/10563/4283/1906/2099/2903/2904/3162/3792/3932/23566/4057/931/4502/5027/5031/5294/5579/5788/22821/79608/6279/6556/6622/7052/64699/9256	44
BP	GO:0050678	regulation of epithelial cell proliferation	37/926	369/17913	9.24E-05	0.000965247	0.000712377	10000/8862/348/374/728/6347/6352/135228/1021/1749/1960/83481/2069/2152/3162/3676/120425/3725/4321/4781/4804/197358/64127/3164/8013/26471/5365/26051/6091/6331/5268/6662/6899/7128/8626/7422/7538	37
BP	GO:0050673	epithelial cell	41/926	425/17913	9.36	0.00	0.000720129	10000/8862/348/374/728/6347/6352/135228/1021/1749/1960/83481/2069/2099/2152/55612/3162/3664/3676/120425/37	41

© 2020 Yu Y et al. *JAMA Network Open.*

		proliferation			E-05	0975751		25/4321/4781/4804/197358/64127/3164/8013/26471/5365/26051/6091/6331/5268/6662/6899/7128/8600/8626/7422/7538	
BP	GO:2001057	reactive nitrogen species metabolic process	14/926	84/17913	9.52E-05	0.000990607	0.000731093	54/199/384/948/1906/3383/3553/3689/64757/9722/5806/140885/7097/10333	14
BP	GO:0021675	nerve development	13/926	74/17913	9.63E-05	0.000999702	0.000737806	1959/2048/3213/3676/26018/89797/5979/10371/6507/84189/6899/7020/7412	13
BP	GO:0032757	positive regulation of interleukin-8 production	10/926	46/17913	9.66E-05	0.000999702	0.000737806	929/914/2219/3304/3553/64127/7096/7097/51284/51311	10
BP	GO:1900015	regulation of cytokine production involved in inflammatory response	10/926	46/17913	9.66E-05	0.000999702	0.000737806	113/923/115362/7850/4210/197358/64127/5187/140885/10333	10
BP	GO:0051480	regulation of cytosolic calcium ion concentration	31/926	289/17913	9.93E-05	0.00102387	0.000755642	719/6363/6366/6348/1236/930/948/952/1043/57214/11151/3627/6373/10563/4283/1906/2099/2903/2904/3932/23566/931/5027/5031/5294/5788/22821/79608/6622/7052/9256	31
BP	GO:0050679	positive regulation of epithelial cell	24/926	199/17913	9.94E-05	0.001023	0.000755642	10000/8862/374/728/6352/1749/1960/2152/3162/3676/120425/3725/4321/64127/3164/8013/5365/26051/6331/6662/6899/7128/8626/7422	24

© 2020 Yu Y et al. *JAMA Network Open.*

		proliferation				87			
BP	GO:0043406	positive regulation of MAP kinase activity	28/926	250/17913	0.000101413	0.001042722	0.000769556	150/728/6363/972/7852/1906/10595/2322/2357/3553/23566/120892/11184/64127/3084/5027/5063/5294/23533/5788/10125/5979/6091/10333/8600/23043/7422/9839	28
BP	GO:0002576	platelet degranulation	18/926	128/17913	0.000103328	0.00105812	0.00078092	135228/948/1521/2207/83706/3481/22915/5341/440915/5874/6414/6403/5265/710/5552/6812/7273/7422	18
BP	GO:0007498	mesoderm development	18/926	128/17913	0.000103328	0.00105812	0.00078092	655/660/695/8320/3055/8091/10320/22806/3675/3691/8013/5017/27330/6398/6899/8463/8626/7422	18
BP	GO:1903317	regulation of protein maturation	22/926	175/17913	0.000103668	0.001059458	0.000781908	712/713/714/717/718/719/720/721/728/730/147372/930/629/3426/1378/1380/3553/7850/120892/710/55061/11326	22
BP	GO:0043372	positive regulation of CD4-positive, alpha-beta T cell differentiation	8/926	30/17913	0.0001046	0.001062559	0.000784196	301/6363/941/942/3594/3071/114548/54440	8
BP	GO:0046633	alpha-beta T cell proliferation	8/926	30/17913	0.000104	0.001062	0.000784196	384/729230/29126/940/916/1794/5788/7535	8

© 2020 Yu Y et al. *JAMA Network Open.*

					6	559			
BP	GO:0061082	myeloid leukocyte cytokine production	8/926	30/17913	0.0001046	0.00106255 9	0.000784196	948/972/2207/51704/3162/11213/10859/8013	8
BP	GO:0002707	negative regulation of lymphocyte mediated immunity	9/926	38/17913	0.00010636 5	0.00107454 7	0.000793043	10225/1378/2213/84868/3134/3575/10859/5788/5272	9
BP	GO:0002711	positive regulation of T cell mediated immunity	9/926	38/17913	0.00010636 5	0.00107454 7	0.000793043	3134/3594/3553/3569/114548/5027/5788/91543/54440	9
BP	GO:0009636	response to toxic substance	46/926	499/17913	0.00010641 6	0.00107454 7	0.000793043	100/113/241/348/374/655/6348/6351/6352/929/939/948/999/1536/1843/1906/1958/2903/2904/9734/3162/3359/3383/3569/3725/3775/120892/4129/64757/4929/8013/26471/10891/55607/5742/26575/5997/6279/140885/6648/6688/7054/7097/7128/140803/7412	46
BP	GO:0006801	superoxide metabolic process	12/926	65/17913	0.00010811 1	0.00108949 7	0.000804077	54/655/948/1536/1906/2358/3684/3689/653361/4688/6648/7305	12
BP	GO:0042475	odontogenesis of dentin-containing tooth	14/926	85/17913	0.00010860	0.00109012	0.000804542	655/1441/54757/3691/4038/5818/152816/64065/860/6331/6899/4982/8626/256764	14

					4	7			
BP	GO:1903901	negative regulation of viral life cycle	14/926	85/17913	0.000108604	0.001090127	0.000804542	6352/2219/8091/3428/3434/8519/10581/10410/4057/4599/8638/5806/91543/11074	14
BP	GO:1901224	positive regulation of NIK/NF-kappaB signaling	13/926	75/17913	0.000111055	0.001112527	0.000821074	6363/929/939/1116/1906/84868/3553/64127/4306/7097/10333/51284/54209	13
BP	GO:0032703	negative regulation of interleukin-2 production	7/926	23/17913	0.000113942	0.001139192	0.000840753	2633/84868/3902/5788/7128/11326/7538	7
BP	GO:0051588	regulation of neurotransmitter transport	18/926	129/17913	0.000114397	0.001141489	0.000842449	107/321/8862/8938/8564/10859/120892/4129/79772/5027/55607/5579/5997/6622/9627/6812/91683/8600	18
BP	GO:0031334	positive regulation of protein complex assembly	27/926	239/17913	0.000115885	0.001154074	0.000851736	9447/274/146206/6366/1236/948/11151/10563/2099/342184/115362/3055/3304/3383/10788/3512/3725/3936/4478/3071/4868/5341/10333/129293/7422/7453/7454	27
BP	GO:0043370	regulation of CD4-positive, alpha-beta T cell differentiatio	10/926	47/17913	0.00011713	0.00116190 2	0.000857513	301/6363/941/942/3594/3662/3718/3071/114548/54440	10

		n							
BP	GO:0046638	positive regulation of alpha-beta T cell differentiation	10/926	47/17913	0.00011713	0.001161902	0.000857513	100/301/6363/941/942/3594/3071/114548/54440/7535	10
BP	GO:0061097	regulation of protein tyrosine kinase activity	14/926	86/17913	0.000123651	0.001224189	0.000903483	150/389658/6352/1436/2069/2209/51704/353514/653361/3084/5788/5649/80725/26228	14
BP	GO:0010517	regulation of phospholipase activity	12/926	66/17913	0.000126007	0.001245085	0.000918905	653145/728/6352/2099/2261/3702/5031/5330/5997/6401/6622/7294	12
BP	GO:0006874	cellular calcium ion homeostasis	37/926	375/17913	0.000128541	0.001267657	0.000935563	348/719/6357/6363/6366/6348/6352/729230/1236/930/948/952/1043/57214/11151/3627/6373/10563/4283/1906/2099/2903/2904/3792/3932/23566/931/5027/5031/5294/5579/5788/22821/79608/6622/7052/9256	37
BP	GO:0033089	positive regulation of T cell differentiation in thymus	5/926	11/17913	0.000129904	0.001273662	0.000939995	100/1960/3575/10125/9840	5
BP	GO:0045059	positive thymic T cell selection	5/926	11/17913	0.000129904	0.001273662	0.000939995	915/972/1794/5788/7535	5

© 2020 Yu Y et al. *JAMA Network Open.*

BP	GO:0072610	interleukin-12 secretion	5/926	11/17913	0.000129904	0.001273662	0.000939995	6363/1236/353514/10859/51311	5
BP	GO:0051928	positive regulation of calcium ion transport	17/926	119/17913	0.000131002	0.001281946	0.000946109	9914/6347/6348/6351/6352/930/57214/3627/6373/4283/353514/23566/5027/5031/6622/6769/342667	17
BP	GO:0042269	regulation of natural killer cell mediated cytotoxicity	9/926	39/17913	0.000131991	0.001289138	0.000951417	84868/3134/3902/10859/10125/5272/4068/114836/7409	9
BP	GO:0045920	negative regulation of exocytosis	8/926	31/17913	0.000134642	0.00131125	0.000968659	150/301/729230/8832/2213/3134/3162/6622	8
BP	GO:0061041	regulation of wound healing	19/926	143/17913	0.000144992	0.001410685	0.001041122	150/84830/301/348/135228/948/7852/1906/83481/2152/2811/1839/5329/5341/5588/6403/710/7035/7128	19
BP	GO:0050918	positive chemotaxis	12/926	67/17913	0.000146382	0.001421404	0.001049033	6348/6352/1233/11151/3627/3576/2152/2358/10457/3603/5365/7422	12
B	GO:00	immunoglob	15/92	98/17913	0.	0.	0.001049033	695/933/940/2213/115352/10875/3119/90865/3569/3575/94	1

© 2020 Yu Y et al. *JAMA Network Open.*

P	02377	ulin production	6		000146935	001421404		52/54900/51237/5788/10673	5
BP	GO:0042136	neurotransmitter biosynthetic process	15/926	98/17913	0.000146935	0.001421404	0.001049033	54/199/384/948/1806/1906/3383/3553/3689/9722/5806/140885/6507/7054/10333	15
BP	GO:0001914	regulation of T cell mediated cytotoxicity	7/926	24/17913	0.000153721	0.001484214	0.001095388	2213/3134/3594/3575/10859/5027/5788	7
BP	GO:1905954	positive regulation of lipid localization	13/926	78/17913	0.000167626	0.001615392	0.001192201	5244/348/10396/718/948/1906/29923/3553/4023/4481/5027/6696/8600	13
BP	GO:1903793	positive regulation of anion transport	10/926	49/17913	0.000169255	0.001627998	0.001201504	5243/5244/348/10396/1906/3553/8564/5027/6812/8600	10
BP	GO:0043552	positive regulation of phosphatidylinositol 3-kinase activity	8/926	32/17913	0.000171454	0.001642914	0.001212513	6363/6366/1236/930/2261/2322/2358/64127	8
BP	GO:0071276	cellular response to	8/926	32/17913	0.00	0.00	0.001212513	1536/3162/3725/4318/4489/4493/4501/653361	8

© 2020 Yu Y et al. *JAMA Network Open.*

		cadmium ion			0171454	1642914			
BP	GO:0046328	regulation of JNK cascade	21/926	169/17913	0.000174068	0.001664815	0.001228676	6363/6366/1236/939/1906/10595/2213/3553/11184/653361/64127/5187/26191/10125/10371/140885/6504/10333/8600/23043/9839	21
BP	GO:0060402	calcium ion transport into cytosol	19/926	145/17913	0.000174418	0.00166502	0.001228828	6363/6366/6348/1236/930/57214/11151/3627/6373/4283/2903/2904/3932/931/5027/5031/5788/22821/6622	19
BP	GO:0045071	negative regulation of viral genome replication	11/926	59/17913	0.000189636	0.001806887	0.001333529	6352/8091/3428/3434/8519/10581/10410/4057/4599/8638/91543	11
BP	GO:0099173	postsynapse organization	20/926	158/17913	0.00019155	0.001821698	0.00134446	348/22866/55619/2048/2213/2904/3556/9118/3675/10288/4038/120892/10397/4744/9722/4897/5063/55607/5649/80725	20
BP	GO:0032720	negative regulation of tumor necrosis factor production	12/926	69/17913	0.000195689	0.001857575	0.001370938	54/384/10457/84868/11213/10859/197358/26191/140885/6504/7128/54209	12
BP	GO:0010769	regulation of cell morphogen	28/926	260/17913	0.0001	0.0018	0.001384121	348/1630/2048/23105/2633/3792/3897/23566/4038/120892/4804/4897/9423/10439/5063/55607/5800/5649/5979/6091/6281/10371/6696/80725/23043/347733/7422/9839	28

© 2020 Yu Y et al. *JAMA Network Open.*

		esis involved in differentiation			9794	754438			
BP	GO:2000404	regulation of T cell migration	9/926	41/17913	0.000198848	0.000188059	0.001387871	199/6364/6366/6352/729230/3627/10563/3676/9750	9
BP	GO:0046677	response to antibiotic	33/926	328/17913	0.000203785	0.000192063	0.001417056	100/107/113/249/374/1233/929/939/4261/1396/1536/1843/1906/1958/2903/2904/3162/3359/3383/3569/3725/120892/4129/8013/5997/6279/140885/6507/375611/6688/7054/7128/7412	33
BP	GO:0090026	positive regulation of monocyte chemotaxis	6/926	18/17913	0.000203786	0.000192063	0.001417056	199/6352/729230/3627/2358/7941	6
BP	GO:0051209	release of sequestered calcium ion into cytosol	16/926	112/17913	0.00020456	0.0001920214	0.001417168	6363/6366/6348/1236/930/57214/11151/3627/6373/4283/3932/5027/5031/5788/22821/6622	16
BP	GO:0051283	negative regulation of sequestering of calcium ion	16/926	112/17913	0.00020456	0.0001920214	0.001417168	6363/6366/6348/1236/930/57214/11151/3627/6373/4283/3932/5027/5031/5788/22821/6622	16
BP	GO:2001056	positive regulation of cysteine-typ	18/926	135/17913	0.0002	0.0019	0.001420393	9447/1285/26999/112399/2152/355/2903/2904/3428/3932/4210/4804/22861/114548/64065/6091/6279/6622	18

© 2020 Yu Y et al. *JAMA Network Open.*

		e endopeptidase activity			05405	24585			
BP	GO:0050878	regulation of body fluid levels	45/926	499/17913	0.000207307	0.001938818	0.001430898	100/107/113/150/84830/653145/8862/348/948/56603/139189/139818/1906/2048/2152/2207/83706/2811/8356/3099/3569/3932/3937/22915/4939/5027/5029/5294/23533/5329/5341/5577/5579/5588/5800/6403/5265/710/6812/7035/7980/8626/7409/7422/7454	45
BP	GO:1903900	regulation of viral life cycle	19/926	147/17913	0.000208959	0.001950668	0.001439644	6352/940/972/3576/2219/8091/3428/3429/3434/8519/10581/10410/27074/4057/4599/8638/5806/91543/11074	19
BP	GO:0002517	T cell tolerance induction	5/926	12/17913	0.000213221	0.001961552	0.001447676	916/29851/3620/3559/10288	5
BP	GO:0002863	positive regulation of inflammatory response to antigenic stimulus	5/926	12/17913	0.000213221	0.001961552	0.001447676	695/718/1236/940/2207	5
BP	GO:0043301	negative regulation of leukocyte degranulation	5/926	12/17913	0.000213221	0.001961552	0.001447676	729230/8832/2213/3134/3162	5
BP	GO:0043922	negative regulation by host of viral	5/926	12/17913	0.00021 3	0.001961	0.001447676	6348/6351/6352/8091/3725	5

		transcription			221	552			
BP	GO:0045410	positive regulation of interleukin-6 biosynthetic process	5/926	12/17913	0.000213221	0.000196152	0.001447676	2069/3553/7096/10333/7305	5
BP	GO:0045779	negative regulation of bone resorption	5/926	12/17913	0.000213221	0.000196152	0.001447676	952/3569/5027/7128/4982	5
BP	GO:0072540	T-helper 17 cell lineage commitment	5/926	12/17913	0.000213221	0.000196152	0.001447676	3594/3569/3662/4063/114836	5
BP	GO:2001198	regulation of dendritic cell differentiation	5/926	12/17913	0.000213221	0.000196152	0.001447676	2213/10859/10288/55365/28959	5
BP	GO:0001913	T cell mediated cytotoxicity	8/926	33/17913	0.000216155	0.000198494	0.00146494	2213/3134/3594/3575/10859/5027/5551/5788	8
BP	GO:0097366	response to bronchodilator	10/926	51/17913	0.00023964	0.00219665	0.001621186	6363/1236/1800/2903/9734/3383/4929/26575/5997/7054	10

© 2020 Yu Y et al. *JAMA Network Open.*

					3	3			
BP	GO:0007596	blood coagulation	33/926	331/17913	0.000241059	0.002202634	0.001625601	150/84830/653145/348/948/139189/139818/1906/2152/2207/83706/2811/8356/3569/3932/3937/22915/5027/5294/23533/5329/5341/5577/5579/5588/6403/5265/710/6812/7035/7980/7409/7454	33
BP	GO:0045429	positive regulation of nitric oxide biosynthetic process	9/926	42/17913	0.000241599	0.002202634	0.001625601	199/948/1906/3383/3553/3689/9722/5806/10333	9
BP	GO:1900271	regulation of long-term synaptic potentiation	9/926	42/17913	0.000241599	0.002202634	0.001625601	107/348/2048/2903/10288/27065/55607/5649/7305	9
BP	GO:0046209	nitric oxide metabolic process	13/926	81/17913	0.000247353	0.002251046	0.00166133	54/199/384/948/1906/3383/3553/3689/9722/5806/140885/7097/10333	13
BP	GO:0051282	regulation of sequestering of calcium ion	16/926	114/17913	0.000251742	0.002286881	0.001687777	6363/6366/6348/1236/930/57214/11151/3627/6373/4283/3932/5027/5031/5788/22821/6622	16
BP	GO:0007263	nitric oxide mediated signal transduction	7/926	26/17913	0.000266838	0.002411051	0.001779418	348/948/2357/4502/9722/51655/7422	7

© 2020 Yu Y et al. *JAMA Network Open.*

BP	GO:0060142	regulation of syncytium formation by plasma membrane fusion	7/926	26/17913	0.000266838	0.002411051	0.001779418	575/963/3627/4283/9750/54209/7305	7
BP	GO:0060706	cell differentiation involved in embryonic placenta development	7/926	26/17913	0.000266838	0.002411051	0.001779418	79733/8320/2709/79977/3856/639/9021	7
BP	GO:0034142	toll-like receptor 4 signaling pathway	8/926	34/17913	0.000269977	0.00243508	0.001797152	929/3684/3689/4057/23643/118788/26191/7128	8
BP	GO:0002313	mature B cell differentiation involved in immune response	6/926	19/17913	0.000284877	0.002534007	0.001870162	100/55619/139818/2213/1880/9452	6
BP	GO:0002544	chronic inflammatory response	6/926	19/17913	0.000284877	0.002534007	0.001870162	6352/10563/3620/6279/7128/7412	6
BP	GO:0046597	negative regulation of viral entry into host cell	6/926	19/17913	0.000284877	0.002534007	0.001870162	2219/8519/10581/10410/5806/11074	6

© 2020 Yu Y et al. *JAMA Network Open.*

BP	GO:0051767	nitric-oxide synthase biosynthetic process	6/926	19/17913	0.000284877	0.002534007	0.001870162	6347/1906/2208/120892/64127/7097	6
BP	GO:0051769	regulation of nitric-oxide synthase biosynthetic process	6/926	19/17913	0.000284877	0.002534007	0.001870162	6347/1906/2208/120892/64127/7097	6
BP	GO:0061081	positive regulation of myeloid leukocyte cytokine production involved in immune response	6/926	19/17913	0.000284877	0.002534007	0.001870162	948/972/2207/51704/10859/8013	6
BP	GO:0140131	positive regulation of lymphocyte chemotaxis	6/926	19/17913	0.000284877	0.002534007	0.001870162	6366/6348/6351/6352/729230/10563	6
BP	GO:0035690	cellular response to drug	35/926	362/17913	0.000284945	0.002534007	0.001870162	107/113/122622/199/9447/969/999/7852/1536/1800/1906/1958/2350/2729/8091/3162/3383/3553/3569/120892/11184/4502/64127/8013/5027/5029/5142/10891/26191/140885/56833/6662/6688/7054/7128	35
BP	GO:0070588	calcium ion transmembrane transport	30/926	293/17913	0.00028[illegible]	0.002563[illegible]	0.001891661	150/309/274/777/6363/6366/6348/1236/930/57214/11151/3627/6373/4283/2903/2904/3932/9722/5027/5031/5142/5294/5788/22821/6236/6622/6769/342667/80036/140803	30

© 2020 Yu Y et al. *JAMA Network Open.*

					726	137			
BP	GO:0050850	positive regulation of calcium-mediated signaling	9/926	43/17913	0.000291 7	0.0025760 02	0.001901156	100/6348/6351/916/3084/5031/50852/54209/7535	9
BP	GO:1904407	positive regulation of nitric oxide metabolic process	9/926	43/17913	0.000291 7	0.0025760 02	0.001901156	199/948/1906/3383/3553/3689/9722/5806/10333	9
BP	GO:1904646	cellular response to amyloid-beta	9/926	43/17913	0.000291 7	0.0025760 02	0.001901156	948/2213/2358/3383/3676/4804/10333/54209/7412	9
BP	GO:0043550	regulation of lipid kinase activity	11/926	62/17913	0.00029898 5	0.0026357 51	0.001945252	6363/6366/1236/930/1917/2261/2322/2358/64127/23533/9021	11
BP	GO:0022612	gland morphogenesis	16/926	116/17913	0.00030806 8	0.0027111 05	0.002000865	374/655/1396/1436/2099/3400/284217/4478/4781/9423/10371/5268/6662/7052/7128/8626	16
BP	GO:0031589	cell-substrate adhesion	32/926	322/17913	0.00031501	0.00276740	0.002042417	56999/4059/6366/1236/948/916/960/10225/1021/11151/55612/83706/342184/2633/2651/3675/3676/3683/3689/3691/3897/4321/64005/64098/5800/5880/6281/6614/9806/80725/7412/7422	32

© 2020 Yu Y et al. *JAMA Network Open.*

					1	6			
BP	GO:0007599	hemostasis	33/926	336/17913	0.000316851	0.002778747	0.002050787	150/84830/653145/348/948/139189/139818/1906/2152/2207/83706/2811/8356/3569/3932/3937/22915/5027/5294/23533/5329/5341/5577/5579/5588/6403/5265/710/6812/7035/7980/7409/7454	33
BP	GO:0007229	integrin-mediated signaling pathway	14/926	94/17913	0.000323772	0.00283454	0.002091963	2207/55612/83706/3055/3675/3676/3680/3683/3684/3687/3689/5341/7305/7409	14
BP	GO:0016485	protein processing	30/926	295/17913	0.000324366	0.002834851	0.002092193	712/713/714/717/718/719/720/721/728/730/147372/930/629/3426/1378/1380/26999/2152/2903/3428/3553/7850/5653/120892/5027/64065/710/5552/55061/11326	30
BP	GO:0002864	regulation of acute inflammatory response to antigenic stimulus	5/926	13/17913	0.000331766	0.002862739	0.002112775	695/718/1236/2207/2213	5
BP	GO:0002921	negative regulation of humoral immune response	5/926	13/17913	0.000331766	0.002862739	0.002112775	1378/2213/710/11005/11326	5
BP	GO:1900225	regulation of NLRP3 inflammasome complex assembly	5/926	13/17913	0.000331766	0.002862739	0.002112775	948/115362/4210/197358/10333	5

© 2020 Yu Y et al. *JAMA Network Open.*

BP	GO:0010959	regulation of metal ion transport	35/926	365/17913	0.000332744	0.002862739	0.002112775	150/9914/274/6347/6348/6351/6352/930/8832/57214/11151/3627/6373/4283/7852/115352/3383/3603/3792/353514/10859/10288/23566/9722/5027/5031/5142/5187/5294/6236/6331/6622/6769/342667/54209	35
BP	GO:0000768	syncytium formation by plasma membrane fusion	10/926	53/17913	0.0003330337	0.002862739	0.002112775	575/135228/963/50937/3627/4283/4868/9750/54209/7305	10
BP	GO:0043551	regulation of phosphatidylinositol 3-kinase activity	10/926	53/17913	0.0003330337	0.002862739	0.002112775	6363/6366/1236/930/2261/2322/2358/64127/23533/9021	10
BP	GO:0140253	cell-cell fusion	10/926	53/17913	0.0003330337	0.002862739	0.002112775	575/135228/963/50937/3627/4283/4868/9750/54209/7305	10
BP	GO:0030224	monocyte differentiation	8/926	35/17913	0.0003342274	0.002862739	0.002112775	972/1021/1436/3428/133396/3725/8544/7422	8
BP	GO:0045066	regulatory T cell differentiation	8/926	35/17913	0.0003342274	0.002862739	0.002112775	940/1378/1493/3559/3902/10288/11006/26279	8
B	GO:00	regulation of	8/926	35/17913	0.	0.	0.002112775	301/6363/941/942/3594/3662/3718/114548	8

© 2020 Yu Y et al. *JAMA Network Open.*

P	45622	T-helper cell differentiatio n			00 03 34 27 4	00 28 62 73 9			
B P	GO:19 03131	mononuclea r cell differentiatio n	8/926	35/17913	0. 00 03 34 27 4	0. 00 28 62 73 9	0.002112775	972/1021/1436/3428/133396/3725/8544/7422	8
B P	GO:00 50817	coagulation	33/92 6	337/1791 3	0. 00 03 34 33 4	0. 00 28 62 73 9	0.002112775	150/84830/653145/348/948/139189/139818/1906/2152/2207 /83706/2811/8356/3569/3932/3937/22915/5027/5294/23533/ 5329/5341/5577/5579/5588/6403/5265/710/6812/7035/7980/ 7409/7454	3 3
B P	GO:00 50688	regulation of defense response to virus	12/92 6	73/17913	0. 00 03 37 66	0. 00 28 86 34	0.002130194	9447/330/55601/10875/51191/3434/3594/3553/3559/10859/ 4321/7128	1 2
B P	GO:00 14066	regulation of phosphatidy linositol 3-kinase signaling	16/92 6	117/1791 3	0. 00 03 40 09 8	0. 00 28 97 40 8	0.002138362	6352/940/2322/3059/653361/197358/118788/5293/5294/235 33/8395/26051/10125/5649/6403/6662	1 6
B P	GO:00 51208	sequesterin g of calcium ion	16/92 6	117/1791 3	0. 00 03 40 09 8	0. 00 28 97 40 8	0.002138362	6363/6366/6348/1236/930/57214/11151/3627/6373/4283/39 32/5027/5031/5788/22821/6622	1 6
B P	GO:00 46640	regulation of alpha-beta T cell	7/926	27/17913	0. 00 03	0. 00 29	0.00215383	384/729230/29126/940/916/5788/7535	7

© 2020 Yu Y et al. *JAMA Network Open.*

		proliferation			44286	18367			
BP	GO:0050858	negative regulation of antigen receptor-mediated signaling pathway	7/926	27/17913	0.000344286	0.00291836 7	0.00215383	933/2213/115352/2633/11006/23228/26191	7
BP	GO:0060512	prostate gland morphogenesis	7/926	27/17913	0.000344286	0.002918367	0.00215383	655/1396/2099/3400/5268/6662/8626	7
BP	GO:0045766	positive regulation of angiogenesis	23/926	203/17913	0.000346214	0.002929811	0.002162276	10000/301/306/718/719/728/147372/1116/3576/7852/1536/2152/9734/3099/8091/3162/3553/3687/3689/3696/26051/5579/7422	23
BP	GO:0043254	regulation of protein complex assembly	38/926	409/17913	0.000353716	0.002988307	0.002205448	119/9447/348/274/146206/6366/1236/948/11151/10563/2099/342184/115362/3055/3059/3304/3383/10788/3512/3725/3936/4210/4478/3071/197358/4868/5063/5341/55607/6403/6622/6812/10333/147138/129293/7422/7453/7454	38
BP	GO:0045069	regulation of viral genome replication	14/926	95/17913	0.000362009	0.003053283	0.002253401	6352/940/3576/8091/3428/3429/3434/8519/10581/10410/4057/4599/8638/91543	14
BP	GO:0034612	response to tumor necrosis	29/926	284/17913	0.0003	0.0031	0.002351464	3899/9447/27063/330/6347/6348/6352/929/939/1116/3576/1906/2633/3304/3383/3856/4050/10891/5696/5698/6401/7128/4982/23495/8600/10673/10537/7412/7538	29

		factor			78391	86154			
BP	GO:0030193	regulation of blood coagulation	12/926	74/17913	0.000384376	0.003225832	0.002380747	84830/348/948/1906/2152/2811/5329/5341/5588/6403/710/7035	12
BP	GO:0060291	long-term synaptic potentiation	12/926	74/17913	0.000384376	0.003225832	0.002380747	107/348/2048/2903/10288/27065/55607/5649/22941/6622/91683/7305	12
BP	GO:0038061	NIK/NF-kappaB signaling	21/926	179/17913	0.000385616	0.003230891	0.002384481	222487/330/655/6363/929/939/1116/1906/84868/3553/197358/114548/64127/4306/5696/5698/26191/7097/10333/51284/54209	21
BP	GO:0050807	regulation of synapse organization	24/926	218/17913	0.000398582	0.003334017	0.00246059	575/348/1006/2048/2213/2904/57549/3587/3556/10288/4038/120892/266727/5818/4897/9423/5063/55607/5800/5649/84189/6622/80725/7097	24
BP	GO:0042417	dopamine metabolic process	8/926	36/17913	0.000410518	0.003428193	0.002530095	2903/3684/3689/4129/4929/6622/9627/7054	8
BP	GO:0032874	positive regulation of stress-activated MAPK	19/926	155/17913	0.00041 4	0.003452	0.002548383	6363/6366/1236/939/1906/10595/2213/3553/11184/653361/64127/10125/10371/6504/10333/8600/23043/7422/9839	19

© 2020 Yu Y et al. *JAMA Network Open.*

		cascade			166	974			
BP	GO:0050922	negative regulation of chemotaxis	9/926	45/17913	0.000417801	0.003477556	0.002566526	199/6347/10563/1843/11240/5800/6091/56833/26228	9
BP	GO:1904018	positive regulation of vasculature development	25/926	232/17913	0.000421598	0.003503414	0.00258561	10000/301/306/718/719/728/147372/1116/3576/7852/1536/1958/2152/9734/3099/8091/3162/3553/3687/3689/3696/26051/5579/6648/7422	25
BP	GO:0051235	maintenance of location	29/926	286/17913	0.000424879	0.003524898	0.002601466	348/718/6363/6366/6348/1236/930/948/57214/11151/3627/6373/4283/23136/2201/29923/3099/3553/3569/3932/4023/4481/5027/5031/5788/22821/6279/6622/5552	29
BP	GO:1900046	regulation of hemostasis	12/926	75/17913	0.000436437	0.003604538	0.002660242	84830/348/948/1906/2152/2811/5329/5341/5588/6403/710/7035	12
BP	GO:0001516	prostaglandin biosynthetic process	7/926	28/17913	0.000438745	0.003604538	0.002660242	972/1906/2171/3553/5730/5742/6916	7
BP	GO:0001773	myeloid dendritic cell activation	7/926	28/17913	0.00043874	0.00360453	0.002660242	914/1794/84868/3662/6504/6688/10537	7

© 2020 Yu Y et al. *JAMA Network Open.*

					5	8			
BP	GO:0032814	regulation of natural killer cell activation	7/926	28/17913	0.000438745	0.003604538	0.002660242	84868/3134/639/26191/10125/9760/7305	7
BP	GO:0045577	regulation of B cell differentiation	7/926	28/17913	0.000438745	0.003604538	0.002660242	695/939/115352/22806/3071/150372/56833	7
BP	GO:0046457	prostanoid biosynthetic process	7/926	28/17913	0.000438745	0.003604538	0.002660242	972/1906/2171/3553/5730/5742/6916	7
BP	GO:0070304	positive regulation of stress-activated protein kinase signaling cascade	19/926	156/17913	0.000449288	0.003682647	0.002717889	6363/6366/1236/939/1906/10595/2213/3553/11184/653361/64127/10125/10371/6504/10333/8600/23043/7422/9839	19
BP	GO:0098742	cell-cell adhesion via plasma-membrane adhesion molecules	25/926	233/17913	0.000449706	0.003682647	0.002717889	57863/30835/923/8832/999/1952/1525/1824/1829/3383/3556/3683/3684/3689/120425/266727/5818/5365/5979/6091/54549/6401/6402/6403/7412	25
BP	GO:0006949	syncytium formation	10/926	55/17913	0.00045498	0.00371922	0.002744881	575/135228/963/50937/3627/4283/4868/9750/54209/7305	10

© 2020 Yu Y et al. *JAMA Network Open.*

					1	1			
BP	GO:0032872	regulation of stress-activated MAPK cascade	24/926	220/17913	0.000455639	0.003719221	0.002744881	6363/6366/1236/939/1843/1906/10595/355/2213/3553/11184/653361/64127/5187/26191/10125/10371/140885/6504/10333/8600/23043/7422/9839	24
BP	GO:0045428	regulation of nitric oxide biosynthetic process	11/926	65/17913	0.000457179	0.003725791	0.00274973	54/199/948/1906/3383/3553/3689/9722/5806/140885/10333	11
BP	GO:0060401	cytosolic calcium ion transport	19/926	157/17913	0.000486963	0.003962146	0.002924166	6363/6366/6348/1236/930/57214/11151/3627/6373/4283/2903/2904/3932/931/5027/5031/5788/22821/6622	19
BP	GO:0010935	regulation of macrophage cytokine production	5/926	14/17913	0.000494187	0.003971014	0.002930711	948/972/51704/11213/10859	5
BP	GO:0019883	antigen processing and presentation of endogenous antigen	5/926	14/17913	0.000494187	0.003971014	0.002930711	972/64167/3134/6890/6891	5
BP	GO:0030889	negative regulation of B cell proliferation	5/926	14/17913	0.00049418	0.00397101	0.002930711	695/1493/2213/4332/7305	5

© 2020 Yu Y et al. *JAMA Network Open.*

					7	4			
BP	GO:0046851	negative regulation of bone remodeling	5/926	14/17913	0.000494187	0.003971014	0.002930711	952/3569/5027/7128/4982	5
BP	GO:0050862	positive regulation of T cell receptor signaling pathway	5/926	14/17913	0.000494187	0.003971014	0.002930711	100/1236/3932/9840/50852	5
BP	GO:0051770	positive regulation of nitric-oxide synthase biosynthetic process	5/926	14/17913	0.000494187	0.003971014	0.002930711	6347/2208/120892/64127/7097	5
BP	GO:0070166	enamel mineralization	5/926	14/17913	0.000494187	0.003971014	0.002930711	54757/5818/152816/6899/256764	5
BP	GO:0006809	nitric oxide biosynthetic process	12/926	76/17913	0.000494932	0.003971014	0.002930711	54/199/384/948/1906/3383/3553/3689/9722/5806/140885/10333	12
BP	GO:0030041	actin filament polymerization	18/926	145/17913	0.000498998	0.003984425	0.002940608	119/199/274/146206/6366/1236/23242/11151/342184/3055/3059/3383/10788/3071/4868/5063/55607/7454	18

© 2020 Yu Y et al. *JAMA Network Open.*

BP	GO:0042133	neurotransmitter metabolic process	18/926	145/17913	0.000498998	0.003984425	0.002940608	54/199/384/948/1806/1906/2903/3383/3553/3689/4129/9722/5806/140885/6507/7054/7097/10333	18
BP	GO:0050806	positive regulation of synaptic transmission	18/926	145/17913	0.000498998	0.003984425	0.002940608	107/348/8938/6347/729230/2048/2903/8564/10288/27065/55607/5649/22941/6507/6622/6812/91683/7305	18
BP	GO:0043029	T cell homeostasis	8/926	37/17913	0.000500305	0.003984425	0.002940608	11151/84636/3559/3718/3071/5027/124976/1831	8
BP	GO:0045124	regulation of bone resorption	8/926	37/17913	0.000500305	0.003984425	0.002940608	952/1436/53832/3569/5027/7128/4982/8600	8
BP	GO:0050848	regulation of calcium-mediated signaling	14/926	98/17913	0.000500706	0.003984425	0.002940608	100/6348/6351/933/916/2633/120892/3084/5031/5341/1827/50852/54209/7535	14
BP	GO:2000117	negative regulation of cysteine-type endopeptidase activity	13/926	87/17913	0.000506795	0.0040202037	0.002967137	939/960/1800/3428/2537/27074/4057/4318/4804/5329/5272/6622/7422	13
B	GO:00	actin	20/92	170/1791	0.	0.	0.002967137	119/199/274/146206/6366/1236/23242/11151/342184/3055/	2

© 2020 Yu Y et al. *JAMA Network Open.*

P	08154	polymerization or depolymerization	6	3	0005068 09	0040 2037		3059/3383/10788/3071/4868/5063/5341/55607/7454/7456	0
BP	GO:0050803	regulation of synapse structure or activity	24/926	222/17913	0.00051972 6	0.0041 0997	0.003033264	575/348/1006/2048/2213/2904/57549/3587/3556/10288/4038/120892/266727/5818/4897/9423/5063/55607/5800/5649/84189/6622/80725/7097	24
BP	GO:0070302	regulation of stress-activated protein kinase signaling cascade	24/926	222/17913	0.00051972 6	0.0041 0997	0.003033264	6363/6366/1236/939/1843/1906/10595/355/2213/3553/11184/653361/64127/5187/26191/10125/10371/140885/6504/10333/8600/23043/7422/9839	24
BP	GO:1903409	reactive oxygen species biosynthetic process	16/926	122/17913	0.00054694 5	0.0043 1841 4	0.003187101	54/575/199/384/948/1536/1906/3383/3553/3689/4129/9722/5806/140885/6622/10333	16
BP	GO:0048545	response to steroid hormone	35/926	375/17913	0.00054778 9	0.0043 1841 4	0.003187101	21/23205/84830/199/249/301/374/655/952/1536/1601/1829/1843/1906/2099/2322/23426/3383/3569/4129/4306/3164/4929/8013/11240/5069/5187/10891/6696/7035/7054/7068/7097/8626/7538	35
BP	GO:0072538	T-helper 17 type immune response	7/926	29/17913	0.00055278 9	0.0043 5107	0.003211201	3594/3569/3662/4063/114548/5588/114836	7
BP	GO:1902106	negative regulation of	14/926	99/17913	0.00	0.00	0.003224794	301/714/6348/972/1021/1493/2213/3718/3902/10859/11006/4057/55365/28959	14

© 2020 Yu Y et al. *JAMA Network Open.*

		leukocyte differentiation			0555991	4369487			
BP	GO:0045637	regulation of myeloid cell differentiation	26/926	250/17913	0.000563651	0.00044228 3	0.003264162	714/6348/972/1021/1441/2124/2811/29909/3059/8356/8361/3218/3304/3725/10859/11006/4057/3071/8013/5579/5588/6688/8600/54209/7305/7538	26
BP	GO:0045862	positive regulation of proteolysis	32/926	333/17913	0.000566646	0.00044394 6	0.003276436	150/9447/348/718/147372/1285/1378/26999/1601/112399/2152/355/2729/2903/2904/3428/3553/90865/3932/120892/4210/4804/22861/114548/26471/26577/64065/149628/6091/6279/6622/54209	32
BP	GO:0043122	regulation of I-kappaB kinase/NF-kappaB signaling	24/926	224/17913	0.000591556	0.000462746 5	0.003415188	330/6363/6366/1236/948/972/2099/51704/3162/3553/4057/197358/64127/5187/11040/5579/6398/140885/7052/10333/7128/8600/10346/10537	24
BP	GO:0001501	skeletal system development	42/926	479/17913	0.000593565	0.000463602 7	0.003421507	54/249/655/960/1116/50515/1310/55790/56603/1749/1906/2201/2261/342184/79977/8091/3209/3213/3215/3216/3218/3481/3696/10859/4057/4094/4318/4781/5027/5079/5251/5788/860/6662/124976/6899/7020/4982/8600/8626/7227/7305	42
BP	GO:0032418	lysosome localization	11/926	67/17913	0.000597474	0.000465937 6	0.00343874	30817/695/8832/2207/3162/8013/5293/5294/5880/10125/6812	11
BP	GO:0071496	cellular response to external	31/926	320/17913	0.0005	0.0046	0.003443879	199/27063/467/1536/1824/355/2350/2729/3162/3383/3428/3553/3676/3725/57535/4023/120892/4316/4929/5027/5029/5251/5788/9750/58528/143686/6513/6662/51284/51311/7412	31

© 2020 Yu Y et al. *JAMA Network Open.*

		stimulus			99287	6634			
BP	GO:0050856	regulation of T cell receptor signaling pathway	8/926	38/17913	0.000605355	0.004706359	0.003473414	100/1236/2633/3932/11006/26191/9840/50852	8
BP	GO:1903428	positive regulation of reactive oxygen species biosynthetic process	10/926	57/17913	0.000611861	0.004749656	0.003505368	575/199/948/1906/3383/3553/3689/9722/5806/10333	10
BP	GO:0051402	neuron apoptotic process	25/926	238/17913	0.000616331	0.004773845	0.00352322	728/6347/6348/10752/11151/112399/2729/3162/3684/3725/4804/22861/4929/8013/26471/5063/10891/54463/6622/6648/6812/7020/7068/8626/7305	25
BP	GO:0035821	modification of morphology or physiology of other organism	19/926	160/17913	0.000616861	0.004773845	0.00352322	348/6357/6348/6351/6352/30835/10332/2208/10578/8091/3429/3725/4057/4069/219972/5027/5551/5806/5272	19
BP	GO:1903305	regulation of regulated secretory pathway	17/926	136/17913	0.000652623	0.005042904	0.003721793	107/30817/150/321/8938/729230/8832/2207/2213/3134/3162/3684/3689/120892/5579/5880/6812	17
BP	GO:0008360	regulation of cell shape	16/926	124/17913	0.00	0.00	0.0037338	301/55843/6357/6347/6348/11151/1436/23136/752/3055/3383/3689/4478/57619/375611/7422	16

© 2020 Yu Y et al. *JAMA Network Open.*

					06 55 72 6	50 59 17 3			
BP	GO:0010950	positive regulation of endopeptidase activity	19/926	161/17913	0.00066336	0.00513321 9	0.003788448	9447/1285/1378/26999/112399/2152/355/2903/2904/3428/3932/4210/4804/22861/114548/64065/6091/6279/6622	19
BP	GO:0099177	regulation of trans-synaptic signaling	36/926	394/17913	0.00068040 5	0.00523179 6	0.0038612	107/575/321/348/8938/6347/729230/952/999/1630/1906/1959/2048/2171/2903/2904/3274/3553/8564/10288/120892/79772/27065/23228/55607/5577/5579/5649/22941/6507/6622/9627/6812/91683/347733/7305	36
BP	GO:1903426	regulation of reactive oxygen species biosynthetic process	14/926	101/17913	0.00068222 9	0.00523179 6	0.0038612	54/575/199/384/948/1906/3383/3553/3689/9722/5806/140885/6622/10333	14
BP	GO:2000379	positive regulation of reactive oxygen species metabolic process	14/926	101/17913	0.00068222 9	0.00523179 6	0.0038612	575/199/948/1906/2358/3383/3553/3684/3689/9722/5806/6622/10333/7305	14
BP	GO:0032656	regulation of interleukin-13 production	6/926	22/17913	0.00068568 6	0.00523455 2	0.003863235	384/3556/90865/3662/353514/114548	6
BP	GO:0045624	positive regulation of	6/926	22/17913	0.00	0.00	0.003863235	301/6363/941/942/3594/114548	6

© 2020 Yu Y et al. *JAMA Network Open.*

		T-helper cell differentiation			06856866	52345552			
BP	GO:1903306	negative regulation of regulated secretory pathway	6/926	22/17913	0.0006856866	0.0052345552	0.003863235	150/729230/8832/2213/3134/3162	6
BP	GO:0032689	negative regulation of interferon-gamma production	7/926	30/17913	0.000689195	0.0052356188	0.003864021	29126/10225/84868/9173/90865/10859/80380	7
BP	GO:0046596	regulation of viral entry into host cell	7/926	30/17913	0.000689195	0.0052356188	0.003864021	972/2219/8519/10581/10410/5806/11074	7
BP	GO:0007254	JNK cascade	22/926	200/17913	0.000690798	0.0052356188	0.003864021	6363/6366/1236/939/1906/10595/2213/3553/11184/653361/64127/4868/5187/26191/10125/10371/140885/6504/10333/8600/23043/9839	22
BP	GO:0009612	response to mechanical stimulus	22/926	200/17913	0.000690798	0.0052356188	0.003864021	84059/27063/1116/3627/7852/1906/355/2729/3553/3725/4316/5027/9750/6507/375611/6513/84189/6662/64220/51284/51311/7273	22
BP	GO:0017157	regulation of exocytosis	21/926	187/17913	0.0006	0.0052	0.003864021	107/30817/150/301/321/8938/729230/8832/2207/2213/3134/3162/3684/3689/120892/5579/5874/5880/6622/6812/7447	21

© 2020 Yu Y et al. *JAMA Network Open.*

					9099	35618			
BP	GO:0050818	regulation of coagulation	12/926	79/17913	0.000708107	0.005307322	0.00391694	84830/348/948/1906/2152/2811/5329/5341/5588/6403/710/7035	12
BP	GO:0046717	acid secretion	15/926	113/17913	0.000708747	0.005307322	0.00391694	5244/366/1906/3274/3553/8564/5027/26279/8497/6446/6507/6622/6812/8600/9256	15
BP	GO:0002467	germinal center formation	5/926	15/17913	0.000709877	0.005307322	0.00391694	100/10563/89857/7128/10673	5
BP	GO:0002716	negative regulation of natural killer cell mediated immunity	5/926	15/17913	0.000709877	0.005307322	0.00391694	10225/84868/3134/10859/5272	5
BP	GO:0010819	regulation of T cell chemotaxis	5/926	15/17913	0.000709877	0.005307322	0.00391694	6366/6352/729230/3627/10563	5
BP	GO:0034134	toll-like receptor 2 signaling pathway	5/926	15/17913	0.000709	0.005307	0.00391694	118788/7096/7097/10333/7128	5

© 2020 Yu Y et al. *JAMA Network Open.*

					87<br>7	32<br>2			
BP	GO:0044406	adhesion of symbiont to host	5/926	15/17913	0.000709877	0.005307322	0.00391694	30835/10332/3383/4057/5818	5
BP	GO:0090197	positive regulation of chemokine secretion	5/926	15/17913	0.000709877	0.005307322	0.00391694	199/1436/9173/90865/4023	5
BP	GO:2000484	positive regulation of interleukin-8 secretion	5/926	15/17913	0.000709877	0.005307322	0.00391694	929/914/2219/7096/7097	5
BP	GO:0030890	positive regulation of B cell proliferation	8/926	39/17913	0.000727514	0.005431175	0.004008347	100/952/972/115352/1880/3071/5788/10673	8
BP	GO:0007160	cell-matrix adhesion	23/926	214/17913	0.000729922	0.005441136	0.004015699	56999/4059/6366/1236/948/916/960/10225/1021/55612/342184/3675/3676/3683/3689/3691/3897/4321/64098/6281/6614/7412/7422	23
BP	GO:0014065	phosphatidylinositol 3-kinase signaling	17/926	138/17913	0.00077170	0.00574418	0.004239359	6352/940/1906/2322/3059/653361/197358/118788/5293/5294/23533/8395/26051/10125/5649/6403/6662	17

					9	9			
BP	GO:0006958	complement activation, classical pathway	13/926	91/17913	0.000785257	0.005836466	0.004307462	712/713/714/717/718/720/721/730/3426/1378/1380/710/55061	13
BP	GO:0031529	ruffle organization	9/926	49/17913	0.000804966	0.005974191	0.004409107	199/146206/6366/1236/23242/1436/3383/5341/26228	9
BP	GO:0048568	embryonic organ development	37/926	413/17913	0.000831014	0.006158493	0.004545127	100/655/50515/23242/3576/1749/79733/1906/8320/2048/2201/2709/79977/3215/3216/3218/3481/3856/26018/4647/8013/9423/5079/113026/639/9750/860/6535/84189/9021/6662/64220/6899/8463/7020/7054/7422	37
BP	GO:0007605	sensory perception of sound	17/926	139/17913	0.000837907	0.006200509	0.004576136	84059/57214/53405/1285/3383/26018/4647/89797/9750/6507/375611/84189/6899/7020/7054/7068/7399	17
BP	GO:0002724	regulation of T cell cytokine production	7/926	31/17913	0.000850939	0.006287772	0.004640537	729230/3134/3553/3569/114548/91543/54440	7
BP	GO:0010975	regulation of neuron projection development	40/926	458/17913	0.000855885	0.006315111	0.004660715	389658/27063/348/655/952/23242/7852/1630/2048/23105/23426/3675/3792/5653/3897/23566/4038/120892/4139/4804/4897/9423/10439/5063/5365/55607/5800/5649/5979/56963/5997/6091/10371/6696/80725/51760/23043/347733/7422/9839	40

© 2020 Yu Y et al. *JAMA Network Open.*

BP	GO:0002369	T cell cytokine production	8/926	40/17913	0.000868748	0.006391414	0.004717028	729230/3134/3553/3569/114548/91543/54440/6556	8
BP	GO:1902893	regulation of pri-miRNA transcription by RNA polymerase II	8/926	40/17913	0.000868748	0.006391414	0.004717028	8862/3725/688/11006/4781/4804/6662/6688	8
BP	GO:0034767	positive regulation of ion transmembrane transport	18/926	152/17913	0.000876445	0.006429375	0.004745045	5243/6347/729230/930/57214/3627/6373/4283/2048/3359/9722/5027/5031/5649/6622/6769/342667/54209	18
BP	GO:1903707	negative regulation of hemopoiesis	18/926	152/17913	0.000876445	0.006429375	0.004745045	301/714/6348/972/1021/1493/2213/29909/8361/3218/3718/3902/10859/11006/4057/55365/28959/7538	18
BP	GO:0060143	positive regulation of syncytium formation by plasma membrane fusion	6/926	23/17913	0.000887478	0.006491526	0.004790913	575/963/4283/9750/54209/7305	6
BP	GO:0071677	positive regulation of mononuclear cell migration	6/926	23/17913	0.000887478	0.006491526	0.004790913	199/6352/729230/3627/2358/7941	6

© 2020 Yu Y et al. *JAMA Network Open.*

BP	GO:0014068	positive regulation of phosphatidylinositol 3-kinase signaling	12/926	81/17913	0.000889813	0.006499224	0.004796595	6352/940/2322/3059/653361/118788/5293/5294/23533/5649/6403/6662	12
BP	GO:0060538	skeletal muscle organ development	19/926	165/17913	0.000899962	0.006563895	0.004844324	467/50937/1134/1310/56603/1958/1959/8320/2817/9734/59269/3792/688/4868/26471/5079/9750/64220/6899	19
BP	GO:1905517	macrophage migration	9/926	50/17913	0.000937349	0.006826756	0.005038322	719/728/6347/6348/6352/729230/56833/26228/54209	9
BP	GO:0044070	regulation of anion transport	13/926	93/17913	0.000966839	0.007021849	0.005182306	5243/5244/348/10396/1906/3553/8564/5027/5997/6098/6622/6812/8600	13
BP	GO:0031345	negative regulation of cell projection organization	19/926	166/17913	0.000968292	0.007021849	0.005182306	348/6366/952/1630/2048/23105/2904/3675/4038/120892/4117/4804/9423/5365/5800/56963/10371/6696/26228	19
BP	GO:0051262	protein tetramerization	19/926	166/17913	0.000968292	0.007021849	0.005182306	6352/919/972/973/978/115362/2904/8356/8361/4038/931/6281/375611/6622/6648/6999/8626/80036/140803	19
B	GO:00	neutrophil	5/926	16/17913	0.	0.	0.005254816	301/729230/3569/5142/5293	5

© 2020 Yu Y et al. *JAMA Network Open.*

P	01780	homeostasis			0.0009888 64	0.0071200 99			
BP	GO:0002295	T-helper cell lineage commitment	5/926	16/17913	0.0009888 64	0.0071200 99	0.005254816	3594/3569/3662/4063/114836	5
BP	GO:0006957	complement activation, alternative pathway	5/926	16/17913	0.0009888 64	0.0071200 99	0.005254816	718/730/629/1378/11326	5
BP	GO:0010934	macrophage cytokine production	5/926	16/17913	0.0009888 64	0.0071200 99	0.005254816	948/972/51704/11213/10859	5
BP	GO:0034116	positive regulation of heterotypic cell-cell adhesion	5/926	16/17913	0.0009888 64	0.0071200 99	0.005254816	655/960/2651/3553/3932	5
BP	GO:0030833	regulation of actin filament polymerization	16/926	129/17913	0.00101139	0.0072719 69	0.005366901	119/274/146206/6366/1236/11151/342184/3055/3059/3383/10788/3071/4868/5063/55607/7454	16
BP	GO:0014002	astrocyte developmen	8/926	41/17913	0.00	0.00	0.005463994	712/728/1021/2358/3553/3569/6279/54209	8

		t			10<br>31<br>14<br>8	74<br>03<br>52<br>8			
B<br>P	GO:00<br>01975	response to amphetamin<br>e	7/926	32/17913	0.<br>00<br>10<br>41<br>19<br>5	0.<br>00<br>74<br>65<br>08<br>9	0.005509428	2903/9734/3383/4929/26575/5997/7054	7
B<br>P	GO:00<br>10952	positive regulation of peptidase activity	20/92<br>6	180/1791<br>3	0.<br>00<br>10<br>46<br>62<br>6	0.<br>00<br>74<br>93<br>43	0.005530344	9447/1285/1378/26999/112399/2152/355/2903/2904/3428/3<br>932/4210/4804/22861/114548/26577/64065/6091/6279/6622	2<br>0
B<br>P	GO:00<br>45778	positive regulation of ossification	13/92<br>6	94/17913	0.<br>00<br>10<br>70<br>04<br>7	0.<br>00<br>76<br>46<br>34<br>4	0.005643199	84059/655/2201/54857/3400/8519/3569/4057/4745/5027/86<br>0/7020/8626	1<br>3
B<br>P	GO:00<br>32147	activation of protein kinase activity	29/92<br>6	303/1791<br>3	0.<br>00<br>10<br>71<br>00<br>1	0.<br>00<br>76<br>46<br>34<br>4	0.005643199	107/113/150/728/6363/6352/972/1116/7852/10595/2357/517<br>04/3553/23566/120892/11184/3071/64127/3084/5027/5063/<br>5577/5788/5979/6556/10333/8600/23043/7422	2<br>9
B<br>P	GO:00<br>01954	positive regulation of cell-matrix adhesion	9/926	51/17913	0.<br>00<br>10<br>86<br>85<br>7	0.<br>00<br>77<br>16<br>07<br>9	0.005694665	6366/1236/948/916/1021/55612/342184/6281/7422	9
B<br>P	GO:00<br>06584	catecholami<br>ne<br>metabolic	9/926	51/17913	0.<br>00<br>10	0.<br>00<br>77	0.005694665	2903/3684/3689/4129/4929/6622/9627/6799/7054	9

© 2020 Yu Y et al. *JAMA Network Open.*

		process			86857	16079			
BP	GO:0009712	catechol-containing compound metabolic process	9/926	51/17913	0.001086857	0.007716079	0.005694665	2903/3684/3689/4129/4929/6622/9627/6799/7054	9
BP	GO:0021545	cranial nerve development	9/926	51/17913	0.001086857	0.007716079	0.005694665	1959/2048/3213/89797/10371/6507/84189/6899/7020	9
BP	GO:2000377	regulation of reactive oxygen species metabolic process	21/926	194/17913	0.00111525	0.00788017	0.005815769	54/575/199/384/655/948/1906/2358/3099/3383/3553/3684/3689/9722/5806/5880/140885/6622/7020/10333/7305	21
BP	GO:0002861	regulation of inflammatory response to antigenic stimulus	6/926	24/17913	0.001132064	0.00798113	0.00589028	695/718/1236/940/2207/2213	6
BP	GO:0034162	toll-like receptor 9 signaling pathway	6/926	24/17913	0.001132064	0.00798113	0.00589028	115352/84868/118788/91543/51284/51311	6
BP	GO:0045954	positive regulation of natural killer cell	6/926	24/17913	0.001132	0.007981	0.00589028	3134/3902/10125/4068/114836/7409	6

		mediated cytotoxicity			06 4	13			
BP	GO:0048143	astrocyte activation	6/926	24/17913	0.0011320 64	0.0079811 3	0.00589028	712/728/2358/3553/3569/54209	6
BP	GO:0002664	regulation of T cell tolerance induction	4/926	10/17913	0.0011593 6	0.0080821 92	0.005964866	916/3620/3559/10288	4
BP	GO:0002887	negative regulation of myeloid leukocyte mediated immunity	4/926	10/17913	0.0011593 6	0.0080821 92	0.005964866	729230/8832/2213/3162	4
BP	GO:0019062	virion attachment to host cell	4/926	10/17913	0.0011593 6	0.0080821 92	0.005964866	30835/10332/3383/5818	4
BP	GO:0032308	positive regulation of prostaglandin secretion	4/926	10/17913	0.0011593 6	0.0080821 92	0.005964866	1906/3553/5027/8600	4
BP	GO:0032621	interleukin-18 production	4/926	10/17913	0.0011593 6	0.0080821 9	0.005964866	8832/115362/114548/7097	4

© 2020 Yu Y et al. *JAMA Network Open.*

						2			
BP	GO:0045625	regulation of T-helper 1 cell differentiation	4/926	10/17913	0.00115936	0.008082192	0.005964866	301/6363/941/3718	4
BP	GO:0070391	response to lipoteichoic acid	4/926	10/17913	0.00115936	0.008082192	0.005964866	929/948/7097/54209	4
BP	GO:0071223	cellular response to lipoteichoic acid	4/926	10/17913	0.00115936	0.008082192	0.005964866	929/948/7097/54209	4
BP	GO:0031346	positive regulation of cell projection organization	33/926	362/17913	0.001160749	0.008082192	0.005964866	389658/27063/348/655/146206/6363/6366/1236/23242/7852/139818/5168/23426/3675/3897/23566/4804/9423/5027/5063/26499/5365/55607/5880/5649/5979/5997/9750/6091/51760/347733/7422/9839	33
BP	GO:0007519	skeletal muscle tissue development	18/926	156/17913	0.001186071	0.008247178	0.00608663	467/50937/1134/1310/56603/1958/1959/8320/2817/9734/59269/3792/688/4868/26471/5079/9750/6899	18
BP	GO:0048167	regulation of synaptic plasticity	19/926	169/17913	0.001200556	0.008336461	0.006152523	107/575/348/952/1959/2048/2903/2904/3274/10288/79772/27065/55607/5649/22941/6622/6812/91683/7305	19

© 2020 Yu Y et al. *JAMA Network Open.*

BP	GO:0048732	gland development	37/926	422/17913	0.0012271366	0.0085093755	0.006280138	100/8862/374/9914/655/999/23242/1396/1436/79733/2099/3099/3162/3213/3274/3400/3481/3664/3725/284217/4478/4781/3084/9423/4939/6091/10371/5268/6662/64220/6899/29842/7052/7128/8600/8626/7422	37
BP	GO:0030838	positive regulation of actin filament polymerization	12/926	84/17913	0.0012340177	0.008545454	0.006306726	274/146206/6366/1236/11151/342184/3055/3383/10788/3071/4868/7454	12
BP	GO:0050804	modulation of chemical synaptic transmission	35/926	393/17913	0.001256566	0.0086899909	0.006413377	107/575/321/348/8938/6347/729230/952/999/1630/1906/1959/2048/2903/2904/3274/3553/8564/10288/120892/79772/27065/23228/55607/5577/5579/5649/22941/6507/6622/9627/6812/91683/347733/7305	35
BP	GO:0045589	regulation of regulatory T cell differentiation	7/926	33/17913	0.0012633321	0.0087121262	0.006430138	940/1378/1493/3559/3902/10288/11006	7
BP	GO:0055094	response to lipoprotein particle	7/926	33/17913	0.0012633321	0.0087121262	0.006430138	84830/348/948/2207/3689/4023/10333	7
BP	GO:0043281	regulation of cysteine-type endopeptidase activity involved in apoptotic	21/926	196/17913	0.0012660085	0.0087198817	0.00643545	939/960/1285/1800/112399/2152/355/2537/27074/3932/4318/4804/22861/114548/5329/6091/6279/5272/6622/8626/7422	21

		process							
BP	GO:0009991	response to extracellular stimulus	42/926	498/17913	0.001276757	0.008781385	0.006480889	100/122622/199/249/348/467/655/717/916/3627/1536/56603/1824/355/2350/2729/3162/3383/3428/3553/3676/3725/57535/4023/120892/4316/64127/4929/5027/5029/5251/10891/5788/58528/143686/6581/6513/6696/7054/4982/7412/7538	42
BP	GO:0060759	regulation of response to cytokine stimulus	20/926	183/17913	0.001283348	0.008814756	0.006505517	330/6352/972/1906/3304/7850/3569/11213/3977/4321/84166/11240/5788/6091/9021/26228/7097/7128/54209/7294	20
BP	GO:0001959	regulation of cytokine-mediated signaling pathway	19/926	170/17913	0.001287848	0.008833697	0.006519496	330/6352/972/1906/3304/7850/3569/11213/3977/4321/84166/11240/5788/6091/9021/26228/7128/54209/7294	19
BP	GO:0030282	bone mineralization	14/926	108/17913	0.00133145	0.009120436	0.006731117	84059/655/6348/2201/2261/10457/4057/4745/5027/5251/6662/5552/7020/7286	14
BP	GO:0021783	preganglionic parasympathetic fiber development	5/926	17/17913	0.001341704	0.009141258	0.006746484	1959/89797/10371/6899/7020	5
BP	GO:0032740	positive regulation of interleukin-17 production	5/926	17/17913	0.001341704	0.009141258	0.006746484	3569/4063/64127/5588/114836	5

BP	GO:0043373	CD4-positive, alpha-beta T cell lineage commitment	5/926	17/17913	0.001341704	0.009141258	0.006746484	3594/3569/3662/4063/114836	5
BP	GO:1903975	regulation of glial cell migration	5/926	17/17913	0.001341704	0.009141258	0.006746484	6348/729230/1880/26228/54209	5
BP	GO:0002719	negative regulation of cytokine production involved in immune response	6/926	25/17913	0.001425126	0.009670631	0.007137175	10225/3134/3162/11213/3718/10859	6
BP	GO:0032616	interleukin-13 production	6/926	25/17913	0.001425126	0.009670631	0.007137175	384/3556/90865/3662/353514/114548	6
BP	GO:0060384	innervation	6/926	25/17913	0.001425126	0.009670631	0.007137175	3676/26018/5979/10371/84189/7412	6
BP	GO:0043616	keratinocyte proliferation	8/926	43/17913	0.001428405	0.009679924	0.007144033	374/135228/83481/2069/55612/3664/8626/7538	8

© 2020 Yu Y et al. *JAMA Network Open.*

BP	GO:0050954	sensory perception of mechanical stimulus	18/926	159/17913	0.001475363	0.009984803	0.007369042	84059/57214/53405/1285/7852/3383/26018/4647/89797/9750/6507/375611/84189/6899/7020/7054/7068/7399	18
BP	GO:0010574	regulation of vascular endothelial growth factor production	7/926	34/17913	0.001520855	0.010251614	0.007565955	718/719/728/147372/729230/3553/3569	7
BP	GO:0032660	regulation of interleukin-17 production	7/926	34/17913	0.001520855	0.010251614	0.007565955	384/3569/4063/3071/64127/5588/114836	7
BP	GO:0032735	positive regulation of interleukin-12 production	7/926	34/17913	0.001520855	0.010251614	0.007565955	6363/1236/948/3620/3394/4050/7097	7
BP	GO:0002064	epithelial cell development	21/926	199/17913	0.001532262	0.010314787	0.007612579	54507/1021/7852/2099/79977/3274/3383/3553/688/4478/4647/4868/26051/639/9750/6098/57619/84189/6662/29842/8626	21
BP	GO:0000187	activation of MAPK activity	17/926	147/17913	0.001564261	0.010492514	0.007743746	728/6363/972/7852/10595/2357/3553/23566/120892/11184/64127/3084/5027/5063/5788/5979/8600	17
B	GO:00	regulation of	17/92	147/1791	0.	0.	0.007743746	119/274/146206/6366/1236/11151/342184/3055/3059/3383/	1

P	08064	actin polymerization or depolymerization	6	3	001564261	010492514		10788/3071/4868/5063/5341/55607/7454	7
BP	GO:0006919	activation of cysteine-type endopeptidase activity involved in apoptotic process	11/926	75/17913	0.001564873	0.010492514	0.007743746	1285/112399/2152/355/3932/4804/22861/114548/6091/6279/6622	11
BP	GO:0033559	unsaturated fatty acid metabolic process	13/926	98/17913	0.001579464	0.010576356	0.007805623	241/972/1557/1573/8560/1906/6785/2171/3553/5730/5742/79966/6916	13
BP	GO:0048015	phosphatidylinositol-mediated signaling	18/926	160/17913	0.001584188	0.010593994	0.007818641	6352/940/1436/1906/2322/3059/653361/197358/118788/5293/5294/23533/8395/26051/10125/5649/6403/6662	18
BP	GO:0038093	Fc receptor signaling pathway	24/926	241/17913	0.001639338	0.010948362	0.008080173	695/919/917/26999/9844/2207/2209/2212/2213/2214/3055/3702/3725/3937/64005/3071/8013/5588/5696/5698/5788/7409/7454/7456	24
BP	GO:0032715	negative regulation of interleukin-6 production	9/926	54/17913	0.001654 4	0.011034415	0.008143682	55024/84868/11213/3071/197358/26191/140885/6504/7128	9

© 2020 Yu Y et al. *JAMA Network Open.*

BP	GO:0030225	macrophage differentiation	8/926	44/17913	0.001668029	0.011096117	0.00818922	714/1436/3059/133396/4318/6688/7097/7422	8
BP	GO:0048483	autonomic nervous system development	8/926	44/17913	0.001668029	0.011096117	0.00818922	1959/89797/5979/10371/6899/7020/8626/7412	8
BP	GO:0060560	developmental growth involved in morphogenesis	22/926	214/17913	0.001680205	0.011158136	0.008234992	348/374/23242/7852/26999/1630/9201/2099/342184/23105/3676/3897/23566/4897/9423/10439/10371/6662/6696/51760/7422/9839	22
BP	GO:1903035	negative regulation of response to wounding	12/926	87/17913	0.001681755	0.011158136	0.008234992	84830/348/135228/1906/83481/2811/5329/56963/710/89790/6696/7035	12
BP	GO:0030832	regulation of actin filament length	17/926	148/17913	0.001684368	0.011160866	0.008237007	119/274/146206/6366/1236/11151/342184/3055/3059/3383/10788/3071/4868/5063/5341/55607/7454	17
BP	GO:0002483	antigen processing and presentation of endogenous peptide	4/926	11/17913	0.00174762	0.011430563	0.00843605	64167/3134/6890/6891	4

© 2020 Yu Y et al. *JAMA Network Open.*

		antigen							
BP	GO:0002638	negative regulation of immunoglobulin production	4/926	11/17913	0.0017476 2	0.0114305 63	0.00843605	933/2213/115352/90865	4
BP	GO:0002923	regulation of humoral immune response mediated by circulating immunoglobulin	4/926	11/17913	0.0017476 2	0.0114305 63	0.00843605	1378/2208/2213/5788	4
BP	GO:0032306	regulation of prostaglandin secretion	4/926	11/17913	0.0017476 2	0.0114305 63	0.00843605	1906/3553/5027/8600	4
BP	GO:0045793	positive regulation of cell size	4/926	11/17913	0.0017476 2	0.0114305 63	0.00843605	10000/1906/5979/375611	4
BP	GO:0072672	neutrophil extravasation	4/926	11/17913	0.0017476 2	0.0114305 63	0.00843605	120425/5293/5294/9750	4
BP	GO:0097048	dendritic cell apoptotic process	4/926	11/17913	0.001747	0.011430	0.00843605	6363/6366/1236/10859	4

© 2020 Yu Y et al. *JAMA Network Open.*

					62	563			
BP	GO:0099188	postsynaptic cytoskeleton organization	4/926	11/17913	0.00174762	0.0114305563	0.00843605	9118/4744/9722/55607	4
BP	GO:2000668	regulation of dendritic cell apoptotic process	4/926	11/17913	0.00174762	0.0114305563	0.00843605	6363/6366/1236/10859	4
BP	GO:2001204	regulation of osteoclast development	4/926	11/17913	0.00174762	0.0114305563	0.00843605	10859/4057/8600/7305	4
BP	GO:0048668	collateral sprouting	6/926	26/17913	0.00177261 2	0.0115487 8	0.008523297	23242/1630/23105/23566/6696/9839	6
BP	GO:0072539	T-helper 17 cell differentiation	6/926	26/17913	0.00177261 2	0.0115487 8	0.008523297	3594/3569/3662/4063/114548/114836	6
BP	GO:0001779	natural killer cell differentiation	5/926	18/17913	0.00177936	0.0115487 8	0.008523297	5293/639/5788/10125/9760	5

© 2020 Yu Y et al. *JAMA Network Open.*

					4				
BP	GO:0046641	positive regulation of alpha-beta T cell proliferation	5/926	18/17913	0.0017793634	0.01154878	0.008523297	729230/940/916/5788/7535	5
BP	GO:0090196	regulation of chemokine secretion	5/926	18/17913	0.0017793634	0.01154878	0.008523297	199/1436/9173/90865/4023	5
BP	GO:1901739	regulation of myoblast fusion	5/926	18/17913	0.0017793634	0.01154878	0.008523297	575/963/3627/4283/9750	5
BP	GO:0042063	gliogenesis	27/926	285/17913	0.0018141412	0.011721258	0.00865059	374/274/712/728/6347/6348/729230/1021/1436/7852/1959/2358/2817/1880/3400/3553/90865/3569/10397/4781/3084/5649/6279/6662/26228/7097/54209	27
BP	GO:0048246	macrophage chemotaxis	7/926	35/17913	0.00181175	0.011721258	0.00865059	719/728/6347/6348/6352/56833/26228	7
BP	GO:0071402	cellular response to lipoprotein particle stimulus	7/926	35/17913	0.00181175	0.011721258	0.00865059	84830/348/948/2207/3689/4023/10333	7

© 2020 Yu Y et al. *JAMA Network Open.*

BP	GO:0090322	regulation of superoxide metabolic process	7/926	35/17913	0.001817 5	0.011721258	0.00865059	54/655/948/2358/3684/3689/7305	7
BP	GO:2000310	regulation of NMDA receptor activity	7/926	35/17913	0.0018175	0.011721258	0.00865059	6347/729230/2048/2903/2904/10891/5649	7
BP	GO:0051403	stress-activated MAPK cascade	26/926	271/17913	0.001829878	0.01178609	0.008698438	6363/6366/1236/939/1843/1906/10595/355/2213/3553/11184/653361/64127/4868/5187/26191/10125/10371/140885/6504/10333/8600/23043/7422/9839/7538	26
BP	GO:0000302	response to reactive oxygen species	23/926	230/17913	0.001920499	0.012354076	0.009117626	100/348/374/655/695/6363/1236/948/1800/1843/1906/3162/3569/3725/120892/4318/653361/8013/10891/143686/140885/6648/7128	23
BP	GO:0046850	regulation of bone remodeling	8/926	45/17913	0.001938349	0.012437331	0.009179071	952/1436/53832/3569/5027/7128/4982/8600	8
BP	GO:0072604	interleukin-6 secretion	8/926	45/17913	0.001938349	0.012437331	0.009179071	199/55024/3553/3556/353514/4023/26191/51311	8
B	GO:00	astrocyte	11/92	77/17913	0.	0.	0.009182404	274/712/728/1021/2358/3400/3553/3569/6279/6662/54209	1

© 2020 Yu Y et al. *JAMA Network Open.*

P	48708	differentiation	6		001943962	012441848			1
BP	GO:0097061	dendritic spine organization	11/926	77/17913	0.001943962	0.012441848	0.009182404	348/55619/2048/2213/2904/3675/120892/5063/55607/5649/80725	11
BP	GO:0097696	STAT cascade	17/926	150/17913	0.001947964	0.012451741	0.009189706	6347/6352/729230/1436/2261/2322/3059/3587/11009/133396/3569/3575/3718/5788/5979/9021/6775	17
BP	GO:0048017	inositol lipid-mediated signaling	18/926	163/17913	0.001952219	0.012463221	0.009198178	6352/940/1436/1906/2322/3059/653361/197358/118788/5293/5294/23533/8395/26051/10125/5649/6403/6662	18
BP	GO:0044344	cellular response to fibroblast growth factor stimulus	16/926	138/17913	0.002064748	0.013165038	0.009716137	8862/6347/6352/960/10563/3576/1960/54845/2261/2729/2817/3164/860/6622/6899/7538	16
BP	GO:0048661	positive regulation of smooth muscle cell proliferation	13/926	101/17913	0.002081278	0.013240727	0.009771998	199/6352/1906/2069/1839/3162/3569/3725/4318/8013/5031/10891/7052	13
BP	GO:0048705	skeletal system	21/926	204/17913	0.00	0.01	0.009771998	54/249/655/50515/55790/56603/1749/2201/2261/342184/79977/3215/3216/3218/4057/5027/5079/860/6662/6899/7020	21

© 2020 Yu Y et al. *JAMA Network Open.*

		morphogenesis			0.002081843	0.01324072 7			
BP	GO:0010573	vascular endothelial growth factor production	7/926	36/17913	0.002157117	0.013685137	0.010099984	718/719/728/147372/729230/3553/3569	7
BP	GO:1905521	regulation of macrophage migration	7/926	36/17913	0.002157117	0.013685137	0.010099984	719/728/6348/6352/56833/26228/54209	7
BP	GO:0043304	regulation of mast cell degranulation	6/926	27/17913	0.002180705	0.013817494	0.010197667	30817/8832/2207/3162/5880/6812	6
BP	GO:0007187	G protein-coupled receptor signaling pathway, coupled to cyclic nucleotide second messenger	19/926	178/17913	0.002201518	0.013931951	0.010282139	107/113/150/301/6347/3627/6373/4283/1906/2357/2358/8477/27201/23566/8685/4886/5996/5997/7253	19
BP	GO:0045669	positive regulation of osteoblast differentiation	10/926	67/17913	0.00220652	0.01394623	0.010292683	655/2201/54857/3400/8519/3569/4057/4745/860/8626	10

© 2020 Yu Y et al. *JAMA Network Open.*

					6	7			
BP	GO:0061614	pri-miRNA transcription by RNA polymerase II	8/926	46/17913	0.002242018	0.014121004	0.010421665	8862/3725/688/11006/4781/4804/6662/6688	8
BP	GO:0070169	positive regulation of biomineral tissue development	8/926	46/17913	0.002242018	0.014121004	0.010421665	84059/655/2201/4057/4745/152816/5027/7020	8
BP	GO:0001764	neuron migration	17/926	152/17913	0.00224532	0.014121004	0.010421665	1233/10752/7852/1630/9201/285313/3675/4139/266727/4929/4897/3084/9423/5649/10371/347733/7422	17
BP	GO:0030168	platelet activation	17/926	152/17913	0.00224532	0.014121004	0.010421665	150/348/139189/2207/83706/2811/3569/3932/3937/5294/23533/5341/5579/5588/6403/6812/7409	17
BP	GO:0002363	alpha-beta T cell lineage commitment	5/926	19/17913	0.00231311	0.014422083	0.010643869	3594/3569/3662/4063/114836	5
BP	GO:0002689	negative regulation of leukocyte chemotaxis	5/926	19/17913	0.00231311	0.014422083	0.010643869	6347/1843/11240/56833/26228	5

© 2020 Yu Y et al. *JAMA Network Open.*

BP	GO:0002710	negative regulation of T cell mediated immunity	5/926	19/17913	0.0023131 1	0.0144220 83	0.010643869	2213/3134/3575/10859/5788	5
BP	GO:0002726	positive regulation of T cell cytokine production	5/926	19/17913	0.0023131 1	0.0144220 83	0.010643869	3553/3569/114548/91543/54440	5
BP	GO:0034104	negative regulation of tissue remodeling	5/926	19/17913	0.0023131 1	0.0144220 83	0.010643869	952/3569/5027/7128/4982	5
BP	GO:0048486	parasympathetic nervous system development	5/926	19/17913	0.0023131 1	0.0144220 83	0.010643869	1959/89797/10371/6899/7020	5
BP	GO:0071294	cellular response to zinc ion	5/926	19/17913	0.0023131 1	0.0144220 83	0.010643869	4489/4493/4499/4501/4502	5
BP	GO:0034765	regulation of ion transmembrane transport	38/926	453/17913	0.0023326 09	0.0145257 93	0.01072041	5243/150/274/777/6347/729230/930/57214/53405/11151/3627/6373/4283/1536/2048/2903/2904/3359/3738/56479/3792/4318/9722/5027/5031/5142/5294/10891/5649/5997/6236/6328/6331/375611/6622/6769/342667/54209	38
B	GO:01	regulation of	18/92	166/1791	0.	0.	0.010964796	146206/6363/6366/1236/23242/139818/3383/688/4117/5027	1

© 2020 Yu Y et al. *JAMA Network Open.*

P	20032	plasma membrane bounded cell projection assembly	6	3	00238968 7	01485692 9		/26499/5365/26051/55607/5880/9750/26228/7454	8
BP	GO:0007259	JAK-STAT cascade	16/926	140/17913	0.0023940 72	0.0148569 29	0.010964796	6347/6352/729230/1436/2261/2322/3059/3587/11009/133396/3569/3718/5788/5979/9021/6775	16
BP	GO:0043154	negative regulation of cysteine-type endopeptidase activity involved in apoptotic process	11/926	79/17913	0.0023945 77	0.0148569 29	0.010964796	939/960/1800/2537/27074/4318/4804/5329/5272/6622/7422	11
BP	GO:0010518	positive regulation of phospholipase activity	9/926	57/17913	0.0024379 03	0.0150704 02	0.011122345	728/6352/2099/2261/3702/5031/5330/6401/7294	9
BP	GO:0045453	bone resorption	9/926	57/17913	0.0024379 03	0.0150704 02	0.011122345	952/1436/53832/3569/5027/5880/7128/4982/8600	9
BP	GO:0046686	response to cadmium ion	9/926	57/17913	0.0024 37	0.0150 70	0.011122345	1536/2729/3162/3725/4318/4489/4493/4501/653361	9

© 2020 Yu Y et al. *JAMA Network Open.*

					903	402			
BP	GO:0016045	detection of bacterium	4/926	12/17913	0.00251482	0.015395678	0.011362407	64127/7096/7097/10333	4
BP	GO:0038110	interleukin-2-mediated signaling pathway	4/926	12/17913	0.00251482	0.015395678	0.011362407	3559/3560/3561/3718	4
BP	GO:0044650	adhesion of symbiont to host cell	4/926	12/17913	0.00251482	0.015395678	0.011362407	30835/10332/3383/5818	4
BP	GO:0051608	histamine transport	4/926	12/17913	0.00251482	0.015395678	0.011362407	100/695/1906/6581	4
BP	GO:0060100	positive regulation of phagocytosis, engulfment	4/926	12/17913	0.00251482	0.015395678	0.011362407	718/948/3071/26228	4
BP	GO:0060707	trophoblast giant cell differentiation	4/926	12/17913	0.00251482	0.01539567	0.011362407	79733/2709/639/9021	4

						8			
BP	GO:0070493	thrombin-activated receptor signaling pathway	4/926	12/17913	0.00251482	0.015395678	0.011362407	2811/10788/5341/6622	4
BP	GO:1905155	positive regulation of membrane invagination	4/926	12/17913	0.00251482	0.015395678	0.011362407	718/948/3071/26228	4
BP	GO:0051590	positive regulation of neurotransmitter transport	7/926	37/17913	0.002543706	0.015553736	0.011479058	8862/8938/8564/5027/6622/6812/8600	7
BP	GO:0031098	stress-activated protein kinase signaling cascade	26/926	278/17913	0.002605816	0.015914315	0.011745174	6363/6366/1236/939/1843/1906/10595/355/2213/3553/11184/653361/64127/4868/5187/26191/10125/10371/140885/6504/10333/8600/23043/7422/9839/7538	26
BP	GO:0002717	positive regulation of natural killer cell mediated immunity	6/926	28/17913	0.002655785	0.016122366	0.011898722	3134/3902/10125/4068/114836/7409	6
BP	GO:0034367	protein-containing complex remodeling	6/926	28/17913	0.002655785	0.016122366	0.011898722	341/348/1071/4018/4023/7941	6

© 2020 Yu Y et al. *JAMA Network Open.*

BP	GO:0034368	protein-lipid complex remodeling	6/926	28/17913	0.002655785	0.016122366	0.011898722	341/348/1071/4018/4023/7941	6
BP	GO:0034369	plasma lipoprotein particle remodeling	6/926	28/17913	0.002655785	0.016122366	0.011898722	341/348/1071/4018/4023/7941	6
BP	GO:2000108	positive regulation of leukocyte apoptotic process	6/926	28/17913	0.002655785	0.016122366	0.011898722	6352/29126/3620/8013/5027/5293	6
BP	GO:0060491	regulation of cell projection assembly	18/926	168/17913	0.002724725	0.016521089	0.01219299	146206/6363/6366/1236/23242/139818/3383/688/4117/5027/26499/5365/26051/55607/5880/9750/26228/7454	18
BP	GO:0006979	response to oxidative stress	37/926	442/17913	0.002738084	0.016582258	0.012238134	100/199/348/374/655/695/6363/1236/948/952/1536/139189/1800/1843/1906/2729/3162/3304/3569/3725/120892/79772/4318/653361/4688/4689/4929/8013/10891/5742/6414/143686/140885/6622/6648/10333/7128	37
BP	GO:0009988	cell-cell recognition	10/926	69/17913	0.002757469	0.016679729	0.012310069	6363/6366/1236/30835/923/10332/1794/84868/4478/5551	10
B	GO:00	Fc receptor	16/92	142/1791	0.	0.	0.012333205	919/917/26999/9844/2209/2212/2213/2214/3055/64005/307	1

P	02431	mediated stimulatory signaling pathway	6	3	00276594 9	01671107 7		1/8013/5788/7409/7454/7456	6
BP	GO:0071356	cellular response to tumor necrosis factor	25/926	266/17913	0.0029201 77	0.0176218 77	0.013005399	9447/27063/330/6347/6348/6352/939/1116/3576/1906/2633/3304/3383/3856/4050/10891/5696/5698/7128/4982/23495/8600/10673/7412/7538	25
BP	GO:0043523	regulation of neuron apoptotic process	21/926	210/17913	0.0029534 88	0.0177018 94	0.013064454	728/6347/6348/10752/11151/112399/2729/3162/3684/3725/4929/8013/26471/5063/10891/54463/6622/6648/6812/7020/7305	21
BP	GO:0042053	regulation of dopamine metabolic process	5/926	20/17913	0.0029543 9	0.0177018 94	0.013064454	3684/3689/4129/4929/6622	5
BP	GO:0042069	regulation of catecholamine metabolic process	5/926	20/17913	0.0029543 9	0.0177018 94	0.013064454	3684/3689/4129/4929/6622	5
BP	GO:0043369	CD4-positive or CD8-positive, alpha-beta T cell lineage commitment	5/926	20/17913	0.0029543 9	0.0177018 94	0.013064454	3594/3569/3662/4063/114836	5
B	GO:00	chemokine	5/926	20/17913	0.	0.	0.013064454	199/1436/9173/90865/4023	5

P	90195	secretion			00<br>29<br>54<br>39	01<br>77<br>01<br>89<br>4			
B<br>P	GO:19<br>05523	positive regulation of macrophage migration	5/926	20/17913	0.<br>00<br>29<br>54<br>39	0.<br>01<br>77<br>01<br>89<br>4	0.013064454	719/728/6348/6352/54209	5
B<br>P	GO:19<br>03727	positive regulation of phospholipi d metabolic process	8/926	48/17913	0.<br>00<br>29<br>60<br>50<br>6	0.<br>01<br>77<br>17<br>59<br>8	0.013076044	6363/6366/1236/930/2261/2322/2358/64127	8
B<br>P	GO:00<br>43547	positive regulation of GTPase activity	30/92<br>6	339/1791<br>3	0.<br>00<br>29<br>67<br>35<br>8	0.<br>01<br>77<br>37<br>66	0.01309085	9744/216/55843/9411/23526/64333/6363/6366/1236/10563/55619/139818/1794/8477/3383/3725/120892/3071/22821/153020/10125/10235/729857/5996/26575/5997/9628/6281/117289/7409	3<br>0
B<br>P	GO:00<br>07520	myoblast fusion	7/926	38/17913	0.<br>00<br>29<br>81<br>39<br>8	0.<br>01<br>77<br>79<br>65<br>7	0.013121845	575/963/50937/3627/4283/4868/9750	7
B<br>P	GO:00<br>32620	interleukin-1<br>7 production	7/926	38/17913	0.<br>00<br>29<br>81<br>39<br>8	0.<br>01<br>77<br>79<br>65<br>7	0.013121845	384/3569/4063/3071/64127/5588/114836	7
B<br>P	GO:00<br>15711	organic anion	36/92<br>6	430/1791<br>3	0.<br>00	0.<br>01	0.013602395	5243/5244/341/348/366/10396/771/768/948/1071/1906/2350/3553/8564/4948/5027/26279/8497/5997/6556/117247/6507/	3<br>6

© 2020 Yu Y et al. *JAMA Network Open.*

		transport			30 94 22	84 30 78 6		375611/6513/9498/159963/6538/6535/6545/9056/6578/1130 9/6622/6812/8600/9256	
BP	GO:00 50728	negative regulation of inflammatory response	18/926	170/17913	0.00 30 98 08 6	0.01 84 32 15 9	0.013603408	54/100/113/348/2213/2358/7850/3559/4210/197358/114548/ 4907/5788/89790/140885/56833/9021/7128	18
BP	GO:19 03169	regulation of calcium ion transmembrane transport	16/926	144/17913	0.00 31 84 45 6	0.01 89 09 27 6	0.013955533	150/274/930/57214/11151/3627/6373/4283/9722/5031/5142/ 5294/6236/6622/6769/342667	16
BP	GO:00 02888	positive regulation of myeloid leukocyte mediated immunity	6/926	29/17913	0.00 32 04 39 3	0.01 89 09 27 6	0.013955533	695/718/2207/3684/3689/6812	6
BP	GO:00 10575	positive regulation of vascular endothelial growth factor production	6/926	29/17913	0.00 32 04 39 3	0.01 89 09 27 6	0.013955533	718/719/728/147372/3553/3569	6
BP	GO:00 21602	cranial nerve morphogenesis	6/926	29/17913	0.00 32 04 39 3	0.01 89 09 27 6	0.013955533	1959/2048/3213/10371/6899/7020	6
BP	GO:00 33006	regulation of mast cell	6/926	29/17913	0.00	0.01	0.013955533	30817/8832/2207/3162/5880/6812	6

© 2020 Yu Y et al. *JAMA Network Open.*

		activation involved in immune response			32 04 39 3	89 09 27 6			
B P	GO:00 48873	homeostasi s of number of cells within a tissue	6/926	29/17913	0. 00 32 04 39 3	0. 01 89 09 27 6	0.013955533	10000/729230/11151/5027/54440/6662	6
B P	GO:20 00778	positive regulation of interleukin-6 secretion	6/926	29/17913	0. 00 32 04 39 3	0. 01 89 09 27 6	0.013955533	199/3553/3556/353514/4023/51311	6
B P	GO:19 90823	response to leukemia inhibitory factor	12/92 6	94/17913	0. 00 32 58 43	0. 01 91 83 48 6	0.014157907	8745/3099/3383/9118/688/7805/4744/11240/26577/9021/98 06/8600	1 2
B P	GO:19 90830	cellular response to leukemia inhibitory factor	12/92 6	94/17913	0. 00 32 58 43	0. 01 91 83 48 6	0.014157907	8745/3099/3383/9118/688/7805/4744/11240/26577/9021/98 06/8600	1 2
B P	GO:19 04062	regulation of cation transmembr ane transport	28/92 6	312/1791 3	0. 00 33 00 85 6	0. 01 94 10 71 7	0.014325609	150/274/6347/729230/930/57214/11151/3627/6373/4283/20 48/2903/2904/3792/4318/9722/5031/5142/5294/10891/5649/ 5997/6236/6331/6622/6769/342667/54209	2 8
B P	GO:00 35272	exocrine system developmen	8/926	49/17913	0. 00 33	0. 01 98	0.01465686	655/3481/284217/4781/10371/6662/29842/7052	8

© 2020 Yu Y et al. *JAMA Network Open.*

		t			81099	59551			
BP	GO:0002262	myeloid cell homeostasis	16/926	145/17913	0.0034125 32	0.0199324 16	0.014710637	301/729230/1021/2207/3059/3162/3216/3304/10320/3569/3071/5142/5293/6688/7422/7538	16
BP	GO:0071774	response to fibroblast growth factor	16/926	145/17913	0.0034125 32	0.0199324 16	0.014710637	8862/6347/6352/960/10563/3576/1960/54845/2261/2729/2817/3164/860/6622/6899/7538	16
BP	GO:0001960	negative regulation of cytokine-mediated signaling pathway	10/926	71/17913	0.0034131 66	0.0199324 16	0.014710637	6352/7850/3569/11213/4321/84166/11240/5788/6091/26228	10
BP	GO:0002312	B cell activation involved in immune response	10/926	71/17913	0.0034131 66	0.0199324 16	0.014710637	100/930/940/55619/139818/2213/1880/9452/23228/5788	10
BP	GO:0030500	regulation of bone mineralization	10/926	71/17913	0.0034131 66	0.0199324 16	0.014710637	84059/655/6348/2201/4057/4745/5027/6662/5552/7020	10
BP	GO:0030278	regulation of ossification	20/926	199/17913	0.003462	0.020051	0.014798883	84059/374/655/6348/1021/1959/2201/54857/3400/8519/3569/4038/4057/4745/5027/860/6662/5552/7020/8626	20

© 2020 Yu Y et al. *JAMA Network Open.*

					407	987			
BP	GO:0034764	positive regulation of transmembrane transport	20/926	199/17913	0.0034624077	0.0200519987	0.014798883	5243/718/6347/729230/930/57214/3627/6373/4283/2048/3359/9722/8013/5027/5031/5649/6622/6769/342667/54209	20
BP	GO:0030501	positive regulation of bone mineralization	7/926	39/17913	0.0034744366	0.0200519987	0.014798883	84059/655/2201/4057/4745/5027/7020	7
BP	GO:0033003	regulation of mast cell activation	7/926	39/17913	0.0034744366	0.0200519987	0.014798883	30817/8832/2207/3162/8013/5880/6812	7
BP	GO:0014902	myotube differentiation	13/926	107/17913	0.0034798788	0.0200519987	0.014798883	575/963/50937/3627/4283/56603/9734/3792/688/50804/4868/9750/6899	13
BP	GO:0002679	respiratory burst involved in defense response	4/926	13/17913	0.0034850666	0.0200519987	0.014798883	3055/5293/5294/56833	4
BP	GO:0032754	positive regulation of interleukin-5 production	4/926	13/17913	0.0034850 6	0.02005198	0.014798883	3556/9173/90865/114548	4

© 2020 Yu Y et al. *JAMA Network Open.*

					6	7			
BP	GO:0034374	low-density lipoprotein particle remodeling	4/926	13/17913	0.000348506 6	0.020051987	0.014798883	348/1071/4018/7941	4
BP	GO:0045073	regulation of chemokine biosynthetic process	4/926	13/17913	0.000348506 6	0.020051987	0.014798883	1958/3162/3553/3569	4
BP	GO:0061888	regulation of astrocyte activation	4/926	13/17913	0.000348506 6	0.020051987	0.014798883	712/728/3569/54209	4
BP	GO:0070431	nucleotide-binding oligomerization domain containing 2 signaling pathway	4/926	13/17913	0.000348506 6	0.020051987	0.014798883	3304/64127/26191/7128	4
BP	GO:0071352	cellular response to interleukin-2	4/926	13/17913	0.000348506 6	0.020051987	0.014798883	3559/3560/3561/3718	4
BP	GO:0098543	detection of other organism	4/926	13/17913	0.000348506	0.0200519 8	0.014798883	64127/7096/7097/10333	4

© 2020 Yu Y et al. *JAMA Network Open.*

					6	7			
BP	GO:0043123	positive regulation of I-kappaB kinase/NF-kappaB signaling	18/926	172/17913	0.0035130 5	0.0201900 83	0.014900801	330/6363/6366/1236/948/972/51704/3162/4057/64127/11040/5579/6398/7052/10333/8600/10346/10537	18
BP	GO:0006925	inflammatory cell apoptotic process	5/926	21/17913	0.0037147 3	0.0212527 81	0.0156851	6352/1514/3569/64127/5293	5
BP	GO:0010884	positive regulation of lipid storage	5/926	21/17913	0.0037147 3	0.0212527 81	0.0156851	718/948/29923/4023/4481	5
BP	GO:0032928	regulation of superoxide anion generation	5/926	21/17913	0.0037147 3	0.0212527 81	0.0156851	54/2358/3684/3689/7305	5
BP	GO:0051589	negative regulation of neurotransmitter transport	5/926	21/17913	0.0037147 3	0.0212527 81	0.0156851	10859/4129/55607/5997/6622	5
BP	GO:0031667	response to nutrient levels	38/926	466/17913	0.0037699 04	0.0215441 31	0.015900124	100/122622/249/348/467/655/717/916/3627/1536/56603/1824/355/2350/2729/3162/3383/3428/3553/3725/57535/4023/120892/4316/64127/5027/5029/5251/10891/58528/143686/6581/6513/6696/7054/4982/7412/7538	38

© 2020 Yu Y et al. *JAMA Network Open.*

BP	GO:0007202	activation of phospholipase C activity	6/926	30/17913	0.003833192	0.021831969	0.016112556	728/3702/5031/5330/6401/7294	6
BP	GO:0021772	olfactory bulb development	6/926	30/17913	0.003833192	0.021831969	0.016112556	1436/1749/8320/120892/6091/10371	6
BP	GO:0050690	regulation of defense response to virus by virus	6/926	30/17913	0.003833192	0.021831969	0.016112556	919/940/1794/9844/3055/3932	6
BP	GO:0002931	response to ischemia	8/926	50/17913	0.003846576	0.021859071	0.016132558	1436/1958/3099/5027/10891/375611/54209/7345	8
BP	GO:0050771	negative regulation of axonogenesis	8/926	50/17913	0.003846576	0.021859071	0.016132558	1630/2048/23105/4038/4804/9423/5800/6696	8
BP	GO:0018958	phenol-containing compound metabolic process	12/926	96/17913	0.003880651	0.022003378	0.01623906	2903/3684/3689/4129/4929/4948/6622/9627/6799/27284/7054/9839	12
B	GO:00	regulation of	12/92	96/17913	0.	0.	0.01623906	107/321/8938/8564/120892/79772/55607/5579/6622/9627/6	1

© 2020 Yu Y et al. *JAMA Network Open.*

P	46928	neurotransmitter secretion	6		003880651	022003378		812/91683	2
BP	GO:0006865	amino acid transport	15/926	134/17913	0.003930903	0.022263402	0.016430964	8564/4948/5027/8497/5997/6556/117247/6507/6538/6535/6545/9056/6622/6812/9256	15
BP	GO:0019835	cytolysis	7/926	40/17913	0.004027157	0.02273236	0.016777068	730/3001/3002/10859/4069/5027/5551	7
BP	GO:0051281	positive regulation of release of sequestered calcium ion into cytosol	7/926	40/17913	0.004027157	0.02273236	0.016777068	930/57214/3627/6373/4283/5031/6622	7
BP	GO:2000273	positive regulation of signaling receptor activity	7/926	40/17913	0.004027157	0.02273236	0.016777068	150/6347/729230/2048/2069/653361/5649	7
BP	GO:0042737	drug catabolic process	13/926	109/17913	0.004087658	0.023048206	0.01701017	100/51816/217/1118/1557/1577/1806/259307/4129/4907/6622/6799/27284	13
BP	GO:0016358	dendrite developmen	21/926	216/17913	0.00	0.02	0.017077405	348/655/916/1952/23242/1630/9201/55619/2048/23105/23426/57549/4038/120892/4139/5063/55607/5649/10371/80725	21

© 2020 Yu Y et al. *JAMA Network Open.*

		t			4112945	3139307		/23043	
BP	GO:0048588	developmental cell growth	21/926	216/17913	0.004112945	0.023139307	0.017077405	348/23242/7852/26999/1630/9201/1906/23105/3676/3897/23566/4897/9423/10439/5997/10371/6662/6696/51760/7422/9839	21
BP	GO:0042552	myelination	14/926	122/17913	0.004192716	0.023561949	0.017389325	23205/7852/1959/23136/2817/3400/3687/3792/5653/10397/3084/7097/1890/7368	14
BP	GO:0106027	neuron projection organization	11/926	85/17913	0.004273318	0.023988318	0.017703997	348/55619/2048/2213/2904/3675/120892/5063/55607/5649/80725	11
BP	GO:0002712	regulation of B cell mediated immunity	8/926	51/17913	0.004360011	0.024420882	0.01802324	695/718/940/1378/2207/2208/2213/5788	8
BP	GO:0002889	regulation of immunoglobulin mediated immune response	8/926	51/17913	0.004360011	0.024420882	0.01802324	695/718/940/1378/2207/2208/2213/5788	8
BP	GO:0032479	regulation of type I interferon	14/926	123/17913	0.0045	0.0252	0.018631847	84868/51191/3428/10859/4321/197358/84166/26191/140885/7097/51284/51311/7128/81030	14

		production			125557	45524			
BP	GO:1901653	cellular response to peptide	31/926	364/17913	0.004517201	0.025245524	0.018631847	107/113/948/1906/2213/2358/2729/9734/3383/3481/3553/3676/3718/688/4023/51237/4804/64127/3164/4929/8013/5140/5577/5579/5588/26191/143686/10333/54209/7253/7412	31
BP	GO:0097305	response to alcohol	22/926	232/17913	0.004537873	0.025333125	0.018696499	107/113/6363/6366/6348/1236/929/939/999/1536/2903/2904/3359/3383/4129/11184/5031/5997/6279/6688/7054/7412	22
BP	GO:0021988	olfactory lobe development	6/926	31/17913	0.004548933	0.025339058	0.018700878	1436/1749/8320/120892/6091/10371	6
BP	GO:0035587	purinergic receptor signaling pathway	6/926	31/17913	0.004548933	0.025339058	0.018700878	100/29909/5027/27334/5029/5031	6
BP	GO:0099175	regulation of postsynapse organization	12/926	98/17913	0.004594973	0.025352678	0.01871093	348/2213/2904/3556/10288/4038/120892/4897/5063/55607/5649/80725	12
BP	GO:0033622	integrin activation	5/926	22/17913	0.00460 5	0.025352	0.01871093	10563/83706/51237/5341/6403	5

© 2020 Yu Y et al. *JAMA Network Open.*

					625	678			
BP	GO:0035590	purinergic nucleotide receptor signaling pathway	5/926	22/17913	0.0046056625	0.0253526678	0.01871093	29909/5027/27334/5029/5031	5
BP	GO:0043302	positive regulation of leukocyte degranulation	5/926	22/17913	0.0046056625	0.0253526678	0.01871093	2207/3134/3684/3689/6812	5
BP	GO:0045117	azole transport	5/926	22/17913	0.0046056625	0.0253526678	0.01871093	100/695/1906/80704/6581	5
BP	GO:0048305	immunoglobulin secretion	5/926	22/17913	0.0046056625	0.0253526678	0.01871093	933/90865/3569/54900/10673	5
BP	GO:0050927	positive regulation of positive chemotaxis	5/926	22/17913	0.0046056625	0.0253526678	0.01871093	1233/3576/2152/3603/7422	5
BP	GO:0050995	negative regulation of lipid catabolic process	5/926	22/17913	0.00460562	0.02535267	0.01871093	150/341/3553/5140/5294	5

					5	8			
BP	GO:1900016	negative regulation of cytokine production involved in inflammatory response	5/926	22/17913	0.004605625	0.025352678	0.01871093	113/7850/4210/197358/140885	5
BP	GO:0006692	prostanoid metabolic process	7/926	41/17913	0.004643983	0.025352678	0.01871093	972/1906/2171/3553/5730/5742/6916	7
BP	GO:0006693	prostaglandin metabolic process	7/926	41/17913	0.004643983	0.025352678	0.01871093	972/1906/2171/3553/5730/5742/6916	7
BP	GO:0050830	defense response to Gram-positive bacterium	11/926	86/17913	0.004677184	0.025352678	0.01871093	54/728/948/84868/3569/4069/219972/114548/5027/55576/7097	11
BP	GO:1903725	regulation of phospholipid metabolic process	11/926	86/17913	0.004677184	0.025352678	0.01871093	341/6363/6366/1236/930/2261/2322/2358/64127/23533/9021	11
BP	GO:0002523	leukocyte migration involved in inflammatory response	4/926	14/17913	0.0046818141	0.0253525267	0.01871093	3689/6279/6401/56833	4

© 2020 Yu Y et al. *JAMA Network Open.*

					8	8			
BP	GO:0006837	serotonin transport	4/926	14/17913	0.004681418	0.025352678	0.01871093	2207/10859/4129/6622	4
BP	GO:0006910	phagocytosis, recognition	4/926	14/17913	0.004681418	0.025352678	0.01871093	575/948/64581/2219	4
BP	GO:0010820	positive regulation of T cell chemotaxis	4/926	14/17913	0.004681418	0.025352678	0.01871093	6366/6352/729230/10563	4
BP	GO:0014048	regulation of glutamate secretion	4/926	14/17913	0.004681418	0.025352678	0.01871093	8564/5027/6622/6812	4
BP	GO:0032310	prostaglandin secretion	4/926	14/17913	0.004681418	0.025352678	0.01871093	1906/3553/5027/8600	4
BP	GO:0035589	G protein-coupled purinergic nucleotide receptor	4/926	14/17913	0.004681418	0.025352678	0.01871093	29909/27334/5029/5031	4

© 2020 Yu Y et al. *JAMA Network Open.*

		signaling pathway							
BP	GO:0042033	chemokine biosynthetic process	4/926	14/17913	0.004681418	0.025352678	0.01871093	1958/3162/3553/3569	4
BP	GO:0045953	negative regulation of natural killer cell mediated cytotoxicity	4/926	14/17913	0.004681418	0.025352678	0.01871093	84868/3134/10859/5272	4
BP	GO:0050755	chemokine metabolic process	4/926	14/17913	0.004681418	0.025352678	0.01871093	1958/3162/3553/3569	4
BP	GO:0060099	regulation of phagocytosis, engulfment	4/926	14/17913	0.004681418	0.025352678	0.01871093	718/948/3071/26228	4
BP	GO:0070669	response to interleukin-2	4/926	14/17913	0.004681418	0.025352678	0.01871093	3559/3560/3561/3718	4
BP	GO:0097202	activation of cysteine-type endopeptidase activity	4/926	14/17913	0.00468141	0.02535267	0.01871093	26999/2903/3428/64065	4

© 2020 Yu Y et al. *JAMA Network Open.*

					8	8			
BP	GO:0051271	negative regulation of cellular component movement	31/926	365/17913	0.004700499	0.025428846	0.018767144	100/56999/575/84830/199/348/274/6347/6366/999/10563/1800/1843/83481/3162/8519/11009/90865/79772/3084/11240/5365/10891/5800/9750/6091/10371/56833/50859/26228/7454	31
BP	GO:1901654	response to ketone	19/926	191/17913	0.004818964	0.026041927	0.019219614	5244/107/768/6363/6366/1236/952/1536/1829/1843/1906/3383/4129/11184/4478/5031/6696/7054/7097	19
BP	GO:0051495	positive regulation of cytoskeleton organization	20/926	205/17913	0.004838578	0.026120077	0.01927729	274/146206/6366/1236/11151/1906/342184/8477/3055/3059/3304/3383/10788/3071/4868/5027/5341/6281/26228/7454	20
BP	GO:1905952	regulation of lipid localization	16/926	151/17913	0.005080493	0.027396826	0.020219564	5244/341/348/10396/718/948/1071/1906/29923/3553/3569/4023/4481/5027/6696/8600	16
BP	GO:0032606	type I interferon production	14/926	125/17913	0.005211474	0.028073283	0.020718807	84868/51191/3428/10859/4321/197358/84166/26191/140885/7097/51284/51311/7128/81030	14
BP	GO:0015849	organic acid transport	26/926	293/17913	0.005241819	0.028176861	0.02079525	5244/348/366/948/1906/2350/3553/8564/4948/5027/26279/8497/5997/6556/117247/6507/375611/159963/6538/6535/6545/9056/6622/6812/8600/9256	26

© 2020 Yu Y et al. *JAMA Network Open.*

BP	GO:0046942	carboxylic acid transport	26/926	293/17913	0.005241819	0.028176861	0.02079525	5244/348/366/948/1906/2350/3553/8564/4948/5027/26279/8497/5997/6556/117247/6507/375611/159963/6538/6535/6545/9056/6622/6812/8600/9256	26
BP	GO:0051604	protein maturation	30/926	353/17913	0.005313946	0.028534316	0.02105906	712/713/714/717/718/719/720/721/728/730/147372/930/629/3426/1378/1380/26999/2152/2903/3428/3553/7850/5653/120892/5027/64065/710/5552/55061/11326	30
BP	GO:0010863	positive regulation of phospholipase C activity	7/926	42/17913	0.005329394	0.028556764	0.021075628	728/2099/3702/5031/5330/6401/7294	7
BP	GO:0014047	glutamate secretion	7/926	42/17913	0.005329394	0.028556764	0.021075628	8564/5027/8497/6507/6622/6812/9256	7
BP	GO:0043409	negative regulation of MAPK cascade	18/926	179/17913	0.005344155	0.028605621	0.021111685	348/467/655/1843/2048/2633/3553/11213/54900/5027/5187/26191/5788/5997/27330/57556/140885/7345	18
BP	GO:0009164	nucleoside catabolic process	6/926	32/17913	0.005358411	0.028621482	0.021123392	100/51816/978/1806/4907/1890	6
B	GO:00	myeloid cell	6/926	32/17913	0.	0.	0.021123392	6352/1514/2207/3569/64127/5293	6

© 2020 Yu Y et al. *JAMA Network Open.*

P	33028	apoptotic process			0053584111	0286214822			
BP	GO:0060135	maternal process involved in female pregnancy	9/926	64/17913	0.0054138836	0.0288870 9	0.021319417	113/1829/2099/84868/3675/4316/639/5997/6696	9
BP	GO:0090596	sensory organ morphogenesis	23/926	250/17913	0.005438133	0.0289862 2	0.021392578	655/50937/56603/1749/1906/2048/2201/26018/4647/5818/8013/9423/639/9750/54549/84189/6662/64220/6899/7020/7054/7068/7422	23
BP	GO:0002433	immune response-regulating cell surface receptor signaling pathway involved in phagocytosis	15/926	139/17913	0.005535941	0.029392595	0.021692493	919/917/26999/9844/2209/2212/2213/2214/3055/64005/3071/5788/7409/7454/7456	15
BP	GO:0038096	Fc-gamma receptor signaling pathway involved in phagocytosis	15/926	139/17913	0.005535941	0.029392595	0.021692493	919/917/26999/9844/2209/2212/2213/2214/3055/64005/3071/5788/7409/7454/7456	15
BP	GO:0051384	response to glucocorticoid	15/926	139/17913	0.0055	0.0293	0.021692493	21/23205/199/249/301/374/1843/1906/2322/3383/3569/4129/5069/7054/7538	15

© 2020 Yu Y et al. *JAMA Network Open.*

					35941	92595			
BP	GO:0001655	urogenital system development	28/926	324/17913	0.005542867	0.029392595	0.021692493	384/655/1285/1286/1396/56603/1958/2048/2099/342184/2297/2650/3400/3675/3691/4038/120892/4318/4868/5017/5800/5979/5268/6662/64220/7020/8626/7422	28
BP	GO:0046456	icosanoid biosynthetic process	8/926	53/17913	0.005543366	0.029392595	0.021692493	241/972/1906/2171/3553/5730/5742/6916	8
BP	GO:0070167	regulation of biomineral tissue development	11/926	88/17913	0.005575443	0.029500959	0.021772468	84059/655/6348/2201/4057/4745/152816/5027/6662/5552/7020	11
BP	GO:1904894	positive regulation of STAT cascade	11/926	88/17913	0.005575443	0.029500959	0.021772468	6352/1436/2261/2322/3059/3587/11009/133396/3569/3575/9021	11
BP	GO:0007272	ensheathment of neurons	14/926	126/17913	0.005592248	0.029528235	0.021792598	23205/7852/1959/23136/2817/3400/3687/3792/5653/10397/3084/7097/1890/7368	14
BP	GO:0008366	axon ensheathment	14/926	126/17913	0.005592	0.029528	0.021792598	23205/7852/1959/23136/2817/3400/3687/3792/5653/10397/3084/7097/1890/7368	14

© 2020 Yu Y et al. *JAMA Network Open.*

					248	235			
BP	GO:0010758	regulation of macrophage chemotaxis	5/926	23/17913	0.0056384 43	0.0296178 95	0.02185877	719/728/6352/56833/26228	5
BP	GO:0034123	positive regulation of toll-like receptor signaling pathway	5/926	23/17913	0.0056384 43	0.0296178 95	0.02185877	4057/26191/91543/7096/7097	5
BP	GO:0035588	G protein-coupled purinergic receptor signaling pathway	5/926	23/17913	0.0056384 43	0.0296178 95	0.02185877	100/29909/27334/5029/5031	5
BP	GO:0050926	regulation of positive chemotaxis	5/926	23/17913	0.0056384 43	0.0296178 95	0.02185877	1233/3576/2152/3603/7422	5
BP	GO:0051968	positive regulation of synaptic transmission, glutamatergic	5/926	23/17913	0.0056384 43	0.0296178 95	0.02185877	6347/729230/8564/5649/6812	5
BP	GO:0032271	regulation of protein polymerizati	18/926	180/17913	0.0056	0.0297	0.021920103	119/274/146206/6366/1236/11151/342184/3055/3059/3304/3383/10788/3071/4868/5063/55607/6622/7454	18

© 2020 Yu Y et al. *JAMA Network Open.*

		on			60123	00999			
BP	GO:0031668	cellular response to extracellular stimulus	23/926	251/17913	0.005703622	0.029898305	0.022065719	199/467/1536/1824/355/2350/3162/3383/3428/3676/3725/57535/4023/120892/4929/5027/5029/5251/5788/58528/143686/6513/7412	23
BP	GO:0019233	sensory perception of pain	12/926	101/17913	0.005859618	0.030684302	0.022645805	6347/6348/729230/7852/1906/2171/2903/3218/4886/5027/54463/7345	12
BP	GO:1904064	positive regulation of cation transmembrane transport	15/926	140/17913	0.005912965	0.030931703	0.022828394	6347/729230/930/57214/3627/6373/4283/2048/9722/5031/5649/6622/6769/342667/54209	15
BP	GO:0045471	response to ethanol	14/926	127/17913	0.005995059	0.031305926	0.02310458	113/929/939/1536/2903/2904/3359/3383/4129/5997/6279/6688/7054/7412	14
BP	GO:0035924	cellular response to vascular endothelial growth factor stimulus	9/926	65/17913	0.0060030303	0.031305926	0.02310458	301/147372/1960/2322/3164/6091/57556/7412/7422	9
BP	GO:0048864	stem cell development	9/926	65/17913	0.0060	0.0313	0.02310458	655/1906/3084/5788/5979/10371/6662/6899/9839	9

© 2020 Yu Y et al. *JAMA Network Open.*

					0303	05926			
BP	GO:0051602	response to electrical stimulus	7/926	43/17913	0.0060 8792	0.03161989 8	0.0233363	122622/199/929/1917/5027/10891/7054	7
BP	GO:0002693	positive regulation of cellular extravasation	4/926	15/17913	0.0061 25615	0.03161989 8	0.0233363	729230/3383/3676/9750	4
BP	GO:0015732	prostaglandin transport	4/926	15/17913	0.0061 25615	0.03161989 8	0.0233363	1906/3553/5027/8600	4
BP	GO:0032645	regulation of granulocyte macrophage colony-stimulating factor production	4/926	15/17913	0.0061 25615	0.03161989 8	0.0233363	941/8832/3553/10125	4
BP	GO:0032736	positive regulation of interleukin-13 production	4/926	15/17913	0.0061 25615	0.03161989 8	0.0233363	3556/90865/3662/114548	4
BP	GO:0042535	positive regulation of tumor	4/926	15/17913	0.0061	0.0316	0.0233363	729230/1536/7096/7305	4

© 2020 Yu Y et al. *JAMA Network Open.*

		necrosis factor biosynthetic process			25615	198998			
BP	GO:1900451	positive regulation of glutamate receptor signaling pathway	4/926	15/17913	0.00612561 5	0.031619898	0.0233363	6347/729230/2048/5649	4
BP	GO:1901741	positive regulation of myoblast fusion	4/926	15/17913	0.006125615	0.031619898	0.0233363	575/963/4283/9750	4
BP	GO:1905153	regulation of membrane invagination	4/926	15/17913	0.006125615	0.031619898	0.0233363	718/948/3071/26228	4
BP	GO:2000402	negative regulation of lymphocyte migration	4/926	15/17913	0.006125615	0.031619898	0.0233363	84830/6347/11240/9750	4
BP	GO:0034109	homotypic cell-cell adhesion	10/926	77/17913	0.006145742	0.031627175	0.02334167	6352/1525/1824/1829/83706/5294/5341/5588/6812/8600	10
BP	GO:0060761	negative regulation of response to	10/926	77/17913	0.0061	0.0316	0.02334167	6352/7850/3569/11213/4321/84166/11240/5788/6091/26228	10

© 2020 Yu Y et al. *JAMA Network Open.*

		cytokine stimulus			45742	27175			
BP	GO:0071260	cellular response to mechanical stimulus	10/926	77/17913	0.0061457 42	0.0316271 75	0.02334167	27063/355/2729/3553/4316/9750/6513/6662/51284/51311	10
BP	GO:0010524	positive regulation of calcium ion transport into cytosol	8/926	54/17913	0.006219708	0.031942956	0.023574725	930/57214/3627/6373/4283/5027/5031/6622	8
BP	GO:0060760	positive regulation of response to cytokine stimulus	8/926	54/17913	0.006219708	0.031942956	0.023574725	972/1906/3304/4321/84166/7097/54209/7294	8
BP	GO:0032892	positive regulation of organic acid transport	6/926	33/17913	0.006268431	0.032160605	0.023735356	1906/3553/8564/5027/6812/8600	6
BP	GO:0038094	Fc-gamma receptor signaling pathway	15/926	141/17913	0.006310408	0.032343236	0.023870141	919/917/26999/9844/2209/2212/2213/2214/3055/64005/3071/5788/7409/7454/7456	15
BP	GO:0006690	icosanoid metabolic process	12/926	102/17913	0.006337	0.032449	0.023948579	241/972/1557/1573/1800/1906/2171/3553/5730/5742/6916/7097	12

© 2020 Yu Y et al. *JAMA Network Open.*

					546	516			
BP	GO:0031960	response to corticosteroid	16/926	155/17913	0.000652269 3	0.033363807	0.024623349	21/23205/199/249/301/374/1536/1843/1906/2322/3383/3569/4129/5069/7054/7538	16
BP	GO:0061387	regulation of extent of cell growth	11/926	90/17913	0.000660438 7	0.033747 62	0.024906613	348/1630/23105/3897/23566/4897/9423/10439/10371/6696/7422	11
BP	GO:0022617	extracellular matrix disassembly	10/926	78/17913	0.000673191 7	0.034364641	0.025361991	146206/960/1514/1803/3569/5650/3936/4321/4316/4318	10
BP	GO:0021952	central nervous system projection neuron axonogenesis	5/926	24/17913	0.000682433 2	0.034731467	0.025632718	1630/9201/4781/4929/9839	5
BP	GO:0033081	regulation of T cell differentiation in thymus	5/926	24/17913	0.000682433 2	0.034731467	0.025632718	100/1960/3575/10125/9840	5
BP	GO:0042481	regulation of odontogenesis	5/926	24/17913	0.00068 24	0.034731	0.025632718	1906/152816/860/7020/4982	5

© 2020 Yu Y et al. *JAMA Network Open.*

					332	467			
BP	GO:1904892	regulation of STAT cascade	14/926	129/17913	0.006870357	0.034930629	0.025779705	6352/1436/2261/2322/3059/3587/11009/133396/3569/3575/3718/5788/5979/9021	14
BP	GO:0032273	positive regulation of protein polymerization	13/926	116/17913	0.006920424	0.035063277	0.025877602	274/146206/6366/1236/11151/342184/3055/3304/3383/10788/3071/4868/7454	13
BP	GO:0001754	eye photoreceptor cell differentiation	7/926	44/17913	0.006924115	0.035063277	0.025877602	4647/639/54549/6662/7054/7068/7422	7
BP	GO:0030850	prostate gland development	7/926	44/17913	0.006924115	0.035063277	0.025877602	655/1396/2099/3400/5268/6662/8626	7
BP	GO:1900274	regulation of phospholipase C activity	7/926	44/17913	0.006924115	0.035063277	0.025877602	728/2099/3702/5031/5330/6401/7294	7
BP	GO:0001101	response to acid chemical	28/926	330/17913	0.00707208	0.03577684	0.026404236	5244/6363/6366/1236/948/952/1536/56603/1843/1906/1958/2350/2729/2707/3383/3856/4023/5031/10891/5979/58528/375611/6662/6899/8463/7054/7097/7422	28

© 2020 Yu Y et al. *JAMA Network Open.*

					6	8			
BP	GO:0042531	positive regulation of tyrosine phosphorylation of STAT protein	9/926	67/17913	0.007328412	0.037036611	0.027333974	6352/1436/2261/2322/3059/11009/133396/3569/9021	9
BP	GO:0006766	vitamin metabolic process	14/926	130/17913	0.007344663	0.03708177	0.027367302	54/316/8029/56603/1593/2350/2729/3553/8564/5947/80704/6513/6515/8875	14
BP	GO:0002761	regulation of myeloid leukocyte differentiation	13/926	117/17913	0.007428317	0.037466802	0.027651466	714/6348/972/1021/2124/3059/3725/10859/11006/4057/8600/54209/7305	13
BP	GO:1902905	positive regulation of supramolecular fiber organization	18/926	185/17913	0.007476794	0.037673826	0.027804254	348/274/146206/6366/1236/11151/1906/342184/8477/3055/3304/3383/10788/3071/4868/5341/6281/7454	18
BP	GO:2000146	negative regulation of cell motility	29/926	347/17913	0.007598436	0.038248731	0.028228549	100/56999/575/84830/199/348/6347/6366/999/10563/1800/1843/83481/3162/8519/11009/90865/79772/3084/11240/5365/10891/9750/6091/10371/56833/50859/26228/7454	29
BP	GO:0033619	membrane protein proteolysis	8/926	56/17913	0.007758047	0.039013432	0.028792919	8728/150/348/3553/4804/5027/5588/5979	8

© 2020 Yu Y et al. *JAMA Network Open.*

BP	GO:0001916	positive regulation of T cell mediated cytotoxicity	4/926	16/17913	0.007837865	0.03916637	0.02890579	3134/3594/5027/5788	4
BP	GO:0032305	positive regulation of icosanoid secretion	4/926	16/17913	0.007837865	0.03916637	0.02890579	1906/3553/5027/8600	4
BP	GO:0032604	granulocyte macrophage colony-stimulating factor production	4/926	16/17913	0.007837865	0.03916637	0.02890579	941/8832/3553/10125	4
BP	GO:0036035	osteoclast development	4/926	16/17913	0.007837865	0.03916637	0.02890579	10859/4057/8600/7305	4
BP	GO:0051482	positive regulation of cytosolic calcium ion concentration involved in phospholipase C-activating G protein-coupled signaling pathway	4/926	16/17913	0.007837865	0.03916637	0.02890579	719/1906/23566/7052	4

© 2020 Yu Y et al. *JAMA Network Open.*

BP	GO:0060192	negative regulation of lipase activity	4/926	16/17913	0.007837865	0.0391666337	0.02890579	653145/341/9734/5997	4
BP	GO:0035987	endodermal cell differentiation	7/926	45/17913	0.007842546	0.0391666337	0.02890579	8320/8091/3676/3689/3909/3914/4318	7
BP	GO:0007416	synapse assembly	17/926	172/17913	0.007887842	0.0393935381	0.029044126	119/575/2048/3556/4038/266727/5818/4897/3084/9423/55607/54549/22941/84189/6622/9806/7097	17
BP	GO:0007584	response to nutrient	20/926	215/17913	0.008116584	0.0404045226	0.029856999	100/249/655/717/916/3627/1536/56603/2350/2729/3162/3553/4023/64127/5027/5029/5251/6696/4982/7412	20
BP	GO:0031664	regulation of lipopolysaccharide-mediated signaling pathway	5/926	25/17913	0.008174134	0.0405427746	0.029921591	4064/948/4057/23643/7128	5
BP	GO:0035640	exploration behavior	5/926	25/17913	0.008174134	0.0405427746	0.029921591	348/10752/1803/3675/120892	5
B	GO:00	regulation of	5/926	25/17913	0.	0.	0.029921591	2069/3553/7096/10333/7305	5

P	45408	interleukin-6 biosynthetic process			008174134	040542746			
BP	GO:0060669	embryonic placenta morphogenesis	5/926	25/17913	0.008174134	0.040542746	0.029921591	655/2709/79977/3481/9021	5
BP	GO:0060740	prostate gland epithelium morphogenesis	5/926	25/17913	0.008174134	0.040542746	0.029921591	655/2099/3400/6662/8626	5
BP	GO:0010831	positive regulation of myotube differentiation	6/926	35/17913	0.008417135	0.041639331	0.030730899	575/963/4283/56603/9750/6899	6
BP	GO:0048710	regulation of astrocyte differentiation	6/926	35/17913	0.008417135	0.041639331	0.030730899	274/712/728/3400/3569/54209	6
BP	GO:0048010	vascular endothelial growth factor receptor signaling pathway	11/926	93/17913	0.008419869	0.041639331	0.030730899	1536/26999/9844/3553/653361/4688/4689/3071/5579/7409/7422	11
B	GO:00	regulation of	8/926	57/17913	0.	0.	0.031454894	1906/3553/8564/5027/5997/6622/6812/8600	8

© 2020 Yu Y et al. *JAMA Network Open.*

P	32890	organic acid transport			008626642	042620319			
BP	GO:0014075	response to amine	7/926	46/17913	0.008847769	0.043627763	0.032198413	2903/9734/3383/4929/26575/5997/7054	7
BP	GO:0032480	negative regulation of type I interferon production	7/926	46/17913	0.008847769	0.043627763	0.032198413	84868/51191/10859/197358/84166/140885/7128	7
BP	GO:2001259	positive regulation of cation channel activity	9/926	69/17913	0.008866385	0.043677072	0.032234805	6347/729230/2048/9722/5031/5649/6769/342667/54209	9
BP	GO:0021537	telencephalon development	22/926	246/17913	0.008893647	0.043768831	0.032302525	306/597/1021/50937/1436/7852/1749/8320/2048/3400/120892/4781/8013/3084/5079/5649/6091/6331/10371/6513/7054/9839	22
BP	GO:0048562	embryonic organ morphogenesis	24/926	276/17913	0.00905403	0.044514915	0.032853155	655/50515/1749/1906/2048/2201/79977/3215/3216/3218/26018/4647/8013/9423/5079/9750/860/84189/6662/64220/6899/8463/7020/7054	24
BP	GO:0030316	osteoclast differentiatio	11/926	94/17913	0.00	0.04	0.033002155	6348/135228/1436/2207/1880/10859/11006/4057/8600/54209/7305	11

© 2020 Yu Y et al. *JAMA Network Open.*

		n			91039159 5	47168068 6			
BP	GO:0046425	regulation of JAK-STAT cascade	13/926	120/17913	0.0091304911	0.0448039288	0.033066453	6352/1436/2261/2322/3059/3587/11009/133396/3569/3718/5788/5979/9021	13
BP	GO:0007260	tyrosine phosphorylation of STAT protein	10/926	82/17913	0.0095205277	0.0466726822	0.034445642	6352/1436/2261/2322/3059/11009/133396/3569/3718/9021	10
BP	GO:0032370	positive regulation of lipid transport	8/926	58/17913	0.0095659333	0.0468499644	0.034576481	5244/348/10396/1906/3553/5027/6696/8600	8
BP	GO:0002360	T cell lineage commitment	5/926	26/17913	0.0096983066	0.0472461044	0.034868843	3594/3569/3662/4063/114836	5
BP	GO:0002691	regulation of cellular extravasation	5/926	26/17913	0.0096983066	0.0472461044	0.034868843	6366/729230/3383/3676/9750	5
BP	GO:0010996	response to auditory stimulus	5/926	26/17913	0.0096	0.0472	0.034868843	3627/6507/375611/84189/64220	5

© 2020 Yu Y et al. *JAMA Network Open.*

					98306	46104			
BP	GO:0032647	regulation of interferon-alpha production	5/926	26/17913	0.0096983 06	0.0472461 04	0.034868843	84868/4321/197358/51284/51311	5
BP	GO:0034114	regulation of heterotypic cell-cell adhesion	5/926	26/17913	0.0096983 06	0.0472461 04	0.034868843	655/960/2651/3553/3932	5
BP	GO:0007200	phospholipase C-activating G protein-coupled receptor signaling pathway	9/926	70/17913	0.0097213 82	0.0472461 04	0.034868843	150/719/1906/2099/2357/23566/5029/5031/7052	9
BP	GO:0060193	positive regulation of lipase activity	9/926	70/17913	0.0097213 82	0.0472461 04	0.034868843	728/6352/2099/2261/3702/5031/5330/6401/7294	9
BP	GO:1904427	positive regulation of calcium ion transmembrane transport	9/926	70/17913	0.0097213 82	0.0472461 04	0.034868843	930/57214/3627/6373/4283/5031/6622/6769/342667	9
BP	GO:0051146	striated muscle cell	23/926	263/17913	0.00	0.04	0.035080602	575/27063/963/50937/1525/3627/4283/56603/1906/9734/3481/3792/688/3856/50804/4868/3084/5997/9750/6899/7273/7	23

© 2020 Yu Y et al. *JAMA Network Open.*

		differentiation			9811951	75330031		345/7422	
BP	GO:0007188	adenylate cyclase-modulating G protein-coupled receptor signaling pathway	16/926	162/17913	0.0098275 01	0.04753303 1	0.035080602	107/113/150/3627/6373/4283/1906/2357/2358/8477/27201/8685/4886/5996/5997/7253	16
BP	GO:0042472	inner ear morphogenesis	11/926	95/17913	0.00983008 8	0.04753303 1	0.035080602	1749/2048/26018/4647/8013/9423/9750/84189/6662/6899/7020	11
BP	GO:0032930	positive regulation of superoxide anion generation	4/926	17/17913	0.00983668 4	0.04753303 1	0.035080602	2358/3684/3689/7305	4
BP	GO:0048535	lymph node development	4/926	17/17913	0.00983668 4	0.04753303 1	0.035080602	3575/4050/124976/9760	4
BP	GO:1904996	positive regulation of leukocyte adhesion to vascular endothelial cell	4/926	17/17913	0.00983668 4	0.04753303 1	0.035080602	729230/3383/3676/3689	4
B	GO:00	positive	12/92	108/1791	0.	0.	0.035224677	5243/6347/729230/2048/3359/9722/5031/5649/6446/6769/3	1

P	32411	regulation of transporter activity	6	3	009886498	047728248		42667/54209	2
BP	GO:0001961	positive regulation of cytokine-mediated signaling pathway	7/926	47/17913	0.009944315	0.047916098	0.035363315	972/1906/3304/4321/84166/54209/7294	7
BP	GO:0002832	negative regulation of response to biotic stimulus	7/926	47/17913	0.009944315	0.047916098	0.035363315	384/10875/84868/3559/4057/11005/7128	7
BP	GO:0007269	neurotransmitter secretion	15/926	149/17913	0.010320338	0.049680919	0.036665799	107/321/8938/8564/120892/79772/5027/8497/55607/5579/6622/9627/6812/91683/9256	15
CC	GO:0098552	side of membrane	75/939	359/18678	1.39E-26	6.26E-24	4.76E-24	100/290/301/9914/4059/146206/929/9332/930/914/30835/940/948/916/921/969/972/973/925/999/1493/8029/3627/4283/1917/2152/2208/2213/2214/2219/2350/2811/3055/3105/3106/3107/3113/3115/3118/3119/3120/3122/3123/3125/3134/3136/3383/29851/9173/3559/3560/3561/3575/3675/3684/3689/3902/10859/120892/931/4897/5027/26191/5778/5788/22821/5996/5997/6403/55576/6769/342667/7054/51311/7412	75
CC	GO:0009897	external side of plasma membrane	48/939	199/18678	3.73E-20	8.39E-18	6.38E-18	100/290/301/4059/929/9332/930/914/30835/940/948/916/921/969/972/973/925/1493/8029/3627/4283/2152/2208/2213/2214/2219/2350/2811/3383/29851/9173/3559/3560/3561/3575/3675/3684/3689/3902/10859/931/4897/5027/5788/6403/55576/51311/7412	48
CC	GO:0042611	MHC protein complex	17/939	25/18678	5.38	8.07	6.13E-15	972/3105/3106/3107/3108/3109/3112/3113/3115/3118/3119/3120/3122/3123/3125/3134/3136	17

© 2020 Yu Y et al. *JAMA Network Open.*

					E-17	E-15			
CC	GO:0030667	secretory granule membrane	53/939	293/18678	3.06E-16	3.01E-14	2.29E-14	21/84658/222487/290/10396/719/728/135228/929/948/952/960/963/160364/1378/3577/1536/8560/2171/2204/2207/2212/2215/2357/2358/11010/3136/10788/3683/3684/3687/3689/3903/27074/10288/3071/150372/4973/51365/5329/5788/5874/54509/6402/6403/140885/10326/6556/6515/6518/6622/7097/7305	53
CC	GO:0098797	plasma membrane protein complex	68/939	451/18678	3.34E-16	3.01E-14	2.29E-14	54518/730/777/919/915/916/917/923/972/973/925/999/1134/1439/1536/355/2707/127534/2709/375519/2903/2904/3105/3106/3107/3108/3109/3112/3113/3115/3118/3119/3120/3122/3123/3125/3134/3136/3359/3594/3559/3560/3569/3675/3676/3680/3683/3684/3687/3689/3696/3775/56479/3856/653361/4688/4689/9722/9180/5142/5979/6331/22941/7096/7097/50852/7412/7535	68
CC	GO:0043235	receptor complex	49/939	292/18678	8.42E-14	6.31E-12	4.80E-12	84059/150/54518/929/919/948/915/916/917/960/923/972/973/925/1134/1380/1439/83416/2903/2904/3359/3594/3559/3560/3569/3675/3676/3680/3683/3684/3687/3689/3691/3696/3822/3977/23643/4306/4973/9180/5979/22941/7096/7097/10333/51284/50852/7253/7535	49
CC	GO:0042613	MHC class II protein complex	12/939	16/18678	3.68E-13	2.36E-11	1.80E-11	972/3108/3109/3112/3113/3115/3118/3119/3120/3122/3123/3125	12
CC	GO:0030139	endocytic vesicle	47/939	289/18678	8.84E-13	4.97E-11	3.78E-11	306/348/9332/948/972/57214/11151/1493/8029/1536/1803/2209/752/10578/1839/3105/3106/3107/3113/3115/3118/3119/3120/3122/3123/3125/3134/3136/4057/8685/219972/4481/653361/4688/4689/5880/6556/23166/55576/6812/6890/6891/7096/7097/10333/51284/7454	47
CC	GO:0030666	endocytic vesicle membrane	33/939	161/18678	3.79E-12	1.89E-10	1.44E-10	306/348/9332/948/972/11151/1536/2209/1839/3105/3106/3107/3113/3115/3118/3119/3120/3122/3123/3125/3134/3136/8685/4481/5880/6556/23166/55576/6890/6891/7096/7097/10333	33
CC	GO:0045121	membrane raft	47/939	309/18678	1.02E-	4.61E-	3.50E-10	5244/107/119/84830/330/695/929/930/914/948/962/973/925/999/1525/1803/355/2817/23426/3055/3162/3383/3684/3689/3738/3932/3937/4038/120892/54742/114569/931/9722/4973	47

© 2020 Yu Y et al. *JAMA Network Open.*

					11	10		/5577/5788/5979/6281/6331/6401/6404/6513/7035/7096/7097/10333/7535	
CC	GO:0098857	membrane microdomain	47/939	310/18678	1.15E-11	4.71E-10	3.58E-10	5244/107/119/84830/330/695/929/930/914/948/962/973/925/999/1525/1803/355/2817/23426/3055/3162/3383/3684/3689/3738/3932/3937/4038/120892/54742/114569/931/9722/4973/5577/5788/5979/6281/6331/6401/6404/6513/7035/7096/7097/10333/7535	47
CC	GO:0071556	integral component of lumenal side of endoplasmic reticulum membrane	14/939	29/18678	2.29E-11	7.50E-10	5.70E-10	972/3105/3106/3107/3113/3115/3118/3119/3120/3122/3123/3125/3134/3136	14
CC	GO:0098553	lumenal side of endoplasmic reticulum membrane	14/939	29/18678	2.29E-11	7.50E-10	5.70E-10	972/3105/3106/3107/3113/3115/3118/3119/3120/3122/3123/3125/3134/3136	14
CC	GO:0098802	plasma membrane receptor complex	31/939	153/18678	2.33E-11	7.50E-10	5.70E-10	54518/919/915/916/917/923/973/925/1134/1439/2903/2904/3359/3594/3559/3560/3569/3675/3676/3680/3683/3684/3687/3689/3696/9180/22941/7096/7097/50852/7535	31
CC	GO:0098589	membrane region	47/939	321/18678	3.97E-11	1.19E-09	9.06E-10	5244/107/119/84830/330/695/929/930/914/948/962/973/925/999/1525/1803/355/2817/23426/3055/3162/3383/3684/3689/3738/3932/3937/4038/120892/54742/114569/931/9722/4973/5577/5788/5979/6281/6331/6401/6404/6513/7035/7096/7097/10333/7535	47
CC	GO:0031012	extracellular matrix	57/939	468/18678	5.53E-10	1.55E-08	1.18E-08	8728/27299/56999/54507/249/301/309/653145/348/4059/655/712/713/714/147372/50937/1116/1310/340267/1285/1286/131873/1297/1476/1514/84034/2152/2201/2219/10875/2817/3383/3897/284217/3909/3914/4321/4316/4318/22915/4583/89797/9423/5266/5806/5649/6281/6279/5265/5272/710/9806/7980/7052/4982/7399/7422	57
CC	GO:0001772	immunological synapse	14/939	36/18678	8.06	2.13	1.62E-08	940/951/916/963/923/11151/3001/3002/84868/3383/3932/5588/388325/7535	14

© 2020 Yu Y et al. *JAMA Network Open.*

					E-10	E-08			
CC	GO:0045335	phagocytic vesicle	26/939	128/18678	8.96E-10	2.24E-08	1.70E-08	306/948/11151/1536/752/10578/3105/3106/3107/3134/3136/4057/219972/653361/4688/4689/5880/6556/6812/6890/6891/7096/7097/10333/51284/7454	26
CC	GO:0042101	T cell receptor complex	9/939	17/18678	3.33E-08	7.89E-07	6.00E-07	54518/919/915/916/917/923/925/50852/7535	9
CC	GO:0070820	tertiary granule	27/939	164/18678	4.91E-08	1.10E-06	8.40E-07	84658/963/978/1118/160364/1378/1476/2919/1536/2204/2207/2357/2358/3684/3687/3689/3903/10288/4057/4069/4318/3071/4973/5806/140885/6556/6515	27
CC	GO:0012507	ER to Golgi transport vesicle membrane	15/939	55/18678	5.32E-08	1.14E-06	8.67E-07	374/972/3105/3106/3107/3113/3115/3118/3119/3120/3122/3123/3125/3134/3136	15
CC	GO:0042581	specific granule	26/939	160/18678	1.13E-07	2.32E-06	1.76E-06	222487/306/10396/719/948/963/1116/1118/160364/2919/1536/8560/1794/2204/2358/3683/3684/3689/3903/4057/4069/4973/5329/5806/6515/6518	26
CC	GO:0030134	COPII-coated ER to Golgi transport vesicle	16/939	69/18678	2.27E-07	4.45E-06	3.38E-06	374/972/3105/3106/3107/3113/3115/3118/3119/3120/3122/3123/3125/3134/3136/5265	16
CC	GO:0098636	protein complex involved in cell adhesion	11/939	33/18678	3.41E-07	6.31E-06	4.80E-06	940/941/1493/3675/3676/3680/3683/3684/3687/3689/3696	11
CC	GO:0062023	collagen-containing extracellular matrix	45/939	399/18678	3.50E-07	6.31E-06	4.80E-06	8728/27299/56999/54507/301/309/653145/348/4059/655/712/713/714/50937/1310/340267/1285/1286/131873/1297/1476/1514/84034/2152/2201/2219/10875/2817/3383/3897/284217/3909/3914/4318/22915/4583/89797/9423/6281/6279/526	45

© 2020 Yu Y et al. *JAMA Network Open.*

								5/5272/710/7052/7399	
CC	GO:0030669	clathrin-coated endocytic vesicle membrane	12/939	40/18678	3.68E-07	6.36E-06	4.84E-06	348/972/2209/1839/3113/3115/3118/3119/3120/3122/3123/3125	12
CC	GO:0030665	clathrin-coated vesicle membrane	20/939	110/18678	5.18E-07	8.40E-06	6.39E-06	348/374/915/917/972/57214/1601/2069/2209/1839/3113/3115/3118/3119/3120/3122/3123/3125/3575/9182	20
CC	GO:0035579	specific granule membrane	18/939	91/18678	5.23E-07	8.40E-06	6.39E-06	222487/10396/719/948/963/160364/1536/8560/2204/2358/3683/3684/3689/3903/4973/5329/6515/6518	18
CC	GO:0045334	clathrin-coated endocytic vesicle	14/939	59/18678	9.56E-07	1.48E-05	1.13E-05	348/972/57214/1493/2209/1839/3113/3115/3118/3119/3120/3122/3123/3125	14
CC	GO:0030662	coated vesicle membrane	25/939	169/18678	1.22E-06	1.83E-05	1.39E-05	348/374/915/917/972/57214/1601/2069/2209/1839/3105/3106/3107/3113/3115/3118/3119/3120/3122/3123/3125/3134/3136/3575/9182	25
CC	GO:0030136	clathrin-coated vesicle	26/939	182/18678	1.48E-06	2.14E-05	1.63E-05	5244/348/374/23245/915/917/972/57214/1493/1601/1906/2069/2209/1839/3113/3115/3118/3119/3120/3122/3123/3125/3575/5874/9182/85439	26
CC	GO:0070821	tertiary granule membrane	15/939	73/18678	2.78E-06	3.92E-05	2.98E-05	963/160364/1536/2204/2207/2358/3684/3687/3689/3903/10288/4973/140885/6556/6515	15
CC	GO:0030135	coated vesicle	32/939	261/18678	2.97E-06	4.05E-05	3.08E-05	5244/348/374/23245/915/917/972/57214/1493/1601/1906/2069/2209/1839/3105/3106/3107/3113/3115/3118/3119/3120/3122/3123/3125/3134/3136/3575/5874/9182/5265/85439	32
CC	GO:0030670	phagocytic vesicle membrane	15/939	74/18678	3.33E-	4.39E-	3.33E-05	306/11151/1536/3105/3106/3107/3134/3136/5880/6556/6890/6891/7096/7097/10333	15

					06	05			
CC	GO:0031225	anchored component of membrane	23/939	156/18678	3.41E-06	4.39E-05	3.33E-05	249/420/135228/929/914/962/1043/1368/1800/2215/2350/2674/2811/2817/54742/266727/10232/4907/5329/5874/56963/7035/8875	23
CC	GO:0044853	plasma membrane raft	18/939	106/18678	5.24E-06	6.55E-05	4.98E-05	119/84830/925/999/3055/3162/3684/3689/3937/4038/120892/931/9722/6331/6401/6404/6513/7035	18
CC	GO:0101003	ficolin-1-rich granule membrane	13/939	61/18678	8.36E-06	0.000101706	7.73E-05	84658/1378/2204/2207/2357/2358/3687/3689/10288/3071/140885/6556/6515	13
CC	GO:0030176	integral component of endoplasmic reticulum membrane	19/939	125/18678	1.52E-05	0.000180446	0.000137181	972/10170/6785/3105/3106/3107/3113/3115/3118/3119/3120/3122/3123/3125/3134/3136/54463/6890/6891	19
CC	GO:0031227	intrinsic component of endoplasmic reticulum membrane	19/939	131/18678	3.00E-05	0.000345621	0.000262752	972/10170/6785/3105/3106/3107/3113/3115/3118/3119/3120/3122/3123/3125/3134/3136/54463/6890/6891	19
CC	GO:0030660	Golgi-associated vesicle membrane	16/939	102/18678	4.76E-05	0.000535205	0.000406881	374/972/3105/3106/3107/3113/3115/3118/3119/3120/3122/3123/3125/3134/3136/9182	16
CC	GO:0101002	ficolin-1-rich granule	23/939	185/18678	5.71E-	0.0006	0.000476063	84658/51411/978/1378/1476/2204/2207/2219/10875/2357/2358/3101/3304/3687/3689/10288/4318/4332/3071/5265/140885/6556/6515	23

© 2020 Yu Y et al. *JAMA Network Open.*

					05	26206			
CC	GO:0042629	mast cell granule	7/939	22/18678	6.96E-05	0.000745471	0.000566733	301/695/8013/5293/5294/10125/5552	7
CC	GO:0008305	integrin complex	8/939	30/18678	8.61E-05	0.000901368	0.00068525	3675/3676/3680/3683/3684/3687/3689/3696	8
CC	GO:0005581	collagen trimer	14/939	86/18678	9.23E-05	0.000943879	0.000717569	712/713/714/147372/1310/340267/1285/1286/131873/1297/84034/2219/8685/4481	14
CC	GO:0005798	Golgi-associated vesicle	19/939	145/18678	0.000122133	0.001221327	0.000928495	374/972/3105/3106/3107/3113/3115/3118/3119/3120/3122/3123/3125/3134/3136/120892/5874/9182/5265	19
CC	GO:0005911	cell-cell junction	39/939	414/18678	0.000128214	0.00125427	0.000953539	5244/575/301/57863/914/916/963/999/11151/1525/1803/1824/1829/23136/83481/2707/127534/2709/375519/79977/3702/120425/3856/284217/3937/10397/5818/4868/5027/64065/11187/6331/57619/6513/387357/23650/7409/7454/7535	39
CC	GO:0005765	lysosomal membrane	34/939	344/18678	0.00013737	0.0001288	0.000979865	290/309/10396/719/972/8029/1601/1803/1917/2357/11010/59345/3108/3109/3112/3113/3115/3118/3119/3120/3122/3123/3125/3134/3136/10410/57535/27074/7805/4647/150372/4948/51284/51311	34

© 2020 Yu Y et al. *JAMA Network Open.*

					483	899			
CC	GO:0098852	lytic vacuole membrane	34/939	344/18678	0.000137483	0.001288899	0.000979865	290/309/10396/719/972/8029/1601/1803/1917/2357/11010/59345/3108/3109/3112/3113/3115/3118/3119/3120/3122/3123/3125/3134/3136/10410/57535/27074/7805/4647/150372/4948/51284/51311	34
CC	GO:0030658	transport vesicle membrane	22/939	188/18678	0.000199898	0.001835796	0.001395634	374/10396/972/3105/3106/3107/3113/3115/3118/3119/3120/3122/3123/3125/3134/3136/120892/79772/5874/9182/6622/91683	22
CC	GO:0060205	cytoplasmic vesicle lumen	33/939	338/18678	0.00021516	0.001936437	0.001472145	100/51816/348/23526/64333/51411/718/978/1116/1118/1476/1521/2919/1794/2171/2219/83706/10578/3101/3481/4057/4069/22915/4332/11240/51316/5806/6279/6414/5265/710/5552/7422	33
CC	GO:0031983	vesicle lumen	33/939	339/18678	0.000227194	0.002004656	0.001524007	100/51816/348/23526/64333/51411/718/978/1116/1118/1476/1521/2919/1794/2171/2219/83706/10578/3101/3481/4057/4069/22915/4332/11240/51316/5806/6279/6414/5265/710/5552/7422	33
CC	GO:0031252	cell leading edge	36/939	384/18678	0.000252074	0.002181411	0.001658383	30817/84059/199/54518/23526/660/146206/960/999/1021/23242/11151/7852/1803/23136/55612/54857/10788/3691/3936/4210/64005/5341/26499/55607/5800/117177/5880/10235/9750/64092/388325/80725/10381/7399/7456	36
CC	GO:0016324	apical plasma membrane	30/939	300/18678	0.00027202	0.00230966	0.001755888	5243/5244/10149/301/948/960/53405/8029/1800/1803/1829/3775/114569/4478/23255/4647/9180/5031/5800/5874/9750/22941/57619/6559/6513/6518/159963/23043/140803/7399	30

© 2020 Yu Y et al. *JAMA Network Open.*

					8	9			
CC	GO:0001891	phagocytic cup	7/939	27/18678	0.000290607	0.002421723	0.001841076	575/199/301/51411/11151/3936/64005	7
CC	GO:0010008	endosome membrane	41/939	464/18678	0.000339544	0.002778091	0.002111999	9744/301/309/653145/8938/929/911/29126/8029/2209/10457/3105/3106/3107/3108/3109/3112/3113/3115/3118/3119/3120/3122/3123/3125/3134/3136/10410/57535/23643/4360/4689/10397/27065/4948/5874/5979/6556/79689/51284/51311	41
CC	GO:0005774	vacuolar membrane	35/939	382/18678	0.0004674	0.003755894	0.002855358	290/309/10396/719/972/8029/1601/1803/1917/2357/11010/59345/3108/3109/3112/3113/3115/3118/3119/3120/3122/3123/3125/3134/3136/10410/57535/27074/7805/4647/150372/4948/80183/51284/51311	35
CC	GO:0032994	protein-lipid complex	8/939	39/18678	0.000605905	0.004783457	0.003636546	341/348/274/1071/4018/4023/4481/7941	8
CC	GO:0030133	transport vesicle	32/939	349/18678	0.00079266	0.006149948	0.004675399	321/374/10396/972/1906/2261/2903/3055/3105/3106/3107/3113/3115/3118/3119/3120/3122/3123/3125/3134/3136/90865/120892/79772/5142/5874/9182/6622/9627/85439/91683/7054	32
CC	GO:0043025	neuronal cell body	39/939	454/18678	0.000807353	0.00615778	0.004681353	100/199/306/348/23245/720/729230/1233/933/22866/23242/1536/1917/2048/3359/3676/3775/3897/4038/120892/653361/10439/5027/10846/10891/55607/5577/5979/22941/6507/6515/9498/6622/9627/80725/7054/64699/7345/7399	39

© 2020 Yu Y et al. *JAMA Network Open.*

CC	GO:0034774	secretory granule lumen	30/939	321/18678	0.000839409	0.006214564	0.004724522	51816/23526/64333/51411/718/978/1116/1118/1476/1521/2919/1794/2171/2219/83706/3101/3481/4057/4069/22915/4332/11240/51316/5806/6279/6414/5265/710/5552/7422	30
CC	GO:0045177	apical part of cell	33/939	365/18678	0.000842419	0.006214564	0.004724522	5243/5244/10149/301/728/948/960/10752/53405/8029/1800/1803/1829/3775/114569/4478/23255/4647/9180/5031/5800/5874/9750/22941/57619/6559/6513/6518/159963/23043/140803/7399/7412	33
CC	GO:0098562	cytoplasmic side of membrane	16/939	138/18678	0.001558779	0.011313721	0.008601075	9914/146206/914/999/1917/3055/120892/26191/5778/5788/22821/5996/5997/6769/342667/7054	16
CC	GO:0009898	cytoplasmic side of plasma membrane	14/939	114/18678	0.001733586	0.012382761	0.009413795	9914/146206/914/999/3055/26191/5778/5788/22821/5996/5997/6769/342667/7054	14
CC	GO:0031256	leading edge membrane	17/939	153/18678	0.001802259	0.01267213	0.009633783	30817/84059/199/23526/660/960/1803/23136/55612/3936/64005/5341/26499/10235/9750/388325/7399	17
CC	GO:0031091	platelet alpha granule	12/939	91/18678	0.001969886	0.013637674	0.010367823	135228/948/83706/3481/22915/6403/5265/710/6622/5552/6812/7422	12
C	GO:00	plasma	7/939	37/18678	0.	0.	0.011099519	341/348/1071/4018/4023/4481/7941	7

© 2020 Yu Y et al. *JAMA Network Open.*

C	34358	lipoprotein particle			0021737798	0146001137			
CC	GO:1990777	lipoprotein particle	7/939	37/18678	0.0021737798	0.0146001137	0.011099519	341/348/1071/4018/4023/4481/7941	7
CC	GO:0001931	uropod	4/939	13/18678	0.0031502988	0.0205454119	0.015619324	4478/8395/388325/6404	4
CC	GO:0031254	cell trailing edge	4/939	13/18678	0.0031502988	0.0205454119	0.015619324	4478/8395/388325/6404	4
CC	GO:0032588	trans-Golgi network membrane	11/939	85/18678	0.0034660733	0.0222818988	0.016939454	8938/972/3113/3115/3118/3119/3120/3122/3123/3125/27065	11
CC	GO:0005884	actin filament	10/939	74/18678	0.0038024144	0.0240998088	0.018321491	199/301/23242/11151/342184/54857/3055/3936/5880/7454	10
CC	GO:0005767	secondary lysosome	4/939	14/18678	0.00	0.02	0.020129804	120892/653361/4688/4689	4

© 2020 Yu Y et al. *JAMA Network Open.*

					42365 49	64784 34			
CC	GO:0055038	recycling endosome membrane	10/939	76/18678	0.00461796 8	0.02846692 6	0.021641522	9744/8938/29126/3105/3106/3107/3134/3136/10397/27065	10
CC	GO:0019898	extrinsic component of membrane	19/939	199/18678	0.00558745 8	0.03397778 7	0.025831066	301/146206/999/22866/8029/2219/2674/3055/94025/653361/5293/5294/23533/5329/5996/9628/6281/6769/342667	19
CC	GO:0098858	actin-based cell projection	19/939	200/18678	0.00589237 8	0.03499139 2	0.026601643	84059/768/960/1525/1800/10788/3675/3936/120892/4354/4478/64005/4647/55607/9750/80725/10381/7399/7412	19
CC	GO:1904724	tertiary granule lumen	8/939	55/18678	0.00590965 7	0.03499139 2	0.026601643	978/1118/1476/2919/4057/4069/4318/5806	8
CC	GO:0005901	caveola	10/939	79/18678	0.00609227 9	0.03560423	0.027067543	84830/999/3055/3162/120892/9722/6331/6401/6513/7035	10
CC	GO:0071437	invadopodium	4/939	16/18678	0.0071	0.0410	0.031180298	1803/3675/4478/5329	4

© 2020 Yu Y et al. *JAMA Network Open.*

					09108	14084			
CC	GO:0030027	lamellipodium	18/939	191/18678	0.0078352910	0.0446314030	0.033930306	199/54518/146206/960/999/11151/1803/54857/10788/4210/64005/26499/55607/5800/117177/5880/80725/10381	18
CC	GO:0016328	lateral plasma membrane	8/939	58/18678	0.0081555640	0.0458754730	0.03487609	301/999/1829/23255/27065/5800/6331/375611	8
CC	GO:0031092	platelet alpha granule membrane	4/939	17/18678	0.0089321110	0.0490176820	0.037264905	135228/948/6403/6622	4
CC	GO:0042599	lamellar body	4/939	17/18678	0.0089321110	0.0490176820	0.037264905	21/5650/27074/11005	4
MF	GO:0004896	cytokine receptor activity	24/897	78/16969	8.65E-13	6.22E-10	5.12E-10	729230/1233/1236/960/972/1439/3577/7852/10663/2322/2674/3594/3595/7850/3556/9173/50615/3559/3560/3561/133396/3575/3977/9180	24
MF	GO:0042277	peptide binding	36/897	215/16969	6.91E-10	2.15E-07	1.77E-07	348/712/929/911/30835/948/972/57214/10332/1396/2048/2213/2358/2903/2904/3105/3106/3107/3113/3115/3119/3122/3123/3125/3134/3684/3689/10288/8685/4481/4804/64127/6890/7097/10333/54209	36
M	GO:00	glycosamino	34/89	199/1696	1.	2.	1.77E-07	51816/30817/348/655/960/57214/3627/6373/10563/10457/1	3

© 2020 Yu Y et al. *JAMA Network Open.*

F	05539	glycan binding	7	9	19 E-09	15 E-07		45864/1839/3512/4018/4023/4057/4316/89797/114548/64127/26577/26279/50512/5788/83998/6402/6403/9806/50859/23166/55576/7097/54209/7422	4
MF	GO:0042605	peptide antigen binding	13/897	30/16969	1.20E-09	2.15E-07	1.77E-07	30835/10332/3105/3106/3107/3113/3115/3119/3122/3123/3125/3134/6890	13
MF	GO:0019865	immunoglobulin binding	11/897	23/16969	6.38E-09	9.17E-07	7.55E-07	933/2204/2208/2209/2212/2213/9103/2214/2215/3512/931	11
MF	GO:0001848	complement binding	10/897	20/16969	1.85E-08	1.72E-06	1.41E-06	720/721/728/629/1378/1380/3684/3689/5806/11326	10
MF	GO:0023023	MHC protein complex binding	11/897	25/16969	1.91E-08	1.72E-06	1.41E-06	972/925/3108/3109/3112/3122/3123/3822/10859/10288/931	11
MF	GO:0001540	amyloid-beta binding	18/897	71/16969	1.91E-08	1.72E-06	1.41E-06	348/712/948/972/2048/2213/2358/2903/2904/3684/3689/10288/8685/4481/4804/7097/10333/54209	18
MF	GO:0005125	cytokine activity	27/897	154/16969	3.46E-08	2.76E-06	2.27E-06	374/6357/6363/6366/6348/6351/6352/3627/6373/10563/3576/4283/1906/3603/3553/11009/9235/90865/3569/4050/3084/6398/6696/4982/8600/10673/7422	27
MF	GO:0030246	carbohydrate binding	36/897	252/16969	5.18E-08	3.72E-06	3.06E-06	721/30835/933/969/1116/1117/10462/160364/388512/9976/10332/64581/112399/5168/2208/2219/11226/3099/3101/3820/3822/4360/5818/4973/5806/83998/6401/6402/6403/6614/89790/89858/946/6515/6518/56241	36
MF	GO:0033218	amide binding	37/897	266/16969	6.85E-08	4.22E-06	3.47E-06	348/712/929/911/30835/948/972/57214/10332/1396/2048/2213/2350/2358/2903/2904/3105/3106/3107/3113/3115/3119/3122/3123/3125/3134/3684/3689/10288/8685/4481/4804/64127/6890/7097/10333/54209	37
M	GO:00	cargo	19/89	85/16969	7.	4.	3.47E-06	9332/948/921/923/3426/8029/1601/5168/2350/2358/3684/36	1

F	38024	receptor activity	7		04 E-08	22 E-06		89/8685/4360/4481/23166/55576/56241/64699	9
M F	GO:00 42379	chemokine receptor binding	14/89 7	47/16969	8. 01 E-08	4. 43 E-06	3.64E-06	6357/6363/6347/6364/6366/6348/6351/6352/729230/3627/6 373/10563/3576/4283	1 4
M F	GO:00 32395	MHC class II receptor activity	7/897	10/16969	1. 18 E-07	6. 04 E-06	4.97E-06	3112/3113/3118/3119/3120/3122/3125	7
M F	GO:00 01618	virus receptor activity	17/89 7	74/16969	2. 24 E-07	9. 55 E-06	7.86E-06	290/30835/941/942/10332/1378/1380/1525/7852/1803/2838/ 3304/3383/4360/5818/6404/6504	1 7
M F	GO:01 04005	hijacked molecular function	17/89 7	74/16969	2. 24 E-07	9. 55 E-06	7.86E-06	290/30835/941/942/10332/1378/1380/1525/7852/1803/2838/ 3304/3383/4360/5818/6404/6504	1 7
M F	GO:00 19955	cytokine binding	20/89 7	100/1696 9	2. 26 E-07	9. 55 E-06	7.86E-06	2532/729230/1236/135228/948/972/1436/1441/3577/7852/1 0663/2633/3587/3594/7850/3560/3561/133396/3676/7538	2 0
M F	GO:00 15026	coreceptor activity	12/89 7	40/16969	6. 25 E-07	2. 50 E-05	2.06E-05	940/941/942/925/7852/10663/115350/2838/3676/23643/581 8/4804	1 2
M F	GO:00 03823	antigen binding	24/89 7	150/1696 9	1. 12 E-06	4. 23 E-05	3.48E-05	28/911/30835/962/10332/3105/3106/3107/3113/3115/3119/3 122/3123/3125/3134/3575/3676/3512/3822/3902/11006/140 885/6504/6890	2 4
M F	GO:00 08009	chemokine activity	10/89 7	30/16969	1. 85 E-06	6. 65 E-05	5.47E-05	6357/6363/6366/6348/6352/3627/6373/10563/3576/4283	1 0
M	GO:00	IgG binding	6/897	10/16969	3.	0.	0.000100884	2209/2212/2213/9103/2214/2215	6

© 2020 Yu Y et al. *JAMA Network Open.*

F	19864				75 E-06	00 01 22 61 3			
MF	GO:0035325	Toll-like receptor binding	6/897	10/16969	3.75 E-06	0.00 01 22 61 3	0.000100884	948/23643/6279/7096/7097/10333	6
MF	GO:0048020	CCR chemokine receptor binding	9/897	26/16969	4.28 E-06	0.00 01 33 79 5	0.000110084	6363/6347/6364/6366/6348/6351/6352/729230/10563	9
MF	GO:0023026	MHC class II protein complex binding	7/897	16/16969	8.46 E-06	0.00 02 53 56 4	0.000208627	972/3108/3109/3112/3122/3123/931	7
MF	GO:0005044	scavenger receptor activity	12/897	51/16969	1.04 E-05	0.00 02 98 46 2	0.000245568	9332/948/921/923/3426/5168/8685/4481/23166/55576/56241/64699	12
MF	GO:0008329	signaling pattern recognition receptor activity	7/897	17/16969	1.37 E-05	0.00 03 78 90 9	0.000311759	929/948/64581/2219/23643/8685/7097	7
MF	GO:0019956	chemokine binding	8/897	23/16969	1.42	0.00	0.000311759	2532/729230/1236/3577/7852/10663/3676/7538	8

© 2020 Yu Y et al. *JAMA Network Open.*

					E-05	0378909			
MF	GO:0001530	lipopolysaccharide binding	9/897	30/16969	1.62E-05	0.000415612	0.000341957	575/929/923/4057/23643/5027/6403/7097/54209	9
MF	GO:0038187	pattern recognition receptor activity	7/897	18/16969	2.14E-05	0.000531742	0.000437507	929/948/64581/2219/23643/8685/7097	7
MF	GO:0030414	peptidase inhibitor activity	22/897	157/16969	2.72E-05	0.000651874	0.000536349	718/720/721/135228/939/340267/1285/1476/1800/4018/4057/89932/51050/5266/5265/5272/710/6622/11005/9806/50859/7035	22
MF	GO:0001664	G protein-coupled receptor binding	26/897	205/16969	3.15E-05	0.000729481	0.000600203	51816/150/8862/718/6357/6363/6347/6364/6366/6348/6351/6352/729230/3627/6373/10563/3576/4283/1906/2219/2357/51704/3304/3691/8685/7345	26
MF	GO:0004866	endopeptidase inhibitor activity	21/897	149/16969	3.77E-05	0.000823756	0.00067777	718/720/721/135228/939/340267/1285/1476/1800/4018/4057/89932/5266/5265/5272/710/6622/11005/9806/50859/7035	21
MF	GO:0098632	cell-cell adhesion mediator	9/897	33/16969	3.78E-	0.0008	0.00067777	301/1525/1824/1829/4897/11187/5365/140885/23650	9

© 2020 Yu Y et al. *JAMA Network Open.*

		activity			05	23756			
MF	GO:0098631	cell adhesion mediator activity	10/897	42/16969	5.08E-05	0.001010736 42	0.000883372	301/4059/1525/1824/1829/4897/11187/5365/140885/23650	10
MF	GO:0031406	carboxylic acid binding	23/897	176/16969	5.54E-05	0.001137956	0.000936288	241/341/933/960/57214/56603/112399/2171/2172/2219/2350/2729/2904/145864/5730/6279/6401/6403/6507/23166/55576/6999/7054	23
MF	GO:0061135	endopeptidase regulator activity	21/897	154/16969	6.17E-05	0.001233077	0.001014551	718/720/721/135228/939/340267/1285/1476/1800/4018/4057/89932/5266/5265/5272/710/6622/11005/9806/50859/7035	21
MF	GO:0043177	organic acid binding	23/897	178/16969	6.61E-05	0.001285302	0.001057521	241/341/933/960/57214/56603/112399/2171/2172/2219/2350/2729/2904/145864/5730/6279/6401/6403/6507/23166/55576/6999/7054	23
MF	GO:0001846	opsonin binding	6/897	15/16969	7.11E-05	0.001345592	0.001107126	720/1378/3684/3689/5806/11326	6
MF	GO:0061134	peptidase regulator activity	24/897	192/16969	7.90E-05	0.001456	0.001198172	718/720/721/135228/939/340267/1285/1476/1800/4018/4057/22861/89932/26577/51050/5266/5265/5272/710/6622/11005/9806/50859/7035	24

© 2020 Yu Y et al. *JAMA Network Open.*

						248			
MF	GO:0045236	CXCR chemokine receptor binding	5/897	10/16969	8.23E-05	0.001479489	0.001217294	3627/6373/10563/3576/4283	5
MF	GO:0008201	heparin binding	20/897	148/16969	0.000104075	0.001825127	0.001501678	51816/348/655/3627/6373/10563/10457/1839/4018/4023/4057/4316/89797/26577/26279/5788/83998/6402/6403/7422	20
MF	GO:0004497	monooxygenase activity	14/897	86/16969	0.000155808	0.00266729	0.002194593	9023/1589/56603/1593/1562/1557/1573/29785/1577/2327/2328/8564/6916/7054	14
MF	GO:0048018	receptor ligand activity	38/897	397/16969	0.000300088	0.005030999	0.004139406	51816/8862/374/655/6357/6363/6366/6348/6351/6352/2919/3627/6373/10563/3576/4283/1906/10457/1839/3481/3603/3553/11009/9235/90865/3569/4050/3084/145957/6398/10371/6696/7031/4982/8600/10673/1890/7422	38
MF	GO:0016705	oxidoreductase activity, acting on paired donors, with incorporation or reduction of molecular oxygen	18/897	137/16969	0.000322902	0.005276516	0.004341413	9023/1589/56603/1593/1562/1573/29785/1577/8560/112399/2327/2328/3162/8564/5742/79966/6916/7054	18
M	GO:00	protein	5/897	13/16969	0.	0.	0.004836206	1525/1824/1829/4897/140885	5

© 2020 Yu Y et al. *JAMA Network Open.*

F	86080	binding involved in heterotypic cell-cell adhesion			00036787 9	00587788 3			
MF	GO:0042834	peptidoglycan binding	5/897	14/16969	0.00054744 2	0.00855675 1	0.007040325	3512/114548/64127/7097/54209	5
MF	GO:0019838	growth factor binding	16/897	121/16969	0.00063308 7	0.00968488 6	0.007968532	135228/948/10563/1843/2261/23105/2817/3587/7850/3560/3561/3691/3977/4804/9180/6331	16
MF	GO:0042287	MHC protein binding	8/897	38/16969	0.00070070 1	0.01049592 3	0.008635837	972/925/64581/3902/10859/10288/6890/6891	8
MF	GO:0005126	cytokine receptor binding	23/897	210/16969	0.00075713 7	0.01107548 2	0.009112687	6357/6363/6347/6364/6366/6348/6351/6352/729230/3627/6373/10563/3576/4283/3594/3553/3569/3977/4050/26228/8600/10673/7422	23
MF	GO:0004222	metalloendopeptidase activity	13/897	89/16969	0.00077770 8	0.01107548 2	0.009112687	8728/8745/10863/27299/56999/9427/3792/4321/4316/4318/5069/5251/129293	13
MF	GO:0030169	low-density lipoprotein	5/897	15/16969	0.00	0.01	0.009112687	948/4481/23166/55576/54209	5

		particle binding			07856 04	10754 82			
MF	GO:1990782	protein tyrosine kinase binding	12/897	80/16969	0.000963406	0.011332093 5	0.010960201	389658/55024/914/919/115352/3687/4038/3084/140885/26228/50852/54209	12
MF	GO:0030545	receptor regulator activity	38/897	425/16969	0.001102384	0.0149549 78	0.01230466	51816/8862/374/655/6357/6363/6366/6348/6351/6352/2919/3627/6373/10563/3576/4283/1906/10457/1839/3481/3603/3553/11009/9235/90865/3569/4050/3084/145957/6398/10371/6696/7031/4982/8600/10673/1890/7422	38
MF	GO:0048029	monosaccharide binding	10/897	62/16969	0.001425526	0.018980621	0.015616879	30835/388512/10332/112399/3099/3101/4360/6403/6515/6518	10
MF	GO:0001637	G protein-coupled chemoattractant receptor activity	6/897	25/16969	0.001597539	0.02051126	0.016876258	729230/1233/1236/3577/7852/10663	6
MF	GO:0004950	chemokine receptor activity	6/897	25/16969	0.001597539	0.02051126	0.016876258	729230/1233/1236/3577/7852/10663	6
MF	GO:0004713	protein tyrosine kinase	15/897	120/16969	0.0016	0.0207	0.017099107	640/660/695/1436/2048/2261/2322/3055/3702/3718/3932/5979/6098/7273/7535	15

© 2020 Yu Y et al. *JAMA Network Open.*

		activity			47539	82109			
MF	GO:0071813	lipoprotein particle binding	7/897	34/16969	0.00172814 5	0.02105993 1	0.017327694	348/948/4023/4481/23166/55576/54209	7
MF	GO:0071814	protein-lipid complex binding	7/897	34/16969	0.00172814 5	0.02105993 1	0.017327694	348/948/4023/4481/23166/55576/54209	7
MF	GO:0004175	endopeptidase activity	30/897	322/16969	0.00189787 4	0.0227428 6	0.018712374	8728/8745/10863/27299/56999/717/629/3426/1514/1803/9427/2152/3001/3002/3003/3792/5653/5650/4018/4057/4321/4316/4318/5069/5251/5696/5698/64699/129293/7345	30
MF	GO:0043394	proteoglycan binding	7/897	36/16969	0.0024462 2	0.02883331 9	0.023723483	51816/348/1514/10457/4023/26279/5788	7
MF	GO:0005178	integrin binding	14/897	113/16969	0.00252306 4	0.02910159 5	0.023944216	8745/1285/1525/2208/83706/3383/3481/3553/3675/120425/3936/3084/6696/7412	14
MF	GO:0008395	steroid hydroxylase activity	5/897	19/16969	0.00252549	0.02910101	0.023944216	9023/1589/1593/1557/1577	5

© 2020 Yu Y et al. *JAMA Network Open.*

					931	595			
MF	GO:0033691	sialic acid binding	4/897	12/16969	0.002728292	0.030650657	0.025218753	933/2219/6401/6403	4
MF	GO:0001784	phosphotyrosine residue binding	7/897	37/16969	0.002881742	0.031876501	0.026227353	3055/3932/64092/9021/26228/7409/7535	7
MF	GO:0008237	metallopeptidase activity	17/897	153/16969	0.0030218 4	0.032919742	0.027085711	8728/8745/10863/27299/56999/290/51200/1800/9427/64167/3792/4321/4316/4318/5069/5251/129293	17
MF	GO:0005540	hyaluronic acid binding	5/897	20/16969	0.003253727	0.034745753	0.028588117	960/57214/145864/23166/55576	5
MF	GO:1901681	sulfur compound binding	22/897	221/16969	0.003286107	0.034745753	0.028588117	51816/30817/348/655/51363/3627/6373/10563/10457/1839/4018/4023/4057/4316/89797/26577/26279/5788/83998/6402/6403/7422	22
MF	GO:0003924	GTPase activity	22/897	224/16969	0.00386771	0.04030266	0.033160233	122622/2633/115361/115362/168537/120892/389643/5874/51655/158158/387496/5996/26575/5997/9628/54509/6236/58528/7280/347733/10381/84617	22

© 2020 Yu Y et al. *JAMA Network Open.*

						4			
MF	GO:0005506	iron ion binding	16/897	144/16969	0.003946528	0.040536483	0.033352615	54/316/9023/1589/56603/1593/1562/1557/1573/29785/1577/112399/4057/6622/6916/7054	16
MF	GO:0051861	glycolipid binding	5/897	21/16969	0.004087172	0.041389809	0.034054715	911/1800/284217/6402/6403	5
MF	GO:0004252	serine-type endopeptidase activity	15/897	133/16969	0.0044891 5	0.044543533	0.036649536	717/629/3426/1803/2152/3001/3002/3003/5653/5650/4018/4057/4316/4318/64699	15
MF	GO:0005518	collagen binding	9/897	61/16969	0.004522501	0.044543533	0.036649536	147372/960/1514/3675/3680/4321/4318/26577/7399	9
MF	GO:0002020	protease binding	14/897	121/16969	0.004726401	0.045922732	0.037784314	54507/274/147372/940/10752/1476/1803/2152/3675/6402/5265/5272/7128/7273	14
MF	GO:0030159	receptor signaling complex scaffold activity	5/897	22/16969	0.005062534	0.04750033	0.03908233	916/917/23426/120892/22941	5

© 2020 Yu Y et al. *JAMA Network Open.*

MF	GO:0043274	phospholipase binding	4/897	14/16969	0.005069334	0.04750033	0.03908233	6401/6622/6812/7454	4
MF	GO:0017124	SH3 domain binding	14/897	122/16969	0.005086962	0.04750033	0.03908233	8728/916/9829/9844/342184/3059/10656/653361/5063/26191/22941/140885/7454/7456	14

© 2020 Yu Y et al. *JAMA Network Open.*

eTable 12. Gene Ontology Pathway Enrichment Analysis of Immune-Desert Class in IMvigor210 Trial

Ontology	ID	Description	Gene ratio	Bg ratio	*P* value	*P* adjust	Q value	Gene ID	Count
BP	GO:0030198	extracellular matrix organization	75/408	334/17913	3.95E-53	1.49E-49	1.09E-49	8038/140766/9509/9507/165/84168/633/151887/1300/1301/1303/1307/1277/1278/1281/1289/1290/50509/1291/1292/1293/1295/1296/1311/90993/83716/1513/1545/1634/4921/11117/2191/2199/2200/23768/2331/2335/2303/2621/26585/3037/5654/22801/3678/3690/4015/4016/4017/4060/4148/4237/8076/4320/4327/4313/4314/22795/255743/5156/10630/90102/7837/5054/6423/6678/23213/55959/7042/7057/7076/7077/7092/3371/1462/7490	75
BP	GO:0043062	extracellular structure organization	77/408	387/17913	2.12E-50	4.00E-47	2.92E-47	8038/140766/9509/9507/165/185/84168/633/151887/1300/1301/1303/1307/1277/1278/1281/1289/1290/50509/1291/1292/1293/1295/1296/1311/90993/83716/1513/1545/1634/4921/11117/2191/2199/2200/23768/2331/2335/2303/2621/26585/3037/5654/22801/3678/3690/4015/4016/4017/4060/4148/4237/8076/4320/4327/4313/4314/22795/255743/5156/10630/90102/5360/7837/5054/6423/6678/23213/55959/7042/7057/7076/7077/7092/3371/1462/7490	77
BP	GO:0030199	collagen fibril organization	24/408	45/17913	4.72E-28	5.94E-25	4.34E-25	140766/9509/165/1301/1303/1277/1278/1281/1289/1290/50509/1311/1545/4921/11117/2331/2303/26585/4015/4017/4060/4320/6423/7042	24
BP	GO:0001501	skeletal system development	58/408	479/17913	7.19E-26	6.79E-23	4.96E-23	81792/9507/56172/8313/658/1009/1300/1301/1277/1278/1281/1311/1513/1893/2202/2121/2138/56975/2200/2260/2303/2702/2736/2737/26585/9464/3037/4015/4017/10234/4060/4148/4313/4883/9060/5125/56034/5156/91461/5549/5396/5745/5744/4920/6423/6424/6591/6678/6781/23213/55959/7042/7043/7045/7076/7092/7291/64131	58
BP	GO:0001503	ossification	49/408	388/17913	9.62E-23	7.26E-20	5.31E-20	56172/54829/8313/658/1009/1301/1277/1278/1290/1291/1311/90993/115908/1513/4921/1893/5167/56975/2303/2736/2737/26585/9464/3082/3488/22801/4015/4313/4330/9902/255743/4958/91461/5587/5743/5745/5744/4920/6423/6591/6678/6781/7042/7043/3371/63923/7291/1462/64131	49
B	G	cell-substrate adhesion	40	32	1.8	1.1	8.4	88/81792/84168/558/151887/1012/1307/1277/1281/50509/1295/1842/10085/11117/20	4

© 2020 Yu Y et al. *JAMA Network Open*.

P	O:0031589		/408	2/17913	4E-18	6E-15	6E-16	42/10979/2316/2335/2621/26585/3037/22801/3678/3690/3956/91663/22795/255743/10630/90102/5328/10631/8499/5054/8910/25992/6695/7057/7070/63923	0
BP	GO:0061448	connective tissue development	35/408	243/17913	2.40E-18	1.30E-15	9.47E-16	59/81792/8313/658/1301/1277/1289/1311/1513/1893/2202/2121/2260/2303/2736/2737/26585/9464/4015/4017/4060/5159/91461/5396/5745/5744/4920/6423/6591/6781/23213/55959/7045/7076/7490	35
BP	GO:0051216	cartilage development	29/408	182/17913	1.34E-16	6.33E-14	4.63E-14	81792/8313/658/1301/1277/1311/1513/1893/2202/2121/2260/2736/2737/26585/9464/4017/4060/91461/5396/5745/5744/4920/6423/6591/6781/23213/55959/7045/7076	29
BP	GO:0002062	chondrocyte differentiation	20/408	97/17913	4.85E-14	2.04E-11	1.49E-11	81792/8313/658/1301/1311/1893/2202/2260/2736/2737/26585/4017/91461/5745/5744/6423/6591/23213/55959/7045	20
BP	GO:0030282	bone mineralization	20/408	108/17913	4.16E-13	1.57E-10	1.15E-10	56172/54829/8313/658/1278/1311/4921/1893/5167/56975/26585/4015/4958/91461/5743/5745/5744/4920/7043/7291	20
BP	GO:0031214	biomineral tissue development	23/408	154/17913	8.26E-13	2.83E-10	2.07E-10	56172/54829/8313/658/1277/1278/1311/4921/1893/5167/56975/2621/26585/9622/4015/4958/91461/5743/5745/5744/4920/7043/7291	23
BP	GO:0007	cell-matrix adhesion	26/408	214/1791	3.52E-12	1.11E-09	8.10E-10	88/81792/1012/1307/1281/50509/1842/11117/2042/10979/2335/26585/22801/3690/22795/255743/90102/5328/10631/8499/5054/8910/25992/7057/7070/63923	26

© 2020 Yu Y et al. *JAMA Network Open*.

	160			3					
BP	GO:0001649	osteoblast differentiation	26/408	222/17913	8.21E-12	2.39E-09	1.74E-09	8313/658/1277/1291/90993/115908/4921/56975/2736/2737/26585/9464/3082/3488/22801/4015/9902/255743/5587/5745/6423/6591/3371/63923/7291/1462	26
BP	GO:0032963	collagen metabolic process	18/408	102/17913	1.47E-11	3.98E-09	2.91E-09	140766/9509/1306/1277/1278/1289/90993/1513/11117/2191/4320/4313/4314/9902/10536/5159/7043/7431	18
BP	GO:0001655	urogenital system development	31/408	324/17913	1.64E-11	4.14E-09	3.02E-09	59/170690/9370/185/9244/1588/9420/1634/2138/2200/2260/2303/2736/2737/84662/26585/3037/3764/55083/3976/255743/5125/5156/5159/5176/23213/55959/9096/7042/3371/7490	31
BP	GO:0030278	regulation of ossification	24/408	199/17913	2.85E-11	6.72E-09	4.91E-09	56172/8313/658/1311/115908/4921/1893/5167/56975/2737/26585/9464/3082/3488/255743/4958/91461/5587/6423/6591/7042/7043/63923/7291	24
BP	GO:0006024	glycosaminoglycan biosynthetic process	18/408	107/17913	3.40E-11	7.56E-09	5.52E-09	633/4166/1464/1634/29940/92126/2331/11227/2239/10082/3037/4060/4958/5159/5549/6383/1462/64131	18
BP	GO:0006023	aminoglycan biosynthetic process	18/408	108/17913	4.00E-11	8.39E-09	6.13E-09	633/4166/1464/1634/29940/92126/2331/11227/2239/10082/3037/4060/4958/5159/5549/6383/1462/64131	18

© 2020 Yu Y et al. *JAMA Network Open*.

BP	GO:0060485	mesenchyme development	27/408	258/17913	4.52E-11	8.99E-09	6.57E-09	59/8313/54796/1277/1909/2042/2260/2335/2303/26585/9464/3037/3082/3357/4017/8828/5159/10630/90102/6423/6591/7042/7043/7291/7472/7490/23414	27
BP	GO:0050673	epithelial cell proliferation	35/408	425/17913	5.41E-11	1.02E-08	7.47E-09	185/857/6356/1012/1295/1296/6387/9420/1893/2138/2191/2260/3037/3082/3357/5654/3488/3690/57608/687/4017/8828/5587/7857/5176/6423/6591/6678/23213/55959/7042/7057/7060/7291/7472	35
BP	GO:0007178	transmembrane receptor protein serine/threonine kinase signaling pathway	30/408	323/17913	7.38E-11	1.33E-08	9.70E-09	54829/658/857/8483/1278/1281/1311/11117/2200/10979/2331/11167/26585/5654/94031/3624/4015/2615/4053/255743/56937/4920/6423/6424/23213/7042/7043/7057/7431/23090	30
BP	GO:0002576	platelet degranulation	19/408	128/17913	9.24E-11	1.58E-08	1.16E-08	88/1893/2162/2316/2335/2621/3082/3671/3690/5099/5627/5919/5054/6678/7042/7043/7057/7076/7078	19
BP	GO:0072001	renal system development	28/408	291/17913	1.42E-10	2.34E-08	1.71E-08	59/170690/9370/185/9244/1634/2138/2200/2260/2303/2736/2737/84662/26585/3037/3764/55083/3976/255743/5125/5156/5159/5176/23213/55959/9096/7042/7490	28
BP	GO:0001822	kidney development	27/408	276/17913	2.11E-10	3.26E-08	2.38E-08	59/170690/9370/185/9244/1634/2138/2200/2260/2303/2736/2737/84662/26585/3037/3764/55083/3976/255743/5125/5156/5159/5176/23213/55959/7042/7490	27
BP	GO:00	regulation of cell-substrate adhesion	23/408	201/17	2.16E-1	3.26E-0	2.38E-0	151887/1012/1307/1277/1295/10085/2042/2316/2335/26585/3037/3678/3956/91663/255743/10630/90102/5328/10631/5054/6695/7057/7070	23

© 2020 Yu Y et al. *JAMA Network Open.*

	10810			913	0	8	8		
BP	GO:0071559	response to transforming growth factor beta	25/408	239/17913	2.43E-10	3.53E-08	2.58E-08	54829/857/8483/1277/1278/1281/11117/2200/10979/2331/5654/94031/4015/2615/4053/25878/50507/255743/5139/56937/10631/7042/7043/7057/7472	25
BP	GO:0060348	bone development	22/408	189/17913	3.85E-10	5.39E-08	3.94E-08	8313/658/1277/1311/56975/2200/2737/26585/3037/4015/10234/9060/56034/6423/6424/6678/6781/23213/55959/7043/7291/64131	22
BP	GO:0071560	cellular response to transforming growth factor beta stimulus	24/408	233/17913	7.67E-10	1.03E-07	7.56E-08	54829/857/8483/1277/1278/1281/11117/2200/10979/2331/5654/94031/4015/2615/4053/50507/255743/5139/56937/10631/7042/7043/7057/7472	24
BP	GO:0033627	cell adhesion mediated by integrin	13/408	61/17913	8.95E-10	1.16E-07	8.51E-08	1307/1545/2200/2303/22801/3678/3690/255743/5328/5054/6423/6591/7042	13
BP	GO:0048762	mesenchymal cell differentiation	22/408	199/17913	1.04E-09	1.31E-07	9.55E-08	8313/1277/1909/2042/2260/2335/2303/26585/9464/3037/3082/3357/4017/8828/10630/90102/6423/6591/7042/7043/7291/7472	22
BP	GO:0048771	tissue remodeling	20/408	170/17913	2.03E-09	2.47E-07	1.80E-07	558/633/857/1464/115908/1513/2303/2702/26585/9464/3488/3690/3953/3976/4286/50507/5745/7043/7060/7076	20

© 2020 Yu Y et al. *JAMA Network Open.*

BP	GO:0030203	glycosaminoglycan metabolic process	19/408	157/17913	3.20E-09	3.78E-07	2.76E-07	633/4166/1464/1634/29940/92126/2331/11227/2239/10082/3037/3082/4060/4958/5159/5549/6383/1462/64131	19
BP	GO:0060541	respiratory system development	21/408	193/17913	3.34E-09	3.82E-07	2.79E-07	9509/220/83716/2260/2627/2736/2737/3488/3976/4015/9242/5156/5159/10630/91461/6678/7043/3371/7472/7490/23414	21
BP	GO:0035987	endodermal cell differentiation	11/408	45/17913	3.79E-09	4.21E-07	3.07E-07	1301/1303/1289/1290/1291/1295/2335/2627/3624/3678/4313	11
BP	GO:0042476	odontogenesis	17/408	126/17913	4.10E-09	4.42E-07	3.23E-07	54829/8313/1277/1278/56975/2736/2737/9464/5654/3624/9622/5156/5054/7042/7043/3371/7291	17
BP	GO:0045765	regulation of angiogenesis	30/408	383/17913	4.39E-09	4.53E-07	3.31E-07	8038/185/6356/90993/1545/1634/1893/11117/2303/2627/26585/3082/3678/57608/4223/10769/5587/5740/5743/5054/5176/6423/6678/27286/23213/7042/7057/7058/7060/7291	30
BP	GO:0007369	gastrulation	20/408	178/17913	4.55E-09	4.53E-07	3.31E-07	9370/1301/1303/1289/1290/1291/1295/2138/2260/2335/2303/2627/3624/3678/3690/4313/91663/90102/6423/26011	20
BP	GO:00	regulation of chondrocyte differentiation	11/408	46/179	4.88E-0	4.53E-0	3.31E-0	81792/8313/658/2202/2736/2737/26585/4017/91461/5744/6591	11

© 2020 Yu Y et al. *JAMA Network Open.*

	32330			13	9	7	7		
BP	GO:0006022	aminoglycan metabolic process	19/408	161/17913	4.89E-09	4.53E-07	3.31E-07	633/4166/1464/1634/29940/92126/2331/11227/2239/10082/3037/3082/4060/4958/5159/5549/6383/1462/64131	19
BP	GO:0090287	regulation of cellular response to growth factor stimulus	25/408	276/17913	4.91E-09	4.53E-07	3.31E-07	81792/54829/857/90993/1634/11117/56975/2200/2260/26585/5654/94031/3678/3690/57608/4015/255743/56937/6423/6424/23213/55959/7043/7057/23090	25
BP	GO:0002063	chondrocyte development	9/408	27/17913	4.93E-09	4.53E-07	3.31E-07	8313/658/1301/1311/1893/5744/6423/23213/55959	9
BP	GO:0018108	peptidyl-tyrosine phosphorylation	28/408	346/17913	7.17E-09	6.29E-07	4.60E-07	9370/558/857/1272/9244/1464/4921/2202/2042/2260/2621/2690/26585/3082/3678/3690/3976/50507/4915/56034/5156/5159/91461/4919/4920/6423/7060/7070	28
BP	GO:0050678	regulation of epithelial cell proliferation	29/408	369/17913	7.43E-09	6.29E-07	4.60E-07	185/857/6356/1012/6387/9420/1893/2138/2260/3037/3357/5654/3690/57608/687/8828/5587/7857/5176/6423/6591/6678/23213/55959/7042/7057/7060/7291/7472	29
BP	GO:0070252	actin-mediated cell contraction	16/408	115/17913	7.49E-09	6.29E-07	4.60E-07	88/781/857/2316/122786/2702/23704/3764/4620/10630/6330/6444/6781/7168/7169/7431	16

© 2020 Yu Y et al. *JAMA Network Open*.

BP	GO:0060537	muscle tissue development	30/408	392/17913	7.50E-09	6.29E-07	4.60E-07	88/857/1301/1281/1634/2138/2260/80206/2303/2627/2702/26585/3488/57158/3776/4015/4223/9242/50507/5156/5159/5999/6444/26011/7042/7168/7291/7472/7490/23414	30
BP	GO:0018212	peptidyl-tyrosine modification	28/408	349/17913	8.67E-09	7.11E-07	5.20E-07	9370/558/857/1272/9244/1464/4921/2202/2042/2260/2621/2690/26585/3082/3678/3690/3976/50507/4915/56034/5156/5159/91461/4919/4920/6423/7060/7070	28
BP	GO:0001704	formation of primary germ layer	16/408	117/17913	9.66E-09	7.60E-07	5.55E-07	1301/1303/1289/1290/1291/1295/2138/2260/2335/2303/2627/3624/3678/3690/4313/6423	16
BP	GO:0060021	roof of mouth development	14/408	87/17913	9.67E-09	7.60E-07	5.55E-07	54796/2737/9464/3624/2615/4223/9242/5156/91461/5396/6591/7042/7043/7291	14
BP	GO:0090090	negative regulation of canonical Wnt signaling pathway	19/408	169/17913	1.10E-08	8.47E-07	6.19E-07	8313/80114/857/1000/115908/51339/2737/26585/23072/3489/85409/144165/27303/4920/6423/6424/6591/9096/63923	19
BP	GO:0070167	regulation of biomineral tissue development	14/408	88/17913	1.13E-08	8.50E-07	6.21E-07	56172/54829/658/1311/4921/1893/5167/56975/2621/26585/4958/91461/7043/7291	14
BP	GO:00	negative regulation of cell migration	27/408	332/17	1.21E-0	8.73E-0	6.37E-0	9370/4345/1281/6387/1588/1545/1634/1809/11117/26585/3488/4223/4286/89795/90102/127435/5592/5054/5176/6423/6781/23213/7057/7070/7076/63923/7168	27

© 2020 Yu Y et al. *JAMA Network Open*.

	30336			913	8	7	7		
BP	GO:0003012	muscle system process	32/408	448/17913	1.23E-08	8.73E-07	6.37E-07	59/88/781/800/857/1311/1410/1909/2316/2627/2702/2982/9464/3357/3488/23704/3764/29995/4620/10398/255743/5592/5743/5999/6330/6444/6781/23213/55959/7168/7169/7431	32
BP	GO:0001706	endoderm formation	11/408	50/17913	1.26E-08	8.73E-07	6.37E-07	1301/1303/1289/1290/1291/1295/2335/2627/3624/3678/4313	11
BP	GO:1900047	negative regulation of hemostasis	11/408	50/17913	1.26E-08	8.73E-07	6.37E-07	170692/114897/1311/2191/5156/5328/5592/5627/5054/5270/7057	11
BP	GO:1900046	regulation of hemostasis	13/408	75/17913	1.29E-08	8.73E-07	6.37E-07	170692/114897/857/1311/2191/5156/10630/5328/5592/5627/5054/5270/7057	13
BP	GO:1901342	regulation of vasculature development	31/408	425/17913	1.29E-08	8.73E-07	6.37E-07	8038/185/6356/90993/1545/1634/1893/11117/2303/2627/26585/3082/3678/57608/4223/10769/5587/5740/5743/5054/5176/6423/6678/27286/23213/7042/7057/7058/7060/7291/7490	31
BP	GO:2000027	regulation of animal organ morphogenesis	23/408	250/17913	1.53E-08	1.01E-06	7.39E-07	54829/115908/51339/2138/2260/2303/2239/10082/26585/9464/3082/3976/144165/4919/4920/6423/6591/23213/55959/7042/7291/7472/7490	23

BP	GO:0030323	respiratory tube development	19/408	173/17913	1.62E-08	1.05E-06	7.69E-07	9509/83716/2260/2627/2736/2737/3488/3976/4015/5125/5156/5159/10630/91461/6678/7043/3371/7472/23414	19
BP	GO:0022617	extracellular matrix disassembly	13/408	78/17913	2.12E-08	1.35E-06	9.89E-07	9507/1513/4921/2191/5654/4320/4327/4313/4314/10630/7076/7077/7092	13
BP	GO:0001654	eye development	27/408	342/17913	2.26E-08	1.42E-06	1.04E-06	170692/220/658/1289/1290/1295/1296/1410/1545/2202/2200/2303/2737/3624/4237/4286/4915/5156/5159/5176/55714/7042/7070/7291/7431/7472/7490	27
BP	GO:0006936	muscle contraction	27/408	343/17913	2.40E-08	1.48E-06	1.08E-06	59/88/781/800/857/1311/1410/1909/2316/2702/2982/3357/23704/3764/4620/10398/255743/5592/5743/6330/6444/6781/23213/55959/7168/7169/7431	27
BP	GO:0061035	regulation of cartilage development	12/408	66/17913	2.69E-08	1.64E-06	1.20E-06	81792/8313/658/1513/2202/2736/2737/26585/4017/91461/5744/6591	12
BP	GO:0035265	organ growth	19/408	179/17913	2.83E-08	1.69E-06	1.24E-06	54796/1311/1588/4921/1893/2121/2260/2303/2627/3776/3953/5159/5999/6781/26011/7042/7472/7490/23414	19
BP	GO:01	visual system development	27/408	346/17	2.88E-0	1.70E-0	1.24E-0	170692/220/658/1289/1290/1295/1296/1410/1545/2202/2200/2303/2737/3624/4237/4286/4915/5156/5159/5176/55714/7042/7070/7291/7431/7472/7490	27

	50063			913	8	6	6		
BP	GO:2000146	negative regulation of cell motility	27/408	347/17913	3.06E-08	1.77E-06	1.30E-06	9370/4345/1281/6387/1588/1545/1634/1809/11117/26585/3488/4223/4286/89795/90102/127435/5592/5054/5176/6423/6781/23213/7057/7070/7076/63923/7168	27
BP	GO:0045667	regulation of osteoblast differentiation	16/408	128/17913	3.56E-08	2.04E-06	1.49E-06	8313/658/115908/4921/56975/2737/26585/9464/3082/3488/255743/5587/6423/6591/63923/7291	16
BP	GO:0048738	cardiac muscle tissue development	21/408	221/17913	3.72E-08	2.09E-06	1.53E-06	88/1301/2260/80206/2303/2627/2702/26585/57158/3776/50507/5156/5159/5999/6444/26011/7042/7168/7472/7490/23414	21
BP	GO:0048880	sensory system development	27/408	351/17913	3.88E-08	2.15E-06	1.57E-06	170692/220/658/1289/1290/1295/1296/1410/1545/2202/2200/2303/2737/3624/4237/4286/4915/5156/5159/5176/55714/7042/7070/7291/7431/7472/7490	27
BP	GO:0045766	positive regulation of angiogenesis	20/408	203/17913	4.31E-08	2.33E-06	1.70E-06	8038/185/6356/1545/1893/2303/2627/26585/3082/3678/57608/10769/5587/5740/5743/5054/6423/27286/7057/7291	20
BP	GO:0014706	striated muscle tissue development	28/408	376/17913	4.31E-08	2.33E-06	1.70E-06	88/857/1301/1634/2138/2260/80206/2303/2627/2702/26585/57158/3776/4015/4223/9242/50507/5156/5159/5999/6444/26011/7042/7168/7291/7472/7490/23414	28

© 2020 Yu Y et al. *JAMA Network Open*.

BP	GO:0048705	skeletal system morphogenesis	20/408	204/17913	4.68E-08	2.49E-06	1.82E-06	8313/658/1301/1277/1311/2138/2260/2303/2737/26585/3037/4313/5156/5396/4920/6423/6424/6781/7043/7291	20
BP	GO:0001935	endothelial cell proliferation	19/408	185/17913	4.83E-08	2.53E-06	1.85E-06	185/857/6356/1012/6387/1893/2260/3357/3690/57608/4017/8828/5587/7857/6678/23213/7057/7060/7472	19
BP	GO:0007517	muscle organ development	29/408	403/17913	5.19E-08	2.68E-06	1.96E-06	857/1301/1281/1293/1410/1634/2115/2121/2260/2303/2627/26585/22801/57158/3776/3976/4015/4223/9242/5999/6444/8910/6876/7042/7168/7291/7472/7490/23414	29
BP	GO:0030048	actin filament-based movement	16/408	132/17913	5.53E-08	2.82E-06	2.06E-06	88/781/857/2316/122786/2702/23704/3764/4620/10630/6330/6444/6781/7168/7169/7431	16
BP	GO:0060840	artery development	14/408	100/17913	6.04E-08	3.04E-06	2.22E-06	11174/1281/1311/2138/2303/2702/2737/9464/4015/4016/5159/144165/5396/7042	14
BP	GO:0030324	lung development	18/408	169/17913	6.23E-08	3.10E-06	2.26E-06	9509/83716/2260/2627/2736/2737/3488/3976/4015/5156/5159/10630/91461/6678/7043/3371/7472/23414	18
BP	GO:00	regulation of bone mineralization	12/408	71/179	6.32E-0	3.10E-0	2.26E-0	56172/658/1311/4921/1893/5167/56975/26585/4958/91461/7043/7291	12

© 2020 Yu Y et al. *JAMA Network Open*.

	30500			13	8	6	6		
BP	GO:0070527	platelet aggregation	11/408	58/17913	6.51E-08	3.15E-06	2.30E-06	170692/114897/1311/2316/2621/3690/10398/5156/10630/5592/5270	11
BP	GO:0001936	regulation of endothelial cell proliferation	18/408	170/17913	6.83E-08	3.26E-06	2.38E-06	185/857/6356/1012/6387/1893/2260/3357/3690/57608/8828/5587/7857/6678/23213/7057/7060/7472	18
BP	GO:0051271	negative regulation of cellular component movement	27/408	365/17913	8.66E-08	4.09E-06	2.99E-06	9370/4345/1281/6387/1588/1545/1634/1809/11117/26585/3488/4223/4286/89795/90102/127435/5592/5054/5176/6423/6781/23213/7057/7070/7076/63923/7168	27
BP	GO:0030193	regulation of blood coagulation	12/408	74/17913	1.02E-07	4.74E-06	3.47E-06	170692/114897/857/2191/5156/10630/5328/5592/5627/5054/5270/7057	12
BP	GO:0030574	collagen catabolic process	9/408	37/17913	1.07E-07	4.92E-06	3.59E-06	140766/9509/1306/1513/2191/4320/4313/4314/9902	9
BP	GO:0030208	dermatan sulfate biosynthetic process	6/408	12/17913	1.11E-07	5.04E-06	3.68E-06	633/1464/1634/29940/92126/1462	6

© 2020 Yu Y et al. *JAMA Network Open*.

BP	GO:0006027	glycosaminoglycan catabolic process	11/408	61/17913	1.12E-07	5.06E-06	3.69E-06	633/1464/1634/2331/2239/10082/4060/4958/5549/6383/1462	11
BP	GO:0030195	negative regulation of blood coagulation	10/408	49/17913	1.25E-07	5.56E-06	4.06E-06	170692/114897/2191/5156/5328/5592/5627/5054/5270/7057	10
BP	GO:0001667	ameboidal-type cell migration	30/408	446/17913	1.34E-07	5.90E-06	4.31E-06	9590/1012/1545/1634/4921/2191/2260/2335/2303/26585/9464/3037/3357/3690/57608/4017/4223/8828/10769/5587/5743/7857/5176/6678/27286/6781/7042/7057/7076/7291	30
BP	GO:0030178	negative regulation of Wnt signaling pathway	19/408	198/17913	1.43E-07	6.13E-06	4.48E-06	8313/80114/857/1000/115908/51339/2737/26585/23072/3489/85409/144165/27303/4920/6423/6424/6591/9096/63923	19
BP	GO:0001938	positive regulation of endothelial cell proliferation	14/408	107/17913	1.44E-07	6.13E-06	4.48E-06	185/6356/1012/6387/1893/2260/3357/3690/57608/8828/5587/7857/7060/7472	14
BP	GO:0016055	Wnt signaling pathway	32/408	498/17913	1.44E-07	6.13E-06	4.48E-06	8313/80114/857/1000/1277/1363/8532/115908/51339/10979/2737/2239/10082/26585/23072/3489/4286/85409/144165/26108/27303/4919/4920/6423/6424/6591/23213/55959/9096/83439/63923/7472	32
BP	GO:00	positive regulation of epithelial cell proliferation	19/408	199/17	1.55E-0	6.48E-0	4.74E-0	185/6356/1012/6387/9420/1893/2138/2260/3037/3357/5654/3690/57608/8828/5587/7857/7060/7291/7472	19

© 2020 Yu Y et al. *JAMA Network Open.*

	50679			913	7	6	6		
BP	GO:0198738	cell-cell signaling by wnt	32/408	500/17913	1.58E-07	6.51E-06	4.75E-06	8313/80114/857/1000/1277/1363/8532/115908/51339/10979/2737/2239/10082/26585/23072/3489/4286/85409/144165/26108/27303/4919/4920/6423/6424/6591/23213/55959/9096/83439/63923/7472	32
BP	GO:0006026	aminoglycan catabolic process	11/408	63/17913	1.59E-07	6.51E-06	4.75E-06	633/1464/1634/2331/2239/10082/4060/4958/5549/6383/1462	11
BP	GO:0034109	homotypic cell-cell adhesion	12/408	77/17913	1.60E-07	6.51E-06	4.75E-06	170692/114897/1311/2316/2621/3690/3956/10398/5156/10630/5592/5270	12
BP	GO:0040013	negative regulation of locomotion	27/408	377/17913	1.67E-07	6.70E-06	4.89E-06	9370/4345/1281/6387/1588/1545/1634/1809/11117/26585/3488/4223/4286/89795/90102/127435/5592/5054/5176/6423/6781/23213/7057/7070/7076/63923/7168	27
BP	GO:0007596	blood coagulation	25/408	331/17913	1.77E-07	7.04E-06	5.14E-06	170692/114897/857/1277/1278/1281/1311/2162/2151/2191/2316/2621/2627/3690/10398/9060/5156/10630/5328/5592/5627/5054/5270/7057/23414	25
BP	GO:0030205	dermatan sulfate metabolic process	6/408	13/17913	2.02E-07	7.85E-06	5.74E-06	633/1464/1634/29940/92126/1462	6

BP	GO:0070208	protein heterotrimerization	6/408	13/17913	2.02E-07	7.85E-06	5.74E-06	9370/114897/1277/1278/1291/1292	6
BP	GO:0060070	canonical Wnt signaling pathway	24/408	311/17913	2.11E-07	8.09E-06	5.91E-06	8313/80114/857/1000/1277/115908/51339/2737/26585/23072/3489/4286/85409/144165/26108/27303/4920/6423/6424/6591/55959/9096/63923/7472	24
BP	GO:0006029	proteoglycan metabolic process	12/408	79/17913	2.14E-07	8.09E-06	5.91E-06	81792/633/658/1301/1464/1634/29940/92126/23213/55959/1462/64131	12
BP	GO:0050818	regulation of coagulation	12/408	79/17913	2.14E-07	8.09E-06	5.91E-06	170692/114897/857/2191/5156/10630/5328/5592/5627/5054/5270/7057	12
BP	GO:0007179	transforming growth factor beta receptor signaling pathway	18/408	184/17913	2.28E-07	8.53E-06	6.23E-06	54829/857/1278/1281/11117/2200/10979/2331/5654/94031/4015/2615/4053/255743/56937/7042/7043/7057	18
BP	GO:0007599	hemostasis	25/408	336/17913	2.36E-07	8.72E-06	6.37E-06	170692/114897/857/1277/1278/1281/1311/2162/2151/2191/2316/2621/2627/3690/10398/9060/5156/10630/5328/5592/5627/5054/5270/7057/23414	25
BP	GO:00	coagulation	25/408	337/17	2.49E-0	9.13E-0	6.67E-0	170692/114897/857/1277/1278/1281/1311/2162/2151/2191/2316/2621/2627/3690/10398/9060/5156/10630/5328/5592/5627/5054/5270/7057/23414	25

© 2020 Yu Y et al. *JAMA Network Open.*

	50817			913	7	6	6		
BP	GO:1903510	mucopolysaccharide metabolic process	14/408	112/17913	2.56E-07	9.30E-06	6.79E-06	633/4166/1464/1634/29940/92126/2331/3037/3082/4060/4958/5549/1462/64131	14
BP	GO:0050819	negative regulation of coagulation	10/408	53/17913	2.74E-07	9.85E-06	7.19E-06	170692/114897/2191/5156/5328/5592/5627/5054/5270/7057	10
BP	GO:0090596	sensory organ morphogenesis	21/408	250/17913	3.04E-07	1.08E-05	7.91E-06	220/1301/1289/1290/1295/1296/115908/2202/2138/2200/2260/2736/2737/4237/4915/5396/4920/55714/7070/7291/7472	21
BP	GO:0043010	camera-type eye development	23/408	295/17913	3.16E-07	1.12E-05	8.15E-06	220/658/1295/1296/1410/1545/2202/2200/2303/2737/3624/4286/4915/5156/5159/5176/55714/7042/7070/7291/7431/7472/7490	23
BP	GO:0008217	regulation of blood pressure	17/408	169/17913	3.32E-07	1.16E-05	8.48E-06	59/170690/9370/185/552/1066/1278/10699/1909/2621/2702/2982/4883/5125/10631/5743/7168	17
BP	GO:1904018	positive regulation of vasculature development	20/408	232/17913	3.85E-07	1.33E-05	9.74E-06	8038/185/6356/1545/1893/2303/2627/26585/3082/3678/57608/10769/5587/5740/5743/5054/6423/27286/7057/7291	20

© 2020 Yu Y et al. *JAMA Network Open*.

BP	GO:0022612	gland morphogenesis	14/408	116/17913	3.97E-07	1.36E-05	9.96E-06	857/6356/9420/2260/2736/2737/3082/3488/6591/23213/55959/7042/7043/3371	14
BP	GO:0050651	dermatan sulfate proteoglycan biosynthetic process	6/408	15/17913	5.66E-07	1.89E-05	1.38E-05	633/1464/1634/29940/92126/1462	6
BP	GO:0090288	negative regulation of cellular response to growth factor stimulus	16/408	156/17913	5.66E-07	1.89E-05	1.38E-05	81792/54829/857/90993/1634/11117/2200/26585/5654/94031/56937/6423/23213/55959/7043/7057	16
BP	GO:0048608	reproductive structure development	28/408	426/17913	5.66E-07	1.89E-05	1.38E-05	558/658/1588/9420/1634/2316/2627/2736/2737/5654/3624/3976/4327/5156/5159/5740/5743/4920/5270/5176/6423/6781/23213/7042/3371/7472/7490/23414	28
BP	GO:0048736	appendage development	17/408	176/17913	5.92E-07	1.94E-05	1.42E-05	658/1311/2260/2702/2736/2737/26585/9464/4223/5125/91461/5396/4920/57167/6423/7042/7291	17
BP	GO:0060173	limb development	17/408	176/17913	5.92E-07	1.94E-05	1.42E-05	658/1311/2260/2702/2736/2737/26585/9464/4223/5125/91461/5396/4920/57167/6423/7042/7291	17
BP	GO:00	reproductive system development	28/408	429/17	6.52E-0	2.12E-0	1.55E-0	558/658/1588/9420/1634/2316/2627/2736/2737/5654/3624/3976/4327/5156/5159/5740/5743/4920/5270/5176/6423/6781/23213/7042/3371/7472/7490/23414	28

© 2020 Yu Y et al. *JAMA Network Open.*

	61458			913	7	5	5		
BP	GO:0061138	morphogenesis of a branching epithelium	17/408	178/17913	6.95E-07	2.24E-05	1.64E-05	170690/6356/1909/2138/2260/2303/2736/2737/26585/3082/255743/6423/6591/23213/3371/7472/7490	17
BP	GO:0001837	epithelial to mesenchymal transition	15/408	140/17913	7.30E-07	2.33E-05	1.71E-05	8313/1277/2042/26585/3037/3082/4017/10630/90102/6423/6591/7042/7043/7291/7472	15
BP	GO:0060828	regulation of canonical Wnt signaling pathway	21/408	264/17913	7.47E-07	2.34E-05	1.71E-05	8313/80114/857/1000/1277/115908/51339/2737/26585/23072/3489/85409/144165/27303/4920/6423/6424/6591/55959/9096/63923	21
BP	GO:0007492	endoderm development	11/408	73/17913	7.49E-07	2.34E-05	1.71E-05	1301/1303/1289/1290/1291/1295/2335/2627/3624/3678/4313	11
BP	GO:0061045	negative regulation of wound healing	11/408	73/17913	7.49E-07	2.34E-05	1.71E-05	170692/114897/2191/5156/90102/5328/5592/5627/5054/5270/7057	11
BP	GO:0072006	nephron development	15/408	142/17913	8.76E-07	2.70E-05	1.97E-05	59/170690/9370/2138/2303/2737/26585/55083/3976/255743/5156/5159/23213/55959/7490	15

© 2020 Yu Y et al. *JAMA Network Open.*

BP	GO:1905330	regulation of morphogenesis of an epithelium	17/408	181/17913	8.79E-07	2.70E-05	1.97E-05	81792/115908/51339/2260/2239/10082/26585/3082/3976/90102/144165/4919/4920/6423/6591/23213/7472	17
BP	GO:0050655	dermatan sulfate proteoglycan metabolic process	6/408	16/17913	8.88E-07	2.70E-05	1.98E-05	633/1464/1634/29940/92126/1462	6
BP	GO:0048645	animal organ formation	10/408	61/17913	1.08E-06	3.25E-05	2.38E-05	8313/2138/2260/2627/2736/2737/9464/23213/7472/7490	10
BP	GO:0007162	negative regulation of cell adhesion	21/408	271/17913	1.14E-06	3.42E-05	2.50E-05	170692/9370/114897/1012/1277/6387/1545/2737/3956/2615/91663/90102/10631/5592/5054/5270/6591/6695/7045/7057/3371	21
BP	GO:0044273	sulfur compound catabolic process	9/408	48/17913	1.15E-06	3.43E-05	2.51E-05	633/1464/1634/2331/2687/4060/4958/5549/1462	9
BP	GO:0003007	heart morphogenesis	20/408	249/17913	1.18E-06	3.48E-05	2.54E-05	8313/1301/1289/1363/2138/23768/2303/2627/2702/9464/3037/3357/8828/6423/6591/7042/7168/7291/7472/23414	20
BP	GO:00	appendage morphogenesis	15/408	146/17	1.25E-0	3.63E-0	2.65E-0	658/2260/2702/2736/2737/26585/9464/5125/91461/5396/4920/57167/6423/7042/7291	15

	35107			913	6	5	5		
BP	GO:0035108	limb morphogenesis	15/408	146/17913	1.25E-06	3.63E-05	2.65E-05	658/2260/2702/2736/2737/26585/9464/5125/91461/5396/4920/57167/6423/7042/7291	15
BP	GO:0061037	negative regulation of cartilage development	7/408	26/17913	1.37E-06	3.91E-05	2.86E-05	81792/1513/2202/2736/26585/5744/6591	7
BP	GO:0098868	bone growth	7/408	26/17913	1.37E-06	3.91E-05	2.86E-05	54796/1311/4921/1893/2121/3953/6781	7
BP	GO:0043542	endothelial cell migration	21/408	275/17913	1.45E-06	4.11E-05	3.00E-05	1012/1545/1634/2191/2260/2303/26585/3690/57608/4017/4223/8828/10769/5587/5743/7857/5176/6678/27286/6781/7057	21
BP	GO:0045778	positive regulation of ossification	12/408	94/17913	1.47E-06	4.14E-05	3.03E-05	658/115908/4921/56975/2737/3082/255743/91461/5587/6423/7042/7043	12
BP	GO:0071230	cellular response to amino acid stimulus	10/408	64/17913	1.70E-06	4.76E-05	3.48E-05	1307/1277/1278/1281/1290/1291/4313/4915/56034/5156	10

© 2020 Yu Y et al. *JAMA Network Open.*

BP	GO:0003205	cardiac chamber development	16/408	170/17913	1.79E-06	4.98E-05	3.64E-05	11174/1301/1363/2303/2627/2702/9464/3776/8828/5125/57167/6423/7042/7168/7472/23414	16
BP	GO:0048565	digestive tract development	14/408	132/17913	1.93E-06	5.32E-05	3.89E-05	79827/1281/51339/2627/2736/2737/5125/56034/5156/91461/5919/6423/7042/7043	14
BP	GO:0001763	morphogenesis of a branching structure	17/408	192/17913	2.00E-06	5.46E-05	3.99E-05	170690/6356/1909/2138/2260/2303/2736/2737/26585/3082/255743/6423/6591/23213/3371/7472/7490	17
BP	GO:0030168	platelet activation	15/408	152/17913	2.08E-06	5.66E-05	4.13E-05	170692/114897/1277/1278/1281/1311/2151/2316/2621/3690/10398/5156/10630/5592/5270	15
BP	GO:0090092	regulation of transmembrane receptor protein serine/threonine kinase signaling pathway	18/408	215/17913	2.24E-06	6.05E-05	4.42E-05	54829/857/8483/11117/2200/26585/5654/94031/3624/4015/255743/56937/6423/6424/23213/7043/7057/23090	18
BP	GO:0007566	embryo implantation	9/408	52/17913	2.34E-06	6.25E-05	4.57E-05	3490/3976/4313/5125/5740/5743/6781/7076/7216	9
BP	GO:00	cardiocyte differentiation	15/408	154/17	2.46E-0	6.53E-0	4.77E-0	88/80206/2627/26585/9464/50507/5156/5159/144165/5999/6444/26011/7042/7291/7490	15

© 2020 Yu Y et al. *JAMA Network Open.*

	35051			913	6	5	5		
BP	GO:0048706	embryonic skeletal system development	13/408	117/17913	2.66E-06	7.03E-05	5.13E-05	1301/1277/2138/2303/2737/5125/5156/5396/23213/55959/7043/7291/64131	13
BP	GO:0032331	negative regulation of chondrocyte differentiation	6/408	19/17913	2.84E-06	7.44E-05	5.44E-05	81792/2202/2736/26585/5744/6591	6
BP	GO:0048568	embryonic organ development	26/408	413/17913	3.20E-06	8.32E-05	6.08E-05	220/1301/115908/2202/2138/2200/2260/2303/2702/2736/2737/9464/3976/4237/5125/56034/5156/91461/5396/5919/4920/7042/7043/7291/7472/23414	26
BP	GO:0033002	muscle cell proliferation	19/408	243/17913	3.25E-06	8.39E-05	6.13E-05	9370/1012/2260/2303/2627/3488/3776/4313/4883/5159/5592/5743/26011/7042/7043/7057/7168/7472/23414	19
BP	GO:0090130	tissue migration	24/408	363/17913	3.33E-06	8.53E-05	6.23E-05	59/1012/1545/1634/2191/2260/2303/26585/3037/3690/57608/4017/4223/8828/10769/5587/5743/7857/5176/6678/27286/6781/7042/7057	24
BP	GO:1903053	regulation of extracellular matrix organization	8/408	41/17913	3.34E-06	8.53E-05	6.23E-05	165/84168/4921/11117/2191/3037/10630/90102	8

© 2020 Yu Y et al. *JAMA Network Open*.

BP	GO:0030111	regulation of Wnt signaling pathway	23/408	341/17913	3.83E-06	9.70E-05	7.09E-05	8313/80114/857/1000/1277/115908/51339/2737/26585/23072/3489/85409/144165/27303/4920/6423/6424/6591/23213/55959/9096/83439/63923	23
BP	GO:0014812	muscle cell migration	12/408	103/17913	3.92E-06	9.85E-05	7.20E-05	9370/3037/3488/3690/50507/5159/5328/10631/5592/5054/7060/7168	12
BP	GO:0044706	multi-multicellular organism process	18/408	225/17913	4.27E-06	0.000106749	7.80E-05	552/1307/10699/1833/3488/3490/3678/3976/4313/5125/5740/5743/5744/5270/6781/7043/7076/7216	18
BP	GO:1903035	negative regulation of response to wounding	11/408	87/17913	4.43E-06	0.000110109	8.04E-05	170692/114897/2191/5156/90102/5328/5592/5627/5054/5270/7057	11
BP	GO:0010470	regulation of gastrulation	8/408	43/17913	4.88E-06	0.000120358	8.79E-05	9370/1289/1290/2260/91663/90102/6423/26011	8
BP	GO:0055123	digestive system development	14/408	143/17913	5.00E-06	0.000121751	8.89E-05	79827/1281/51339/2627/2736/2737/5125/56034/5156/91461/5919/6423/7042/7043	14
BP	GO:00	regulation of wound healing	14/408	143/17	5.00E-0	0.00012	8.89E-0	170692/114897/857/2191/2303/5156/10630/90102/5328/5592/5627/5054/5270/7057	14

© 2020 Yu Y et al. *JAMA Network Open*.

	61041			913	6	1751	5		
BP	GO:0060326	cell chemotaxis	19/408	253/17913	5.84E-06	0.0000141348	0.0000103265	185/6356/6387/1588/9420/2260/2621/26585/3082/4015/5156/5159/5587/5919/7857/5054/7042/7057/7060	19
BP	GO:0048592	eye morphogenesis	14/408	145/17913	5.88E-06	0.0000141426	0.0000103321	220/1289/1290/1295/1296/2202/2200/2737/4237/4915/55714/7070/7291/7472	14
BP	GO:0034330	cell junction organization	20/408	277/17913	6.02E-06	0.0000143733	0.0000105007	88/857/1009/1012/1000/1307/2042/10979/2316/2335/2702/26585/3678/90102/11228/6591/7042/7043/7057/7070	20
BP	GO:0060349	bone morphogenesis	11/408	90/17913	6.20E-06	0.0000147111	0.0000107474	8313/658/1277/1311/2737/3037/6423/6424/6781/7043/7291	11
BP	GO:0071772	response to BMP	14/408	147/17913	6.90E-06	0.0000158968	0.0000116137	81792/658/1311/2200/11167/2627/26585/5654/94031/4920/6423/6424/23213/23090	14
BP	GO:0071773	cellular response to BMP stimulus	14/408	147/17913	6.90E-06	0.0000158968	0.0000116137	81792/658/1311/2200/11167/2627/26585/5654/94031/4920/6423/6424/23213/23090	14

BP	GO:0001656	metanephros development	11/408	91/17913	6.91E-06	0.000158968	0.000116137	9370/2138/2200/2303/2737/26585/55083/3976/5156/5159/7490	11
BP	GO:0010717	regulation of epithelial to mesenchymal transition	11/408	91/17913	6.91E-06	0.000158968	0.000116137	8313/1277/2042/26585/4017/10630/90102/6423/7042/7043/7291	11
BP	GO:0014909	smooth muscle cell migration	11/408	91/17913	6.91E-06	0.000158968	0.000116137	9370/3037/3488/3690/50507/5159/5328/10631/5592/5054/7168	11
BP	GO:0010631	epithelial cell migration	23/408	354/17913	7.10E-06	0.000162552	0.000118755	1012/1545/1634/2191/2260/2303/26585/3037/3690/57608/4017/4223/8828/10769/5587/5743/7857/5176/6678/27286/6781/7042/7057	23
BP	GO:0010811	positive regulation of cell-substrate adhesion	12/408	110/17913	7.80E-06	0.000177477	0.000129659	151887/1012/1307/1295/10085/2316/2335/3037/3678/91663/255743/7070	12
BP	GO:0090132	epithelium migration	23/408	357/17913	8.15E-06	0.000184331	0.000134667	1012/1545/1634/2191/2260/2303/26585/3037/3690/57608/4017/4223/8828/10769/5587/5743/7857/5176/6678/27286/6781/7042/7057	23
BP	GO:00	female pregnancy	16/408	193/17	9.28E-0	0.00020	0.00015	1307/10699/1833/3488/3490/3678/3976/4313/5125/5740/5743/5744/6781/7043/7076/7216	16

© 2020 Yu Y et al. *JAMA Network Open.*

	0756 5			913	6	8464	2297		
BP	GO:0048566	embryonic digestive tract development	7/408	34/17913	9.55E-06	0.0002133329	0.0001555852	2736/2737/5125/5156/91461/5919/7042	7
BP	GO:0003416	endochondral bone growth	6/408	23/17913	9.78E-06	0.0002171777	0.0001586662	54796/1311/4921/1893/2121/6781	6
BP	GO:0001764	neuron migration	14/408	152/17913	1.02E-05	0.0002243316	0.0001633878	558/1281/6387/2260/2316/23768/2621/4131/8828/4915/5592/6695/63923/7291	14
BP	GO:0060562	epithelial tube morphogenesis	21/408	313/17913	1.10E-05	0.0002403334	0.000175558	81792/170690/6356/115908/1909/2138/2303/2736/2737/26585/9464/55083/255743/144165/57167/6423/7042/3371/7291/7472/7490	21
BP	GO:0050878	regulation of body fluid levels	28/408	499/17913	1.18E-05	0.0002580894	0.0001885555	170692/114897/857/1277/1278/1281/1311/2162/2151/2191/2316/2621/2627/2702/3037/3690/10398/4883/9060/5156/10630/5328/5592/5627/5054/5270/7057/23414	28
BP	GO:0003151	outflow tract morphogenesis	10/408	80/17913	1.34E-05	0.0002903312	0.0002122093	2138/2303/2627/2702/9464/8828/6423/7042/7291/23414	10

© 2020 Yu Y et al. *JAMA Network Open*.

BP	GO:0050730	regulation of peptidyl-tyrosine phosphorylation	18/408	245/17913	1.38E-05	0.000298572	0.000218127	9370/857/1272/9244/1464/2621/2690/26585/3082/3678/3690/3976/50507/4915/56034/6423/7060/7070	18
BP	GO:0071634	regulation of transforming growth factor beta production	7/408	36/17913	1.42E-05	0.000303896	0.000222017	4345/2627/2615/4060/5743/7042/7057	7
BP	GO:0098801	regulation of renal system process	7/408	36/17913	1.42E-05	0.000303896	0.000222017	9370/185/552/10699/2621/2702/6781	7
BP	GO:0034110	regulation of homotypic cell-cell adhesion	6/408	25/17913	1.65E-05	0.000350108	0.000255778	170692/114897/3956/10630/5592/5270	6
BP	GO:0003073	regulation of systemic arterial blood pressure	10/408	83/17913	1.86E-05	0.000392367	0.000286651	170690/185/552/1066/10699/2621/2702/5125/10631/7168	10
BP	GO:0003014	renal system process	12/408	120/17913	1.91E-05	0.000399923	0.000292171	9370/185/552/10699/2621/2702/3037/4883/5125/6781/23213/55959	12
BP	GO:00	positive regulation of osteoblast differentiation	9/408	67/179	2.01E-0	0.00041	0.00030	658/115908/4921/56975/2737/3082/255743/5587/6423	9

© 2020 Yu Y et al. *JAMA Network Open*.

	45669			13	5	889	6028		
BP	GO:0071604	transforming growth factor beta production	7/408	38/17913	2.07E-05	0.000429621	0.000313868	4345/2627/2615/4060/5743/7042/7057	7
BP	GO:0030206	chondroitin sulfate biosynthetic process	6/408	26/17913	2.11E-05	0.000434247	0.000317247	633/1464/1634/29940/1462/64131	6
BP	GO:0042698	ovulation cycle	9/408	68/17913	2.27E-05	0.000462974	0.000338234	558/658/3037/3624/4327/5156/5176/7042/7043	9
BP	GO:0086003	cardiac muscle cell contraction	9/408	68/17913	2.27E-05	0.000462974	0.000338234	781/857/2316/2702/23704/3764/6330/6444/6781	9
BP	GO:0048839	inner ear development	15/408	185/17913	2.29E-05	0.000464047	0.000339018	220/1301/115908/2138/2260/2736/2737/3776/5159/5396/4919/4920/6678/7042/7043	15
BP	GO:0014910	regulation of smooth muscle cell migration	10/408	85/17913	2.30E-05	0.000464047	0.000339018	9370/3037/3488/50507/5159/5328/10631/5592/5054/7168	10

BP	GO:0050920	regulation of chemotaxis	15/408	186/17913	2.44E-05	0.000481119	0.00035149	1012/6387/1588/2260/2621/26585/5156/5159/91584/5587/5919/7857/5054/7057/7060	15
BP	GO:0030166	proteoglycan biosynthetic process	8/408	53/17913	2.44E-05	0.000481119	0.00035149	633/658/1464/1634/29940/92126/1462/64131	8
BP	GO:0032964	collagen biosynthetic process	8/408	53/17913	2.44E-05	0.000481119	0.00035149	1277/1289/90993/11117/10536/5159/7043/7431	8
BP	GO:0030326	embryonic limb morphogenesis	12/408	123/17913	2.45E-05	0.000481119	0.00035149	2260/2702/2736/2737/26585/9464/5396/4920/57167/6423/7042/7291	12
BP	GO:0035113	embryonic appendage morphogenesis	12/408	123/17913	2.45E-05	0.000481119	0.00035149	2260/2702/2736/2737/26585/9464/5396/4920/57167/6423/7042/7291	12
BP	GO:0030204	chondroitin sulfate metabolic process	7/408	39/17913	2.48E-05	0.000484169	0.00035371 8	633/1464/1634/29940/92126/1462/64131	7
BP	GO:00	substrate adhesion-dependent cell spreading	10/408	86/179	2.55E-0	0.00049	0.00036	84168/558/10979/2316/2335/3037/3690/91663/10630/10631	10

	34446			13	5	6039	239		
BP	GO:0006939	smooth muscle contraction	11/408	105/17913	2.74E-05	0.000529479	0.000386821	59/857/1311/1909/2982/3357/255743/5592/5743/23213/55959	11
BP	GO:0061005	cell differentiation involved in kidney development	8/408	54/17913	2.81E-05	0.000541728	0.000395769	59/9370/2303/2737/84662/26585/3976/7490	8
BP	GO:0043583	ear development	16/408	212/17913	2.98E-05	0.000571786	0.000417729	220/1301/115908/2138/2260/2736/2737/3776/5159/5396/4919/4920/6678/7042/7043/7291	16
BP	GO:0048754	branching morphogenesis of an epithelial tube	13/408	146/17913	3.01E-05	0.000573668	0.000419103	170690/6356/1909/2138/2303/2736/2737/26585/255743/6423/3371/7472/7490	13
BP	GO:0045785	positive regulation of cell adhesion	23/408	388/17913	3.08E-05	0.000583572	0.000426339	857/151887/1012/1307/1295/6387/10085/2316/2335/2303/2736/2737/3037/3678/55083/3956/91663/255743/10630/6423/7042/7070/7168	23
BP	GO:0044272	sulfur compound biosynthetic process	15/408	190/17913	3.13E-05	0.000590589	0.000431466	633/23743/4166/1464/1634/29940/92126/2331/2687/4060/4958/9060/5549/1462/64131	15

© 2020 Yu Y et al. *JAMA Network Open.*

BP	GO:0085029	extracellular matrix assembly	6/408	28/17913	3.31E-05	0.000622481	0.000454765	84168/1278/11117/2621/3037/4015	6
BP	GO:0072210	metanephric nephron development	7/408	41/17913	3.48E-05	0.000650169	0.000474993	9370/26585/55083/3976/5156/5159/7490	7
BP	GO:0003206	cardiac chamber morphogenesis	12/408	129/17913	3.94E-05	0.000732222	0.000534938	1301/1363/2303/2627/2702/9464/8828/6423/7042/7168/7472/23414	12
BP	GO:0090330	regulation of platelet aggregation	5/408	18/17913	4.01E-05	0.000742385	0.000542363	170692/114897/10630/5592/5270	5
BP	GO:0048732	gland development	24/408	422/17913	4.03E-05	0.000742537	0.000542474	220/857/6356/1588/9420/2260/2627/2736/2737/9464/3082/3488/8660/5156/5270/5176/6591/23213/55959/7042/7043/3371/7472/7490	24
BP	GO:0010863	positive regulation of phospholipase C activity	7/408	42/17913	4.09E-05	0.000743981	0.000543529	552/1909/2260/3357/4915/5156/5159	7
BP	GO:00	chondroitin sulfate proteoglycan metabolic process	7/408	42/179	4.09E-0	0.00074	0.00054	633/1464/1634/29940/92126/1462/64131	7

© 2020 Yu Y et al. *JAMA Network Open*.

	50654			13	5	3981	3529		
BP	GO:0050650	chondroitin sulfate proteoglycan biosynthetic process	6/408	29/17913	4.10E-05	0.000743981	0.000543529	633/1464/1634/29940/1462/64131	6
BP	GO:0010518	positive regulation of phospholipase activity	8/408	57/17913	4.21E-05	0.000756579	0.000552733	185/552/1909/2260/3357/4915/5156/5159	8
BP	GO:0035904	aorta development	8/408	57/17913	4.21E-05	0.000756579	0.000552733	11174/1281/2138/4015/4016/5159/144165/7042	8
BP	GO:0032102	negative regulation of response to external stimulus	21/408	343/17913	4.26E-05	0.000762369	0.000556962	170692/9370/114897/4345/1588/2191/26585/3082/5654/5156/90102/5328/5592/5627/5740/5054/5270/5176/6591/7057/7130	21
BP	GO:1903034	regulation of response to wounding	14/408	173/17913	4.36E-05	0.000776635	0.000567385	170692/114897/857/2191/2303/5156/10630/90102/5328/5592/5627/5054/5270/7057	14
BP	GO:0010543	regulation of platelet activation	6/408	30/17913	5.03E-05	0.000886666	0.000647765	170692/114897/5156/10630/5592/5270	6

© 2020 Yu Y et al. *JAMA Network Open.*

BP	GO:0060325	face morphogenesis	6/408	30/17913	5.03E-05	0.000886666	0.000647765	1277/83690/83716/4313/5156/7043	6
BP	GO:0071229	cellular response to acid chemical	15/408	199/17913	5.34E-05	0.000933823	0.000682221	552/1307/1277/1278/1281/1290/1291/2042/4313/4915/56034/5156/5176/3371/7472	15
BP	GO:0010544	negative regulation of platelet activation	5/408	19/17913	5.34E-05	0.000933823	0.000682221	170692/114897/5156/5592/5270	5
BP	GO:0010975	regulation of neuron projection development	25/408	458/17913	5.45E-05	0.000948881	0.000693222	1272/6387/1809/2042/2260/2335/3082/3956/4131/4915/10769/91584/5376/5587/5396/5789/6383/5176/6423/6695/11075/55714/7070/7431/22891	25
BP	GO:0010466	negative regulation of peptidase activity	16/408	223/17913	5.50E-05	0.000950827	0.000694644	3730/1293/1410/1470/1893/2621/3082/5627/5743/5054/6423/6695/7057/7076/7077/7078	16
BP	GO:0007229	integrin-mediated signaling pathway	10/408	94/17913	5.54E-05	0.000950827	0.000694644	1307/1281/10979/2316/22801/3678/3690/5587/7070/7076	10
BP	GO:00	artery morphogenesis	9/408	76/179	5.59E-0	0.00095	0.00069	1281/1311/2138/2303/2702/9464/5159/5396/7042	9

© 2020 Yu Y et al. *JAMA Network Open.*

	48844			13	5	0827	4644		
BP	GO:0030850	prostate gland development	7/408	44/17913	5.59E-05	0.0009508827	0.0006946644	1588/9420/2736/2737/5176/23213/3371	7
BP	GO:1900274	regulation of phospholipase C activity	7/408	44/17913	5.59E-05	0.0009508827	0.0006946644	552/1909/2260/3357/4915/5156/5159	7
BP	GO:0009612	response to mechanical stimulus	15/408	200/17913	5.66E-05	0.0009578848	0.0006997773	1301/1277/1281/6387/1634/2115/3776/4131/9379/63895/10631/5743/5270/7057/3371	15
BP	GO:0030509	BMP signaling pathway	12/408	134/17913	5.73E-05	0.0009608863	0.0007019776	658/1311/2200/11167/26585/5654/94031/4920/6423/6424/23213/23090	12
BP	GO:0034332	adherens junction organization	12/408	134/17913	5.73E-05	0.0009608863	0.0007019776	88/1009/1012/1000/1307/2042/10979/26585/90102/11228/7057/7070	12
BP	GO:0017015	regulation of transforming growth factor beta receptor signaling pathway	11/408	115/17913	6.38E-05	0.0010650512	0.0007780064	54829/857/11117/2200/5654/94031/4015/255743/56937/7043/7057	11

© 2020 Yu Y et al. *JAMA Network Open*.

BP	GO:0033628	regulation of cell adhesion mediated by integrin	7/408	45/17913	6.49E-05	0.00107983 2	0.00078889 1	1545/2303/5328/5054/6423/6591/7042	7
BP	GO:0007044	cell-substrate junction assembly	10/408	96/17913	6.64E-05	0.00109887	0.00080279 9	88/1307/2042/10979/2335/26585/3678/90102/7057/7070	10
BP	GO:0014032	neural crest cell development	8/408	61/17913	6.93E-05	0.00113722 4	0.00083081 9	1909/2335/2303/9464/3357/8828/6591/7291	8
BP	GO:0032835	glomerulus development	8/408	61/17913	6.93E-05	0.00113722 4	0.00083081 9	59/9370/2303/5156/5159/23213/55959/7490	8
BP	GO:0001101	response to acid chemical	20/408	330/17913	7.44E-05	0.00120959 6	0.00088369 2	9370/552/1307/1277/1278/1281/1290/1291/2042/3490/4313/4915/56034/5156/5159/5743/5176/6678/3371/7472	20
BP	GO:0050900	leukocyte migration	25/408	467/17913	7.45E-05	0.00120959 6	0.00088369 2	857/6356/4345/1277/1278/6387/1588/9420/1893/11117/2335/2621/26585/3678/3690/5627/5919/4920/7857/6383/5054/7042/7057/7060/7070	25
BP	GO:19	regulation of cellular response to transforming growth	11/408	117/17	7.47E-0	0.00120	0.00088	54829/857/11117/2200/5654/94031/4015/255743/56937/7043/7057	11

	038844	factor beta stimulus		913	5	9596	3692		
BP	GO:0060324	face development	7/408	46/17913	7.51E-05	0.0012160 3	0.0008851 59	220/1277/83690/83716/4313/5156/7043	7
BP	GO:0072028	nephron morphogenesis	9/408	79/17913	7.60E-05	0.0012212 07	0.0008921 75	170690/2138/2737/26585/55083/3976/255743/5159/7490	9
BP	GO:0001657	ureteric bud development	10/408	98/17913	7.92E-05	0.0012660 69	0.0009249 5	170690/9244/2138/2260/2303/2737/26585/55083/255743/7490	10
BP	GO:0003018	vascular process in circulatory system	13/408	161/17913	8.32E-05	0.0013250 16	0.0009680 15	59/185/9590/857/1311/1909/2303/2702/2982/3357/5139/5592/5743	13
BP	GO:0050731	positive regulation of peptidyl-tyrosine phosphorylation	14/408	184/17913	8.53E-05	0.0013522 52	0.0009879 12	9370/1272/9244/1464/2621/2690/26585/3082/3678/3690/3976/50507/4915/7060	14
BP	GO:0072163	mesonephric epithelium development	10/408	99/17913	8.63E-05	0.0013572 25	0.0009915 46	170690/9244/2138/2260/2303/2737/26585/55083/255743/7490	10

© 2020 Yu Y et al. *JAMA Network Open*.

BP	GO:0072164	mesonephric tubule development	10/408	99/17913	8.63E-05	0.001357225	0.000991546	170690/9244/2138/2260/2303/2737/26585/55083/255743/7490	10
BP	GO:0072073	kidney epithelium development	12/408	140/17913	8.77E-05	0.001373146	0.001003177	170690/9370/9244/2138/2260/2303/2737/26585/55083/3976/255743/7490	12
BP	GO:0046849	bone remodeling	9/408	81/17913	9.26E-05	0.001444293	0.001055154	115908/1513/26585/3690/3953/4286/50507/5745/7043	9
BP	GO:0010171	body morphogenesis	7/408	48/17913	9.93E-05	0.00154339	0.001127551	1277/83690/83716/4313/5156/90102/7043	7
BP	GO:0090101	negative regulation of transmembrane receptor protein serine/threonine kinase signaling pathway	11/408	121/17913	0.000101306	0.001567331	0.001145042	54829/857/8483/11117/2200/26585/5654/94031/56937/6423/7043	11
BP	GO:0018149	peptide cross-linking	6/408	34/17913	0.000105415	0.001624244	0.001186621	633/1281/1634/2162/2335/7057	6
BP	GO:00	mesenchymal cell development	8/408	65/179	0.00010	0.00167	0.00122	1909/2335/2303/9464/3357/8828/6591/7291	8

© 2020 Yu Y et al. *JAMA Network Open*.

	140031			13	9713	0022	0065		
BP	GO:0035924	cellular response to vascular endothelial growth factor stimulus	8/408	65/17913	0.000109713	0.001670022	0.001220065	81792/1634/2619/57608/8828/5156/5159/5587	8
BP	GO:0048864	stem cell development	8/408	65/17913	0.000109713	0.001670022	0.001220065	1909/2335/2303/9464/3357/8828/6591/7291	8
BP	GO:0042340	keratan sulfate catabolic process	4/408	12/17913	0.000113554	0.001714662	0.001252677	2331/4060/4958/5549	4
BP	GO:0090331	negative regulation of platelet aggregation	4/408	12/17913	0.000113554	0.001714662	0.001252677	170692/114897/5592/5270	4
BP	GO:0032970	regulation of actin filament-based process	20/408	341/17913	0.000116216	0.001747867	0.001276936	88/857/6356/6387/2042/80206/2316/122786/2702/91663/50507/5156/5159/10630/90102/130271/5999/6781/7043/7168	20
BP	GO:0051146	striated muscle cell differentiation	17/408	263/17913	0.00011813	0.001769603	0.001292816	88/8038/552/1000/1311/80206/2627/26585/3488/4015/50507/5156/5159/5999/6444/7168/7490	17

© 2020 Yu Y et al. *JAMA Network Open.*

BP	GO:0001823	mesonephros development	10/408	103/17913	0.000120548	0.00179863	0.00131406	170690/9244/2138/2260/2303/2737/26585/55083/255743/7490	10
BP	GO:0010517	regulation of phospholipase activity	8/408	66/17913	0.000122399	0.00181916	0.00132898	185/552/1909/2260/3357/4915/5156/5159	8
BP	GO:0048333	mesodermal cell differentiation	6/408	35/17913	0.000124803	0.0018403 5	0.00134450 1	2138/2260/2303/3624/3690/6423	6
BP	GO:0060323	head morphogenesis	6/408	35/17913	0.000124803	0.00184035	0.00134501	1277/83690/83716/4313/5156/7043	6
BP	GO:1901343	negative regulation of vasculature development	14/408	191/17913	0.000126948	0.00186470 8	0.00136229 6	90993/1634/11117/4223/10769/5054/5176/6678/23213/7042/7057/7058/7060/7490	14
BP	GO:0010718	positive regulation of epithelial to mesenchymal transition	7/408	50/17913	0.000129609	0.00188182 1	0.00137479 8	8313/1277/4017/10630/7042/7043/7291	7
BP	GO:00	coronary vasculature development	7/408	50/179	0.00012	0.00188	0.00137	11174/2627/9464/5125/5159/144165/6444	7

© 2020 Yu Y et al. *JAMA Network Open*.

	60976			13	9609	1821	4798		
BP	GO:0086009	membrane repolarization	7/408	50/17913	0.000129609	0.0018821	0.00137 4798	781/857/2316/2702/23704/3764/6330	7
BP	GO:0042475	odontogenesis of dentin-containing tooth	9/408	85/17913	0.0001348 95	0.0019510 68	0.0014253 88	56975/2736/2737/9464/5654/9622/5156/5054/3371	9
BP	GO:0097529	myeloid leukocyte migration	13/408	170/17913	0.0001442 01	0.0020777	0.0015179 02	6356/4345/6387/1588/11117/26585/5919/4920/7857/5054/7042/7057/7060	13
BP	GO:0003156	regulation of animal organ formation	6/408	36/17913	0.0001469 13	0.0021065 9	0.0015390 08	2138/2260/9464/23213/7472/7490	6
BP	GO:0048146	positive regulation of fibroblast proliferation	7/408	51/17913	0.0001473 22	0.0021065 9	0.0015390 08	4921/2335/2621/56034/5156/5159/7472	7
BP	GO:0060038	cardiac muscle cell proliferation	8/408	68/17913	0.0001514 16	0.0021569 68	0.0015758 12	2260/2303/2627/3776/26011/7042/7472/23414	8

© 2020 Yu Y et al. *JAMA Network Open.*

BP	GO:0043200	response to amino acid	10/408	106/17913	0.000153229	0.002174584	0.001588682	1307/1277/1278/1281/1290/1291/4313/4915/56034/5156	10
BP	GO:0016485	protein processing	18/408	295/17913	0.000155226	0.002194682	0.001603365	9509/715/716/1066/3075/1311/10699/2619/2737/85409/5122/5125/5328/5627/5054/5270/10418/7057	18
BP	GO:0045995	regulation of embryonic development	11/408	128/17913	0.000167562	0.002360245	0.00172432	9370/1289/1290/2260/91663/90102/6423/23213/9096/26011/7472	11
BP	GO:0045861	negative regulation of proteolysis	19/408	324/17913	0.000171628	0.002400466	0.001753704	3730/1293/1410/1470/1893/2619/2621/3082/4130/5627/5743/5054/5270/6423/6695/7057/7076/7077/7078	19
BP	GO:0010634	positive regulation of epithelial cell migration	13/408	173/17913	0.000171689	0.002400466	0.001753704	2260/2303/3037/3690/57608/8828/10769/5587/5743/6678/27286/7042/7057	13
BP	GO:0072012	glomerulus vasculature development	5/408	24/17913	0.000177811	0.002476891	0.001809538	59/2303/5156/5159/7490	5
BP	GO:00	positive regulation of endothelial cell migration	11/408	129/17	0.00017	0.00249	0.00181	2260/2303/3690/57608/8828/10769/5587/5743/6678/27286/7057	11

	10595			913	9511	0349	937		
BP	GO:2001236	regulation of extrinsic apoptotic signaling pathway	12/408	151/17913	0.000180097	0.002490349	0.001819937	658/857/2260/3082/3624/7857/5054/6423/6591/8406/7057/7078	12
BP	GO:0014033	neural crest cell differentiation	8/408	70/17913	0.000185876	0.002551566	0.001864093	1909/2335/2303/9464/3357/8828/6591/7291	8
BP	GO:0060193	positive regulation of lipase activity	8/408	70/17913	0.000185876	0.002551566	0.001864093	185/552/1909/2260/3357/4915/5156/5159	8
BP	GO:0050921	positive regulation of chemotaxis	11/408	130/17913	0.000192176	0.002628494	0.001920294	1012/6387/2260/2621/5159/5587/5919/7857/5054/7057/7060	11
BP	GO:0072009	nephron epithelium development	10/408	109/17913	0.000193077	0.002631287	0.001922335	170690/9370/2138/2303/2737/26585/55083/3976/255743/7490	10
BP	GO:0042310	vasoconstriction	8/408	71/17913	0.000205379	0.002771789	0.002024981	59/185/857/1311/1909/2702/3357/5743	8

© 2020 Yu Y et al. *JAMA Network Open.*

BP	GO:0003231	cardiac ventricle development	11/408	131/17913	0.000020559	0.002771789	0.002024981	1301/1363/2303/2702/9464/3776/57167/6423/7042/7168/23414	11
BP	GO:0007224	smoothened signaling pathway	11/408	131/17913	0.000020559	0.002771789	0.002024981	22873/5167/2121/132884/2619/2736/2737/84662/5396/4920/5270	11
BP	GO:0048562	embryonic organ morphogenesis	17/408	276/17913	0.00002103355	0.00282594	0.00206454 1	220/1301/115908/2202/2138/2200/2260/2303/2736/2737/9464/4237/5156/5396/4920/7043/7291	17
BP	GO:0070206	protein trimerization	7/408	54/17913	0.0000212536	0.0028451 24	0.0020785 57	9370/114897/1277/1278/1291/1292/11117	7
BP	GO:0016525	negative regulation of angiogenesis	13/408	177/17913	0.000021526	0.002871398	0.002097752	90993/1634/11117/4223/10769/5054/5176/6678/23213/7042/7057/7058/7060	13
BP	GO:0042730	fibrinolysis	5/408	25/17913	0.0000218137	0.0028995 32	0.0021183 06	2191/5328/5627/5054/7057	5
BP	GO:00	chondroitin sulfate catabolic process	4/408	14/179	0.000022	0.00292	0.00213	633/1464/1634/1462	4

© 2020 Yu Y et al. *JAMA Network Open*.

	30207			13	1468	3225	5615		
BP	GO:0072216	positive regulation of metanephros development	4/408	14/17913	0.000221468	0.002923225	0.002135615	9370/3976/5159/7490	4
BP	GO:0042692	muscle cell differentiation	20/408	359/17913	0.000230351	0.003029885	0.002213537	88/8038/552/1000/1311/80206/2627/26585/3488/4015/50507/255743/93145/5156/5159/5999/6444/23345/7168/7490	20
BP	GO:0050880	regulation of blood vessel size	11/408	133/17913	0.000234807	0.003077759	0.002248513	59/185/857/1311/1909/2303/2702/2982/3357/5592/5743	11
BP	GO:2000181	negative regulation of blood vessel morphogenesis	13/408	179/17913	0.000240386	0.003139996	0.002293981	90993/1634/11117/4223/10769/5054/5176/6678/23213/7042/7057/7058/7060	13
BP	GO:0060419	heart growth	10/408	112/17913	0.000241294	0.003140987	0.002294705	2260/2303/2627/3776/5999/26011/7042/7472/7490/23414	10
BP	GO:0070482	response to oxygen levels	21/408	389/17913	0.000248077	0.003218182	0.002351101	9370/857/1277/1410/6387/1909/2627/3776/4017/50507/5159/5328/5352/10631/5740/5743/6781/7042/7043/7057/7291	21

© 2020 Yu Y et al. *JAMA Network Open*.

BP	GO:0003208	cardiac ventricle morphogenesis	8/408	73/17913	0.000249434	0.003224708	0.002355869	1301/1363/2303/9464/6423/7042/7168/23414	8
BP	GO:0035150	regulation of tube size	11/408	134/17913	0.000250684	0.0032298	0.002359589	59/185/857/1311/1909/2303/2702/2982/3357/5592/5743	11
BP	GO:0061437	renal system vasculature development	5/408	26/17913	0.000265062	0.003369057	0.002461326	59/2303/5156/5159/7490	5
BP	GO:0061440	kidney vasculature development	5/408	26/17913	0.000265062	0.003369057	0.002461326	59/2303/5156/5159/7490	5
BP	GO:0070168	negative regulation of biomineral tissue development	5/408	26/17913	0.000265062	0.003369057	0.002461326	54829/1893/5167/2621/26585	5
BP	GO:0072273	metanephric nephron morphogenesis	5/408	26/17913	0.000265062	0.003369057	0.002461326	26585/55083/3976/5159/7490	5
BP	GO:00	cardiac muscle cell action potential involved in contraction	7/408	56/179	0.00026	0.00337	0.00246	781/857/2316/2702/23704/3764/6330	7

© 2020 Yu Y et al. *JAMA Network Open*.

	86002			13	7671	3824	4808		
BP	GO:0002040	sprouting angiogenesis	13/408	181/17913	0.000267982	0.003373824	0.002464808	185/1012/90993/2303/26585/3678/57608/4017/4223/10769/5743/27286/7057	13
BP	GO:0038084	vascular endothelial growth factor signaling pathway	6/408	40/17913	0.000268118	0.003373824	0.002464808	1634/57608/8828/5156/5159/5587	6
BP	GO:0010594	regulation of endothelial cell migration	15/408	231/17913	0.000279588	0.003506463	0.00256171	1634/2260/2303/3690/57608/4223/8828/10769/5587/5743/5176/6678/27286/6781/7057	15
BP	GO:0060191	regulation of lipase activity	9/408	94/17913	0.000291197	0.003639963	0.002659241	185/552/1909/2260/3357/4915/5125/5156/5159	9
BP	GO:0034111	negative regulation of homotypic cell-cell adhesion	4/408	15/17913	0.000296592	0.003683007	0.002690688	170692/114897/5592/5270	4
BP	GO:0072109	glomerular mesangium development	4/408	15/17913	0.000296592	0.003683007	0.002690688	59/2303/5159/7490	4

© 2020 Yu Y et al. *JAMA Network Open.*

BP	GO:0098742	cell-cell adhesion via plasma-membrane adhesion molecules	15/408	233/17913	0.000306623	0.003795092	0.002772573	9370/1012/1000/64084/120114/2239/3678/91663/5099/5101/5789/91179/55714/7042/7216	15
BP	GO:0048015	phosphatidylinositol-mediated signaling	12/408	160/17913	0.000308318	0.003803594	0.002778784	1634/2260/3082/3357/8660/4883/4915/5156/5159/5270/7042/7291	12
BP	GO:0033273	response to vitamin	9/408	95/17913	0.000315322	0.003864747	0.002823461	1277/2621/4131/10631/5743/6591/6678/6781/3371	9
BP	GO:0060993	kidney morphogenesis	9/408	95/17913	0.000315322	0.003864747	0.002823461	170690/2138/2737/26585/55083/3976/255743/5159/7490	9
BP	GO:1902903	regulation of supramolecular fiber organization	18/408	313/17913	0.000320419	0.003914499	0.002859809	88/165/6356/1410/6387/11117/80206/2316/4131/91663/89795/50507/90102/130271/5999/11075/7043/7168	18
BP	GO:0014065	phosphatidylinositol 3-kinase signaling	11/408	138/17913	0.000323573	0.003940286	0.002878647	1634/2260/3082/3357/8660/4915/5156/5159/5270/7042/7291	11
BP	GO:00	negative regulation of endopeptidase activity	14/408	210/17	0.00033	0.00412	0.00300	3730/1293/1410/1470/2621/3082/5627/5743/5054/6423/6695/7057/7076/7078	14

	10951			913	9426	0038	9968		
BP	GO:0001952	regulation of cell-matrix adhesion	10/408	117/17913	0.000343395	0.004148284	0.003030604	1012/1307/2042/26585/90102/5328/10631/5054/7057/7070	10
BP	GO:0014066	regulation of phosphatidylinositol 3-kinase signaling	10/408	117/17913	0.000343395	0.004148284	0.003030604	1634/2260/3082/8660/4915/5156/5159/5270/7042/7291	10
BP	GO:0099622	cardiac muscle cell membrane repolarization	6/408	42/17913	0.000352647	0.004239663	0.003097339	781/2316/2702/23704/3764/6330	6
BP	GO:0030279	negative regulation of ossification	8/408	77/17913	0.000360812	0.004310339	0.003148996	8313/1893/5167/26585/9464/3488/63923/7291	8
BP	GO:0072088	nephron epithelium morphogenesis	8/408	77/17913	0.000360812	0.004310339	0.003148996	170690/2138/2737/26585/55083/3976/255743/7490	8
BP	GO:0048017	inositol lipid-mediated signaling	12/408	163/17913	0.000365434	0.004351773	0.003179267	1634/2260/3082/3357/8660/4883/4915/5156/5159/5270/7042/7291	12

© 2020 Yu Y et al. *JAMA Network Open.*

BP	GO:0018146	keratan sulfate biosynthetic process	5/408	28/17913	0.000381471	0.004528471	0.003308356	4166/2331/4060/4958/5549	5
BP	GO:0045165	cell fate commitment	15/408	238/17913	0.000384256	0.004547232	0.003322063	2138/2260/2303/2619/2627/2736/2737/4286/10630/5396/4920/6423/26011/7490/25925	15
BP	GO:0007568	aging	18/408	318/17913	0.000387422	0.004553175	0.003326405	1311/1410/1634/3488/4017/50507/9891/5159/10769/5549/5743/5745/5054/5176/7043/7076/7077/7291	18
BP	GO:0010934	macrophage cytokine production	4/408	16/17913	0.000388377	0.004553175	0.003326405	2621/10417/7042/7043	4
BP	GO:0072224	metanephric glomerulus development	4/408	16/17913	0.000388377	0.004553175	0.003326405	9370/5156/5159/7490	4
BP	GO:0008589	regulation of smoothened signaling pathway	8/408	78/17913	0.000394194	0.004592848	0.003355389	5167/2121/2619/2736/2737/84662/5396/5270	8
BP	GO:00	negative regulation of transforming growth factor beta receptor	8/408	78/179	0.00039	0.00459	0.00335	54829/857/11117/2200/5654/94031/56937/7043	8

	3051 2	signaling pathway		13	4194	2848	5389		
BP	GO:0003044	regulation of systemic arterial blood pressure mediated by a chemical signal	6/408	43/17913	0.000402058	0.004670055	0.003411793	185/1066/10699/2702/5125/7168	6
BP	GO:0034329	cell junction assembly	14/408	214/17913	0.000410759	0.004756483	0.003474935	88/857/1307/2042/10979/2316/2335/2702/26585/3678/90102/6591/7057/7070	14
BP	GO:0097756	negative regulation of blood vessel diameter	8/408	79/17913	0.000430045	0.004964581	0.003626965	59/185/857/1311/1909/2702/3357/5743	8
BP	GO:0010632	regulation of epithelial cell migration	17/408	294/17913	0.000438229	0.005043639	0.003684722	1634/2260/2303/3037/3690/57608/4223/8828/10769/5587/5743/5176/6678/27286/6781/7042/7057	17
BP	GO:0055007	cardiac muscle cell differentiation	10/408	121/17913	0.000450251	0.005166252	0.003774299	88/80206/2627/26585/50507/5156/5159/5999/6444/7490	10
BP	GO:0035850	epithelial cell differentiation involved in kidney development	6/408	44/17913	0.000456712	0.005224504	0.003816856	59/9370/2303/26585/3976/7490	6

© 2020 Yu Y et al. *JAMA Network Open*.

BP	GO:0043271	negative regulation of ion transport	11/408	144/17913	0.000465849	0.005279344	0.003856692	88/857/2669/8660/5743/5999/5270/6424/6781/7057/7291	11
BP	GO:0043536	positive regulation of blood vessel endothelial cell migration	8/408	80/17913	0.000468498	0.005279344	0.003856692	2260/2303/57608/10769/5587/5743/27286/7057	8
BP	GO:0050886	endocrine process	8/408	80/17913	0.000468498	0.005279344	0.003856692	185/114897/1066/10699/2260/2702/3624/5125	8
BP	GO:0110110	positive regulation of animal organ morphogenesis	8/408	80/17913	0.000468498	0.005279344	0.003856692	2260/26585/9464/3976/7042/7291/7472/7490	8
BP	GO:1903845	negative regulation of cellular response to transforming growth factor beta stimulus	8/408	80/17913	0.000468498	0.005279344	0.003856692	54829/857/11117/2200/5654/94031/56937/7043	8
BP	GO:0071774	response to fibroblast growth factor	11/408	145/17913	0.000494015	0.005550322	0.004054889	3730/1277/90993/56975/2260/23768/10631/23213/55959/7057/3371	11
BP	GO:00	chondrocyte proliferation	4/408	17/179	0.000049	0.00555	0.00405	658/1311/4921/6781	4

© 2020 Yu Y et al. *JAMA Network Open*.

	35988			13	8793	4405	7871		
BP	GO:0040037	negative regulation of fibroblast growth factor receptor signaling pathway	4/408	17/17913	0.000498793	0.005554405	0.004057871	90993/23213/55959/7057	4
BP	GO:2000811	negative regulation of anoikis	4/408	17/17913	0.000498793	0.005554405	0.004057871	857/3678/4915/6591	4
BP	GO:0007045	cell-substrate adherens junction assembly	8/408	81/17913	0.000509694	0.005609603	0.004098197	88/1307/2042/10979/26585/90102/7057/7070	8
BP	GO:0014068	positive regulation of phosphatidylinositol 3-kinase signaling	8/408	81/17913	0.000509694	0.005609603	0.004098197	1634/2260/3082/8660/4915/5156/5159/7042	8
BP	GO:0030510	regulation of BMP signaling pathway	8/408	81/17913	0.000509694	0.005609603	0.004098197	2200/26585/5654/94031/6423/6424/23213/23090	8
BP	GO:0048041	focal adhesion assembly	8/408	81/17913	0.000509694	0.005609603	0.004098197	88/1307/2042/10979/26585/90102/7057/7070	8

© 2020 Yu Y et al. *JAMA Network Open*.

BP	GO:0022602	ovulation cycle process	6/408	45/17913	0.000516985	0.005673312	0.004144741	658/3624/4327/5156/7042/7043	6
BP	GO:0001666	response to hypoxia	19/408	354/17913	0.000519518	0.005684584	0.004152976	9370/857/1410/6387/1909/2627/3776/4017/50507/5328/5352/10631/5740/5743/6781/7042/7043/7057/7291	19
BP	GO:0016331	morphogenesis of embryonic epithelium	11/408	146/17913	0.000523593	0.005712608	0.004173449	220/115908/2736/26585/144165/57167/6423/23213/7042/7291/7472	11
BP	GO:0014855	striated muscle cell proliferation	8/408	82/17913	0.000553775	0.006007183	0.004388657	2260/2303/2627/3776/26011/7042/7472/23414	8
BP	GO:0048644	muscle organ morphogenesis	8/408	82/17913	0.000553775	0.006007183	0.004388657	1301/1281/2303/3976/7042/7168/7472/23414	8
BP	GO:0035296	regulation of tube diameter	10/408	125/17913	0.000582517	0.006282864	0.00459006	59/185/857/1311/1909/2702/2982/3357/5592/5743	10
BP	GO:00	regulation of blood vessel diameter	10/408	125/17	0.00058	0.00628	0.00459	59/185/857/1311/1909/2702/2982/3357/5592/5743	10

© 2020 Yu Y et al. *JAMA Network Open*.

	97746			913	2517	2864	006		
BP	GO:0044319	wound healing, spreading of cells	5/408	31/17913	0.000623543	0.006649359	0.00485781	1289/2316/3678/10630/90102	5
BP	GO:0086011	membrane repolarization during action potential	5/408	31/17913	0.000623543	0.006649359	0.00485781	781/857/2316/23704/3764	5
BP	GO:0090505	epiboly involved in wound healing	5/408	31/17913	0.000623543	0.006649359	0.00485781	1289/2316/3678/10630/90102	5
BP	GO:0099625	ventricular cardiac muscle cell membrane repolarization	5/408	31/17913	0.000623543	0.006649359	0.00485781	781/2702/23704/3764/6330	5
BP	GO:0090257	regulation of muscle system process	15/408	251/17913	0.000669234	0.007116502	0.00519909	857/114907/2316/2702/2982/9464/3488/29995/10398/255743/5592/5743/5999/6781/7168	15
BP	GO:1904888	cranial skeletal system development	7/408	65/17913	0.000674353	0.007150792	0.005224141	2303/2737/5156/5396/7042/7043/7291	7

© 2020 Yu Y et al. *JAMA Network Open.*

BP	GO:0048704	embryonic skeletal system morphogenesis	8/408	85/17913	0.000704829	0.007453023	0.005444941	1301/2138/2303/2737/5156/5396/7043/7291	8
BP	GO:0055017	cardiac muscle tissue growth	9/408	106/17913	0.000707958	0.007465198	0.005453836	2260/2303/2627/3776/5999/26011/7042/7472/23414	9
BP	GO:0040036	regulation of fibroblast growth factor receptor signaling pathway	5/408	32/17913	0.000725344	0.007564019	0.005526031	90993/56975/23213/55959/7057	5
BP	GO:0060317	cardiac epithelial to mesenchymal transition	5/408	32/17913	0.000725344	0.007564019	0.005526031	3037/6591/7042/7291/7472	5
BP	GO:0071295	cellular response to vitamin	5/408	32/17913	0.000725344	0.007564019	0.005526031	1277/2621/10631/6591/3371	5
BP	GO:0090504	epiboly	5/408	32/17913	0.000725344	0.007564019	0.005526031	1289/2316/3678/10630/90102	5
BP	GO:19	carbohydrate derivative catabolic process	12/408	176/17	0.00072	0.00756	0.00552	81792/633/1464/1634/2331/2239/10082/4060/4958/5549/6383/1462	12

© 2020 Yu Y et al. *JAMA Network Open*.

	01136			913	7402	4576	6438		
BP	GO:1901861	regulation of muscle tissue development	11/408	152/17913	0.0007338 47	0.0076066 32	0.0055571 63	2260/2627/26585/3488/57158/3776/4015/5999/7291/7472/23414	11
BP	GO:0060389	pathway-restricted SMAD protein phosphorylation	6/408	48/17913	0.0007354 76	0.0076066 32	0.0055571 63	11117/26585/3624/56937/7042/7043	6
BP	GO:0060675	ureteric bud morphogenesis	7/408	66/17913	0.0007398 39	0.0076100 6	0.0055596 67	170690/2138/2737/26585/55083/255743/7490	7
BP	GO:1903672	positive regulation of sprouting angiogenesis	7/408	66/17913	0.0007398 39	0.0076100 6	0.0055596 67	185/2303/3678/57608/10769/5743/27286	7
BP	GO:0036293	response to decreased oxygen levels	19/408	365/17913	0.0007513 15	0.0077071 04	0.0056305 65	9370/857/1410/6387/1909/2627/3776/4017/50507/5328/5352/10631/5740/5743/6781/7042/7043/7057/7291	19
BP	GO:0001710	mesodermal cell fate commitment	4/408	19/17913	0.0007835 52	0.0079728 03	0.0058246 76	2138/2260/2303/6423	4

BP	GO:0032332	positive regulation of chondrocyte differentiation	4/408	19/17913	0.000783552	0.007972803	0.005824676	658/2737/4017/91461	4
BP	GO:0060973	cell migration involved in heart development	4/408	19/17913	0.000783552	0.007972803	0.005824676	9464/5159/6591/7291	4
BP	GO:0046683	response to organophosphorus	10/408	130/17913	0.000791512	0.008032141	0.005868027	9370/1277/3488/4327/50507/5139/5743/6678/6781/7490	10
BP	GO:0050769	positive regulation of neurogenesis	22/408	455/17913	0.000795044	0.008046353	0.005878409	1272/6387/1809/2042/2260/2316/2335/2736/2737/3082/3976/4131/4915/5587/5789/5270/5176/11075/55714/26011/7077/22891	22
BP	GO:0010812	negative regulation of cell-substrate adhesion	7/408	67/17913	0.000810255	0.008156568	0.005958929	1277/3956/90102/10631/5054/6695/7057	7
BP	GO:0072171	mesonephric tubule morphogenesis	7/408	67/17913	0.000810255	0.008156568	0.005958929	170690/2138/2737/26585/55083/255743/7490	7
BP	GO:00	regulation of collagen metabolic process	6/408	49/179	0.00082	0.00823	0.00601	90993/11117/2191/5159/7043/7431	6

© 2020 Yu Y et al. *JAMA Network Open*.

	10712			13	224	3307	4992		
BP	GO:2001238	positive regulation of extrinsic apoptotic signaling pathway	6/408	49/17913	0.000822224	0.008233307	0.006014992	658/857/3624/8406/7057/7078	6
BP	GO:0003338	metanephros morphogenesis	5/408	33/17913	0.000839067	0.008322191	0.006079927	26585/55083/3976/5159/7490	5
BP	GO:0042339	keratan sulfate metabolic process	5/408	33/17913	0.000839067	0.008322191	0.006079927	4166/2331/4060/4958/5549	5
BP	GO:0048048	embryonic eye morphogenesis	5/408	33/17913	0.000839067	0.008322191	0.006079927	220/2202/2200/4237/7291	5
BP	GO:0045598	regulation of fat cell differentiation	10/408	131/17913	0.000839935	0.008322191	0.006079927	9370/5167/84935/4320/5743/5919/862/6423/6591/23414	10
BP	GO:0003015	heart process	16/408	284/17913	0.000845689	0.008357268	0.006105554	10060/552/781/857/10699/2316/2702/23704/3764/50507/5999/6330/6444/6781/7042/7168	16

BP	GO:0052547	regulation of peptidase activity	20/408	398/17913	0.000851884	0.008396509	0.006134222	3730/84168/857/1293/1410/1470/1893/2335/2621/3082/5118/5627/5743/5054/6423/6695/7057/7076/7077/7078	20
BP	GO:1903524	positive regulation of blood circulation	7/408	68/17913	0.000885866	0.008708645	0.006362259	552/857/2702/5743/5999/7042/7168	7
BP	GO:0060048	cardiac muscle contraction	10/408	132/17913	0.000890767	0.008734141	0.006380885	781/857/2316/2702/23704/3764/6330/6444/6781/7168	10
BP	GO:1905517	macrophage migration	6/408	50/17913	0.000916669	0.008965037	0.006549571	4345/1588/11117/5919/4920/7057	6
BP	GO:1901652	response to peptide	23/408	491/17913	0.000933395	0.009104823	0.006651693	88/9370/185/773/781/857/1277/6387/1759/5167/2200/2303/2690/3488/8660/4131/4313/4314/5743/5919/6678/7076/7431	23
BP	GO:0010002	cardioblast differentiation	4/408	20/17913	0.000961961	0.009322211	0.006810051	2627/26585/144165/7042	4
BP	GO:00	positive regulation of transforming growth factor beta production	4/408	20/179	0.00096	0.00932	0.00681	4345/4060/5743/7057	4

© 2020 Yu Y et al. *JAMA Network Open*.

	71636			13	1961	2211	051		
BP	GO:0007435	salivary gland morphogenesis	5/408	34/17913	0.000965559	0.00932211	0.00681051	2260/3082/6591/7042/7043	5
BP	GO:0060795	cell fate commitment involved in formation of primary germ layer	5/408	34/17913	0.000965559	0.00932211	0.00681051	2138/2260/2303/2627/6423	5
BP	GO:0045668	negative regulation of osteoblast differentiation	6/408	51/17913	0.001019272	0.00979072	0.00715288	8313/26585/9464/3488/63923/7291	6
BP	GO:0072132	mesenchyme morphogenesis	6/408	51/17913	0.001019272	0.00979072	0.00715288	59/2260/2303/6591/7042/7291	6
BP	GO:0034333	adherens junction assembly	8/408	90/17913	0.001028937	0.00983508	0.007184047	88/1307/2042/10979/26585/90102/7057/7070	8
BP	GO:1901888	regulation of cell junction assembly	8/408	90/17913	0.001028937	0.00983508	0.007184047	857/1307/2042/26585/90102/6591/7057/7070	8

© 2020 Yu Y et al. *JAMA Network Open.*

BP	GO:0006790	sulfur compound metabolic process	18/408	346/17913	0.001033449	0.009851689	0.00719733	633/23743/4166/1464/1634/29940/92126/5167/2331/2687/2690/4060/50507/4958/9060/5549/1462/64131	18
BP	GO:0007601	visual perception	13/408	209/17913	0.001043871	0.009925978	0.007251603	1301/1277/1545/2202/2070/2743/83872/4060/4942/93145/25769/7045/7078	13
BP	GO:0001818	negative regulation of cytokine production	16/408	290/17913	0.001054143	0.009998467	0.007304561	9370/558/4345/2260/2335/2621/2627/3082/3624/2615/89795/284297/7042/7043/7057/7291	16
BP	GO:0050954	sensory perception of mechanical stimulus	11/408	159/17913	0.001063373	0.010060731	0.007350049	1301/1277/6387/2138/2070/2260/125336/63895/4919/5270/6591	11
BP	GO:0045923	positive regulation of fatty acid metabolic process	5/408	35/17913	0.001105685	0.010408879	0.007604395	9370/552/8660/5743/7291	5
BP	GO:0110111	negative regulation of animal organ morphogenesis	5/408	35/17913	0.001105685	0.010408879	0.007604395	54829/2303/26585/23213/7042	5
BP	GO:00	stem cell differentiation	14/408	237/17	0.00111	0.01050	0.00767	1909/2335/2303/2627/26585/9464/3357/3976/8828/5156/144165/6591/7042/7291	14

© 2020 Yu Y et al. *JAMA Network Open.*

	48863			913	8956	7609	6525		
BP	GO:2000242	negative regulation of reproductive process	6/408	52/17913	0.0011304445	0.0105891 59	0.0077361 02	3976/5176/23213/7076/7490/23414	6
BP	GO:0003094	glomerular filtration	4/408	21/17913	0.0011671 15	0.0107986 76	0.0078891 69	2621/2702/23213/55959	4
BP	GO:0030728	ovulation	4/408	21/17913	0.0011671 15	0.0107986 76	0.0078891 69	3624/4327/5743/7130	4
BP	GO:0071711	basement membrane organization	4/408	21/17913	0.0011671 15	0.0107986 76	0.0078891 69	23768/4320/22795/90102	4
BP	GO:1903010	regulation of bone development	4/408	21/17913	0.0011671 15	0.0107986 76	0.0078891 69	2200/2737/26585/4015	4
BP	GO:1903055	positive regulation of extracellular matrix organization	4/408	21/17913	0.0011671 15	0.0107986 76	0.0078891 69	4921/11117/10630/90102	4

BP	GO:0002685	regulation of leukocyte migration	12/408	186/17913	0.001177513	0.0108682 46	0.0079399 94	4345/6387/1588/1893/11117/2621/26585/5919/5054/7057/7060/7070	12
BP	GO:0050953	sensory perception of light stimulus	13/408	213/17913	0.0012403 17	0.0113568 74	0.0082969 7	1301/1277/1545/2202/2070/2743/83872/4060/4942/93145/25769/7045/7078	13
BP	GO:0097191	extrinsic apoptotic signaling pathway	13/408	213/17913	0.0012403 17	0.0113568 74	0.0082969 7	658/857/2260/3082/3624/7857/5054/6423/6591/8406/7042/7057/7078	13
BP	GO:0032836	glomerular basement membrane development	3/408	10/17913	0.0012494 06	0.0113568 74	0.0082969 7	23213/55959/7490	3
BP	GO:0033629	negative regulation of cell adhesion mediated by integrin	3/408	10/17913	0.0012494 06	0.0113568 74	0.0082969 7	1545/5054/6591	3
BP	GO:0060346	bone trabecula formation	3/408	10/17913	0.0012494 06	0.0113568 74	0.0082969 7	1277/26585/4313	3
BP	GO:00	cartilage morphogenesis	3/408	10/179	0.00124	0.01135	0.00829	9464/6591/6781	3

© 2020 Yu Y et al. *JAMA Network Open*.

	60536			13	9406	6874	697		
BP	GO:0044344	cellular response to fibroblast growth factor stimulus	10/408	138/17913	0.00125151 2	0.01135687 4	0.00829697	3730/1277/90993/56975/2260/23768/10631/23213/55959/7057	10
BP	GO:1905521	regulation of macrophage migration	5/408	36/17913	0.00126032 5	0.01140942 1	0.00833535 9	4345/1588/11117/5919/7057	5
BP	GO:0042471	ear morphogenesis	9/408	115/17913	0.00126790 6	0.01142770 3	0.00834871 6	220/1301/115908/2138/2260/2736/5396/4920/7291	9
BP	GO:0048010	vascular endothelial growth factor receptor signaling pathway	8/408	93/17913	0.00127445 4	0.01142770 3	0.00834871 6	558/11117/2303/3678/3690/8828/5587/23213	8
BP	GO:0051591	response to cAMP	8/408	93/17913	0.00127445 4	0.01142770 3	0.00834871 6	9370/1277/3488/4327/50507/6678/6781/7490	8
BP	GO:0072080	nephron tubule development	8/408	93/17913	0.00127445 4	0.01142770 3	0.00834871 6	170690/2138/2737/26585/55083/3976/255743/7490	8

© 2020 Yu Y et al. *JAMA Network Open.*

BP	GO:0030595	leukocyte chemotaxis	12/408	188/17913	0.0012907 42	0.0115463 34	0.0084353 83	6356/6387/1588/9420/2621/26585/5919/7857/5054/7042/7057/7060	12
BP	GO:0051604	protein maturation	18/408	353/17913	0.0012945 15	0.0115527 05	0.0084400 38	9509/715/716/1066/3075/1311/10699/2619/2737/85409/5122/5125/5328/5627/5054/5270/10418/7057	18
BP	GO:0031639	plasminogen activation	4/408	22/17913	0.0014010 57	0.0123863 94	0.0090491 05	5328/5054/5270/7057	4
BP	GO:0033622	integrin activation	4/408	22/17913	0.0014010 57	0.0123863 94	0.0090491 05	1307/6387/10979/2335	4
BP	GO:0045649	regulation of macrophage differentiation	4/408	22/17913	0.0014010 57	0.0123863 94	0.0090491 05	9370/3624/3976/4920	4
BP	GO:0097205	renal filtration	4/408	22/17913	0.0014010 57	0.0123863 94	0.0090491 05	2621/2702/23213/55959	4
BP	GO:00	neural crest cell migration	5/408	37/179	0.00143	0.01249	0.00913	2335/9464/3357/8828/7291	5

© 2020 Yu Y et al. *JAMA Network Open.*

	01755			13	0374	922	1532		
BP	GO:0001990	regulation of systemic arterial blood pressure by hormone	5/408	37/17913	0.0014303 74	0.0124992 2	0.0091315 32	185/1066/10699/2702/5125	5
BP	GO:0014912	negative regulation of smooth muscle cell migration	5/408	37/17913	0.0014303 74	0.0124992 2	0.0091315 32	9370/3488/5592/5054/7168	5
BP	GO:0060306	regulation of membrane repolarization	5/408	37/17913	0.0014303 74	0.0124992 2	0.0091315 32	781/857/2316/2702/6330	5
BP	GO:1905314	semi-lunar valve development	5/408	37/17913	0.0014303 74	0.0124992 2	0.0091315 32	11117/2702/6591/7042/7291	5
BP	GO:0042472	inner ear morphogenesis	8/408	95/17913	0.0014625 26	0.0127024 77	0.0092800 25	220/1301/115908/2138/2260/2736/5396/4920	8
BP	GO:0061326	renal tubule development	8/408	95/17913	0.0014625 26	0.0127024 77	0.0092800 25	170690/2138/2737/26585/55083/3976/255743/7490	8

© 2020 Yu Y et al. *JAMA Network Open.*

BP	GO:0086001	cardiac muscle cell action potential	7/408	74/17913	0.00146372 9	0.01270247 7	0.00928002 5	781/857/2316/2702/23704/3764/6330	7
BP	GO:0048008	platelet-derived growth factor receptor signaling pathway	6/408	55/17913	0.00152023 5	0.01316258 1	0.00961616 2	9370/4015/56034/5156/5159/5157	6
BP	GO:0031100	animal organ regeneration	7/408	75/17913	0.00158342 8	0.01364712 6	0.00997015 5	558/6387/2621/2737/3082/4837/55959	7
BP	GO:0072078	nephron tubule morphogenesis	7/408	75/17913	0.00158342 8	0.01364712 6	0.00997015 5	170690/2138/2737/26585/55083/255743/7490	7
BP	GO:0043687	post-translational protein modification	18/408	360/17913	0.00160964	0.01383941 4	0.01011063 5	83987/1000/56975/2200/114907/2335/11167/2621/3488/3490/3956/4148/54587/7857/6383/7076/3371/1462	18
BP	GO:0007431	salivary gland development	5/408	38/17913	0.00161673 7	0.01383941 4	0.01011063 5	2260/3082/6591/7042/7043	5
BP	GO:00	positive regulation of cell migration involved in sprouting	5/408	38/179	0.00161	0.01383	0.01011	2303/57608/10769/5743/27286	5

© 2020 Yu Y et al. *JAMA Network Open.*

	90050	angiogenesis		13	6737	9414	0635		
BP	GO:1903670	regulation of sprouting angiogenesis	10/408	143/17913	0.0016360045	0.0139730009	0.0102082035	185/90993/2303/3678/57608/4223/10769/5743/27286/7057	10
BP	GO:0071526	semaphorin-plexin signaling pathway	4/408	23/17913	0.00166582	0.01407522	0.010282907	2316/9464/8828/91584	4
BP	GO:0072215	regulation of metanephros development	4/408	23/17913	0.00166582	0.01407522	0.010282907	9370/3976/5159/7490	4
BP	GO:0060047	heart contraction	15/408	275/17913	0.001673005	0.01407522	0.010282907	10060/552/781/857/10699/2316/2702/23704/3764/5999/6330/6444/6781/7042/7168	15
BP	GO:0006813	potassium ion transport	12/408	194/17913	0.001685733	0.01407522	0.010282907	10060/88/857/2316/2702/3764/3776/343450/259232/5999/25769/57419	12
BP	GO:0031099	regeneration	12/408	194/17913	0.001685733	0.01407522	0.010282907	558/6387/2621/2737/3082/4131/4837/10631/5396/55959/7070/3371	12

© 2020 Yu Y et al. *JAMA Network Open.*

BP	GO:0010755	regulation of plasminogen activation	3/408	11/17913	0.0016890 26	0.0140752 2	0.0102829 07	5054/5270/7057	3
BP	GO:0014831	gastro-intestinal system smooth muscle contraction	3/408	11/17913	0.0016890 26	0.0140752 2	0.0102829 07	3357/23213/55959	3
BP	GO:0032908	regulation of transforming growth factor beta1 production	3/408	11/17913	0.0016890 26	0.0140752 2	0.0102829 07	2627/4060/7057	3
BP	GO:0097048	dendritic cell apoptotic process	3/408	11/17913	0.0016890 26	0.0140752 2	0.0102829 07	558/6387/2621	3
BP	GO:1901201	regulation of extracellular matrix assembly	3/408	11/17913	0.0016890 26	0.0140752 2	0.0102829 07	84168/11117/3037	3
BP	GO:2000668	regulation of dendritic cell apoptotic process	3/408	11/17913	0.0016890 26	0.0140752 2	0.0102829 07	558/6387/2621	3
BP	GO:00	muscle tissue morphogenesis	7/408	76/179	0.0017 1	0.0142 2	0.0103 9	1301/1281/2303/7042/7168/7472/23414	7

© 2020 Yu Y et al. *JAMA Network Open*.

	60415			13	0611	3692	1376		
BP	GO:0045834	positive regulation of lipid metabolic process	10/408	144/17913	0.001723373	0.014298315	0.010445894	9370/185/552/3357/8660/5156/5159/5587/5743/7291	10
BP	GO:0014074	response to purine-containing compound	10/408	145/17913	0.001814437	0.01497111	0.010937416	9370/1277/3488/4327/50507/5139/5743/6678/6781/7490	10
BP	GO:0030049	muscle filament sliding	5/408	39/17913	0.001820328	0.01497111	0.010937416	88/4620/7168/7169/7431	5
BP	GO:0033275	actin-myosin filament sliding	5/408	39/17913	0.001820328	0.01497111	0.010937416	88/4620/7168/7169/7431	5
BP	GO:2000826	regulation of heart morphogenesis	5/408	39/17913	0.001820328	0.01497111	0.010937416	2138/2303/9464/7042/7291	5
BP	GO:0060688	regulation of morphogenesis of a branching structure	6/408	57/17913	0.001831877	0.015000673	0.010959014	2260/26585/3082/6591/23213/7472	6

© 2020 Yu Y et al. *JAMA Network Open.*

BP	GO:1902041	regulation of extrinsic apoptotic signaling pathway via death domain receptors	6/408	57/17913	0.00183187	0.015000673	0.010959014	658/3082/5054/6423/7057/7078	6
BP	GO:1902904	negative regulation of supramolecular fiber organization	9/408	122/17913	0.001914207	0.015640979	0.011426801	1410/11117/80206/4131/91663/89795/90102/130271/11075	9
BP	GO:0009743	response to carbohydrate	13/408	224/17913	0.001945019	0.015858418	0.011585655	9370/55107/1292/81035/2621/8660/3956/4131/50507/5743/5176/6678/7057	13
BP	GO:0046697	decidualization	4/408	24/17913	0.001963422	0.015939608	0.01164497	3976/5740/5743/6781	4
BP	GO:2000209	regulation of anoikis	4/408	24/17913	0.001963422	0.015939608	0.01164497	857/3678/4915/6591	4
BP	GO:0048660	regulation of smooth muscle cell proliferation	11/408	172/17913	0.00199727	0.016179602	0.011820302	9370/1012/3488/4313/4883/5159/5592/5743/7043/7057/7168	11
BP	GO:00	lung alveolus development	5/408	40/179	0.00204	0.01650	0.01205	2627/3488/3976/91461/7043	5

© 2020 Yu Y et al. *JAMA Network Open.*

	48<br>28<br>6			13	20<br>73	71<br>18	95<br>75		
B<br>P	G<br>O:<br>00<br>61<br>33<br>3	renal tubule morphogenesis	7/<br>40<br>8	79<br>/1<br>79<br>13	0.0<br>02<br>14<br>02<br>21	0.0<br>17<br>26<br>35<br>33	0.0<br>12<br>61<br>21<br>87	170690/2138/2737/26585/55083/255743/7490	7
B<br>P	G<br>O:<br>00<br>02<br>04<br>2	cell migration involved in sprouting angiogenesis	8/<br>40<br>8	10<br>1/<br>17<br>91<br>3	0.0<br>02<br>16<br>17<br>29	0.0<br>17<br>36<br>28<br>23	0.0<br>12<br>68<br>47<br>26	2303/26585/57608/4223/10769/5743/27286/7057	8
B<br>P	G<br>O:<br>00<br>55<br>02<br>4	regulation of cardiac muscle tissue development	8/<br>40<br>8	10<br>1/<br>17<br>91<br>3	0.0<br>02<br>16<br>17<br>29	0.0<br>17<br>36<br>28<br>23	0.0<br>12<br>68<br>47<br>26	2260/2627/26585/57158/3776/5999/7472/23414	8
B<br>P	G<br>O:<br>00<br>48<br>65<br>9	smooth muscle cell proliferation	11<br>/4<br>08	17<br>4/<br>17<br>91<br>3	0.0<br>02<br>18<br>71<br>8	0.0<br>17<br>51<br>97<br>47	0.0<br>12<br>79<br>93<br>69	9370/1012/3488/4313/4883/5159/5592/5743/7043/7057/7168	1<br>1
B<br>P	G<br>O:<br>00<br>09<br>88<br>6	post-embryonic animal morphogenesis	3/<br>40<br>8	12<br>/1<br>79<br>13	0.0<br>02<br>21<br>41<br>84	0.0<br>17<br>51<br>97<br>47	0.0<br>12<br>79<br>93<br>69	2202/2200/4237	3
B<br>P	G<br>O:<br>00<br>32<br>90<br>5	transforming growth factor beta1 production	3/<br>40<br>8	12<br>/1<br>79<br>13	0.0<br>02<br>21<br>41<br>84	0.0<br>17<br>51<br>97<br>47	0.0<br>12<br>79<br>93<br>69	2627/4060/7057	3

BP	GO:0048934	peripheral nervous system neuron differentiation	3/408	12/17913	0.0022141 84	0.0175197 47	0.0127993 69	2115/9464/4915	3
BP	GO:0048935	peripheral nervous system neuron development	3/408	12/17913	0.0022141 84	0.0175197 47	0.0127993 69	2115/9464/4915	3
BP	GO:0060394	negative regulation of pathway-restricted SMAD protein phosphorylation	3/408	12/17913	0.0022141 84	0.0175197 47	0.0127993 69	11117/26585/56937	3
BP	GO:1905522	negative regulation of macrophage migration	3/408	12/17913	0.0022141 84	0.0175197 47	0.0127993 69	4345/1588/11117	3
BP	GO:0016202	regulation of striated muscle tissue development	10/408	149/17913	0.0022183 94	0.0175197 47	0.0127993 69	2260/2627/26585/57158/3776/4015/5999/7291/7472/23414	10
BP	GO:0090184	positive regulation of kidney development	5/408	41/17913	0.0022829	0.0179438 11	0.0131091 77	9370/26585/3976/5159/7490	5
BP	GO:00	renal system process involved in regulation of systemic arterial blood	4/408	25/179	0.0022 9	0.0179 4	0.0131 0	185/2621/2702/5125	4

© 2020 Yu Y et al. *JAMA Network Open*.

	03071	pressure		13	5857	3811	9177		
BP	GO:0044062	regulation of excretion	4/408	25/17913	0.002295857	0.017943811	0.0131091 77	185/552/10699/6781	4
BP	GO:0060561	apoptotic process involved in morphogenesis	4/408	25/17913	0.002295857	0.017943811	0.0131091 77	1410/2303/9464/7042	4
BP	GO:0060740	prostate gland epithelium morphogenesis	4/408	25/17913	0.002295857	0.017943811	0.0131091 77	9420/2736/23213/3371	4
BP	GO:0042063	gliogenesis	15/408	285/17913	0.0023628 95	0.0184296 02	0.0134640 8	1000/1464/2737/3953/3976/54587/4915/4919/4920/5270/26011/7042/10100/7431/22891	15
BP	GO:0001658	branching involved in ureteric bud morphogenesis	6/408	60/17913	0.0023871 17	0.0185459 83	0.0135491 04	170690/2138/2737/26585/255743/7490	6
BP	GO:0051668	localization within membrane	9/408	126/17913	0.0023876 42	0.0185459 83	0.0135491 04	1012/1000/2316/2743/2239/10082/3690/9379/7070	9

© 2020 Yu Y et al. *JAMA Network Open*.

BP	GO:0043410	positive regulation of MAPK cascade	22/408	499/17913	0.00254700 4	0.01974320 2	0.01442375 4	9590/114897/1000/1464/2260/2621/2690/9464/3082/3357/57608/3976/50507/255743/4915/5156/5159/4920/7042/7043/7057/7077	22
BP	GO:0034113	heterotypic cell-cell adhesion	6/408	61/17913	0.00259769 8	0.01997212 1	0.01459099 5	9370/4345/3678/3690/91663/7070	6
BP	GO:0042733	embryonic digit morphogenesis	6/408	61/17913	0.00259769 8	0.01997212 1	0.01459099 5	2736/2737/9464/4920/6423/7291	6
BP	GO:0051893	regulation of focal adhesion assembly	6/408	61/17913	0.00259769 8	0.01997212 1	0.01459099 5	1307/2042/26585/90102/7057/7070	6
BP	GO:0090109	regulation of cell-substrate junction assembly	6/408	61/17913	0.00259769 8	0.01997212 1	0.01459099 5	1307/2042/26585/90102/7057/7070	6
BP	GO:0010721	negative regulation of cell development	16/408	317/17913	0.00261128 4	0.02003576 7	0.01463749 3	1281/1809/2200/2737/3956/10769/5376/10631/5744/5999/6695/11075/7070/63923/7431/22891	16
BP	GO:00	axon development	20/408	438/17	0.00264	0.02004	0.01464	3730/658/1009/6387/2042/2115/23768/2335/2736/2737/8660/4131/4685/8828/4915/91584/7070/3371/63923/10100	20

© 2020 Yu Y et al. *JAMA Network Open*.

	61564			913	5155	1284	1524		
BP	GO:0002053	positive regulation of mesenchymal cell proliferation	4/408	26/17913	0.002665093	0.020041284	0.014641524	2260/8660/5396/7472	4
BP	GO:0019934	cGMP-mediated signaling	4/408	26/17913	0.002665093	0.020041284	0.014641524	3357/5139/5592/7057	4
BP	GO:0030201	heparan sulfate proteoglycan metabolic process	4/408	26/17913	0.002665093	0.020041284	0.014641524	29940/23213/55959/64131	4
BP	GO:0032967	positive regulation of collagen biosynthetic process	4/408	26/17913	0.002665093	0.020041284	0.014641524	90993/5159/7043/7431	4
BP	GO:0045992	negative regulation of embryonic development	4/408	26/17913	0.002665093	0.020041284	0.014641524	1289/1290/6423/23213	4
BP	GO:0050919	negative chemotaxis	4/408	26/17913	0.002665093	0.020041284	0.014641524	23768/3690/8828/91584	4

© 2020 Yu Y et al. *JAMA Network Open*.

BP	GO:0060351	cartilage development involved in endochondral bone morphogenesis	4/408	26/17913	0.0026650 93	0.0200412 84	0.0146415 24	8313/1277/1311/6781	4
BP	GO:1903792	negative regulation of anion transport	4/408	26/17913	0.0026650 93	0.0200412 84	0.0146415 24	8660/5999/6424/7057	4
BP	GO:1905048	regulation of metallopeptidase activity	4/408	26/17913	0.0026650 93	0.0200412 84	0.0146415 24	84168/7076/7077/7078	4
BP	GO:0048634	regulation of muscle organ development	10/408	153/17913	0.0026917 43	0.0202014 49	0.0147585 35	2260/2627/26585/57158/3776/4015/5999/7291/7472/23414	10
BP	GO:0007389	pattern specification process	19/408	408/17913	0.0026997 27	0.0202211 7	0.0147729 42	8313/80114/658/2138/2260/2303/2736/2737/26585/9464/4223/8828/5125/4920/6423/6781/9096/7472/7490	19
BP	GO:0002688	regulation of leukocyte chemotaxis	8/408	105/17913	0.0027577 33	0.0206147 39	0.0150604 71	6387/1588/2621/26585/5919/5054/7057/7060	8
BP	GO:00	keratinocyte proliferation	5/408	43/179	0.0028 2	0.0207 4	0.0151 5	1012/3037/687/5587/6591	5

© 2020 Yu Y et al. *JAMA Network Open*.

	43616			13	5546	502	5651		
BP	GO:0044060	regulation of endocrine process	5/408	43/17913	0.002825546	0.02074502	0.01515565651	185/114897/2260/2702/3624	5
BP	GO:0060443	mammary gland morphogenesis	5/408	43/17913	0.002825546	0.02074502	0.01515565651	857/6356/2736/2737/3488	5
BP	GO:0010763	positive regulation of fibroblast migration	3/408	13/17913	0.002830115	0.02074502	0.01515565651	9590/4921/7057	3
BP	GO:0031953	negative regulation of protein autophosphorylation	3/408	13/17913	0.002830115	0.02074502	0.01515565651	9370/857/5167	3
BP	GO:0051917	regulation of fibrinolysis	3/408	13/17913	0.002830115	0.02074502	0.01515565651	2191/5054/7057	3
BP	GO:0061430	bone trabecula morphogenesis	3/408	13/17913	0.002830115	0.02074502	0.01515565651	1277/26585/4313	3

© 2020 Yu Y et al. *JAMA Network Open*.

BP	GO:0098903	regulation of membrane repolarization during action potential	3/408	13/17913	0.002830115	0.02074502	0.015155651	781/857/2316	3
BP	GO:1903960	negative regulation of anion transmembrane transport	3/408	13/17913	0.002830115	0.02074502	0.015155651	8660/5999/7057	3
BP	GO:2001044	regulation of integrin-mediated signaling pathway	3/408	13/17913	0.002830115	0.02074502	0.015155651	2316/5587/7076	3
BP	GO:0090066	regulation of anatomical structure size	20/408	442/17913	0.002932876	0.021456601	0.015675509	59/88/185/857/6356/1311/6387/1909/80206/2335/2303/2702/2982/3357/4131/91663/130271/91584/5592/5743	20
BP	GO:0031960	response to corticosteroid	10/408	155/17913	0.002956983	0.02159112	0.015773785	9370/552/1277/114907/3490/687/5743/5176/6678/6781	10
BP	GO:0060420	regulation of heart growth	7/408	84/17913	0.003036132	0.022126253	0.016164735	2260/2627/3776/5999/7472/7490/23414	7
BP	GO:00	cardiac muscle tissue morphogenesis	6/408	63/179	0.00306	0.02218	0.01620	1301/2303/7042/7168/7472/23414	6

	55008			13	0311	1278	4935		
BP	GO:0003081	regulation of systemic arterial blood pressure by renin-angiotensin	4/408	27/17913	0.003073062	0.022181278	0.016204935	185/1066/2702/5125	4
BP	GO:0003148	outflow tract septum morphogenesis	4/408	27/17913	0.003073062	0.022181278	0.016204935	2627/8828/7042/23414	4
BP	GO:0010714	positive regulation of collagen metabolic process	4/408	27/17913	0.003073062	0.022181278	0.016204935	90993/5159/7043/7431	4
BP	GO:0060512	prostate gland morphogenesis	4/408	27/17913	0.003073062	0.022181278	0.016204935	9420/2736/23213/3371	4
BP	GO:0009952	anterior/posterior pattern specification	11/408	182/17913	0.003098834	0.022288471	0.016283247	8313/2303/2736/2737/4223/5125/4920/6423/9096/7472/7490	11
BP	GO:0060071	Wnt signaling pathway, planar cell polarity pathway	8/408	107/17913	0.003100197	0.022288471	0.016283247	115908/51339/2239/10082/144165/4919/4920/6423	8

© 2020 Yu Y et al. *JAMA Network Open*.

BP	GO:0030225	macrophage differentiation	5/408	44/17913	0.000312924 2	0.022288471	0.016283247	9370/3624/3976/4920/10100	5
BP	GO:0032965	regulation of collagen biosynthetic process	5/408	44/17913	0.000312924 2	0.022288471	0.016283247	90993/11117/5159/7043/7431	5
BP	GO:0048483	autonomic nervous system development	5/408	44/17913	0.000312924 2	0.022288471	0.016283247	1909/2335/9464/8828/91584	5
BP	GO:0048701	embryonic cranial skeleton morphogenesis	5/408	44/17913	0.000312924 2	0.022288471	0.016283247	2303/5156/5396/7043/7291	5
BP	GO:0060393	regulation of pathway-restricted SMAD protein phosphorylation	5/408	44/17913	0.000312924 2	0.022288471	0.016283247	11117/26585/3624/56937/7043	5
BP	GO:0010976	positive regulation of neuron projection development	14/408	265/17913	0.000315010 8	0.022394836	0.016360954	1272/6387/1809/2042/2260/2335/3082/4131/4915/5587/5789/5176/11075/55714	14
BP	GO:00	regulation of cell migration involved in sprouting angiogenesis	7/408	85/179	0.00324	0.02302	0.01682	2303/57608/4223/10769/5743/27286/7057	7

	90049			13	5352	8576	3944		
BP	GO:0007015	actin filament organization	16/408	325/17913	0.003335021	0.023620458	0.017256354	88/6356/84952/6387/1809/80206/2316/91663/50507/27295/90102/130271/5999/7043/7168/165904	16
BP	GO:0010463	mesenchymal cell proliferation	5/408	45/17913	0.003455774	0.024132503	0.017630438	2260/9464/8660/5396/7472	5
BP	GO:0071675	regulation of mononuclear cell migration	5/408	45/17913	0.003455774	0.024132503	0.017630438	6387/26585/5919/5054/7057	5
BP	GO:0033138	positive regulation of peptidyl-serine phosphorylation	7/408	86/17913	0.003465398	0.024132503	0.017630438	857/2621/3976/4915/5587/5743/6423	7
BP	GO:0048145	regulation of fibroblast proliferation	7/408	86/17913	0.003465398	0.024132503	0.017630438	4921/2335/2621/56034/5156/5159/7472	7
BP	GO:0008543	fibroblast growth factor receptor signaling pathway	8/408	109/17913	0.003474863	0.024132503	0.017630438	3730/90993/56975/2260/23768/23213/55959/7057	8

© 2020 Yu Y et al. *JAMA Network Open*.

BP	GO:0048593	camera-type eye morphogenesis	8/408	109/17913	0.00347486 3	0.02413250 3	0.01763043 8	220/1295/1296/2737/55714/7070/7291/7472	8
BP	GO:0090175	regulation of establishment of planar polarity	8/408	109/17913	0.00347486 3	0.02413250 3	0.01763043 8	115908/51339/2239/10082/144165/4919/4920/6423	8
BP	GO:0032956	regulation of actin cytoskeleton organization	15/408	297/17913	0.00348681 4	0.02413250 3	0.01763043 8	88/6356/6387/2042/80206/2316/91663/50507/5156/5159/90102/130271/5999/7043/7168	15
BP	GO:0010001	glial cell differentiation	12/408	212/17913	0.00350333 8	0.02413250 3	0.01763043 8	1000/2737/3976/54587/4915/4919/4920/5270/26011/10100/7431/22891	12
BP	GO:0033688	regulation of osteoblast proliferation	4/408	28/17913	0.00352166	0.02413250 3	0.01763043 8	115908/26585/4883/63923	4
BP	GO:0050974	detection of mechanical stimulus involved in sensory perception	4/408	28/17913	0.00352166	0.02413250 3	0.01763043 8	1301/6387/63895/5270	4
BP	GO:19	response to angiotensin	4/408	28/179	0.00352	0.02413	0.01763	88/185/857/5743	4

© 2020 Yu Y et al. *JAMA Network Open.*

	90<br>77<br>6			13	16<br>6	25<br>03	04<br>38		
B<br>P	G<br>O:<br>00<br>07<br>50<br>1	mesodermal cell fate specification	3/<br>40<br>8	14<br>/1<br>79<br>13	0.0<br>03<br>54<br>15<br>65	0.0<br>24<br>13<br>25<br>03	0.0<br>17<br>63<br>04<br>38	2138/2260/6423	3
B<br>P	G<br>O:<br>00<br>10<br>93<br>5	regulation of macrophage cytokine production	3/<br>40<br>8	14<br>/1<br>79<br>13	0.0<br>03<br>54<br>15<br>65	0.0<br>24<br>13<br>25<br>03	0.0<br>17<br>63<br>04<br>38	10417/7042/7043	3
B<br>P	G<br>O:<br>00<br>35<br>79<br>1	platelet-derived growth factor receptor-beta signaling pathway	3/<br>40<br>8	14<br>/1<br>79<br>13	0.0<br>03<br>54<br>15<br>65	0.0<br>24<br>13<br>25<br>03	0.0<br>17<br>63<br>04<br>38	4015/5159/5157	3
B<br>P	G<br>O:<br>00<br>38<br>16<br>6	angiotensin-activated signaling pathway	3/<br>40<br>8	14<br>/1<br>79<br>13	0.0<br>03<br>54<br>15<br>65	0.0<br>24<br>13<br>25<br>03	0.0<br>17<br>63<br>04<br>38	88/185/857	3
B<br>P	G<br>O:<br>00<br>45<br>98<br>6	negative regulation of smooth muscle contraction	3/<br>40<br>8	14<br>/1<br>79<br>13	0.0<br>03<br>54<br>15<br>65	0.0<br>24<br>13<br>25<br>03	0.0<br>17<br>63<br>04<br>38	2982/5592/5743	3
B<br>P	G<br>O:<br>00<br>48<br>56<br>9	post-embryonic animal organ development	3/<br>40<br>8	14<br>/1<br>79<br>13	0.0<br>03<br>54<br>15<br>65	0.0<br>24<br>13<br>25<br>03	0.0<br>17<br>63<br>04<br>38	2202/2200/4237	3

© 2020 Yu Y et al. *JAMA Network Open.*

BP	GO:1902043	positive regulation of extrinsic apoptotic signaling pathway via death domain receptors	3/408	14/17913	0.000354156 5	0.002413250 3	0.001763043 8	658/7057/7078	3
BP	GO:1903054	negative regulation of extracellular matrix organization	3/408	14/17913	0.000354156 5	0.002413250 3	0.001763043 8	84168/11117/2191	3
BP	GO:1905049	negative regulation of metallopeptidase activity	3/408	14/17913	0.000354156 5	0.002413250 3	0.001763043 8	7076/7077/7078	3
BP	GO:0007548	sex differentiation	14/408	269/17913	0.000360076 5	0.002449169 2	0.001789285	558/658/1588/2316/2627/3624/4327/5156/5159/4920/6423/7042/7490/23414	14
BP	GO:0022407	regulation of cell-cell adhesion	18/408	389/17913	0.000369283 3	0.002504699 8	0.001829853 9	170692/9370/114897/857/6387/2736/2737/3037/55083/3956/2615/91663/10630/5592/5270/6591/55714/7070	18
BP	GO:0048144	fibroblast proliferation	7/408	87/17913	0.000369662 7	0.002504699 8	0.001829853 9	4921/2335/2621/56034/5156/5159/7472	7
BP	GO:00	negative regulation of hydrolase activity	19/408	420/17	0.00370	0.02504	0.01829	3730/83987/1293/1410/1470/1893/2621/3082/3953/5627/5743/5054/6423/6695/7042/7057/7076/7077/7078	19

	51346			913	2311	6998	8539		
BP	GO:0046661	male sex differentiation	10/408	160/17913	0.003711982	0.025067497	0.018313514	2316/2627/3624/5156/5159/4920/6423/7042/7490/23414	10
BP	GO:0010977	negative regulation of neuron projection development	9/408	135/17913	0.003793718	0.025494289	0.018625316	1809/3956/10769/5376/6695/11075/7070/7431/22891	9
BP	GO:0030514	negative regulation of BMP signaling pathway	5/408	46/17913	0.00380608	0.025494289	0.018625316	2200/26585/5654/94031/6423	5
BP	GO:0031638	zymogen activation	5/408	46/17913	0.00380608	0.025494289	0.018625316	715/5328/5054/5270/7057	5
BP	GO:0048806	genitalia development	5/408	46/17913	0.00380608	0.025494289	0.018625316	558/1588/5156/4920/7490	5
BP	GO:0050866	negative regulation of cell activation	11/408	187/17913	0.003808948	0.025494289	0.018625316	170692/558/114897/4345/11117/2737/3624/2615/5156/5592/5270	11

BP	GO:0051965	positive regulation of synapse assembly	6/408	66/17913	0.0038660010	0.0258303610	0.0188708390	23768/4915/5789/139065/27286/7058	6
BP	GO:0046620	regulation of organ growth	8/408	111/17913	0.0038837060	0.0259028090	0.0189237670	2260/2303/2627/3776/5999/7472/7490/23414	8
BP	GO:0042445	hormone metabolic process	12/408	215/17913	0.0039212570	0.0261071320	0.0190730390	220/1066/10699/1588/1545/1734/2260/2690/8630/5122/5125/5156	12
BP	GO:0006929	substrate-dependent cell migration	4/408	29/17913	0.0040127410	0.0266223160	0.0194494160	2335/22801/8828/6591	4
BP	GO:0010575	positive regulation of vascular endothelial growth factor production	4/408	29/17913	0.0040127410	0.0266223160	0.0194494160	1545/5743/23213/55959	4
BP	GO:0031346	positive regulation of cell projection organization	17/408	362/17913	0.0040697010	0.0269528440	0.0196908890	1272/6387/1809/22873/2042/2260/2335/3082/4131/89795/4915/5587/5789/5176/11075/55714/7043	17
BP	GO:19	regulation of adherens junction organization	6/408	67/179	0.00416	0.02749	0.02008	1307/2042/26585/90102/7057/7070	6

© 2020 Yu Y et al. *JAMA Network Open*.

	03391			13	6566	7616	8882		
BP	GO:0008584	male gonad development	9/408	137/17913	0.004179993	0.027497616	0.020088882	2316/2627/3624/5156/5159/6423/7042/7490/23414	9
BP	GO:0014911	positive regulation of smooth muscle cell migration	5/408	47/17913	0.004181094	0.027497616	0.020088882	3037/3488/50507/5159/10631	5
BP	GO:1903115	regulation of actin filament-based movement	5/408	47/17913	0.004181094	0.027497616	0.020088882	857/2316/2702/10630/6781	5
BP	GO:0008406	gonad development	12/408	217/17913	0.00422149	0.027715003	0.020247698	658/1588/2316/2627/3624/4327/5156/5159/6423/7042/7490/23414	12
BP	GO:0050680	negative regulation of epithelial cell proliferation	10/408	163/17913	0.004233576	0.027746092	0.020270411	857/687/7857/5176/6423/6591/6678/23213/7042/7057	10
BP	GO:0003279	cardiac septum development	8/408	113/17913	0.004328874	0.028320612	0.020690137	11174/2627/2702/8828/5125/57167/7042/23414	8

© 2020 Yu Y et al. *JAMA Network Open*.

BP	GO:0034356	NAD biosynthesis via nicotinamide riboside salvage pathway	3/408	15/17913	0.000435280 8	0.002842880 5	0.002076917 9	4837/5740/5743	3
BP	GO:0030879	mammary gland development	9/408	138/17913	0.000438430 6	0.002853578 5	0.002084733 5	857/6356/1588/2736/2737/3488/8660/7043/7472	9
BP	GO:0046546	development of primary male sexual characteristics	9/408	138/17913	0.000438430 6	0.002853578 5	0.002084733 5	2316/2627/3624/5156/5159/6423/7042/7490/23414	9
BP	GO:0006937	regulation of muscle contraction	10/408	164/17913	0.000441973 5	0.002871686 6	0.002097962 8	857/2316/2702/2982/10398/255743/5592/5743/6781/7168	10
BP	GO:0001558	regulation of cell growth	18/408	396/17913	0.000444037 8	0.002880142 4	0.002104140 3	185/552/1410/6387/5167/2335/26585/3488/3490/3624/4131/91584/5999/5270/6423/6695/7042/7490	18
BP	GO:0007202	activation of phospholipase C activity	4/408	30/17913	0.000454811 2	0.002921638 1	0.002134455 7	552/1909/3357/4915	4
BP	GO:00	negative regulation of smoothened signaling pathway	4/408	30/179	0.00454	0.002921	0.002134	5167/2737/84662/5270	4

© 2020 Yu Y et al. *JAMA Network Open*.

	45879			13	8112	6381	4557		
BP	GO:0061082	myeloid leukocyte cytokine production	4/408	30/17913	0.004548112	0.029216381	0.021344557	2621/10417/7042/7043	4
BP	GO:0099623	regulation of cardiac muscle cell membrane repolarization	4/408	30/17913	0.004548112	0.029216381	0.021344557	781/2316/2702/6330	4
BP	GO:1904837	beta-catenin-TCF complex assembly	4/408	30/17913	0.004548112	0.029216381	0.021344557	8313/51339/26108/83439	4
BP	GO:0046660	female sex differentiation	8/408	114/17913	0.004565468	0.029216381	0.021344557	558/658/1588/3624/4327/5156/7490/23414	8
BP	GO:0022411	cellular component disassembly	21/408	492/17913	0.004578513	0.029216381	0.021344557	88/9507/857/1513/4921/2191/5654/4131/4320/4327/4313/4314/89795/10630/130271/6591/11075/7043/7076/7077/7092	21
BP	GO:0002686	negative regulation of leukocyte migration	5/408	48/17913	0.004581748	0.029216381	0.021344557	4345/6387/1588/11117/26585	5

© 2020 Yu Y et al. *JAMA Network Open*.

BP	GO:0048546	digestive tract morphogenesis	5/408	48/17913	0.0045817 48	0.0292163 81	0.0213445 57	51339/2736/2737/5156/6423	5
BP	GO:0055010	ventricular cardiac muscle tissue morphogenesis	5/408	48/17913	0.0045817 48	0.0292163 81	0.0213445 57	1301/2303/7042/7168/23414	5
BP	GO:0051384	response to glucocorticoid	9/408	139/17913	0.0045963 23	0.0292598 99	0.0213763 5	9370/552/114907/3490/687/5743/5176/6678/6781	9
BP	GO:0010927	cellular component assembly involved in morphogenesis	7/408	91/17913	0.0047405 79	0.0301274 2	0.0220101 33	88/80206/5156/5159/90102/26011/7168	7
BP	GO:0071804	cellular potassium ion transport	9/408	140/17913	0.0048162 38	0.0304720 23	0.0222618 9	10060/88/857/2316/3764/3776/343450/259232/5999	9
BP	GO:0071805	potassium ion transmembrane transport	9/408	140/17913	0.0048162 38	0.0304720 23	0.0222618 9	10060/88/857/2316/3764/3776/343450/259232/5999	9
BP	GO:00	negative regulation of smooth muscle cell proliferation	6/408	69/179	0.004 81	0.030 47	0.022 26	9370/3488/4883/5592/7043/7168	6

	48662			13	9019	2023	189		
BP	GO:0009100	glycoprotein metabolic process	17/408	369/17913	0.004918741	0.031050581	0.022684565	81792/633/658/1301/1464/1634/29940/92126/117248/11227/727936/22822/81849/23213/55959/1462/64131	17
BP	GO:0035272	exocrine system development	5/408	49/17913	0.005008964	0.031462298	0.022985352	2260/3082/6591/7042/7043	5
BP	GO:0043113	receptor clustering	5/408	49/17913	0.005008964	0.031462298	0.022985352	1000/2316/2743/9379/7070	5
BP	GO:0060395	SMAD protein signal transduction	5/408	49/17913	0.005008964	0.031462298	0.022985352	8483/4920/7042/7043/7431	5
BP	GO:0033687	osteoblast proliferation	4/408	31/17913	0.005129529	0.032112726	0.023460534	115908/26585/4883/63923	4
BP	GO:0061036	positive regulation of cartilage development	4/408	31/17913	0.005129529	0.032112726	0.023460534	658/2737/4017/91461	4

© 2020 Yu Y et al. *JAMA Network Open.*

BP	GO:0045137	development of primary sexual characteristics	12/408	223/17913	0.0052343 6	0.0326526 74	0.0238550 04	658/1588/2316/2627/3624/4327/5156/5159/6423/7042/7490/23414	12
BP	GO:0016049	cell growth	20/408	466/17913	0.0052652 47	0.0326526 74	0.0238550 04	185/552/1410/6387/5167/2335/26585/3488/3490/3624/4131/8828/91584/10631/5999/5270/6423/6695/7042/7490	20
BP	GO:0002076	osteoblast development	3/408	16/17913	0.0052676 76	0.0326526 74	0.0238550 04	2736/5745/63923	3
BP	GO:0060572	morphogenesis of an epithelial bud	3/408	16/17913	0.0052676 76	0.0326526 74	0.0238550 04	2736/23213/7472	3
BP	GO:0070293	renal absorption	3/408	16/17913	0.0052676 76	0.0326526 74	0.0238550 04	9370/2621/3037	3
BP	GO:0072283	metanephric renal vesicle morphogenesis	3/408	16/17913	0.0052676 76	0.0326526 74	0.0238550 04	26585/55083/3976	3
BP	GO:00	segmentation	7/408	93/179	0.00533	0.03298	0.02409	8313/2303/4223/8828/4920/6423/7490	7

© 2020 Yu Y et al. *JAMA Network Open.*

	35282			13	9091	7018	9265		
BP	GO:0044070	regulation of anion transport	7/408	93/17913	0.005339091	0.032987018	0.024099265	552/2260/8660/5999/6424/6781/7057	7
BP	GO:0022408	negative regulation of cell-cell adhesion	10/408	169/17913	0.005449419	0.033613657	0.024557067	170692/9370/114897/6387/2737/2615/91663/5592/5270/6591	10
BP	GO:0106106	cold-induced thermogenesis	9/408	143/17913	0.005525318	0.033970809	0.024817991	9370/857/1734/2303/3953/4237/4883/56034/23090	9
BP	GO:0120161	regulation of cold-induced thermogenesis	9/408	143/17913	0.005525318	0.033970809	0.024817991	9370/857/1734/2303/3953/4237/4883/56034/23090	9
BP	GO:0001707	mesoderm formation	6/408	71/17913	0.005543606	0.033972584	0.024819287	2138/2260/2303/3624/3690/6423	6
BP	GO:0031670	cellular response to nutrient	6/408	71/17913	0.005543606	0.033972584	0.024819287	1277/2621/10631/5743/6591/3371	6

© 2020 Yu Y et al. *JAMA Network Open.*

BP	GO:0006941	striated muscle contraction	10/408	170/17913	0.005676196	0.03472875	0.025371718	781/857/2316/2702/23704/3764/6330/6444/6781/7168	10
BP	GO:0048545	response to steroid hormone	17/408	375/17913	0.00575719	0.035006583	0.025574694	9370/552/857/1277/9420/114907/3490/687/4015/56937/5743/5176/6678/6781/7042/7043/7057	17
BP	GO:0016486	peptide hormone processing	4/408	32/17913	0.005758699	0.035006583	0.025574694	1066/10699/5122/5125	4
BP	GO:0071709	membrane assembly	4/408	32/17913	0.005758699	0.035006583	0.025574694	857/1000/9379/5789	4
BP	GO:0086019	cell-cell signaling involved in cardiac conduction	4/408	32/17913	0.005758699	0.035006583	0.025574694	781/2316/2702/6330	4
BP	GO:0033135	regulation of peptidyl-serine phosphorylation	8/408	119/17913	0.005901632	0.035817782	0.026167331	857/2621/3082/3976/4915/5587/5743/6423	8
BP	GO:00	adenylate cyclase-inhibiting G protein-coupled	6/408	72/179	0.00593	0.03586	0.02619	9590/1909/2316/2550/2774/4883	6

© 2020 Yu Y et al. *JAMA Network Open*.

	07 19 3	receptor signaling pathway		13	43 37	09 77	88 87		
BP	GO:0030239	myofibril assembly	5/408	51/17913	0.0059467 47	0.0358609 77	0.0261988 87	88/80206/5156/5159/7168	5
BP	GO:0045599	negative regulation of fat cell differentiation	5/408	51/17913	0.0059467 47	0.0358609 77	0.0261988 87	9370/5167/4320/862/23414	5
BP	GO:1900024	regulation of substrate adhesion-dependent cell spreading	5/408	51/17913	0.0059467 47	0.0358609 77	0.0261988 87	2316/3037/91663/10630/10631	5
BP	GO:0008585	female gonad development	7/408	95/17913	0.0059925 64	0.0360796 29	0.0263586 28	658/1588/3624/4327/5156/7490/23414	7
BP	GO:0035567	non-canonical Wnt signaling pathway	9/408	145/17913	0.0060411 28	0.0362019 42	0.0264479 86	115908/51339/2239/10082/144165/4919/4920/6423/6424	9
BP	GO:0055002	striated muscle cell development	9/408	145/17913	0.0060411 28	0.0362019 42	0.0264479 86	88/1311/80206/4015/5156/5159/5999/6444/7168	9

© 2020 Yu Y et al. *JAMA Network Open.*

BP	GO:0051493	regulation of cytoskeleton organization	20/408	472/17913	0.006041649	0.036201942	0.026447986	88/6356/6387/2042/80206/2316/4131/91663/89795/91624/50507/5156/5159/90102/130271/10769/5999/11075/7043/7168	20
BP	GO:0001659	temperature homeostasis	10/408	172/17913	0.006151821	0.036745451	0.026845056	9370/857/1734/2303/3953/4237/4883/56034/5743/23090	10
BP	GO:0007416	synapse assembly	10/408	172/17913	0.006151821	0.036745451	0.026845056	1000/23768/2239/4131/9379/4915/5789/139065/27286/7058	10
BP	GO:0110053	regulation of actin filament organization	12/408	228/17913	0.006218613	0.036983123	0.027018691	88/6356/6387/80206/2316/91663/50507/90102/130271/5999/7043/7168	12
BP	GO:0034284	response to monosaccharide	11/408	200/17913	0.006274317	0.036983123	0.027018691	9370/55107/1292/2621/8660/3956/50507/5743/5176/6678/7057	11
BP	GO:0010715	regulation of extracellular matrix disassembly	3/408	17/17913	0.00628958	0.036983123	0.027018691	4921/2191/10630	3
BP	GO:00	negative regulation of bone mineralization	3/408	17/179	0.00628	0.03698	0.02701	1893/5167/26585	3

© 2020 Yu Y et al. *JAMA Network Open*.

	30502			13	958	3123	8691		
BP	GO:0035313	wound healing, spreading of epidermal cells	3/408	17/17913	0.00628958	0.03698 3123	0.02701 8691	1289/3678/90102	3
BP	GO:0055003	cardiac myofibril assembly	3/408	17/17913	0.006289 58	0.03698 3123	0.02701 8691	80206/5156/5159	3
BP	GO:0060004	reflex	3/408	17/17913	0.00628958	0.0369831 23	0.0270186 91	220/2743/255743	3
BP	GO:0061298	retina vasculature development in camera-type eye	3/408	17/17913	0.00628958	0.0369831 23	0.0270186 91	1545/5156/5159	3
BP	GO:0061318	renal filtration cell differentiation	3/408	17/17913	0.00628958	0.0369831 23	0.0270186 91	9370/2303/7490	3
BP	GO:0072112	glomerular visceral epithelial cell differentiation	3/408	17/17913	0.00628958	0.0369831 23	0.0270186 91	9370/2303/7490	3

© 2020 Yu Y et al. *JAMA Network Open*.

BP	GO:0048511	rhythmic process	14/408	287/17913	0.006324998	0.037133544	0.027128585	9370/558/658/3037/3624/687/4327/4915/5156/5054/5176/7042/7043/7291	14
BP	GO:0048332	mesoderm morphogenesis	6/408	73/17913	0.006344767	0.037191764	0.027171118	2138/2260/2303/3624/3690/6423	6
BP	GO:0051897	positive regulation of protein kinase B signaling	10/408	173/17913	0.006400984	0.037397513	0.027321431	114897/2260/2621/3082/3488/8660/50507/5156/5159/7057	10
BP	GO:0070613	regulation of protein processing	10/408	173/17913	0.006400984	0.037397513	0.027321431	715/716/3075/2619/85409/5627/5054/5270/10418/7057	10
BP	GO:0010092	specification of animal organ identity	4/408	33/17913	0.006437269	0.037397513	0.027321431	8313/2260/2737/7472	4
BP	GO:0010837	regulation of keratinocyte proliferation	4/408	33/17913	0.006437269	0.037397513	0.027321431	3037/687/5587/6591	4
BP	GO:19	apoptotic process involved in development	4/408	33/179	0.00643	0.03739	0.02732	1410/2303/9464/7042	4

© 2020 Yu Y et al. *JAMA Network Open.*

	02742			13	7269	7513	1431		
BP	GO:0003179	heart valve morphogenesis	5/408	52/17913	0.006459912	0.037397513	0.027321431	11117/2702/6591/7042/7291	5
BP	GO:0060350	endochondral bone morphogenesis	5/408	52/17913	0.006459912	0.037397513	0.027321431	8313/658/1277/1311/6781	5
BP	GO:0071320	cellular response to cAMP	5/408	52/17913	0.006459912	0.037397513	0.027321431	9370/3488/50507/6781/7490	5
BP	GO:0003002	regionalization	15/408	318/17913	0.006489056	0.037513303	0.027406024	8313/658/2260/2303/2736/2737/26585/4223/8828/5125/4920/6423/9096/7472/7490	15
BP	GO:0034766	negative regulation of ion transmembrane transport	7/408	97/17913	0.006703974	0.038666829	0.028248753	88/857/2669/8660/5999/7057/7291	7
BP	GO:0070371	ERK1 and ERK2 cascade	14/408	289/17913	0.006709079	0.038666829	0.028248753	9370/9590/6356/11117/2335/2621/9464/3357/3976/50507/255743/5156/5159/7078	14

© 2020 Yu Y et al. *JAMA Network Open*.

BP	GO:0021675	nerve development	6/408	74/17913	0.006775447	0.038989805	0.02848471	2737/8828/91584/5270/23213/55959	6
BP	GO:0032368	regulation of lipid transport	8/408	122/17913	0.006834876	0.039212246	0.028647217	9370/185/114897/1588/8660/3690/5360/7057	8
BP	GO:0034763	negative regulation of transmembrane transport	8/408	122/17913	0.006834876	0.039212246	0.028647217	88/857/5167/2669/8660/5999/7057/7291	8
BP	GO:0010769	regulation of cell morphogenesis involved in differentiation	13/408	260/17913	0.006849442	0.039236182	0.028664704	6387/2316/2335/3037/4131/91663/4915/10630/91584/10631/5789/6383/7070	13
BP	GO:1903317	regulation of protein maturation	10/408	175/17913	0.006922798	0.039596306	0.02892978	715/716/3075/2619/85409/5627/5054/5270/10418/7057	10
BP	GO:0060421	positive regulation of heart growth	5/408	53/17913	0.007001666	0.039986823	0.029213099	2260/2627/7472/7490/23414	5
BP	GO:00	regulation of heart rate	7/408	98/179	0.00708	0.04038	0.02950	552/781/857/2702/5999/6330/7168	7

© 2020 Yu Y et al. *JAMA Network Open.*

	02027			13	2339	6447	5052		
BP	GO:0010464	regulation of mesenchymal cell proliferation	4/408	34/17913	0.007166833	0.040455628	0.029555593	2260/8660/5396/7472	4
BP	GO:0010574	regulation of vascular endothelial growth factor production	4/408	34/17913	0.007166833	0.040455628	0.029555593	1545/5743/23213/55959	4
BP	GO:0035909	aorta morphogenesis	4/408	34/17913	0.007166833	0.040455628	0.029555593	1281/2138/5159/7042	4
BP	GO:0043276	anoikis	4/408	34/17913	0.007166833	0.040455628	0.029555593	857/3678/4915/6591	4
BP	GO:0001508	action potential	8/408	123/17913	0.00716948 7	0.040455628	0.029555593	781/857/2316/2702/23704/3764/4915/6330	8
BP	GO:0001736	establishment of planar polarity	8/408	123/17913	0.007169487	0.040455628	0.029555593	115908/51339/2239/10082/144165/4919/4920/6423	8

© 2020 Yu Y et al. *JAMA Network Open.*

BP	GO:0007164	establishment of tissue polarity	8/408	123/17913	0.007169487	0.040455628	0.029555593	115908/51339/2239/10082/144165/4919/4920/6423	8
BP	GO:0030100	regulation of endocytosis	13/408	262/17913	0.007284884	0.041045426	0.029986481	9370/558/857/1012/1759/2042/2621/26585/3690/5587/5054/6424/84251	13
BP	GO:1903522	regulation of blood circulation	14/408	292/17913	0.007319917	0.04118135	0.030085783	10060/185/552/781/857/10699/2316/2702/5743/5999/6330/6781/7042/7168	14
BP	GO:0060977	coronary vasculature morphogenesis	3/408	18/17913	0.007421528	0.041505558	0.030322655	9464/5159/6444	3
BP	GO:0071371	cellular response to gonadotropin stimulus	3/408	18/17913	0.007421528	0.041505558	0.030322655	2627/3624/7490	3
BP	GO:0072311	glomerular epithelial cell differentiation	3/408	18/17913	0.007421528	0.041505558	0.030322655	9370/2303/7490	3
BP	GO:00	craniofacial suture morphogenesis	3/408	18/179	0.00742	0.04150	0.03032	2737/7043/7291	3

© 2020 Yu Y et al. *JAMA Network Open*.

	97094			13	1528	558	2655		
BP	GO:0021953	central nervous system neuron differentiation	10/408	177/17913	0.0074769 4	0.0417539 23	0.0305040 86	1009/2736/2737/3624/8828/4915/91584/6423/6695/7472	10
BP	GO:0002011	morphogenesis of an epithelial sheet	5/408	54/17913	0.0075752 6	0.0421778 87	0.0308138 21	1289/2316/3678/10630/90102	5
BP	GO:1904645	response to amyloid-beta	5/408	54/17913	0.0075752 6	0.0421778 87	0.0308138 21	773/781/1759/4313/4314	5
BP	GO:0045666	positive regulation of neuron differentiation	16/408	355/17913	0.0076640 4	0.0426093 52	0.0311290 35	1272/6387/1809/2042/2260/2335/2736/3082/4131/4915/5587/5789/5176/11075/55714/7077	16
BP	GO:0046545	development of primary female sexual characteristics	7/408	100/17913	0.0078862 52	0.0437802 97	0.0319844 9	658/1588/3624/4327/5156/7490/23414	7
BP	GO:0001893	maternal placenta development	4/408	35/17913	0.0079489 13	0.0439987 5	0.0321440 85	3976/5740/5743/6781	4

© 2020 Yu Y et al. *JAMA Network Open.*

BP	GO:0007157	heterophilic cell-cell adhesion via plasma membrane cell adhesion molecules	4/408	35/17913	0.007948913	0.04399875	0.032144085	1000/3678/5789/91179	4
BP	GO:1990845	adaptive thermogenesis	9/408	152/17913	0.008143279	0.045008608	0.032881855	9370/857/1734/2303/3953/4237/4883/56034/23090	9
BP	GO:0003229	ventricular cardiac muscle tissue development	5/408	55/17913	0.008180763	0.045083766	0.032936763	1301/2303/7042/7168/23414	5
BP	GO:0031102	neuron projection regeneration	5/408	55/17913	0.008180763	0.045083766	0.032936763	3082/4131/5396/7070/3371	5
BP	GO:0048708	astrocyte differentiation	6/408	77/17913	0.008194472	0.045093486	0.032943865	3976/4919/4920/5270/10100/7431	6
BP	GO:0001502	cartilage condensation	3/408	19/17913	0.008666148	0.047207372	0.034488202	658/1301/4920	3
BP	GO:00	regulation of systemic arterial blood pressure by circulatory	3/408	19/179	0.00866	0.04720	0.03448	1066/2702/5125	3

	01 99 1	renin-angiotensin		13	61 48	73 72	82 02		
BP	GO:0002544	chronic inflammatory response	3/408	19/17913	0.0086661 48	0.0472073 72	0.0344882 02	6356/1588/7057	3
BP	GO:0003215	cardiac right ventricle morphogenesis	3/408	19/17913	0.0086661 48	0.0472073 72	0.0344882 02	9464/7042/23414	3
BP	GO:0032891	negative regulation of organic acid transport	3/408	19/17913	0.0086661 48	0.0472073 72	0.0344882 02	8660/5999/7057	3
BP	GO:0072077	renal vesicle morphogenesis	3/408	19/17913	0.0086661 48	0.0472073 72	0.0344882 02	26585/55083/3976	3
BP	GO:0090280	positive regulation of calcium ion import	3/408	19/17913	0.0086661 48	0.0472073 72	0.0344882 02	6387/5159/6781	3
BP	GO:0001570	vasculogenesis	6/408	78/17913	0.0087116 21	0.0473185 16	0.03456 94	857/3037/4915/5159/7490/23414	6

© 2020 Yu Y et al. *JAMA Network Open*.

BP	GO:0055013	cardiac muscle cell development	6/408	78/17913	0.0087111621	0.0473185516	0.0345694	88/80206/5156/5159/5999/6444	6
BP	GO:0010573	vascular endothelial growth factor production	4/408	36/17913	0.0087849987	0.0475119227	0.0347107	1545/5743/23213/55959	4
BP	GO:0050982	detection of mechanical stimulus	4/408	36/17913	0.0087849987	0.0475119227	0.0347107	1301/6387/63895/5270	4
BP	GO:0086091	regulation of heart rate by cardiac conduction	4/408	36/17913	0.0087849987	0.0475119227	0.0347107	781/857/2702/6330	4
BP	GO:0086065	cell communication involved in cardiac conduction	5/408	56/17913	0.008819902	0.0476277753	0.0347953 19	781/857/2316/2702/6330	5
BP	GO:0046777	protein autophosphorylation	11/408	210/17913	0.008891014	0.0480511113	0.0351046 12	9370/857/4921/5167/2260/26585/4915/5156/5159/5587/7070	11
BP	GO:00	blood vessel endothelial cell migration	10/408	182/17	0.00901	0.04853	0.03545	2260/2303/26585/57608/4223/10769/5587/5743/27286/7057	10

	434534			913	2619	4434	7711		
BP	GO:0046394	carboxylic acid biosynthetic process	18/408	426/17913	0.009187094	0.049390819	0.036083359	9370/220/552/633/23743/1464/9420/1634/29940/92126/2687/3037/340895/4942/5352/5740/5743/1462	18
BP	GO:0021915	neural tube development	9/408	155/17913	0.009197813	0.049390819	0.036083359	115908/51339/2736/2737/144165/57167/6423/7042/7291	9
BP	GO:0032526	response to retinoic acid	7/408	103/17913	0.009215667	0.049392297	0.036089553	1277/2042/3490/5159/5176/3371/7472	7
BP	GO:0001708	cell fate specification	6/408	79/17913	0.009251736	0.049392297	0.036089553	2138/2260/2736/2737/6423/26011	6
BP	GO:0043154	negative regulation of cysteine-type endopeptidase activity involved in apoptotic process	6/408	79/17913	0.009251736	0.049392297	0.036089553	1410/2621/3082/5743/6423/7057	6
BP	GO:0055021	regulation of cardiac muscle tissue growth	6/408	79/17913	0.009251736	0.049392297	0.036089553	2260/2627/3776/5999/7472/23414	6

© 2020 Yu Y et al. *JAMA Network Open.*

CC	GO:0031012	extracellular matrix	103/419	468/18678	2.27E-73	7.62E-71	5.87E-71	81794/9509/9507/9370/165/23452/3730/54829/633/151887/57124/1012/1000/8483/1300/1301/1303/1306/1307/1277/1278/255631/1281/1289/1290/50509/1291/1292/1293/1295/1296/1311/83716/1464/115908/6387/1634/1805/1893/1842/10085/2202/11117/2162/2199/2200/23768/2331/2335/2239/10082/26585/83872/5654/3490/3956/4015/4016/4017/131578/4053/4060/4237/8076/4320/4327/4313/4314/25878/22795/255743/4958/5118/127435/10631/5549/10216/7837/5919/6383/5054/5270/5176/6423/6678/10418/8406/27286/284297/23213/7042/7043/7045/7057/7058/7060/7076/7077/7078/3371/63923/1462/7472	103
CC	GO:0062023	collagen-containing extracellular matrix	94/419	399/18678	1.31E-69	2.21E-67	1.70E-67	81794/9509/9507/9370/165/23452/54829/633/151887/1012/1000/8483/1300/1301/1303/1306/1307/1277/1278/255631/1281/1289/1290/50509/1291/1292/1293/1295/1296/1311/1464/115908/6387/1634/1805/1893/1842/10085/2202/11117/2162/2199/2200/2331/2335/2239/10082/26585/83872/5654/3490/3956/4016/4017/131578/4053/4060/4237/8076/4313/25878/22795/255743/4958/5118/127435/10631/5549/10216/7837/5919/6383/5054/5270/5176/6423/6678/10418/8406/27286/284297/23213/7042/7043/7045/7057/7058/7060/7076/7077/7078/3371/1462/7472	94
CC	GO:0005788	endoplasmic reticulum lumen	44/419	306/18678	5.09E-23	5.70E-21	4.39E-21	92949/340075/1000/1066/1300/1301/1303/1306/1307/1277/1278/255631/1281/1289/1290/50509/1291/1292/1293/1295/1296/56975/2200/60681/2326/2335/11167/2621/493869/3488/3490/3956/4148/54587/283208/56034/5743/7857/6383/10418/7057/7076/3371/1462	44
CC	GO:0005581	collagen trimer	26/419	86/18678	1.29E-22	1.08E-20	8.35E-21	9370/114897/1300/1301/1303/1306/1307/1277/1278/255631/1281/1289/1290/50509/1291/1292/1293/1295/1296/81035/115908/1634/11117/4015/4060/51435	26
CC	GO:0044420	extracellular matrix component	17/419	47/18678	9.90E-17	6.65E-15	5.13E-15	81794/1301/1303/1306/1277/1278/1281/1289/1290/50509/1295/11117/2200/4060/4237/8076/3371	17
CC	GO:0005604	basement membrane	20/419	91/18678	1.01E-14	5.63E-13	4.34E-13	151887/1306/1289/1295/1296/2200/2335/83872/4016/4017/22795/255743/5176/6678/7045/7058/7060/7076/7078/3371	20

© 2020 Yu Y et al. *JAMA Network Open.*

CC	GO:0005583	fibrillar collagen trimer	8/419	11/18678	9.34E-12	3.59E-10	2.77E-10	1301/1277/1278/1281/1289/1290/50509/4060	8
CC	GO:0098643	banded collagen fibril	8/419	11/18678	9.34E-12	3.59E-10	2.77E-10	1301/1277/1278/1281/1289/1290/50509/4060	8
CC	GO:0043292	contractile fiber	25/419	209/18678	9.62E-12	3.59E-10	2.77E-10	10060/59/88/113146/115557/800/1410/114907/10979/80206/2316/91156/57158/3764/4313/4620/10398/91624/255743/23022/27295/81493/23345/7168/7169	25
CC	GO:0031091	platelet alpha granule	17/419	91/18678	1.68E-11	5.65E-10	4.35E-10	88/2162/2335/2621/3082/3671/3690/5099/5627/5054/5270/6678/7042/7043/7057/7058/7076	17
CC	GO:0098644	complex of collagen trimers	9/419	18/18678	5.37E-11	1.64E-09	1.26E-09	1301/1277/1278/1281/1289/1290/50509/1295/4060	9
CC	GO:0044449	contractile fiber part	23/419	192/18678	6.41E-11	1.79E-09	1.38E-09	10060/59/88/113146/115557/1410/114907/10979/80206/2316/91156/57158/4313/4620/10398/91624/255743/23022/27295/81493/23345/7168/7169	23
CC	GO:00	myofibril	23/419	197/18	1.08E-1	2.80E-0	2.16E-0	10060/88/113146/115557/800/1410/114907/10979/80206/2316/91156/57158/3764/4313/4620/10398/91624/23022/27295/81493/23345/7168/7169	23

© 2020 Yu Y et al. *JAMA Network Open.*

	30016			678	0	9	9		
CC	GO:0030017	sarcomere	21/419	174/18678	3.82E-10	9.16E-09	7.06E-09	10060/88/113146/115557/1410/114907/10979/80206/2316/91156/57158/4313/4620/10398/91624/23022/27295/81493/23345/7168/7169	21
CC	GO:0031093	platelet alpha granule lumen	13/419	67/18678	2.56E-09	5.73E-08	4.42E-08	88/2162/2335/2621/3082/3671/5627/5054/6678/7042/7043/7057/7076	13
CC	GO:0005796	Golgi lumen	15/419	102/18678	8.41E-09	1.77E-07	1.36E-07	633/1464/1634/2331/2621/2239/10082/4060/4320/4958/5125/5549/5627/6383/1462	15
CC	GO:0043202	lysosomal lumen	13/419	94/18678	1.76E-07	3.47E-06	2.67E-06	633/1464/1513/1634/2331/2239/10082/4060/4958/5159/5549/6383/1462	13
CC	GO:0031674	I band	14/419	116/18678	3.33E-07	6.21E-06	4.79E-06	88/113146/1410/114907/10979/80206/2316/91156/57158/10398/91624/23022/27295/81493	14
CC	GO:0030018	Z disc	13/419	106/18678	7.27E-07	1.29E-05	9.91E-06	88/113146/1410/114907/80206/2316/91156/57158/10398/91624/23022/27295/81493	13

© 2020 Yu Y et al. *JAMA Network Open*.

CC	GO:0005925	focal adhesion	26/419	397/18678	1.18E-06	1.99E-05	1.53E-05	88/9590/857/1012/1000/1464/4921/2191/10979/2316/23768/22801/3678/3690/9902/91624/50507/23022/5159/90102/5328/91179/7070/3371/7431/165904	26
CC	GO:0005924	cell-substrate adherens junction	26/419	399/18678	1.30E-06	2.08E-05	1.60E-05	88/9590/857/1012/1000/1464/4921/2191/10979/2316/23768/22801/3678/3690/9902/91624/50507/23022/5159/90102/5328/91179/7070/3371/7431/165904	26
CC	GO:0030055	cell-substrate junction	26/419	404/18678	1.64E-06	2.50E-05	1.93E-05	88/9590/857/1012/1000/1464/4921/2191/10979/2316/23768/22801/3678/3690/9902/91624/50507/23022/5159/90102/5328/91179/7070/3371/7431/165904	26
CC	GO:0031252	cell leading edge	25/419	384/18678	2.12E-06	3.09E-05	2.38E-05	59/273/84168/1000/1464/1809/2191/10979/3678/3690/57608/24141/91663/23022/8572/10630/90102/130271/8910/23516/11075/7070/26230/7168/7431	25
CC	GO:0015629	actin cytoskeleton	26/419	432/18678	5.55E-06	7.77E-05	5.99E-05	59/88/273/794/800/1000/84952/10699/1410/4921/1809/10979/80206/2316/91663/4620/10398/50507/23022/27295/8572/130271/4919/7168/7169/165904	26
CC	GO:0031258	lamellipodium membrane	6/419	22/18678	6.76E-06	9.09E-05	7.01E-05	84168/1464/2191/10979/3690/10630	6
CC	GO:00	adherens junction	27/419	496/18	2.24E-0	0.00028	0.00022	88/9590/857/1012/1000/1464/4921/2191/10979/2316/23768/22801/3678/3690/57608/9902/91624/50507/23022/5159/90102/5328/91179/7070/3371/7431/165904	27

© 2020 Yu Y et al. *JAMA Network Open.*

	05912			678	5	98	3342		
CC	GO:0030027	lamellipodium	15/419	191/18678	2.81E-05	0.000349486	0.000269341	59/84168/1000/1464/1809/2191/10979/3690/23022/8572/10630/130271/23516/11075/26230	15
CC	GO:0031256	leading edge membrane	13/419	153/18678	4.23E-05	0.000507326	0.000390984	273/84168/1464/2191/10979/3678/3690/57608/24141/10630/8910/7070/7168	13
CC	GO:0001527	microfibril	4/419	10/18678	4.71E-05	0.000545681	0.000420543	81794/2200/4237/8076	4
CC	GO:0032432	actin filament bundle	8/419	59/18678	4.89E-05	0.000547367	0.000421843	1410/10979/10398/50507/8572/4919/7168/165904	8
CC	GO:0043235	receptor complex	18/419	292/18678	0.000113792	0.001233353	0.000950517	658/11117/2260/2550/2690/2892/22801/3678/3690/3953/84171/255743/8828/4915/93145/91584/5745/4919	18
CC	GO:0042383	sarcolemma	11/419	125/18678	0.000119125	0.001250812	0.000963972	113146/633/781/1000/1291/1292/1293/3764/8910/81493/7043	11

© 2020 Yu Y et al. *JAMA Network Open*.

CC	GO:0034774	secretory granule lumen	19/419	321/18678	0.000126044	0.00128 3359	0.00989055	88/83716/1893/2162/2335/2621/3082/3671/5122/5627/5919/5054/6678/7042/7043/7057/7076/7077/7078	19
CC	GO:0005775	vacuolar lumen	13/419	171/18678	0.000132144	0.00130 5897	0.01006424	633/1464/1513/1634/2331/2239/10082/4060/4958/5159/5549/6383/1462	13
CC	GO:0098797	plasma membrane protein complex	23/419	451/18678	0.000235242	0.00216 0886	0.01665344	10060/658/773/781/1000/11117/2121/132884/2550/2702/2743/2892/22801/3678/3690/3764/3776/255743/93145/6330/6444/8910/84251	23
CC	GO:0060205	cytoplasmic vesicle lumen	19/419	338/18678	0.000244124	0.00216 0886	0.01665344	88/83716/1893/2162/2335/2621/3082/3671/5122/5627/5919/5054/6678/7042/7043/7057/7076/7077/7078	19
CC	GO:0001725	stress fiber	7/419	56/18678	0.000244386	0.00216 0886	0.01665344	10979/10398/50507/8572/4919/7168/165904	7
CC	GO:0097517	contractile actin filament bundle	7/419	56/18678	0.000244386	0.00216 0886	0.01665344	10979/10398/50507/8572/4919/7168/165904	7
CC	GO:00	vesicle lumen	19/419	339/18	0.00025	0.00218	0.0168	88/83716/1893/2162/2335/2621/3082/3671/5122/5627/5919/5054/6678/7042/7043/7057/7076/7077/7078	19

© 2020 Yu Y et al. *JAMA Network Open*.

	31983			678	3392	3071	2442		
CC	GO:0008305	integrin complex	5/419	30/18678	0.0004975 7	0.0041795 85	0.0032211 08	11117/22801/3678/3690/255743	5
CC	GO:0042641	actomyosin	7/419	67/18678	0.0007420 85	0.0060814 78	0.0046868 54	10979/10398/50507/8572/4919/7168/165904	7
CC	GO:0098636	protein complex involved in cell adhesion	5/419	33/18678	0.0007841 85	0.0062734 8	0.0048348 25	11117/22801/3678/3690/255743	5
CC	GO:0031253	cell projection membrane	17/419	317/18678	0.0008651 02	0.0067598 68	0.0052096 73	84168/1464/79901/2121/132884/2191/10979/3678/3690/57608/24141/54587/10630/5745/8910/7070/7168	17
CC	GO:0005614	interstitial matrix	3/419	11/18678	0.0016172 61	0.0123499 96	0.0095178 54	151887/1842/3371	3
CC	GO:0030864	cortical actin cytoskeleton	6/419	57/18678	0.0016979 95	0.0126783 61	0.0097709 17	88/794/800/1000/91663/130271	6

© 2020 Yu Y et al. *JAMA Network Open*.

CC	GO:0030863	cortical cytoskeleton	7/419	81/18678	0.002271057	0.016588588	0.012784438	88/794/800/1000/2316/91663/130271	7
CC	GO:0033267	axon part	17/419	354/18678	0.002796878	0.019994699	0.015409449	1759/1809/3776/24141/4130/4131/4915/23022/5101/4919/5176/6695/11075/7070/26230/7077/63923	17
CC	GO:0031089	platelet dense granule lumen	3/419	14/18678	0.003393653	0.023755574	0.018307867	1893/5919/7078	3
CC	GO:0005865	striated muscle thin filament	3/419	16/18678	0.005050207	0.003462999	0.026688526	80206/7168/7169	3
CC	GO:0005938	cell cortex	12/419	229/18678	0.005735423	0.038542042	0.029703453	88/9590/794/800/857/1000/10979/2316/83872/91663/90102/130271	12
CC	GO:0031092	platelet alpha granule membrane	3/419	17/18678	0.006031429	0.039736473	0.030623974	3690/5099/6678	3
CC	GO:01	distal axon	13/419	262/18	0.00646	0.04176	0.03218	1809/3776/24141/4131/4915/23022/5101/4919/11075/7070/26230/7077/63923	13

© 2020 Yu Y et al. *JAMA Network Open*.

	50034			678	3889	6669	8599		
CC	GO:0031527	filopodium membrane	3/419	18/18678	0.007118699	0.04464609	0.034403701	84168/3690/10630	3
CC	GO:0016528	sarcoplasm	6/419	76/18678	0.007174433	0.04464609	0.034403701	781/57158/6444/6695/7057/7060	6
CC	GO:0098802	plasma membrane receptor complex	9/419	153/18678	0.007731346	0.047231498	0.036400215	658/11117/2550/2892/22801/3678/3690/255743/93145	9
CC	GO:0016324	apical plasma membrane	14/419	300/18678	0.008105826	0.047350887	0.036492225	55107/1000/1464/4921/2151/2335/23704/3776/50507/5159/10630/5745/6781/7070	14
CC	GO:0044448	cell cortex part	8/419	128/18678	0.008291754	0.047350887	0.036492225	88/794/800/1000/2316/91663/90102/130271	8
CC	GO:0031941	filamentous actin	3/419	19/18678	0.008314559	0.047350887	0.036492225	1809/2316/8572	3

© 2020 Yu Y et al. *JAMA Network Open*.

CC	GO:0036379	myofilament	3/419	19/18678	0.00831459	0.047350887	0.036492225	80206/7168/7169	3
MF	GO:0005201	extracellular matrix structural constituent	60/393	158/16969	2.54E-57	1.20E-54	9.88E-55	9370/165/3730/54829/633/8483/1300/1301/1303/1306/1307/1277/1278/255631/1281/1289/1290/50509/1291/1292/1293/1295/1296/1311/115908/1634/1805/1893/10085/2202/11117/2199/2200/2331/2335/83872/3490/4053/4060/4148/4237/8076/25878/22795/255743/5118/127435/10631/5549/10216/7837/6678/10418/8406/27286/7045/7057/7058/3371/1462	60
MF	GO:0005518	collagen binding	20/393	61/16969	3.19E-18	7.51E-16	6.20E-16	165/84168/54829/114897/50509/1311/1513/1634/4921/2335/22801/131578/4060/9902/22795/5118/127435/6678/7045/7057	20
MF	GO:0030020	extracellular matrix structural constituent conferring tensile strength	17/393	41/16969	1.03E-17	1.61E-15	1.33E-15	1300/1301/1303/1306/1307/1277/1278/255631/1281/1289/1290/50509/1291/1292/1293/1295/1296	17
MF	GO:0019838	growth factor binding	23/393	121/16969	5.38E-15	6.34E-13	5.23E-13	1277/1278/1281/1289/1291/2260/5654/94031/3488/3489/3490/3690/2615/4053/85409/8828/4915/5156/5159/7837/27286/7043/7057	23
MF	GO:0005178	integrin binding	22/393	113/16969	1.26E-14	1.19E-12	9.78E-13	88/1307/1281/1289/1311/6387/1842/10085/11117/2191/2200/2335/3678/3690/255743/6423/7045/7057/7060/7070/7077/63923	22
MF	GO:00	glycosaminoglycan binding	25/393	199/16	6.12E-1	4.81E-1	3.96E-1	3730/633/151887/3075/1301/1289/1311/83716/1634/1809/1833/2200/2260/2335/11167/4053/8828/5118/10631/5270/7057/7058/7060/7130/1462	25

© 2020 Yu Y et al. *JAMA Network Open.*

	055539			969	2	0	0		
MF	GO:0030021	extracellular matrix structural constituent conferring compression resistance	9/393	21/16969	4.02E-10	2.71E-08	2.23E-08	54829/633/1634/2331/4060/127435/5549/10216/1462	9
MF	GO:0048407	platelet-derived growth factor binding	7/393	11/16969	1.03E-09	6.08E-08	5.01E-08	1277/1278/1281/1289/1291/5156/5159	7
MF	GO:0008201	heparin binding	19/393	148/16969	1.52E-09	7.95E-08	6.55E-08	3730/151887/3075/1301/1289/1311/83716/2200/2260/2335/11167/4053/8828/5118/10631/5270/7057/7058/7060	19
MF	GO:0050840	extracellular matrix binding	10/393	43/16969	3.84E-08	1.81E-06	1.49E-06	633/1301/1634/1893/3690/3956/131578/284297/7045/7057	10
MF	GO:1901681	sulfur compound binding	21/393	221/16969	4.86E-08	2.08E-06	1.71E-06	3730/151887/3075/1301/1289/1311/83716/1809/5167/2200/2260/2335/11167/4053/8828/5118/10631/5270/7057/7058/7060	21
MF	GO:0004222	metalloendopeptidase activity	13/393	89/16969	1.29E-07	5.08E-06	4.19E-06	8038/81794/81792/140766/170690/170692/9509/9507/11174/2191/4327/4313/7092	13

© 2020 Yu Y et al. *JAMA Network Open*.

MF	GO:0004175	endopeptidase activity	24/393	322/16969	5.28E-07	1.87E-05	1.54E-05	8038/81794/81792/140766/170690/170692/9509/9507/11174/715/716/10699/1513/2191/5654/94031/9622/4327/4313/4314/5122/5125/5328/7092	24
MF	GO:0050839	cell adhesion molecule binding	29/393	444/16969	5.55E-07	1.87E-05	1.54E-05	88/800/1012/1307/1281/1289/1311/1363/6387/1842/10085/11117/2191/2200/2316/2335/3678/3690/255743/9379/90102/5789/6423/7045/7057/7060/7070/7077/63923	29
MF	GO:0001968	fibronectin binding	7/393	24/16969	8.34E-07	2.62E-05	2.16E-05	151887/1513/3690/131578/6423/284297/7057	7
MF	GO:0043394	proteoglycan binding	8/393	36/16969	1.32E-06	3.89E-05	3.21E-05	3075/1289/50509/1311/1513/2335/7057/3371	8
MF	GO:0004714	transmembrane receptor protein tyrosine kinase activity	9/393	49/16969	1.58E-06	4.39E-05	3.62E-05	4921/2202/2042/2260/8828/4915/5156/5159/5157	9
MF	GO:0019199	transmembrane receptor protein kinase activity	10/393	65/16969	2.28E-06	5.96E-05	4.92E-05	658/4921/2202/2042/2260/8828/4915/5156/5159/5157	10
MF	GO:00	platelet-derived growth factor receptor binding	5/393	14/169	1.09E-0	0.00027	0.00022	3678/3690/56034/5156/5159	5

© 2020 Yu Y et al. *JAMA Network Open.*

	05161			69	5	1339	3766		
MF	GO:0008237	metallopeptidase activity	14/393	153/16969	1.31E-05	0.000308889	0.000254734	8038/81794/81792/140766/170690/170692/9509/9507/11174/2191/4327/4313/4314/7092	14
MF	GO:0005520	insulin-like growth factor binding	6/393	26/16969	2.31E-05	0.000517834	0.000427043	5654/94031/3488/3489/3490/3690	6
MF	GO:0061134	peptidase regulator activity	15/393	192/16969	4.24E-05	0.000908759	0.000749429	3730/857/1293/1470/2335/2621/5118/5627/5054/5270/6423/6695/7076/7077/7078	15
MF	GO:0004252	serine-type endopeptidase activity	12/393	133/16969	6.21E-05	0.001272058	0.001049032	715/716/10699/2191/5654/94031/9622/4313/4314/5122/5125/5328	12
MF	GO:0019955	cytokine binding	10/393	100/16969	0.000107374	0.002042721	0.001684577	1311/9244/26585/3690/2615/8828/10630/7837/7043/7057	10
MF	GO:0004713	protein tyrosine kinase activity	11/393	120/16969	0.000108425	0.002042721	0.001684577	558/4921/2202/2042/2260/8828/4915/5156/5159/5157/91461	11

© 2020 Yu Y et al. *JAMA Network Open.*

MF	GO:0002020	protease binding	11/393	121/16969	0.000116837	0.002116542	0.001745456	9507/1277/1278/1281/1311/1893/2191/2335/3690/5054/7077	11
MF	GO:0008307	structural constituent of muscle	6/393	36/16969	0.000160626	0.002740919	0.002260363	88/10398/91624/27295/7168/7169	6
MF	GO:0005044	scavenger receptor activity	7/393	51/16969	0.000162942	0.002740919	0.002260363	81035/5167/4017/84171/10216/51435/284297	7
MF	GO:0046332	SMAD binding	8/393	72/16969	0.000252941	0.00410811	0.003387848	8313/658/1278/1281/1290/90993/2316/56937	8
MF	GO:0043236	laminin binding	5/393	26/16969	0.00028599	0.004490038	0.003702814	1893/3956/131578/284297/7057	5
MF	GO:0008236	serine-type peptidase activity	12/393	158/16969	0.000317972	0.00483112	0.003984095	715/716/10699/2191/5654/94031/9622/4313/4314/5122/5125/5328	12
MF	GO:00	Wnt-protein binding	5/393	27/169	0.00034	0.00506	0.00418	115908/4919/4920/6423/6424	5

	17147			69	4372	8722	0039		
MF	GO:0030545	receptor regulator activity	22/393	425/16969	0.000395997	0.005572604	0.004595577	9370/6356/9244/6387/2202/2200/23768/2621/26585/3082/3624/3976/56034/7837/7857/6423/6781/7042/7043/7060/7076/7472	22
MF	GO:0048018	receptor ligand activity	21/393	397/16969	0.000402269	0.005572604	0.004595577	9370/6356/9244/6387/2202/2200/23768/2621/26585/3082/3624/3976/56034/7857/6423/6781/7042/7043/7060/7076/7472	21
MF	GO:0017171	serine hydrolase activity	12/393	163/16969	0.000422465	0.00568517	0.004688407	715/716/10699/2191/5654/94031/9622/4313/4314/5122/5125/5328	12
MF	GO:0051015	actin filament binding	11/393	143/16969	0.000502785	0.006578102	0.005424784	88/84168/10979/2316/4620/91624/23345/6876/7168/7169/165904	11
MF	GO:0020037	heme binding	9/393	106/16969	0.000794655	0.010115743	0.008342181	1588/1545/9420/2982/125336/50507/5740/5743/7837	9
MF	GO:0005125	cytokine activity	11/393	154/16969	0.000933335	0.011271813	0.009295561	9370/6356/9244/6387/26585/3624/3976/7857/7042/7076/7472	11

© 2020 Yu Y et al. *JAMA Network Open.*

MF	GO:0061135	endopeptidase regulator activity	11/393	154/16969	0.000933335	0.011271813	0.009295561	3730/1293/1470/2621/5627/5054/5270/6423/6695/7076/7078	11
MF	GO:0030414	peptidase inhibitor activity	11/393	157/16969	0.001093057	0.012870742	0.010614155	3730/1293/1470/2621/5627/5054/5270/6695/7076/7077/7078	11
MF	GO:0046906	tetrapyrrole binding	9/393	116/16969	0.001507807	0.01732139	0.014284486	1588/1545/9420/2982/125336/50507/5740/5743/7837	9
MF	GO:0017046	peptide hormone binding	5/393	38/16969	0.001738025	0.01895988	0.015635704	552/2690/3624/4883/5745	5
MF	GO:0005172	vascular endothelial growth factor receptor binding	3/393	11/16969	0.001771199	0.01895988	0.015635704	26585/3678/3690	3
MF	GO:0097493	structural molecule activity conferring elasticity	3/393	11/16969	0.001771199	0.01895988	0.015635704	11117/2199/2200	3
MF	GO:00	growth factor receptor binding	9/393	121/16	0.00202	0.0214	0.01743	1893/2202/23768/26585/3678/3690/56034/5156/5159	9

© 2020 Yu Y et al. *JAMA Network Open*.

	70851			969	0332	6146	8659		
MF	GO:0015026	coreceptor activity	5/393	40/16969	0.002193945	0.021866222	0.018032486	2239/10082/3690/4919/4920	5
MF	GO:0008083	growth factor activity	10/393	147/16969	0.002260774	0.021866222	0.018032486	6387/2202/3082/3624/3976/56034/7042/7043/7060/7076	10
MF	GO:0030246	carbohydrate binding	14/393	252/16969	0.002310619	0.021866222	0.018032486	57124/1272/81035/5167/117248/11227/3956/4017/9902/10536/283208/5352/10216/1462	14
MF	GO:0004065	arylsulfatase activity	3/393	12/16969	0.002321255	0.021866222	0.018032486	340075/23213/55959	3
MF	GO:0032036	myosin heavy chain binding	3/393	12/16969	0.002321255	0.021866222	0.018032486	558/10398/85409	3
MF	GO:0004866	endopeptidase inhibitor activity	10/393	149/16969	0.002495785	0.02304931	0.019008147	3730/1293/1470/2621/5627/5054/5270/6695/7076/7078	10

© 2020 Yu Y et al. *JAMA Network Open.*

MF	GO:0042562	hormone binding	7/393	81/16969	0.002706301	0.024512843	0.020215084	220/552/1012/2690/3624/4883/5745	7
MF	GO:0038024	cargo receptor activity	7/393	85/16969	0.0035515 47	0.031561865	0.026028223	81035/5167/4017/84171/10216/51435/284297	7
MF	GO:0008191	metalloendopeptidase inhibitor activity	3/393	14/16969	0.0037107 49	0.032365981	0.026691355	6695/7076/7078	3
MF	GO:0008484	sulfuric ester hydrolase activity	3/393	15/16969	0.0045594 77	0.038348459	0.031624944	340075/23213/55959	3
MF	GO:0030169	low-density lipoprotein particle binding	3/393	15/16969	0.0045594 77	0.038348459	0.031624944	1012/81035/7057	3

© 2020 Yu Y et al. *JAMA Network Open*.
